# Biological diversity of the Minnesota caddisflies (Insecta, Trichoptera)

**DOI:** 10.3897/zookeys.189.2043

**Published:** 2012-05-03

**Authors:** David C. Houghton

**Affiliations:** 1 Department of Entomology, 1980 Folwell Ave., University of Minnesota, Saint Paul, MN 55108; 2 Department of Biology, Hillsdale College, 33 East College Street, Hillsdale, MI 49242

**Keywords:** Trichoptera, Minnesota, caddisfly, caddisflies, fauna, biodiversity, identification

## Abstract

The caddisfly fauna of Minnesota contains at least 277 species within 21 families and 75 genera. These species are based on examination of 312,884 specimens from 2,166 collections of 937 Minnesota aquatic habitats from 1890 to 2007. Included in these totals is my own quantitative sampling of 4 representative habitat types: small streams, medium rivers, large rivers, and lakes, from each of the 58 major Minnesota watersheds from June through September during 1999–2001. All species are illustrated herein, and their known Minnesota abundances, distributions, adult flight periodicities, and habitat affinities presented. Four species: *Lepidostoma griseum* (Lepidostomatidae), *Psilotreta indecisa* (Odontoceridae), and *Phryganea sayi* and *Ptilostomis angustipennis* (Phryganeidae) are added to the known fauna. An additional 31 dubious species records are removed for various reasons. Of the 5 determined caddisfly regions of the state, species richness per watershed was highest in the Lake Superior and Northern Regions, intermediate in the Southeastern, and lowest in the Northwestern and Southern. Of the 48 individual collections that yielded >40 species, all but 1 were from the Northern Region. Many species, especially within the families Limnephilidae and Phryganeidae, have appeared to decrease in distribution and abundance during the past 75 years, particularly those once common within the Northwestern and Southern Regions. Many species now appear regionally extirpated, and a few have disappeared from the entire state. The loss of species in the Northwestern and Southern Regions, and probably elsewhere, is almost certainly related to the conversion of many habitats to large-scale agriculture during the mid-20th century.

## Introduction

### Biodiversity research

Biological diversity research is necessary for an understanding of ecosystem ecology, organism conservation, and cladistic biogeography (Readka-Kudla et al. 1997, McKamey 1999, Mickevich 1999, Solis 1999). The conservation aspect of this type of research is becoming increasingly important due to a measured decline in worldwide organismal biodiversity and concern over the potential ecological implications of this decline (e.g., Readka-Kudla et al. 1997). Biodiversity databases include both organismal distribution data and the environmental data associated with such distributions. These data are crucial to proposing hypotheses on the factors contributing to organismal biodiversity, particularly changes in biodiversity over time (Mickevich 1999, Houghton and Holzenthal 2010). Documenting the biodiversity of insects is of particular importance due to the species richness of the group and the general lack of knowledge about insects relative to less diverse groups such as birds or mammals (Mickevich 1999, Strayer 2006, USFWS 2009, IUCN 2009).

Documenting the biodiversity of aquatic insects takes on yet an additional measure of importance due to the utility of the group in water quality biomonitoring. Freshwater resources continue to decline in the U.S. and elsewhere (e.g., Karr and Chu 1999, Shapiro et al. 2008). In the U.S., more than 70% of stream length is in “Fair” or “Poor” condition as determined by the Wadeable Streams Assessment; streams in the Plains and Lowlands region of the U.S. are in a similar condition (Paulsen et al. 2008). In biomonitoring, taxonomic data are collected from an aquatic habitat and combined with known information on the pollution tolerance, habitat affinity, and trophic functional group of individual taxa to assess potential habitat disturbances (e.g., Barbour et al. 1999, Karr and Chu 1999, Houghton 2006, Woodcock and Huryn 2007). Documenting these data for specific aquatic insect taxa in different areas, therefore, is necessary to refine water quality biomonitoring techniques.

### Overview of the caddisflies

The caddisflies (Trichoptera) are an order of holometabolous insects found on every continent except Antarctica. Larvae are aquatic and occupy virtually all types of freshwater ecosystems. There are currently approximately 15,000 species of caddisflies known from the world (Holzenthal et al. 2011) with many new species being described every year, primarily from the Neotropical and Oriental regions. Caddisflies are probably best known for their ability as larvae to produce silk from modified glands of the labium. This silk is used to attach together various combinations of mineral and organic materials, and construct portable cases and stationary retreats. These structures can be simple portable tubes, “saddle-cases” that superficially resemble tortoise shells, silken purses, fixed retreats with attached silken filter nets, and even helical cases that closely resemble snail shells (Wiggins 1996). The ability of the order to utilize silk to produce these structures is thought to be an important factor contributing to their ecological success, as it allows them to fill different niches (Mackay and Wiggins 1979). Caddisflies are important in aquatic ecosystems as secondary producers, cycling nutrients and being preyed upon by insectivorous fish and other animals (Wiggins and Mackay 1978, Ross and Wallace 1983, Robison and Buchanan 1988, Wiggins 1996).

Most adult caddisflies are nocturnally active during warm evenings throughout the summer and early fall, and the majority can be captured by attracting to ultraviolet lights (Houghton 2004a). Adults usually live for a few weeks. Individuals rarely fly more than 100 m from their natal stream, and mating and oviposition occur on or near the water (Sode and Wiberg-Larson 1993, Petersen et al. 1999, Sommerhäuser et al. 1999). Adults do not actively feed, although individuals may imbibe water or nectar using their haustellate mouthparts (Figure 294d).

Due to the taxonomic richness and ecological diversity of the caddisflies, along with their varying susceptibilities to pollution and abundance in virtually all freshwater ecosystems, the order has high potential value as a water quality biomonitoring taxon (Mackay and Wiggins 1979, Rosenberg and Resh 1993, Barbour et al. 1999, Dohet 2002, Houghton 2004a, b, Houghton 2007). The ability to predict specific caddisfly assemblages in specific aquatic ecosystems, therefore, will likely improve water quality biomonitoring techniques.

### Previous caddisfly taxonomic research

In most of the United States and adjacent Canadian provinces, caddisflies are either barely known, or known from only a basic species checklist. More comprehensive treatments of the Alabama (Harris et al. 1991), California (Denning 1956), Illinois (Ross 1944), New York (Betten 1934), and North and South Carolina (Unzicker et al. 1982) faunas provided good anecdotal information about the distributions and habitat affinities of the individual species within those areas. They do not, however, rigorously evaluate hypotheses on the important environmental variables affecting species distribution patterns in those regions. Likewise, they do not provide sufficient resources to identify collected specimens. Moulton and Stewart’s (1996) study of the caddisflies of the Interior Highlands of North America—primarily Arkansas and Missouri—assessed caddisfly distribution data with modern statistical methods, allowing for the prediction of individual species distributions based on environmental data, and also including an identification key. This study remains the most comprehensive faunal treatment of a caddisfly fauna in the U.S. Within the northcentral U.S. and southcentral Canada, basic species checklists are available for the Indiana (Waltz and McCafferty 1983), Manitoba (Flannagan and Flannagan 1982), Michigan (Leonard and Leonard 1949), Minnesota (Houghton et al. 2001), North Dakota (Harris et al. 1980), and Wisconsin (Longridge and Hilsenhoff 1973) faunas.

### Previous Minnesota caddisfly research

**Figure 1.**
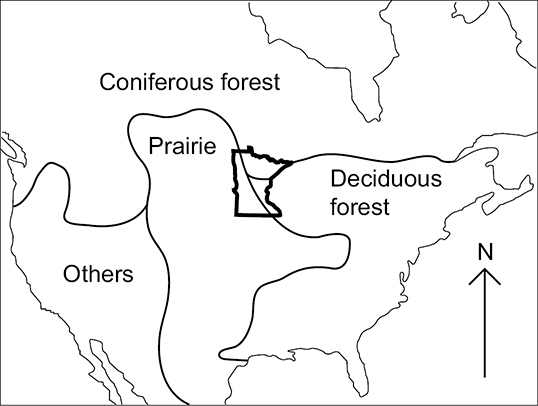
The USA and southern Canada showing the convergence of the Coniferous Forest, Deciduous Forest, and Prairie biotic provinces within the state of Minnesota (Bailey 1980). Others = miscellaneous biotic provinces.

The state of Minnesota is an ideal location to study caddisfly biological diversity. First, the state has an amazing wealth of freshwater resources, including nearly 12,000 natural lakes >4 ha in size, >100,000 km of streams and rivers, and nearly 4 million ha of wetlands (MNDNR 2009). Second, the state is situated on the intersection of the 3 largest biotic provinces of North America: Coniferous Forest, Deciduous Forest, and Prairie (Figure 1). Thus, findings from the state might be representative of large areas of the northcentral US and southcentral Canada. It is crucial, therefore, that the biodiversity of aquatic organisms such as caddisflies is understood so that science-based decisions can be made on water quality management in Minnesota.

**Figure 2.**
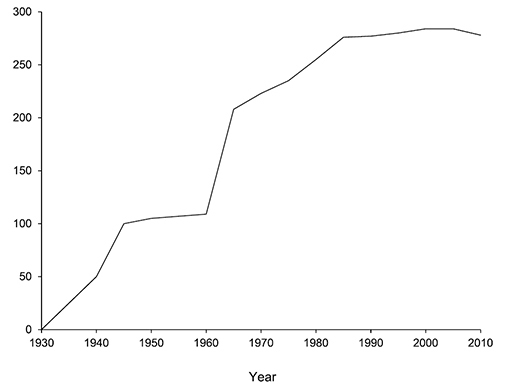
The progression of caddisfly species discovery in Minnesota. At least 31 other species have been reported from Minnesota, but are not included due to doubt about their identity or validity.

Prior to the 2000s, caddisfly taxonomic research in Minnesota was generally that of basic checklists and taxonomic revisions citing Minnesota records. Elkins (1936) published the first study of Minnesota caddisflies, documenting 31 species and hypothesizing that the fauna may include “at least 100 species”. Papers mainly by Ross (1938a, b, 1941 a, b, 1944, 1946, 1947, 1950, 1956) and Denning (1937, 1941, 1942, 1943, 1947a, b, c) in the middle part of the 1900s reported an additional 118 species. Etnier (1965) published the first checklist of the fauna, documenting 208 species. In the latter portion of the 1900s, regional taxonomic studies (Etnier 1968, Lager et al. 1979, Phillippi and Schuster 1987, MacLean 1995, Monson 1997), generic and familial revisions (Nimmo 1971, Morse 1972, Denning and Blickle 1972, Schuster and Etnier 1978, Blickle 1979, Nimmo 1986), and new species descriptions (Wiggins 1975, Monson and Holzenthal 1993, Sykora and Harris 1994) added an additional 48 species to the known fauna. Houghton et al. (2001) updated the checklist of Minnesota caddisflies, documenting a total of 284 species including 28 new state species records, and removing 21 doubtful species. The progression of species discovery in Minnesota is in Figure 2.

**Figure 3.**
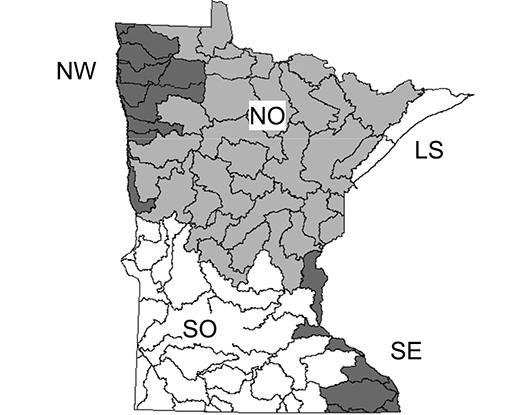
The 5 determined caddisfly regions based on species relative abundance (Houghton 2004a). LS: Lake Superior, NO: Northern, NW: Northwestern, SE: Southeastern, SO: Southern. Smaller regions are Minnesota major watersheds (USGS 2002).

In the early 2000s, a more comprehensive and quantitative approach to caddisfly faunistic research in Minnesota was undertaken. Houghton (2004a) representatively sampled adult caddisflies from 58 major watersheds using ultraviolet light traps, collecting >200,000 specimens. He used detrended correspondence analysis (DCA) and a flexible unweighted pair-group method using arithmetic averages (UPGMA) algorithm to group these watersheds into 5 “caddisfly regions” based on caddisfly relative abundance data (Figure 3). These regions each had a unique caddisfly fauna, and had more than double the classification strength of any *a priori* classification based on ecological data or primary watershed (Houghton 2003). Thus, they appear to be the most appropriate units for sampling caddisflies within Minnesota (e.g., Hawkins et al. 2000), as well as for predicting the geographic distribution of individual species within the state.

**Figure 4.**
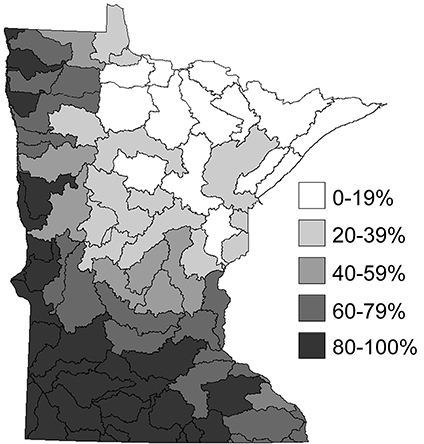
The relative level of disturbed habitat within the 58 major watersheds of Minnesota (USGS 1999). See Houghton (2007) for further explanation of how these disturbance levels were determined.

Houghton (2004a, 2006, 2007) also studied the correlations between caddisfly assemblages and landuse in Minnesota. He determined a significant negative correlation between both the overall caddisfly species richness and the abundance of shredder taxa with the level of disturbed habitat upstream of a sampling site, as well as a significant positive correlation between disturbed habitat and the relative abundance of pollution tolerant filtering collector species in a stream. Specifically, the hydropsychids *Cheumatopsyche campyla*, *Hydropsyche simulans*, and *Potamyia flava*—species normally found in large rivers—increased significantly in small and medium streams when these streams were impacted by organic pollution. This phenomenon was especially pronounced in the Northwestern and Southern Regions of Minnesota. In these regions (Figure 4), large-scale agriculture has modified the entire landscape to the point where nearly all aquatic ecosystems have become “homogenized”; that is, small and medium streams have taken on the characteristics of large rivers due to their increased load of fine particulate organic matter from agricultural runoff (Houghton 2007). These large-river characteristics in small and medium streams promote a caddisfly fauna resembling that of a large river, including a loss of species richness, a loss of shredder taxa, and an increase in filtering collector taxa.

Houghton and Holzenthal (2010) studied the conservation status of caddisflies in Minnesota, determining that the faunas of the Lake Superior, Northern, and Southeastern Regions remained fairly stable between the 1940s and the 1990s. The faunas of the Northwestern and Southern Regions, however, experienced a great deal of biodiversity loss, likely due to their conversion to large-scale agriculture during the interim. In particular, long-lived shredders in the Limnephilidae and Phryganeidae experienced the greatest level of extirpation in disturbed regions of the state, and now are rarely found in disturbed regions where they used to be abundant. In contrast, pollution tolerant filtering collectors in the Hydropsychidae now dominate the assemblages of these disturbed regions.

The general trends in caddisfly biological diversity within Minnesota are now as well known as anywhere in the U.S. The individual species, however, are not. The state still lacks a resource to identify individual species or predict their occurrence geographically and in different habitat types. The purpose of the current study, therefore, was to synthesize all known information about the individual Minnesota caddisflies into a single manual that allows for the identification of species, and the characterization of geographic range, adult flight periodicity, and habitat preference.

## Materials and methods

### Collecting and databasing

This study reflects all specimens stored in the University of Minnesota Insect Collection (UMSP), dating back to the 1890s. Adult collecting techniques have included malaise trapping, sweep netting, aspirating from riparian rocks and vegetation, and suspending several 8-W ultraviolet lights in front of a white sheet for 2 h after dusk, with subsequent capture in a cyanide kill jar. Many of D.G. Denning’s adult collections from the 1930s and 1940s occurred at the nighttime lights at gas stations in the middle of small towns such as Crookston, Hallock, and Finland. Larvae were collected by various means, transported alive back to the laboratory and reared to adult in either standard aquaria, or in a Living Stream (Frigid Units, Sylvania, OH), with approximated photoperiod, temperature, and flow regime of the particular habitat. Such a technique was especially important in obtaining adults of certain species of *Brachycentrus* (Brachycentridae) and *Glossosoma* (Glossosomatidae), which are often diurnal with a highly synchronous emergence.

From 1999 to 2001, I sampled the entire state representatively with light traps. For this technique, an 8-W ultraviolet light was set on top of a white enamel pan filled with 80% ethanol. Lights placed near aquatic habitats for 2 h after dusk attract most caddisfly species. For an in-depth discussion of this technique see Houghton (2004a). Traps were placed near at least 1 small stream, medium-sized river, large river, and lake or wetland for the 58 watersheds completely within Minnesota (Table 1, Figure 3). All watersheds were visited during the summer months of June and July, and again during September. Some collecting was done in August, although effort was less due to a typical lull in adult flight during this month.

**Table 1.** Four aquatic habitat classes and the total number of samples taken from each during 1999-2001 using ultraviolet light traps. Stream width was estimated at each sampling site.

**Class**	**Description**	**Width**	**n**
1	Small stream	<4 m	61
2	Medium river	4–15 m	81
3	Large river	>15 m	64
4	Lentic	N/A	69

**Figure 5.**
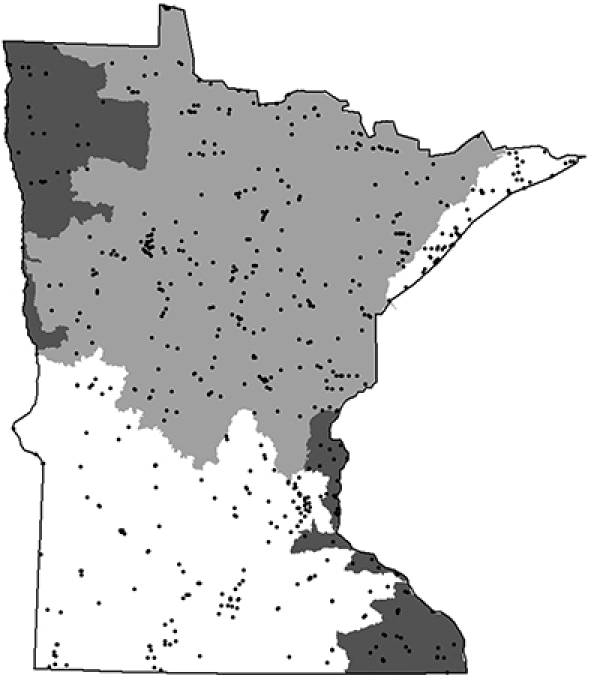
All known collecting localities associated with caddisfly specimens stored in the University of Minnesota Insect Museum.

A grand total of 312,884 specimens from 2166 collections of 937 total Minnesota localities (Figure 5) were entered into the UMSP BIOTA database (Colwell 2007), along with ecological information about each collecting site. A total of 24,167 specimens collected from the 1890s through the 1940s pre-dated the majority of habitat destruction in Minnesota (Omernik 1987, Tester 1995, Houghton and Holzenthal 2010) and allowed for comparisons between historical and contemporary species abundance and distributions. A total of 288,717 specimens collected since 1950 were also entered into the database. Over 97% of these “modern” specimens have been collected since 1999. All specimens analyzed during this study remain stored in the UMSP. Their locality data are accessible at http://www.entomology.umn.edu/museum/databases/.

### Preparation and illustration of specimens

Following the procedure originally described by Ross (1944), adult specimens were prepared for illustration by soaking the genitalic segments in a 10% potassium hydroxide solution overnight. This method dissolves the abdominal viscera and other organic material, leaving behind clear sclerites. Hydroptilids and other specimens <5 mm were cleared whole. For specimens >5 mm, only the abdomen was cleared. For some specimens, an abdominal ethanol injection using a hypodermic syringe was necessary to remove degraded viscera after clearing. For males of some families (e.g., Hydroptilidae and Limnephilidae), the phallus was gently extruded from the genital capsule for a more clear view.

Pencil sketches of specimens were made using a microscope with an ocular grid corresponding to a similar grid scale on graphing paper. Sketches were scanned into the computer program Adobe Illustrator™ for final illustration preparation using the procedure described by Holzenthal (2008). Many of the Hydroptilidae were re-drawn from previous illustrations by Ross (1938a, 1941a, 1944, 1947), Kelley (1984, 1985, 1986), or Kingsolver and Ross (1961).

## Results

### Overview of Minnesota caddisfly biodiversity

A total of 277 species are confirmed as occurring in Minnesota. These species are organized into 21 families and 75 genera. Four species: *Lepidostoma griseum* (Lepidostomatidae), *Psilotreta indecisa* (Odontoceridae), and *Phryganea sayi* and *Ptilostomis angustipennis* (Phryganeidae) are new additions to the state fauna since Houghton et al.’s (2001) checklist. *Psilotreta indecisa*, tentatively identified from larval sclerites, also represents a new genus and family record for the state. A total of 31 species is removed from the Minnesota fauna, mostly due to misidentifications, synonymies, *nomina dubia*, or an inability to locate the cited specimens. Due to these removals, fewer species are treated in this work than in the 2001 checklist (Figure 2). All dubious Minnesota species are listed in their respective genus chapters, including those already removed by Houghton et al. (2001).

**Figure 6.**
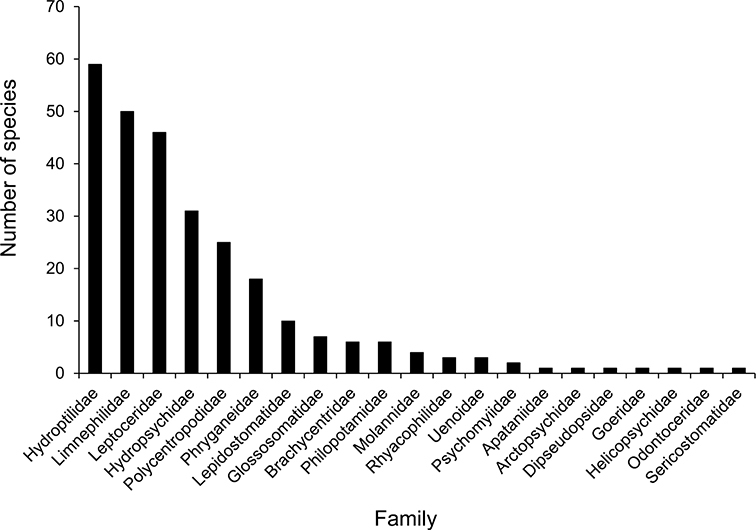
The total number of species known to occur in Minnesota for all of the Minnesota caddisfly families.

**Figure 7.**
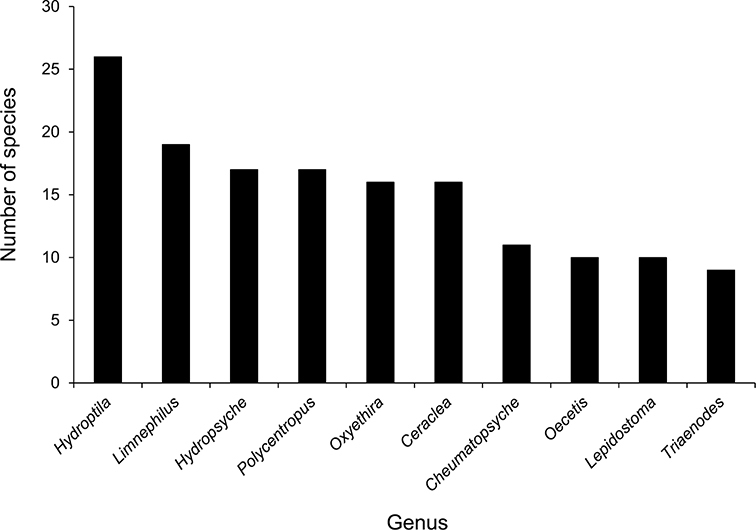
The 10 most species-rich genera in Minnesota.

**Figure 8.**
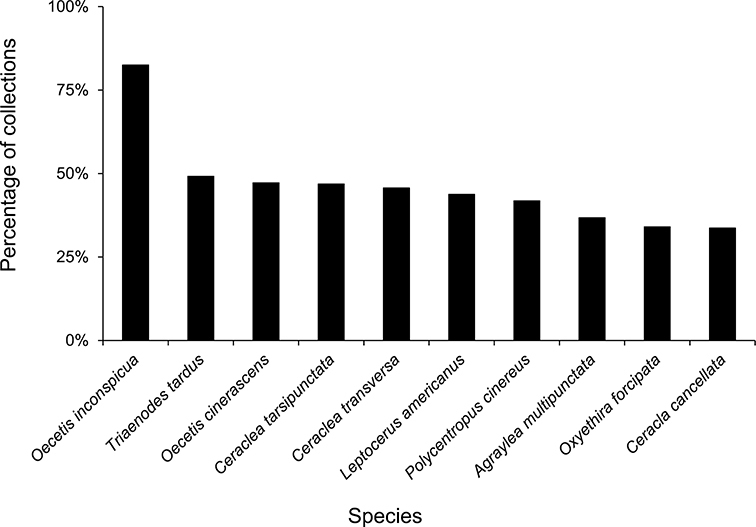
The 10 most widespread caddisfly species in Minnesota based on all specimens in the University of Minnesota Insect Collection.

**Figure 9.**
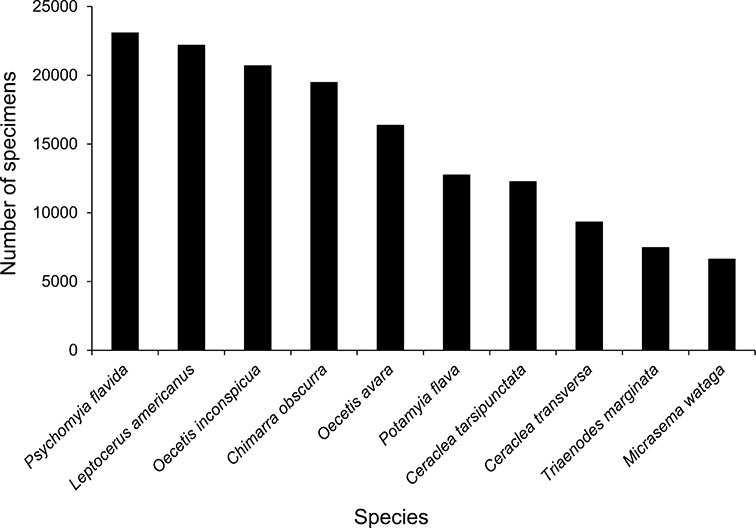
The 10 most abundant caddisfly species in Minnesota based on all specimens in the University of Minnesota Insect Collection.

**Figure 10.**
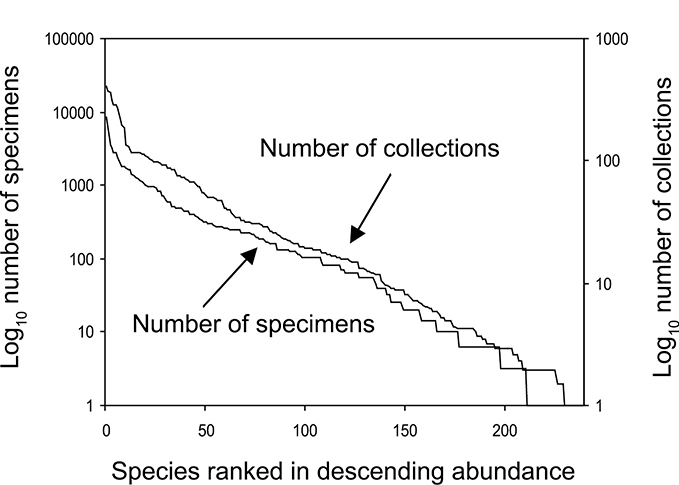
The number of caddisfly specimens known for each Minnesota species, and the number of collections in which each species has been found based on sampling during 1999-2001.

The families Hydroptilidae, Limnephilidae, and Leptoceridae collectively represented over half of the state fauna (Figure 6). Over 40% of the fauna was in 6 genera: *Hydroptila* and *Oxyethira* (Hydroptilidae), *Limnephilus* (Limnephilidae), *Hydropsyche* (Hydropsychidae), *Polycentropus* (Polycentropodidae), and *Ceraclea* (Leptoceridae) (Figure 7). *Oecetis inconspicua* (Leptoceridae) was, by far, the most widespread species in Minnesota, occurring at nearly 85% of all collecting localities in the state. In comparison, the 2nd most widespread species, *Triaenodes tarda* (Leptoceridae), occurred at <50% of all localities. Other widespread species are in Figure 8. *Psychomyia flavida* (Psychomyiidae) was the most abundant species based on all specimens collected, followed by *Leptocerus americanus* (Leptoceridae), and *Oecetis inconspicua* (Figure 9). The top 10 most abundant species represented >50% of all specimens examined. In contrast, almost 30% of the entire fauna has been found from <5 localities, and >25% of species are known from <10 specimens (Figure 10).

### Regional comparison

**Figure 11.**
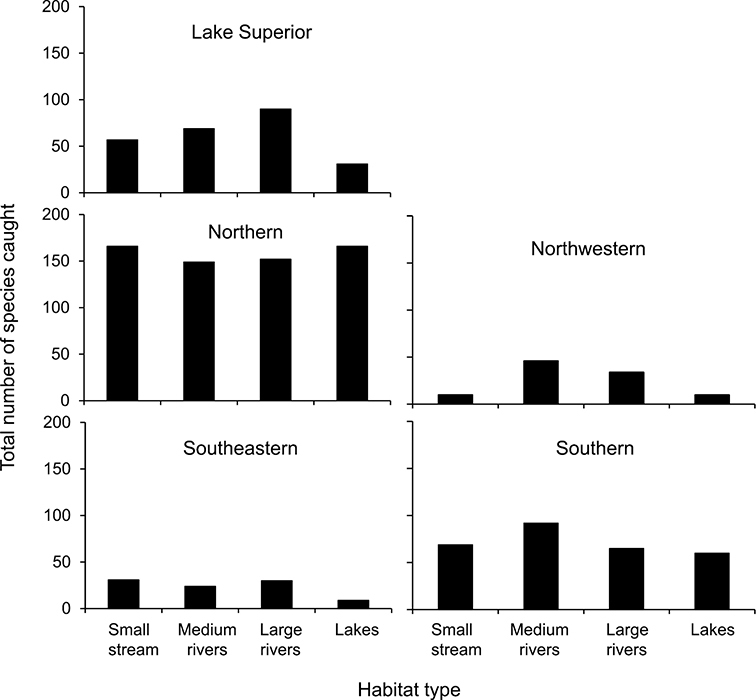
Total species richness for 4 defined habitat types within the 5 caddisfly regions (Table 1, Figure 3), based on all specimens stored in the UMSP.

Total species richness from the 4 different habitat types for the 5 caddisfly regions based on all historical collecting is in Figure 11. Based on the representative sampling during 1999–2001, the Northern Caddisfly Region had the highest total caddisfly species richness, followed by the Southern, Lake Superior, Southeastern, and Northwestern Regions (Table 2). The Lake Superior and Northern Regions had the highest mean species richness per watershed sampling unit, the Southern and Northwestern regions the lowest, and Southeastern had an intermediate mean (One-way Analysis of Variance with *post-hoc* Students-Neuman-Keuls test, *p* < 0.001) (Table 2). In the Lake Superior, Northern, and Southeastern Regions, more species were collected after 1980 than had been collected historically. The Northwestern and Southern Regions, however, have yielded fewer species since the 1980s.

**Table 2.** Summary statistics for 5 regions of Minnesota caddisfly biodiversity (Figure 4). Species per watershed was based on those from sampling during 1999-2001. Superscript letters denote statistically significant groupings based on a One-way Analysis of Variance with Student-Newman-Keuls test (*a* = 0.05). Unique species refer to those within Minnesota based on all historical and recent collecting, not true endemism.

**Region**	**Species prior to 1950**	**Species after 1980**	**Total species**	**Species/ watershed**	**Unique species**
Lake Superior	105	169	175	74^A^	15
Northern	205	219	231	73^A^	49
Southeastern	46	78	84	47^B^	6
Southern	148	110	152	31^C^	8
Northwestern	69	52	69	27^C^	3

### Particularly diverse areas

**Figure 12.**
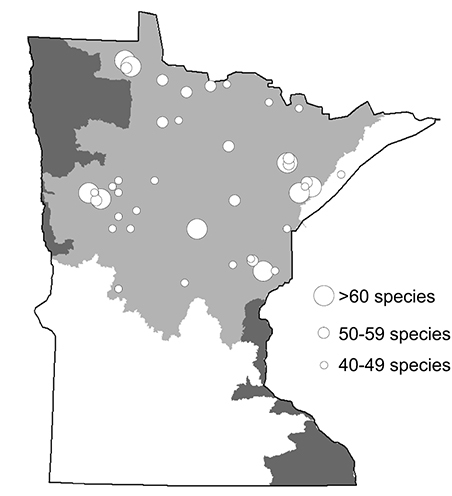
Individual collections from Minnesota during 1999-2001 that yielded >40 caddisfly species.

There were 48 individual collections that each yielded >40 species (Figure 12). All but 1 of these collections were from the Northern Region. A total of 9 collections yielded 60–69 species each, and 12 yielded 50–59 species. Based on these data, it appears that the most species-rich area of Minnesota is the Cloquet River watershed. Three sites within 30 km each yielded 60–69 species, 3 yielded 50–59, and 2 others yielded 40–49. These 8 sites collectively included 115 species collected over 2 nights during 2000—over 40% of total Minnesota caddisfly species richness. Other areas of high species richness include the upper Roseau River area (97 species) and the area around the White Earth Indian Reservation (78 species).

## Discussion

### Description of caddisfly regions

The Lake Superior Caddisfly Region encompasses almost 6,000 km^2^ and is composed of two areas draining directly into Lake Superior. It was originally composed of entirely Coniferous Forest, much of which has been replaced by deciduous forest stands (Stearns 1988). Although a few small towns and mining operations occur in this region, around 95% of the land area remains forested (Tester 1995, USGS 1999). Lakes are cold, deep, and oligotrophic, and many of the streams adjacent to Lake Superior are high gradient, containing numerous waterfalls (Heiskary and Wilson 1989, Tester 1995).

The Northern Caddisfly Region contains a total of 32 watersheds and over 100,000 km^2^. It is composed of mostly Coniferous Forest with a band of Deciduous Forest in its southern portion. The Northern Region contains approximately 85% of Minnesota’s natural lakes, most of which are small, deep, and oligotrophic (Heiskary and Wilson 1989, 1990). Most streams are low gradient and connect lakes (e.g., Tester 1995). Nearly 75% of the land area remains forested although much of the original coniferous forest has been replaced with early- to mid-succesional quaking aspen (*Populas tremuloides*) and paper birch (*Betula papyrifera*) forests (Stearns 1988, USGS 1999).

The Northwestern Caddisfly Region contains 10 watersheds and encompasses approximately 16,000 km^2^; all of its streams drain into the Red River of the North. It is composed approximately equally of Prairie and Coniferous Forest. This region is now dominated by agriculture, with around 82% of the land area under cultivation, and has had almost all of its prairie vegetation removed and lakes, wetlands, and small streams modified to accommodate this landuse practice (Waters 1977, Tester 1995, USGS 1999, Waters 2000). Virtually all aquatic habitats are low gradient medium to large rivers with high levels of sediments and nutrients (Stoner et al. 1998). The region experiences considerable flooding during the spring season of most years (e.g., Waters 2000).

The Southeastern Caddisfly Region is made up of eight watersheds and almost 10,000 km^t^, primarily composed of Deciduous Forest. It is semi-discontinuous, containing most of the watersheds draining into the lower Saint Croix and Mississippi Rivers. The region is dominated by streams and has virtually no natural lakes except in its extreme northern portion (Heiskary and Wilson 1989). Approximately 70% of the land area is under agricultural cultivation, although many of the valleys of small and medium rivers are protected by the State Park system (USGS 1999). Streams are spring fed, moderate–high gradient, and many support naturally reproducing stocks of brook trout (*Salvelinus fontinalis*) (e.g., Tester 1995).

The Southern region contains 29 watersheds and nearly 70,000 km^2^. It is composed of approximately equal amounts of Deciduous Forest and Prairie. As with the Northwestern Region, much of the natural vegetation and many of the lakes, wetlands, and small streams have been replaced with agriculture, which accounts for 85% of landuse (Tester 1995, USGS 1999). This region also includes nearly 70% of Minnesota’s human population, including the Twin Cities metropolitan area. Aquatic habitats are mostly low gradient medium to large rivers although some small streams remain, primarily in state parks. Existing lakes are almost entirely eutrophic or hypereutrophic (Heiskary and Wilson 1989, 1990).

### Biological diversity of caddisfly regions

Differences in regional caddisfly biodiversity appear to reflect both the natural and anthropogenic differences among habitats of the 5 determined regions. The Northwestern Region, for example, had the lowest total species richness of any region, reflecting specimens collected before much of the region was converted to large-scale agriculture. Indeed, several single collections from the Northern Region approximate the entire known fauna of the Northwestern Region from all historical collecting. The prairie habitats that dominate much of this region are low-gradient and with little heterogeneity relative to the forested habitats of the other regions. Further, only 3 species from all historical collecting: *Hydropsyche confusa* (Hydropsychidae),and *Anabolia sordida* and *Philarctus quaeris* (Limnephilidae) are unique to this region (Table 2), again suggesting a natural lack of habitat heterogeneity and species richness. Houghton and Holzenthal (2010) found, however, that the caddisfly fauna of this region prior to 1950 was similar to that of the Northern Region. The contemporary fauna, conversely, is most similar to the Southern Region, reflecting the low species richness of both regions. Further, more species were found prior to 1950 than after 1980, despite a far greater collection effort since 1980. Of the 3 unique northwestern species, 2 are now extirpated from the state. A similar situation occurs in the Southern Region: fewer species caught after 1980 than before 1950. In contrast, more species have been caught in the Lake Superior, Northern, and Southeastern Regions after 1980 than before 1950.

The most likely cause of the observed decrease in caddisfly species richness in the Northwestern and Southern Regions since the 1940s is large-scale agriculture. Intensive agriculture probably has the most extensive impact of any human land use on aquatic ecosystems (Allan 1995, Wang et al. 1997, Sponseller et al. 2001, Williams et al. 2003, Zimmerman et al. 2003, Allan 2004). Agriculture often leads to stream channelization, draining of wetlands, modification or loss of the surrounding floodplain, and removal of riparian canopy cover with subsequent loss of coarse allochthonous input (Gregory et al. 1991, Allan 1995, Delong and Brusven 1998, Quinn 2000, Brinson and Malvarez 2002). Agricultural runoff into aquatic habitats often contains large amounts of sediment and fine organic matter (Turner and Rabalais 1991, Zweig and Rabeni 2001). Collectively, these impacts promote homogenization of stream microhabitats and an increase in secondary production, especially in small to medium streams. Essentially, small streams develop the characteristics of large rivers (Delong and Brusven 1992, 1993, Pringle et al. 1993, Houghton 2007). Riparian disturbance with subsequent nutrient and sediment input was found to be the most widespread stressor of streams throughout the US (Paulsen at al. 2008). The Northwestern and Southern regions are dominated by agricultural land use, whereas much of the Northern, Lake Superior, and portions of the Southeastern region are forested (USGS 2002).

It is unlikely that the caddisfly faunas of the Lake Superior, Northern, and Southeastern regions are completely “natural”. Many of the watersheds throughout Minnesota that are now forested were previously logged or cultivated, with resulting loss of woody debris and sediment, and floodplain and channel modification, effects that can last for tens or hundreds of years (Perkins 1994, Bierley et al. 1999, Johnson et al. 2003, Allan 2004). How much effect historical disturbance has on contemporary biological diversity is not clear. In a study of Appalachian forests, Harding et al. (1998) found that the fauna of former agricultural landscapes was more similar to that of current agricultural landscapes than it was to that of primary forest. Wang et al. (2001) attributed differences in fish diversity along an urbanization gradient to the effects of prior agriculture along the stream. Conversely, Wang et al. (2003) found that anthropogenic disturbance was less important than natural features in predicting fish assemblages in the relatively undisturbed ecosystems of northern Michigan and Wisconsin, a landscape similar to that of northern Minnesota. Allan (2004) suggested that when human disturbance is ‘‘minor,’’ biological diversity is more affected by natural factors than by land use. The Lake Superior, Northern, and Southeastern Regions of Minnesota may not be completely natural—there is evidence that some regional extirpations have occurred (Houghton and Holzenthal 2010)—but they appear in obvious contrast to the Northwestern and Southern Regions.

### Refuge habitats

In some regions of Minnesota, both species richness and the degree to which the contemporary fauna appeared similar to that of historical fauna was influenced by the occurrence of “refuge” habitats: relatively undisturbed ecosystems within a large disturbed area. Even small forested areas can be important for maintaining aquatic biological diversity in a disturbed landscape (Houghton et al. 2011a). The land area of the Southeastern Region, for example, is over 70% disturbed (Figure 3–4). The state park system, however, protects the forested headwaters of several small and medium streams within the larger disturbed watersheds. Such refuge habitats were sampled during 1999–2001 and species recovery of this region was as expected. In contrast, it was difficult to locate refuge habitats within the Southern region, and impossible to locate them in the Northwestern region. Agricultural and small urban habitats dominated the entire landscape in these areas.

Refuge habitats also appear important for protecting species with limited distributions. For example, one of the only refuge habitats in the Southern Region, Minneopa Creek in Minneopa State Park, yielded 3 species: *Hydroptila rono* (Hydroptilidae), *Lepidostoma libum* (Lepidostomatidae), and *Oecetis ditissa* (*Leptoceridae*), found nowhere else in Minnesota, as well as populations of rare species such as *Diplectrona modesta* (Hydropsychidae) and *Pseudostenophylax sparsus* (Limnephilidae). Two of the new state records reported in this monograph: *Lepidostoma griseum* (Lepidostomatidae) and *Ptilostomis angustipennis* (Phryganeidae), were found during the same collection of a first-order intermittent stream, Mill Creek in William O’Brien State Park in the Southeastern Region, a site < 1 hour’s drive from the Twin Cities metropolitan area. The same stream also yielded the only known specimens of *Parapsyche apicalis* (Arctopsychidae) ever collected in Minnesota. Only 8 caddisfly species total have been found in this stream. Thus, nearly 40% of the caddisfly fauna of Mill Creek has been found nowhere else in the state. Houghton et al. (2011b) similarly found 8 new species records from a first-order stream in Michigan. Refuge habitats, especially small streams with low species richness, are easy to overlook, but these observations indicate their importance in protecting biological diversity.

### Future research

Despite a collecting history of >100 years, and an asymptotic species-sample surve, it is possible that additional species remain undiscovered in Minnesota. Most of these species will probably be found in novel habitats, such as intermittent streams, vernal pools, or wetlands. Refuge habitats within disturbed landscapes may also yield new records. Efforts are ongoing to locate adults of *Oligostomis* (Phryganeidae) and *Psilotreta* (Odontoceridae), which are currently only known from larvae, and to find additional populations of rare endemic species, such as *Chilostigma itascae* and *Polycentropus milaca*. The caddisfly fauna of Minnesota is now as well-known as the fauna of any other area of North American. With good baseline data now in place, any future changes to the fauna can be evaluated with greater confidence and precision.

### Identification manual explanation

This manual intentionally avoids the use of dichotomous keys and detailed taxonomic descriptions for species identification. Instead, it relies on the premise that a collector is more likely to encounter a common species than a rare species. Descriptions of the 21 known families are listed in the order of collection likelihood on page 27. Common and abundant species for the 4 different habitat types of each of the 5 caddisfly regions are summarized in Tables 3–7. For example, 95% of all specimens collected from large rivers of the Northwestern Region are represented by the 7 species listed in Table 5. It is, obviously, recommended that users examine the illustrations of the listed species first. Taxonomic keys to Minnesota caddisfly families and genera can be found in Morse and Holzenthal (2008), Schmid (1998), Wiggins (2004, 2008), and references cited throughout this manual.

All species are organized alphabetically by family and genus. Each species plate includes illustrations of the important identification characteristics. For most species, this means a lateral view of the genital capsule and phallus of the male. Other male illustrations may include additional views of the genital capsule, or specific views of the inferior appendages, tergum X, or other characteristics necessary for identifying the species. Most female caddisflies are not readily identifiable. For species that can be identified (e.g., most limnephilids and psychomyiids), a lateral or ventral view of the female genital capsule is included.

Each species plate also includes a distribution map which reflects the total number of specimens and all collecting localities known to yield those specimens from all historical and recent collecting. An explanation is given in the text for species that appear to have been reduced from a portion of their known historical range. Lastly, each species plate includes graphs reflecting the adult flight period and habitat preferences of the species. These graphs were based only on collections since 1980. Many historical collections were from cities not associated with any particular habitat, rendering inclusion into habitat preference graphs impossible. Most specimens collected since 1980 were obtained using quantitative sampling techniques, thus allowing users to accurately compare contemporary abundances of the different species in different habitats. Users can also compare the number of specimens collected since 1980 with the total number collected. For some species, the decrease in abundance and distribution since the 1940s is quite striking. Ideally, viewing all historical collecting localities in conjunction with recent quantitative habitat preference data, and reading the text for each species, should give the best predictive information on where each species should be expected to occur in Minnesota.

**Table 3.** Species found in ≥ 50% of respective habitats of the **Lake Superior** Caddisfly Region (Figure 3) since 1980 and representing ≥ 1% of total specimen abundance in each habitat (Table 1). Species richness totals in Figure 11.

**Habitat**	**Species**	**Family**	**% of fauna**	**Figure**
Small streams	*Hydropsyche sparna*	Hydropsychidae	17%	58
	*Hydropsyche slossonae*	Hydropsychidae	10%	57
	*Dolophilodes distinctus*	Philopotamidae	9%	236
	*Molanna blenda*	Molannidae	9%	227
	*Hydroptila valhalla*	Hydroptilidae	8%	85
	*Nyctiophylax moestus*	Polycentropodidae	7%	263
	*Lepidostoma togatum*	Lepidostomatidae	4%	130
	*Ceraclea transversa*	Leptoceridae	3%	146
	*Ptilostomis semifasciata*	Phryganeidae	2%	255
	*Oecetis inconspicua*	Leptoceridae	2%	160
	*Banksiola crotchi*	Phryganeidae	1%	244
	*Glossosoma nigrior*	Glossosomatidae	1%	26
Medium rivers	*Lepidostoma togatum*	Lepidostomatidae	32%	130
	*Ceraclea transversa*	Leptoceridae	11%	146
	*Ceraclea cancellata*	Leptoceridae	9%	138
	*Cheumatopsyche gracilis*	Hydropsychidae	7%	34
	*Hydroptila valhalla*	Hydroptilidae	7%	85
	*Hydropsyche slossonae*	Hydropsychidae	5%	57
	*Hydropsyche walkeri*	Hydropsychidae	4%	59
	*Hydropsyche sparna*	Hydropsychidae	2%	58
	*Banksiola crotchi*	Phryganeidae	2%	244
	*Glossosoma nigrior*	Glossosomatidae	2%	26
	*Nyctiophylax moestus*	Polycentropodidae	2%	263
	*Hydropsyche dicantha*	Hydropsychidae	2%	50
	*Helicopsyche borealis*	Helicopsychidae	1%	31
	*Polycentropus cinereus*	Polycentropodidae	1%	267
	*Oecetis inconspicua*	Leptoceridae	1%	160
	*Dolophilodes distinctus*	Philopotamidae	1%	236
	*Rhyacophila fuscula*	Rhyacophilidae	1%	284
Large rivers	*Ceraclea transversa*	Leptoceridae	18%	146
	*Chimarra socia*	Philopotamidae	14%	235
	*Lepidostoma togatum*	Lepidostomatidae	10%	130
	*Leptocerus americanus*	Leptoceridae	9%	148
	*Chimarra obscurra*	Philopotamidae	8%	234
	*Oecetis avara*	Leptoceridae	5%	155
	*Psychomia flavida*	Psychomyiidae	5%	282
	*Hydroptila valhalla*	Hydroptilidae	4%	85
	*Ceraclea diluta*	Leptoceridae	4%	139
	*Ceraclea resurgens*	Leptoceridae	2%	144
	*Rhyacophila fuscula*	Rhyacophilidae	2%	284
	*Ceraclea cancellata*	Leptoceridae	2%	138
	*Glossosoma nigrior*	Glossosomatidae	2%	26
	*Oecetis inconspicua*	Leptoceridae	1%	160
Lakes	*Triaenodes injustus*	Leptoceridae	38%	172
	*Polycentropus cinereus*	Polycentropodidae	27%	267
	*Molanna flavicornis*	Molannidae	8%	228
	*Nyctiophylax affinis*	Polycentropodidae	5%	260
	*Oecetis inconspicua*	Leptoceridae	2%	160
	*Phryganea cinerea*	Phryganeidae	2%	251
	*Molanna uniophila*	Molannidae	2%	230
	*Ceraclea cancellata*	Leptoceridae	2%	138
	*Phylocentropus placidus*	Dipseudopsidae	1%	22

**Table 4.** Species found in ≥ 50% of respective habitats of the **Northern** Caddisfly Region (Figure 3) since 1980 and representing ≥ 1% of total specimen abundance in each habitat (Table 1). Species richness totals in Figure 11.

**Habitat**	**Species**	**Family**	**% of fauna**	**Figure**
Small streams	*Leptocerus americanus*	Leptoceridae	16%	148
	*Oecetis inconspicua*	Leptoceridae	14%	160
	*Ceraclea transversa*	Leptoceridae	9%	146
	*Oxyethira forcipata*	Hydroptilidae	6%	109
	*Triaenodes marginata*	Leptoceridae	4%	173
	*Banksiola crotchi*	Phryganeidae	1%	244
	*Triaenodes tarda*	Leptoceridae	1%	175
	*Cheumatopsyche pettiti*	Hydropsychidae	1%	39
Medium rivers	*Chimarra obscurra*	Philopotamidae	22%	234
	*Oecetis avara*	Leptoceridae	10%	155
	*Ceraclea transversa*	Leptoceridae	6%	146
	*Oecetis persimilis*	Leptoceridae	4%	164
	*Leptocerus americanus*	Leptoceridae	3%	148
	*Oecetis inconspicua*	Leptoceridae	3%	160
	*Triaenodes marginata*	Leptoceridae	1%	174
	*Oxyethira forcipata*	Hydroptilidae	1%	109
Large rivers	*Psychomyia flavida*	Psychomyiidae	20%	282
	*Oecetis avara*	Leptoceridae	14%	155
	*Chimarra obscurra*	Philopotamidae	13%	234
	*Ceraclea tarsipunctata*	Leptoceridae	9%	145
	*Cheumatopsyche speciosa*	Hydropsychidae	6%	41
	*Leptocerus americanus*	Leptoceridae	4%	148
	*Ceraclea transversa*	Leptoceridae	3%	146
	*Oecetis inconspicua*	Leptoceridae	3%	160
	*Helicopsyche borealis*	Helicopsychidae	3%	31
	*Lepidostoma togatum*	Lepidostomatidae	2%	130
	*Triaenodes injustus*	Leptoceridae	1%	173
	*Ceraclea cancellata*	Leptoceridae	1%	138
	*Oecetis persimilis*	Leptoceridae	1%	164
	*Triaenodes marginata*	Leptoceridae	1%	174
	*Agraylea multipunctata*	Hydroptilidae	1%	63
	*Cheumatopsyche campyla*	Hydropsychidae	1%	33
	*Oecetis cinerascens*	Leptoceridae	1%	156
	*Triaenodes tarda*	Leptoceridae	1%	175
Lakes	*Leptocerus americanus*	Leptoceridae	17%	148
	*Oecetis inconcpicua*	Leptoceridae	11%	160
	*Oecetis osteni*	Leptoceridae	7%	163
	*Mystacides interjecta*	Leptoceridae	5%	149
	*Ceraclea tarsipunctata*	Leptoceridae	4%	145
	*Oecetis cinerascens*	Leptoceridae	3%	156
	*Triaenodes injustus*	Leptoceridae	3%	172
	*Molanna flavicornis*	Molannidae	3%	228
	*Molanna uniophila*	Molannidae	2%	230
	*Agraylea multipunctata*	Hydroptilidae	2%	63
	*Ceraclea cancellata*	Leptoceridae	2%	138
	*Ceraclea transversa*	Leptoceridae	1%	146
	*Banksiola crotchi*	Phryganeidae	1%	244
	*Oxyethira forcipata*	Hydroptilidae	1%	109

**Table 5.** Species found in ≥ 50% of respective habitats of the **Northwestern** Caddisfly Region (Figure 3) since 1980 and representing ≥ 1% of total specimen abundance in each habitat (Table 1). Species richness totals in Figure 11.

**Habitat**	**Species**	**Family**	**% of fauna**	**Figure**
Small streams	*Hydropsyche bidens*	Hydropsychidae	33%	47
	*Cheumatopsyche speciosa*	Hydropsychidae	17%	41
	*Potamyia flava*	Hydropsychidae	17%	62
	*Cheumatopsyche campyla*	Hydropsychidae	10%	33
	*Hydropsyche simulans*	Hydropsychidae	6%	56
Medium rivers	*Potamyia flava*	Hydropsychidae	37%	62
	*Agraylea multipunctata*	Hydroptilidae	24%	63
	*Cheumatopsyche speciosa*	Hydropsychidae	8%	41
	*Oecetis inconspicua*	Leptoceridae	6%	160
	*Ceraclea alagma*	Leptoceridae	5%	132
	*Cheumatopsyche campyla*	Hydropsychidae	3%	33
	*Cheumatopsyche pettiti*	Hydropsychidae	2%	39
	*Hydropsyche bidens*	Hydropsychidae	2%	47
	*Hydropsyche simulans*	Hydropsychidae	1%	56
Large rivers	*Potamyia flava*	Hydropsychidae	32%	62
	*Hydropsyche confusa*	Hydropsychidae	31%	49
	*Cheumatopsyche speciosa*	Hydropsychidae	14%	41
	*Hydropsyche bidens*	Hydropsychidae	9%	47
	*Hydropsyche simulans*	Hydropsychidae	6%	56
	*Cheumatopsyche campyla*	Hydropsychidae	2%	33
	*Oecetis inconspicua*	Leptoceridae	1%	160
Lakes	*Oecetis inconspicua*	Leptoceridae	85%	160
	*Ceraclea alagma*	Leptoceridae	3%	132
	*Polycentropus cinereus*	Polycentropodidae	2%	267
	*Cheumatopsyche campyla*	Hydropsychidae	1%	33

**Table 6.** Species found in ≥ 50% of respective habitats of the **Southeastern** Caddisfly Region (Figure 3) since 1980 and representing ≥ 1% of total specimen abundance in each habitat (Table 1). Species richness totals in Figure 11.

**Habitat**	**Species**	**Family**	**% of fauna**	**Figure**
Small streams	*Brachycentrus americanus*	Brachycentridae	38%	16
	*Hydropsyche alhedra*	Hydropsychidae	18%	44
	*Hydropsyche slossonae*	Hydropsychidae	12%	57
	*Glossosoma intermedium*	Glossosomatidae	4%	25
	*Hydroptila consimilis*	Hydroptilidae	4%	72
	*Micrasema gelidum*	Brachycentridae	3%	19
	*Hesperophylax designatus*	Limnephilidae	2%	190
Medium rivers	*Ceraclea tarsipunctata*	Leptoceridae	49%	145
	*Brachycentrus americanus*	Brachycentridae	17%	16
	*Triaenodes tarda*	Leptoceridae	9%	176
	*Hydropsyche slossonae*	Hydropsychidae	5%	57
	*Hydropsyche betteni*	Hydropsychidae	3%	46
	*Hydropsyche alhedra*	Hydropsychidae	2%	44
	*Glossosoma intermedium*	Glossosomatidae	1%	25
Large rivers	*Ceraclea tarsipunctata*	Leptoceridae	45%	145
	*Cheumatopsyche pasella*	Hydropsychidae	12%	38
	*Hydropsyche alhedra*	Hydropsychidae	10%	44
	*Potamyia flava*	Hydropsychidae	5%	62
	*Hydropsyche bidens*	Hydropsychidae	5%	47
	*Psychomyia flavida*	Psychomyiidae	4%	282
	*Ceraclea maculata*	Leptoceridae	4%	142
	*Leptocerus americanus*	Leptoceridae	3%	148
	*Cheumatopsyche campyla*	Hydropsychidae	3%	33
Lakes	*Hydroptila waubesiana*	Hydroptilidae	21%	87
	*Leptocerus americanus*	Leptoceridae	18%	148
	*Ceraclea alagma*	Leptoceridae	14%	132
	*Oecetis inconspicua*	Leptoceridae	14%	160
	*Triaenodes tarda*	Leptoceridae	14%	176
	*Polycentropus cinereus*	Polycentropodidae	7%	267

**Table 7.** Species found in ≥ 50% of respective habitats of the **Southern** Caddisfly Region (Figure 3) since 1980 and representing ≥ 1% of total specimen abundance in each habitat (Table 1). Species richness totals in Figure 11.

**Habitat**	**Species**	**Family**	**% of fauna**	**Figure**
Small streams	*Potamyia flava*	Hydropsychidae	28%	62
	*Hydropsyche simulans*	Hydrpopsychidae	14%	56
	*Oecetis inconcpicua*	Leptoceridae	6%	160
	*Hydropsyche morosa*	Hydropsychidae	6%	51
	*Cheumatopsyche pettiti*	Hydropsychidae	4%	39
	*Hydropsyche betteni*	Hydropsychidae	3%	46
	*Cheumatopsyche campyla*	Hydropsychidae	2%	33
	*Oecetis cinerascens*	Leptoceridae	1%	156
	*Triaenodes tarda*	Leptoceridae	1%	176
	*Ceraclea maculata*	Leptoceridae	1%	142
Medium rivers	*Potamyia flava*	Hydropsychidae	41%	62
	*Psychomyia flavida*	Psychomyiidae	18%	282
	*Hydropsyche simulans*	Hydropsychidae	4%	56
	*Cheumatopsyche campyla*	Hydropsychidae	3%	33
	*Hydropsyche morosa*	Hydropsychidae	3%	51
	*Oecetis inconcpicua*	Leptoceridae	2%	160
	*Cheumatopsyche aphanta*	Hydropsychidae	2%	32
	*Hydroptila consimilis*	Hydropsychidae	1%	72
	*Oecetis cinerascens*	Leptoceridae	1%	156
Large rivers	*Potamyia flava*	Hydropsychidae	31%	62
	*Ceraclea tarsipunctata*	Leptoceridae	2%	145
	*Hydropsyche simulans*	Hydropsychidae	1%	56
	*Psychomyia flavida*	Psychomyiidae	1%	282
	*Oecetis inconspicua*	Leptoceridae	1%	160
	*Hydropsyche morosa*	Hydropsychidae	1%	51
	*Oecetis avara*	Leptoceridae	1%	155
	*Cheumatopsyche campyla*	Hydropsychidae	1%	33
	*Oecetis cinerascens*	Leptoceridae	1%	156
	*Agraylea multipunctata*	Hydroptilidae	1%	63
Lakes	*Leptocerus americanus*	Leptoceridae	30%	148
	*Potamyia flava*	Hydropsychidae	19%	62
	*Mystacides interjecta*	Leptoceridae	7%	150
	*Oecetis inconspicua*	Leptoceridae	6%	160
	*Polycentropus cinereus*	Polycentropodidae	5%	267
	*Ceraclea tarsipunctata*	Leptoceridae	3%	145
	*Oecetis cinerascens*	Leptoceridae	3%	156
	*Agraylea multipunctata*	Hydrpoptilidae	2%	63

## Family diagnoses

The caddisfly families of Minnesota are ranked below based on their likelihood of being encountered by a general collector. The most useful diagnostic characters for each family are also listed, as well as general distribution and abundance information, and how to differentiate each family from similar families.

**Figure 13.**
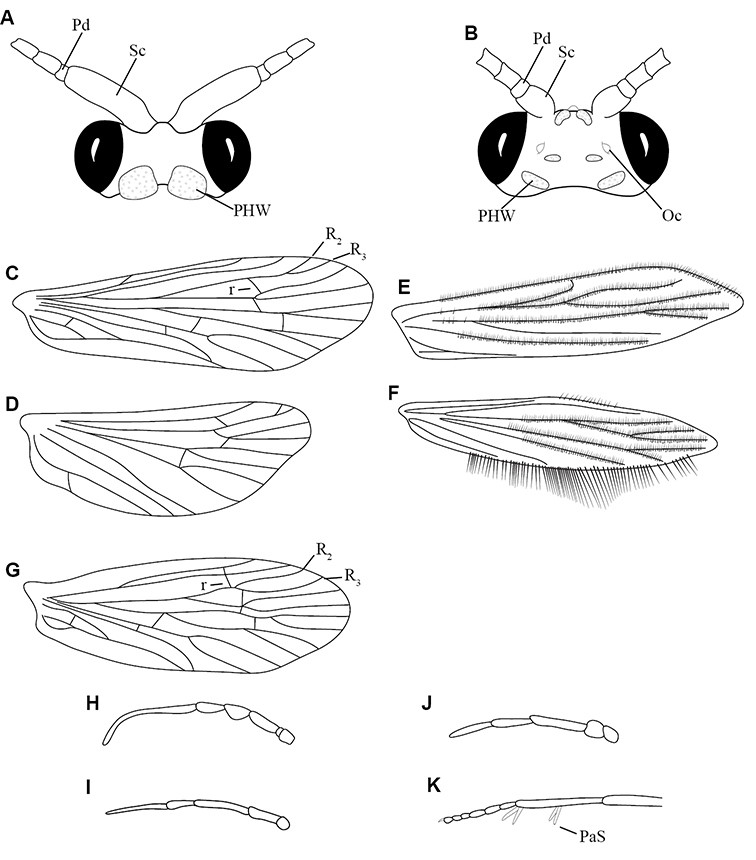
Adult caddisflies **A** head of *Helicopsyche borealis* **B** head of *Limnephilus canadensis* **C** forewing of *Polycentropus interruptus* **D** hindwing of *Polycentropus interruptus* **E** forewing of of *Hydroptila consimilis* **F** hindwing of *Hydroptila consimilis* **G** forewing of *Phylocentropus placidus* **H** maxillary palp of *Hydropsyche simulans* **I** maxillary palp of *Polycentropus interruptus* **J** maxillary palpi of *Rhyacophila fuscula* **K** foreleg of *Banksiola crotchi*. Abbreviations: PaS = preapical spur, PHW: posterior head warts, Pd = pedicel, Oc: ocellus, R = radial vein, r = radial crossvein, Sc = scape.

1. **Leptoceridae**. Forewing length 8–20 mm; usually light brown in color with darker blotches or reticulations; some species bright white or jet black. Antennae >2× length of body. Ocelli absent (Figure 13a). Common, widespread, and abundant throughout state; abundances can be extreme near any habitat type.

Similar families: two species of **Hydropsychidae** have antennae >2× body length, but have maxillary palpi 2× as long as preceding segment, flexible, and usually curved (Figure 13h). Antennal length is otherwise diagnostic, assuming they are intact.

2. **Hydropsychidae**. Forewing length 5–18 mm; usually light brown in color with darker reticulations; sometimes uniformly brown or straw-colored. Terminal segment of maxillary palpi 2× as long as preceding segment, flexible, and usually curved (Figure 13h). Ocelli absent (Figure 13a). Widespread, common, and abundant throughout state; abundances can be extreme near lotic habitats.

Similar families: **Arctopsychidae** is only found in small tributaries of Saint Croix River. **Polycentropodidae** has similar mouthparts, but with terminal segment of maxillary palpi not as long or curved (Figure 13i). **Philopotamidae** has similar mouthparts, but is darker in color and has ocelli (Figure 13b).

3. **Hydroptilidae**. Forewing length 2–5 mm; usually light brown in color; sometimes with darker reticulations. Forewings tapering to point and usually covered with dense setae (Figure 13e). Hindwings also tapering to point and with dense fringe of setae on posterior margin (Figure 13f). Ocelli present (Figure 13b). Widespread and abundant throughout state.

Similar families: some species of **Glossosomatidae**, **Psychomyiidae**, or **Polycentropodidae** may overlap in size. All of these families have forewings widening towards apex, not tapering (Figure 13c).

4. **Polycentropodidae**. Forewing length 5–12 mm; usually drab brown or grey in color; sometimes with darker reticulations. Ocelli absent (Figure 13a). Terminal segment of maxillary palpi 2× as long as preceding segment (Figure 13i). Widespread and abundant throughout state.

Similar families: **Hydropsychidae** has similar mouthparts, but with terminal segment of maxillary palpi longer and more flexible (Figure 13h). **Philopotamidae** has similar mouthparts, but is darker in color and has ocelli (Figure 13b). **Hydroptilidae** is almost always smaller and with forewings tapering to point (Figure 13e). **Dipseudopsidae** has R_2_ of wings branching from R_3_ near radial crossvein (Figure 13g), and is found only in northeastern MN. **Glossosomatidae** and **Psychomyiidae** may overlap in size, but don’t have long terminal segment of maxillary palpi.

5. **Philopotamidae**. Forewing length 8–10 mm; black or dark brown in color. Terminal maxillary segment of maxillary palpi 2× as long as preceding segment (Figure 13i). Ocelli present (Figure 13b). Widespread throughout state; can be extremely abundant near lotic habitats.

Similar families: **Hydropsychidae**, **Dipseudopsidae**, and **Polycentropodidae** all have similar mouthparts but are lighter in color and lack ocelli (Figure 13a).

6. **Psychomyiidae**. Forewing length 4–6 mm; black or dark brown in color. Ocelli absent (Figure 13b). Widespread and abundant throughout state; abundances can be extreme near lotic habitats. Females are far more abundant than males.

Similar families: **Glossosomatidae** and **Hydroptilidae** frequently overlap in size, but have ocelli (Figure 13a). **Polycentropodidae** occasionally overlaps in size, but has elongate terminal segment of maxillary palpi (Figure 13i).

7. **Helicopsychidae**. Forewing length 6–8 mm; light brown in color. Scape >2× as long as pedicel. Rear of head with large quadrate setal warts (Figure 13a). Ocelli absent. Widespread through state; can be abundant near lotic habitats.

Similar families: superficially resembles the **Lepidostomatidae** and **Sericostomatidae**, which lack the enlarged quadrate posterior head warts.

8. **Lepidostomatidae**. Forewing length 8–10 mm; light brown in color. Scape >2× as long as pedicel (Figure 13a). Ocelli absent. Widespread and abundant throughout Lake Superior, Northern, and Southeastern Regions.

Similar families: superficially resembles the **Brachycentridae**, but has scape >2× as long as pedicel. Similar to Helicopsychidae, but without quadrate posterior setal warts on head.

9. **Limnephilidae**. Forewing length 12–40 mm; usually light brown with darker reticulations, but can be nearly any color. Ocelli present (Figure 13b). Each middle tibia with 0 or 1 pre-apical spurs. Widespread throughout state but usually not abundant.

Similar families; frequently confused with the **Phryganeidae** due to large size; can be differentiated by the number of preapical spurs on middle tibiae.

10. **Phryganeidae**. Forewing length 10–35 mm; usually brown, grey or dark orange with dark reticulations. Ocelli present (Figure 13a). Each middle tibia with 2 pre-apical spurs (Figure 13k). Found throughout state, but abundant only in northern MN.

Similar families; frequently confused with the **Limnephilidae** due to large size; can be differentiated by the number of preapical spurs on middle tibiae.

11. **Molannidae**. Forewing length 8–12 mm; black or dark grey in color. Elongate body. Widespread throughout state. Scape >2× as long as pedicel (Figure 13a). Ocelli absent. Widespread throughout state but usually not abundant.

Similar families: Color and body shape are distinctive. Superficially resemble the **Leptoceridae**, but have antennae <2× length of body.

12. **Glossosomatidae**. Forewing length 4–8 mm; black or dark brown in color. Second segment of maxillary palpi globose in shape (Figure 13j). Ocelli present (Figure 13b). Widespread throughout state; abundant only in Southeastern Region.

Similar families: **Rhyacophilidae** has similar mouthparts, but is larger and confined to the Lake Superior Region. Hydroptilidae may overlap in size, but has forewings tapering to a point (Figure 13e)

13. **Brachycentridae**. Forewing length 5–12 mm; dark grey to black in color; often with light spots near apical margins. Ocelli absent (Figure 13b). Widespread throughout state and locally abundant.

Similar families: superficially resemble **Lepidostomatidae** and **Sericostomatidae**, but have scape <3× length of pedicel.

14. **Uenoidae**. Forewing length 8–10 mm; dark brown in color; sometimes with bright orange patches and reticulations. Ocelli present (Figure 13a). Widespread throughout Lake Superior, Northern, and Southeastern Regions. Only collected from mid-August to mid-October.

Similar families: superficially resembles **Limnephilidae** and **Phryganeidae**, but is smaller, usually with bright orange patches on forewings, and only emerges in the fall. Also superficially resembles **Apataniidae**, which is uniformly light brown and found only along the North Shore of Lake Superior.

15. **Rhyacophilidae**. Forewing length 14–18 mm; black or dark brown in color without notable patterning. Second segment of maxillary palpi globose in shape (Figure 13j). Ocelli present (Figure 13a). Found only throughout Lake Superior Region.

Similar families: **Glossosomatidae** has similar mouthparts, but is smaller.

16. **Dipseudopsidae**. Forewing length 10–12 mm; drab brown in color with faint darker reticulations. Ocelli absent (Figure 13a). Found only in northeastern MN. Rarely abundant.

Similar families: **Polycentropodidae** is much more common and has R_2_ and R_3_ of wings either unbranched or else branching near wing margin (Figure 13c).

The following families are unlikely to be collected by a general collector.

17. **Sericostomatidae**. Forewing length 8–12 mm; grey in color. Scape >2× as long as pedicel (Figure 13a). Ocelli absent. Found only in the northeastern portion of the state and rarely abundant.

Similar families: superficially resembles **Lepidostomatidae**.

18. **Apataniidae**. Forewing length 6–8 mm; light brown in color. Ocelli present (Figure 13b). Found only along north shore of Lake Superior.

Similar families: superficially resembles **Uenoidae** and **Limnephilidae**.

19. **Arctopsychidae**. Forewing length 10–12 mm; grey in color with darker reticulations. Terminal segment of maxillary palpi long and flexible, usually curved (Figure 13h). Ocelli absent (Figure 13a). Found only in small tributaries of the Saint Croix River.

Similar families: very similar to **Hydropsychidae**.

20. **Goeridae**. Forewing length 6–8 mm; light brown in color. Found only near Lake Itasca State Park. Ocelli absent (Figure 13a).

Similar families: superficially resembles the **Lepidostomatidae**.

21. **Odontoceridae**. Forewing length 6–8 mm; dark brown in color. Elongate body. Ocelli absent (Figure 13a). Found only in extreme northeastern MN.

Similar families: may superficially resemble the **Molannidae**.

### Family Apataniidae

This family contains a single genus in Minnesota, *Apatania*, and a single species. For additional species, see Schmid (1953) or Nimmo (1971). Larvae of *Apatania* typically consume algae and diatoms from the surfaces of medium and large rocks, and may inhabit either lakes or streams. Larval cases are constructed of small mineral fragments (Wiggins 1996). Adults are light brown in color, and 6–8 mm in length (Figure 290).

### Genus *Apatania*

**Figure 14.**
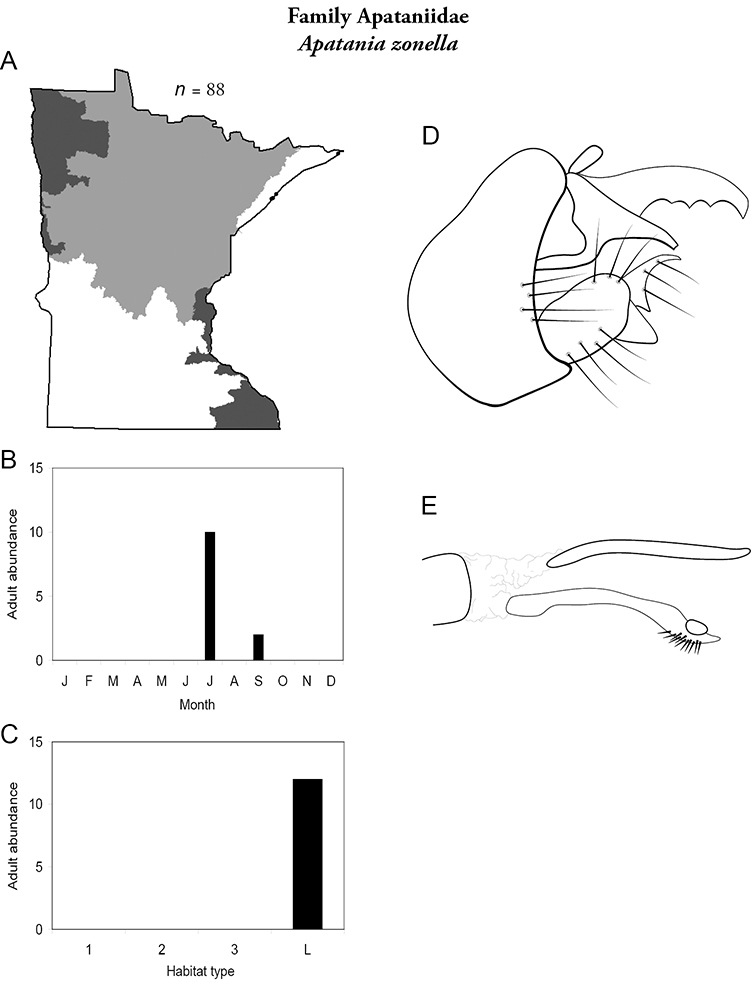
*Apatainia zonella* **A** total specimens collected and all known collecting localities (Figure 4) **B** monthly adult abundance (1980s to present) **C** habitat preference (1980s to present) (Table 1) **D** male genital capsule **E** phallus.

***Apatania zonella*** (Figure 14) has been found only along the north shore of Lake Superior during July and September. Adults were abundant along riparian rocks and vegetation during the day; they were not attracted to lights at night. The apparent bivoltine adult periodicity is probably spurious, and instead likely reflects a lack of collecting during August.

Another *Apatania* species, *Apatania incerta* was reported from northeastern Minnesota based on a female specimen (Etnier 1965). The whereabouts of this specimen is unknown, but it is likely *Apatania zonella*, which is known from this area of Minnesota. Since the presence of *Apatania incerta* cannot of verified, it is not included in this manual.

### Family Arctopsychidae

This family, considered a subfamily of the Hydropsychidae in one recent classification (Holzenthal et al. 2011), contains a single genus in Minnesota, *Parapsyche*, and a single species. For additional species, see Nimmo (1987) or Schmid (1968). Larvae construct web-like nets of silk to capture large suspended organic particles (Wiggins 1996). The mesh size of these nets is larger than nearly any other species of net-spinning caddisfly (Wallace and Malas 1976). Thus, larvae require fast-moving current to suspend their food source. They are also cold water stenotherms usually restricted to streams with rocky substrates. Adults have grey wings with darker reticulations, and are 10–12 mm in length.

### Genus *Parapsyche*

**Figure 15.**
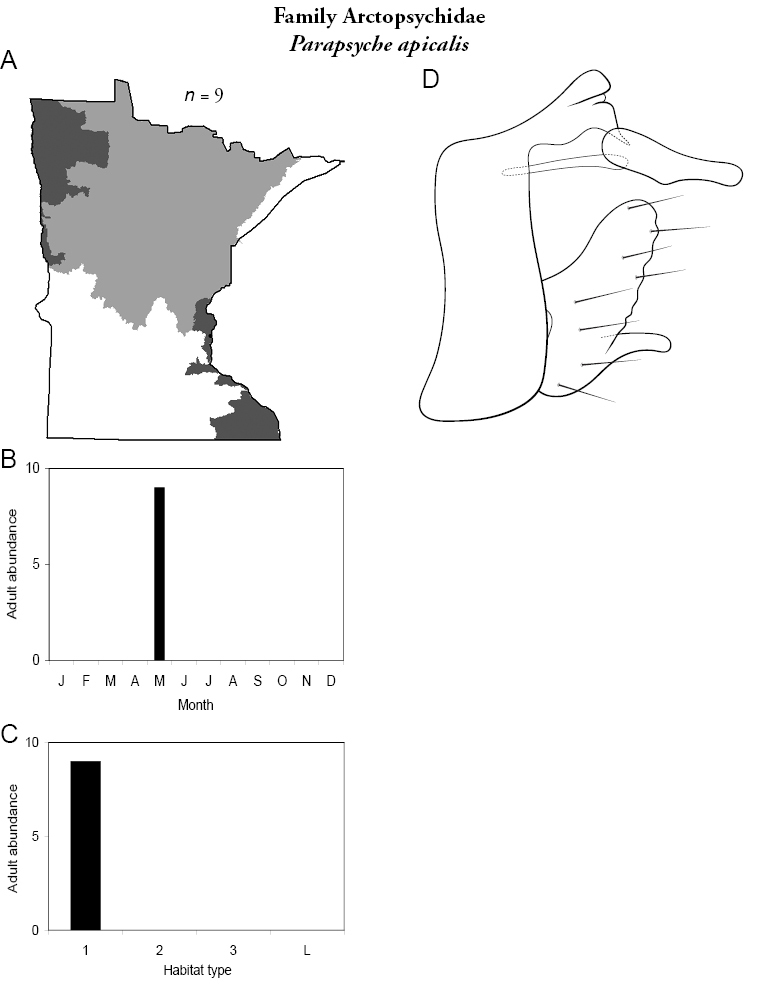
*Parapsyche apicalis* **A** total specimens collected and all known collecting localities (Figure 4) **B** monthly adult abundance (1980s to present) **C** habitat preference (1980s to present) (Table 1) **D** male genital capsule **E** phallus.

***Parapsyche apicalis*** (Figure 15) is known only from adults collected in May 2001 from Mill Creek, William O’Brien State Park, in the Southeastern Region. The species appears to be at the western edge of its range in Minnesota. Furthermore, *Parapsyche apicalis* is almost exclusively found in very small (<1 m wide) streams with dense canopy cover (Wiggins 1996, Houghton et al. 2011b). Small cold water streams appropriate for *Parapsyche apicalis* are very rare in eastcentral Minnesota, and those that remain are under imminent threat of urban development. Thus, the Minnesota Department of Natural Resources has proposed that *Parapsyche apicalis* be listed as “Threatened” (MNDNR 2012).

### Family Brachycentridae

This family contains two genera in Minnesota: *Brachycentrus* and *Micrasema*, and a total of 6 species. Both genera are characteristic of running waters; *Brachycentrus* species tend to inhabit small and medium streams, whereas species of *Micrasema* prefer medium to large rivers. Both genera contain species that tend to have specific habitat requirements; thus collections can be both sporadic and sometimes containing >1,000 specimens.

### Genus *Brachycentrus*

The genus*Brachycentrus* contains 3 species in Minnesota. Two of them are rarely encountered. Larvae typically consume relatively large suspended particulate organic matter that they grasp with their legs (Wiggins 1996). Thus, they typically inhabit only fast-moving woodland streams with a riparian canopy. Cases are constructed of narrow pieces of plant material arranged transversely (Wiggins 1996). Adults are 10–12 mm in length with dark grey wings, often with light spots near the apical margins. For additional species, see Flint (1984).

**Figure 16.**
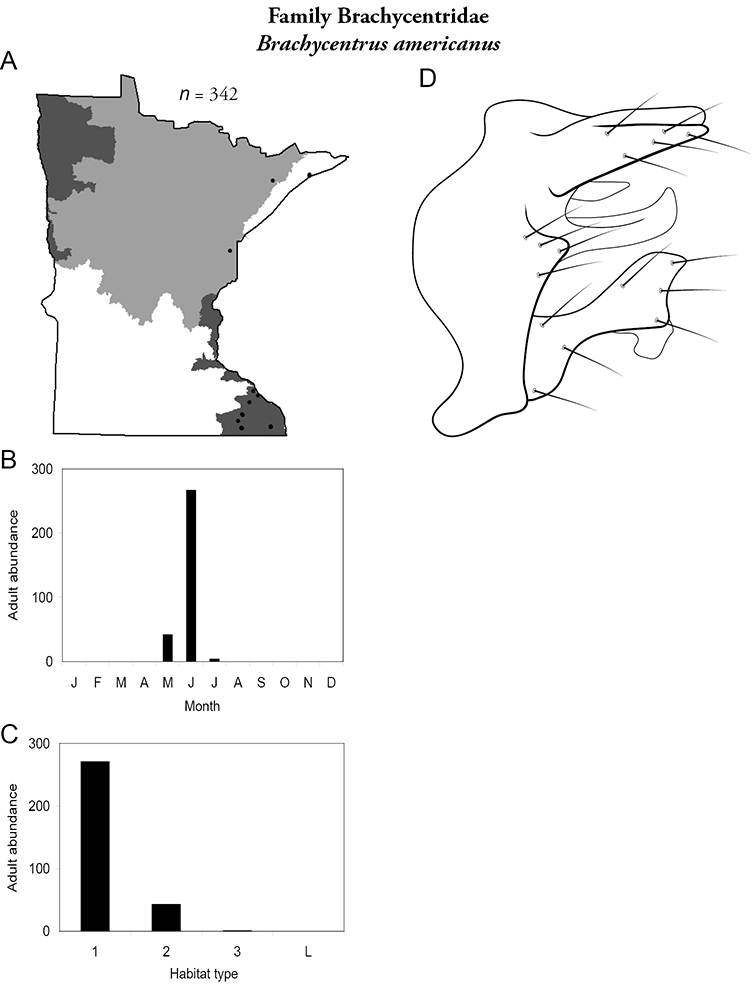
*Brachycentrus americanus* **A** total specimens collected and all known collecting localities (Figure 4) **B** monthly adult abundance (1980s to present) **C** habitat preference (1980s to present) (Table 1) **D** male genital capsule.

***Brachycentrus americanus*** (Figure 16) was the most abundant species in small streams of the Southeastern Region, representing 38% of all collected specimens (Table 6). It was also the second most abundant species in medium streams of the Southeastern Region. It was also found sporadically in northeastern portion of the state. Adults were present from May to early July. Unlike other reports of *Brachycentrus* (Flint 1984, Moulton and Stewart 1996), adults of *Brachycentrus americanus* were easily caught in light traps.

**Figure 17.**
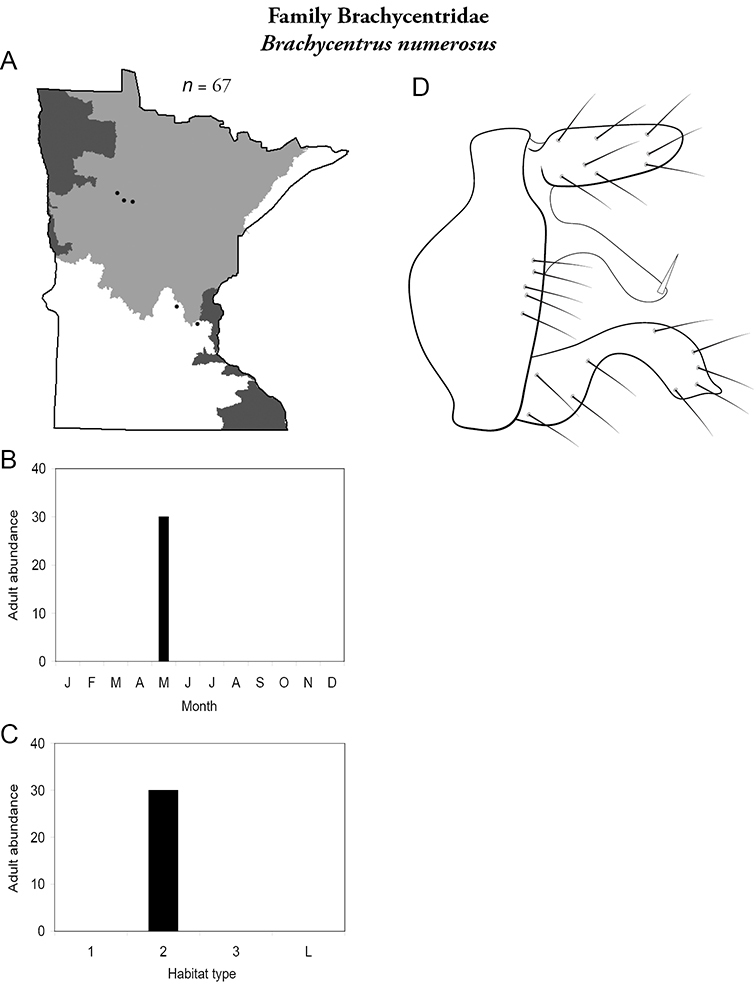
*Brachycentrus numerosus* **A** total specimens collected and all known collecting localities (Figure 4) **B** monthly adult abundance (1980s to present) **C** habitat preference (1980s to present) (Table 1) **D** male genital capsule.

***Brachycentrus numerosus*** (Figure 17) is known mostly from sporadic larval rearing from the Northern and Southern Regions. The only series of adults was collected in May from the Kabekona River, Hubbard County, in the Northern Region using ultraviolet lights.

**Figure 18.**
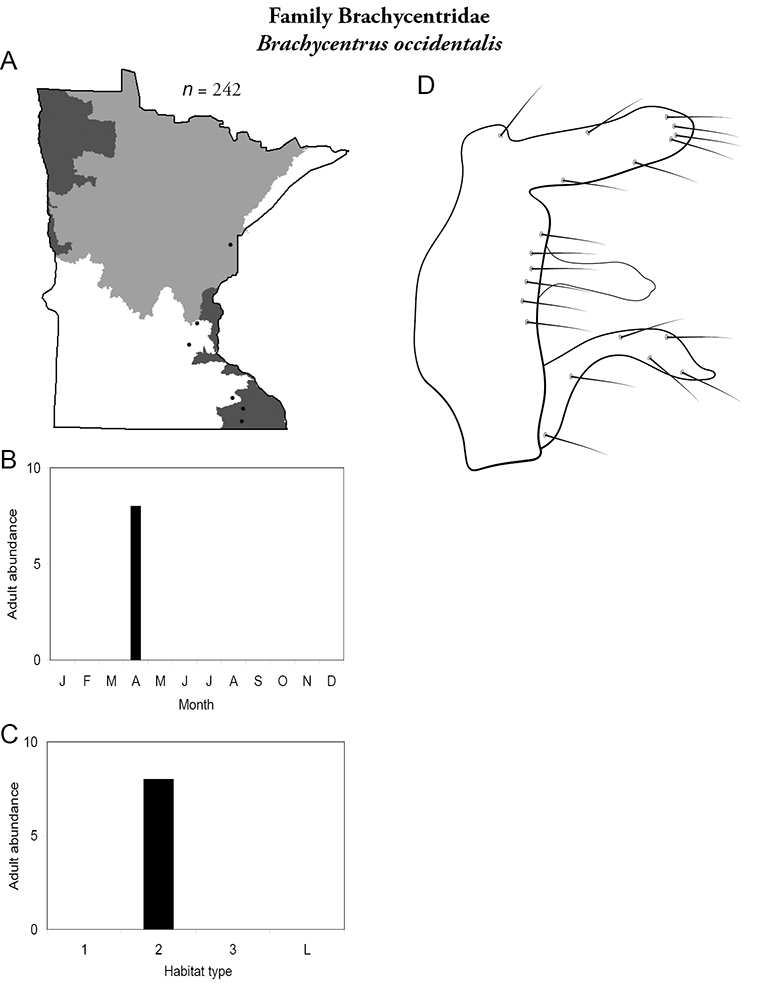
*Brachycentrus occidentalis* **A** total specimens collected and all known collecting localities (Figure 4) **B** monthly adult abundance (1980s to present) **C** habitat preference (1980s to present) (Table 1) **D** male genital capsule.

***Brachycentrus occidentalis*** (Figure 18) is known in Minnesota primarily from the Southeastern Region. Nearly all collections have come from reared larvae that emerged in April or May. The only series of adults came from diurnal sweep-netting in April from Forestville State Park in the Southeastern Region.

Another *Brachycentrus* species, *Brachycentrus fuliginusos* has been reported from Minnesota based on an adult specimen of unknown gender (Morse 1993). The whereabouts of this specimen is unknown. In the absence of specimens, *Brachycentrus fuliginusos* is not included in this manual.

### Genus *Micrasema*

The genus*Micrasema* contains 3 species in Minnesota. For additional species, see Chapin (1978). Larvae feed on periphyton or decaying organic material and are typically found in medium and large rivers, often associated with mats of algae or moss (Chapin 1978). Larval cases are constructed predominantly of very small sand grains or tightly woven silk alone (Wiggins 1996). Adults are black or dark brown and 5–7 mm in length. Some smaller specimens may be confused with the Hydroptilidae.

**Figure 19.**
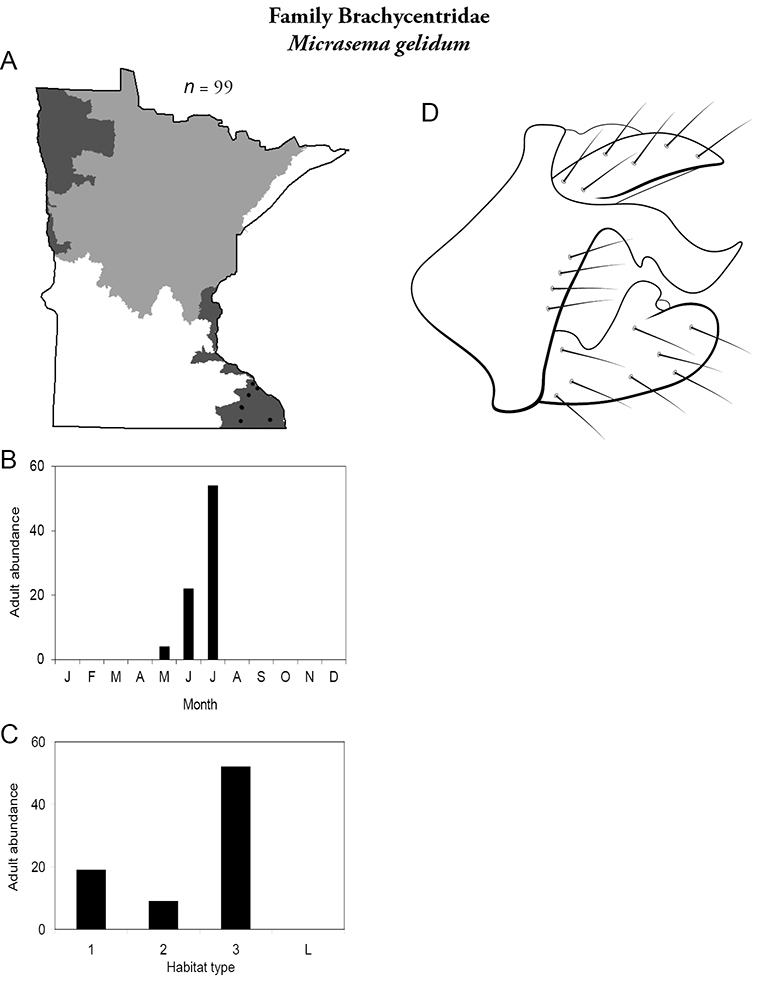
*Micrasema gelidum* **A** total specimens collected and all known collecting localities (Figure 4) **B** monthly adult abundance (1980s to present) **C** habitat preference (1980s to present) (Table 1) **D** male genital capsule.

***Micrasema gelidum*** (Figure 19) is known only from the Southeastern Region, where it was most common in all sizes of streams, especially large rivers. Adults were mainly present in June and July.

**Figure 20.**
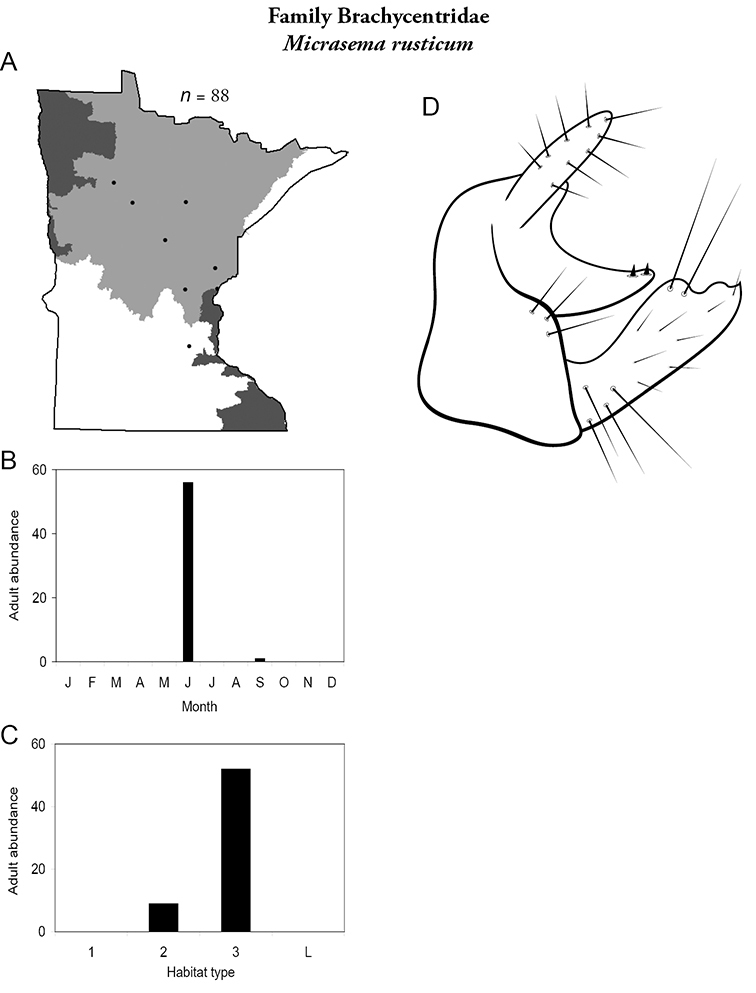
*Micrasema rusticum* **A** total specimens collected and all known collecting localities (Figure 4) **B** monthly adult abundance (1980s to present) **C** habitat preference (1980s to present) (Table 1) **D** male genital capsule.

***Micrasema rusticum*** (Figure 20) has been found predominantly in medium and, especially, large rivers of the Northern Region, mostly during June.

**Figure 21.**
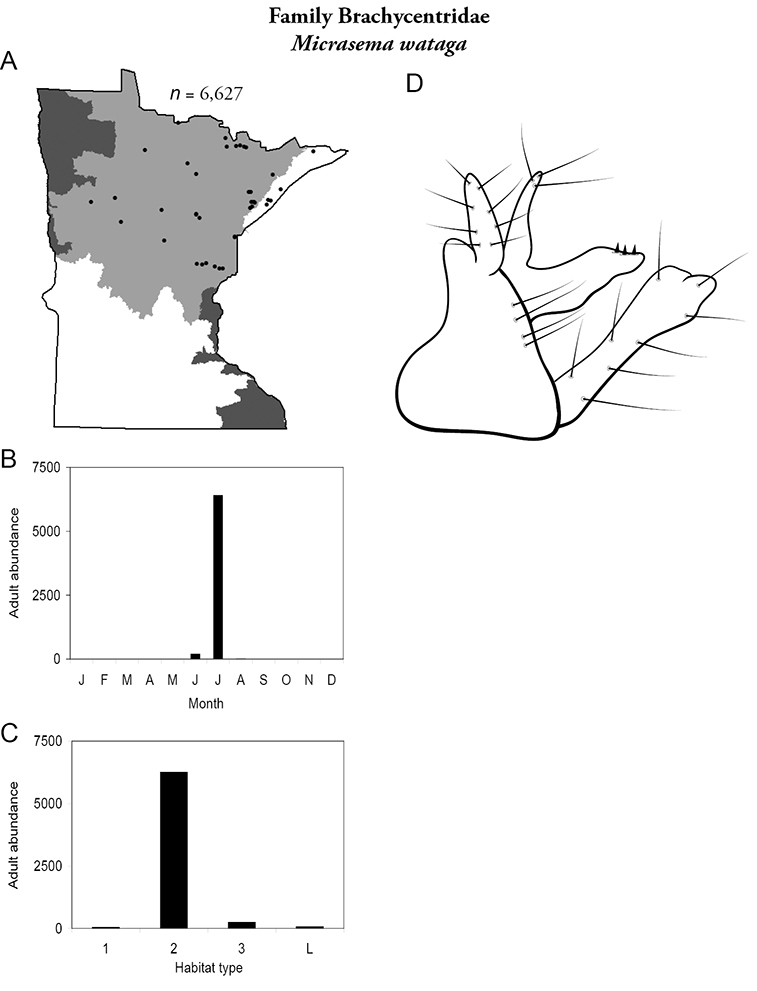
*Micrasema wataga* **A** total specimens collected and all known collecting localities (Figure 4) **B** monthly adult abundance (1980s to present) **C** habitat preference (1980s to present) (Table 1) **D** male genital capsule.

***Micrasema wataga*** (Figure 21) was found throughout the Lake Superior and Northern Regions during June and, especially, July. It was the 10th most abundant species overall in Minnesota (Figure 9); ~90% of this abundance was due to a single collection from the Straight River, Hubbard County, in the Northern Region which yielded >6,000 specimens.

### Family Dipseudopsidae

This family contains a single genus in Minnesota, *Phylocentropus*, and a single species. For additional species, see Armitage and Hamilton (1990) or Schuster and Hamilton (1984). Larvae construct a branching silken retreat which is buried in the sandy substrates of lakes and streams (Wiggins 1996). Larvae are filtering collectors, gathering fine particulate organic matter that circulates through the retreat system (Wallace et al. 1976). Adults are drab brown in color and 10–12 mm in length. The Dipseudopsidae used to be considered a subfamily of the Polycentropodidae, and dipseudopsid species do superficially resemble those of the latter family.

### Genus *Phylocentropus*

**Figure 22.**
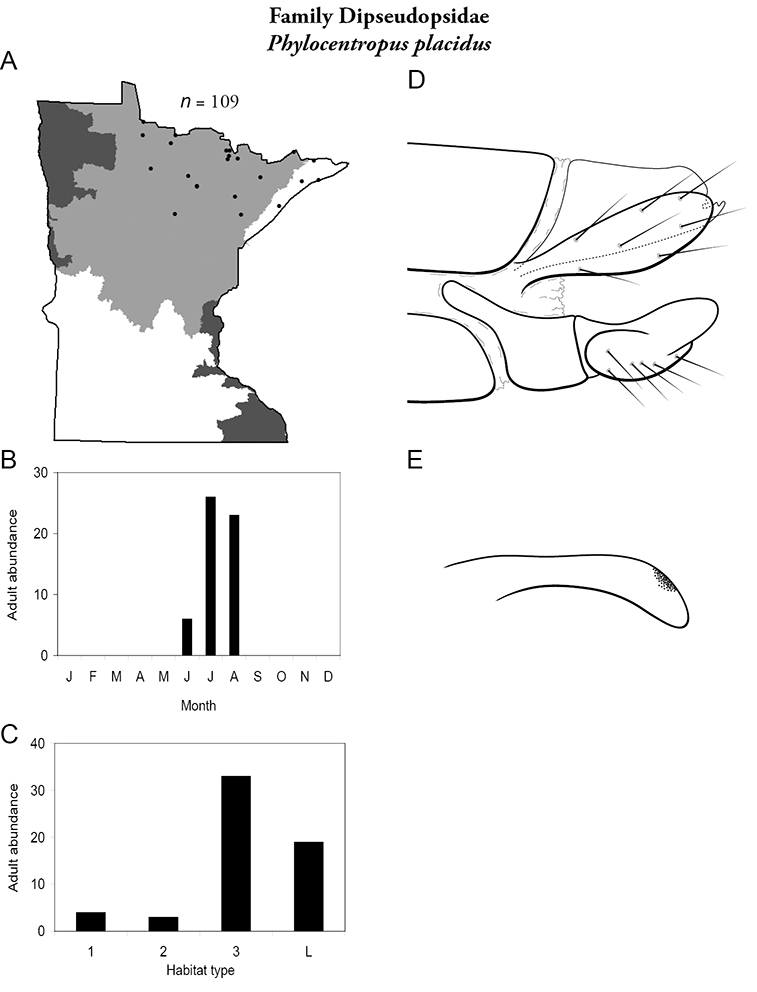
*Phylocentropus placidus* **A** total specimens collected and all known collecting localities (Figure 4) **B** monthly adult abundance (1980s to present) **C** habitat preference (1980s to present) (Table 1) **D** male genital capsule **E** phallus.

***Phylocentropus placidus*** (Figure 22) was found in the Lake Superior and Northern Regions. It was collected most frequently from large, sandy-bottomed rivers, but was also common in lakes. Adults were most abundant in July and August, with some present in June.

### Family Glossosomatidae

This family contains 3 genera in Minnesota: *Agapetus*, *Glossosoma*, and *Protoptila*, and a total of 7 species. Larvae are found on the surfaces of medium to large rocks in fast-moving current where they graze on algae and diatoms. They construct “saddle” cases that superficially resemble turtle shells (Wiggins 1996). Species of *Protoptila* are usually <5 mm in length and may be confused with the Hydroptilidae. Adults of the other genera are between 5–8 mm and black or dark brown in color. Females of *Glossosoma* and *Protoptila* tend to be far more abundant than males; unfortunately, females are difficult to identify. Thus, it is possible that all species are more widespread than they appear.

### Genus *Agapetus*

The genus *Agapetus* contains 2 species in Minnesota. For additional species see Etnier et al. (2010). Larvae are cold-water stenotherms and typically found in high gradient streams. They tend to be highly intolerant of organic pollution. Thus, they often exhibit very specific habitat requirements.

**Figure 23.**
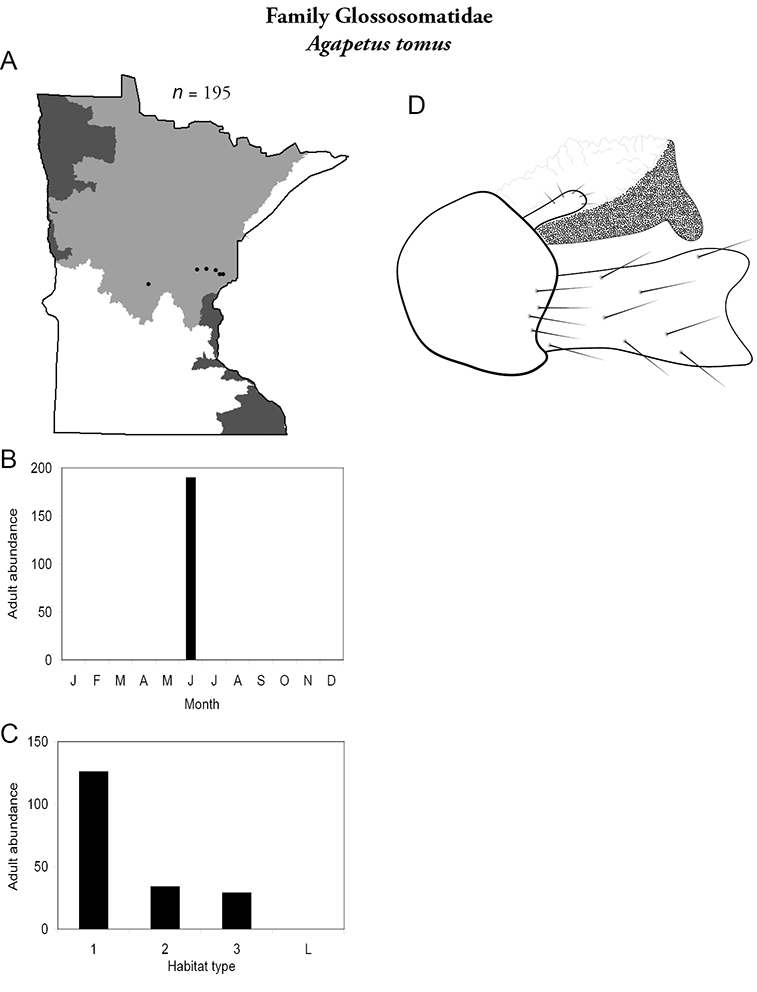
*Agapetus tomus* **A** total specimens collected and all known collecting localities (Figure 4) **B** monthly adult abundance (1980s to present) **C** habitat preference (1980s to present) (Table 1) **D** male genital capsule.

***Agapetus tomus*** (Figure 23) was collected only in June and only from the Northern Region. It was locally abundant in a variety of stream types, especially small streams. Interestingly, all of these streams were in a line approximating the 46° parallel. It is difficult to determine the specific habitat requirements of this species or why it is only found in these specific streams (Houghton and Holzenthal 2003). Due to its rarity and enigmatic distribution in Minnesota, *Agapetus tomus* is listed as “Special Concern” by the Minnesota Department of Natural Resources (MNDNR 2012).

**Figure 24.**
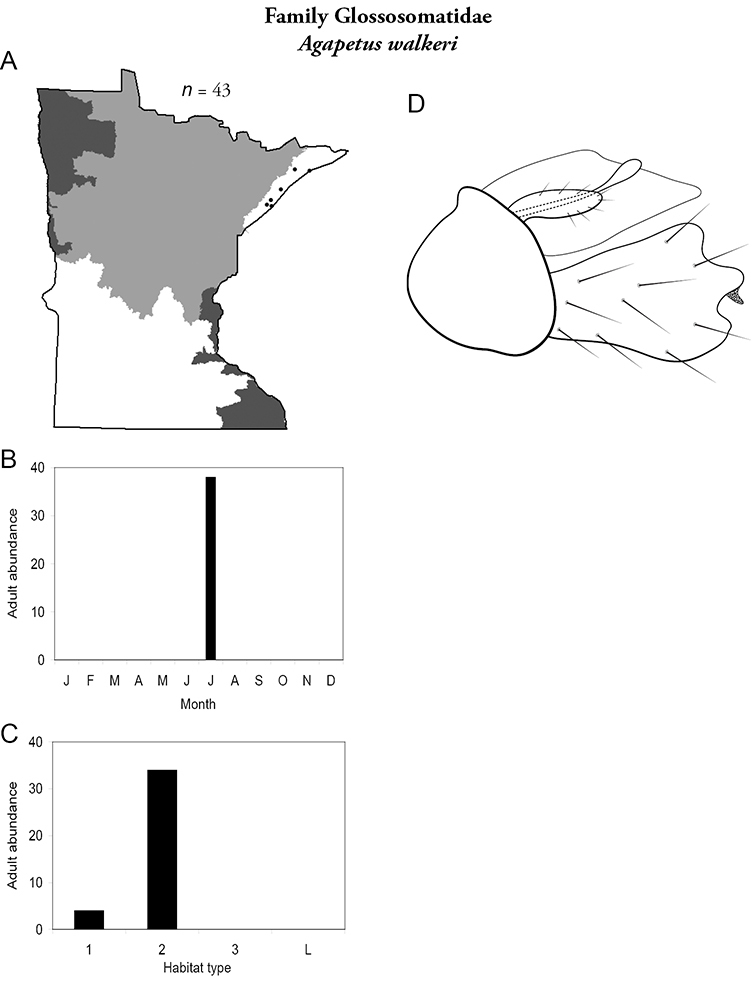
*Agapetuc walkeri* **A** total specimens collected and all known collecting localities (Figure 4) **B** monthly adult abundance (1980s to present) **C** habitat preference (1980s to present) (Table 1) **D** male genital capsule.

***Agapetus walkeri*** (Figure 24) was only found in small and, especially, medium streams of the Lake Superior Region. Adults were collected in July.

Another *Agapetus* species, *A illini* was reported from northeastern Minnesota based on a series of specimens (MacLean 1995). All of these specimens were re-identified as *Agapetus rossi* (Houghton et al. 2001). Thus, *A illini* is not included in this manual. *Agapetus rossi*, reported from Minnesota in Houghton et al. (2001) has been recently designated a junior subjective synonym of *Agapetus walkeri* (Etnier et al. 2010). Thus, it is also not included in this manual.

### Genus *Glossosoma*

The genus *Glossosoma* contains 2 species in Minnesota. For additional species, see Schmid (1982). Like those of *Agapetus*, larvae are typically found in fast-moving cold streams, although they are less stenothermic and are more widely distributed.

**Figure 25.**
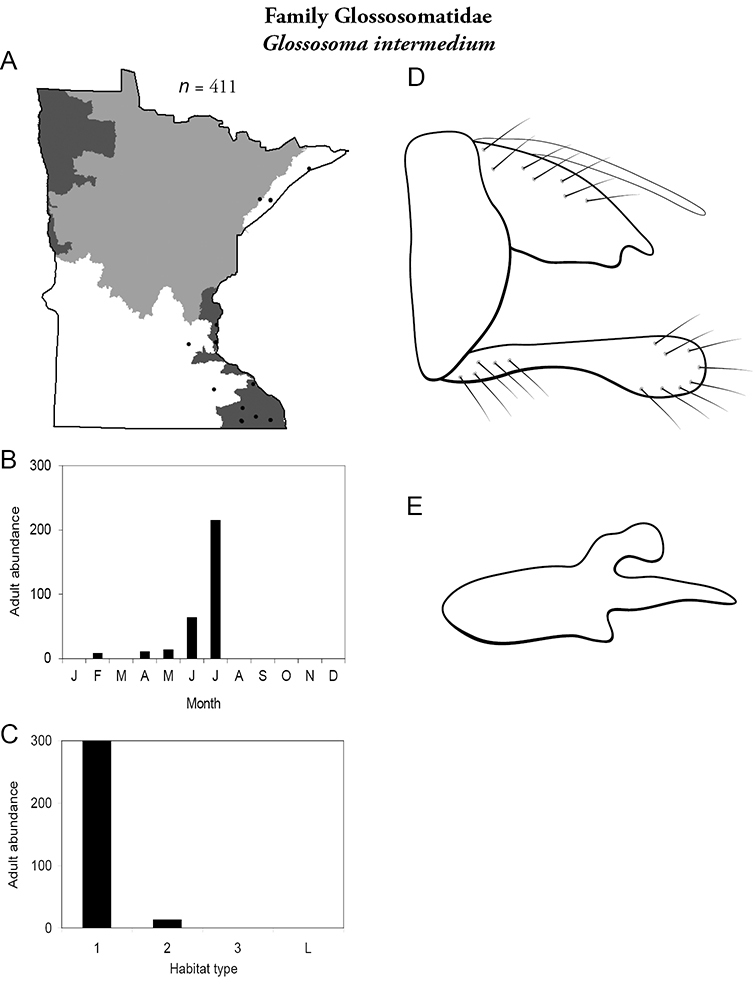
*Glossosoma intermedium* **A** total specimens collected and all known collecting localities (Figure 4) **B** monthly adult abundance (1980s to present) **C** habitat preference (1980s to present) (Table 1) **D** male genital capsule **E** phallus.

***Glossosoma intermedium*** (Figure 25) was found in small and medium streams, predominately in the Lake Superior and, especially, the Southeastern Regions. The majority of adults were collected during July; however, some were found as early as February. Many others have been reared in the lab from larvae and have emerged in March through May.

**Figure 26.**
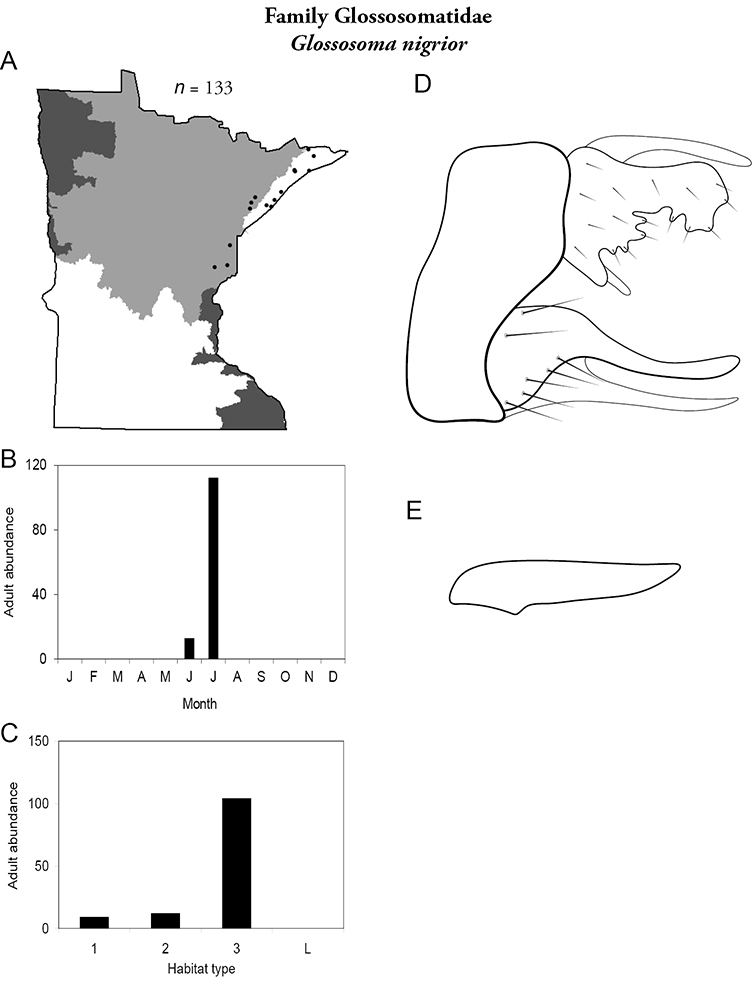
*Glossosoma nigrior* **A** total specimens collected and all known collecting localities (Figure 4) **B** monthly adult abundance (1980s to present) **C** habitat preference (1980s to present) (Table 1) **D** male genital capsule **E** phallus.

***Glossosoma nigrior*** (Figure 26) is known from the Lake Superior and Northern Regions, predominately from large rivers. It was collected during June and, especially, July.

### Genus *Protoptila*

The genus*Protoptila* contains 3 species in Minnesota. For additional species, see Schmid (1982). Larvae typically inhabit larger and warmer rivers than other genera in the family, although they still prefer relatively fast-moving current. Adults are <5mm and brown in color, with a transverse lighter stripe on the forewing (Figure 290).

**Figure 27.**
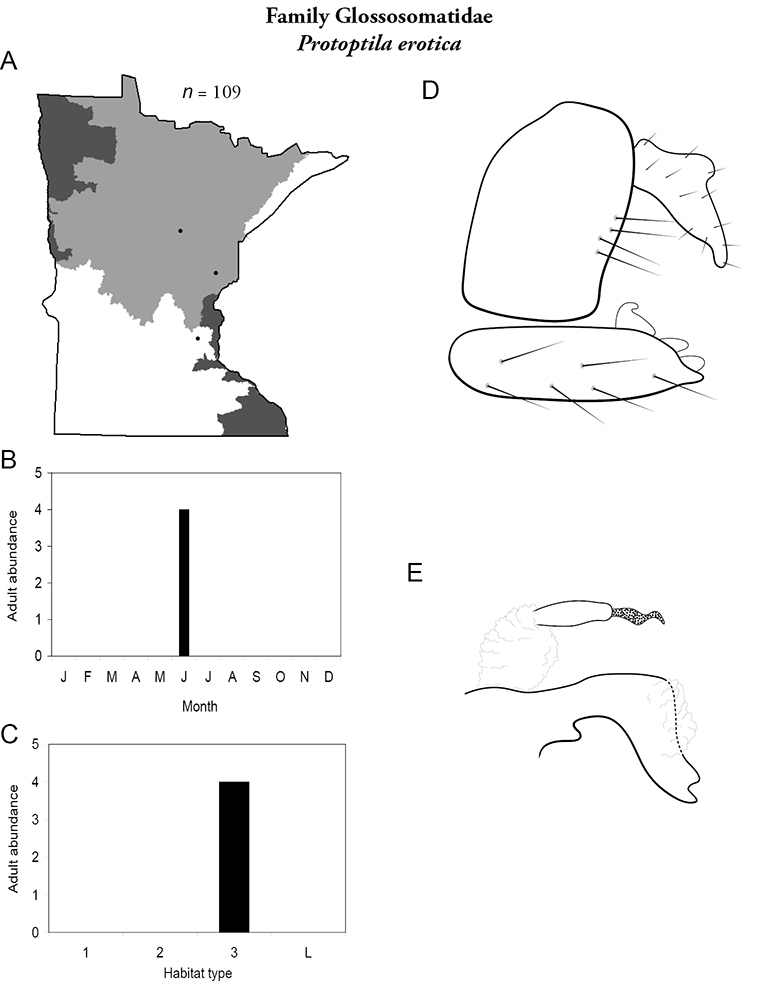
*Protoptila erotica* **A** total specimens collected and all known collecting localities (Figure 4) **B** monthly adult abundance (1980s to present) **C** habitat preference (1980s to present) (Table 1) **D** male genital capsule **E** phallus.

***Protoptila erotica*** (Figure 27) is known from a total of 3 localities in the Northern Region and only 1, the Kettle River in Pine County, since the 1930s. The historical collections yielded >100 specimens. The recent collection yielded only 4. All collections occurred in June. Due to its limited distribution and apparent decrease in abundance since the 1930s, the Minnesota Department of Natural Resources has proposed that *Protoptila erotica* be listed as “Threatened” (MNDNR 2012).

**Figure 28.**
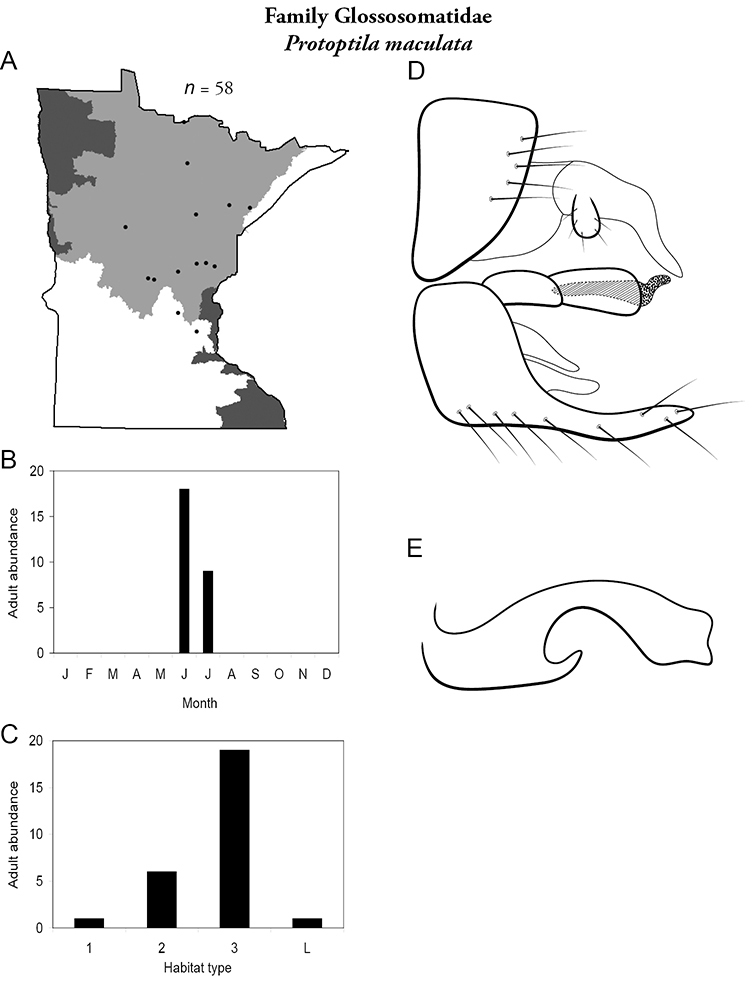
*Protopila maculata* **A** total specimens collected and all known collecting localities (Figure 4) **B** monthly adult abundance (1980s to present) **C** habitat preference (1980s to present) (Table 1) **D** male genital capsule **E** phallus.

***Protoptila maculata*** (Figure 28) has been found mostly in medium and, especially, large rivers during June and July. It is known predominately from the Northern Region.

**Figure 29.**
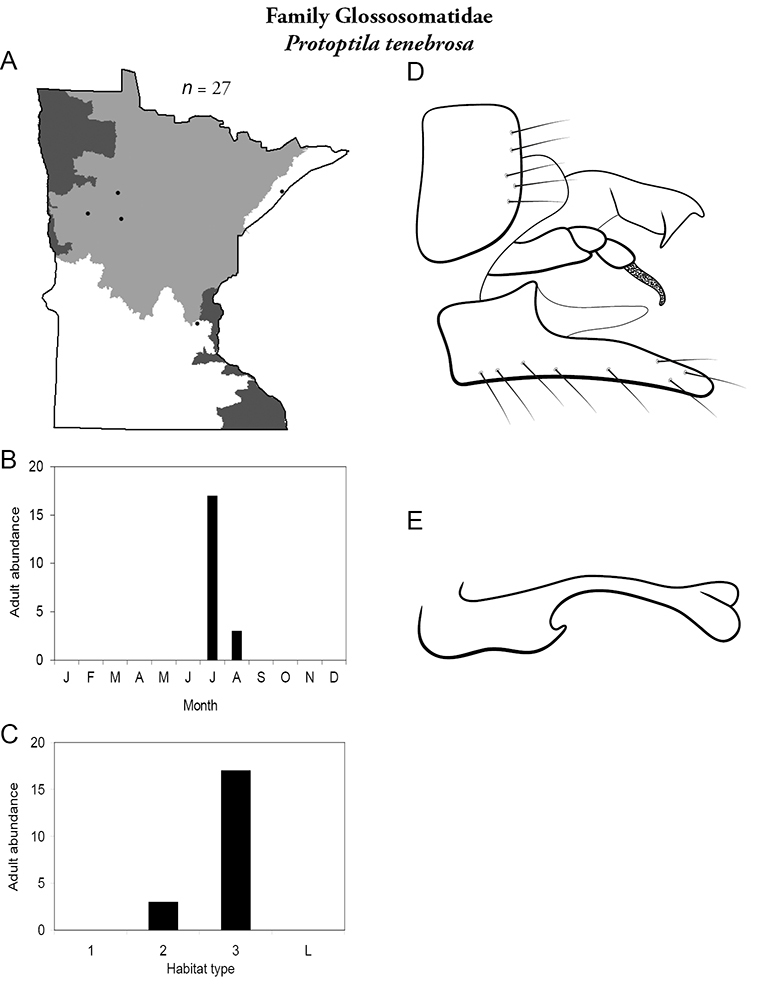
*Protoptila tenebrosa* **A** total specimens collected and all known collecting localities (Figure 4) **B** monthly adult abundance (1980s to present) **C** habitat preference (1980s to present) (Table 1) **D** male genital capsule **E** phallus.

***Protoptila tenebrosa*** (Figure 29) has been collected mostly from large rivers in July. Collecting localities are widely separated from each other.

A fourth *Protoptila* species, *Protoptila talola*, is known worldwide from a single Minnesota specimen collected in 1941 from an unknown habitat in Pine County. Due to its rarity and Minnesota endemism, the species is listed as “Special Concern” by the Minnesota Department of Natural Resources (MNDNR 2012). Despite a concerted collecting effort throughout this area, the species has never been recaptured. Moreover, *Protoptila talola* is very similar in appearance to *Protoptila maculata*. Thorough examination of the only known “*Protoptila talola*” specimen, deposited in the UMSP, suggests that it may, in fact, be an aberrant specimen of *Protoptila maculata*. This taxonomic confusion has caused the Minnesota Department of Natural Resources to propose removing *Protoptila talola* from its list of protected species (MNDNR 2012). Thus, it is not included in this manual.

### Family Goeridae

This family contains a single species in Minnesota, *Goera*, and a single species. Larvae are characteristic of running water where they consume algae and small organic particles from the surfaces of medium and large rocks. Larval cases are constructed of small mineral particles, with larger pebbles used as ballast stones (Wiggins 1996). Adults are light brown in color and 6–8 mm in length. Their general form is similar to species in the Lepidostomatidae.

### Genus *Goera*

**Figure 30.**
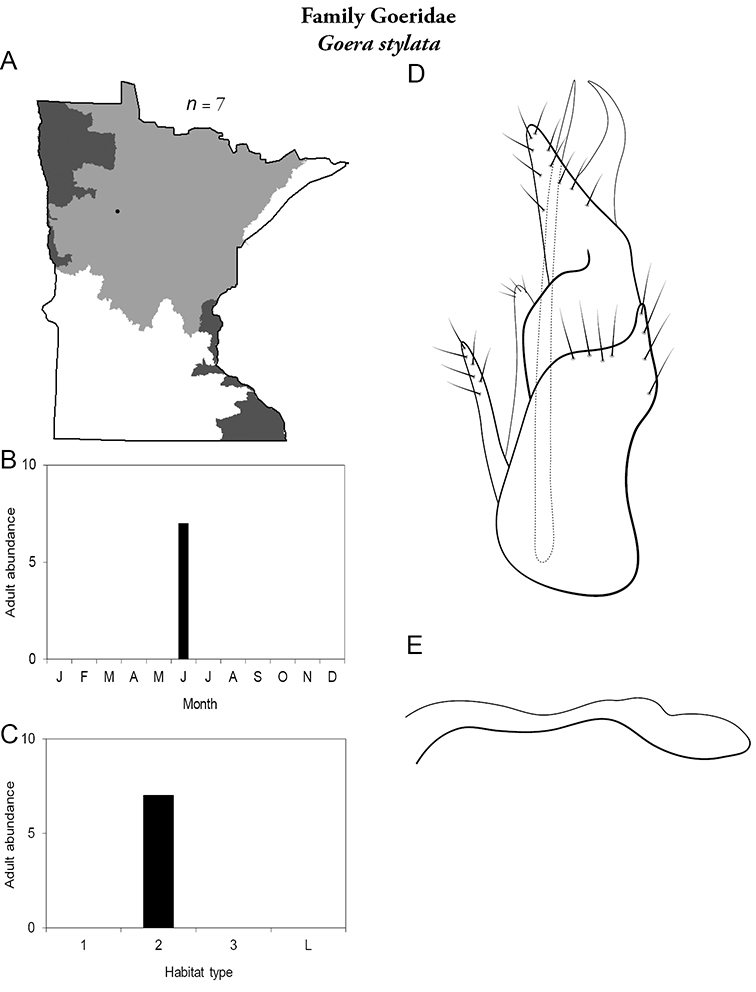
*Goera stylata* **A** total specimens collected and all known collecting localities (Figure 4) **B** monthly adult abundance (1980s to present) **C** habitat preference (1980s to present) (Table 1) **D** male genital capsule (rotated 90 degrees counter-clockwise) **E** phallus.

***Goera stylata*** (Figure 30) is known only from LaSalle Creek, Clearwater County, in the Northern Region. Adults were collected during June. The species tends to have specific habitat requirements, and is typically present as an adult only for a brief period of time (Houghton et al. 2011b). Thus, it may occur in other places in the state and is difficult to collect. Due to its extremely limited known distribution, the Minnesota Department of Natural Resources has proposed “Threatened” status for the species (MNDNR 2012).

Another *Goera* species, *Goera calcarata*, was reported from Minnesota based on a series of larvae (Etnier 1965). No adults of the species have been collected from the state. The specimen whereabouts are unknown, but they are likely *Goera stylata*. Thus, *Goera calcarata* is not included in this manual.

### Family Helicopsychidae

This family contains 1 genus in Minnesota, *Helicopsyche*, and a single species. For additional species, see Johanson (2002). Larval cases are composed of small minerals and are coiled in shape, resembling a snail shell. Larvae are commonly found on medium to large rocks in fast-moving current where they graze on periphyton and diatoms (Wiggins 1996). In fact, their coiled snail-case presents a convex face into current in any direction, thus allowing for larval movement in high current environments (Vaughn 1987). Adults are light brown in color and are typically 6–8 mm long. Some specimens, however, may be <5 mm.

### Genus *Helicopsyche*

**Figure 31.**
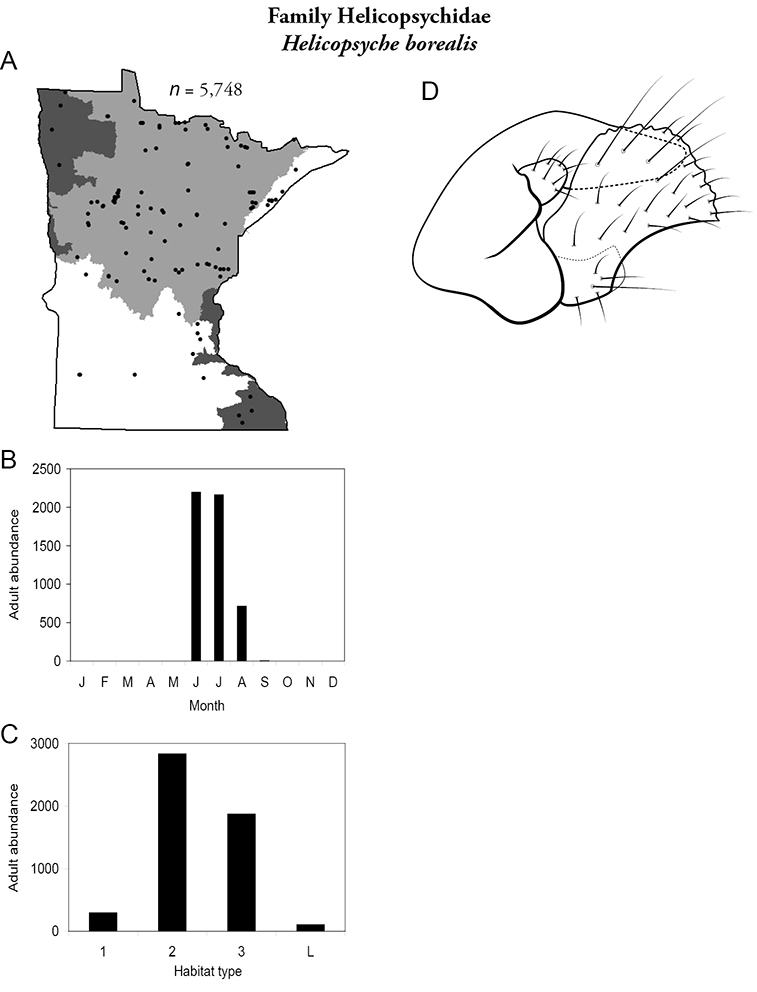
*Helicopsyche borealis* **A** total specimens collected and all known collecting localities (Figure 4) **B** monthly adult abundance (1980s to present) **C** habitat preference (1980s to present) (Table 1) **D** male genital capsule.

***Helicopsyche borealis*** (Figure 31) was common and abundant throughout Minnesota, especially in medium and large rivers of the Lake Superior and Northern Regions. Adults were present June through August.

### Family Hydropsychidae

This family contains 5 genera in Minnesota: *Cheumatopsyche*, *Diplectrona*, *Hydropsyche*, *Macrostemum*, and *Potamyia*, and a total of 29 species. It is the 4th most species-rich family (Figure 6). Larvae are very common and conspicuous members of all types of streams. Indeed, picking up nearly any medium or large rock in the flowing water of virtually any stream is likely to yield larval specimens.

Larvae construct filtering nets of silk that are used to capture suspended particulate organic material in the water column. Different genera and species have nets of different mesh size, thus effectively partitioning the resource (Wiggins 1996). Species with nets of larger mesh size are typically found in smaller streams with coarser particulate matter, whereas species with nets of smaller mesh are found in larger rivers with greater accumulations of fine particulate organic matter. Some large river species, however, can become very common in smaller rivers that have been disturbed by agricultural runoff (Houghton 2004b).

Adults range 5–18 mm in length. Wings are typically brown with darker reticulations, although some are uniformly brown and 1 species is straw-colored. Specimens can be very abundant in light traps, especially below impoundments with high seston loads. Females are usually much more abundant than males and, unfortunately, not readily identifiable. Thus, hydropsychid species may be considerably more abundant than reported. Monson (1997) used subsampling and extrapolation to compensate for this difficulty and found that *Hydropsyche morosa* and *Cheumatopsyche pettiti* were among the five most common caddisflies of the Lake Itasca region of northcentral Minnesota.

### Genus *Cheumatopsyche*

The genus *Cheumatopsyche* contains 11 species in Minnesota. For additional species, see Gordon (1974). Larvae are typically found in medium and, especially, large rivers. Adults are some of the smallest hydropsychids, ranging 5–8 mm in length. Wings can be uniformly brown or tan, although most have a dark banding pattern. Identification of males can be difficult, and typically requires viewing the specimen both laterally and caudally.

**Figure 32.**
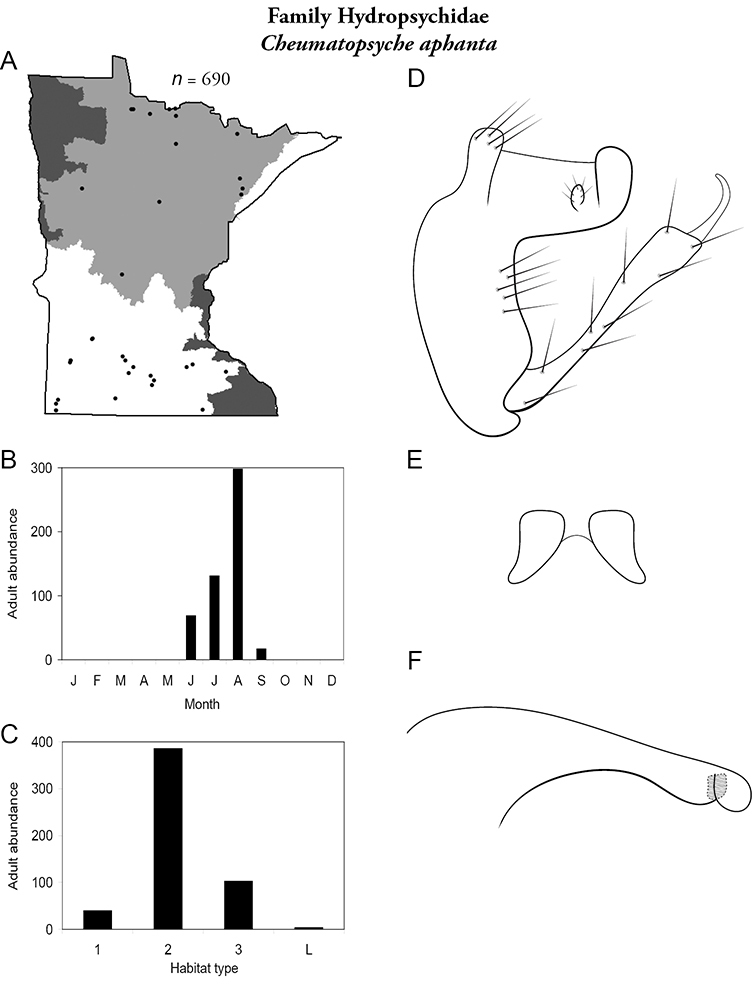
*Cheumatopsyche aphanta* **A** total specimens collected and all known collecting localities (Figure 4) **B** monthly adult abundance (1980s to present) **C** habitat preference (1980s to present) (Table 1) **D** male genital capsule **E** lobes of tergum X (caudal view) **F** phallus.

***Cheumatopsyche aphanta*** (Figure 32) has been collected from throughout the Northern and Southern Regions. Adults were present from June to September and especially abundant in August. It was found in all sizes of streams, particularly medium rivers.

**Figure 33.**
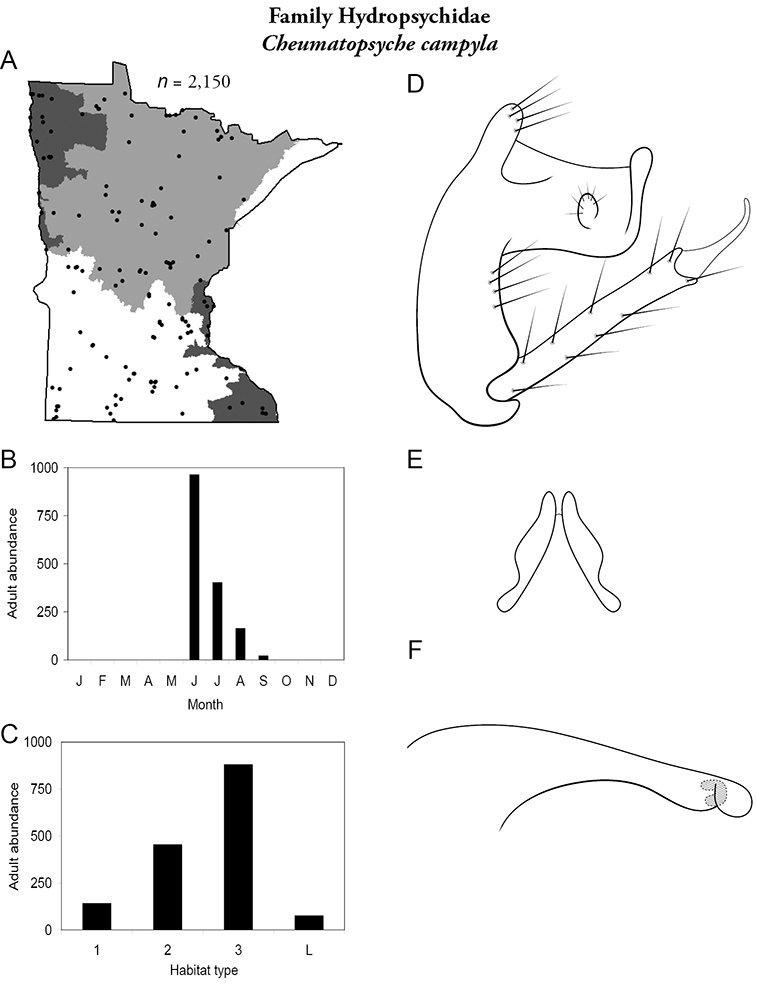
*Cheumatopsyche campyla* **A** total specimens collected and all known collecting localities (Figure 4) **B** monthly adult abundance (1980s to present) **C** habitat preference (1980s to present) (Table 1) **D** male genital capsule **E** lobes of tergum X (caudal view) **F** phallus.

***Cheumatopsyche campyla*** (Figure 33) was common and abundant in all regions except the Lake Superior. Adults were most abundant in June, with decreasing presence through September. Specimens were most abundant in large rivers. In areas of agricultural disturbance, however, *Cheumatopsyche campyla* greatly increased in abundance, constituting an “indicator species” of disturbed small and medium streams (Houghton 2004b). This situation was very common in the Northwestern and, especially, the Southern Region (Houghton 2007).

**Figure 34.**
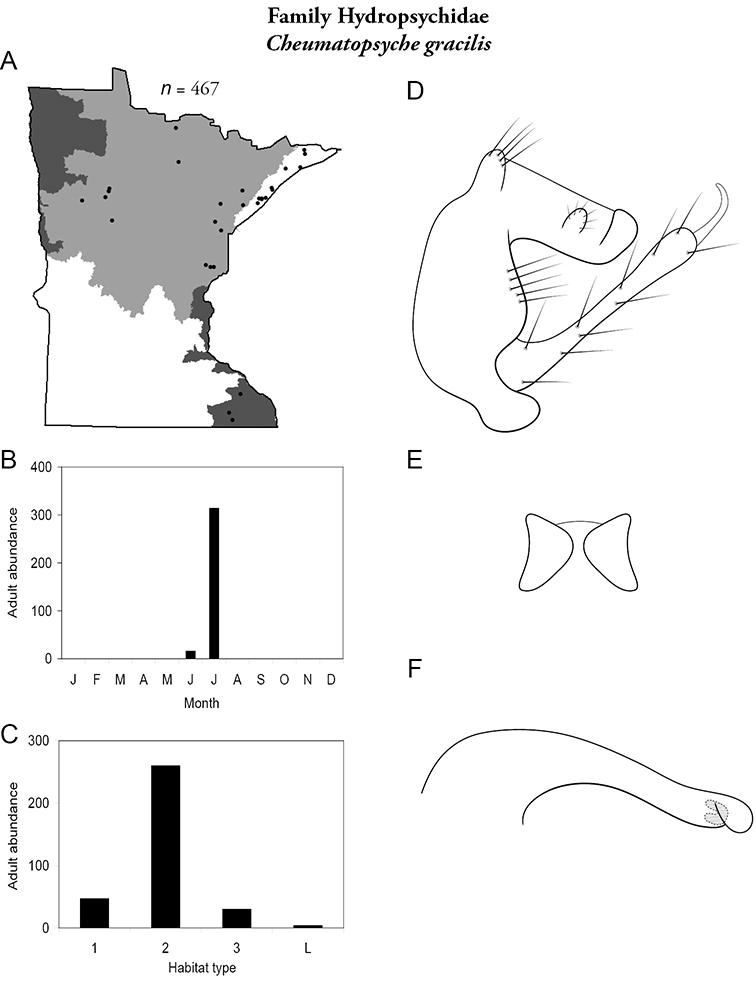
*Cheumatopsyche gracilis* **A** total specimens collected and all known collecting localities (Figure 4) **B** monthly adult abundance (1980s to present) **C** habitat preference (1980s to present) (Table 1) **D** male genital capsule **E** lobes of tergum X (caudal view) **F** phallus.

***Cheumatopsyche gracilis*** (Figure 34) was collected in the Lake Superior, Northern, and Southeastern Regions. It was most abundant in medium rivers and found mainly in July.

**Figure 35.**
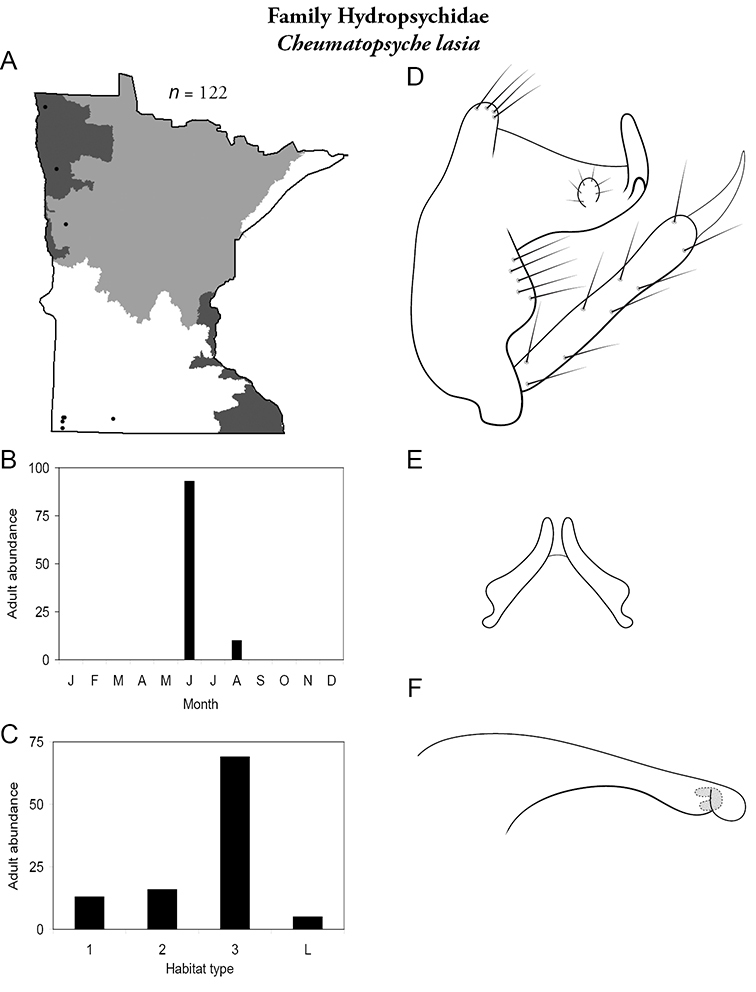
*Cheumatopsyche lasia* **A** total specimens collected and all known collecting localities (Figure 4) **B** monthly adult abundance (1980s to present) **C** habitat preference (1980s to present) (Table 1) **D** male genital capsule **E** Lobes of tergum X (caudal view) **F** phallus.

***Cheumatopsyche lasia*** (Figure 35) is known only from the northwest and southwest corners of the state. It was found mainly in large rivers. Adults were abundant in June, with some present in August.

**Figure 36.**
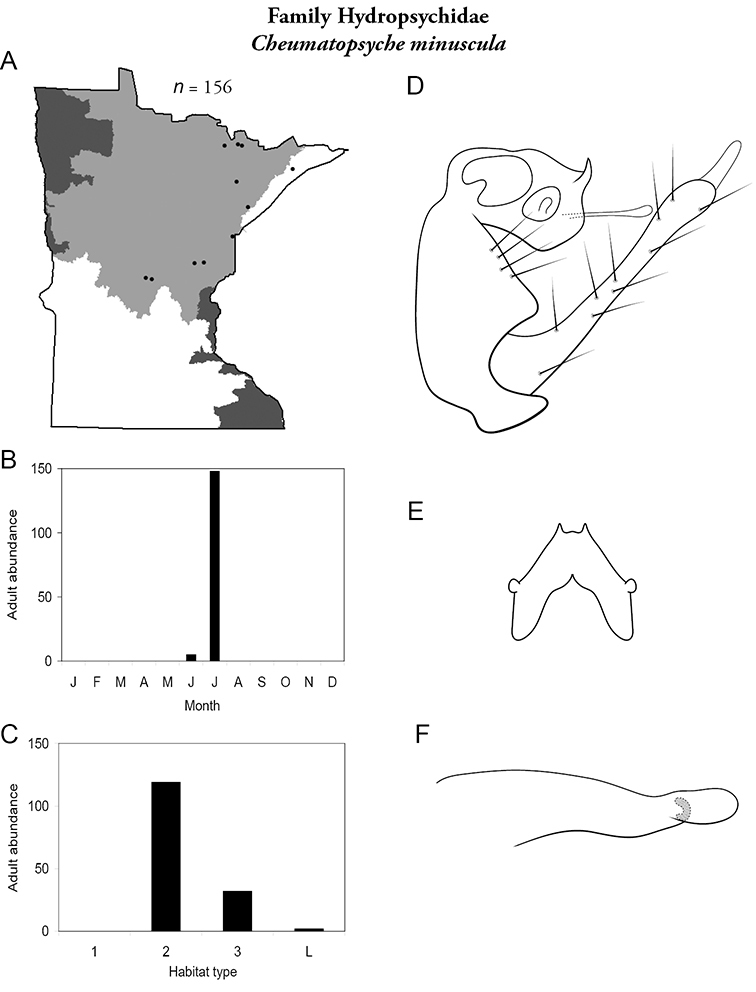
*Cheumatopsyche miniscula* **A** total specimens collected and all known collecting localities (Figure 4) **B** monthly adult abundance (1980s to present) **C** habitat preference (1980s to present) (Table 1) **D** male genital capsule **E** Lobes of tergum X (caudal view) **F** phallus.

***Cheumatopsyche minuscula*** (Figure 36) has been collected in the Lake Superior and Northern Regions, almost exclusively in July. It was found mostly in medium rivers, with some specimens found in large rivers.

**Figure 37.**
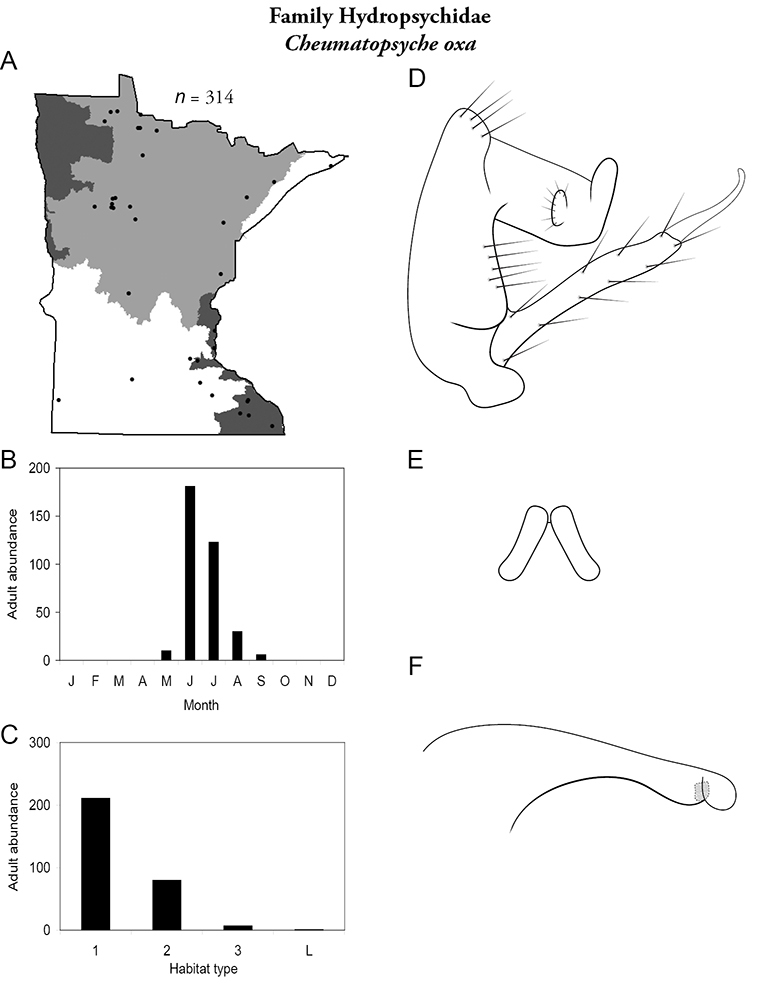
*Cheumatopsyche oxa* **A** total specimens collected and all known collecting localities (Figure 4) **B** monthly adult abundance (1980s to present) **C** habitat preference (1980s to present) (Table 1) **D** male genital capsule **E** Lobes of tergum X (caudal view) **F** phallus.

***Cheumatopsyche oxa*** (Figure 37) has been found in all regions except the Northwestern. It was not particularly abundant, however, especially when compared to some of its congeners. Adults were present from May to September, with a greatest abundance in June and July. It was found mainly in small streams and occasionally in medium rivers.

**Figure 38.**
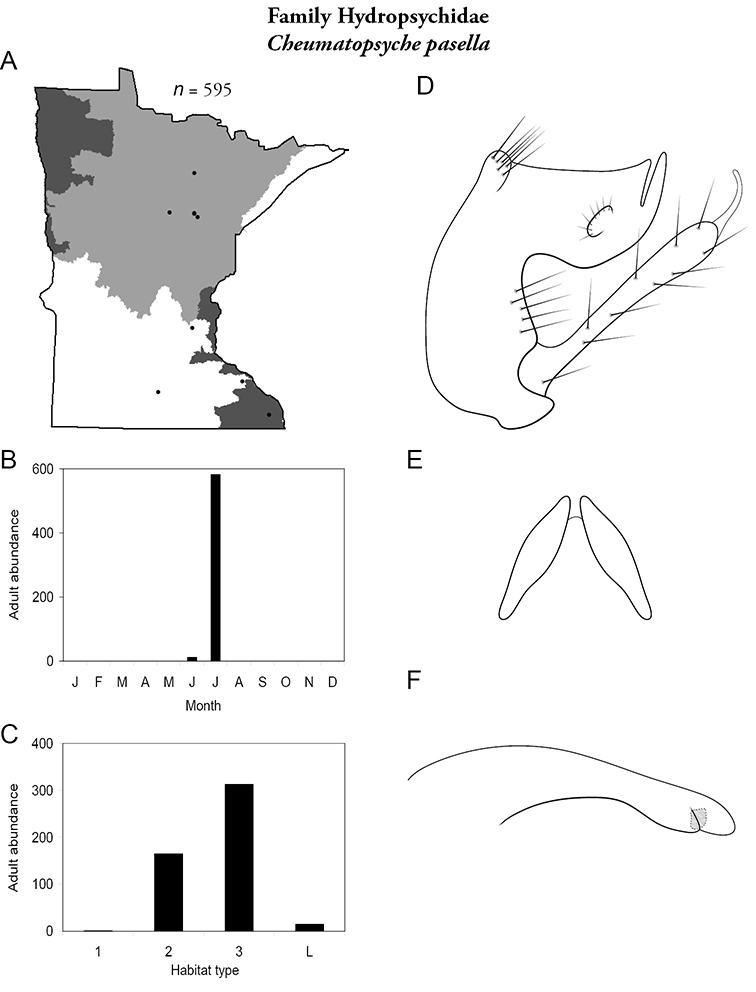
*Cheumatopsyche pasella* **A** total specimens collected and all known collecting localities (Figure 4) **B** monthly adult abundance (1980s to present) **C** habitat preference (1980s to present) (Table 1) **D** male genital capsule **E** Lobes of tergum X (caudal view) **F** phallus.

***Cheumatopsyche pasella*** (Figure 38) has been found sporadically in the Northern, Southeastern, and Southern Regions. It was found almost exclusively in July, and mostly in medium and large rivers. It was the 2nd most abundant species of large rivers of the Southeastern Region (Table 6).

**Figure 39.**
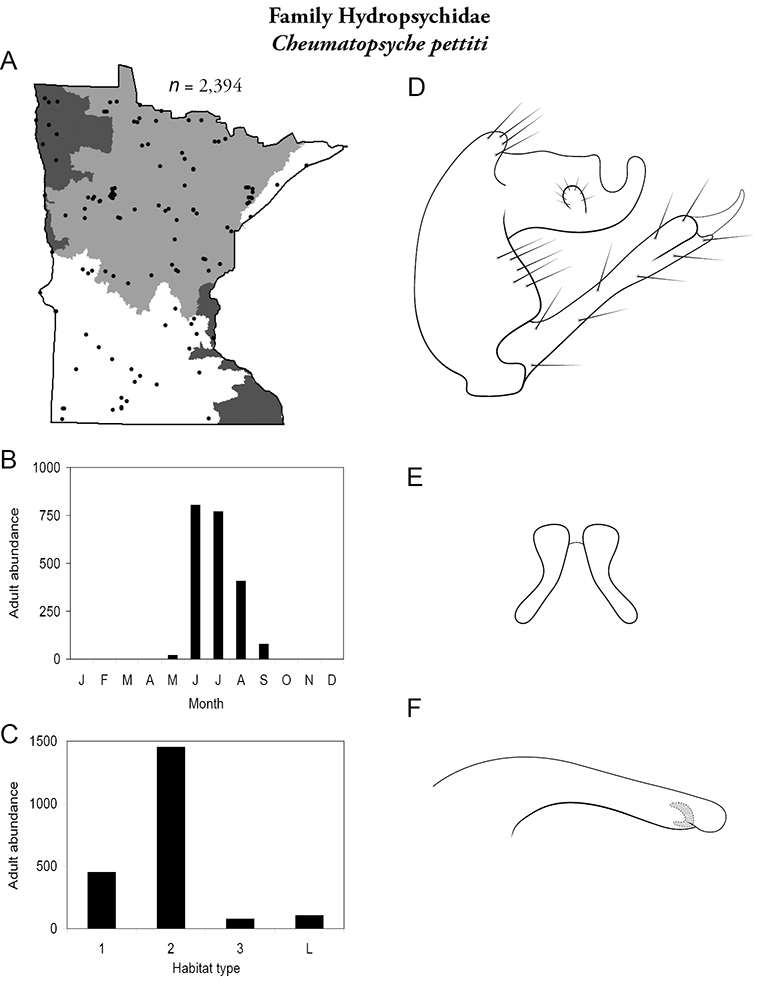
*Cheumatopsyche pettiti* **A** total specimens collected and all known collecting localities (Figure 4) **B** monthly adult abundance (1980s to present) **C** habitat preference (1980s to present) (Table 1) **D** male genital capsule **E** Lobes of tergum X (caudal view) **F** phallus.

***Cheumatopsyche pettiti*** (Figure 39) was the most widespread *Cheumatopsyche* species, collected throughout the state from May to September, and abundant from June through August. It was most abundant in small and, especially, medium rivers.

**Figure 40.**
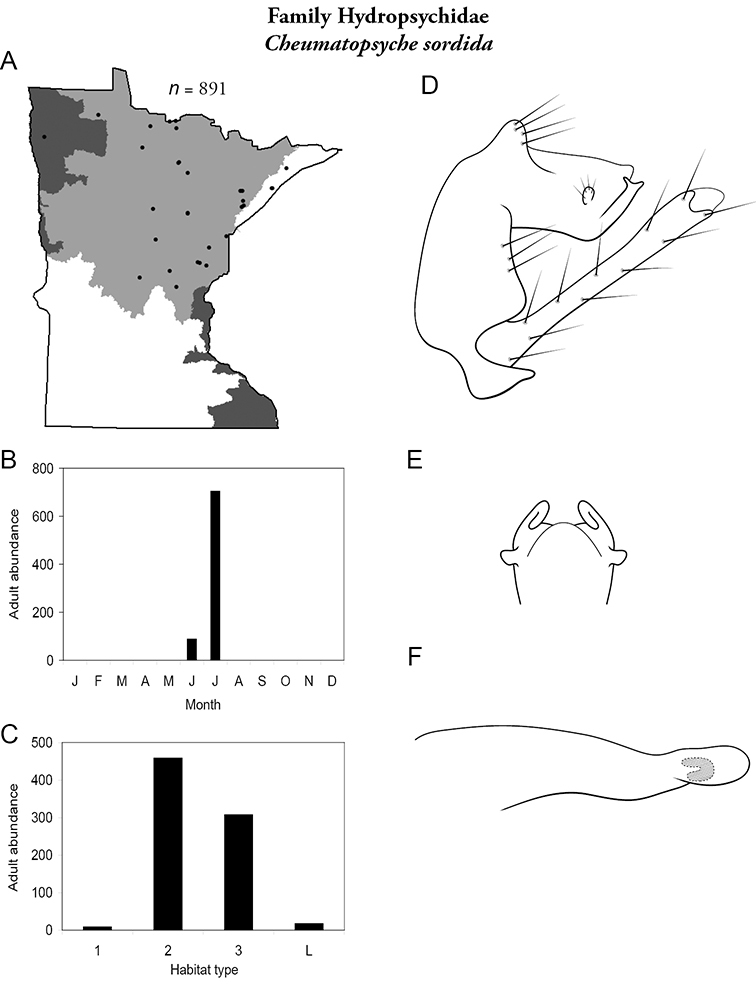
*Cheumatopsyche sordida* **A** total specimens collected and all known collecting localities (Figure 4) **B** monthly adult abundance (1980s to present) **C** habitat preference (1980s to present) (Table 1) **D** male genital capsule **E** Lobes of tergum X (caudal view) **F** phallus.

***Cheumatopsyche sordida*** (Figure 40) is known predominantly from the Northern Region, with occasional collections in the Lake Superior and Northwestern Regions. It was found almost exclusively in medium and large rivers. Adults were present in June and abundant in July.

**Figure 41.**
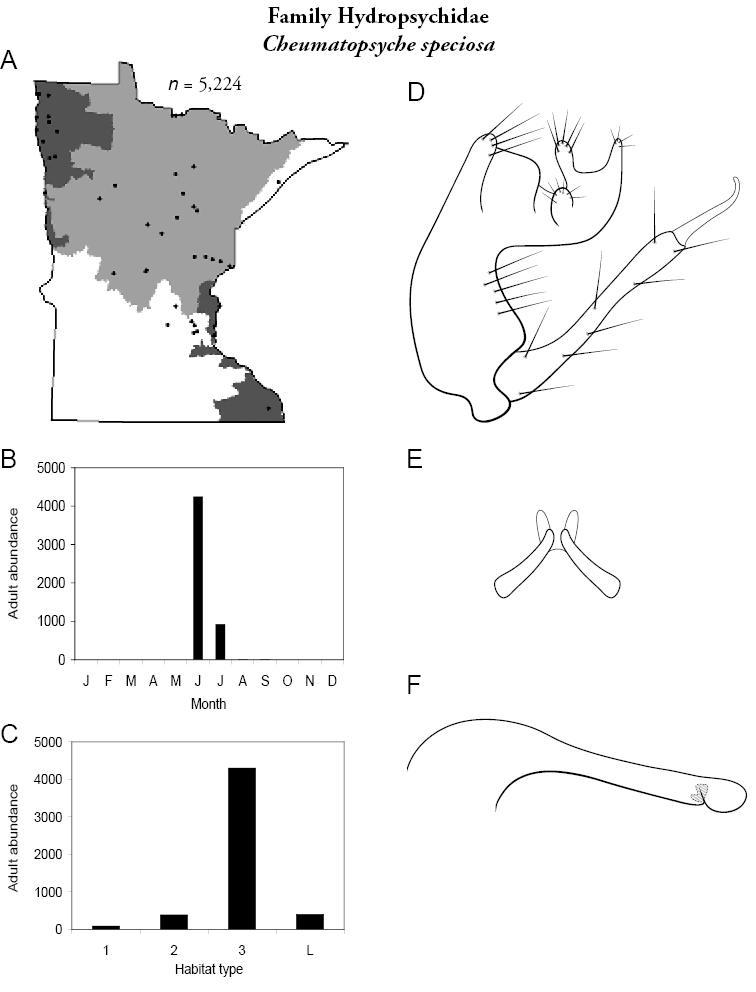
*Cheumatopsyche speciosa* **A** total specimens collected and all known collecting localities (Figure 4) **B** monthly adult abundance (1980s to present) **C** habitat preference (1980s to present) (Table 1) **D** male genital capsule **E** Lobes of tergum X (caudal view) **F** phallus.

***Cheumatopsyche speciosa*** (Figure 41) has been collected in all regions, but was especially abundant in the Northwestern Region. Overall, the species was most abundant in large rivers. Adults were most abundant in June and present in July. This species is the smallest of the *Cheumatopsyche*; adults are around 5 mm in length.

**Figure 42.**
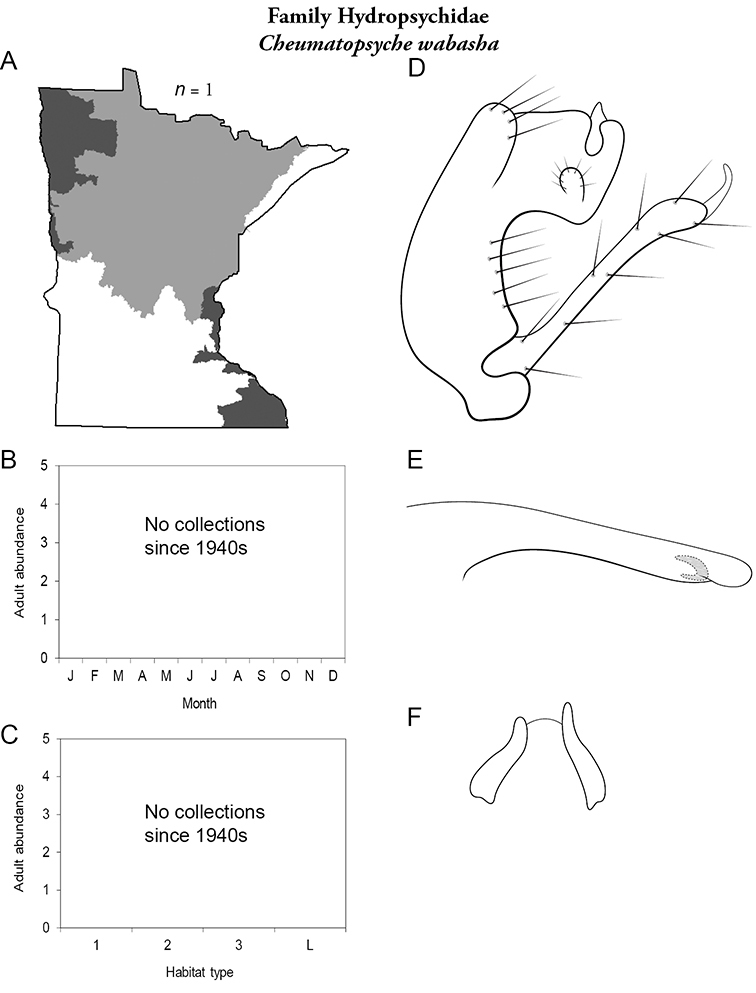
*Cheumatopsyche wabasha* **A** total specimens collected and all known collecting localities (Figure 4) **B** monthly adult abundance (1980s to present) **C** habitat preference (1980s to present) (Table 1) **D** male genital capsule **E** Lobes of tergum X (caudal view) **F** phallus.

***Cheumatopsyche wabasha*** (Figure 42) was described from a specimen collected in the city of Wabasha during July 1941. The species has not been seen in Minnesota since this holotype collection. It has, however, been collected in Oregon and Tennessee (Nimmo 1987). The species is unique in having lobes of tergum X of different size. The odd morphology and distribution of *Cheumatopsyche wabasha* has led some workers (Gordon 1974, Nimmo 1987) to speculate that specimens of the species may be aberrant members of another species, perhaps *Cheumatopsyche pettiti*. Gordon (1974) ultimately considered it a valid species, however, so it is included in this manual.

### Genus *Diplectrona*

The genus *Diplectrona* contains a single species in Minnesota. For additional species, see Morse and Barr (1990). Larvae are characteristic of cold, small streams. Larval retreats are similar to those of *Hydropsyche*, although incorporating more plants material and less sand (Wiggins 1996). Adults range 8–10 mm in length and have grey and dark brown mottled wings.

**Figure 43.**
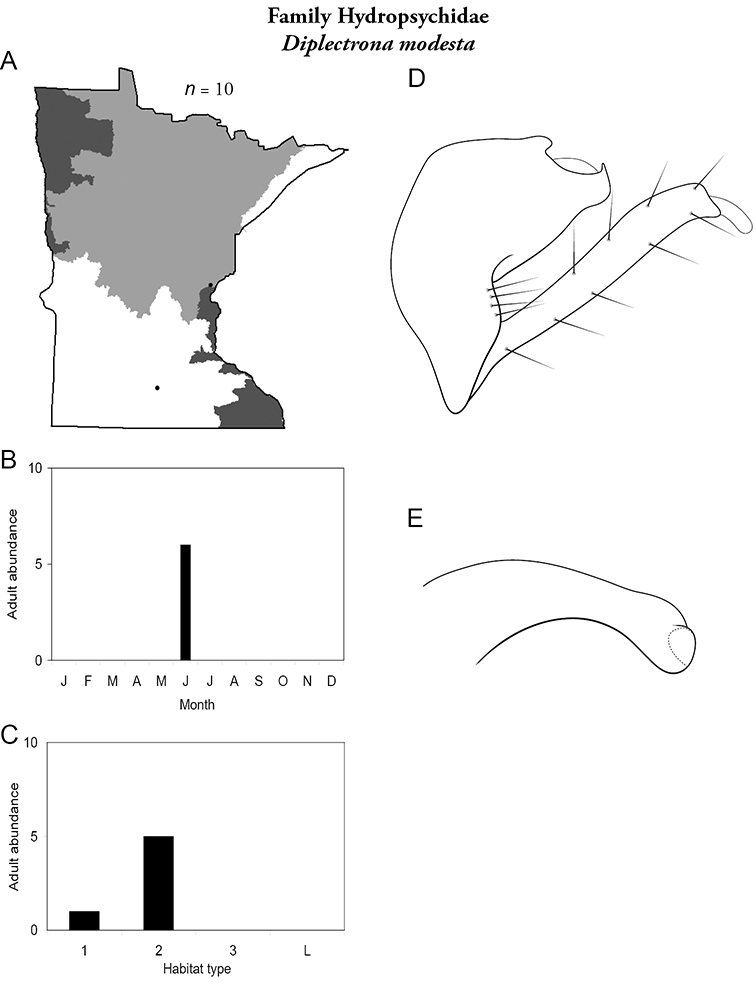
*Diplectrona modesta* **A** total specimens collected and all known collecting localities (Figure 4) **B** monthly adult abundance (1980s to present) **C** habitat preference (1980s to present) (Table 1) **D** male genital capsule **E** phallus.

***Diplectrona modesta*** (Figure 43) is known only from a small and medium river in Minneopa State Park in the Southern Region, and from a small unnamed spring in the Northern Region. All specimens were collected during June.

### Genus *Hydropsyche*

The genus *Hydropsyche* contains 17 species in Minnesota. It is the 3rd most species-rich genus (Figure 7). For additional species, see Nimmo (1987) or Schefter et al. (1986). There is some controversy as to whether species sometimes treated as the *Ceratopsyche* should constitute a separate genus, a subgenus within the *Hydropsyche*, or eliminated as a taxon altogether (e.g., Schuster 1984, Schefter et al. 1986, Geraci et al. 2010). While it is not the purpose of this manual to address systematic questions, species of the “*Ceratopsyche*” do have a genitalic morphology distinctly different than those of the *Hydropsyche sensu stricto*. Thus, species in the former group, while considered to be in *Hydropsyche* for organizational purposes, are designated with a “C”.

Larvae of the *Hydropsyche* are very conspicuous on the undersides of medium and large rocks in nearly any stream. Species are more likely to be in smaller streams than *Cheumatopsyche*, but there are many exceptions. Adults are 8–14 mm in length. Some species have uniformly brown or grey wings. Others include a darker mottled pattern (Figure 290). Separation of males of *Hydropsyche bidens*, *Hydropsyche scalaris*, *Hydropsyche simulans*, and *Hydropsyche orris* requires very careful examination of the tip of the phallus.

**Figure 44.**
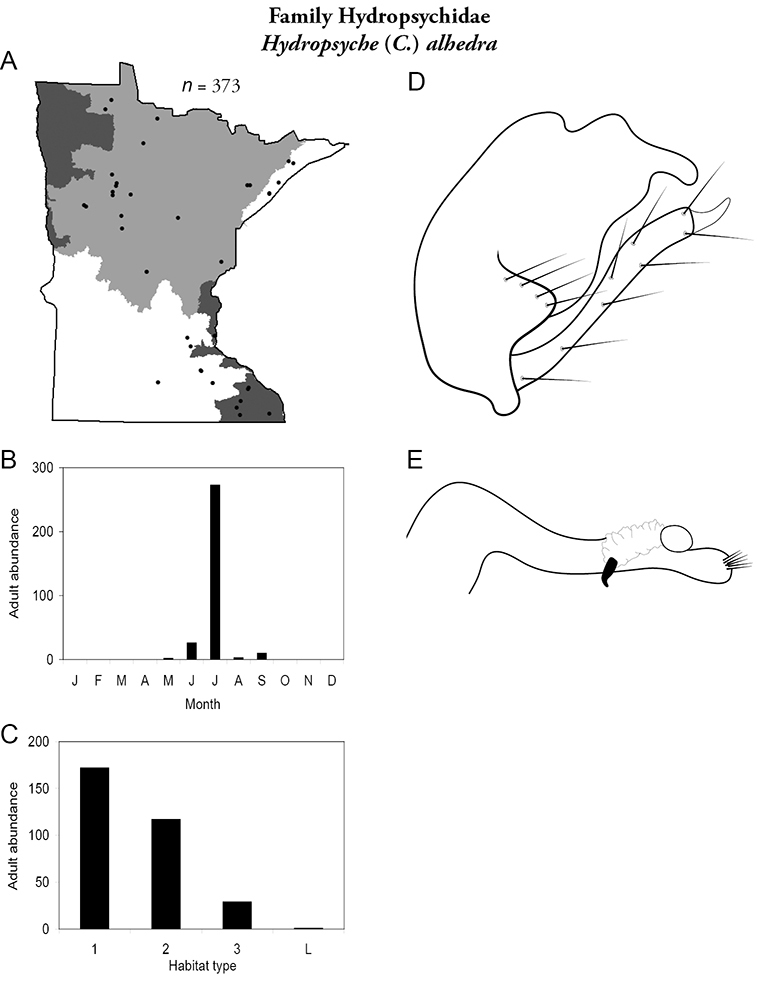
*Hydropsyche alhedra* **A** total specimens collected and all known collecting localities (Figure 4) **B** monthly adult abundance (1980s to present) **C** habitat preference (1980s to present) (Table 1) **D** male genital capsule **E** phallus.

***Hydropsyche (C.) alhedra*** (Figure 44) has been found in all regions except the Northwestern. It was collected from all sizes of streams, especially small and medium streams. Adults were present from May to September, but abundant only in July.

**Figure 45.**
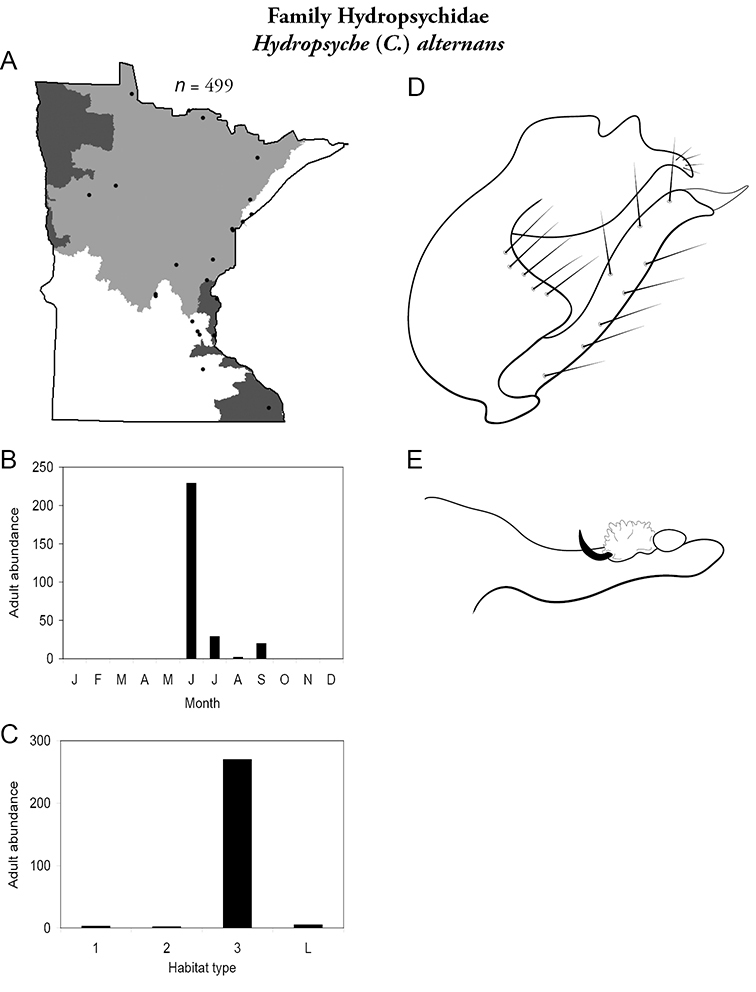
*Hydropsyche alternans* **A** total specimens collected and all known collecting localities (Figure 4) **B** monthly adult abundance (1980s to present) **C** habitat preference (1980s to present) (Table 1) **D** male genital capsule **E** phallus.

***Hydropsyche (C.) alternans*** (Figure 45) is known from the Northern, Southeastern, and Southern Regions, almost exclusively from large rivers. Adults were abundant in June and present from July to September.

**Figure 46.**
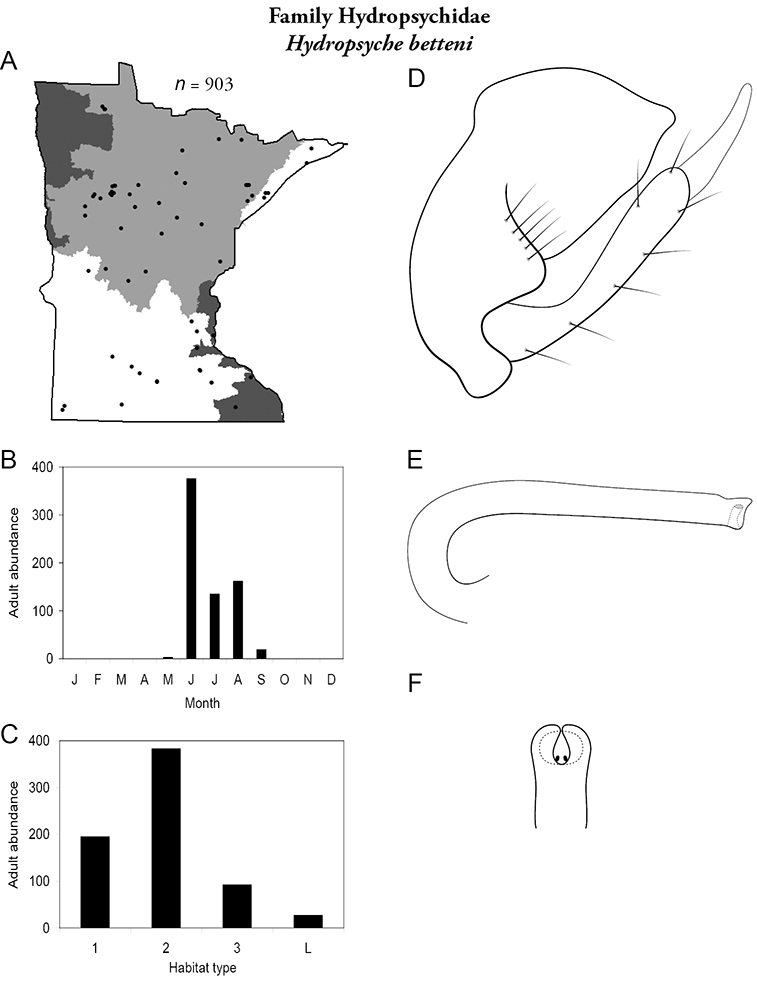
*Hydropsyche betteni* **A** total specimens collected and all known collecting localities (Figure 4) **B** monthly adult abundance (1980s to present) **C** habitat preference (1980s to present) (Table 1) **D** male genital capsule **E** phallus **F** apical tip of phallus (dorsal view).

***Hydropsyche betteni*** (Figure 46) has been found in all regions except the Northwestern. It was most abundant in medium rivers, but also found in small streams and large rivers. Some adults were collected in May and September; the majority were found from June through August.

**Figure 47.**
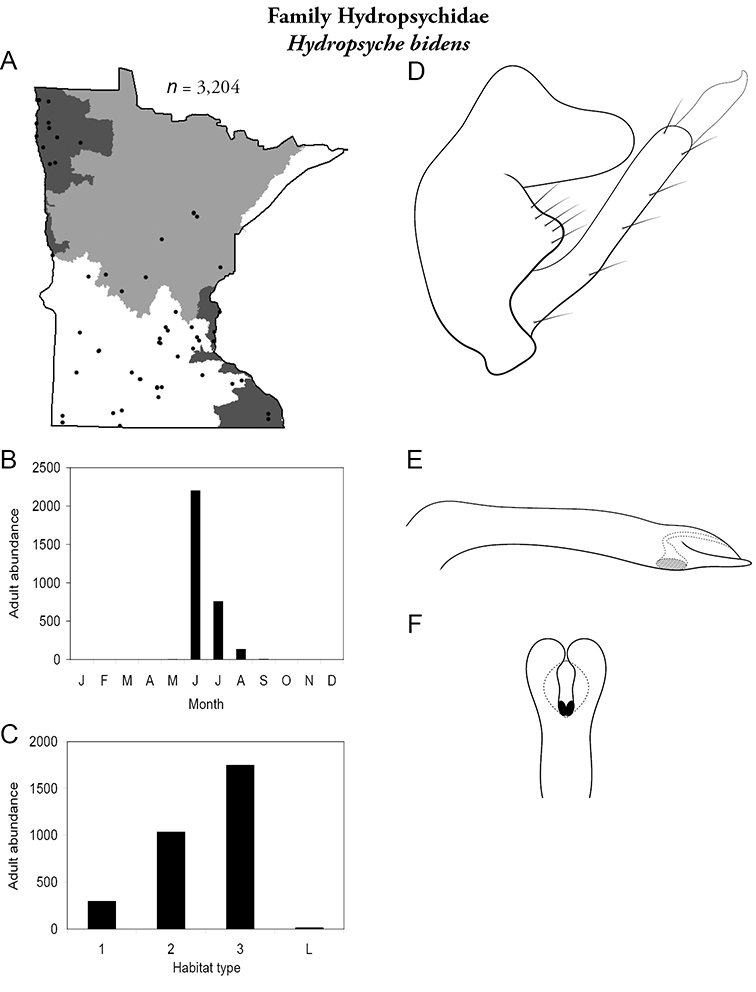
*Hydropsyche bidens* **A** total specimens collected and all known collecting localities (Figure 4) **B** monthly adult abundance (1980s to present) **C** habitat preference (1980s to present) (Table 1) **D** male genital capsule **E** phallus **F** apical tip of phallus (dorsal view).

***Hydropsyche bidens*** (Figure 47) is known from all regions except the Lake Superior. It was most abundant in medium and, especially, large rivers. It was also, however, the most abundant species in small streams of the Northwestern Region (Table 5). Adults were most abundant in June and also found in July and August.

**Figure 48.**
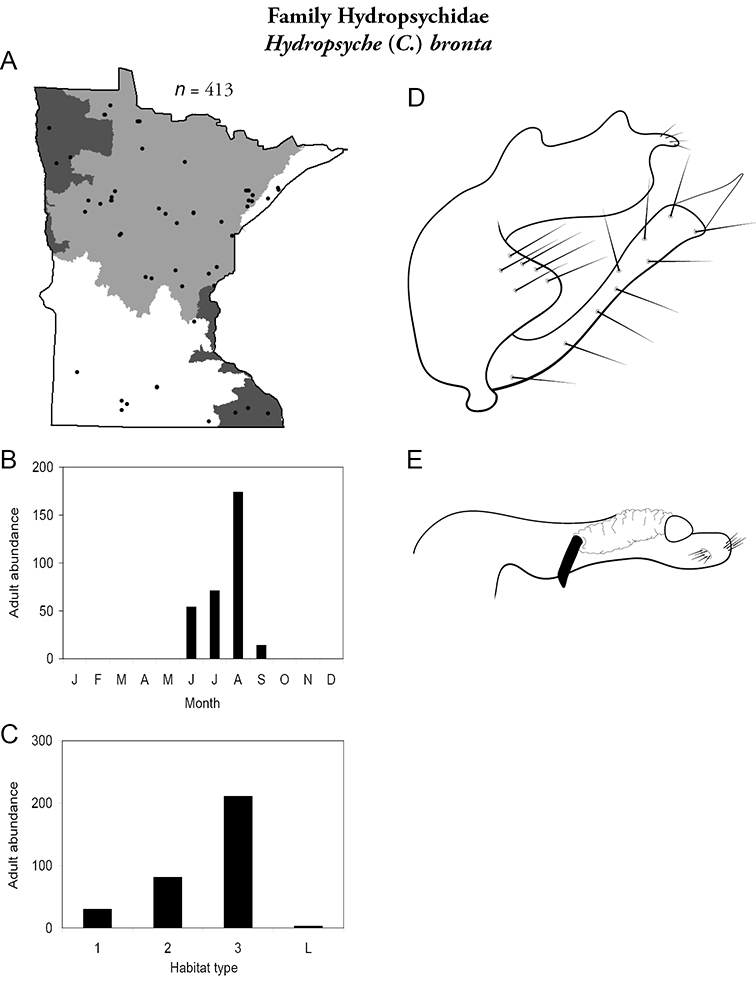
*Hydropsyche bronta* **A** total specimens collected and all known collecting localities (Figure 4) **B** monthly adult abundance (1980s to present) **C** habitat preference (1980s to present) (Table 1) **D** male genital capsule **E** phallus.

***Hydropsyche (C.) bronta*** (Figure 48) has been collected in all regions, but was not particularly abundant. Adults were most abundant in August, with specimens present June through September. It was found in all sizes of streams, especially large rivers.

**Figure 49.**
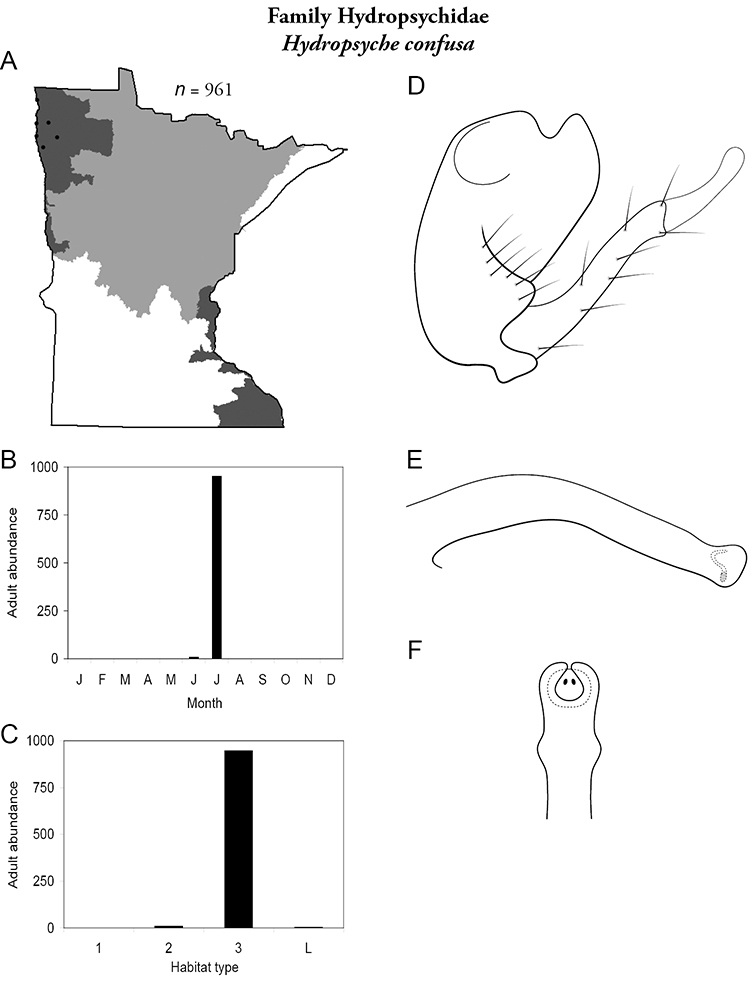
*Hydropsyche confusa* **A** total specimens collected and all known collecting localities (Figure 4) **B** monthly adult abundance (1980s to present) **C** habitat preference (1980s to present) (Table 1) **D** male genital capsule **E** phallus **F** apical tip of phallus (dorsal view).

***Hydropsyche confusa*** (Figure 49) is the only species of caddisfly currently found exclusively in the Northwestern Region. Adults were found in July from large rivers. The species was the second most abundant caddisfly in large rivers of the Northwestern Region (Table 5).

**Figure 50.**
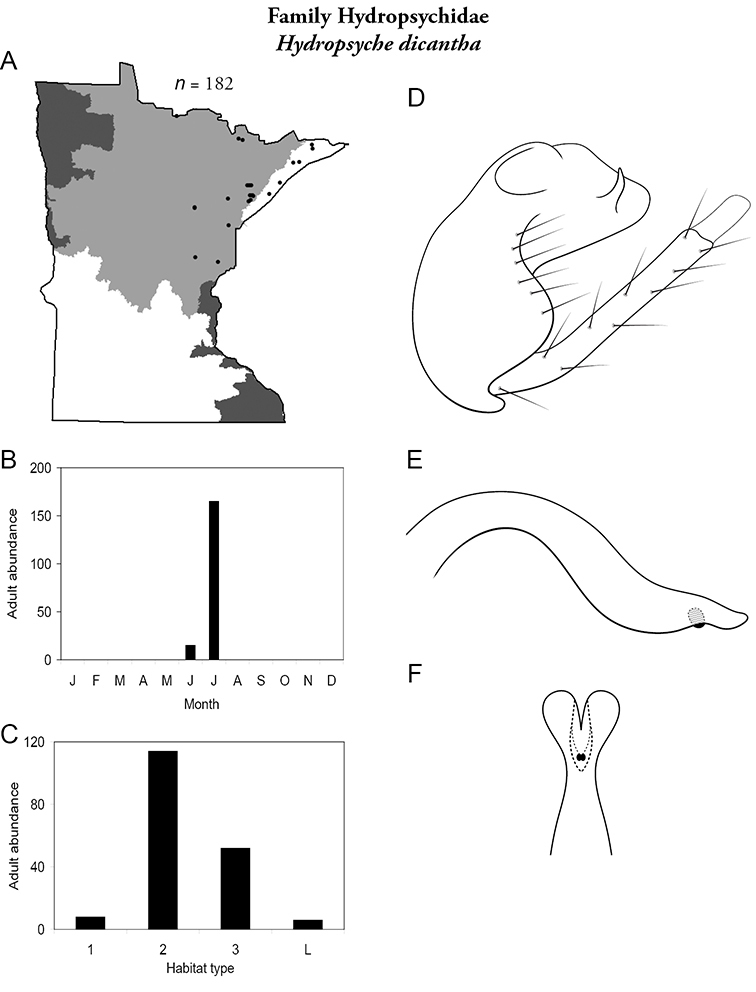
*Hydropsyche dicantha* **A** total specimens collected and all known collecting localities (Figure 4) **B** monthly adult abundance (1980s to present) **C** habitat preference (1980s to present) (Table 1) **D** male genital capsule **E** phallus **F** apical tip of phallus (dorsal view).

***Hydropsyche dicantha*** (Figure 50) is known from the Lake Superior and Northern regions. It was most abundant in large and, especially, medium rivers. Adults were collected primarily in July.

**Figure 51.**
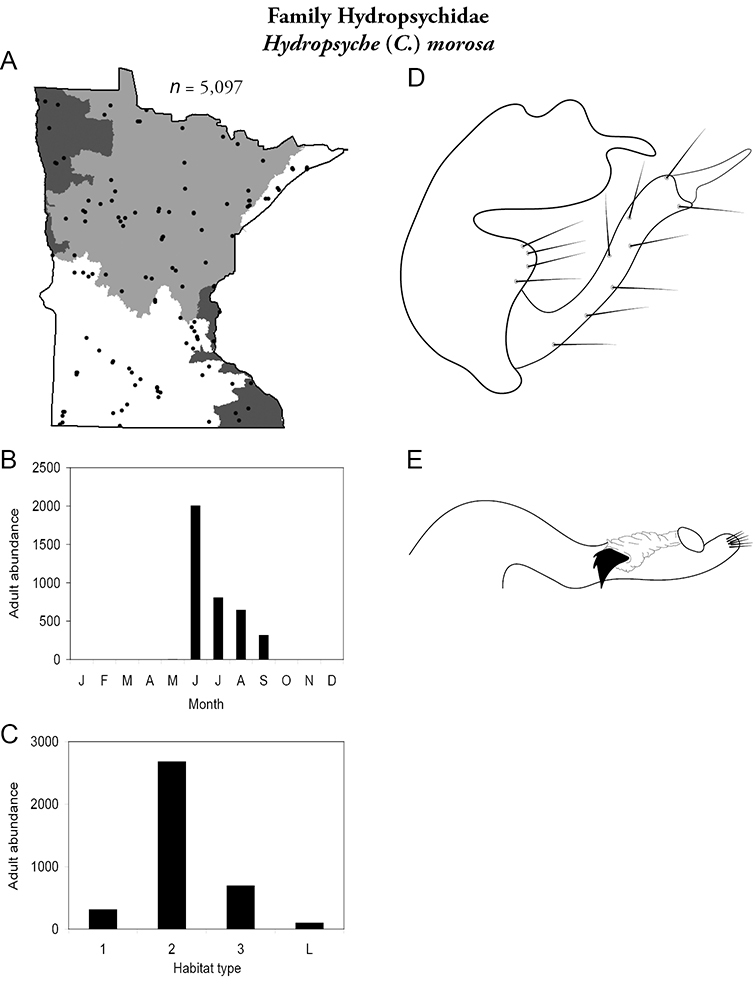
*Hydropsyche morosa* **A** total specimens collected and all known collecting localities (Figure 4) **B** monthly adult abundance (1980s to present) **C** habitat preference (1980s to present) (Table 1) **D** male genital capsule **E** phallus.

***Hydropsyche (C.) morosa*** (Figure 51) was common throughout the state and found from June through September. It was most abundant in medium rivers.

**Figure 52.**
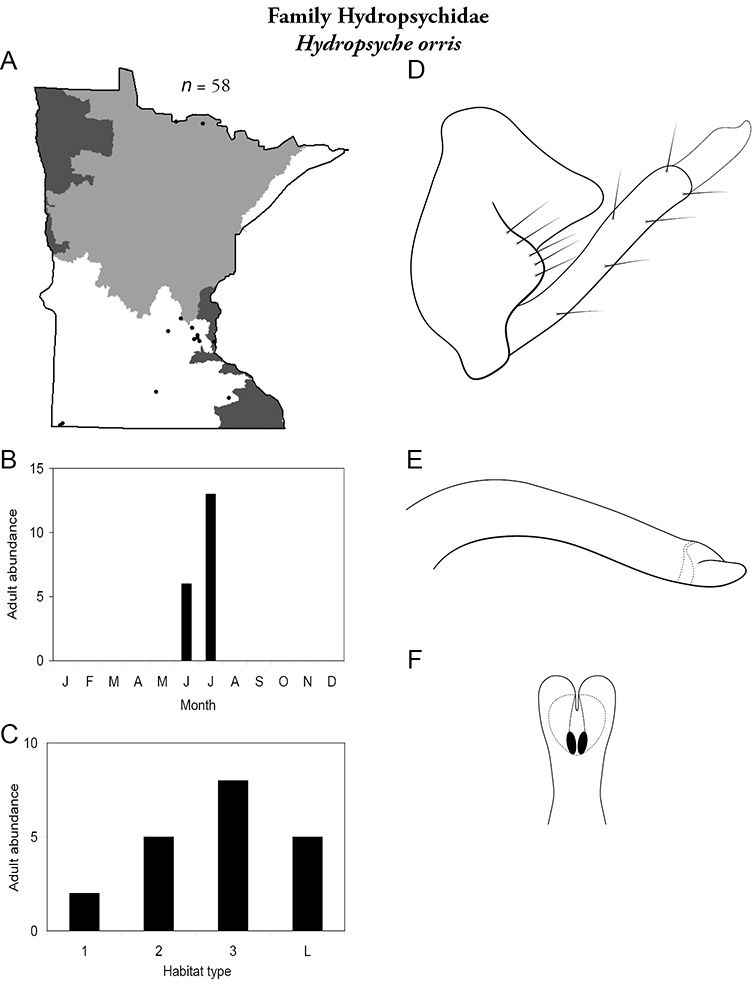
*Hydropsyche orris* **A** total specimens collected and all known collecting localities (Figure 4) **B** monthly adult abundance (1980s to present) **C** habitat preference (1980s to present) (Table 1) **D** male genital capsule **E** phallus **F** apical tip of phallus (dorsal view).

***Hydropsyche orris*** (Figure 52) was collected primarily from the Southern Region, with a couple of collections from large rivers of the Northern Region. Adults were found in June and July from all habitat types, including lakes. Specimens, however, were not particularly abundant.

**Figure 53.**
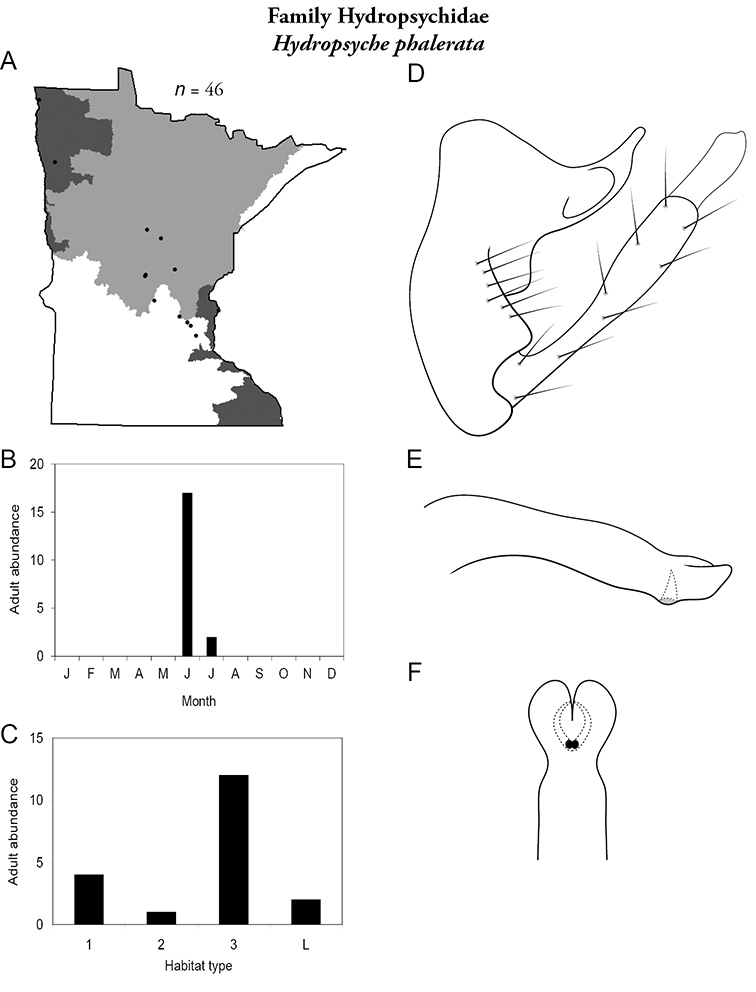
*Hydropsyche phalerata* **A** total specimens collected and all known collecting localities (Figure 4) **B** monthly adult abundance (1980s to present) **C** habitat preference (1980s to present) (Table 1) **D** male genital capsule **E** phallus **F** apical tip of phallus (dorsal view).

***Hydropsyche phalerata*** (Figure 53) has been found primarily in large rivers sporadically throughout the state, except for in the Lake Superior Region. Nearly all adults were collected in June.

**Figure 54.**
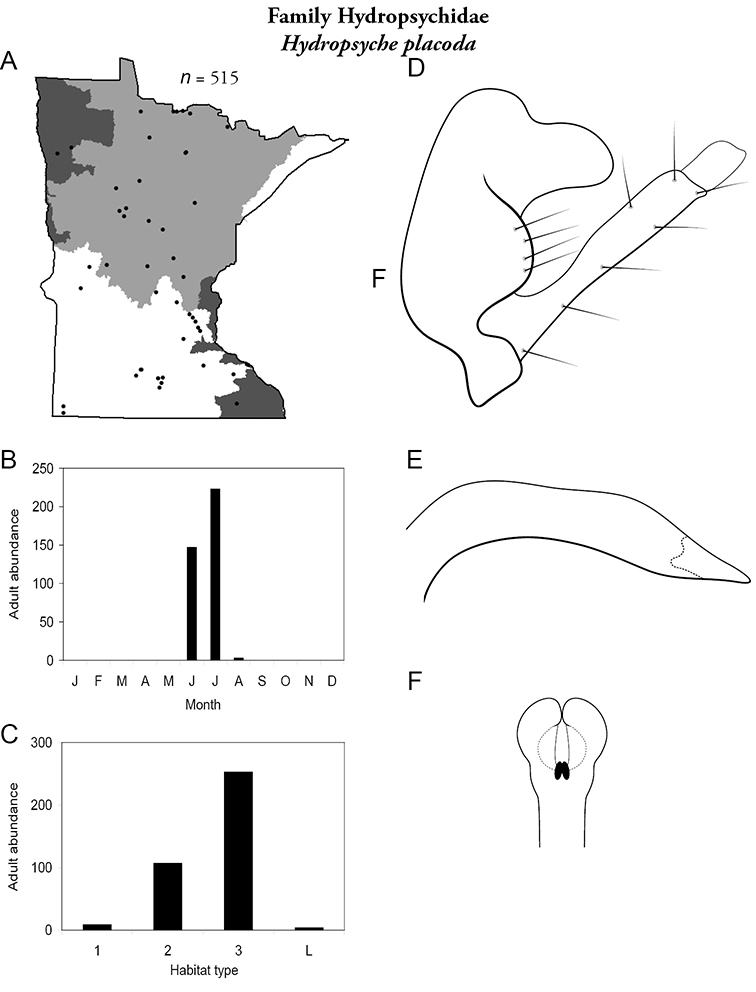
*Hydropsyche placoda* **A** total specimens collected and all known collecting localities (Figure 4) **B** monthly adult abundance (1980s to present) **C** habitat preference (1980s to present) (Table 1) **D** male genital capsule **E** phallus **F** apical tip of phallus (dorsal view).

***Hydropsyche placoda*** (Figure 54) is known from all regions except the Lake Superior. It was found primarily in large rivers, with some presence in medium rivers. Adults were found mostly in June and July. In addition to genitalic characteristics, males of this species can be recognized by their enlarged compound eyes.

**Figure 55.**
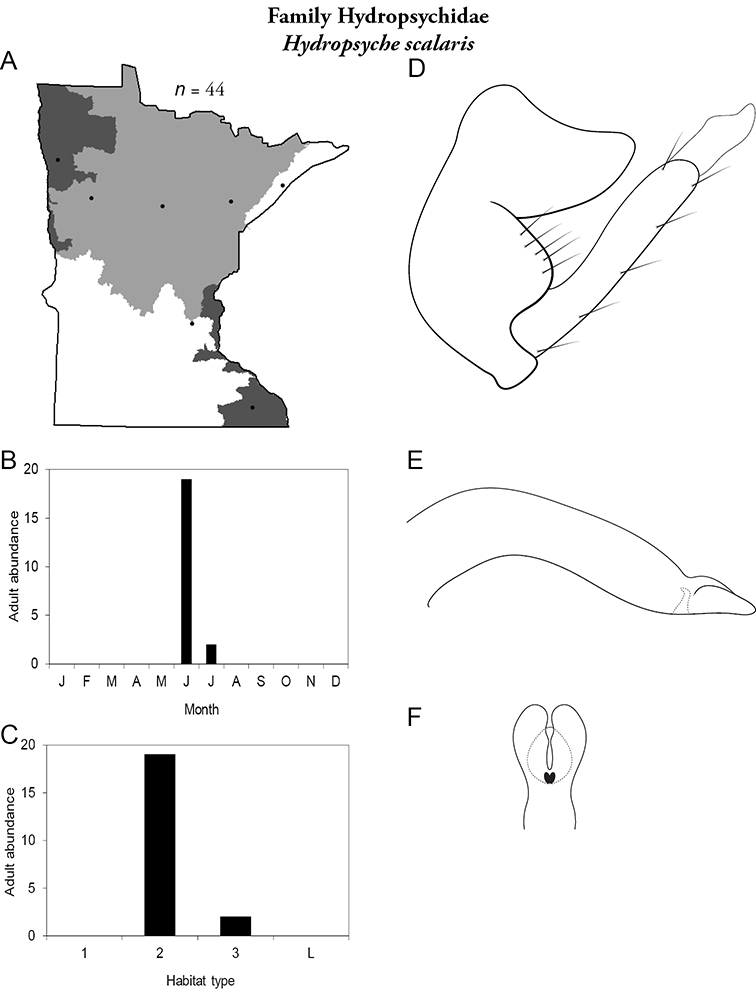
*Hydropsyche scalaris* **A** total specimens collected and all known collecting localities (Figure 4) **B** monthly adult abundance (1980s to present) **C** habitat preference (1980s to present) (Table 1) **D** male genital capsule **E** phallus **F** apical tip of phallus (dorsal view).

***Hydropsyche scalaris*** (Figure 55) has been collected sporadically throughout the state, mostly during June from medium rivers.

**Figure 56.**
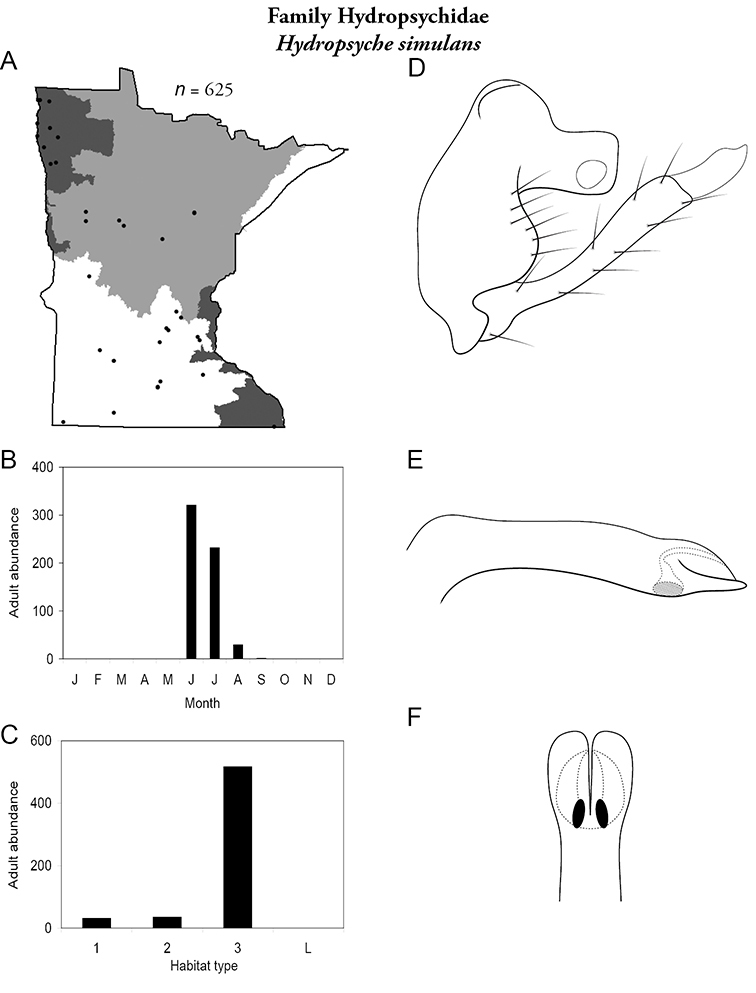
*Hydropsyche simulans* **A** total specimens collected and all known collecting localities (Figure 4) **B** monthly adult abundance (1980s to present) **C** habitat preference (1980s to present) (Table 1) **D** male genital capsule **E** phallus **F** apical tip of phallus (dorsal view).

***Hydropsyche simulans*** (Figure 56) has been collected primarily in the Northern, Northwestern, and Southern Regions. Statewide, it was found primarily in large rivers. It was, however, one of the most abundan species in allsizes of stream of the Southern Region due to excess agricultural input (Table 7). It was also determined to be an “indicator species” of habitat disturbance in small and medium streams (Houghton 2004b).

**Figure 57.**
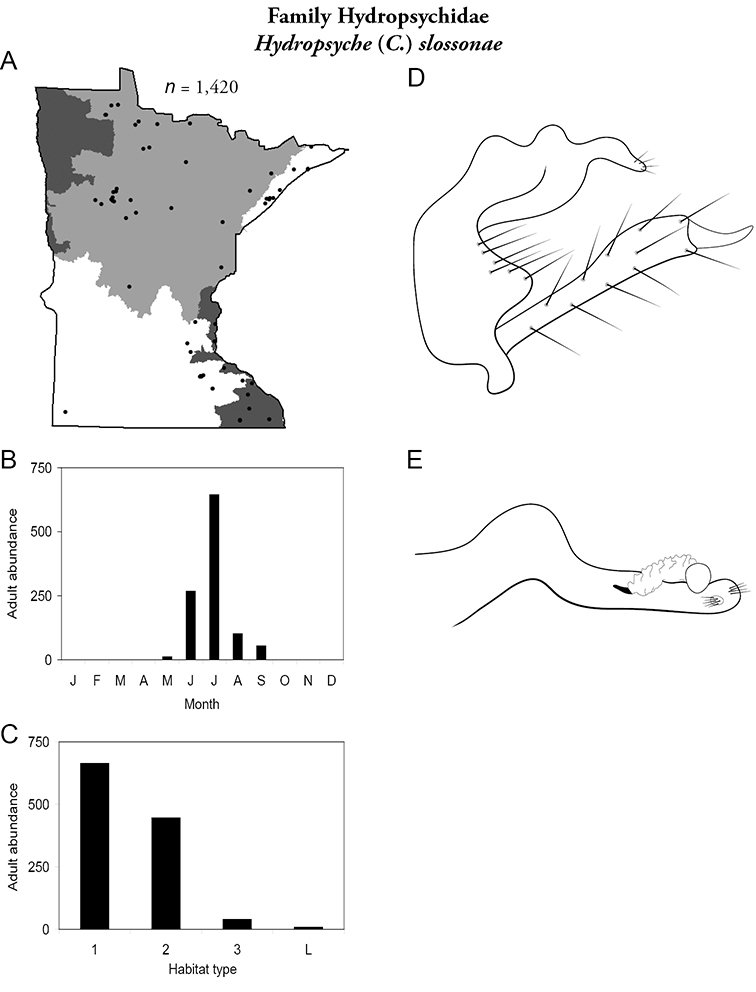
*Hydropsyche slossonae* **A** total specimens collected and all known collecting localities (Figure 4) **B** monthly adult abundance (1980s to present) **C** habitat preference (1980s to present) (Table 1) **D** male genital capsule **E** phallus.

***Hydropsyche (C.) slossonae*** (Figure 57) was found in all regions except the Northwestern. Unlike most hydropsychids, *Hydropsyche slossonae* was most abundant in small undisturbed streams. Adults were present from May to September, with greatest abundance in July.

**Figure 58.**
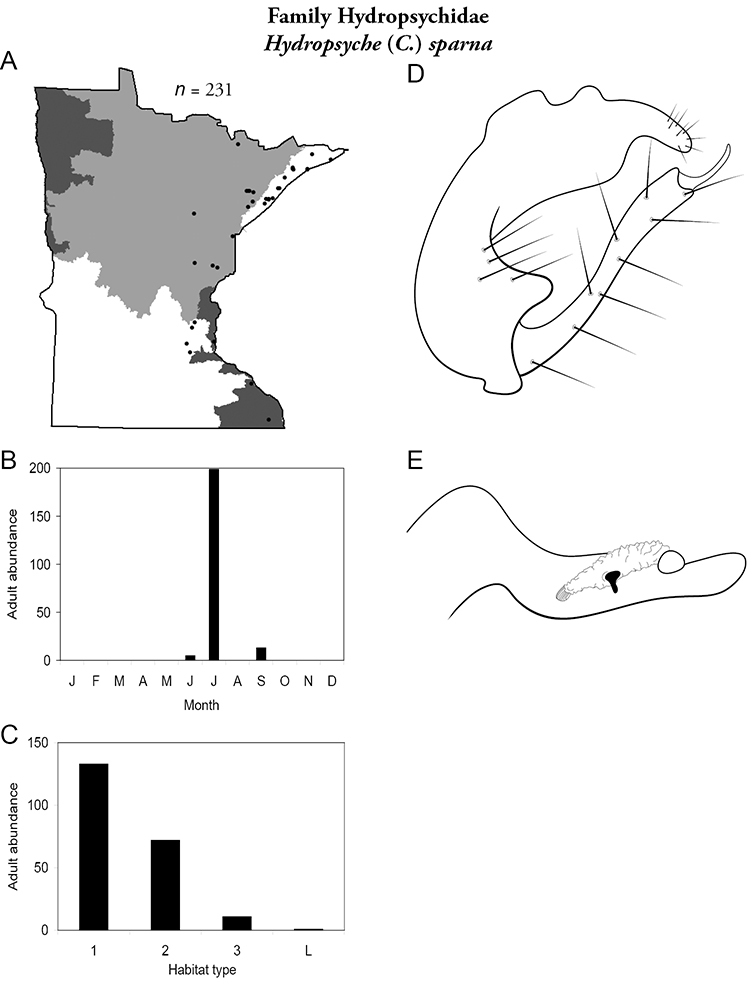
*Hydropsyche sparna* **A** total specimens collected and all known collecting localities (Figure 4) **B** monthly adult abundance (1980s to present) **C** habitat preference (1980s to present) (Table 1) **D** male genital capsule **E** phallus.

***Hydropsyche (C.) sparna*** (Figure 58) was collected from the eastern third of the state. Similar to *Hydropsyche slossonae*, *Hydropsyche sparna* was most abundant in small streams, and fairly abundant in medium rivers. It was the most abundant species of small streams in the Lake Superior Region (Table 3). Nearly all specimens were caught in July.

**Figure 59.**
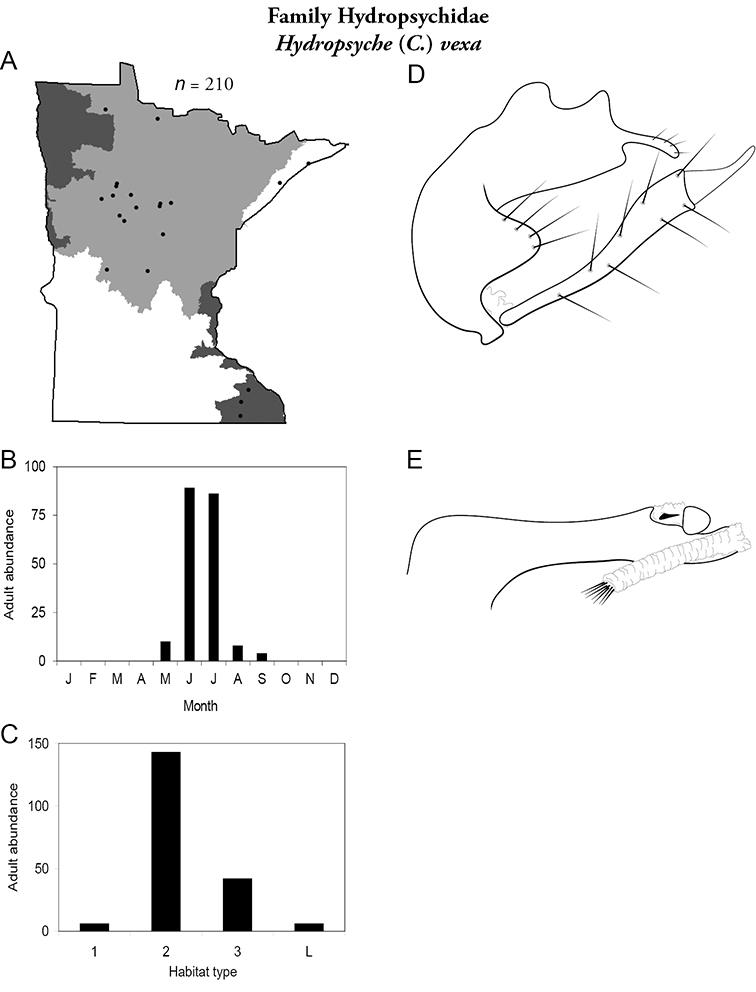
*Hydropsyche vexa* **A** total specimens collected and all known collecting localities (Figure 4) **B** monthly adult abundance (1980s to present) **C** habitat preference (1980s to present) (Table 1) **D** male genital capsule **E** phallus.

***Hydropsyche (C.) vexa*** (Figure 59) is known from the Lake Superior, Northern, and Southeastern Regions. It was most abundant in large and, especially, medium rivers. Adults were present from May to September and most abundant in June and July.

**Figure 60.**
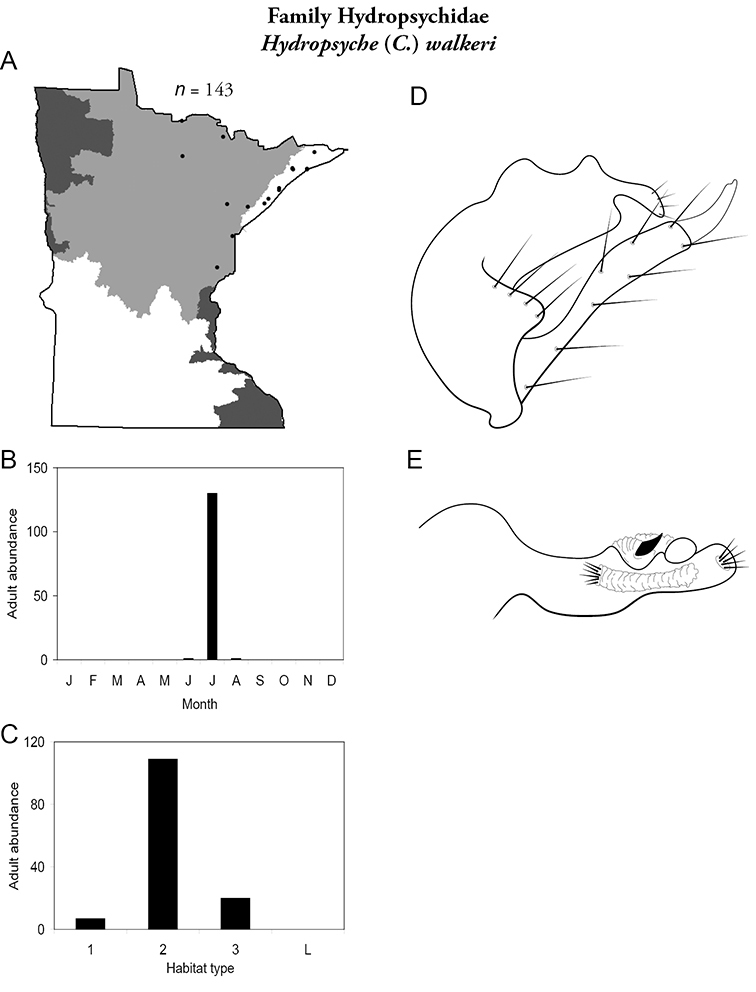
*Hydropsyche walkeri* **A** total specimens collected and all known collecting localities (Figure 4) **B** monthly adult abundance (1980s to present) **C** habitat preference (1980s to present) (Table 1) **D** male genital capsule **E** phallus.

***Hydropsyche (C.) walkeri*** (Figure 60) has been collected from the Lake Superior and Northern Regions, almost exclusively during July. It was most abundant in medium rivers.

Several other Hydropsyche species: *Hydropsyche californica*, *Hydropsyche cuanis*, *Hydropsyche hageni*, *Hydropsyche frisoni*, *Hydropsyche valanis*, and *Hydropsyche ventura*, have been reported from Minnesota based on larval, female or adult specimens of unknown sex (Denning 1943, Lager et al. 1979, Phillipi and Schuster 1987). In all cases, adult males have not been located to confirm the records. Thus, none of these species are included in this manual.

### Genus *Macrostemum*

The genus *Macrostemum* contains a single species in Minnesota. For additional species, see Nimmo (1987). Larval capture nets have very small mesh size; thus, specimens are typically most abundant in large rivers with high loads of fine particulate organic matter (Wiggins 1996). Adults are the largest and most colorful hydropsychids. Forewings are dark brown or black, with a bright yellow or orange pattern. Adults can reach 18 mm in length.

**Figure 61.**
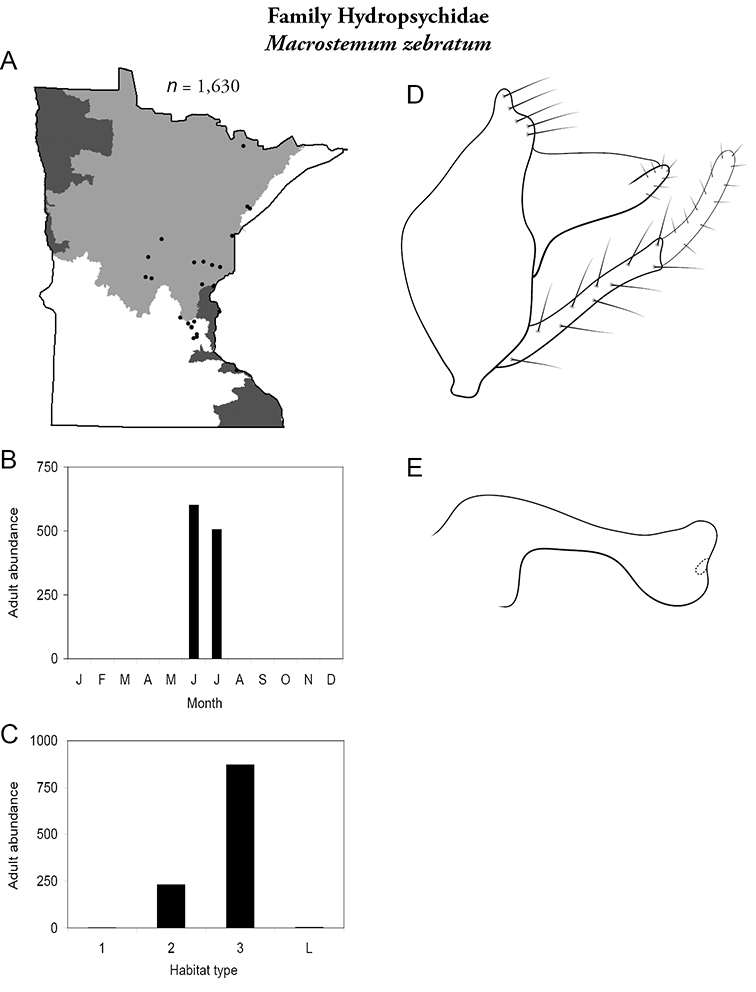
*Macrostemum zebratum* **A** total specimens collected and all known collecting localities (Figure 4) **B** monthly adult abundance (1980s to present) **C** habitat preference (1980s to present) (Table 1) **D** male genital capsule **E** phallus.

***Macrostemum zebratum*** (Figure 61) has been found in the Northern, Southeastern, and Southern Regions. It was most abundant in large rivers, and found during June and July.

### Genus *Potamyia*

The genus *Potamyia* contains a single species in North America and in Minnesota. Larvae are typically found in large rivers, although they can reach a high abundance in smaller streams with high levels of agricultural disturbance. Unlike other hydropsychids, *Potamyia* adults are straw-colored and have antennae >2x the length of the body (Figure 291). These 2 characteristics render both males and females easy to identify without a microscope.

**Figure 62.**
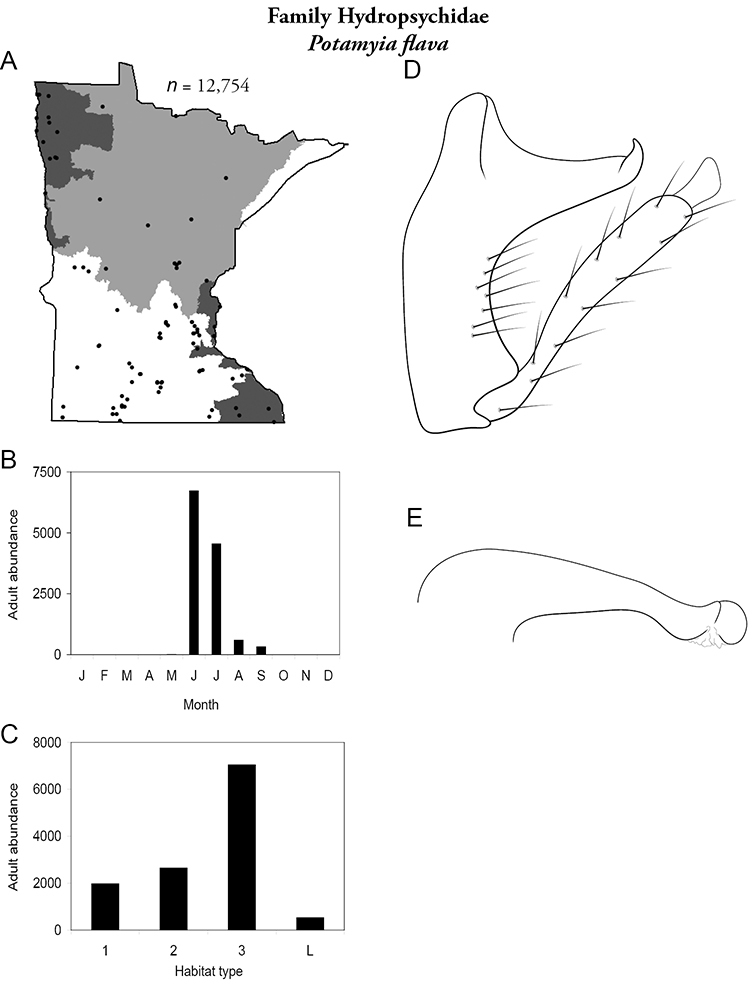
*Potamyia flava* **A** total specimens collected and all known collecting localities (Figure 4) **B** monthly adult abundance (1980s to present) **C** habitat preference (1980s to present) (Table 1) **D** male genital capsule **E** phallus.

***Potamyia flava*** (Figure 62) was found in all regions except the Lake Superior. It was the most abundant species in all sizes of stream in the Southern Region, and was the most abundant species in medium and large rivers of the Northwestern Region (Table 5). Overall, it was the 6th most abundant species in the state (Figure 9). It alsoexhibited a large increase in abundance in small and medium streams with high levels of organic input, and was determined to be an indicator species of such disturbances (Houghton 2004b). Further, the species has greatly increased its overall range throughout disturbed areas of Minnesota (Houghton and Holzenthal 2010). The increase in organic pollution on a landscape level throughout the Northwestern and Southern regions is likely why the species has become so abundant in the last few decades (Houghton 2007).

### Family Hydroptilidae

This family contains 10 genera in Minnesota: *Agraylea*, *Hydroptila*, *Ithytrichia*, *Leucotrichia*, *Mayatrichia*, *Neotrichia*, *Ochrotrichia*, *Orthotrichia*, *Oxyethira*, and *Stactobiella*, and a total of 59 species. It is the most species-rich family in the state. It also contains 2 of the most species-rich genera in the state: *Hydroptila* and *Oxyethira*. Members are often referred to as the “microcaddisflies” due to their small size. For additional species of all genera, see Blickle (1979).

Larvae are found in nearly any type of freshwater habitat, but are usually more abundant in streams. They are unique among caddisflies in their hypermetamorphic life cycle. That is, larvae do not construct cases for the first 4 instars, and are instead free-living. The terminal instar constructs a purse-like case of mainly silk, with occasional algae or small sand grains (Wiggins 1996). Most genera exhibit only minor differences in case morphology. Larvae are most often collected among mats of algae, which is a primary food source for many species. In general, however, larvae are difficult to collect due to their small size.

Most adults range 2–3 mm in length. Females of *Agraylea* may reach 4–5 mm. Wings are usually grey, pointed at their apices, and covered with dense setae. Most genera are macroscopically indistinguishable from each other. Adults can be very abundant in light traps; frequently, more than a dozen species were found together at a single site. Females are typically considerably more abundant than males. Unfortunately, females are not readily identifiable. Thus, species are probably more widespread and abundant than they appear. In fact, due to their small size, even males can be difficult to identify. Specimens must be cleared to have an adequate view of their genitalic structure. Further, the phallus of many species needs to be gently extruded from the genital capsule to obtain a clear view of its structure.

### Genus *Agraylea*

The genus *Agraylea* contains a single species in Minnesota. Larvae inhabit lakes and slow-moving areas of streams. They are typically found in the beds of submerged plants upon which they feed (Wiggins 1996).

**Figure 63.**
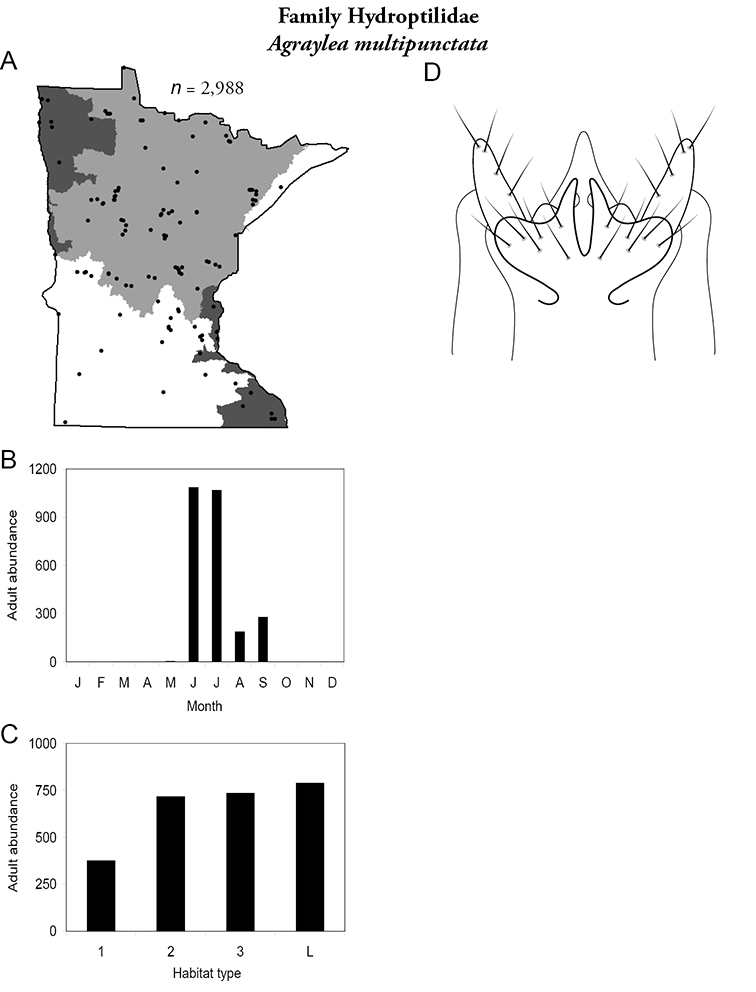
*Agraylea multipunctata* **A** total specimens collected and all known collecting localities (Figure 4) **B** monthly adult abundance (1980s to present) **C** habitat preference (1980s to present) (Table 1) **D** male genital capsule (ventral view).

***Agraylea multipunctata*** (Figure 63) is the largest of the Minnesota hydroptilids, occasionally reaching 5 mm in length. Wings have a distinctive grey and dark brown banding pattern, allowing for easy identification of both males and females with practice (Figure 290). The species is the 8th most widespread caddisfly in Minnesota (Figure 8), found throughout all regions. It was collected from all habitat types. Adults were abundant in June and July, and also present in August and September.

### Genus *Hydroptila*

The genus *Hydroptila* contains 26 species in Minnesota. It is the most species-rich genus in the state. Several of the species, however, have not been collected since the 1960s or earlier. All of these species are known historically from a single or few specimens. Thus, it is difficult to know if they have been extirpated from the state or are just rare and difficult to collect. Several other species are known recently from only a few specimens. Males are rare in collections relative to females, which are not identifiable. Thus, some of the rare species may be more widespread than their known distributions suggest.

Larvae consume the contents of algal cells (Wiggins 1996), and can sometimes be abundant in algal mats. Adults—females in particular—can be extremely abundant in light traps. Adults are macroscopically indistinguishable from other hydroptilid genera. To properly identify males of many species, the phallus should be gently extruded from the cleared genital capsule. This preparation is especially important when separating *Hydroptila amoena*, *Hydroptila ampoda*, *Hydroptila metoeca*, and *Hydroptila hamata*, all of which have a similar genital capsule.

**Figure 64.**
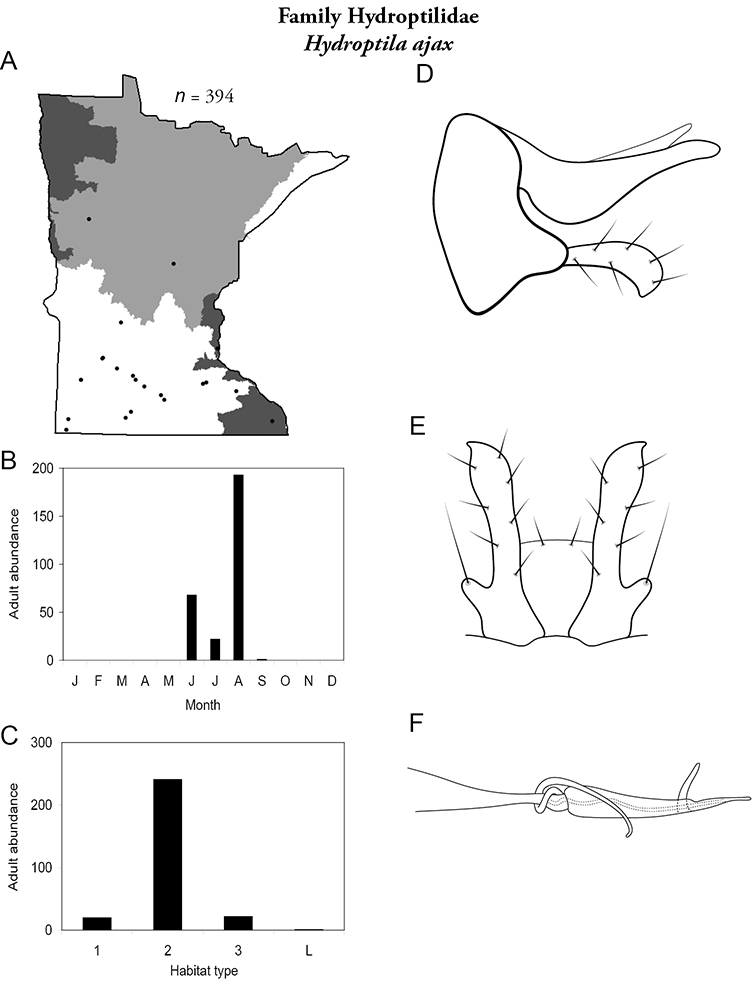
*Hydroptila ajax* **A** total specimens collected and all known collecting localities (Figure 4) **B** monthly adult abundance (1980s to present) **C** habitat preference (1980s to present) (Table 1) **D** male genital capsule **E** male genital capsule (ventral view) **F** phallus.

***Hydroptila ajax*** (Figure 64) is known mainly from the Southern Region and sporadically elsewhere. It has been collected mostly from medium rivers and is most abundant in August. Some adults were collected in June, July, and September.

**Figure 65.**
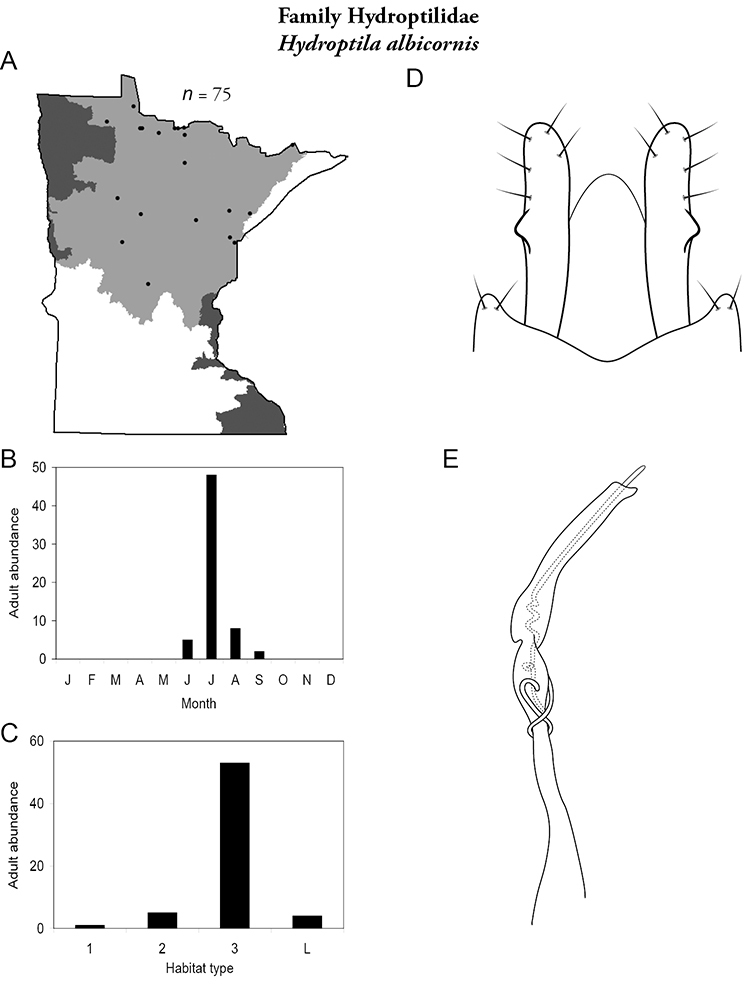
*Hydroptila albicornis* **A** total specimens collected and all known collecting localities (Figure 4) **B** monthly adult abundance (1980s to present) **C** habitat preference (1980s to present) (Table 1) **D** male genital capsule **E** phallus.

***Hydroptila albicornis*** (Figure 65) is known only from the Northern Region. It was most frequently collected from large rivers during July.

**Figure 66.**
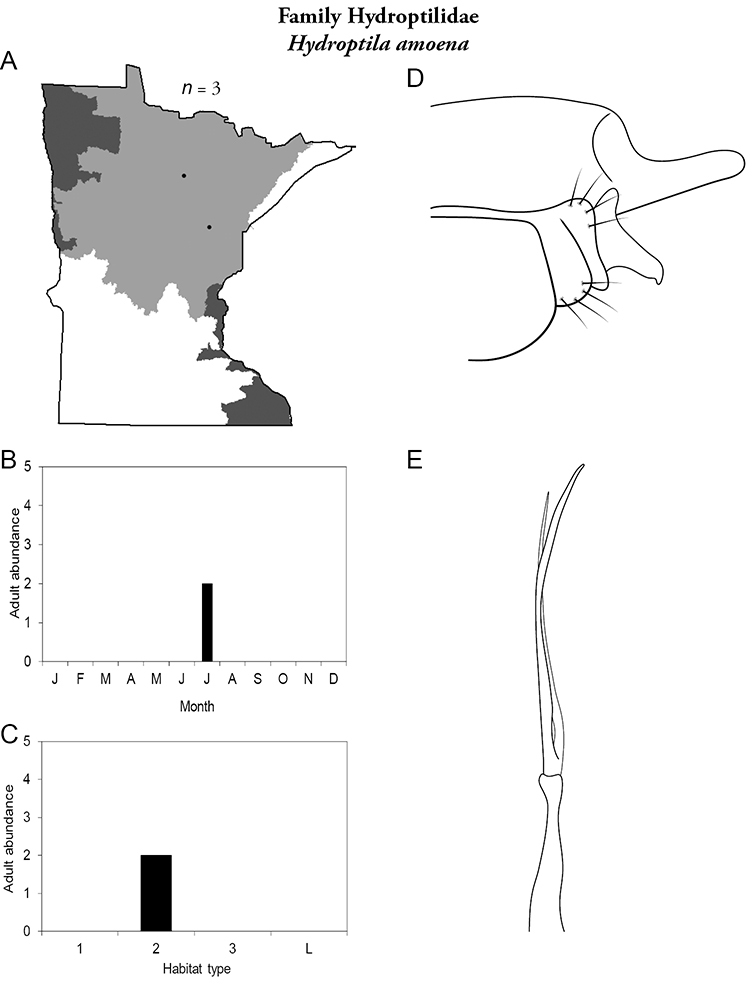
*Hydroptila amoena* **A** total specimens collected and all known collecting localities (Figure 4) **B** monthly adult abundance (1980s to present) **C** habitat preference (1980s to present) (Table 1) **D** male genital capsule **E** phallus.

***Hydroptila amoena*** (Figure 66) is known only from a couple of specimens collected from medium rivers of the Northern Region in July.

**Figure 67.**
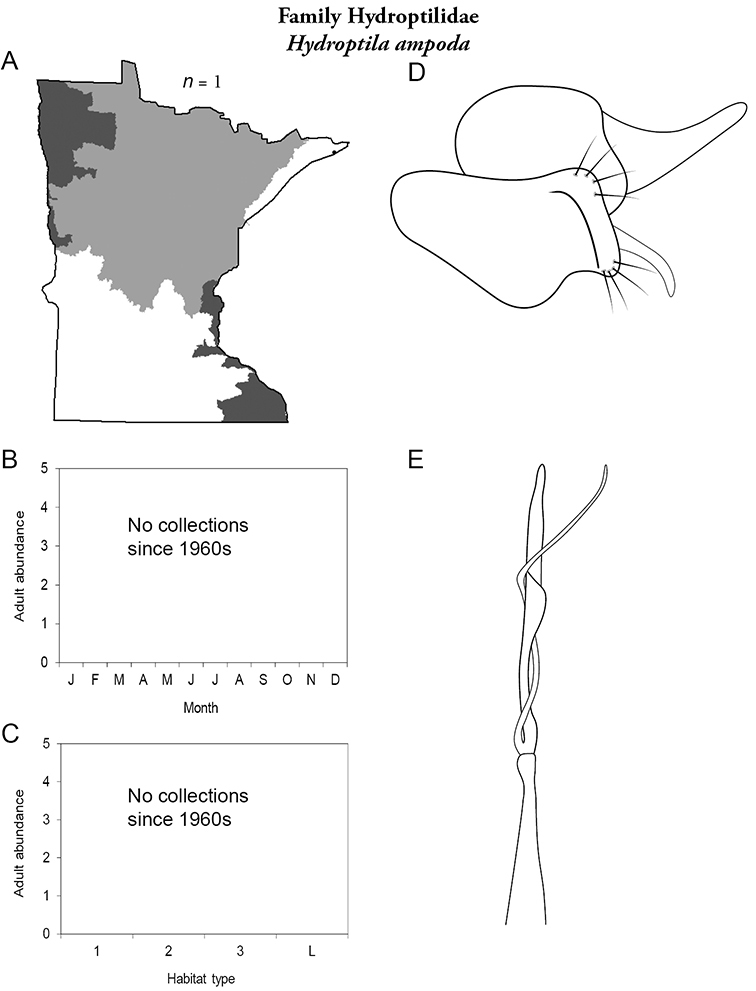
*Hydroptila ampoda* **A** total specimens collected and all known collecting localities (Figure 4) **B** monthly adult abundance (1980s to present) **C** habitat preference (1980s to present) (Table 1) **D** male genital capsule **E** phallus.

***Hydroptila ampoda*** (Figure 67) has only been collected from the city of Hovland in the 1960s. It has not been collected since.

**Figure 68.**
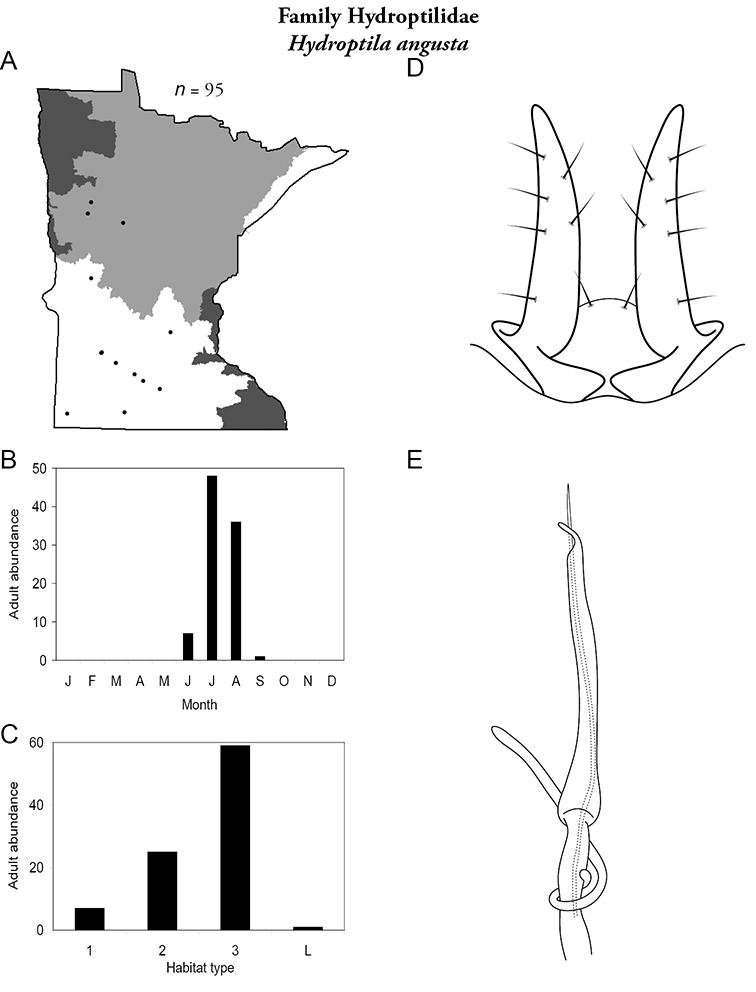
*Hydroptila angusta* **A** total specimens collected and all known collecting localities (Figure 4) **B** monthly adult abundance (1980s to present) **C** habitat preference (1980s to present) (Table 1) **D** male genital capsule **E** phallus.

***Hydroptila angusta*** (Figure 68) has been collected from the Northern and Southern Regions, predominantly from medium and, especially, large rivers. Adults were present from June to September and abundant during July and August.

**Figure 69.**
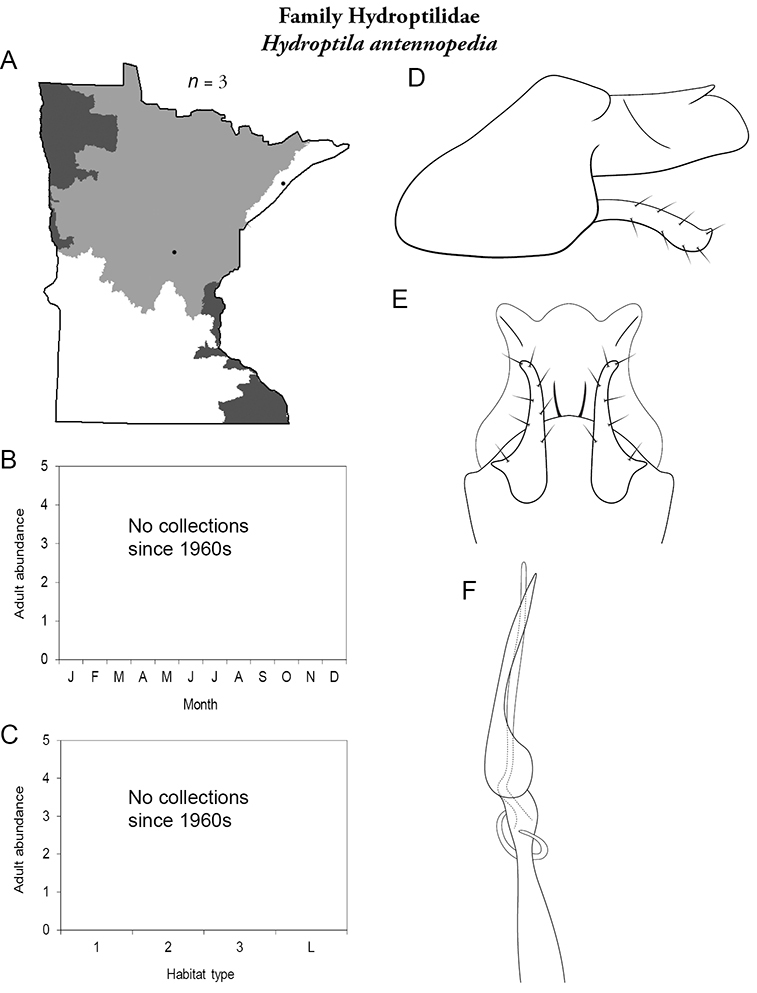
*Hydroptila antennopedia* **A** total specimens collected and all known collecting localities (Figure 4) **B** monthly adult abundance (1980s to present) **C** habitat preference (1980s to present) (Table 1) **D** male genital capsule **E** male genital capsule (ventral view) **F** phallus.

***Hydroptila antennopedia*** (Figure 69) has not been collected since the 1960s. It was found historically from sites in the Lake Superior and Northern Regions.

**Figure 70.**
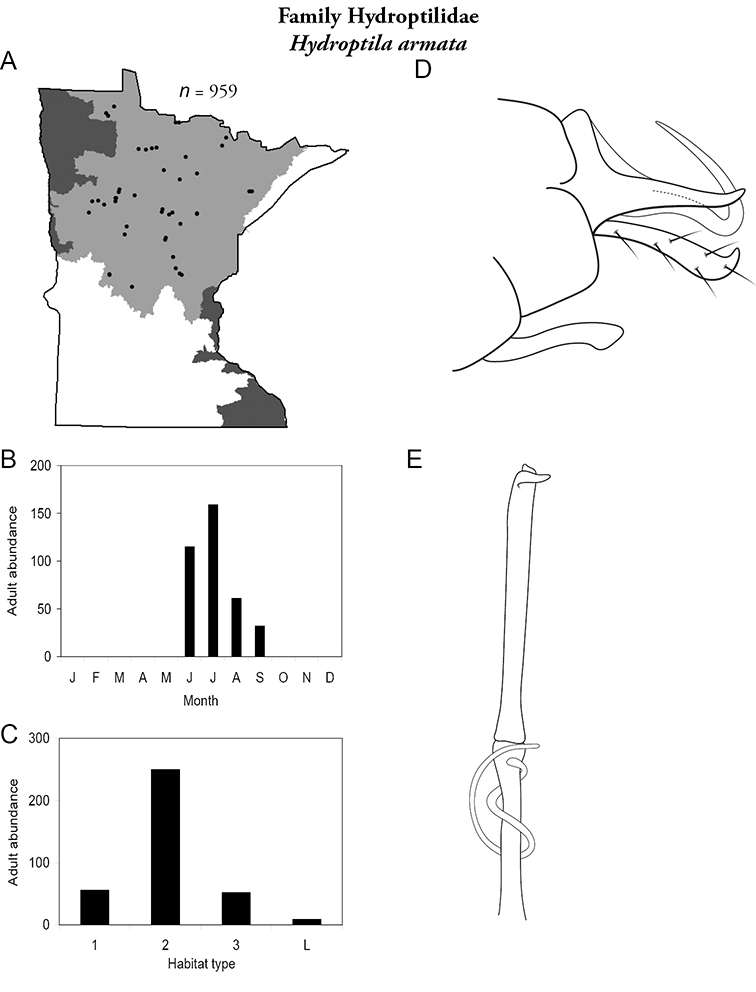
*Hydroptila armata* **A** total specimens collected and all known collecting localities (Figure 4) **B** monthly adult abundance (1980s to present) **C** habitat preference (1980s to present) (Table 1) **D** male genital capsule **E** phallus.

***Hydroptila armata*** (Figure 70) is known exclusively from the Northern Region, from June through September. It was most abundant in medium rivers.

**Figure 71.**
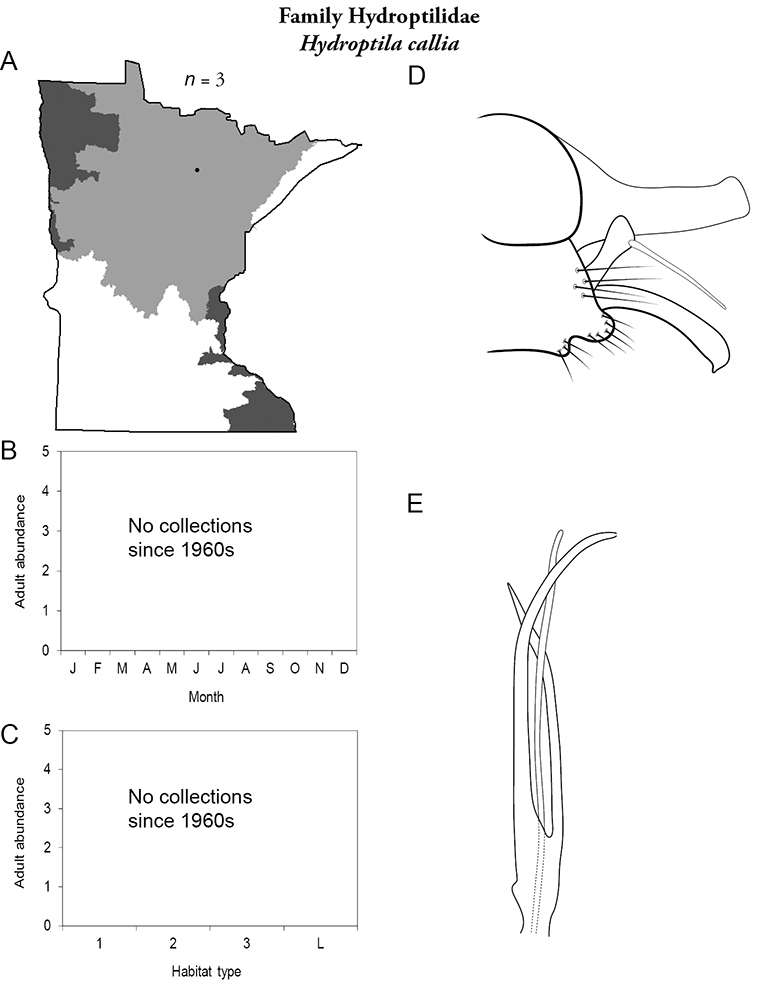
*Hydroptila calia* **A** total specimens collected and all known collecting localities (Figure 4) **B** monthly adult abundance (1980s to present) **C** habitat preference (1980s to present) (Table 1) **D** male genital capsule **E** phallus.

***Hydroptila callia*** (Figure 71) is known only from a single specimen collected from Link (Lynx) Lake, Itasca County, in the Northern Region during July 1966. It has not been collected since.

**Figure 72.**
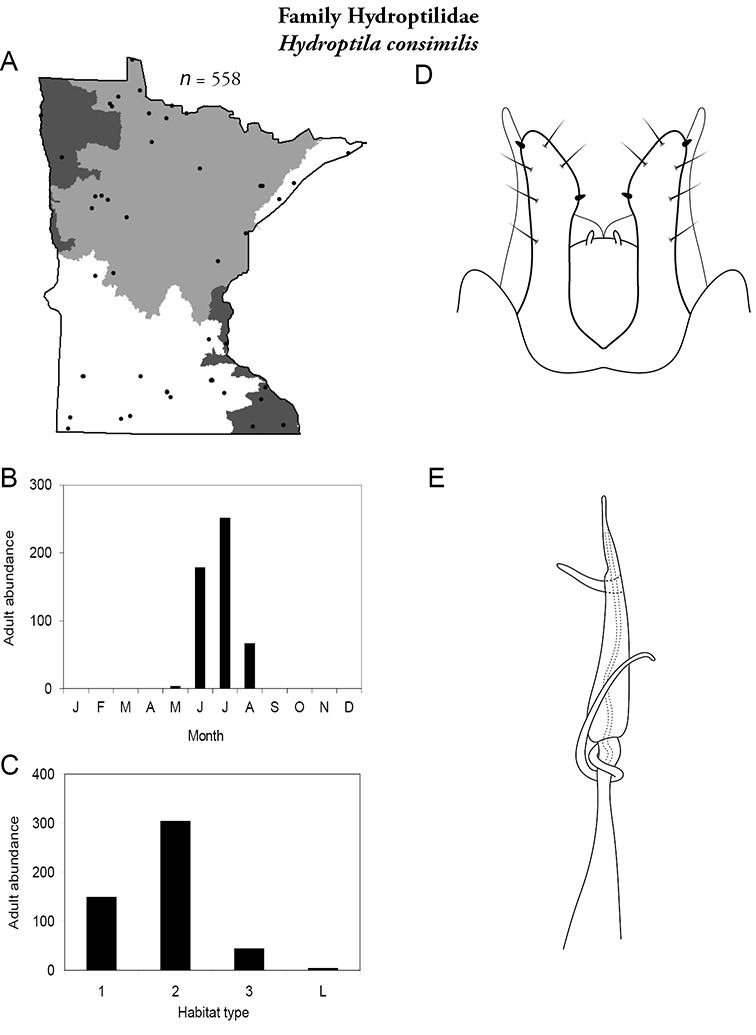
*Hydroptila consimils* **A** total specimens collected and all known collecting localities (Figure 4) **B** monthly adult abundance (1980s to present) **C** habitat preference (1980s to present) (Table 1) **D** male genital capsule **E** phallus.

***Hydroptila consimilis*** (Figure 72) was found in all regions, mostly in small and medium streams. Adults were collected mainly June through August.

**Figure 73.**
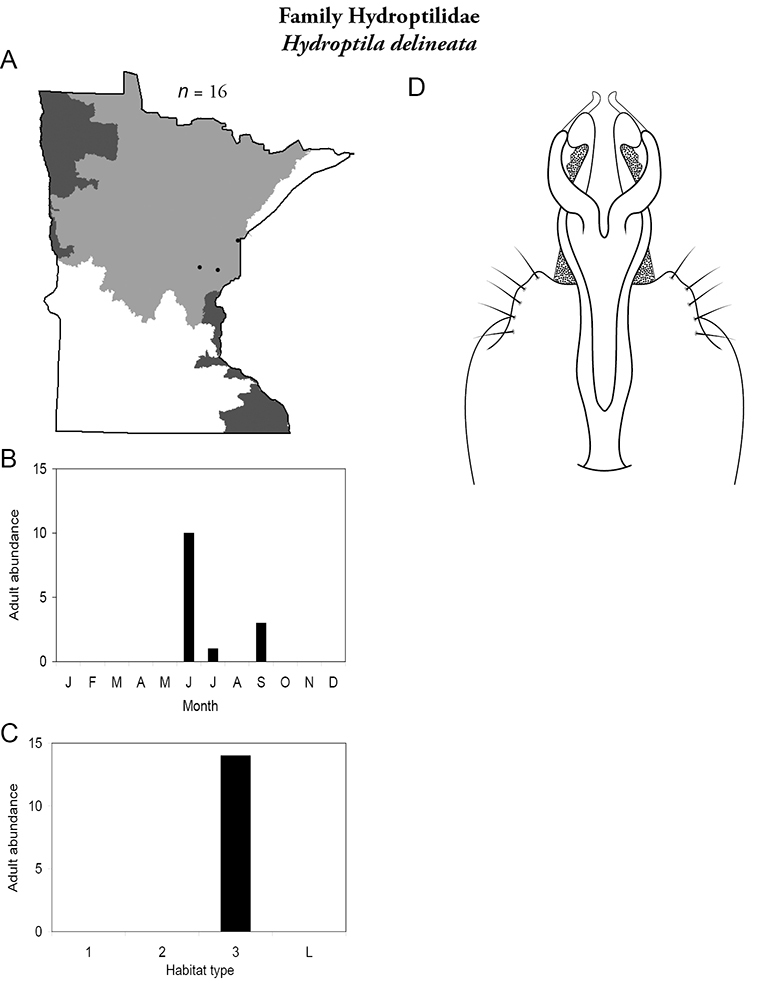
*Hydroptila delineata* **A** total specimens collected and all known collecting localities (Figure 4) **B** monthly adult abundance (1980s to present) **C** habitat preference (1980s to present) (Table 1) **D** male genital capsule (ventral view).

***Hydroptila delineata*** (Figure 73) has been collected only from large rivers within a small area in the southeastern portion of the Northern Region. Adults were present in June, July, and September, but not abundant.

**Figure 74.**
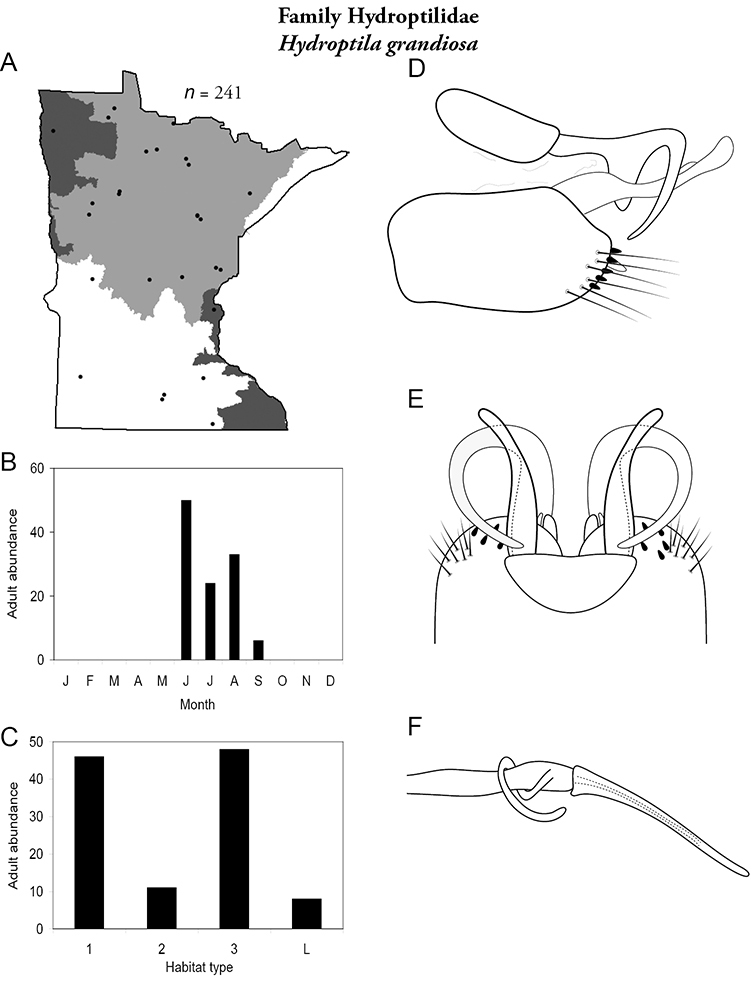
*Hydroptila grandiosa* **A** total specimens collected and all known collecting localities (Figure 4) **B** monthly adult abundance (1980s to present) **C** habitat preference (1980s to present) (Table 1) **D** male genital capsule **E** male genital capsule (ventral view) **F** phallus.

***Hydroptila grandiosa*** (Figure 74) is primarily known from the Northern and Southern Regions. Interestingly, it was most abundant in both small streams and large rivers, but not medium rivers. Adults were abundant from June through August, and present into September.

**Figure 75.**
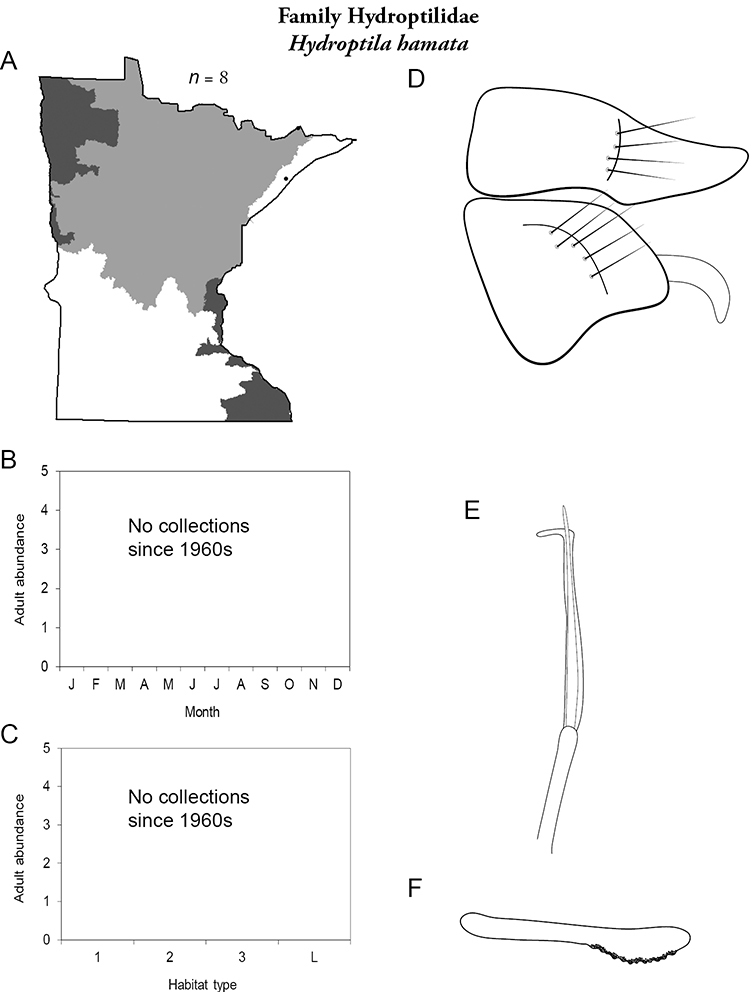
*Hydroptila hamata* **A** total specimens collected and all known collecting localities (Figure 4) **B** monthly adult abundance (1980s to present) **C** habitat preference (1980s to present) (Table 1) **D** male genital capsule **E** phallus **F** styli of 7th abdominal sternum.

***Hydroptila hamata*** (Figure 75) is known only from a couple of collections in the Lake Superior and Northern Regions. It has not been collected since the 1960s.

**Figure 76.**
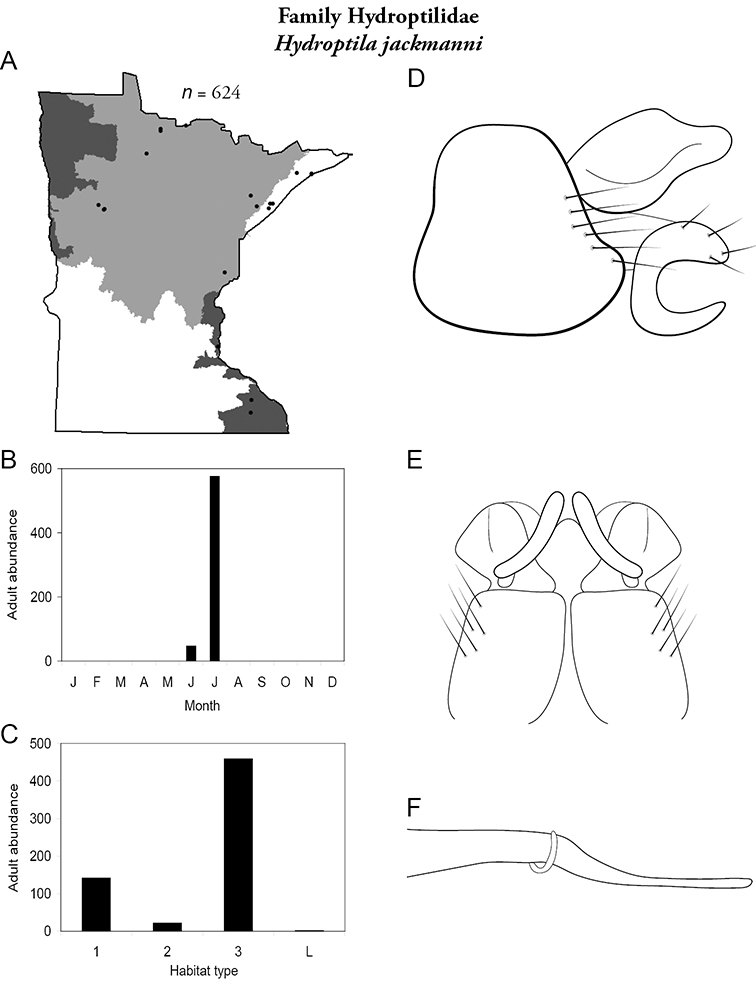
*Hydroptila jackmanni* **A** total specimens collected and all known collecting localities (Figure 4) **B** monthly adult abundance (1980s to present) **C** habitat preference (1980s to present) (Table 1) **D** male genital capsule **E** male genital capsule (ventral view) **F** phallus.

***Hydroptila jackmanni*** (Figure 76) has been collected from the Lake Superior, Northern, and Southeastern Regions. It was most abundant in large rivers and collected almost exclusively during July.

**Figure 77.**
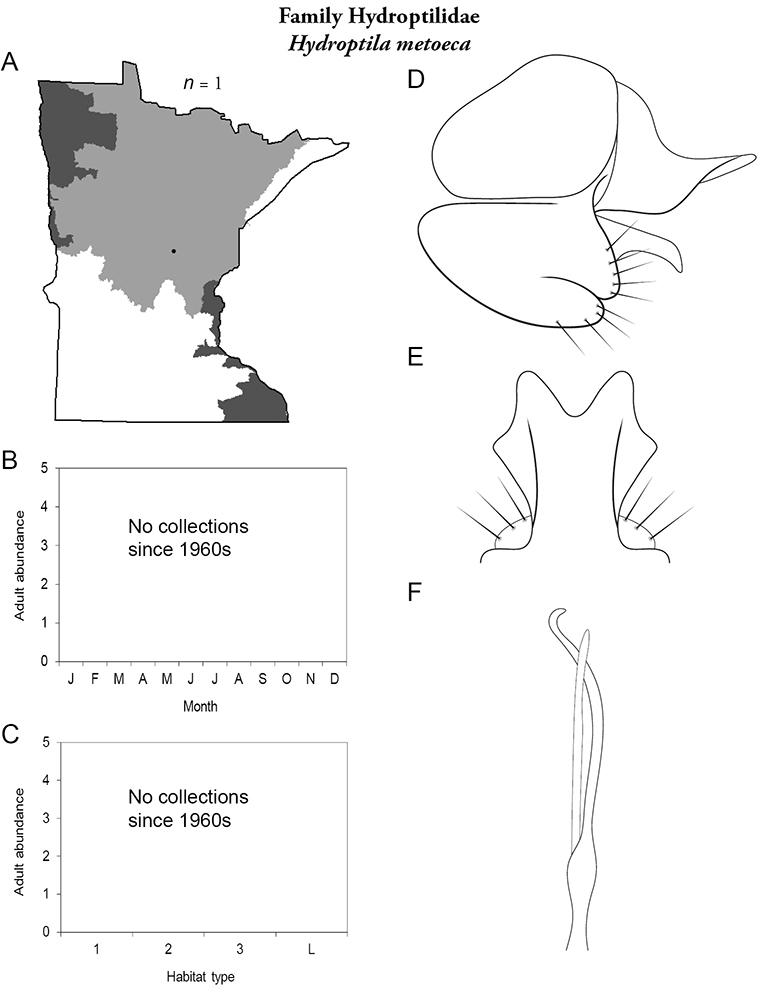
*Hydroptila metoeca* **A** total specimens collected and all known collecting localities (Figure 4) **B** monthly adult abundance (1980s to present) **C** habitat preference (1980s to present) (Table 1) **D** male genital capsule **E** male genital capsule (ventral view) **F** phallus.

***Hydroptila metoeca*** (Figure 77) is known only from a single specimen collected from the city of Garrison, Crow Wing County, in the Northern Region during August 1965. It has not been collected since.

**Figure 78.**
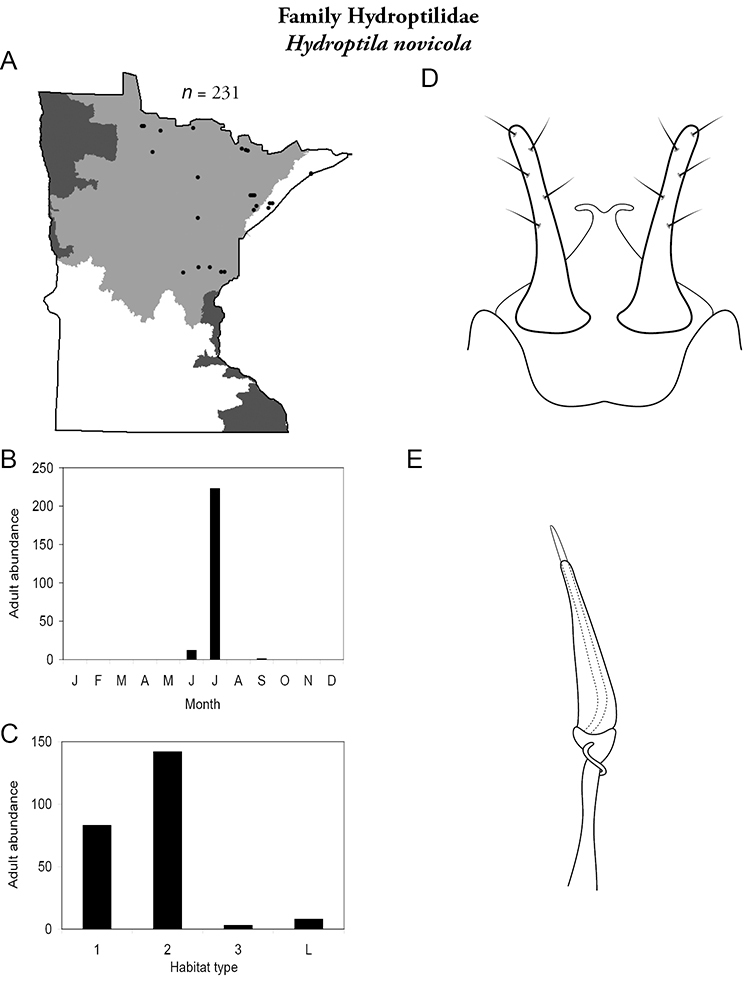
*Hydroptila novicola* **A** total specimens collected and all known collecting localities (Figure 4) **B** monthly adult abundance (1980s to present) **C** habitat preference (1980s to present) (Table 1) **D** male genital capsule (ventral view) **E** phallus.

***Hydroptila novicola*** (Figure 78) is known from the Lake Superior and Northern Regions. It was most abundant in small and medium streams during July. Interestingly, prior to 1999 *Hydroptila novicola* was known only from a single specimen and listed as “Special Concern” by the Minnesota Department of Natural Resources (MNDNR 2012). From 1999 to 2001, however, many additional populations were discovered, prompting its proposed de-listing (Houghton and Holzenthal 2003, MNDNR 2012). The species is readily attracted to light traps, so it is not clear why it appeared so scarce until fairly recently.

**Figure 79.**
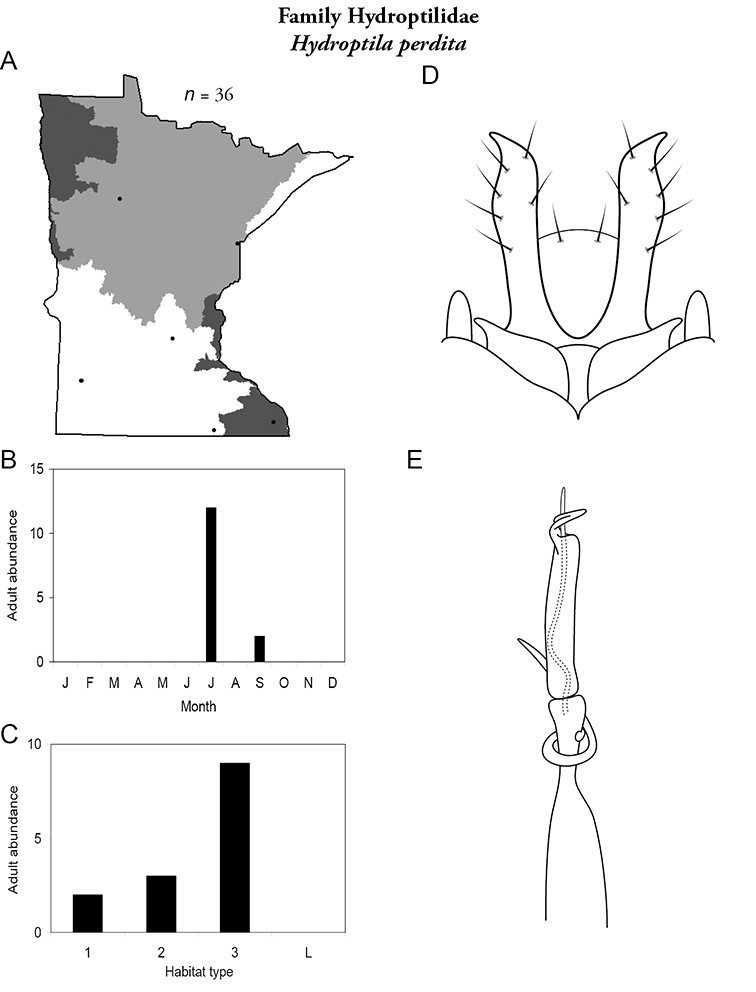
*Hydroptila perdita* **A** total specimens collected and all known collecting localities (Figure 4) **B** monthly adult abundance (1980s to present) **C** habitat preference (1980s to present) (Table 1) **D** male genital capsule (ventral view) **E** phallus.

***Hydroptila perdita*** (Figure 79) has been collected sporadically throughout the state, mostly in July. It was most abundant in large rivers.

**Figure 80.**
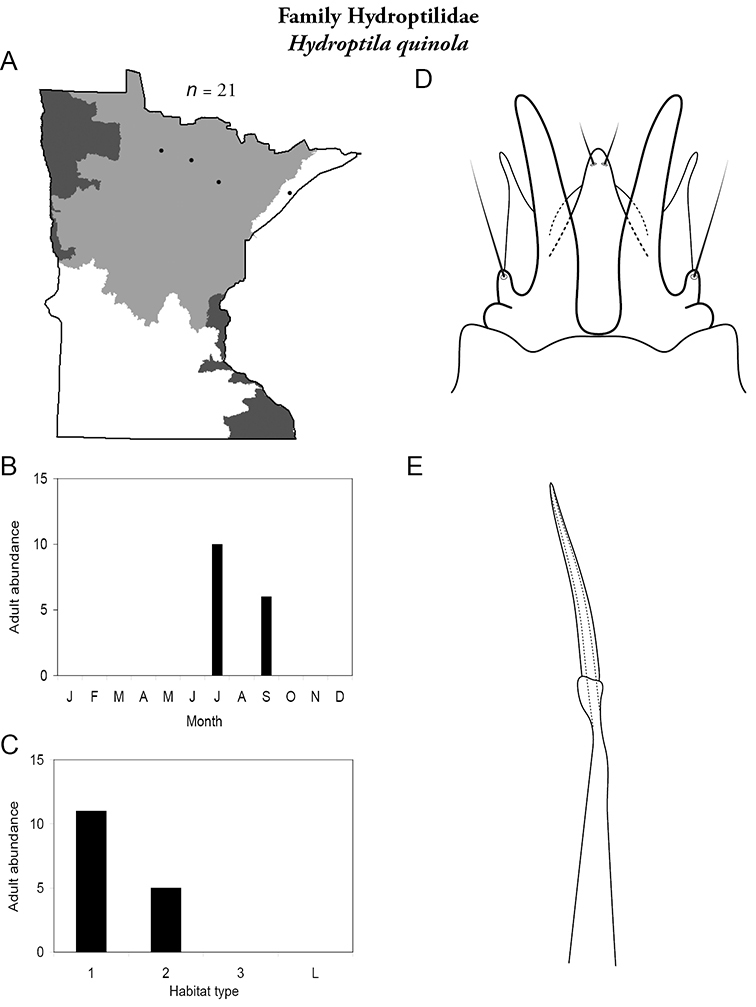
*Hydroptila quinola* **A** total specimens collected and all known collecting localities (Figure 4) **B** monthly adult abundance (1980s to present) **C** habitat preference (1980s to present) (Table 1) **D** male genital capsule (ventral view) **E** phallus.

***Hydroptila quinola*** (Figure 80) is known only from a few small and medium streams in the Lake Superior and Northern Regions. Adults were present in July and September, which probably reflects a lack of collecting in August.

**Figure 81.**
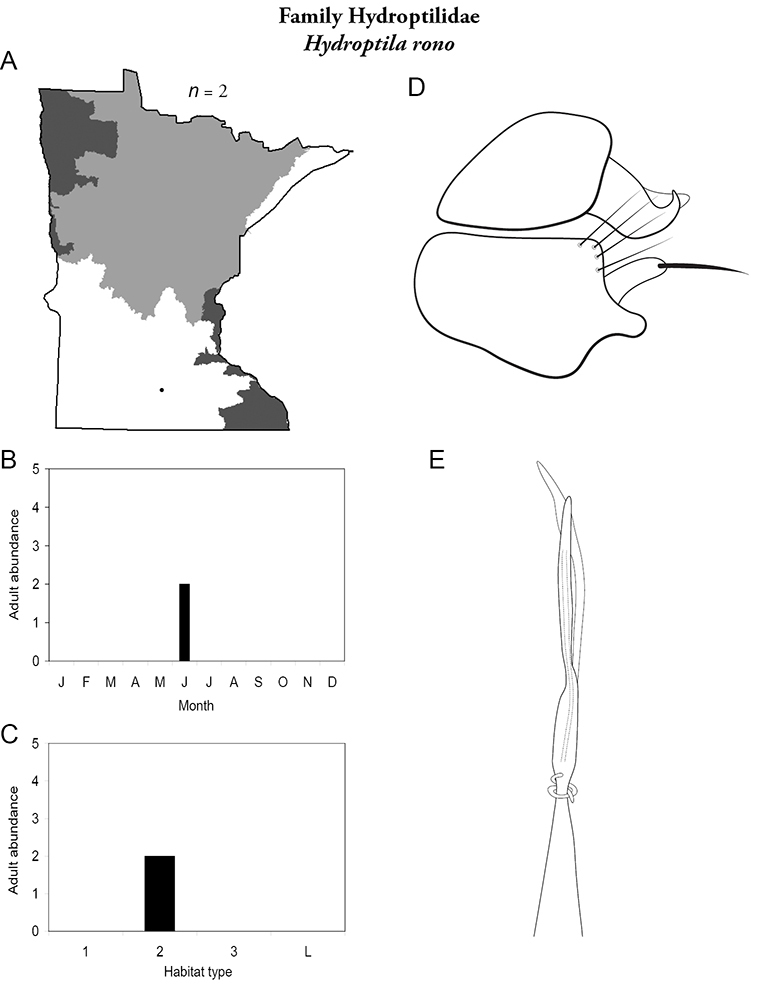
*Hydroptila rono* **A** total specimens collected and all known collecting localities (Figure 4) **B** monthly adult abundance (1980s to present) **C** habitat preference (1980s to present) (Table 1) **D** male genital capsule **E** phallus.

***Hydroptila rono*** (Figure 81) is known only from a single collection from a large waterfall of Minneopa Creek, Minneopa State Park, in the Southern Region during June 2000. The species is typically found in western montane streams, but has also been collected from high gradient rivers in Pennsylvania and Quebec (Morse 2011). Minneopa Creek is, likewise, a high gradient stream, fairly atypical of southern Minnesota. Due to the extreme rarity of the species, and the high degree of habitat disturbance in southern Minnesota, *Hydroptila rono* has been proposed as “Threatened” by the Minnesota Department of Natural Resources (MNDNR 2012).

**Figure 82.**
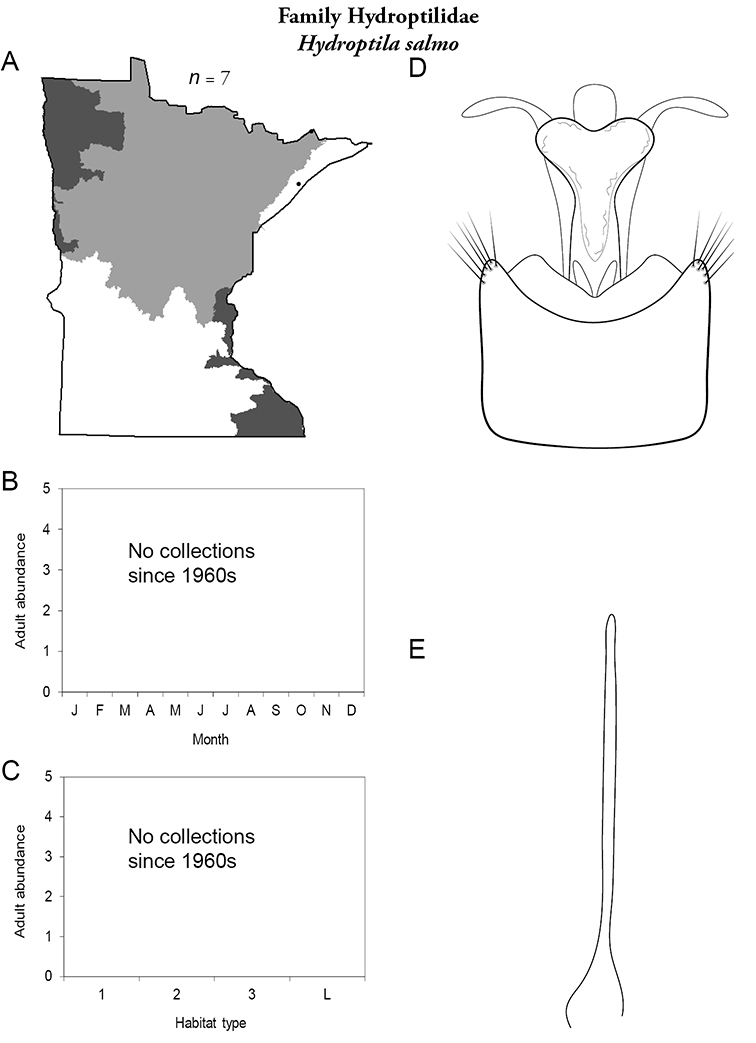
*Hydroptila salmo* **A** total specimens collected and all known collecting localities (Figure 4) **B** monthly adult abundance (1980s to present) **C** habitat preference (1980s to present) (Table 1) **D** male genital capsule (ventral view) **E** phallus.

***Hydroptila salmo*** (Figure 82) is known only from a couple of collections from the Lake Superior and Northern Regions. It has not been collected since the 1960s.

**Figure 83.**
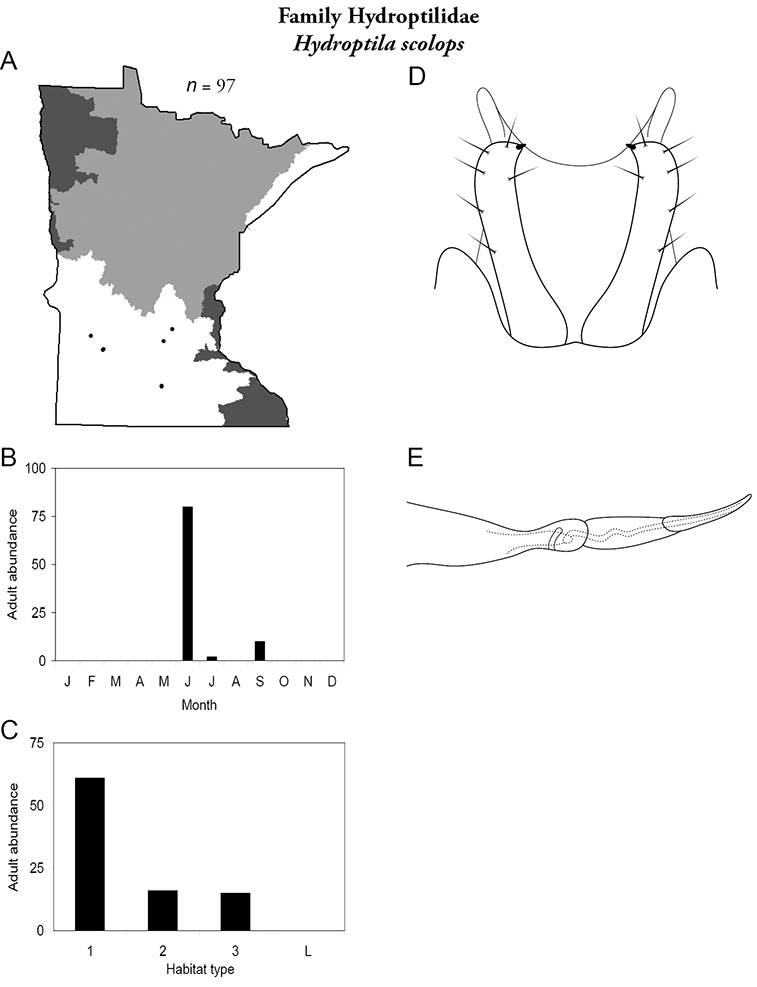
*Hydroptila scolops* **A** total specimens collected and all known collecting localities (Figure 4) **B** monthly adult abundance (1980s to present) **C** habitat preference (1980s to present) (Table 1) **D** male genital capsule (ventral view) **E** phallus.

***Hydroptila scolops*** (Figure 83) has been collected only from the Southern Region. It was most abundant in small streams, and also found in medium and large rivers. Adults were most abundant in June, and also present July though September.

**Figure 84.**
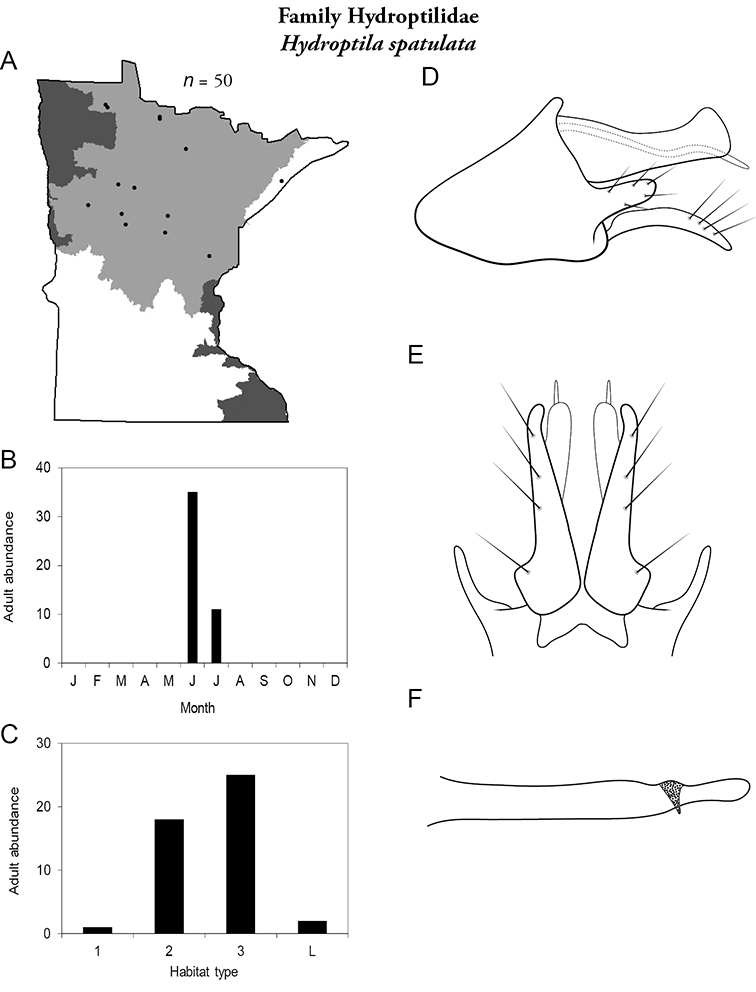
*Hydroptila spatulata* **A** total specimens collected and all known collecting localities (Figure 4) **B** monthly adult abundance (1980s to present) **C** habitat preference (1980s to present) (Table 1) **D** male genital capsule (ventral view) **E** male genital capsule (ventral view) **F** phallus.

***Hydroptila spatulata*** (Figure 84) is known from the Lake Superior and, especially, the Northern Regions. It was collected predominantly from medium and large rivers. Adults were most abundant in June, with some presence in July.

**Figure 85.**
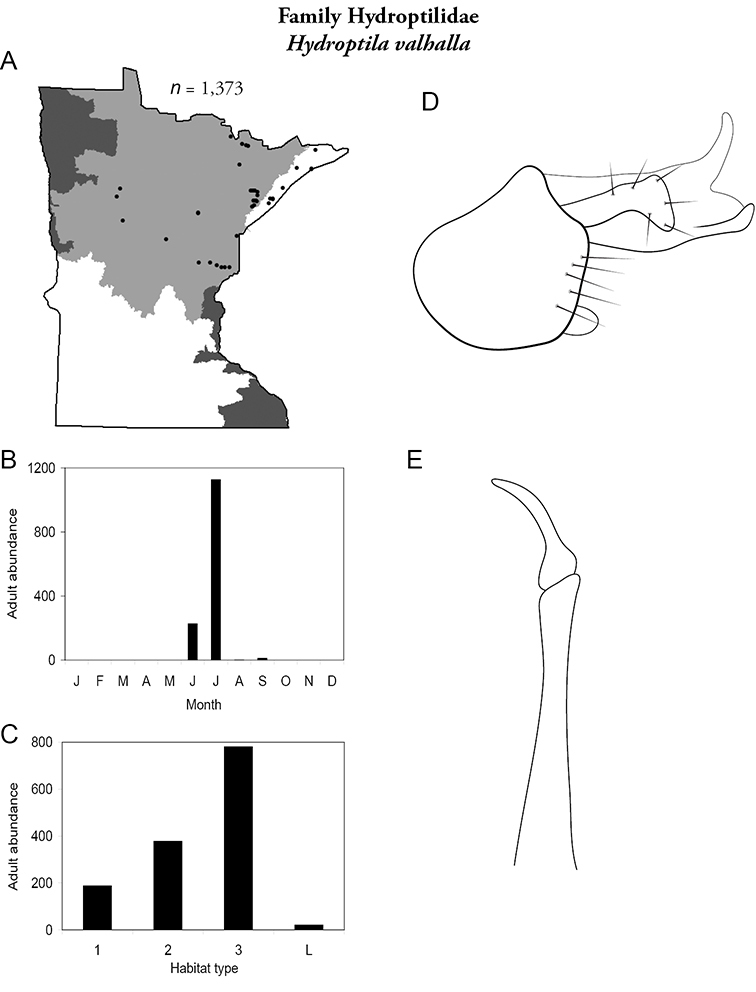
*Hydroptila valhalla* **A** total specimens collected and all known collecting localities (Figure 4) **B** monthly adult abundance (1980s to present) **C** habitat preference (1980s to present) (Table 1) **D** male genital capsule **E** phallus.

***Hydroptila valhalla*** (Figure 85) was found in the Lake Superior and Northern Regions, and was locally abundant at several collecting sites. It was found primarily in medium and, especially, large rivers. Adults were present in June and September, but most abundant in July.

**Figure 86.**
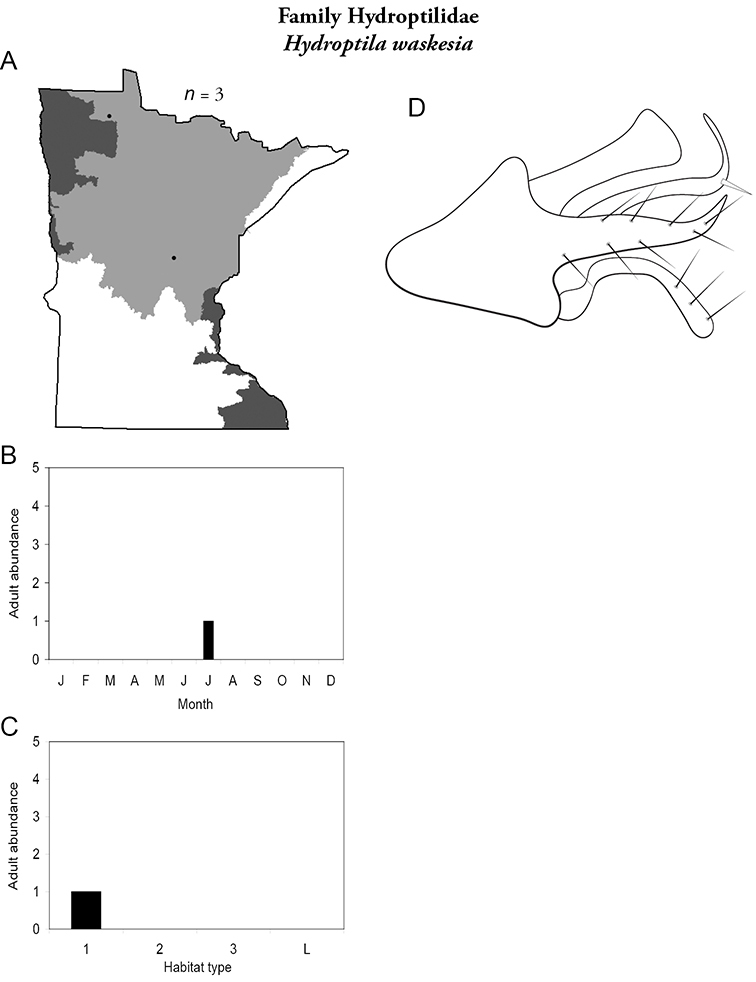
*Hydroptila waskesia* **A** total specimens collected and all known collecting localities (Figure 4) **B** monthly adult abundance (1980s to present) **C** habitat preference (1980s to present) (Table 1) **D** male genital capsule.

***Hydroptila waskesia*** (Figure 86) is known only from the City of Garrison, Crow Wing County, collected in 1964 and 1965, and from Hansen Creek, Roseau County, collected in July of 2000. Both localities are in the Northern Region. Due to the rarity of *Hydroptila waskesia* and the lack of undisturbed habitats in its range (Houghton 2007), the species has been proposed as “Endangered” by the Minnesota Department of Natural Resources (MNDNR 2012).

**Figure 87.**
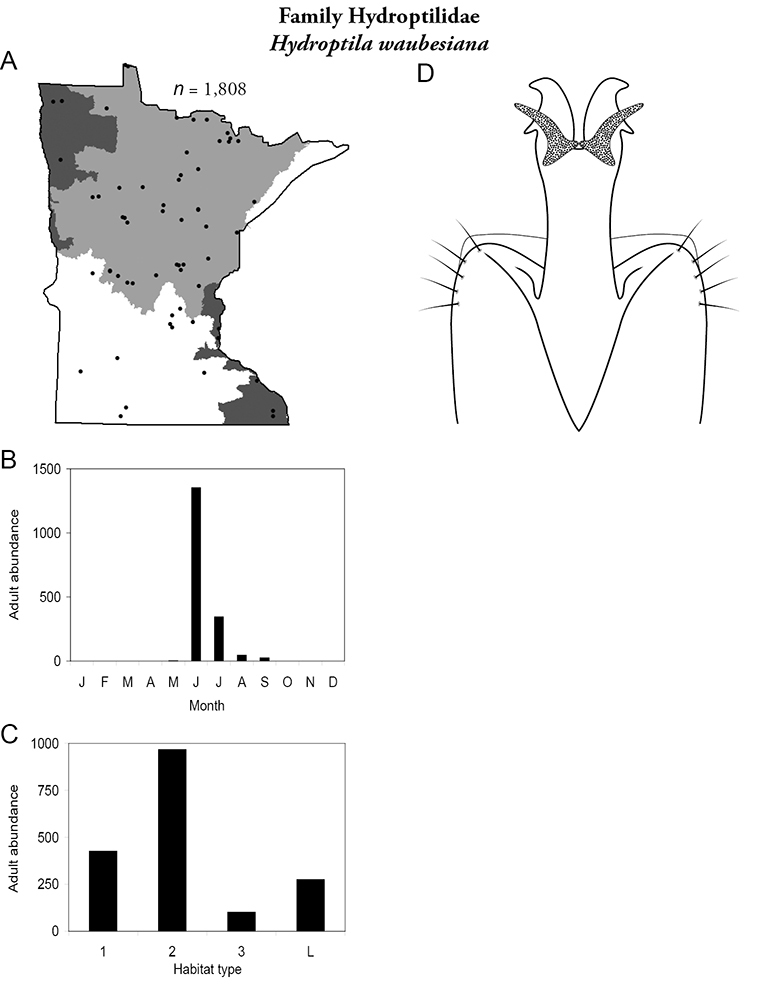
*Hydroptila waubesiana* **A** total specimens collected and all known collecting localities (Figure 4) **B** monthly adult abundance (1980s to present) **C** habitat preference (1980s to present) (Table 1) **D** male genital capsule (ventral view).

***Hydroptila waubesiana*** (Figure 87) has been collected from all regions except the Lake Superior. It was found in all habitat types, and most abundant in medium streams. Adults were present from June to September, reaching highest abundance in June.

**Figure 88.**
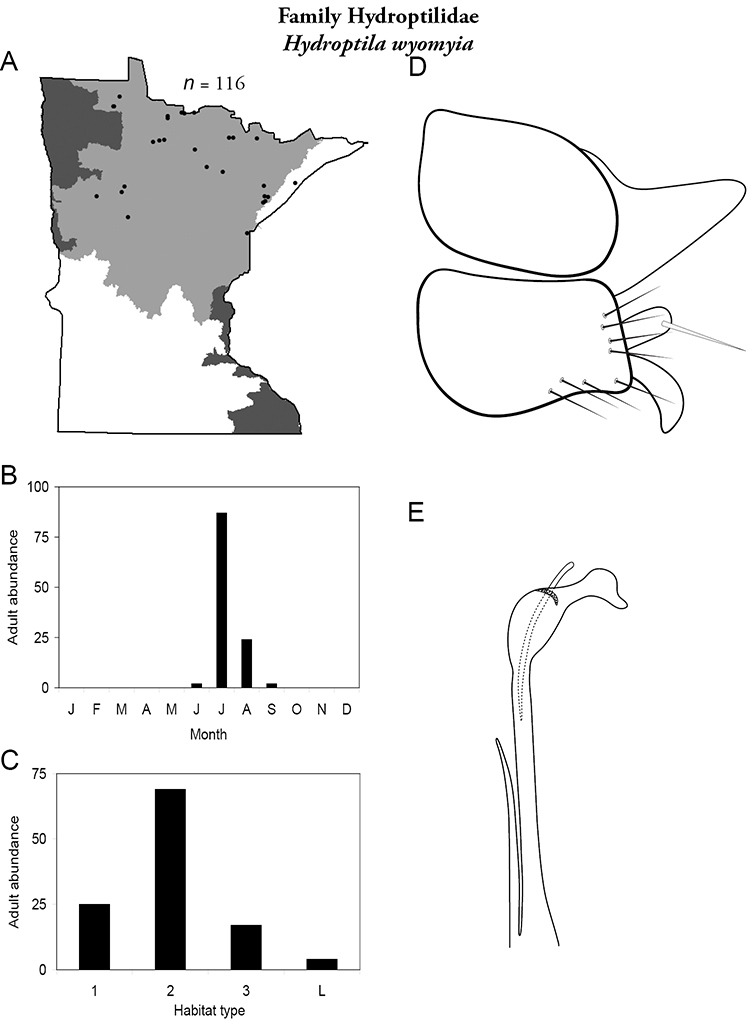
*Hydroptila wyomyia* **A** total specimens collected and all known collecting localities (Figure 4) **B** monthly adult abundance (1980s to present) **C** habitat preference (1980s to present) (Table 1) **D** male genital capsule **E** phallus.

***Hydroptila wyomyia*** (Figure 88) is known from the Lake Superior and, especially, the Northern Regions where it was found primarily in streams, particularly medium rivers. It reached highest adult abundance in July and was also present June, August, and September.

**Figure 89.**
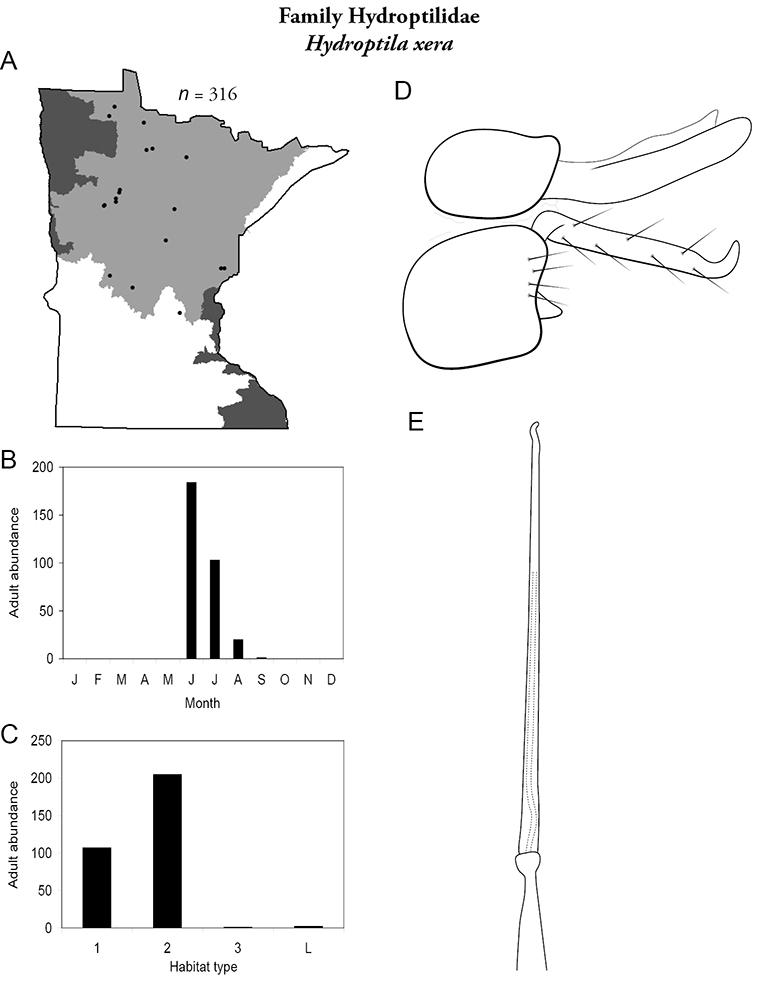
*Hydroptila xera* **A** total specimens collected and all known collecting localities (Figure 4) **B** monthly adult abundance (1980s to present) **C** habitat preference (1980s to present) (Table 1) **D** male genital capsule **E** phallus.

***Hydroptila xera*** (Figure 89) has been collected from and near the Northern Region, predominantly from small and medium streams. Adults were most abundant in June and July, and present in August and September.

Another *Hydroptila* species, *Hydroptila virgata*, was reported from Minnesota based on a female specimen (Etnier 1965). The identity of this specimen is not clear. Without a male record to confirm the species’ presence in Minnesota, *Hydroptila virgata* is not included in this manual. Another *Hydroptila* species, *Hydroptila strepha*, was reported from Minnesota based on a series of male specimens (Etnier 1965). All of these specimens have been re-identified as *Hydroptila antennopedia*. Thus, *Hydroptila strepha* is not included in this manual.

### Genus *Ithytrichia*

The genus *Ithytrichia* contains a single species in Minnesota. Larvae are typically found on the surface of medium to large rocks where they consume algae and diatoms (Wiggins 1996). Adults are macroscopically indistinguishable from other hydroptilid genera.

**Figure 90.**
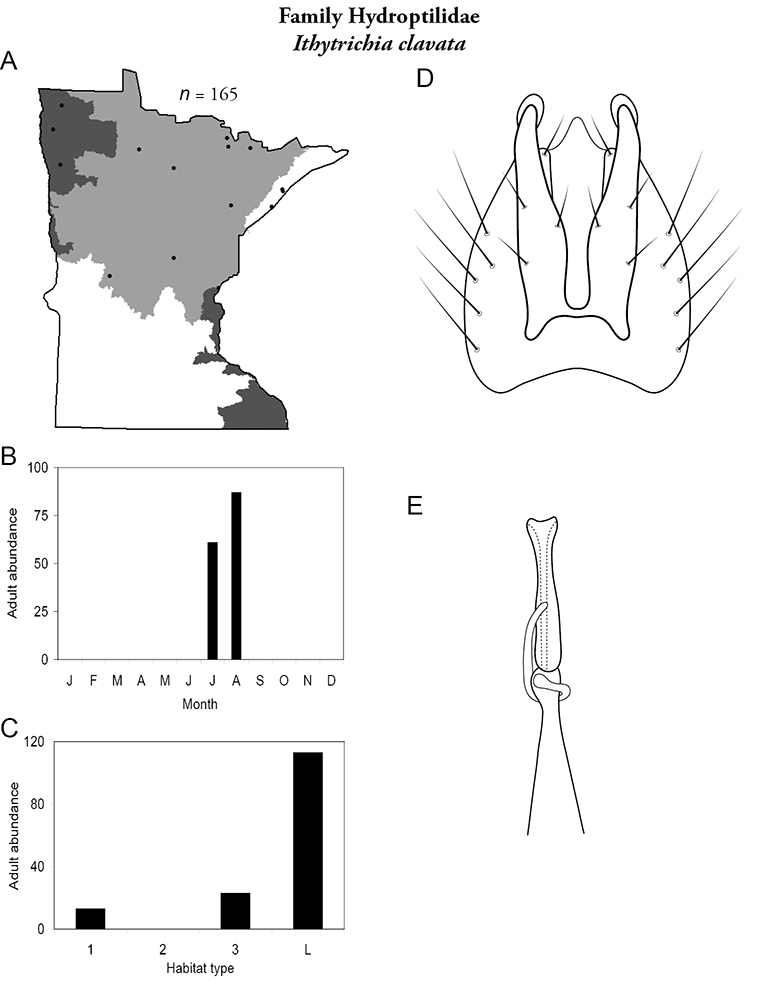
*Ithytrichia clavata* **A** total specimens collected and all known collecting localities (Figure 4) **B** monthly adult abundance (1980s to present) **C** habitat preference (1980s to present) (Table 1) **D** male genital capsule (ventral view) **E** phallus.

***Ithytrichia clavata*** (Figure 90) has been found sporadically from the northern half of the state during July and August. Surprisingly, it was primarily collected from lakes. Numerous other reports (Moulton and Stewart 1996, Wiggins 1996, Houghton and Stewart 1998) note *Ithytrichia clavata* as a lotic species.

### Genus *Leucotrichia*

The genus *Leucotrichia* contains a single species in a Minnesota. For additional species, see Flint (1970). Larvae are typically found in fast and cold streams where they consume periphyton from the surfaces of medium and large stream rocks (Wiggins 1996). They have a distinctive appearance, with abdominal segments V and VI greatly expanded laterally. Larval aggregations can be quite locally abundant. Adults are nearly white in color.

**Figure 91.**
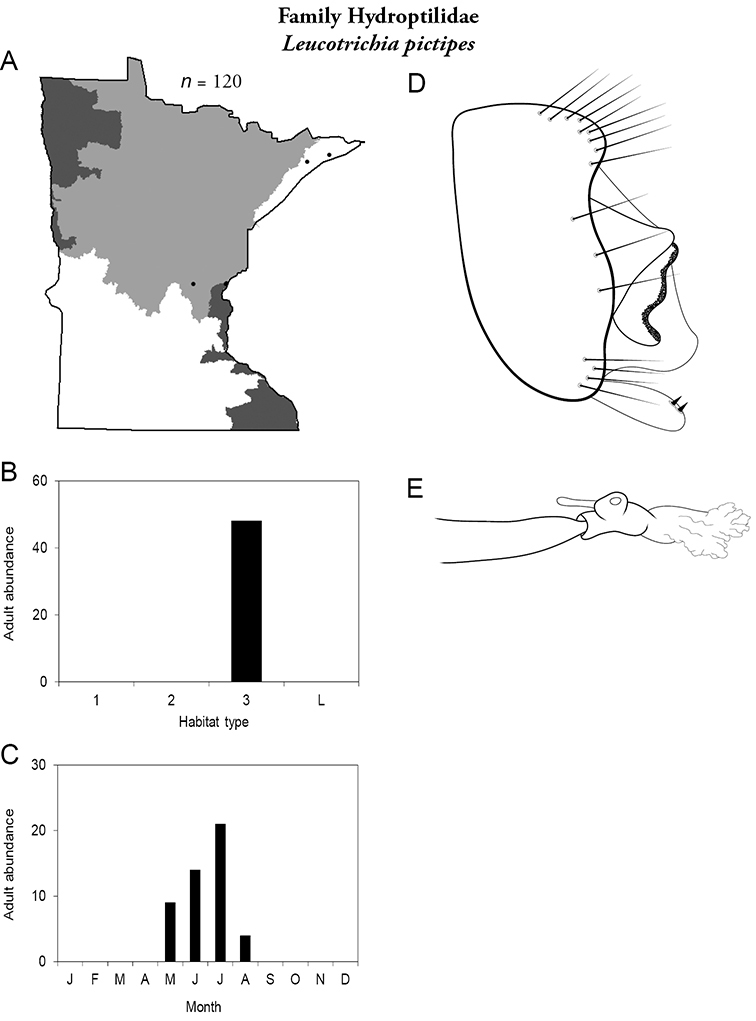
*Leucotrichia pictipes* **A** total specimens collected and all known collecting localities (Figure 4) **B** monthly adult abundance (1980s to present) **C** habitat preference (1980s to present) (Table 1) **D** male genital capsule **E** phallus.

***Leucotrichia pictipes*** (Figure 91) is known from the Lake Superior and Northern Regions, where it appears rare but locally abundant. All specimens have been collected from large rivers. Adults have been collected from May to August. In addition, hundreds of larvae have also been found throughout the same area. Since *Leucotrichia pictipes* is the only known *Leucotrichia* species in the eastern U.S., it is assumed that these larvae are all of this species.

### Genus *Mayatrichia*

The genus *Mayatrichia* contains a single species in a Minnesota. Larvae are typically found in fast-moving areas of medium and large rivers. They appear to consume small organic particles (Wiggins 1996). Adults are macroscopically indistinguishable from other hydroptilid genera.

**Figure 92.**
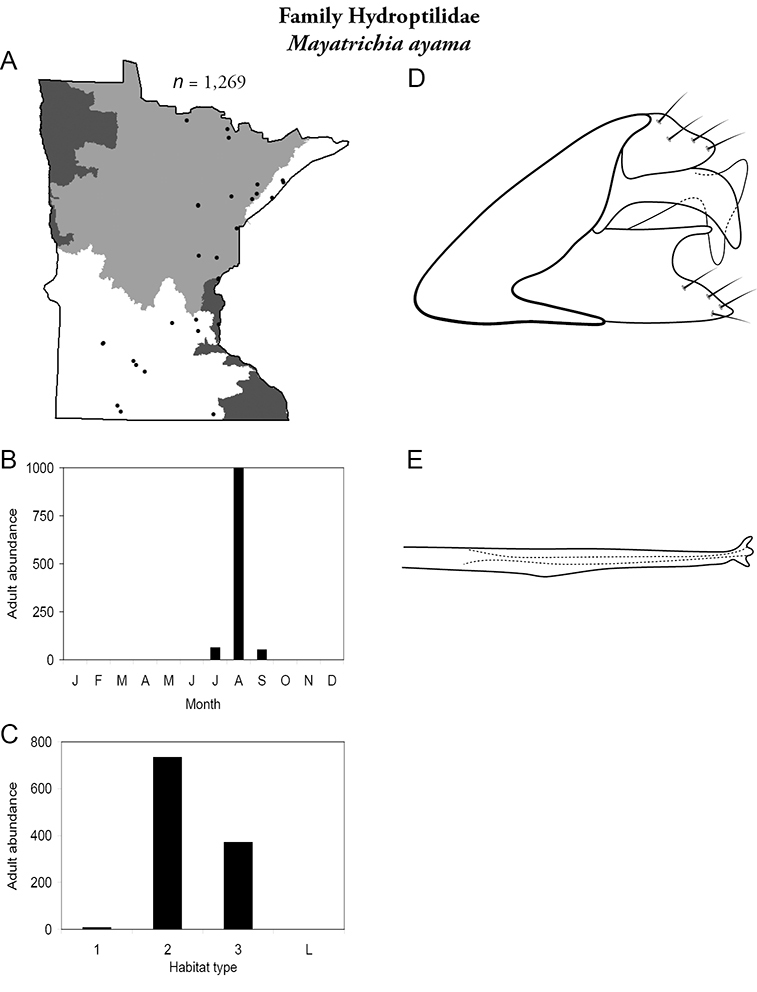
*Mayatrichia ayama* **A** total specimens collected and all known collecting localities (Figure 4) **B** monthly adult abundance (1980s to present) **C** habitat preference (1980s to present) (Table 1) **D** male genital capsule **E** phallus.

***Mayatrichia ayama*** (Figure 92) is known primarily from the Northern and Southern Regions, and sporadically elsewhere. It was found almost exclusively from medium and large rivers, primarily during August.

### Genus *Neotrichia*

The genus *Neotrichia* contains 5 species in Minnesota. They are the smallest of the microcaddisflies, with adults usually around 2 mm in length. Larvae typically prefer large river habitats (Wiggins 1996). Beyond their small size, they are macroscopically indistinguishable from other hydroptilid genera. To properly identify males of several species, the phallus should be gently extruded from the cleared genital capsule.

**Figure 93.**
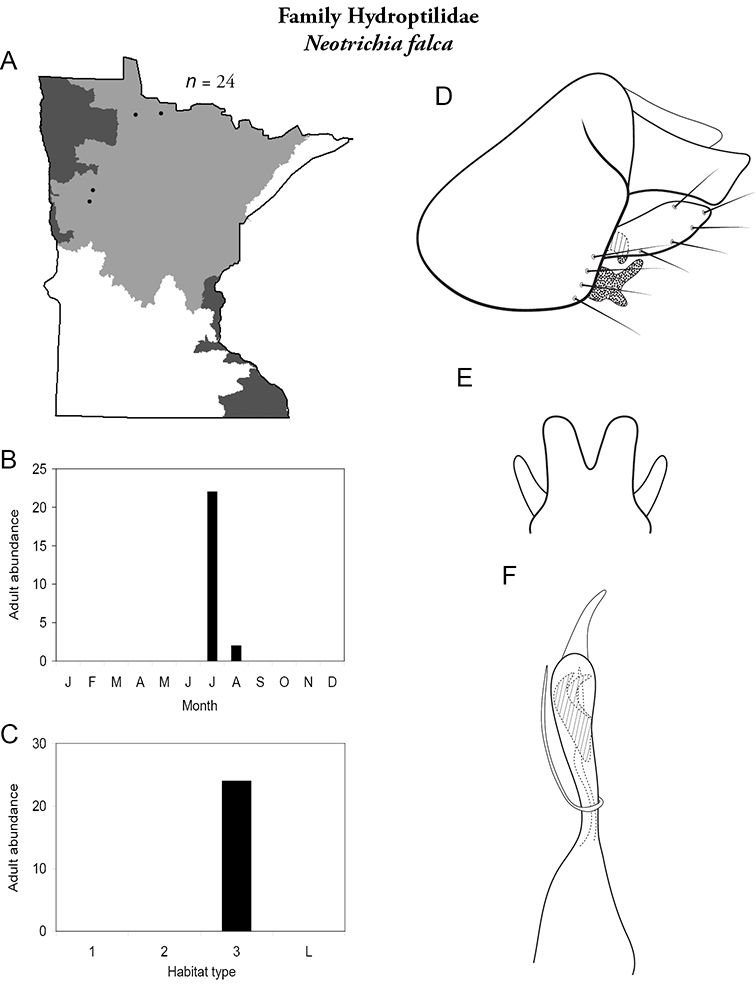
*Neotrichia falca* **A** total specimens collected and all known collecting localities (Figure 4) **B** monthly adult abundance (1980s to present) **C** habitat preference (1980s to present) (Table 1) **D** male genital capsule **E** male tergum X (dorsal view) **F** phallus.

***Neotrichia falca*** (Figure 93) is known only from large rivers of the Northern Region. It was most abundant in July, with some specimens present in August.

**Figure 94.**
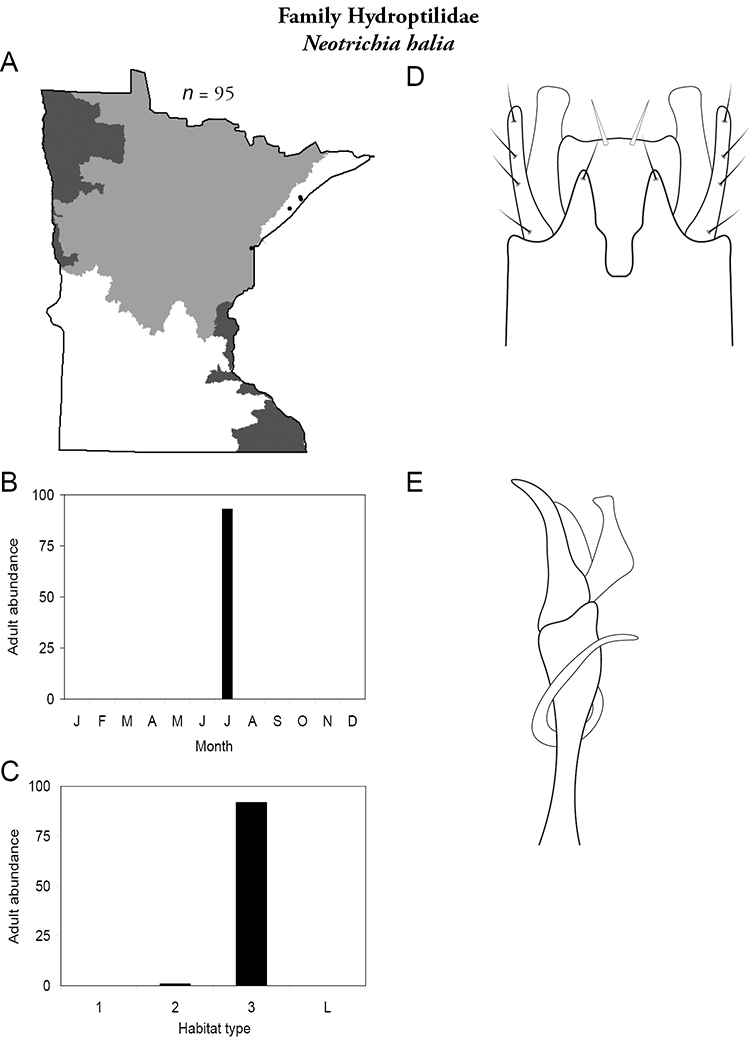
*Neotrichia halia* **A** total specimens collected and all known collecting localities (Figure 4) **B** monthly adult abundance (1980s to present) **C** habitat preference (1980s to present) (Table 1) **D** male genital capsule (ventral view) **E** phallus.

***Neotrichia halia*** (Figure 94) has been found in or near the Lake Superior Region, exclusively during July. It was collected almost entirely from large rivers.

**Figure 95.**
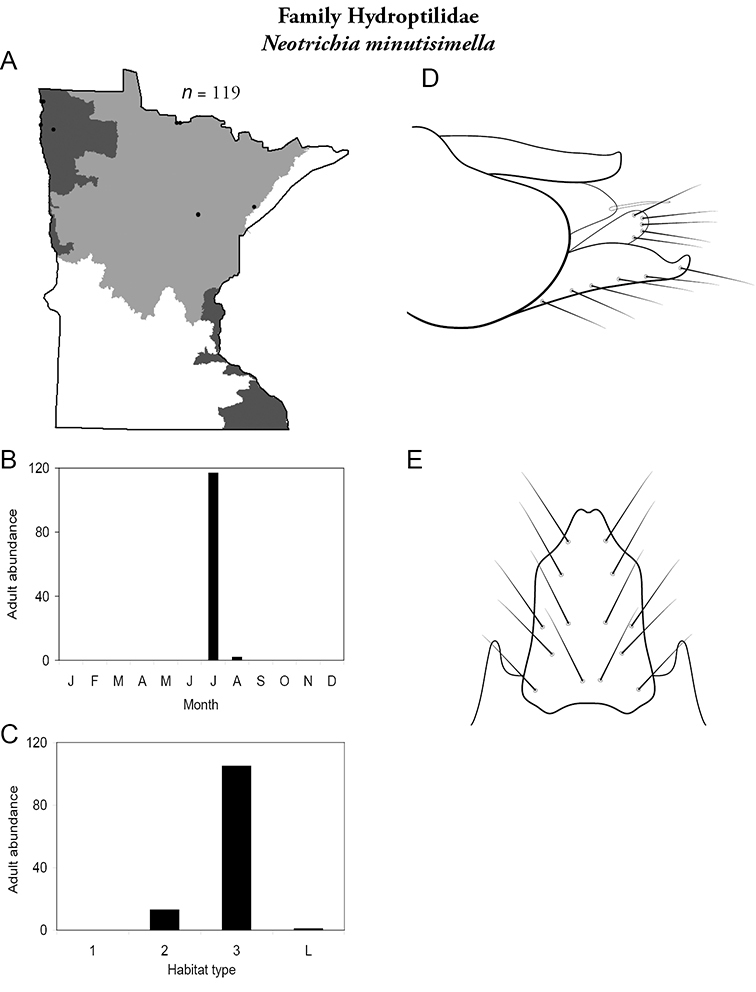
*Neotrichia minutisimella* **A** total specimens collected and all known collecting localities (Figure 4) **B** monthly adult abundance (1980s to present) **C** habitat preference (1980s to present) (Table 1) **D** male genital capsule **E** male genital capsule (ventral view).

***Neotrichia minutisimella*** (Figure 95) may be the single smallest caddisfly in North America (Ross 1944), with adults around 1.5 mm. It was found only in the Northern and Northwestern Regions, and predominantly from large rivers. Adults were present almost exclusively in July.

**Figure 96.**
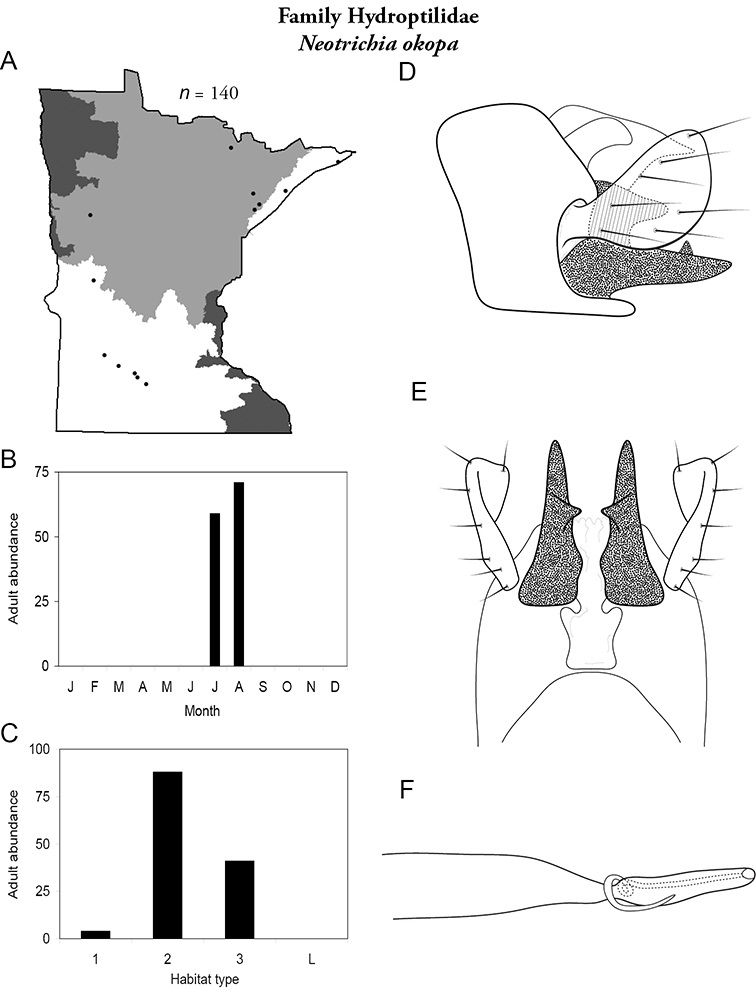
*Neotrichia okopa* **A** total specimens collected and all known collecting localities (Figure 4) **B** monthly adult abundance (1980s to present) **C** habitat preference (1980s to present) (Table 1) **D** male genital capsule **E** male genital capsule (ventral view) **F** phallus.

***Neotrichia okopa*** (Figure 96) is the only *Neotrichia* species in Minnesota that is most abundant in medium rivers, although it was also found in large rivers. Collections have occurred in the Lake Superior, Northern, and Southern Regions during July and August.

**Figure 97.**
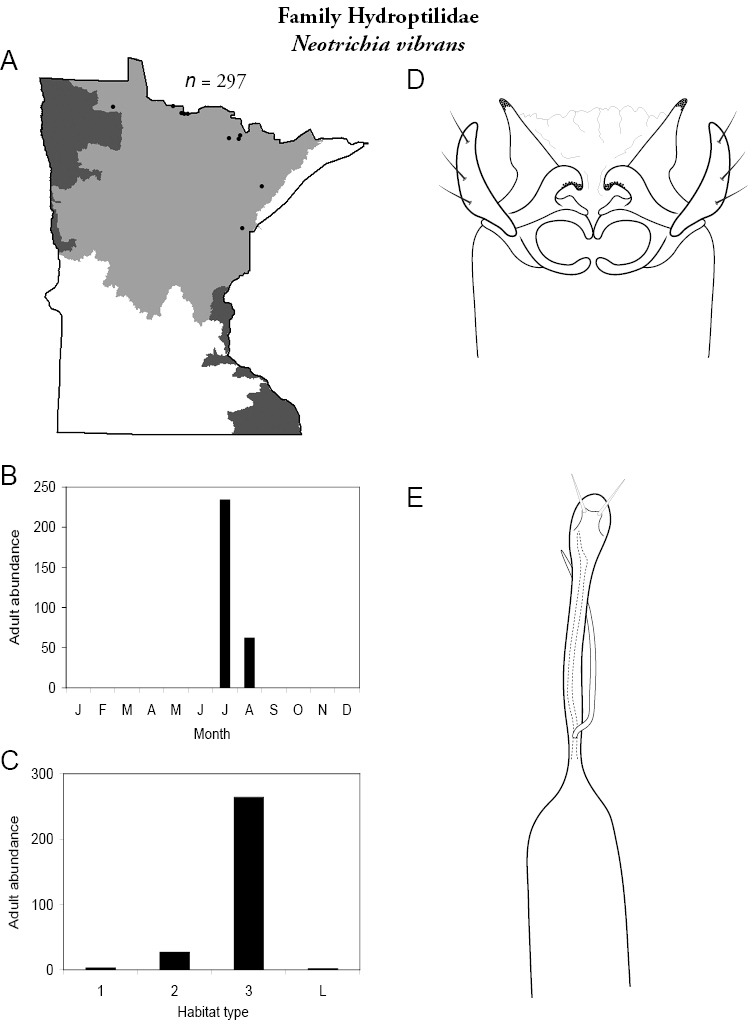
*Neotrichia vibrans* **A** total specimens collected and all known collecting localities (Figure 4) **B** monthly adult abundance (1980s to present) **C** habitat preference (1980s to present) (Table 1) **D** male genital capsule (ventral view) **E** phallus.

***Neotrichia vibrans*** (Figure 97) is known only from the Northern Region, and was found primarily during July. It was typically collected from large rivers.

### Genus *Ochrotrichia*

The genus *Ochrotrichia* contains 2 species in Minnesota. Larvae live in a variety of stream types and consume diatoms from rock surfaces (Wiggins 1996). Adults are macroscopically indistinguishable from other hydroptilid genera.

**Figure 98.**
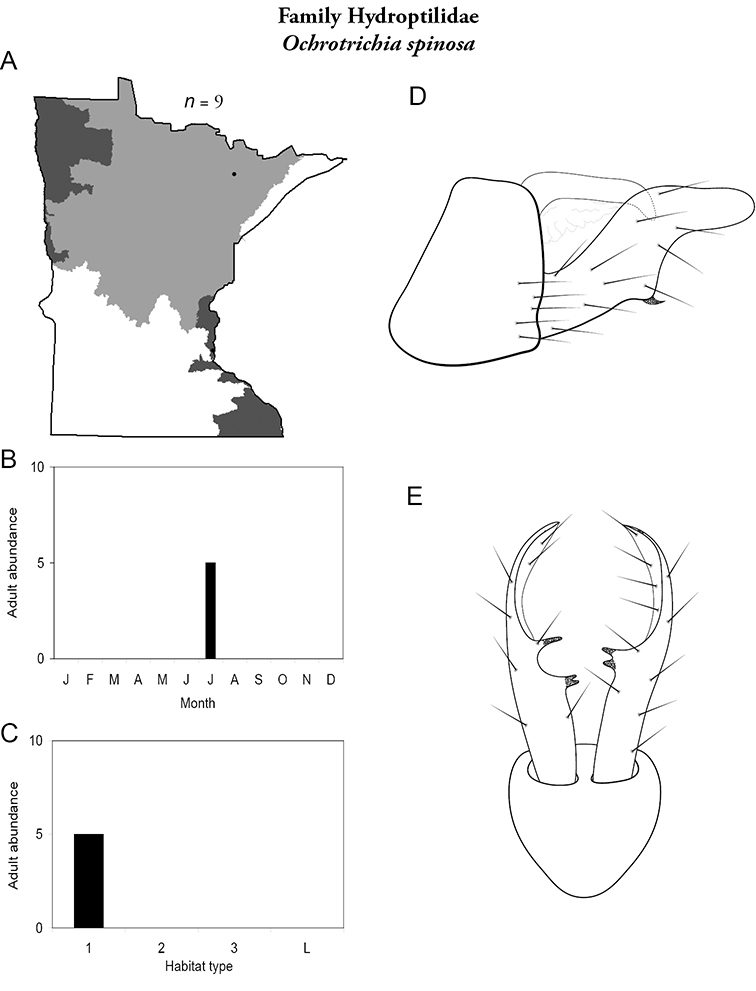
*Ochrotrichia spinosa* **A** total specimens collected and all known collecting localities (Figure 4) **B** monthly adult abundance (1980s to present) **C** habitat preference (1980s to present) (Table 1) **D** male genital capsule **E** male genital capsule (ventral view).

***Ochrotrichia spinosa*** (Figure 98) is known only from 2 small streams in the Northern and Southeastern Regions. Since 1960, the only collection occurred in Valley Creek, Washington County, in the Southeastern Region during July of 2001. Due to its rarity, and the high degree of habitat disturbance around Valley Creek, the Minnesota Department of Natural Resources has proposed “Endangered” status for the species (MNDNR 2012).

**Figure 99.**
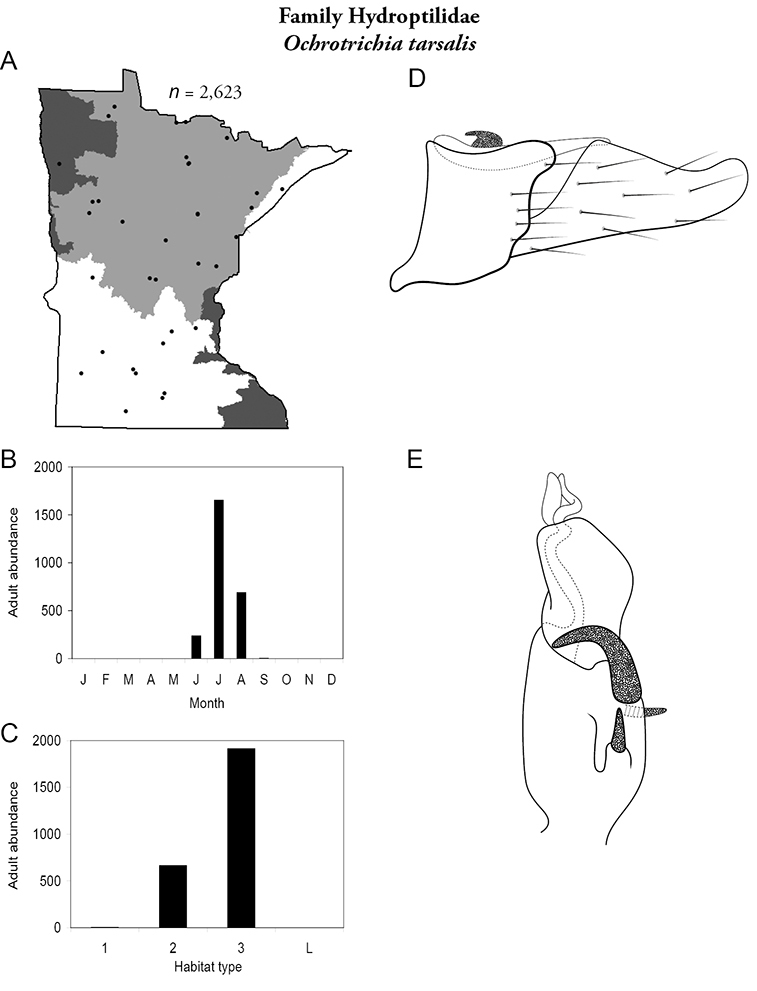
*Ochrotrichia tarsalis* **A** total specimens collected and all known collecting localities (Figure 4) **B** monthly adult abundance (1980s to present) **C** habitat preference (1980s to present) (Table 1) **D** male genital capsule **E** male tergum X (dorsal view).

***Ochrotrichia tarsalis*** (Figure 99) has been found primarily in the Northern and Southern Regions, entirely from medium and large rivers. Adults were most abundant in July and also present in June and August.

Two other *Ochrotrichia* species: *Ochrotrichia stylata* and *Ochrotrichia wojcicky*, were reported from Minnesota, both based on adult specimens of unknown sex (Denning and Blickle 1972). The whereabouts of these specimen is not known. Without specimens to confirm the records, *Ochrotrichia stylata* and *Ochrotrichia wojcicky* are not included in this manual.

### Genus *Orthotrichia*

The genus *Orthotrichia* contains 4 species in Minnesota, 3 of which are common throughout the Northern Region and frequently collected together. Larvae typically inhabit beds of submerged macrophytes in lakes and slow-moving areas of streams where they feed by piercing algal cells (Wiggins 1996). Adults are pale yellow in color. For additional species, see Kingsolver and Ross (1961).

**Figure 100.**
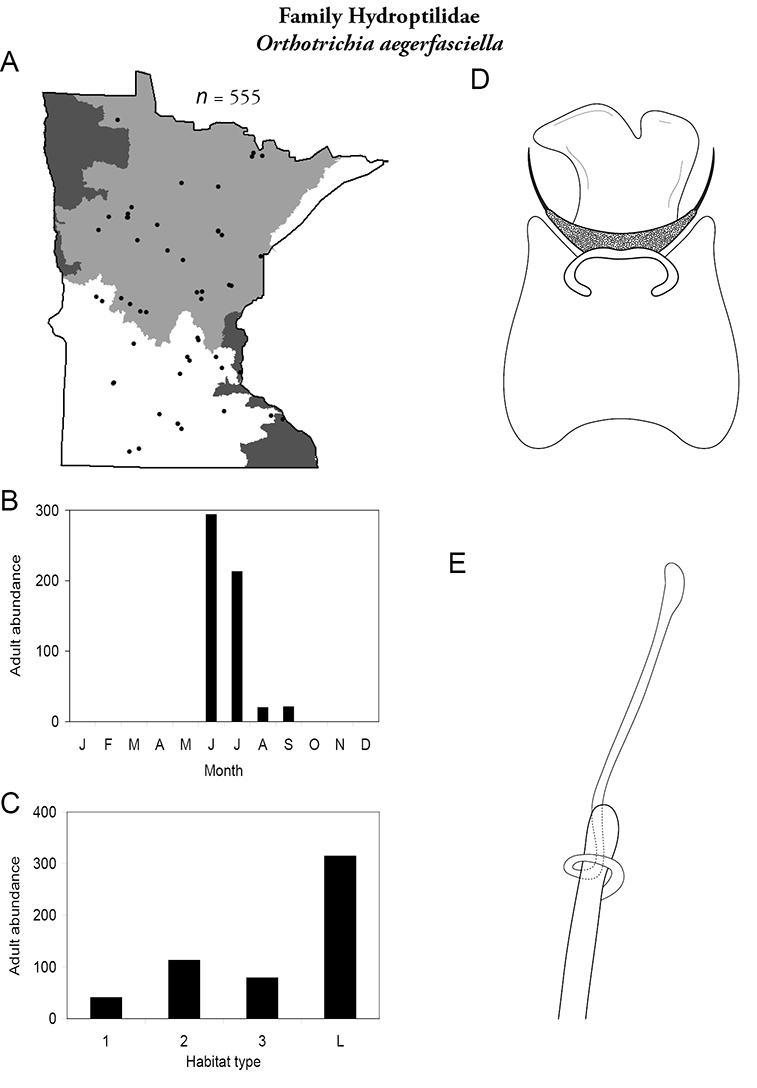
*Orthotrichia aegerfasciella* **A** total specimens collected and all known collecting localities (Figure 4) **B** monthly adult abundance (1980s to present) **C** habitat preference (1980s to present) (Table 1) **D** male genital (ventral view) **E** phallus.

***Orthotrichia aegerfasciella*** (Figure 100) was collected throughout the Northern and Southern Regions, predominantly from lakes, but also from all sizes of streams. Adults were abundant in June and July, and present in August and September.

**Figure 101.**
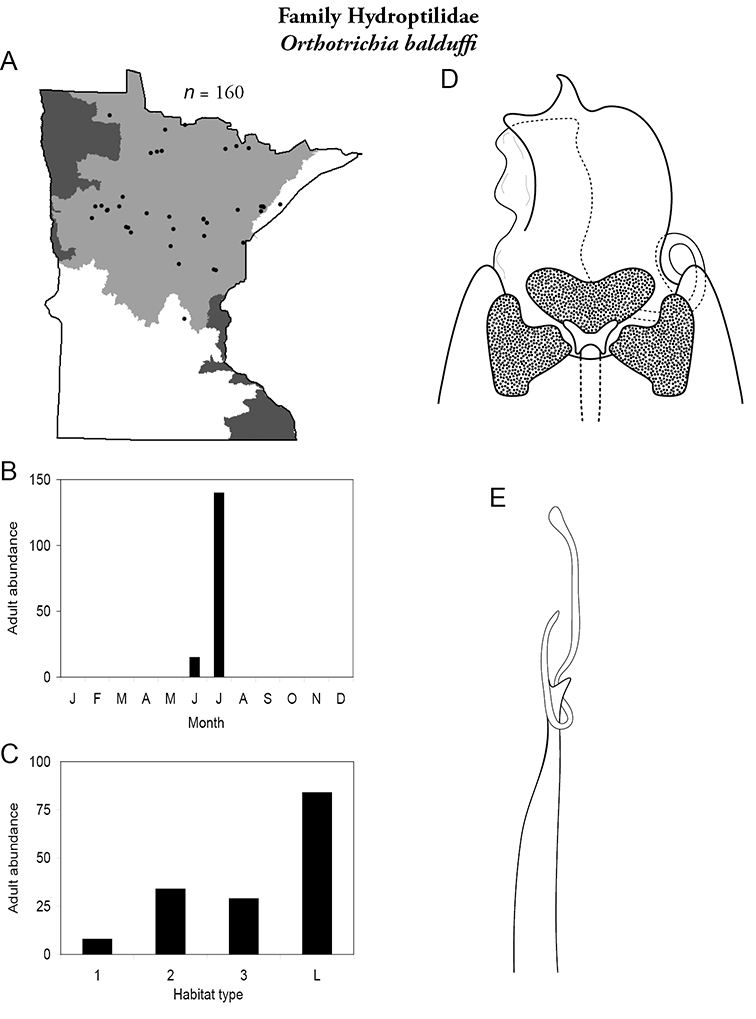
*Orthotrichia balduffi* **A** total specimens collected and all known collecting localities (Figure 4) **B** monthly adult abundance (1980s to present) **C** habitat preference (1980s to present) (Table 1) **D** male genital (ventral view) **E** phallus.

***Orthotrichia balduffi*** (Figure 101) has been found in or near the Northern Region, mostly during July. Like *Ochrotrichia aegerfasciella*, it was collected from all habitat types, but most frequently from lakes.

**Figure 102.**
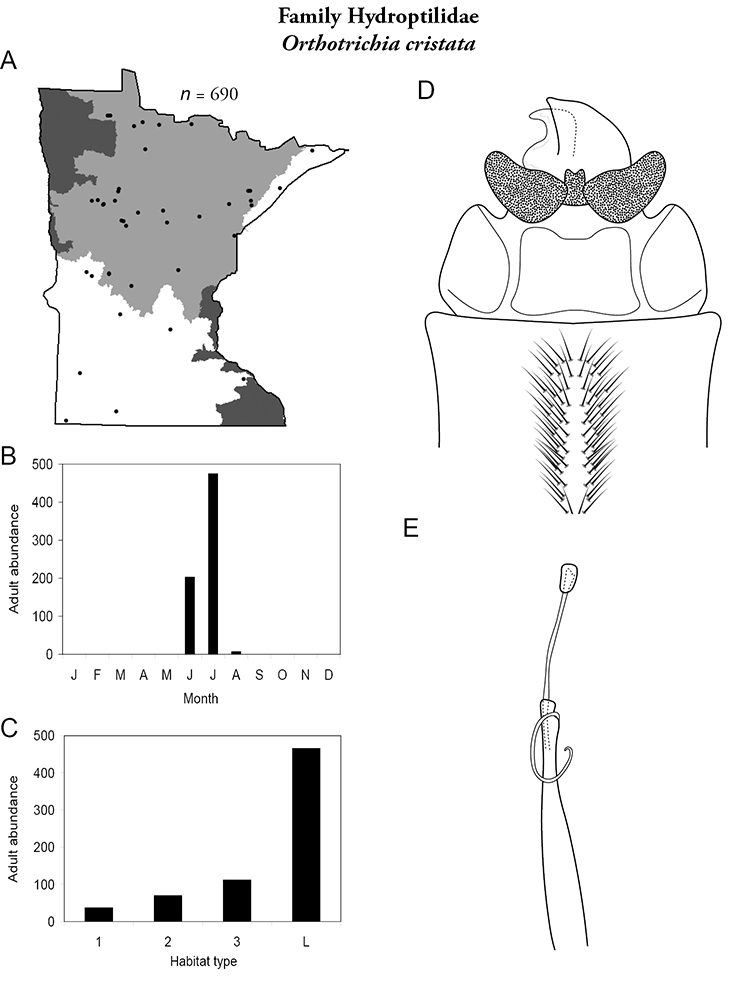
*Orthotrichia cristata* **A** total specimens collected and all known collecting localities (Figure 4) **B** monthly adult abundance (1980s to present) **C** habitat preference (1980s to present) (Table 1) **D** male genital (ventral view) **E** phallus.

***Orthotrichia cristata*** (Figure 102) was the most abundant *Orthotrichia* species, found throughout the Lake Superior, Northern, and Southern Regions. It, too, was most abundant in lakes and occasionally found in streams. Adults were present primarily during June and July, with a few found in August.

**Figure 103.**
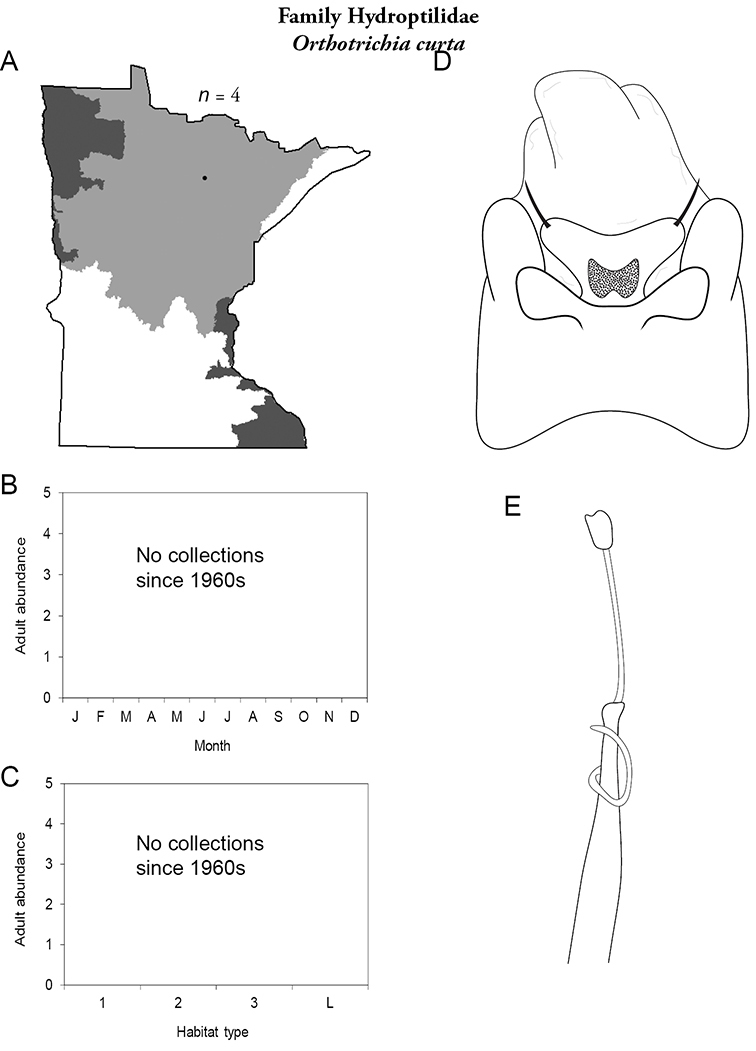
*Orthotrichia curta* **A** total specimens collected and all known collecting localities (Figure 4) **B** monthly adult abundance (1980s to present) **C** habitat preference (1980s to present) (Table 1) **D** male genital (ventral view) **E** phallus.

***Orthotrichia curta*** (Figure 103) is known only from a single specimen collected from Link (Lynx) Lake, Itasca County, in the Northern Region during July 1965. It has not been seen in Minnesota since this collection, and it is difficult to know if the species has been extirpated or is rare and difficult to collect.

### Genus *Oxyethira*

The genus *Oxyethira* contains 16 species in Minnesota. It is the 5th most species-rich genus in the state (Figure 7). Larvae are found in both lakes and a wide variety of streams where they feed on algal cells (Wiggins 1996). Adults are macroscopically indistinguishable from other hydroptilid genera (Figure 290). To properly identify males of many species, the phallus should be gently extruded from the cleared genital capsule. For additional species, see Kelley (1984, 1985, 1986).

**Figure 104.**
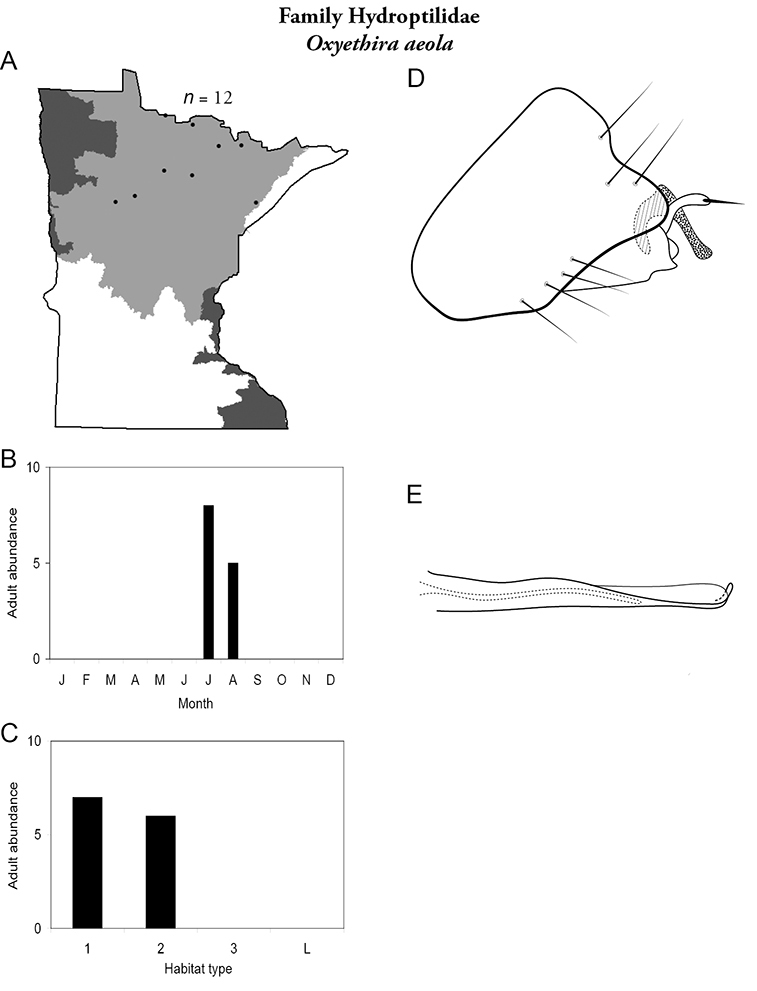
*Oxyethira aeola* **A** total specimens collected and all known collecting localities (Figure 4) **B** monthly adult abundance (1980s to present) **C** habitat preference (1980s to present) (Table 1) **D** male genital capsule **E** phallus.

***Oxyethira aeola*** (Figure 104) is known only from the Northern Region. It has been collected during July and August from small and medium streams.

**Figure 105.**
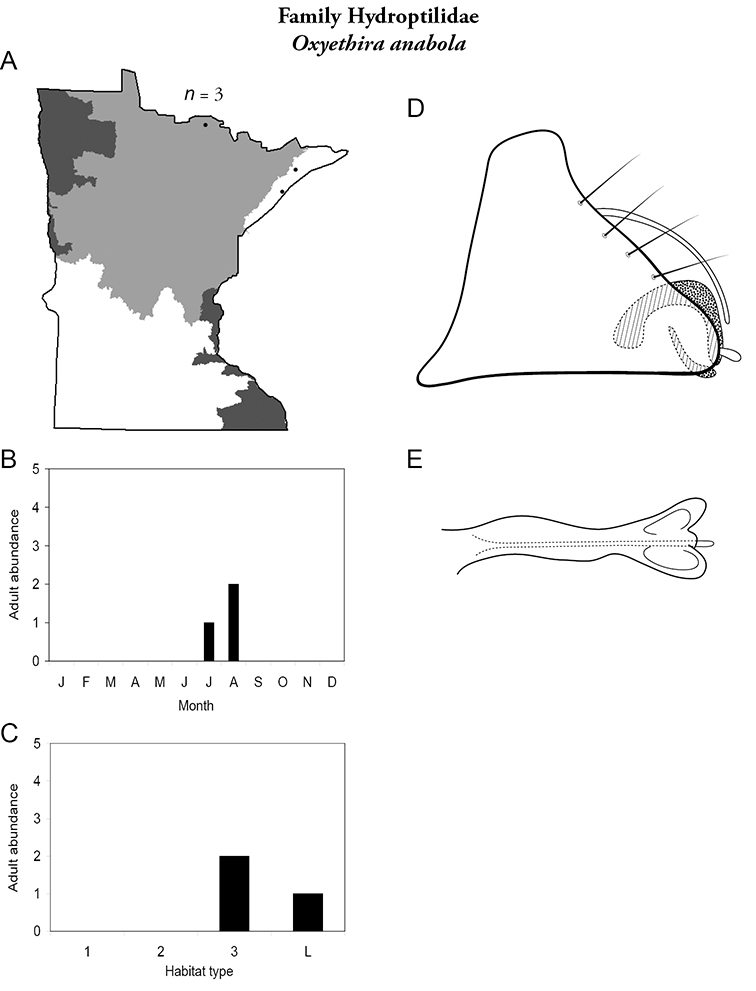
*Oxyethira anabola* **A** total specimens collected and all known collecting localities (Figure 4) **B** monthly adult abundance (1980s to present) **C** habitat preference (1980s to present) (Table 1) **D** male genital capsule **E** phallus.

***Oxyethira anabola*** (Figure 105) is known only from a few specimens found in the Lake Superior and Northern Regions during July and August. It was collected only from lakes and large rivers.

**Figure 106.**
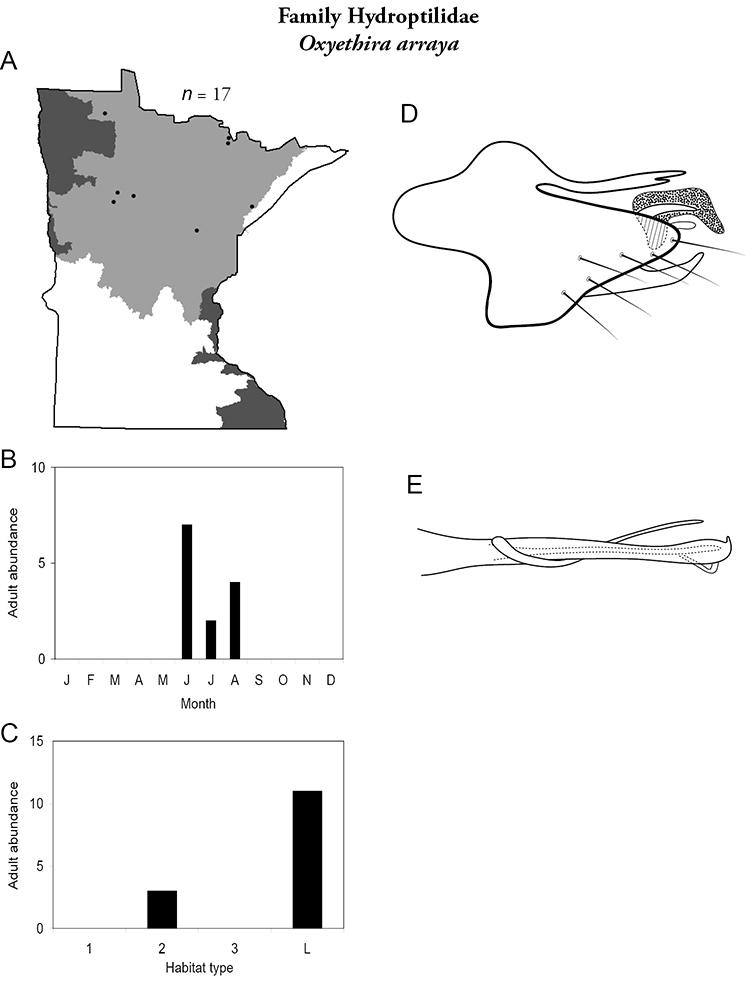
*Oxyethira arraya* **A** total specimens collected and all known collecting localities (Figure 4) **B** monthly adult abundance (1980s to present) **C** habitat preference (1980s to present) (Table 1) **D** male genital capsule **E** phallus.

***Oxyethira arraya*** (Figure 106) has been found in the Northern Region from June through August. Specimens were collected from lakes and medium rivers.

**Figure 107.**
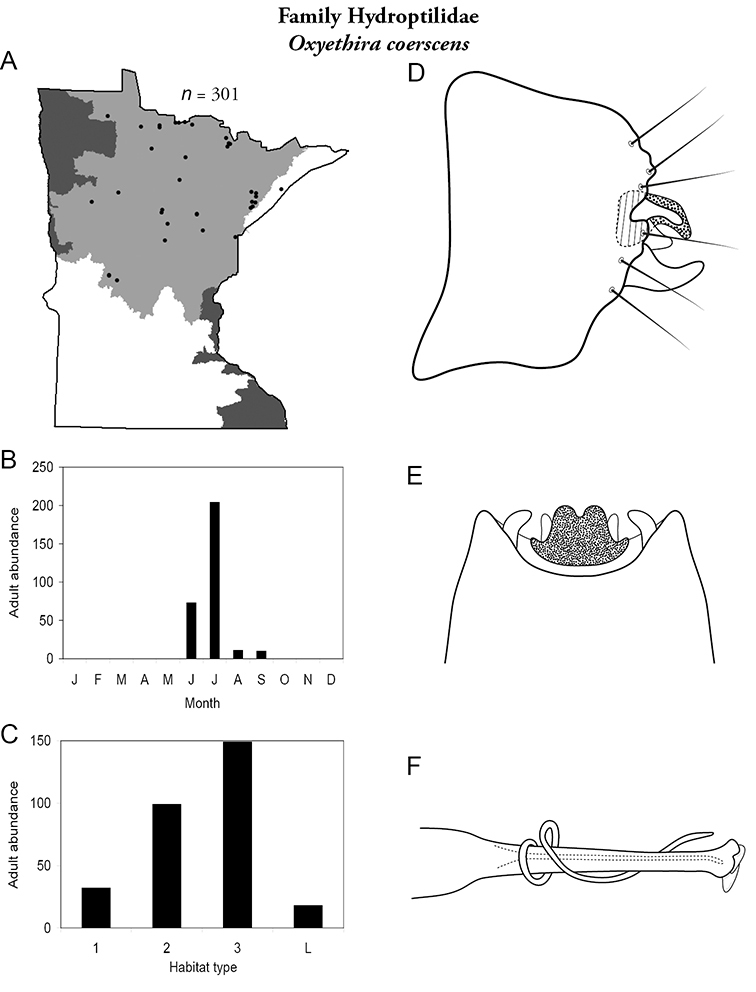
*Oxyethira coerscens* **A** total specimens collected and all known collecting localities (Figure 4) **B** monthly adult abundance (1980s to present) **C** habitat preference (1980s to present) (Table 1) **D** male genital capsule **E** male genital capsule (ventral view) **F** phallus.

***Oxyethira coerscens*** (Figure 107) is known primarily from the Northern Region. It was most abundant in medium and, especially, large rivers. Adults were abundant in June and July and also present in August and September.

**Figure 108.**
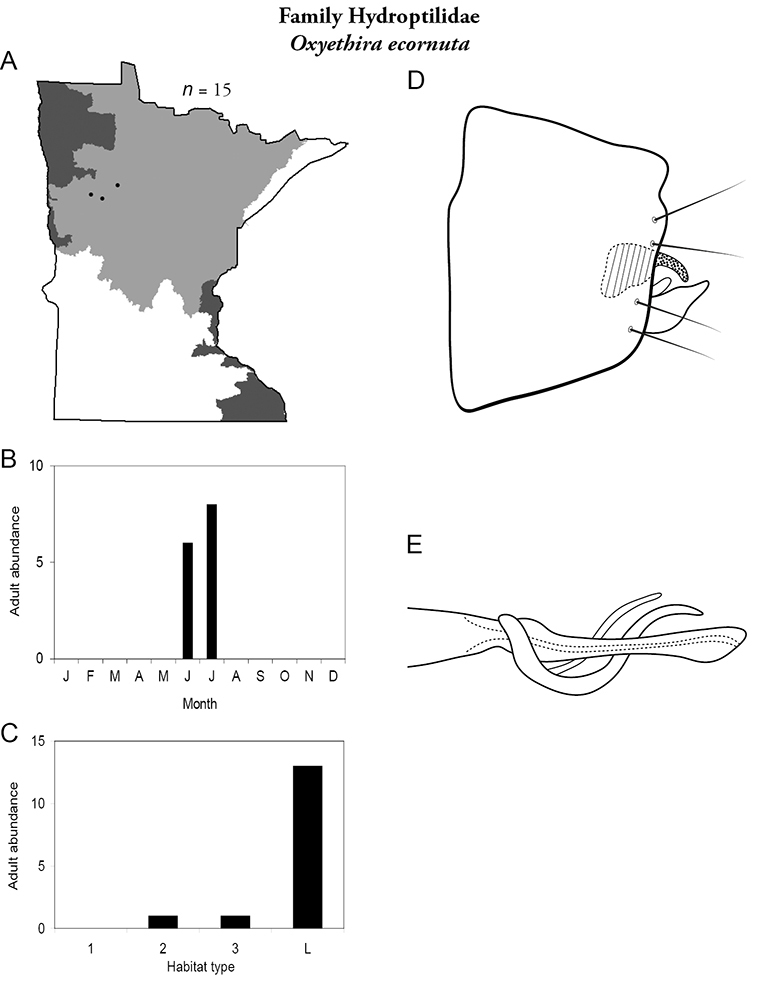
*Oxyethira ecornuta* **A** total specimens collected and all known collecting localities (Figure 4) **B** monthly adult abundance (1980s to present) **C** habitat preference (1980s to present) (Table 1) **D** male genital capsule **E** phallus.

***Oxyethira ecornuta*** (Figure 108) is known only from a few collections from lakes of the Northern Region during June and July. These are the only known collections of *Oxyethira ecornuta* from anywhere in the U.S. Due to its rarity and its exclusive presence in Minnesota, the Minnesota Department of Natural Resources has proposed “Threatened” status for the species.

**Figure 109.**
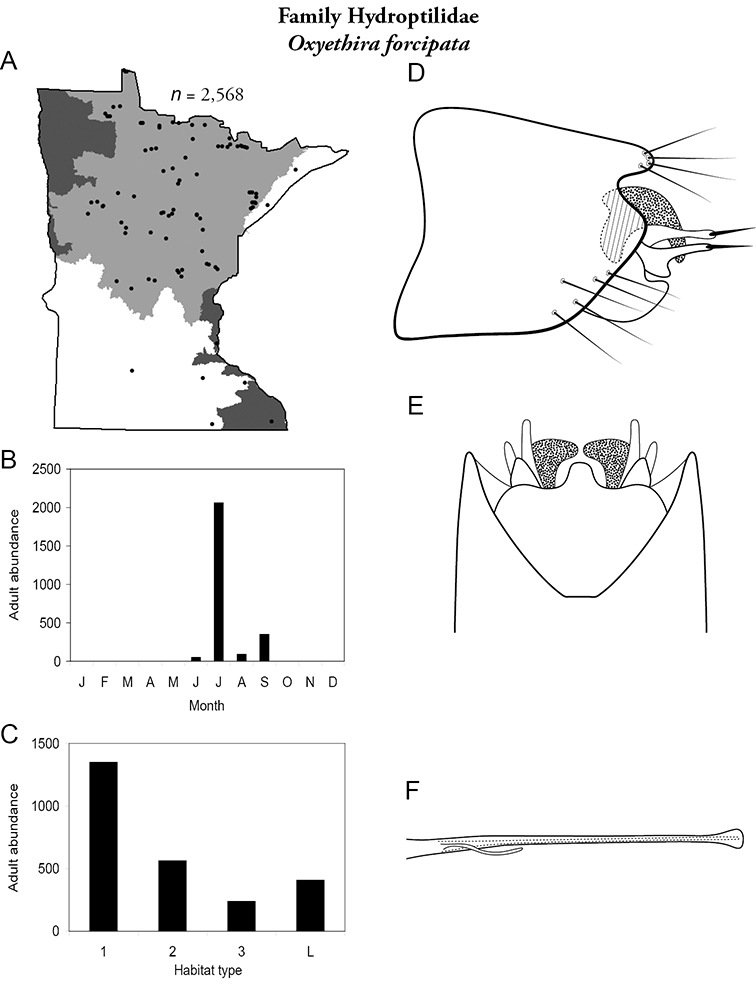
*Oxyethira forcipata* **A** total specimens collected and all known collecting localities (Figure 4) **B** monthly adult abundance (1980s to present) **C** habitat preference (1980s to present) (Table 1) **D** male genital capsule **E** male genital capsule (ventral view) **F** phallus.

***Oxyethira forcipata*** (Figure 109) was the 9th most widespread species overall in Minnesota (Figure 8). It was collected primarily in the Northern Region, and found in nearly every light trap from that region. It is also known sporadically from the Lake Superior, Southeastern, and Southern Regions. It was most abundant in small streams, but found in other habitat types as well. Adults were present from June to September and most abundant in July.

**Figure 110.**
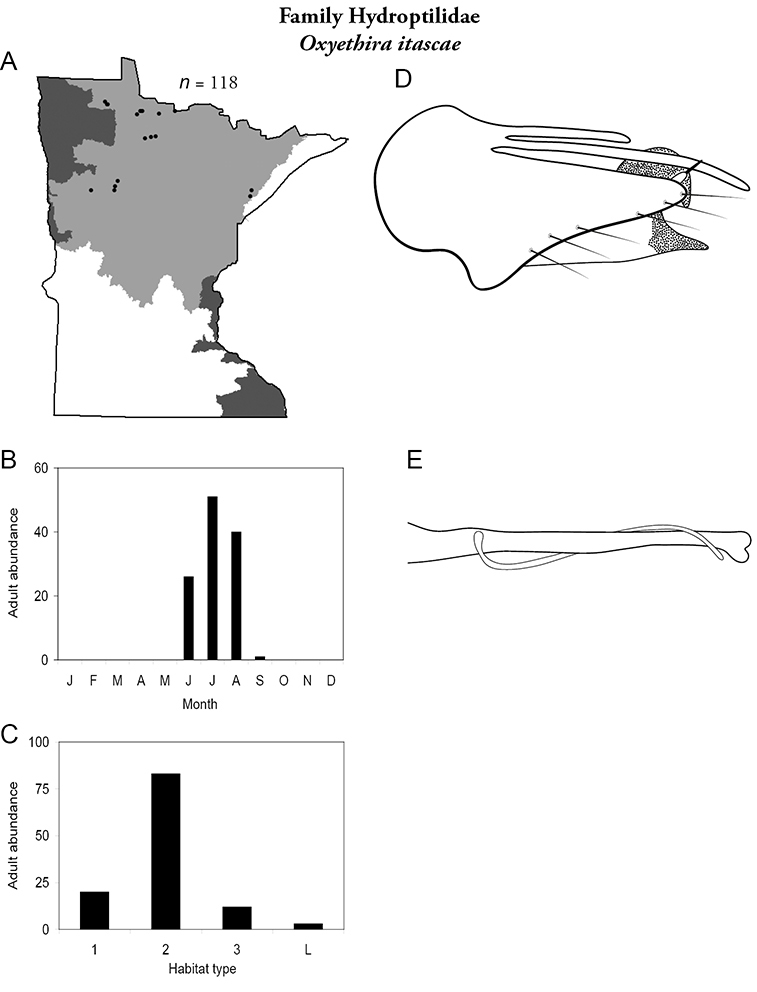
*Oxyethira itascae* **A** total specimens collected and all known collecting localities (Figure 4) **B** monthly adult abundance (1980s to present) **C** habitat preference (1980s to present) (Table 1) **D** male genital capsule **E** phallus.

***Oxyethira itascae*** (Figure 110) was described from Lake Itasca State Park (Monson and Holzenthal 1993). Since then, specimens have been collected from several other localities in the Northern Region. The species is most abundant in medium streams, and is present as an adult primarily from June through August. The collections from Minnesota have yielded the only known specimens of *Oxyethira itascae* worldwide. Thus, it is currently listed as “Threatened” by the Minnesota Department of Natural Resources (MNDNR 2012).

**Figure 111.**
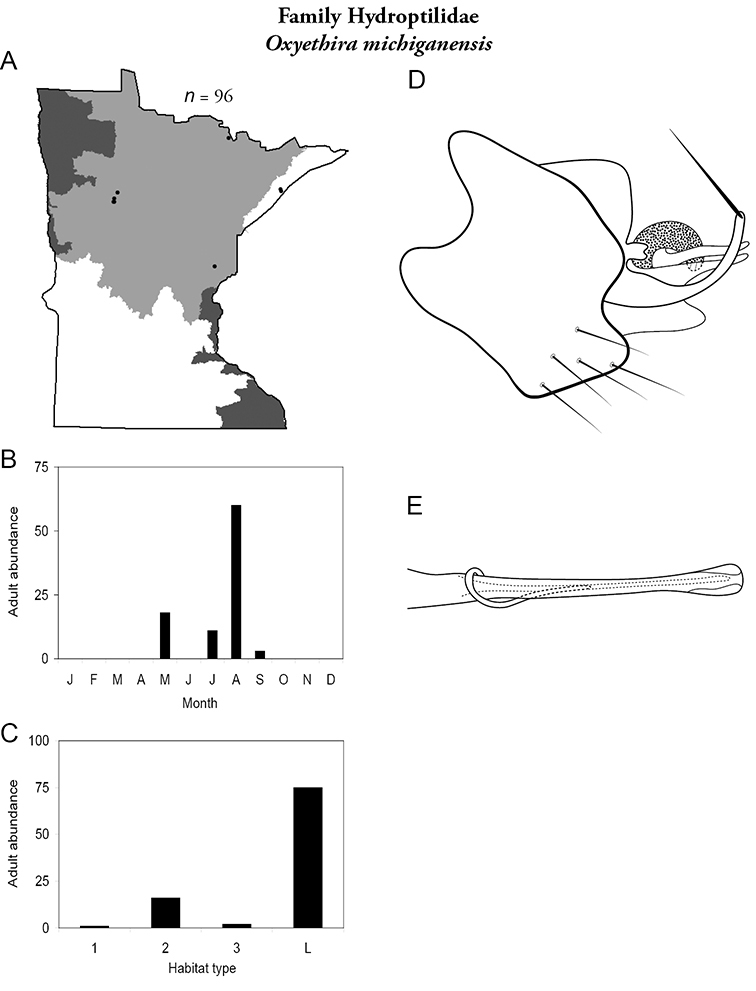
*Oxyethira michiganensis* **A** total specimens collected and all known collecting localities (Figure 4) **B** monthly adult abundance (1980s to present) **C** habitat preference (1980s to present) (Table 1) **D** male genital capsule **E** phallus.

***Oxyethira michiganensis*** (Figure 111) has been found in the Lake Superior and Northern Regions. It was collected mostly from lakes, with some specimens from medium rivers. Some adults emerged as early as May. The highest abundance, however, was in August, suggesting a possible bivoltine life cycle. Interestingly, in Michigan the species is probably univoltine, with peak emergence throughout June and July (Houghton et al. 2011b).

**Figure 112.**
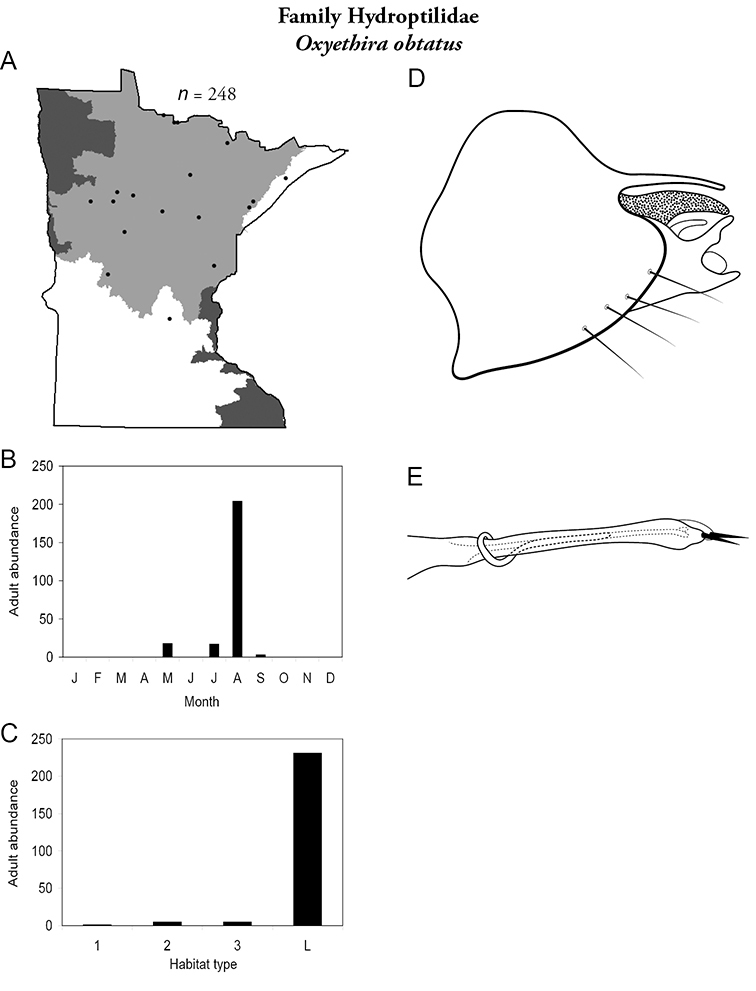
*Oxyethira obtatus* **A** total specimens collected and all known collecting localities (Figure 4) **B** monthly adult abundance (1980s to present) **C** habitat preference (1980s to present) (Table 1) **D** male genital capsule **E** phallus.

***Oxyethira obtatus*** (Figure 112) is known primarily from lakes of the Northern Region. Adults were collected primarily in August, with some present in May, July, and September.

**Figure 113.**
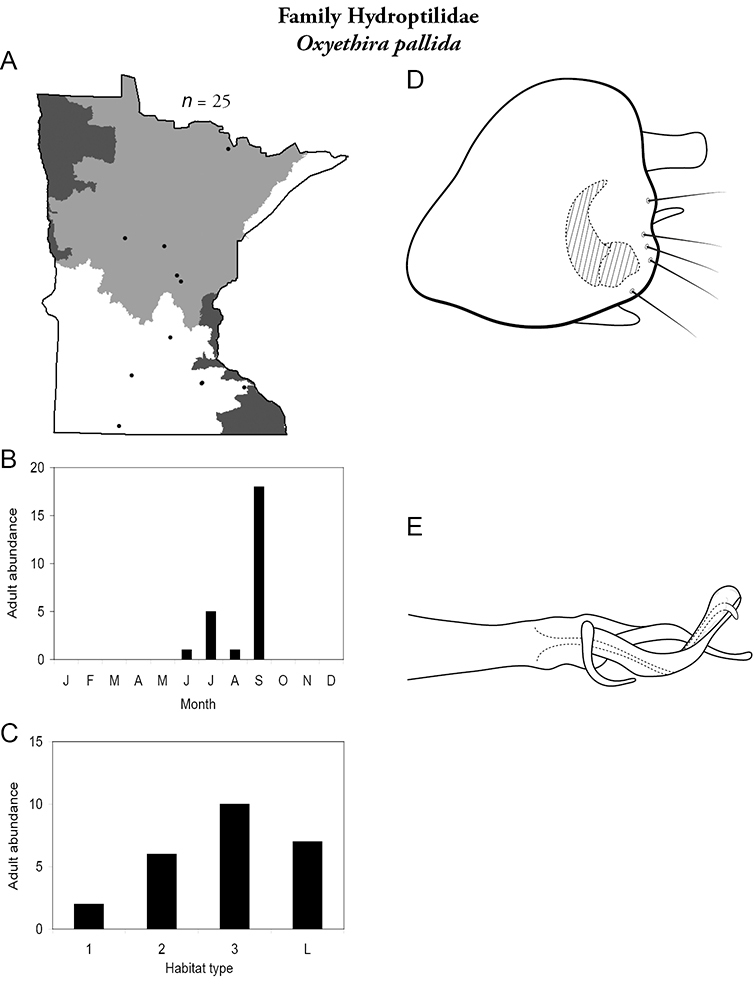
*Oxyethira pallida* **A** total specimens collected and all known collecting localities (Figure 4) **B** monthly adult abundance (1980s to present) **C** habitat preference (1980s to present) (Table 1) **D** male genital capsule **E** phallus.

***Oxyethira pallida*** (Figure 113) has been collected from the Northern and Southern Regions from all habitat types. Adults were most abundant in September, with some present from June through August.

**Figure 114.**
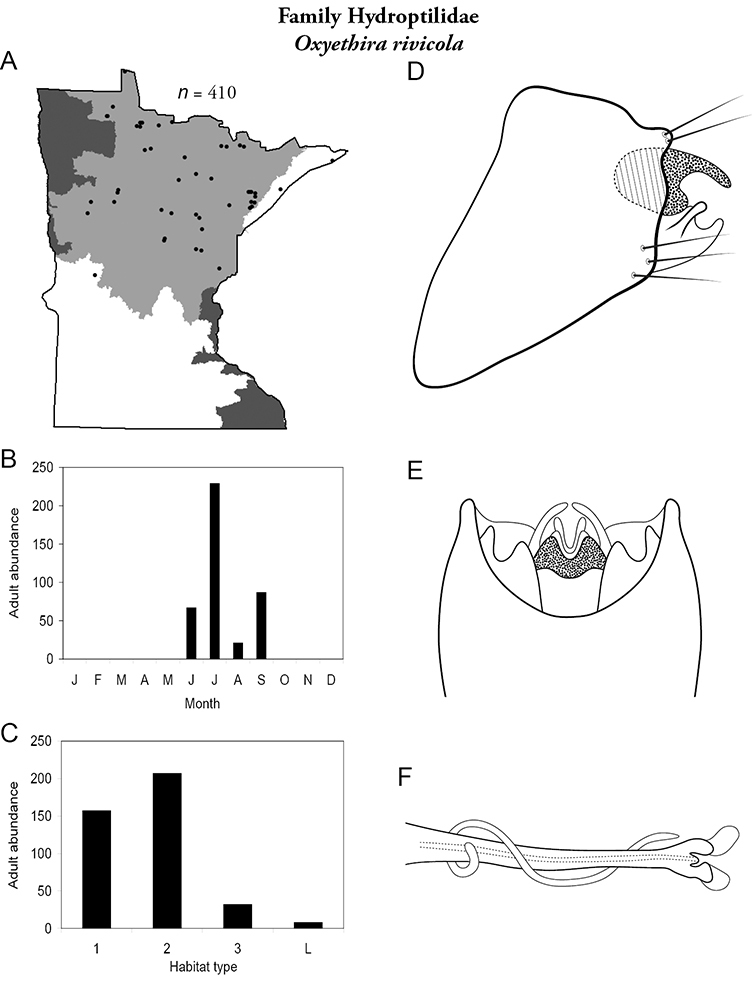
*Oxyethira rivicola* **A** total specimens collected and all known collecting localities (Figure 4) **B** monthly adult abundance (1980s to present) **C** habitat preference (1980s to present) (Table 1) **D** male genital capsule **E** male genital capsule (ventral view) **F** phallus.

***Oxyethira rivicola*** (Figure 114) has been collected primarily from the Northern Region, with some collections from the Lake Superior and Southern Regions. Adults were present from June through September, and most abundant in small and medium streams.

**Figure 115.**
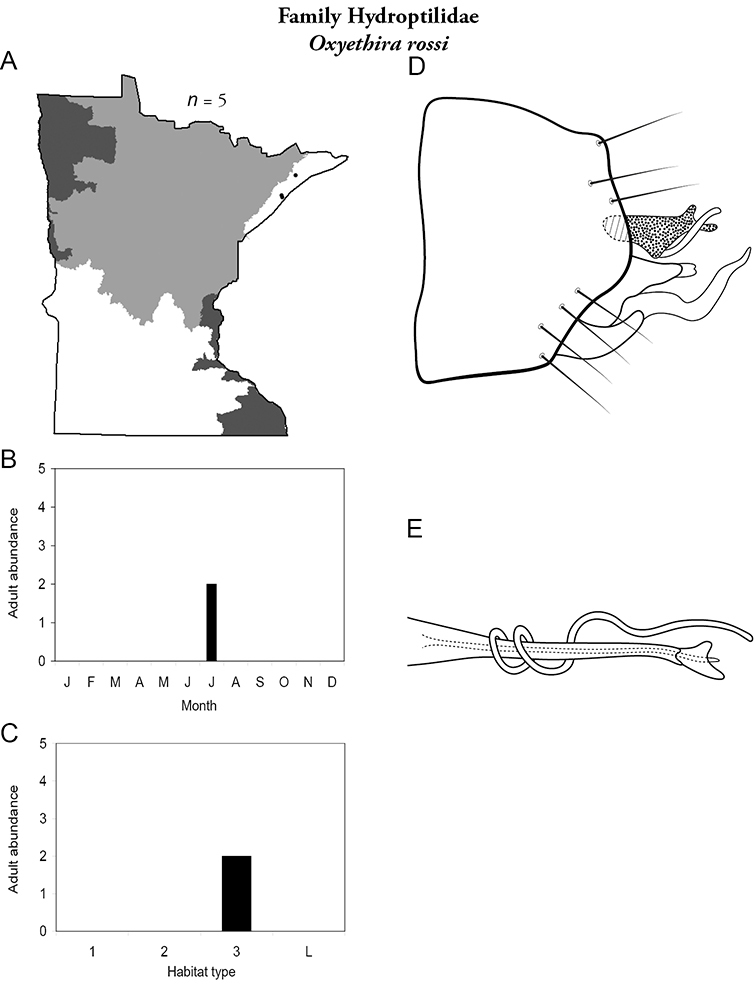
*Oxyethira rossi* **A** total specimens collected and all known collecting localities (Figure 4) **B** monthly adult abundance (1980s to present) **C** habitat preference (1980s to present) (Table 1) **D** male genital capsule **E** phallus.

***Oxyethira rossi*** (Figure 115) is known only from a couple of collections in the Lake Superior Region. All occurred during July and were from large rivers.

**Figure 116.**
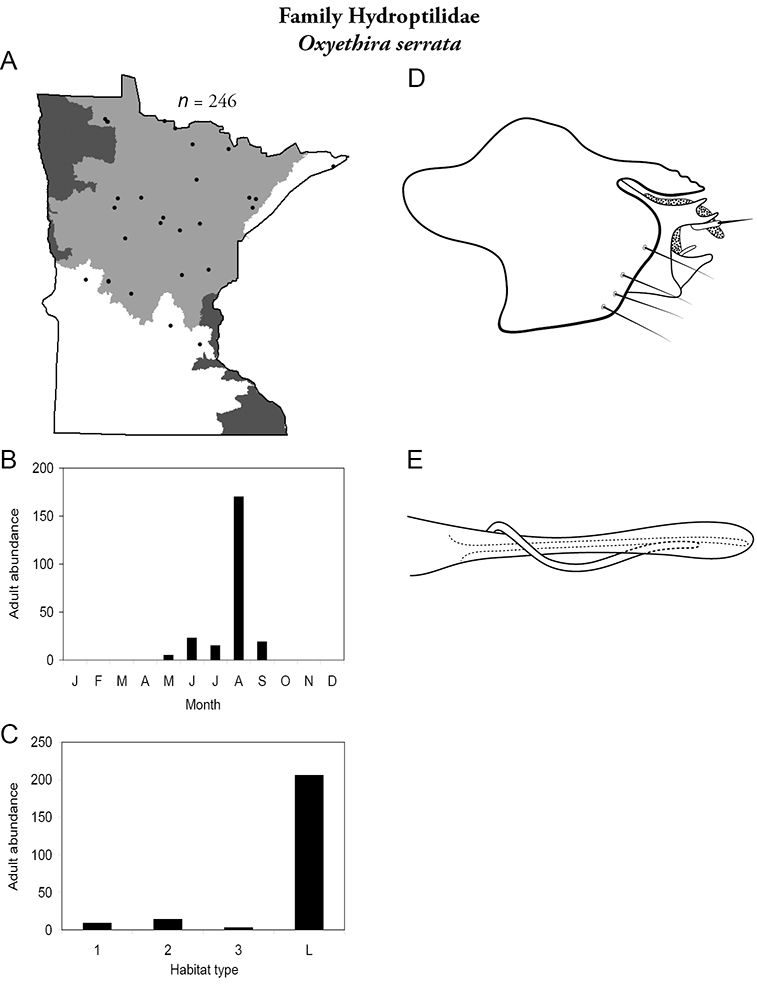
*Oxyethira serrata* **A** total specimens collected and all known collecting localities (Figure 4) **B** monthly adult abundance (1980s to present) **C** habitat preference (1980s to present) (Table 1) **D** male genital capsule **E** phallus.

***Oxyethira serrata*** (Figure 116) has been found mostly in the Northern Region and sporadically elsewhere. It was found almost exclusively in lakes. Adults were present from May to September, with highest abundance occurring in August.

**Figure 117.**
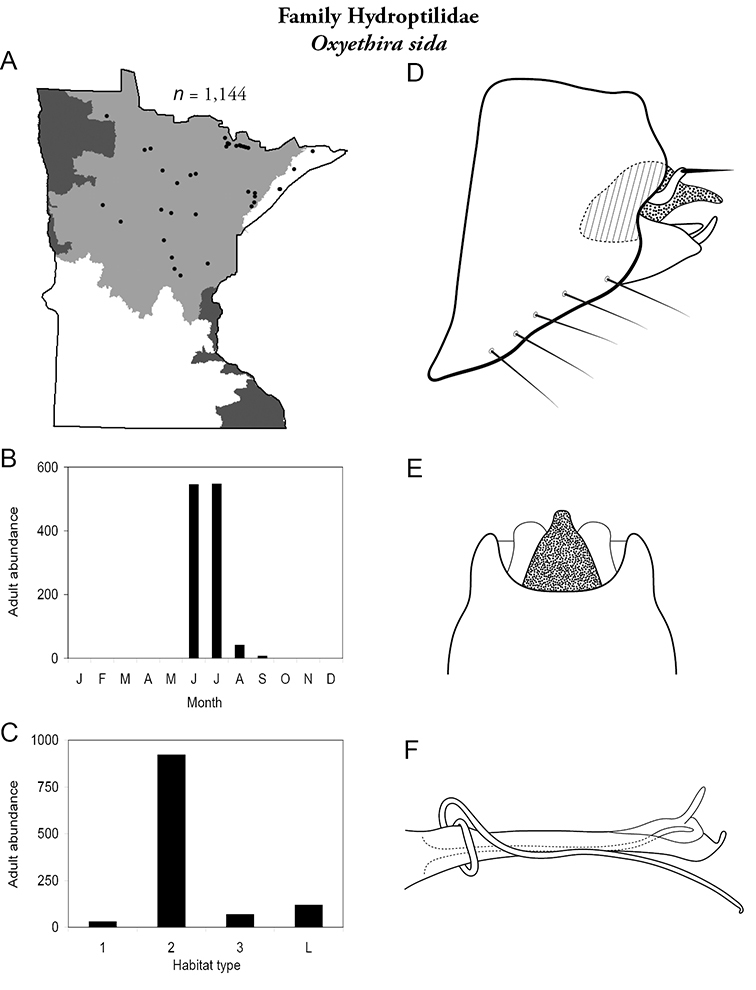
*Oxyethira sida* **A** total specimens collected and all known collecting localities (Figure 4) **B** monthly adult abundance (1980s to present) **C** habitat preference (1980s to present) (Table 1) **D** male genital capsule **E** male genital capsule (ventral view) **F** phallus.

***Oxyethira sida*** (Figure 117) is known from the Lake Superior and Northern Regions, primarily during June and July, from medium rivers.

**Figure 118.**
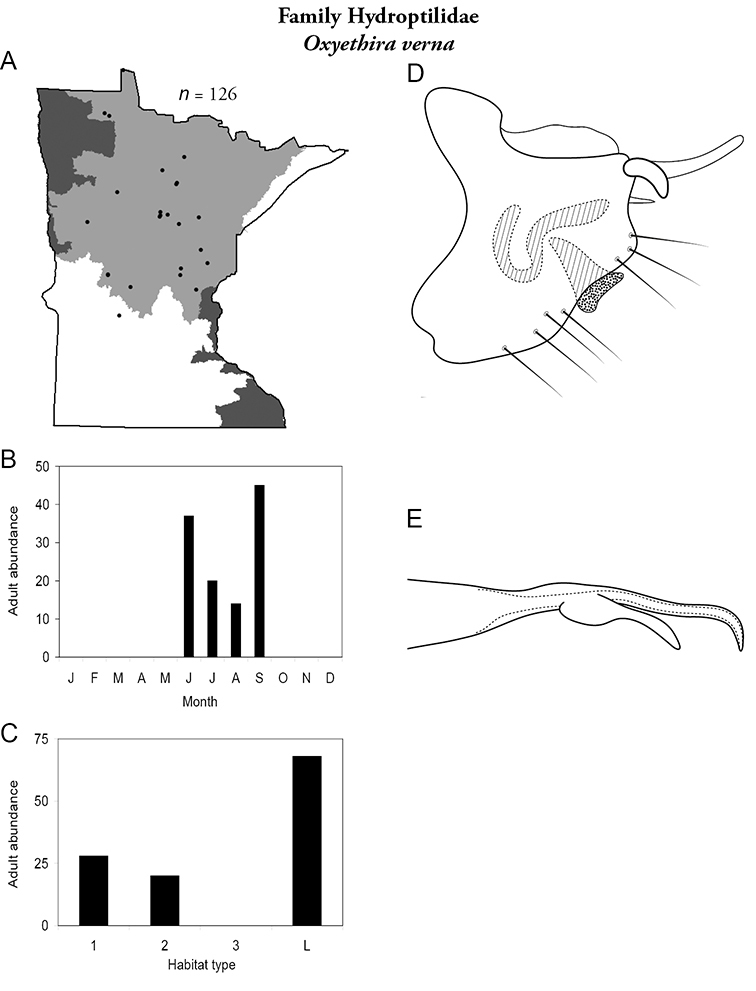
*Oxyethira verna* **A** total specimens collected and all known collecting localities (Figure 4) **B** monthly adult abundance (1980s to present) **C** habitat preference (1980s to present) (Table 1) **D** male genital capsule **E** phallus.

***Oxyethira verna*** (Figure 118) has been collected from and near the Northern Region. It was found in all habitats except large rivers, and present from June to September.

**Figure 119.**
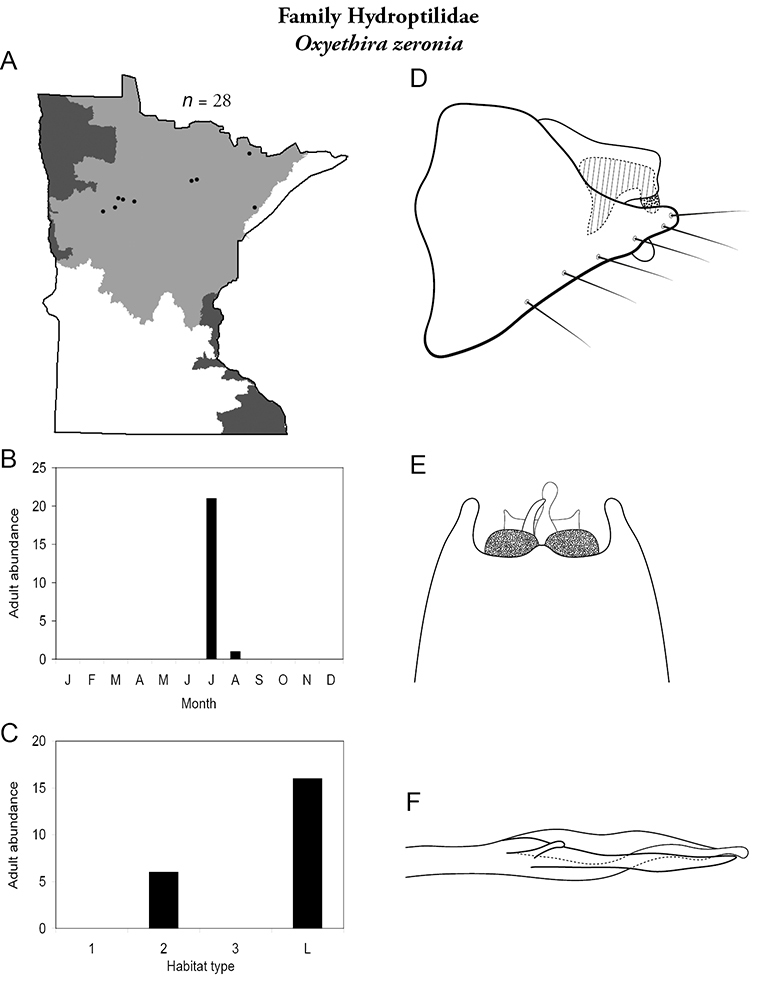
*Oxyethira zeronia* **A** total specimens collected and all known collecting localities (Figure 4) **B** monthly adult abundance (1980s to present) **C** habitat preference (1980s to present) (Table 1) **D** male genital capsule **E** male genital capsule (ventral view) **F** phallus.

***Oxyethira zeronia*** (Figure 119) has been found almost exclusively during July from lakes and medium rivers of the Northern Region.

### Genus *Stactobiella*

The genus *Stactobiella* contains 2 species from Minnesota. Neither are commonly encountered. Larvae usually inhabit fast-moving streams and consume algal cells or diatoms (Wiggins 1996). Adults are macroscopically indistinguishable from other hydroptilid genera.

**Figure 120.**
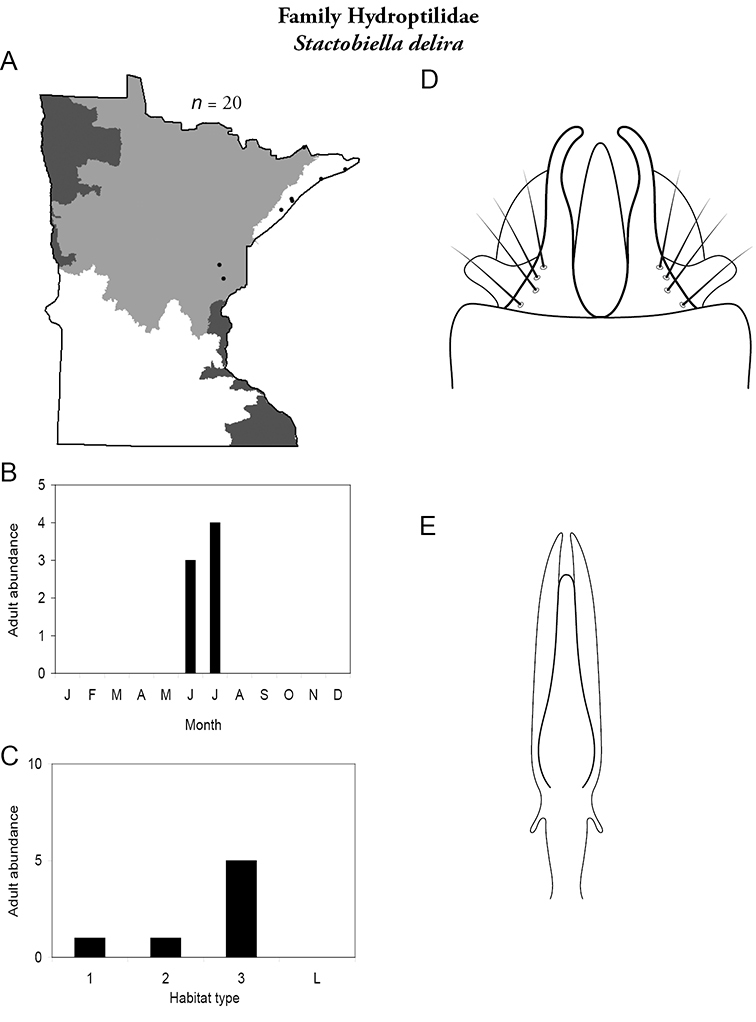
*Stactobiella delira* **A** total specimens collected and all known collecting localities (Figure 4) **B** monthly adult abundance (1980s to present) **C** habitat preference (1980s to present) (Table 1) **D** male genital capsule (ventral view) **E** phallus.

***Stactobiella delira*** (Figure 120) has been found in the Lake Superior and Northern Regions during June and July. It was found exclusively in streams, primarily large rivers.

**Figure 121.**
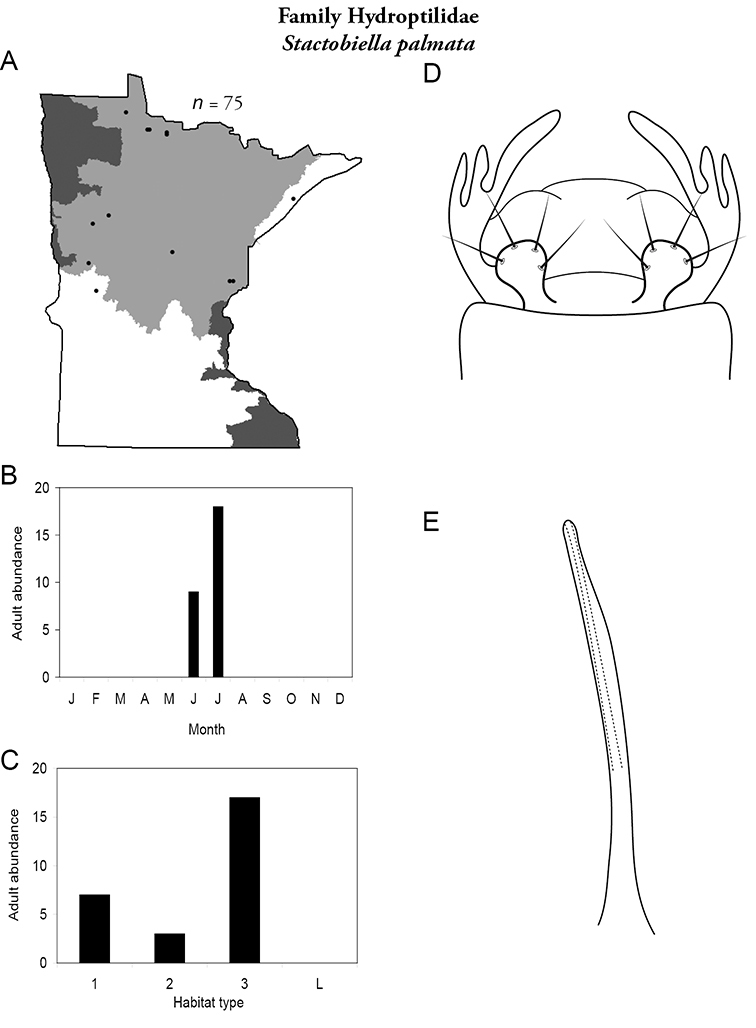
*Stactobiella palmata* **A** total specimens collected and all known collecting localities (Figure 4) **B** monthly adult abundance (1980s to present) **C** habitat preference (1980s to present) (Table 1) **D** male genital capsule (ventral view) **E** phallus.

***Stactobiella palmata*** (Figure 121) is primarily known from the Northern Region, and sporadically elsewhere. Like *Stactobiella delira*, it was found primarily in large rivers during June and July.

### Family Lepidostomatidae

This family contains a single genus in Minnesota, *Lepidostoma*, and a total of 10 species. Larvae are shredders and typically inhabit slow-moving areas of woodland streams with considerable canopy cover. Cases are quadrate tubes constructed of small elongate pieces of wood arranged transversely (Wiggins 1996). Adults are usually tan or brown in color and 8–10 mm in length. Males often have unusual secondary sexual characteristics, most notably enlargement and increased setation of the antennal scapes and maxillary palpi.

### Genus *Lepidostoma*

The genus *Lepidostoma* contains 10 species in Minnesota. It is the 9th most species-rich genus (Figure 7). Most of these species are known only from a single or a few collections. Others are rare but locally abundant. Only one species, *Lepidostoma togatum*, is common. For additional species, see Weaver (1988).

**Figure 122.**
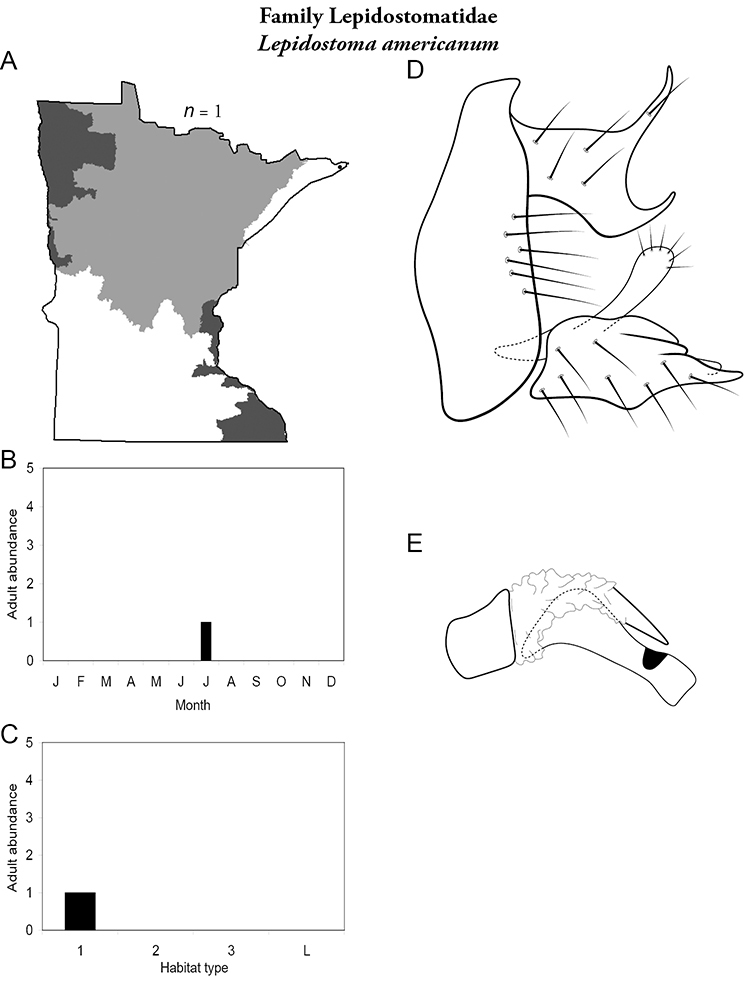
*Lepidostoma americanum* **A** total specimens collected and all known collecting localities (Figure 4) **B** monthly adult abundance (1980s to present) **C** habitat preference (1980s to present) (Table 1) **D** male genital capsule **E** phallus.

***Lepidostoma americanum*** (Figure 122) is known only from a single specimen collected during July 2000 from a small unnamed spring near Grand Portage National Monument in the Lake Superior Region.

**Figure 123.**
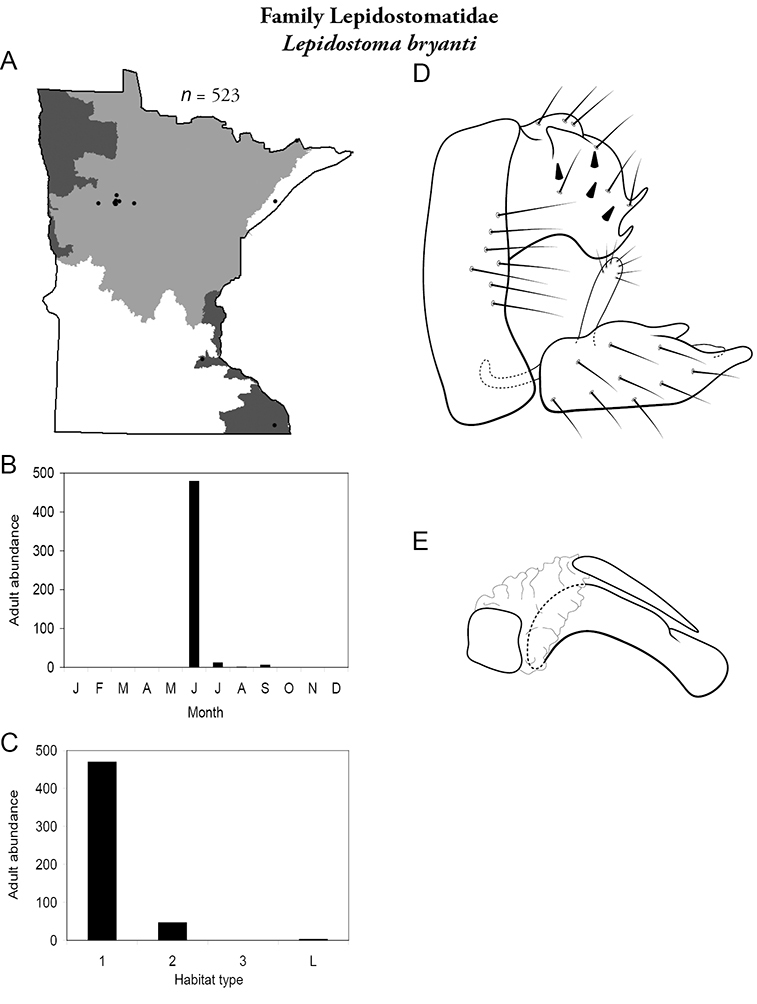
*Lepidostoma bryanti* **A** total specimens collected and all known collecting localities (Figure 4) **B** monthly adult abundance (1980s to present) **C** habitat preference (1980s to present) (Table 1) **D** male genital capsule **E** phallus.

***Lepidostoma bryanti*** (Figure 123) is known mostly from small streams in the Northern Region, with some collections occurring in the Lake Superior and Southeastern Regions. It was most abundant in June, with a few specimens collected in July and September. Nearly 75% of all specimens were collected from Sucker Creek, Clearwater County, in the Northern Region where the species appears to be locally abundant.

**Figure 124.**
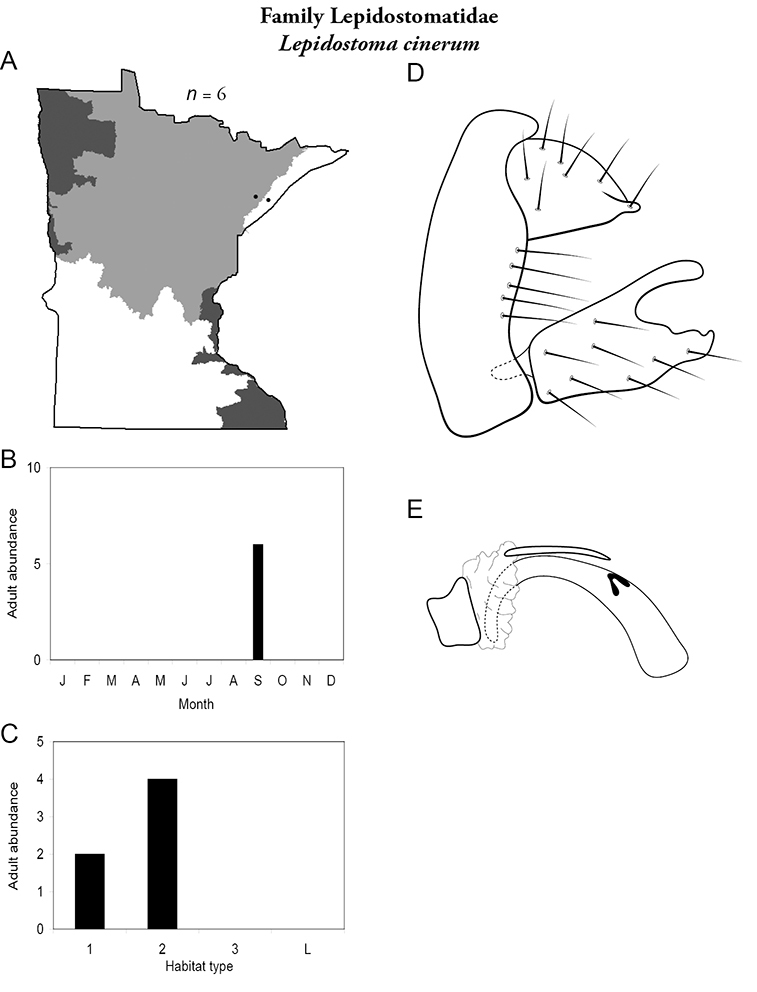
*Lepidostoma cinereum* **A** total specimens collected and all known collecting localities (Figure 4) **B** monthly adult abundance (1980s to present) **C** habitat preference (1980s to present) (Table 1) **D** male genital capsule **E** phallus.

***Lepidostoma cinerum*** (Figure 124) is known from only 6 specimens collected from a small and a medium stream in and near the Lake Superior Region during September 2000.

**Figure 125.**
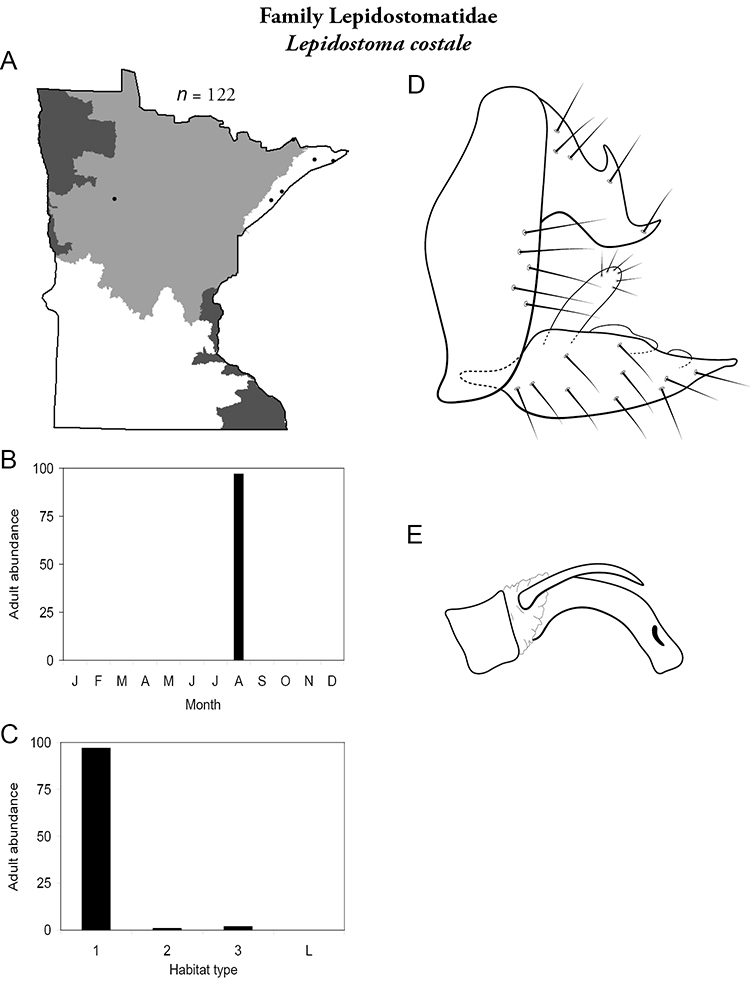
*Lepidostoma costale* **A** total specimens collected and all known collecting localities (Figure 4) **B** monthly adult abundance (1980s to present) **C** habitat preference (1980s to present) (Table 1) **D** male genital capsule **E** phallus.

***Lepidostoma costale*** (Figure 125) has been found exclusively in August, mostly from small streams of the Lake Superior and Northern Regions. As with *Lepidostoma bryanti*, *Lepidostoma costale* appears locally abundant at Sucker Creek in Clearwater County.

**Figure 126.**
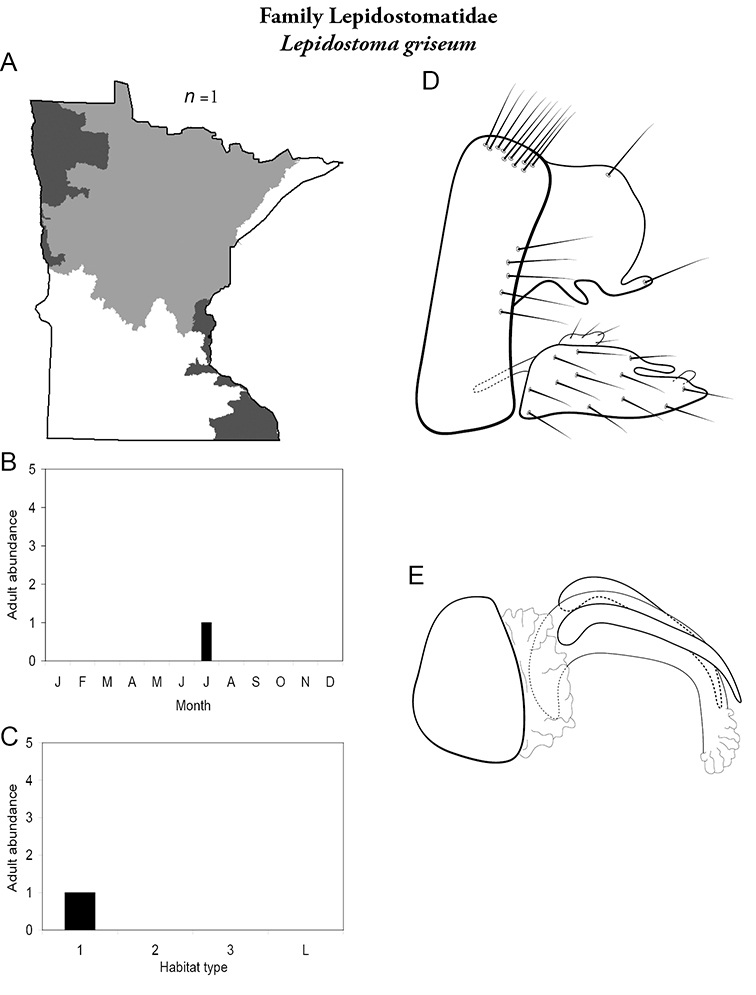
*Lepidostoma griseum* **A** total specimens collected and all known collecting localities (Figure 4) **B** monthly adult abundance (1980s to present) **C** habitat preference (1980s to present) (Table 1) **D** male genital capsule **E** phallus.

***Lepidostoma griseum*** (Figure 126) is known only from a single specimen collected from Mill Creek, William O’Brien State Park, in the Southeastern Region during July 2002.

**Figure 127.**
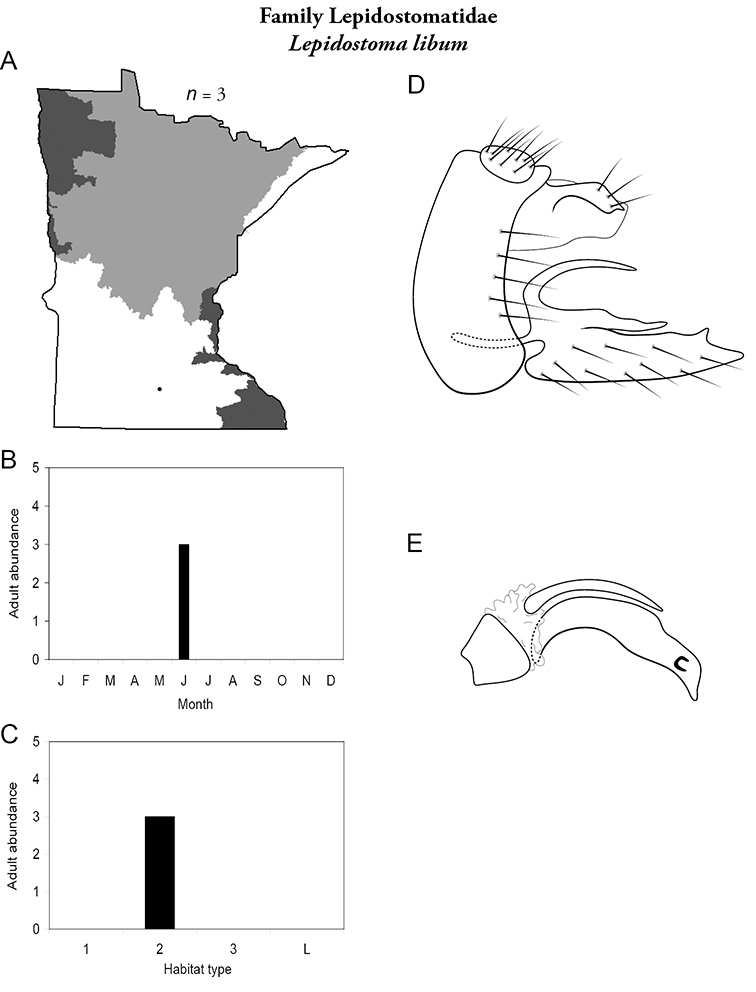
*Lepidostoma libum* **A** total specimens collected and all known collecting localities (Figure 4) **B** monthly adult abundance (1980s to present) **C** habitat preference (1980s to present) (Table 1) **D** male genital capsule **E** phallus.

***Lepidostoma libum*** (Figure 127) is known only from 3 specimens collected from Minneopa Creek, Minneopa State Park, in the Southern Region during June 2000. The species is stenothermic, sensitive to changes in riparian canopy, and typically found in small spring habitats with dense canopy cover (Weaver 1988). Due to the extreme rarity of *Lepidostoma libum* in Minnesota, its sensitivity to habitat disturbance, and the high degree of habitat degradation in southern Minnesota (Houghton 2007), the Minnesota Department of Natural Resources has proposed “Threatened” status for the species (MNDNR 2012).

**Figure 128.**
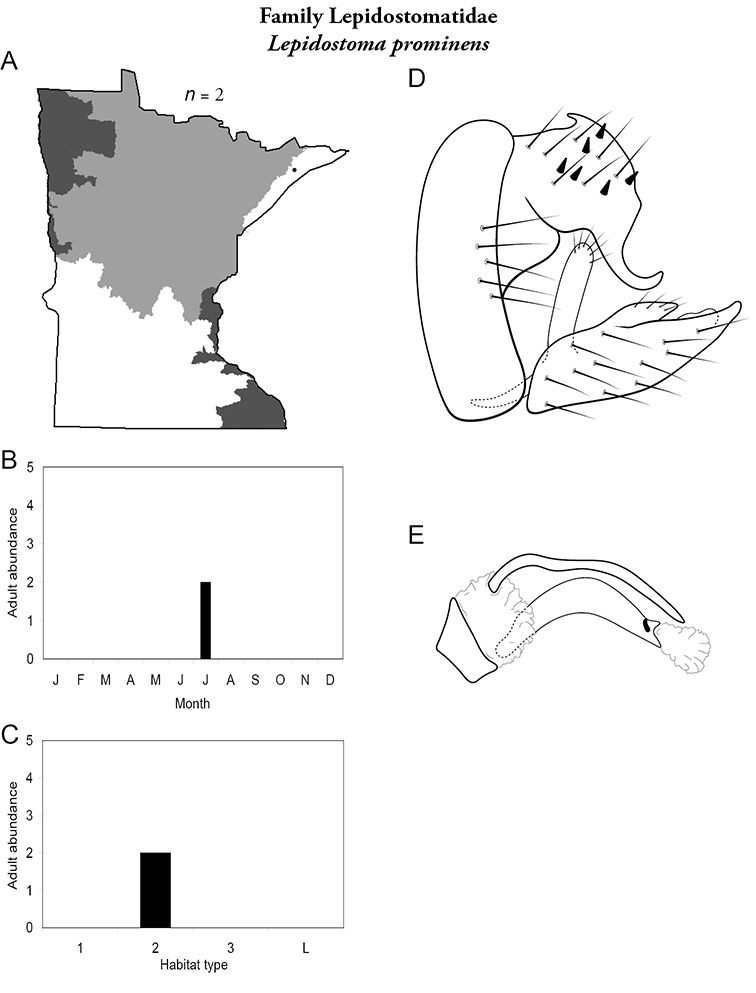
*Lepidostoma prominens* **A** total specimens collected and all known collecting localities (Figure 4) **B** monthly adult abundance (1980s to present) **C** habitat preference (1980s to present) (Table 1) **D** male genital capsule **E** phallus.

***Lepidostoma prominens*** (Figure 128) is known from only 2 specimens collected from the Temperance River, Lake County, in the Lake Superior Region during July 1991.

**Figure 129.**
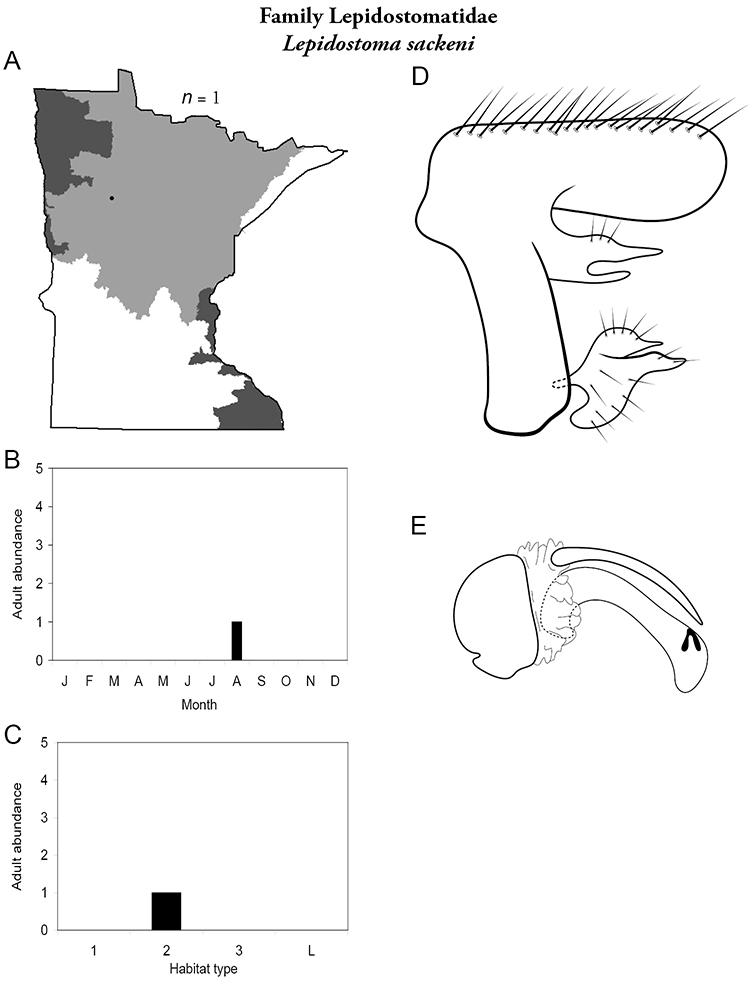
*Lepidostoma sackeni* **A** total specimens collected and all known collecting localities (Figure 4) **B** monthly adult abundance (1980s to present) **C** habitat preference (1980s to present) (Table 1) **D** male genital capsule **E** phallus.

***Lepidostoma sackeni*** (Figure 129) is known only from a single specimen collected from the upper Mississippi River, Clearwater County, in the Northern Region during August 1988.

**Figure 130.**
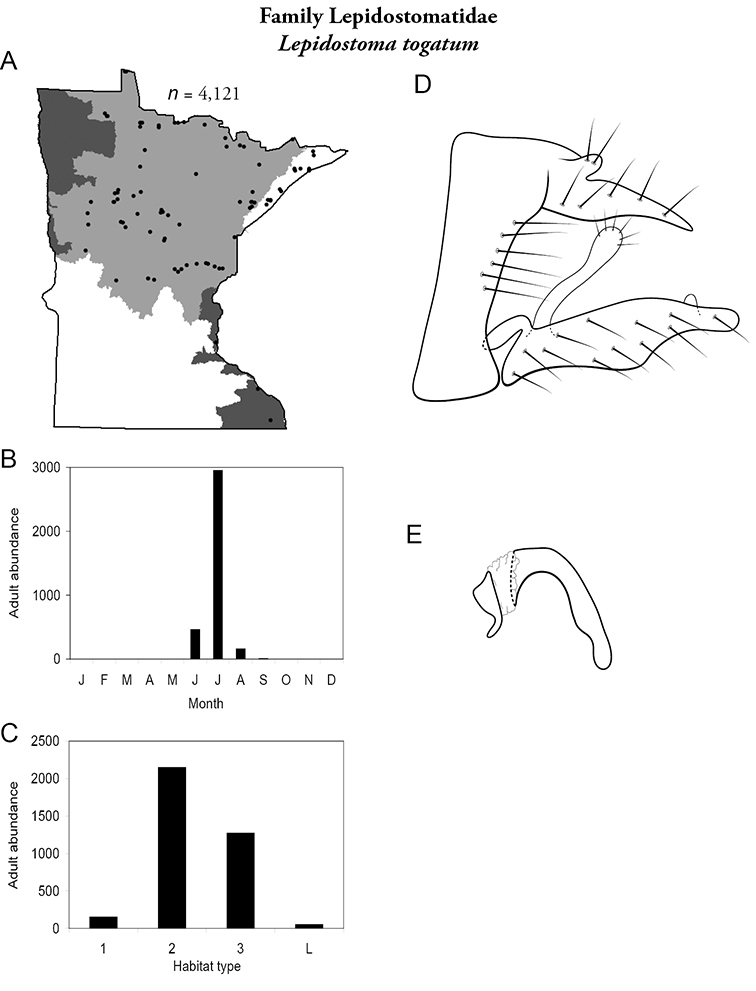
*Lepidostoma togatum* **A** total specimens collected and all known collecting localities (Figure 4) **B** monthly adult abundance (1980s to present) **C** habitat preference (1980s to present) (Table 1) **D** male genital capsule **E** phallus.

***Lepidostoma togatum*** (Figure 130) was, by far, the most common and abundant *Lepidostoma* species. It was found predominantly in medium and large rivers of the Lake Superior and Northern Regions, with some specimens from the Southeastern Region. It was the most abundant species overall in medium rivers of the Lake Superior Region (Table 3). It was most abundant during July, with some specimens in June, August, and September. Houghton (2004b) determined the presence of *Lepidostoma togatum* in medium rivers as one of the best indicators of an undisturbed habitat.

**Figure 131.**
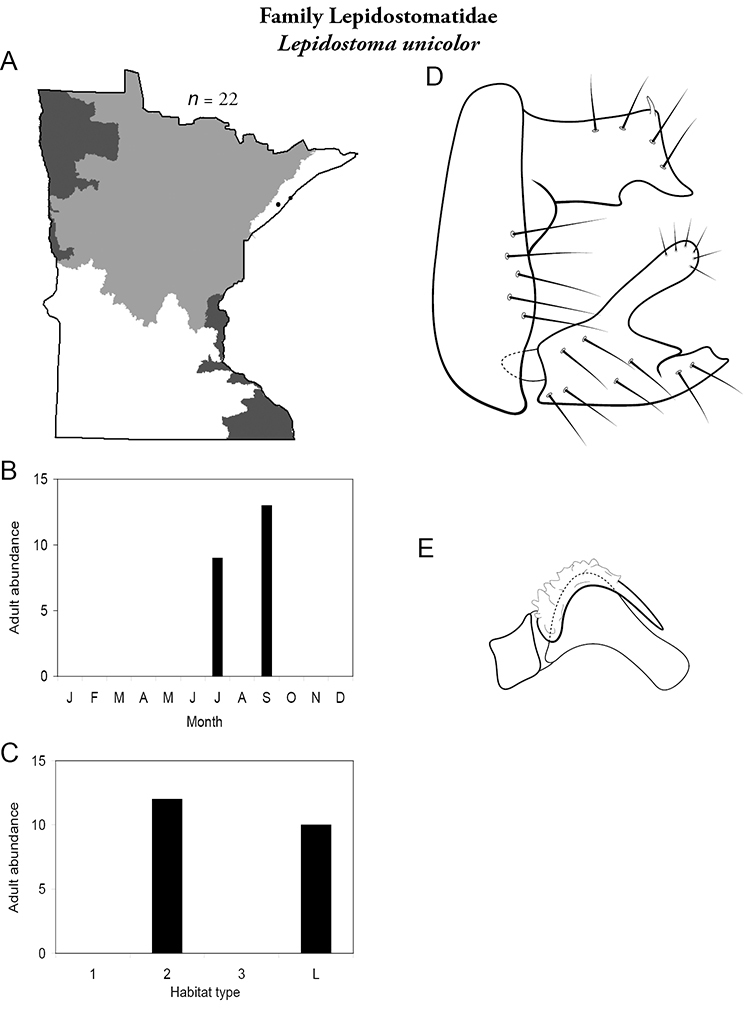
*Lepidostoma unicolor* **A** total specimens collected and all known collecting localities (Figure 4) **B** monthly adult abundance (1980s to present) **C** habitat preference (1980s to present) (Table 1) **D** male genital capsule **E** phallus.

***Lepidostoma unicolor*** (Figure 131) was found exclusively in the Lake Superior Region in July and September. The apparent bivoltine life cycle was probably caused by a lack of collecting in August. Specimens were collected from medium streams, including one as it entered into Lake Superior.

### Family Leptoceridae

This family contains 8 genera in Minnesota: *Ceraclea*, *Leptocerus*, *Mystacides*, *Nectopsyche*, *Oecetis*, *Setodes*, *Triaenodes*, and *Ylodes*, and a total of 46 species. It is the 3rd most species-rich family (Figure 6).

Larvae construct tubular portable cases usually composed of mineral fragments. They are generally more abundant in lakes, although many individual species are more abundant in streams. Larvae are usually either gathering collectors or shredders; some are predators. Adults range 8–20 mm in length. Most are tan or brown in color, although specimens of *Nectopsyche* can be bright white, and those of *Mystacides sepulchralis* are jet black. All adults have antennae >2× longer than their bodies, a useful familial identification characteristic if antennae are intact.

Adults of many leptocerid species were extremely abundant in light traps throughout the state. In fact, of the 10 most abundant species in Minnesota, 6 were leptocerids (Figure 9). Moreover, the top 6 most widespread species overall in the state were all leptocerids (Figure 8).

### Genus *Ceraclea*

The genus *Ceraclea* contains 16 species in Minnesota. It is the 5th most species-rich genus (Figure 7). It contains several species in or near the top 10 of most abundant and widespread species in the state (Figures 8–9). Larvae usually feed on detritus, although some are predatory on freshwater sponges (Resh et al. 1976). Cases are typically composed of small mineral particles and feature a central tube with overhanging dorsal and lateral flanges (Wiggins 1996). Adults are usually 12–18 mm in length, although females are occasionally smaller than that. Wings of adults are usually tan or brown in color, sometimes with lighter reticulations or bands (Figure 292). For additional species, see Morse (1975).

**Figure 132.**
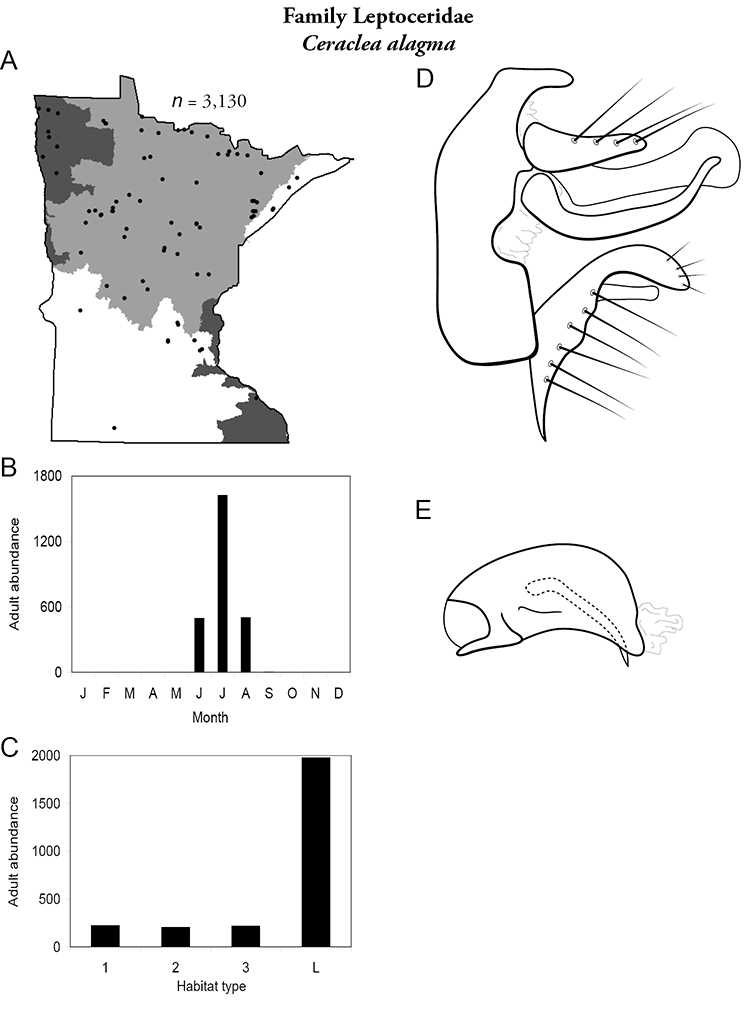
*Ceraclea alagma* **A** total specimens collected and all known collecting localities (Figure 4) **B** monthly adult abundance (1980s to present) **C** habitat preference (1980s to present) (Table 1) **D** male genital capsule **E** phallus.

***Ceraclea alagma*** (Figure 132) was abundant in lakes of all regions, and found June through August. It was the 2nd most abundant species in lakes of the Northwestern Region, and 3rd most abundant in lakes of the Southeastern Region (Table 5–6).

**Figure 133.**
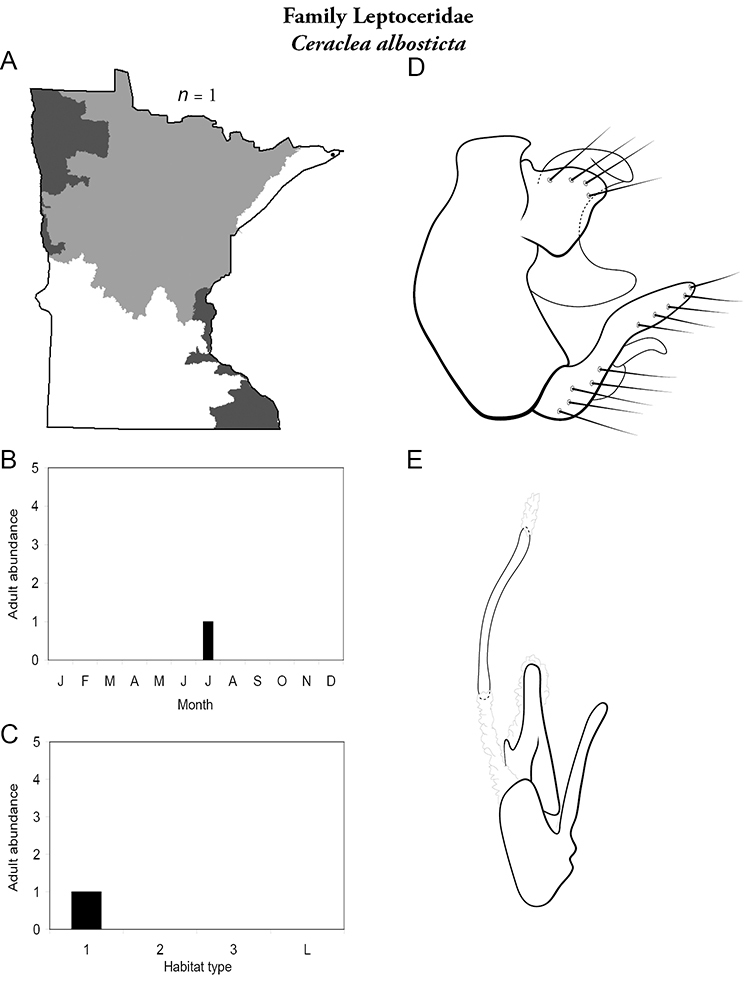
*Ceraclea albosticta* **A** total specimens collected and all known collecting localities (Figure 4) **B** monthly adult abundance (1980s to present) **C** habitat preference (1980s to present) (Table 1) **D** male genital capsule **E** phallus (rotated 90 degrees counter-clockwise).

***Ceraclea albosticta*** (Figure 133) is known only from a single specimen collected in July 2000 from an unnamed spring near Grand Portage National Monument in the Lake Superior Region.

**Figure 134.**
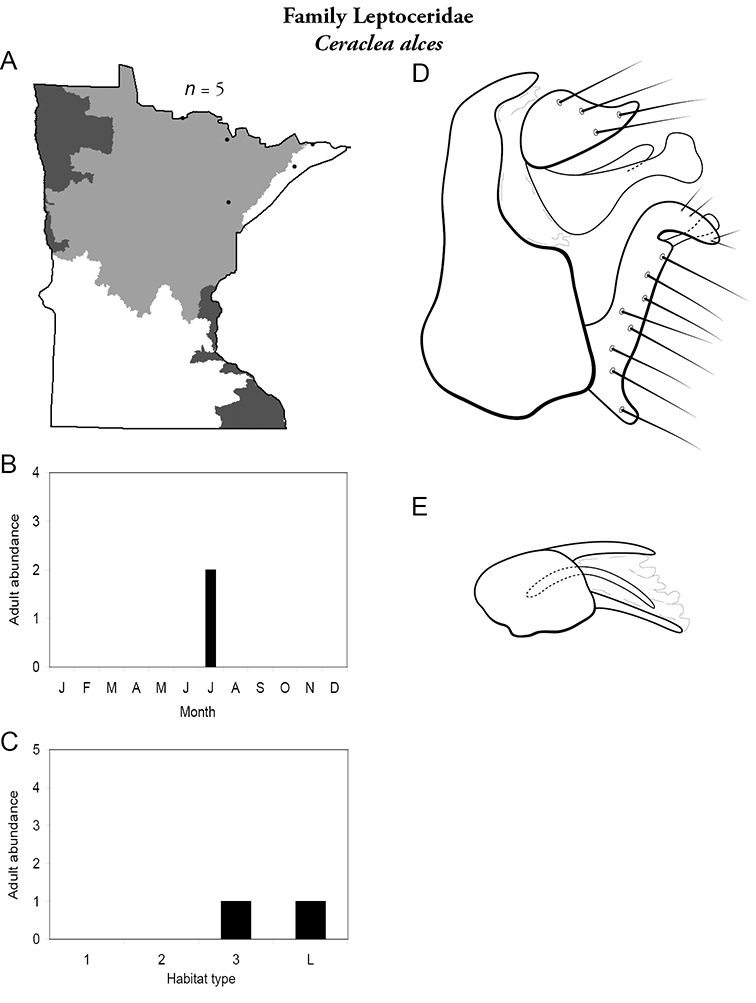
*Ceraclea alces* **A** total specimens collected and all known collecting localities (Figure 4) **B** monthly adult abundance (1980s to present) **C** habitat preference (1980s to present) (Table 1) **D** male genital capsule **E** phallus.

***Ceraclea alces*** (Figure 134) has been collected during July from lakes and large rivers of the Lake Superior and Northern Regions. Only 2 specimens have been collected since the 1950s.

**Figure 135.**
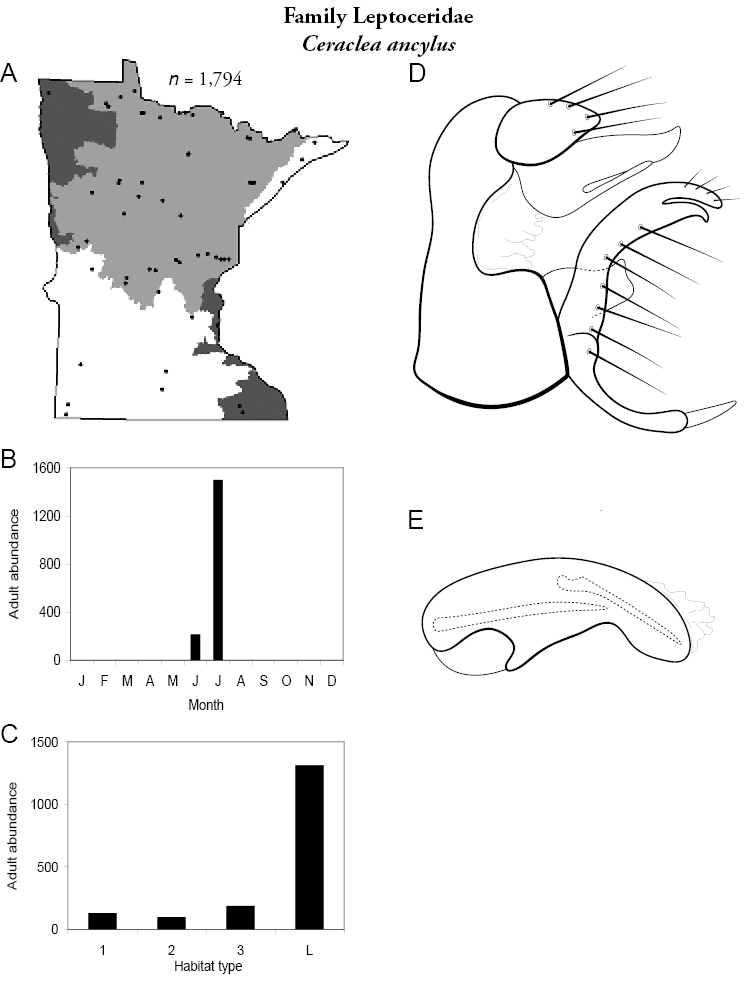
*Ceraclea ancylus* **A** total specimens collected and all known collecting localities (Figure 4) **B** monthly adult abundance (1980s to present) **C** habitat preference (1980s to present) (Table 1) **D** male genital capsule **E** phallus.

***Ceraclea ancylus*** (Figure 135) was found in all regions throughout the state, mostly from lakes. Adults were present in June and, especially, July.

**Figure 136.**
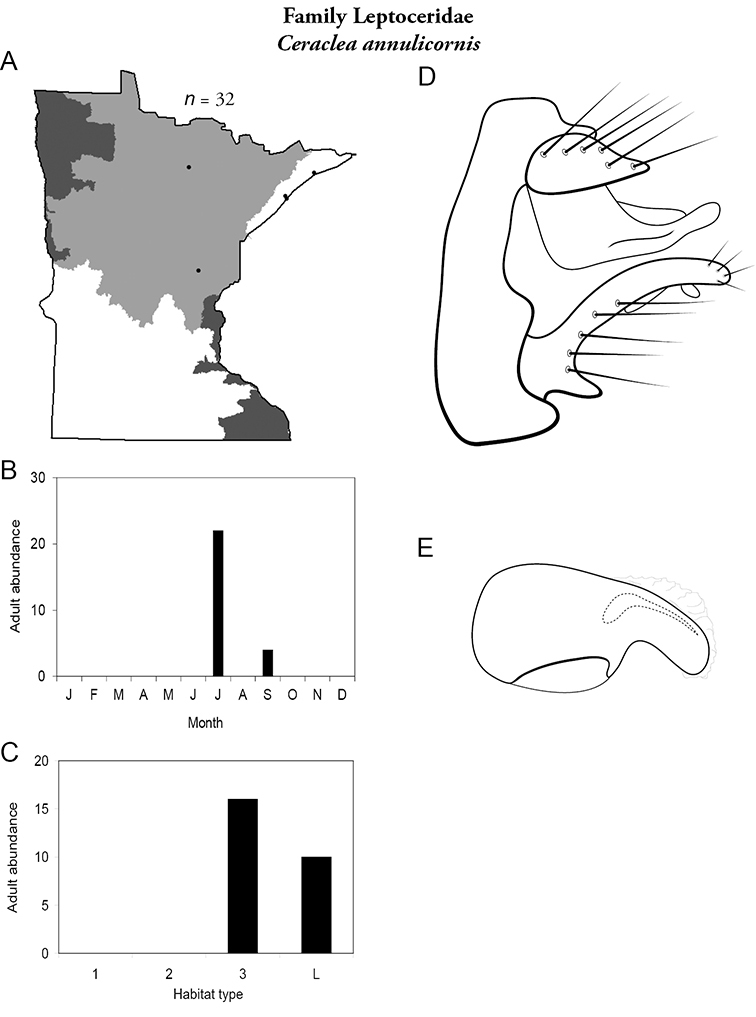
*Ceraclea annulicornis* **A** total specimens collected and all known collecting localities (Figure 4) **B** monthly adult abundance (1980s to present) **C** habitat preference (1980s to present) (Table 1) **D** male genital capsule **E** phallus.

***Ceraclea annulicornis*** (Figure 136) has been found in lakes and large rivers of the Lake Superior and Northern Regions. Adults were present in July and September, which probably reflected a lack of collecting in August.

**Figure 137.**
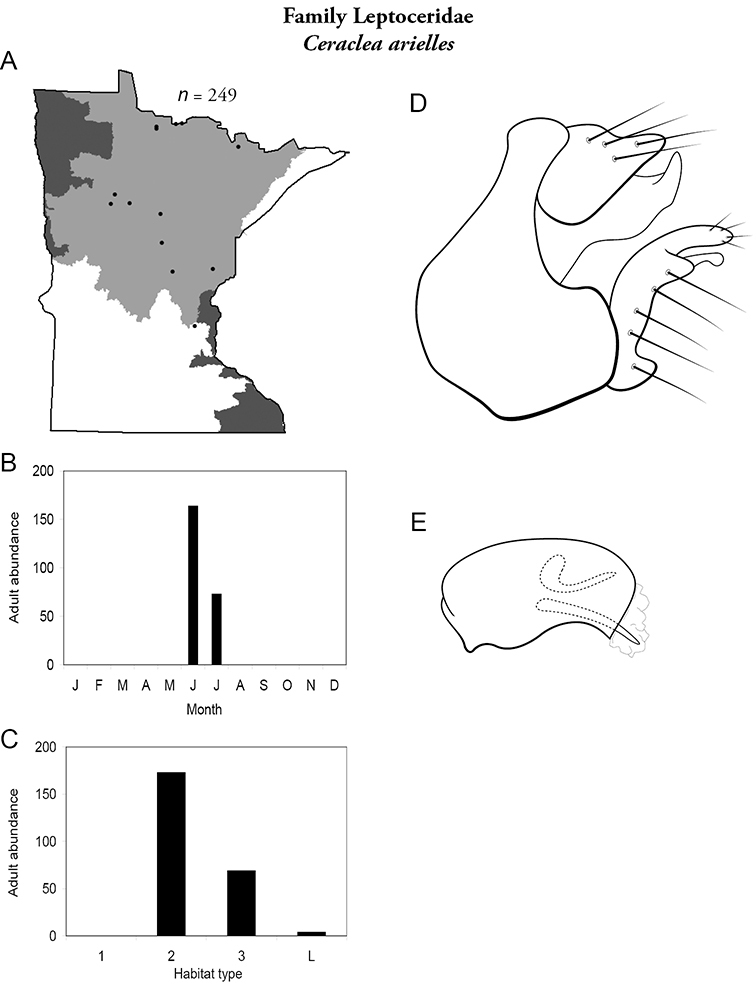
*Ceraclea arielles* **A** total specimens collected and all known collecting localities (Figure 4) **B** monthly adult abundance (1980s to present) **C** habitat preference (1980s to present) (Table 1) **D** male genital capsule **E** phallus.

***Ceraclea arielles*** (Figure 137) is known only from or near the Northern Region. It was found predominantly in medium and large rivers during June and July.

**Figure 138.**
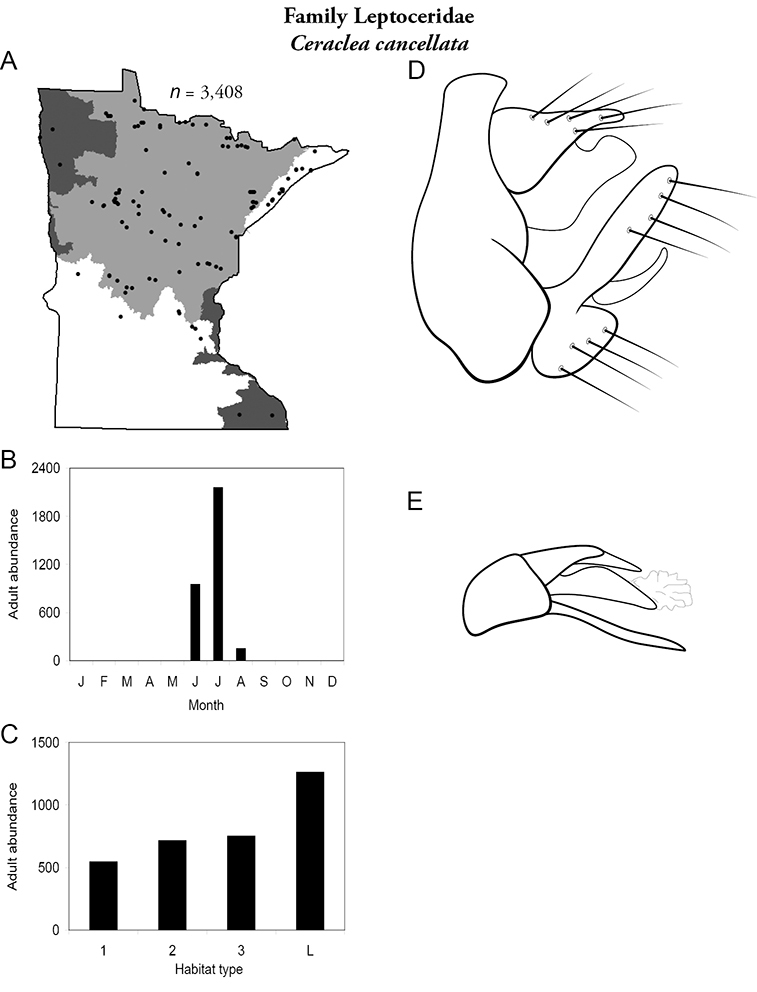
*Ceraclea cancellata* **A** total specimens collected and all known collecting localities (Figure 4) **B** monthly adult abundance (1980s to present) **C** habitat preference (1980s to present) (Table 1) **D** male genital capsule **E** phallus.

***Ceraclea cancellata*** (Figure 138) has historically been found in all regions. Since the 1950s, however, it has been collected predominantly in the Lake Superior and Northern Regions. Overall, it was the 10th most widespread species in the state (Figure 8). It was found in all types of streams, especially medium rivers of the Lake Superior Region (Table 3). Statewide, it was most abundant in lakes. Adults were present from June through August. The presence of this species in medium rivers was determined by Houghton (2004b) as one of the best indicators of an undisturbed habitat.

**Figure 139.**
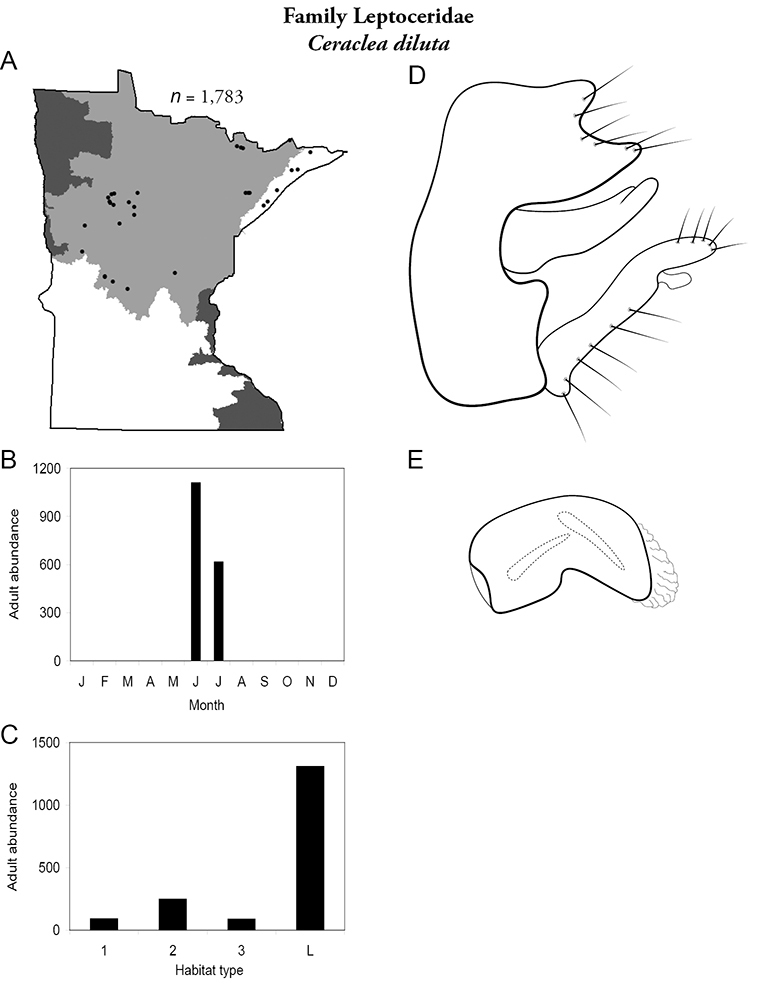
*Ceraclea diluta* **A** total specimens collected and all known collecting localities (Figure 4) **B** monthly adult abundance (1980s to present) **C** habitat preference (1980s to present) (Table 1) **D** male genital capsule **E** phallus.

***Ceraclea diluta*** (Figure 139) is known from the Lake Superior and Northern Regions, primarily in lakes. Adults were present during June and July.

**Figure 140.**
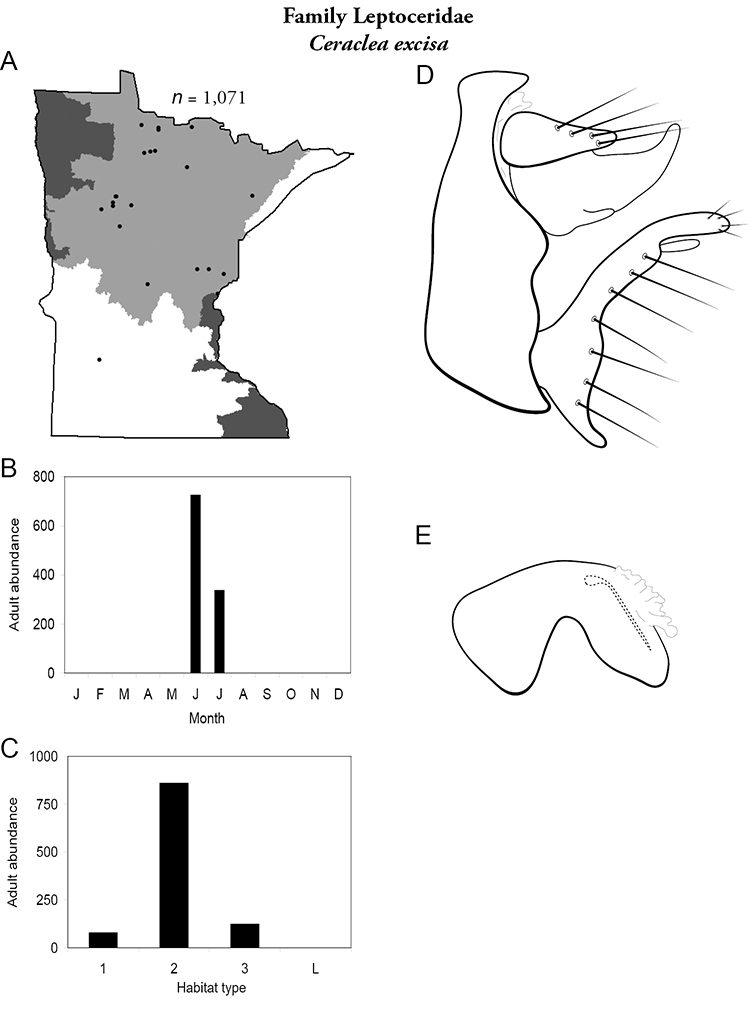
*Ceraclea excisa* **A** total specimens collected and all known collecting localities (Figure 4) **B** monthly adult abundance (1980s to present) **C** habitat preference (1980s to present) (Table 1) **D** male genital capsule **E** phallus.

***Ceraclea excisa*** (Figure 140) was collected in June and July, predominantly in the Northern Region. It was only found in streams, especially medium streams.

**Figure 141.**
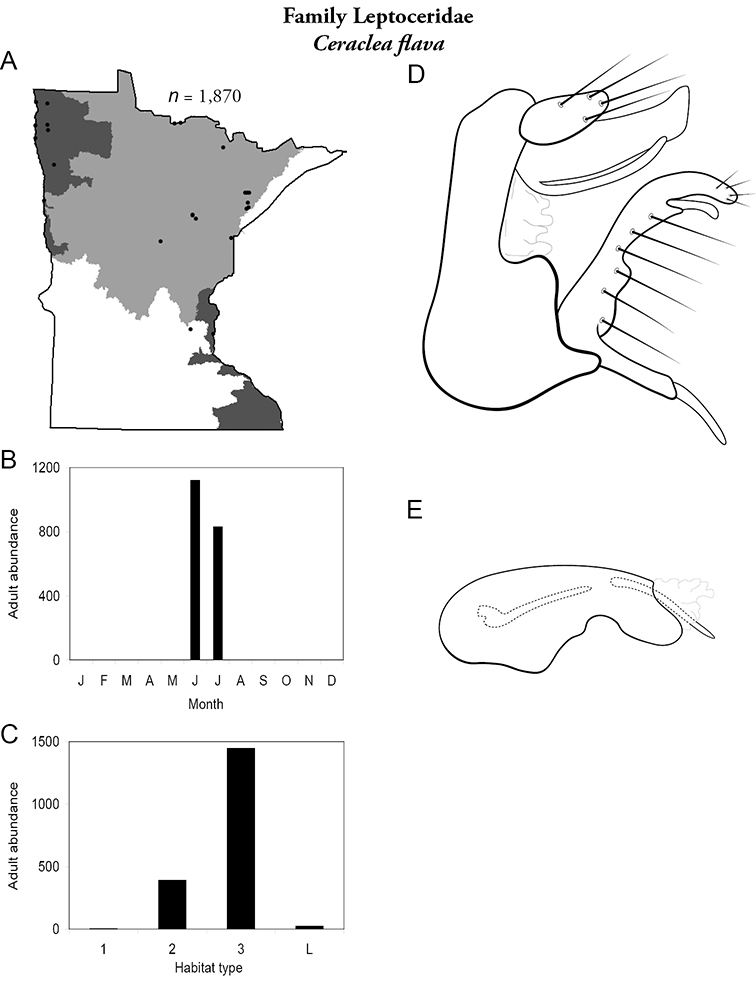
*Ceraclea flava* **A** total specimens collected and all known collecting localities (Figure 4) **B** monthly adult abundance (1980s to present) **C** habitat preference (1980s to present) (Table 1) **D** male genital capsule **E** phallus.

***Ceraclea flava*** (Figure 141) was collected mostly in the Northern and Northwestern Regions. It was found in streams, particularly larger rivers, during June and July.

**Figure 142.**
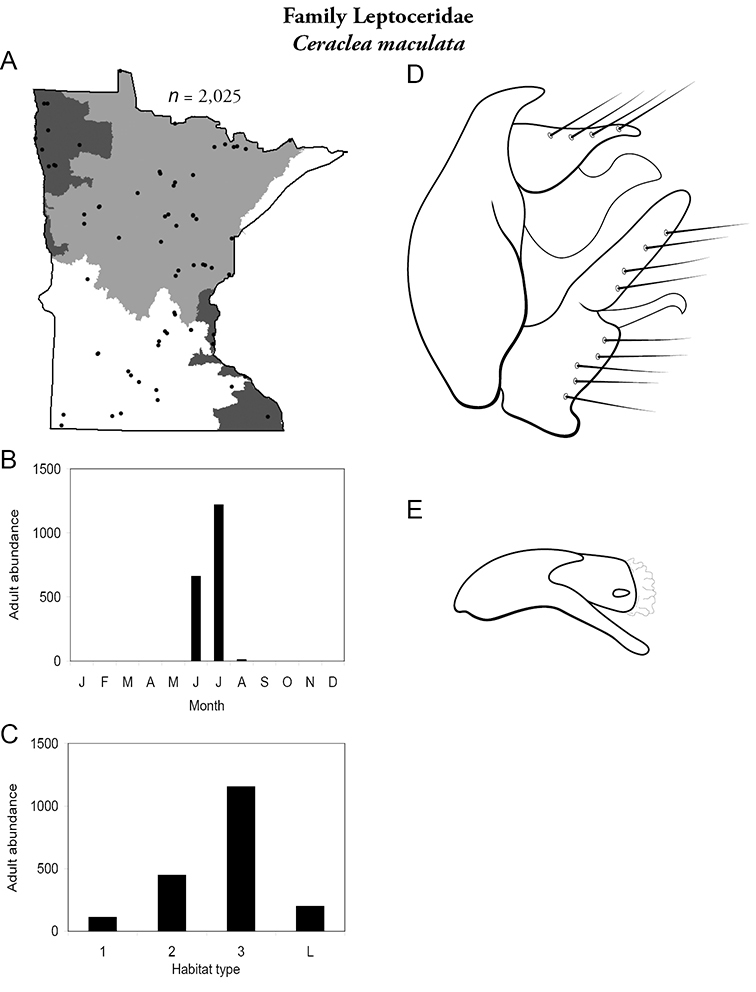
*Ceraclea maculata* **A** total specimens collected and all known collecting localities (Figure 4) **B** monthly adult abundance (1980s to present) **C** habitat preference (1980s to present) (Table 1) **D** male genital capsule **E** phallus.

***Ceraclea maculata*** (Figure 142) is known from all regions except the Lake Superior, with adults present mostly in June and July. It was collected mainly from streams, especially large rivers.

**Figure 143.**
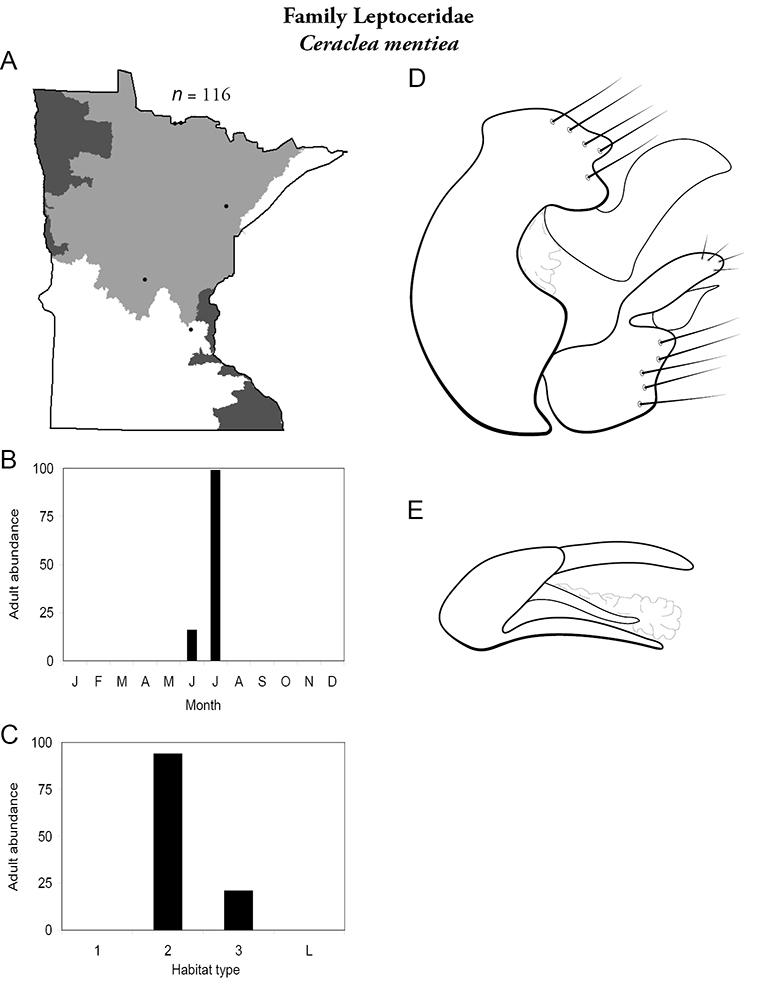
*Ceraclea mentiea* **A** total specimens collected and all known collecting localities (Figure 4) **B** monthly adult abundance (1980s to present) **C** habitat preference (1980s to present) (Table 1) **D** male genital capsule **E** phallus.

***Ceraclea mentiea*** (Figure 143) was collected exclusively from medium and large rivers in or near the Northern Region during June and July.

**Figure 144.**
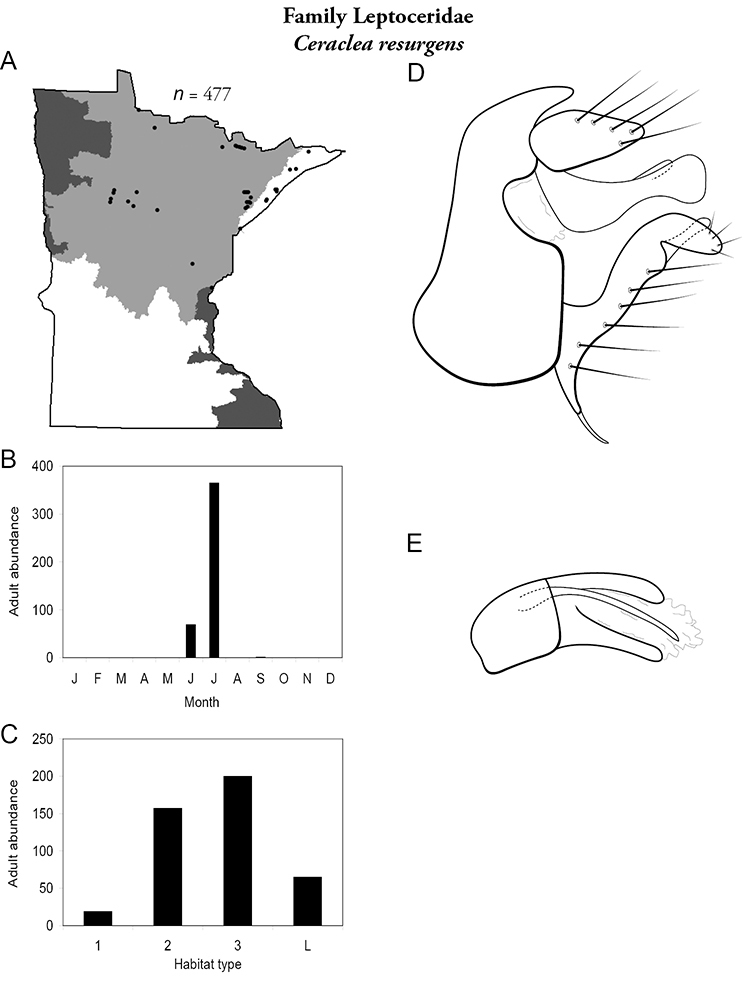
*Ceraclea resurgens* **A** total specimens collected and all known collecting localities (Figure 4) **B** monthly adult abundance (1980s to present) **C** habitat preference (1980s to present) (Table 1) **D** male genital capsule **E** phallus.

***Ceraclea resurgens*** (Figure 144) is known only from the Northern Region. It was most abundant in medium and large rivers during July, with some adults present in June.

**Figure 145.**
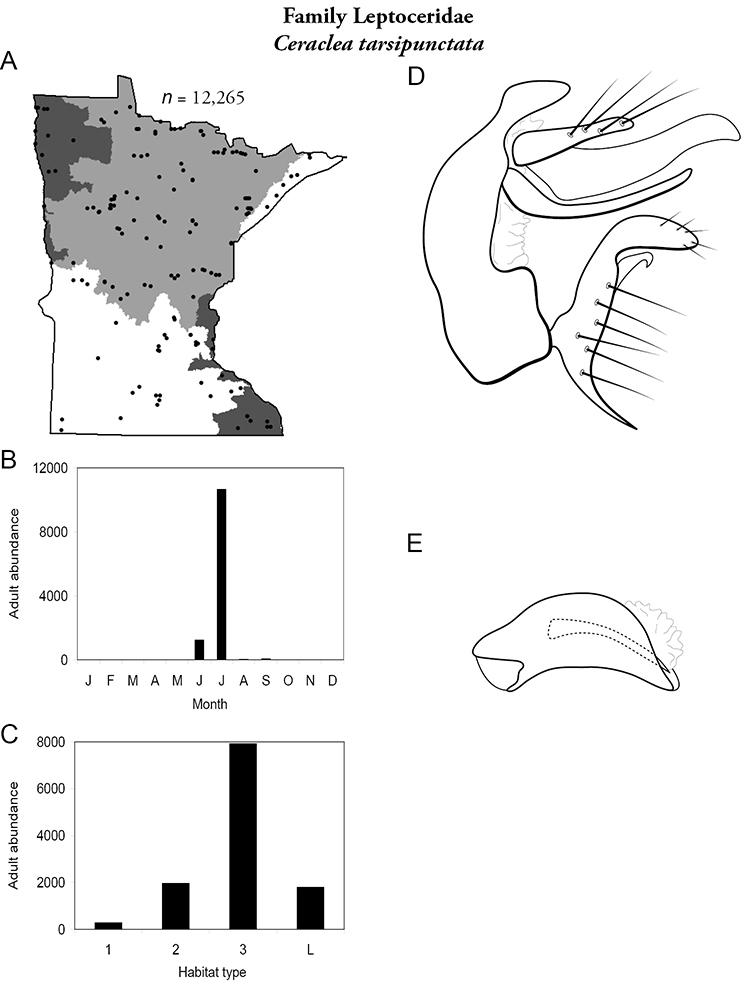
*Ceraclea tarsipunctata* **A** total specimens collected and all known collecting localities (Figure 4) **B** monthly adult abundance (1980s to present) **C** habitat preference (1980s to present) (Table 1) **D** male genital capsule **E** phallus.

***Ceraclea tarsipunctata*** (Figure 145) was abundant in all habitats except small streams, and was especially abundant in large rivers. It was widespread in all regions throughout the state and was the 4th most widespread species overall (Figure 8). It was also the 7th most abundant species in the state (Figure 9). It was the single most abundant species in both medium and large rivers of the Southeastern Region (Table 6). Adults were most abundant in July, with some specimens present in June, August, and September.

**Figure 146.**
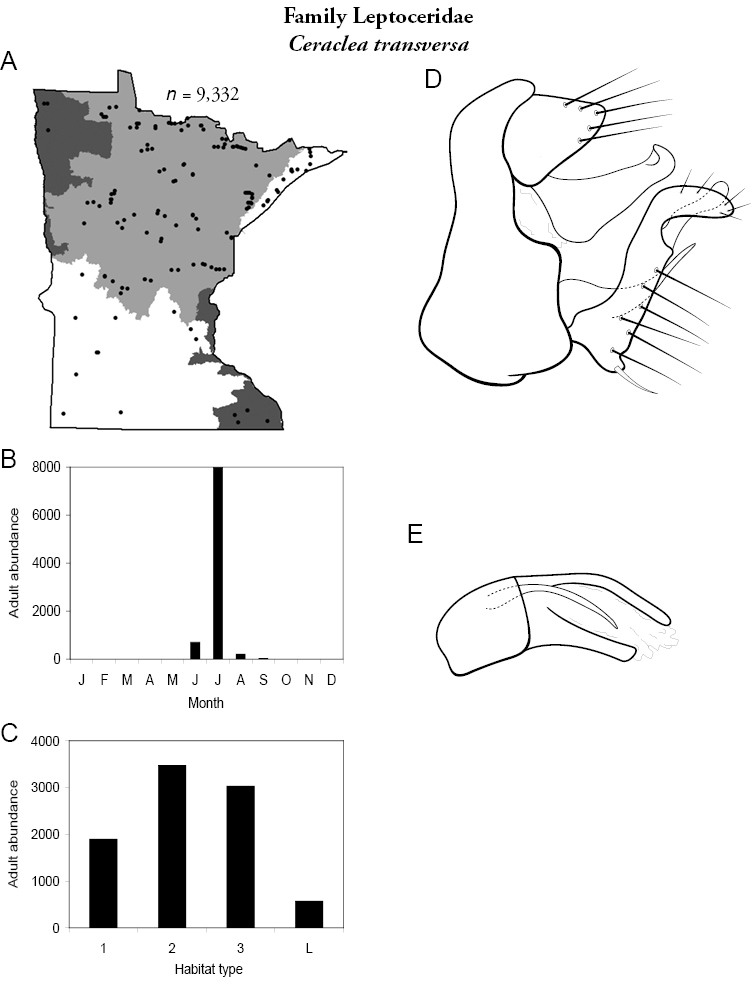
*Ceraclea transversa* **A** total specimens collected and all known collecting localities (Figure 4) **B** monthly adult abundance (1980s to present) **C** habitat preference (1980s to present) (Table 1) **D** male genital capsule **E** phallus.

***Ceraclea transversa*** (Figure 146) was historically found in all regions. Since the 1950s, however, it has been collected commonly in the Lake Superior and Northern Regions and found only sporadically elsewhere. It was abundant in all sizes of streams, especially medium and large rivers. It was the most and 2nd most abundant species in large and medium rivers, respectively, of the Lake Superior Region (Table 3). Overall, it was the 5th most widespread species and the 8th most abundant species in the state (Figure 8–9). Adults were most abundant in July, with some specimens present in June, August, and September. Its presence in medium rivers was determined by Houghton (2004b) as one of the best indicators of an undisturbed habitat.

**Figure 147.**
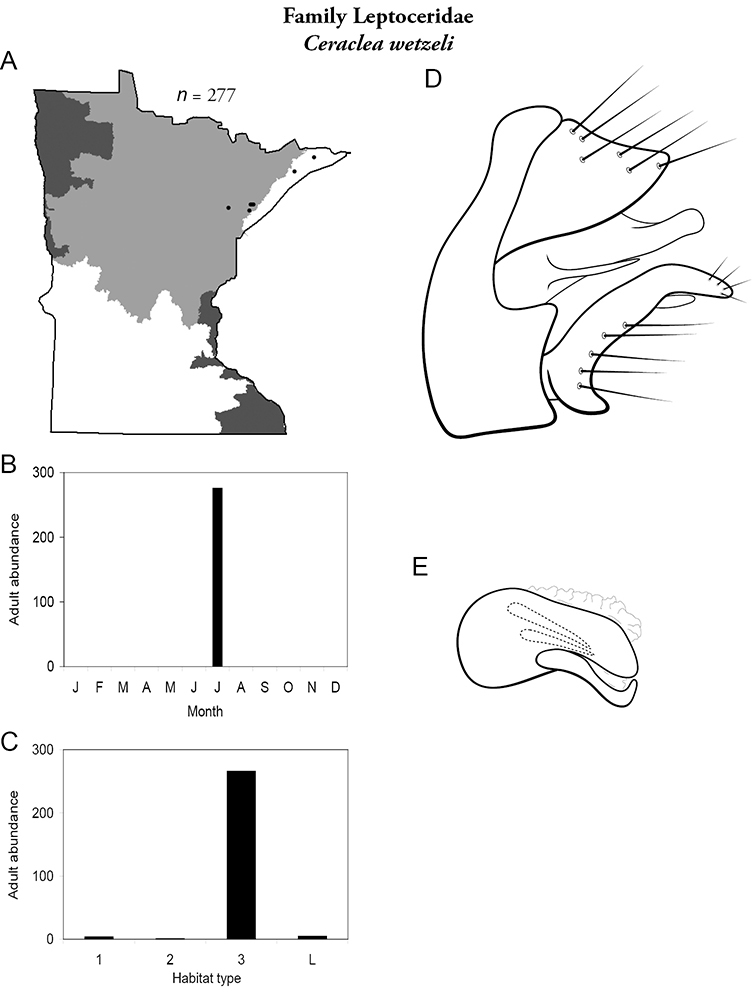
*Ceraclea wetzeli* **A** total specimens collected and all known collecting localities (Figure 4) **B** monthly adult abundance (1980s to present) **C** habitat preference (1980s to present) (Table 1) **D** male genital capsule **E** phallus.

***Ceraclea wetzeli*** (Figure 147) is known only from the Lake Superior and Northern Regions. Adults were only collected in July, almost exclusively from large rivers.

Another *Ceraclea* species, *Ceraclea vertreesi*, has been previously reported from Minnesota from a series of collections made in 1989 in and around Lake Itasca State Park in the Northern Region (Monson 1997). The species is typically found in high-gradient montane habitats of the western U.S. Not only are the known Minnesota habitats low gradient and thus atypical for the species, but they are separated from other known populations by nearly 1000 km. Furthermore, *Ceraclea vertreesi* is very similar in appearance to *Ceraclea resurgens*, a fairly common species in northern Minnesota. A thorough examination of “*Ceraclea vertreesi*” and *Ceraclea resurgens* specimens from Minnesota suggests that all Minnesota populations of “*Ceraclea vertreesi*” are likely *Ceraclea resurgens*. This taxonomic confusion has caused the Minnesota Department of Natural Resources to propose removal of *Ceraclea vertreesi* from its list of protected species (MNDNR 2012). Thus, it is not included in this manual.

Another *Ceraclea* species, *Ceraclea brevis*, is known worldwide from only a single specimen collected in 1965 from an unknown locality in Crow Wing County in the Northern Region (Etnier 1968). Despite extensive recent collecting in this general area, the species has yet to be rediscovered. Moreover, *Ceraclea brevis* is very similar in appearance to *Ceraclea tarsipunctata*, a very common species in Minnesota. Thorough examination of the only known “*Ceraclea brevis*” specimen suggests that it may, in fact, be an aberrant specimen of *Ceraclea tarsipunctata*. This taxonomic confusion has caused the Minnesota Department of Natural Resources to propose removal of *Ceraclea brevis* from its list of protected species (MNDNR 2012). Thus, it is not included in this manual.

Two other *Ceraclea* species: *Ceraclea neffi* and *Ceraclea nepha*, have been reported from Minnesota based on larval records (Lager et al. 1979). Adult specimens of neither species have been found in the state. The former species is restricted to Kentucky and Virginia (Morse 1975), and is very unlikely to occur in Minnesota. The latter species is known from central U.S. Without specimens to verify presence, however, neither species is included in this manual.

### Genus *Leptocerus*

The genus *Leptocerus* contains a single species in North America and in Minnesota. Larvae typically inhabit lakes and slow-moving areas of streams, and can actively swim through beds of aquatic plants. They appear to be generalist feeders on organic particles. Cases are composed of silk only (Wiggins 1996). Adults are very slender and light brown in color (Figure 291). Total length usually ranges 8–10 mm.

**Figure 148.**
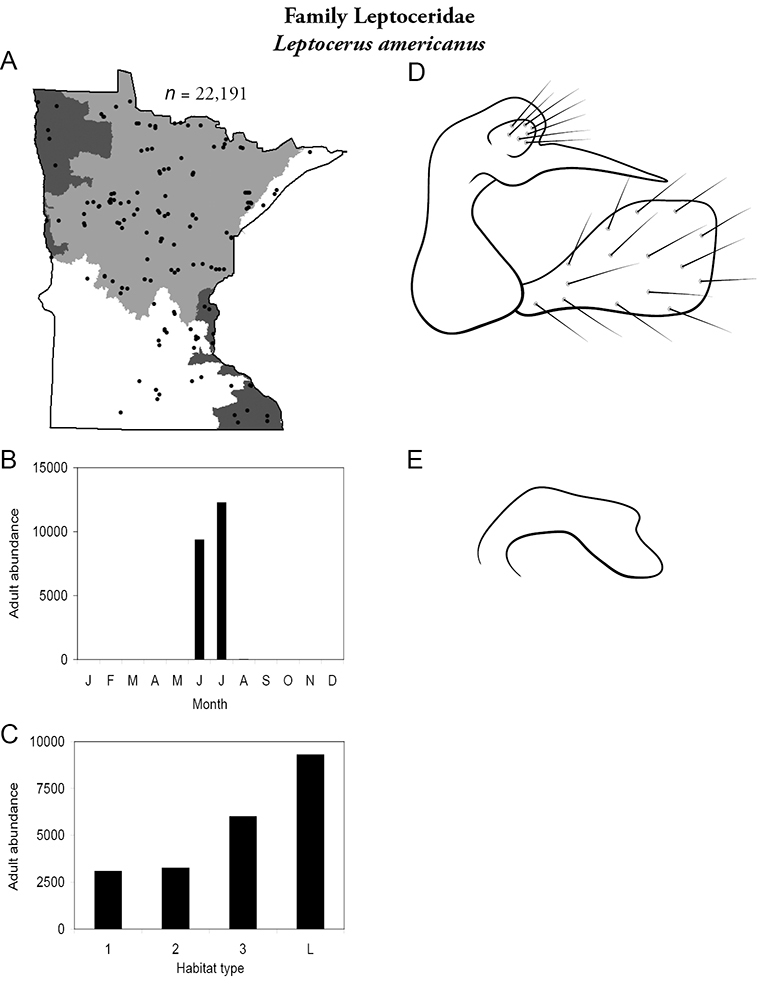
*Leptocerus americanus* **A** total specimens collected and all known collecting localities (Figure 4) **B** monthly adult abundance (1980s to present) **C** habitat preference (1980s to present) (Table 1) **D** male genital capsule **E** phallus.

***Leptocerus americanus*** (Figure 148) has been commonly collected throughout all regions and was the 6th most widespread species overall in the state (Figure 8). It was also the 2nd most abundant species overall in the state (Figure 9), and was abundant in all habitat types, especially lakes. It was the most abundant species in lakes and small streams of the Northern Region (Table 4). It was also the most abundant species in lakes of the Southern Region, and the 2nd most abundant species in lakes of the Southeastern Region (Table 6). Adults were found during June and July.

### Genus *Mystacides*

The genus *Mystacides* contains 2 species in Minnesota. For additional species, see Yamamoto and Wiggins (1964) or Morse and Yang (2002). Larvae are usually found in lakes or slow-moving areas of streams and may consume both plants and animals (Lloyd 1921, Wiggins 1996). Cases are composed primarily of small mineral fragments, usually with longer twigs or conifer needles on their lateral margins. Adults of both species range 8–10 mm in length. Superficially, the 2 species look nothing alike. The forewings of *Mystacides interjecta* are orange with a black banding pattern; whereas, *Mystacides sepulchralis* is jet black (Figure 291). Males and females of both species can be easily identified without a microscope simply by their wing color and pattern.

**Figure 149.**
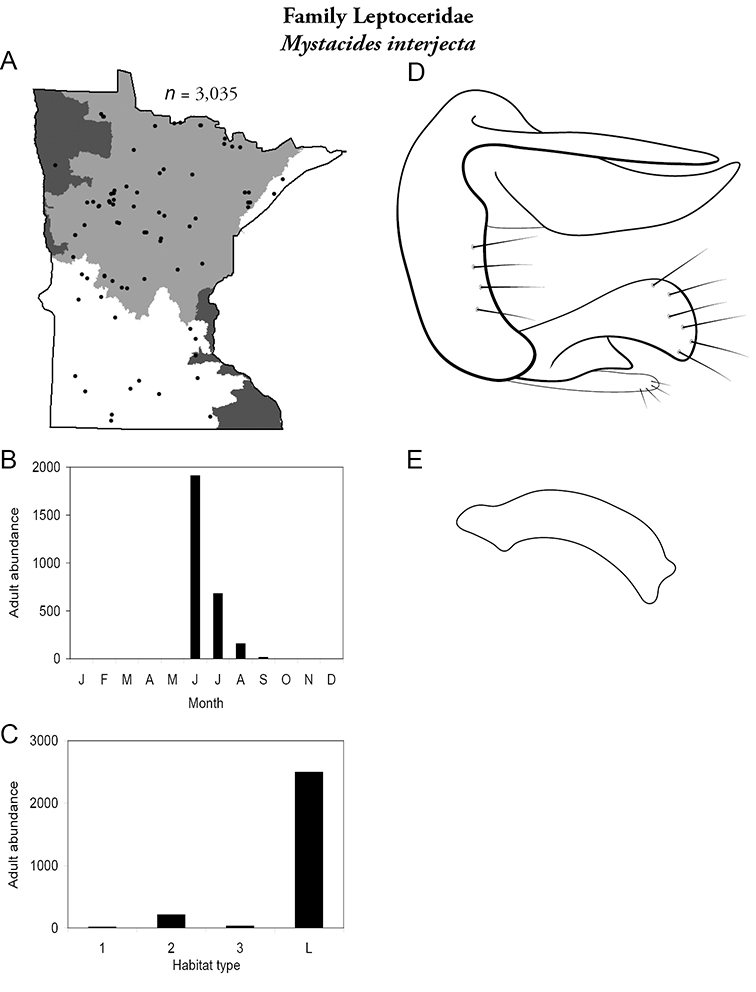
*Mystacides interjecta* **A** total specimens collected and all known collecting localities (Figure 4) **B** monthly adult abundance (1980s to present) **C** habitat preference (1980s to present) (Table 1) **D** male genital capsule **E** phallus.

***Mystacides interjecta*** (Figure 149) has been collected predominantly from lakes of the Northern and Southern Regions, with sporadic records in other regions and habitat types. Adults were most abundant in June, decreasing in abundance through September.

**Figure 150.**
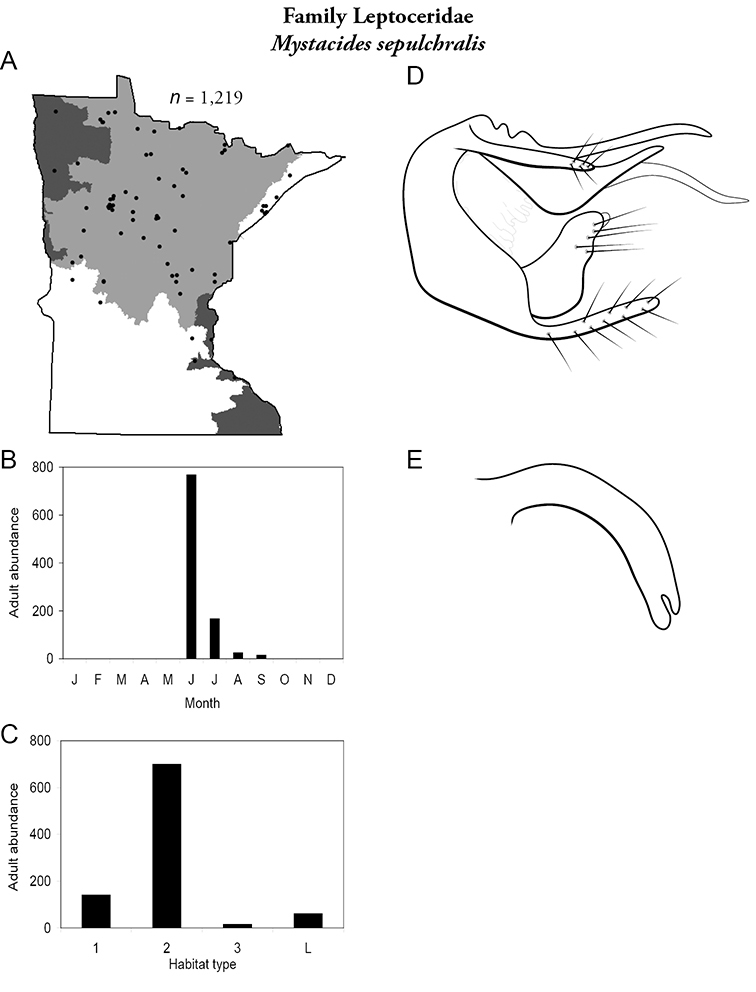
*Mystacides sepulchralis* **A** total specimens collected and all known collecting localities (Figure 4) **B** monthly adult abundance (1980s to present) **C** habitat preference (1980s to present) (Table 1) **D** male genital capsule **E** phallus.

***Mystacides sepulchralis*** (Figure 150) has a similar distribution and flight period as *Mystacides interjecta*, but was more abundant in medium streams and less abundant in the Southern Region.

### Genus *Nectopsyche*

The genus *Nectopsyche* contains 4 species in Minnesota. For additional species, see Haddock (1977). Larvae are typically found in slow moving areas of larger rivers and may feed on both plants and animals (Wiggins 1996). Cases are long and slender, and composed entirely of small mineral particles. Adults are slender and range in size from *Nectopsyche pavida* (8–10 mm) to *Nectopsyche exquisita* (15–18 mm). They are typically yellow to bright white in color, with various patterns of black setae on the forewings (Figure 291).

**Figure 151.**
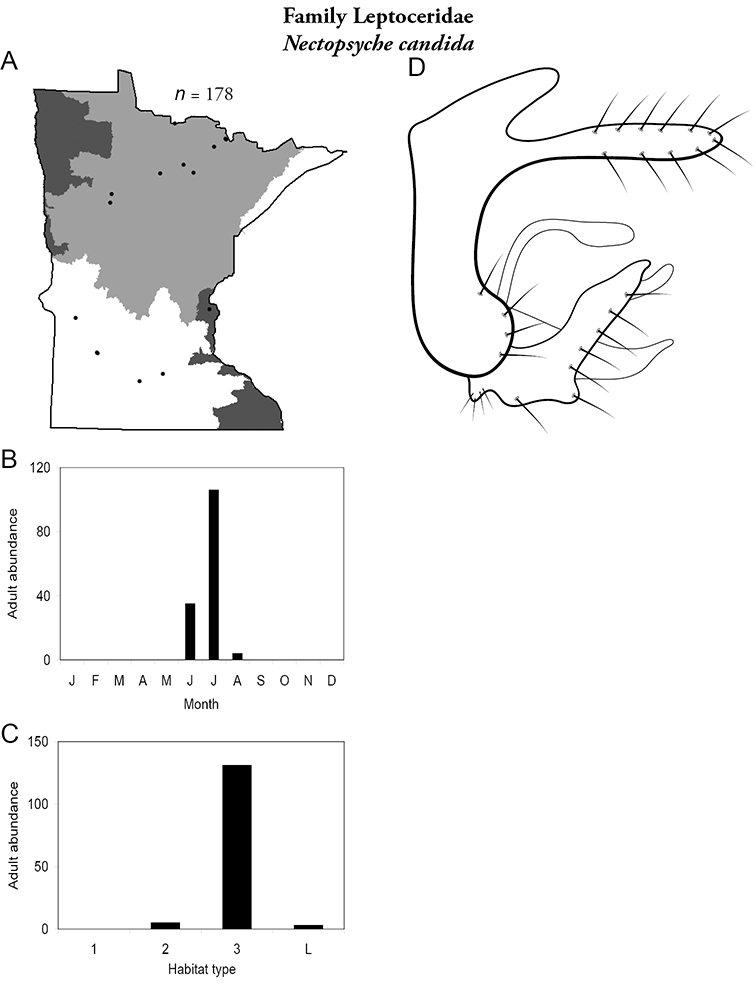
*Nectopsyche candida* **A** total specimens collected and all known collecting localities (Figure 4) **B** monthly adult abundance (1980s to present) **C** habitat preference (1980s to present) (Table 1) **D** male genital capsule.

***Nectopsyche candida*** (Figure 151) was collected mainly from the Northern and Southern Regions, mostly from large rivers in June and July, with a few specimens in August.

**Figure 152.**
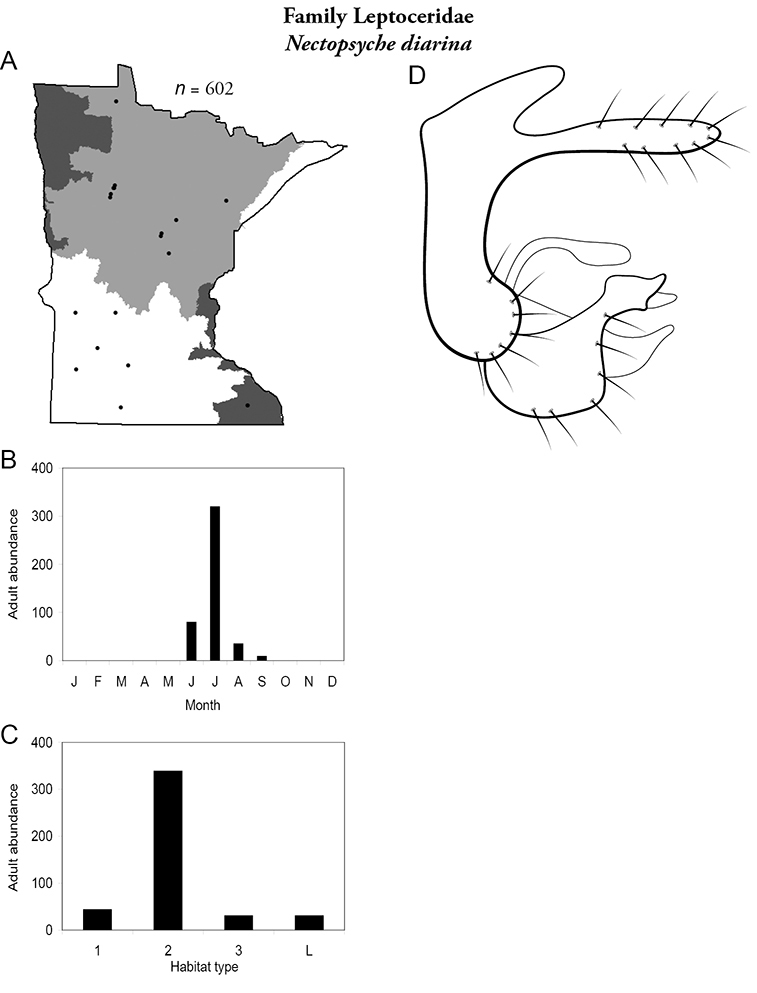
*Nectopsyche diarina* **A** total specimens collected and all known collecting localities (Figure 4) **B** monthly adult abundance (1980s to present) **C** habitat preference (1980s to present) (Table 1) **D** male genital capsule.

***Nectopsyche diarina*** (Figure 152) had a similar general distribution and flight period as *Nectopsyche candida*, but was more common in medium rivers.

**Figure 153.**
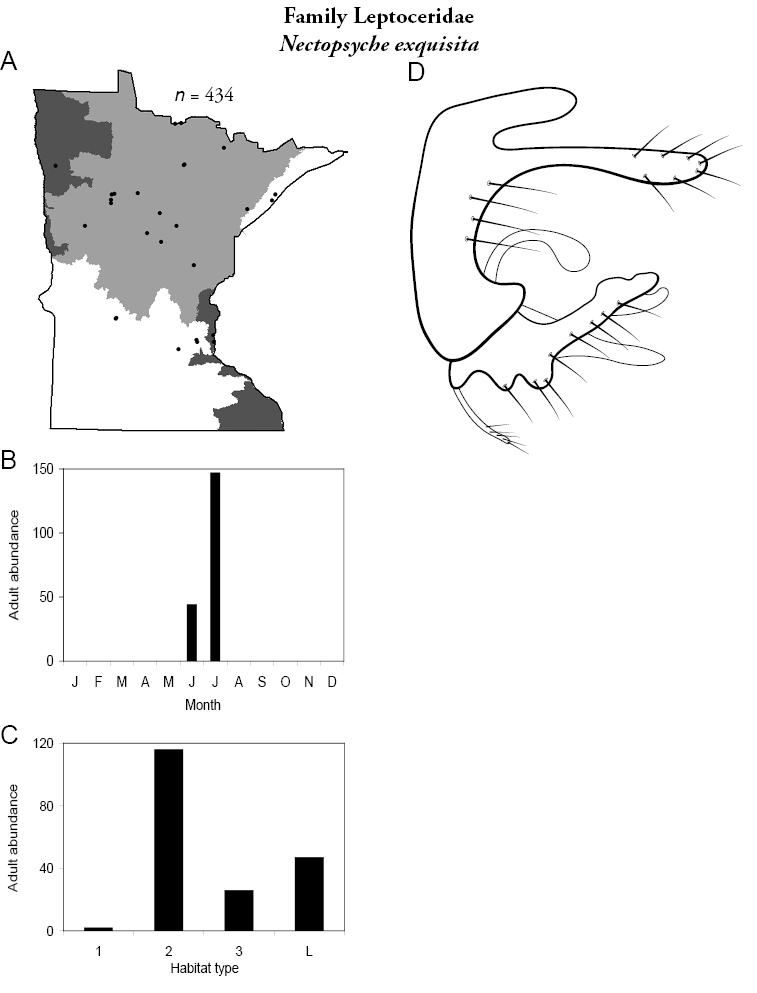
*Nectopsyche exquisita* **A** total specimens collected and all known collecting localities (Figure 4) **B** monthly adult abundance (1980s to present) **C** habitat preference (1980s to present) (Table 1) **D** male genital capsule.

***Nectopsyche exquisita*** (Figure 153) was mostly found in the Northern Region, and sporadically elsewhere in the state. It was found in all habitats except small streams, and was most abundant in medium rivers.

**Figure 154.**
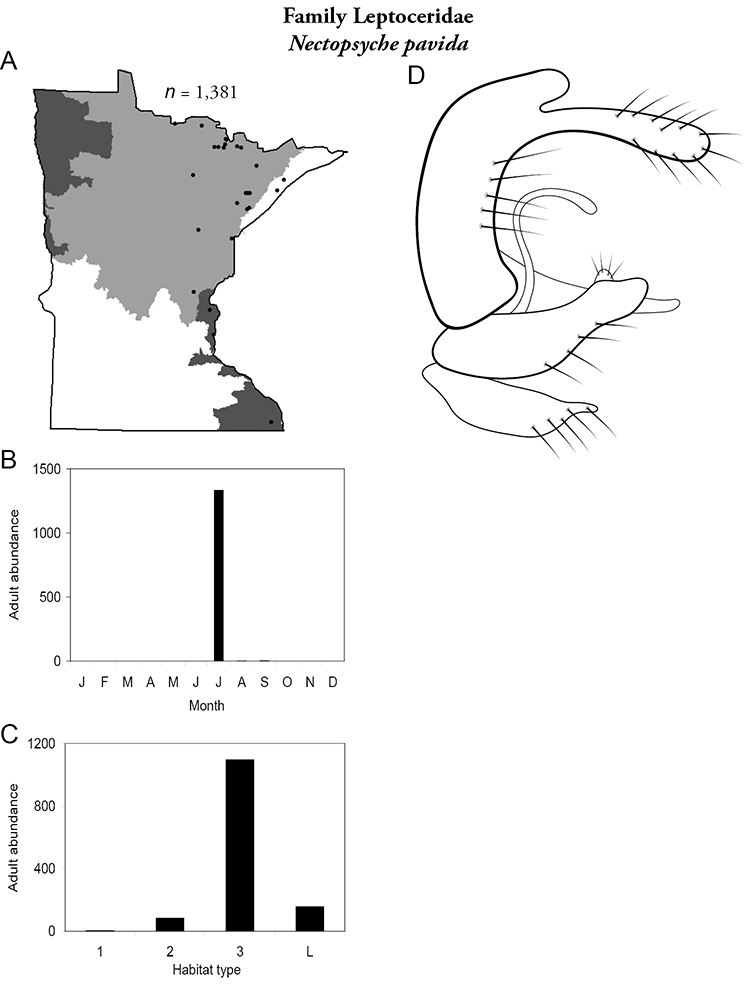
*Nectopsyche pavida* **A** total specimens collected and all known collecting localities (Figure 4) **B** monthly adult abundance (1980s to present) **C** habitat preference (1980s to present) (Table 1) **D** male genital capsule.

***Nectopsyche pavida*** (Figure 154) was collected from the eastern third of the state during July. It was most abundant in large rivers.

### Genus *Oecetis*

The genus *Oecetis* contains 10 species in Minnesota. It is the 8th most species-rich genus in the state (Figure 7). It contains several species in or near the top 10 of most abundant and widespread species in the state (Figures 8–9). Larvae are predatory and can be found on the bottom substrates of lakes and slow-moving rivers. Cases are normally composed of mineral particles, but may also include pieces of bark or leaves (Wiggins 1996). Adult length ranges 8–12 mm. Wings are typically light brown with darker wing bars and are very setose. *Oecetis avara*, *Oecetis cinerascens*, and *Oecetis disjuncta* have spots on their wings instead of bars (Figure 292). Separating the males of *Oecetis ditissa*, *Oecetis inconspicua*, and *Oecetis nocturna* requires careful examination of the inferior appendages in ventral view.

**Figure 155.**
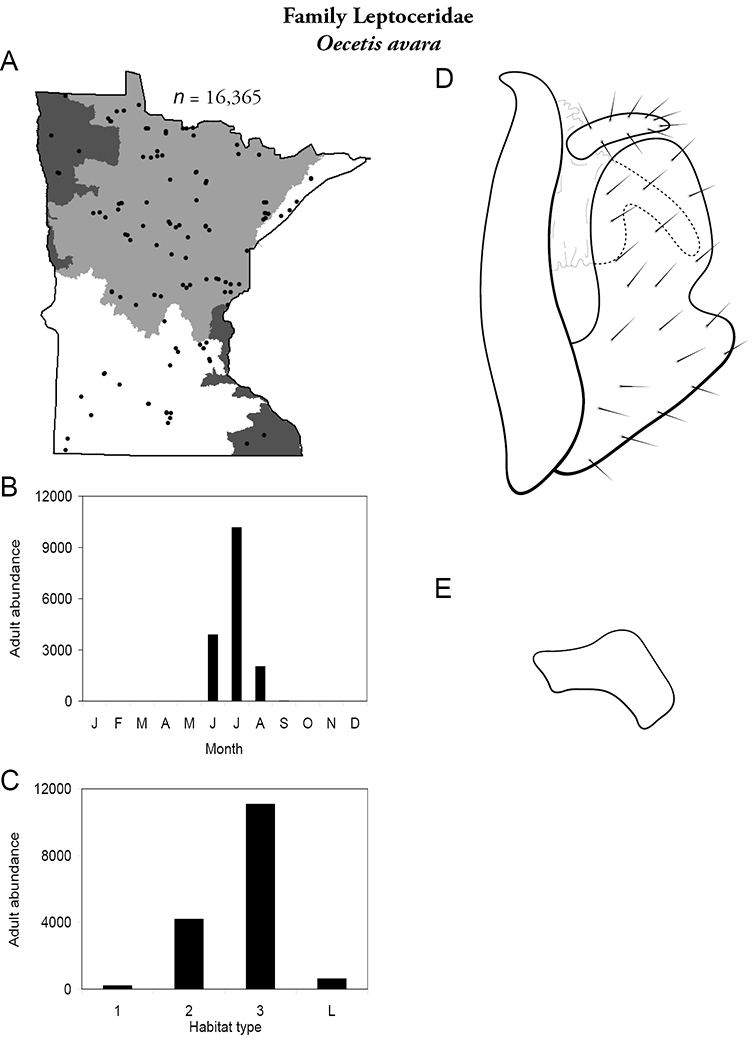
*Oecetis avara* **A** total specimens collected and all known collecting localities (Figure 4) **B** monthly adult abundance (1980s to present) **C** habitat preference (1980s to present) (Table 1) **D** male genital capsule **E** phallus.

***Oecetis avara*** (Figure 155) was abundant in all regions of Minnesota. Overall, it was the 5th most abundant species in the state (Figure 9). It was the 2nd most abundant species in both medium and large rivers of the Northern Region (Table 4). It was found in all habitat types, but was most abundant overall in large rivers. Adults were present from June to August.

**Figure 156.**
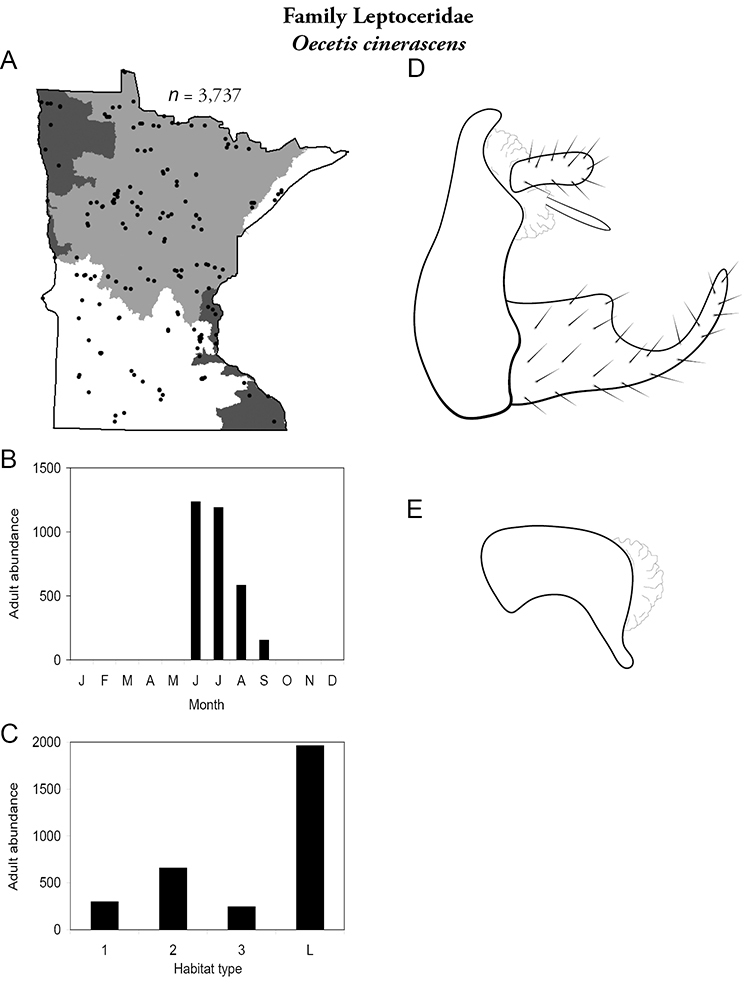
*Oecetis cinerascens* **A** total specimens collected and all known collecting localities (Figure 4) **B** monthly adult abundance (1980s to present) **C** habitat preference (1980s to present) (Table 1) **D** male genital capsule **E** phallus.

***Oecetis cinerascens*** (Figure 156) was found in all regions of Minnesota. Overall, it was the 3rd most widespread species in the state (Figure 8). It was found in all habitat types, but was most abundant in lakes. Adults were abundant in June and July and present in August and September.

**Figure 157.**
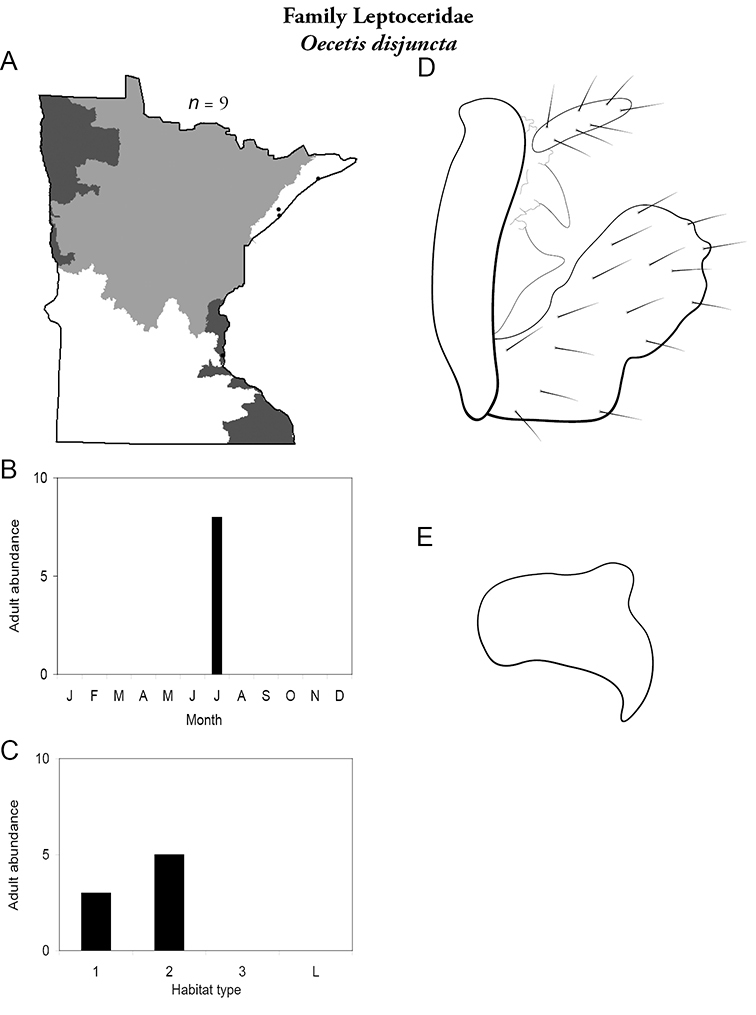
*Oecetis disjuncta* **A** total specimens collected and all known collecting localities (Figure 4) **B** monthly adult abundance (1980s to present) **C** habitat preference (1980s to present) (Table 1) **D** male genital capsule **E** phallus.

***Oecetis disjuncta*** (Figure 157) has been collected only from small and medium streams in the Lake Superior and Southeastern regions. All streams were cold and fast-moving. Adults were present only in July.

**Figure 158.**
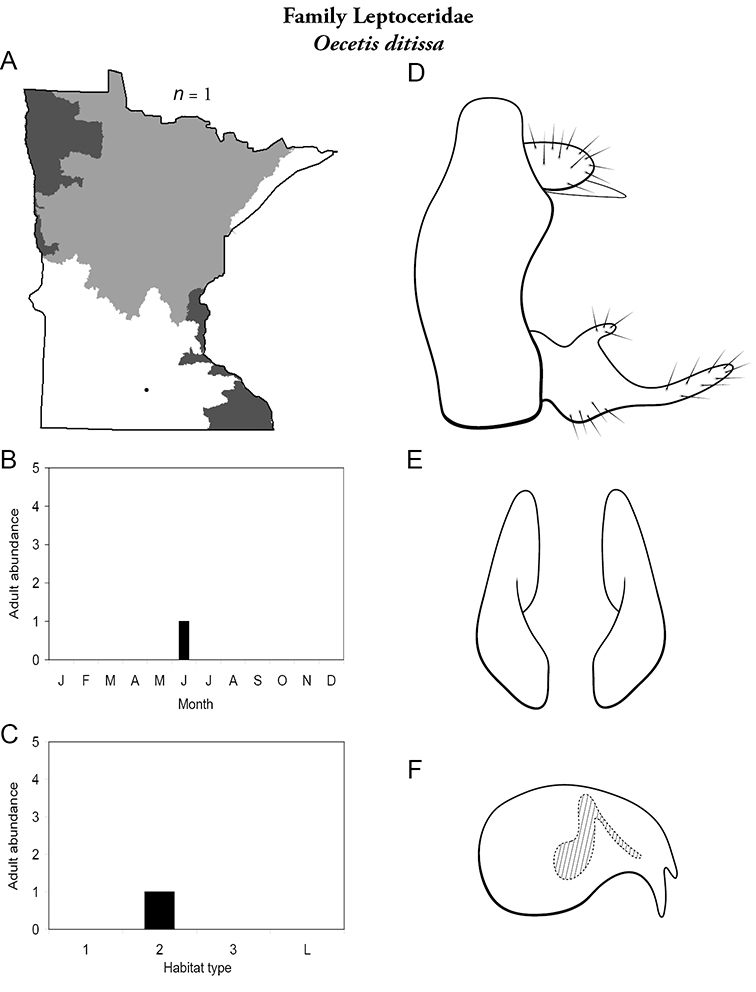
*Oecetis ditissa* **A** total specimens collected and all known collecting localities (Figure 4) **B** monthly adult abundance (1980s to present) **C** habitat preference (1980s to present) (Table 1) **D** male genital capsule **E** male inferior appendages (ventral view) **F** phallus.

***Oecetis ditissa*** (Figure 158) is known in Minnesota only from a single specimen collected from Minneopa Creek, Minneopa State Park, in the Southern Region during June 2000. The species appears to be at the northwestern edge of its known range. Due to the extreme rarity of *Oecetis ditissa* in the state and the high degree of habitat degradation in southern Minnesota (Houghton 2007), the Minnesota Department of Natural Resources has proposed “Threatened” status for the species (MNDNR 2012).

**Figure 159.**
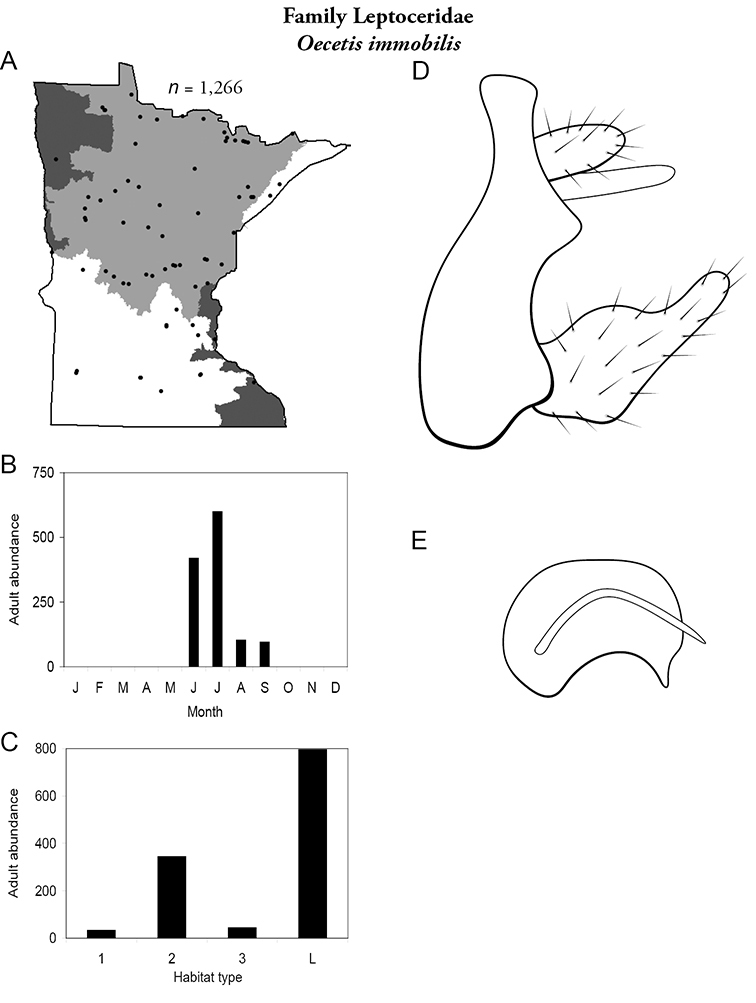
*Oecetis immobilis* **A** total specimens collected and all known collecting localities (Figure 4) **B** monthly adult abundance (1980s to present) **C** habitat preference (1980s to present) (Table 1) **D** male genital capsule **E** phallus.

***Oecetis immobilis*** (Figure 159) has been collected predominantly from the Northern and Southern Regions, but found in all regions. It was found mostly in lakes and medium rivers. Adults were abundant in June and July and also present in August and September.

**Figure 160.**
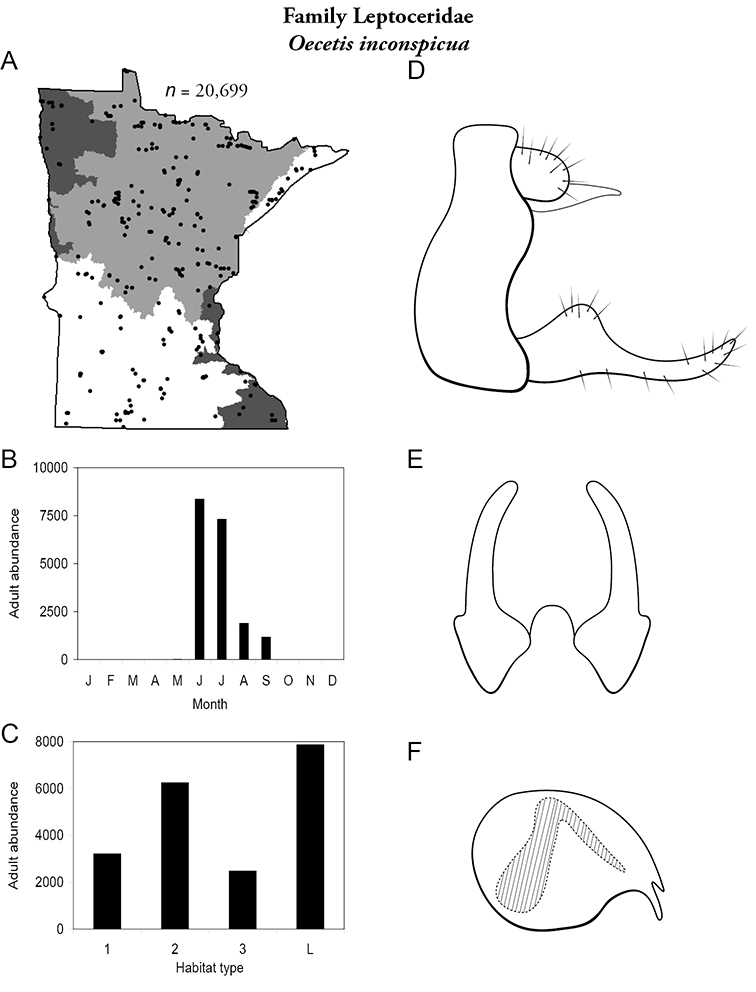
*Oecetis inconspicua* **A** total specimens collected and all known collecting localities (Figure 4) **B** monthly adult abundance (1980s to present) **C** habitat preference (1980s to present) (Table 1) **D** male genital capsule **E** male inferior appendages (ventral view) **F** phallus.

***Oecetis inconspicua*** (Figure 160) was commonly collected throughout all regions of the state. It was, by far, the most widespread species in Minnesota, found in >80% of all collections in the state (Figure 8). It was also the 3rd most abundant species overall (Figure 9). It was abundant in all habitat types, especially lakes and medium rivers. Adults were present from June through September. In short, nearly any ultraviolet light trap set near an aquatic habitat in Minnesota during warm weather will likely yield this species.

**Figure 161.**
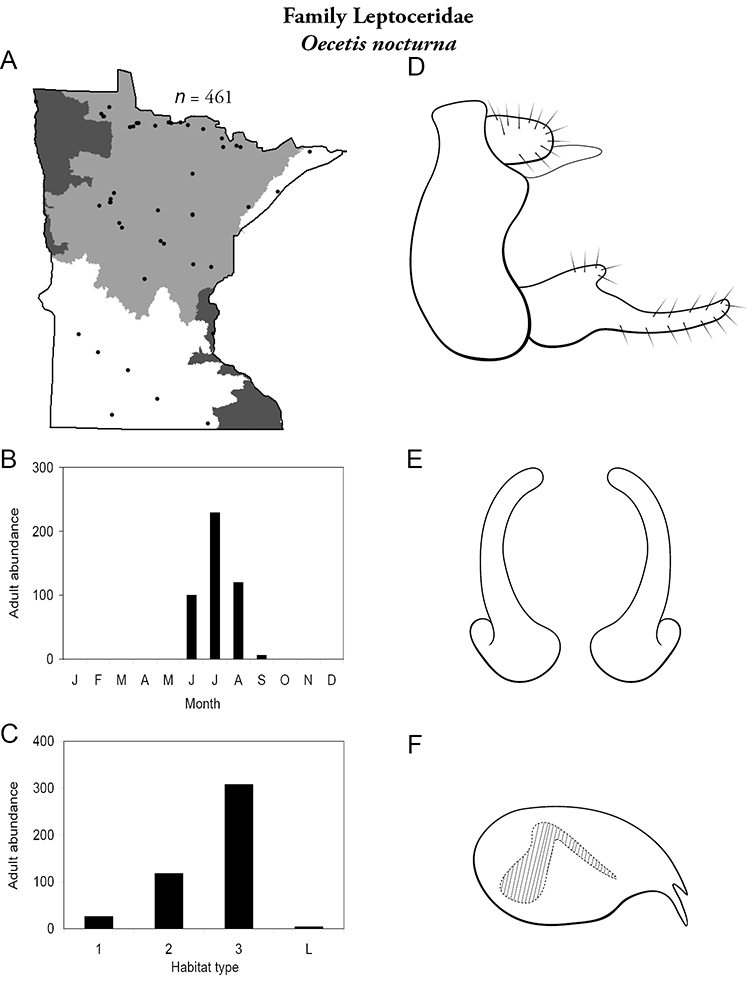
*Oecetis nocturna* **A** total specimens collected and all known collecting localities (Figure 4) **B** monthly adult abundance (1980s to present) **C** habitat preference (1980s to present) (Table 1) **D** male genital capsule **E** male inferior appendages (ventral view) **F** phallus.

***Oecetis nocturna*** (Figure 161) was found predominantly in the Northern and Southern Regions of the state. It was most abundant in medium and, especially, large rivers from June through August.

**Figure 162.**
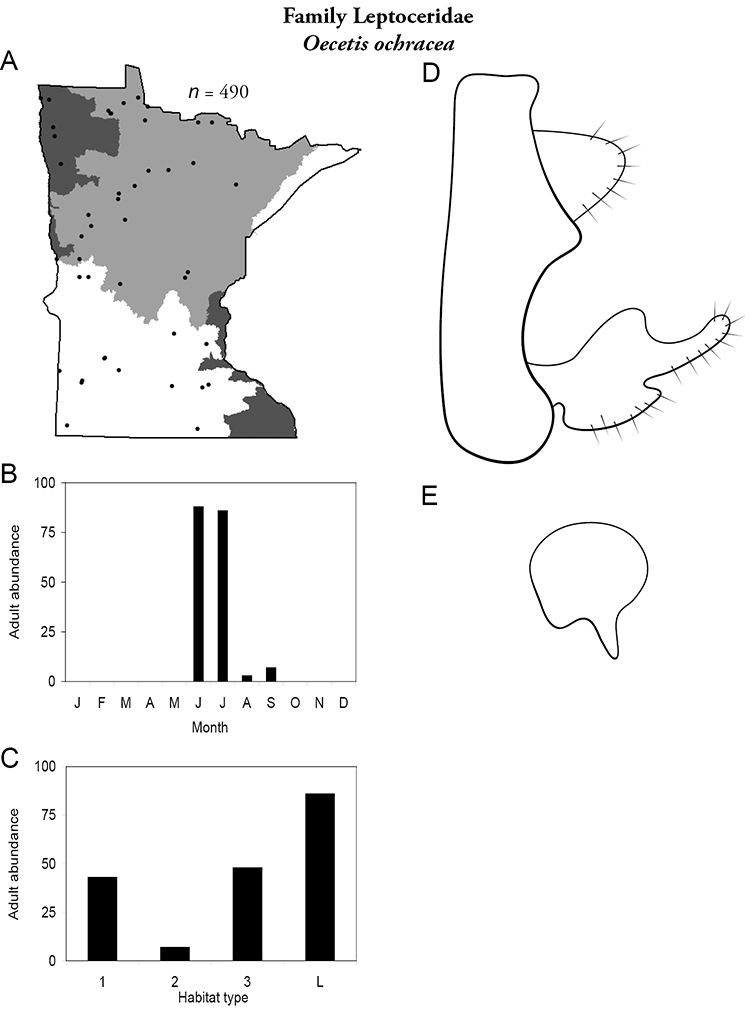
*Oecetis ochracea* **A** total specimens collected and all known collecting localities (Figure 4) **B** monthly adult abundance (1980s to present) **C** habitat preference (1980s to present) (Table 1) **D** male genital capsule **E** phallus.

***Oecetis ochracea*** (Figure 162) was found in the Northern, Northwestern, and Southern Regions. It was abundant in both June and July and found in all habitat types, but was least abundant in medium rivers.

**Figure 163.**
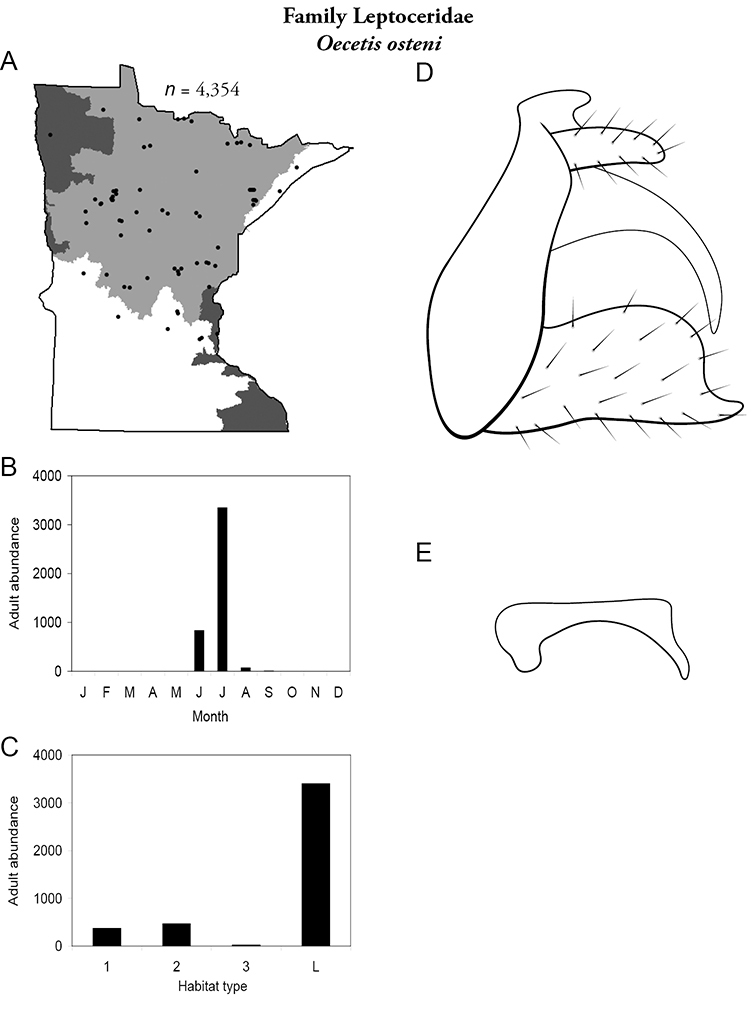
*Oecetis osteni* **A** total specimens collected and all known collecting localities (Figure 4) **B** monthly adult abundance (1980s to present) **C** habitat preference (1980s to present) (Table 1) **D** male genital capsule **E** phallus.

***Oecetis osteni*** (Figure 163) was collected mostly from or near the Northern Region and found sporadically elsewhere in the state. It was most abundant in lakes during July, with some specimens found in June and August. It was the 3rd most abundant species in lakes of the Northern Region (Table 4).

**Figure 164.**
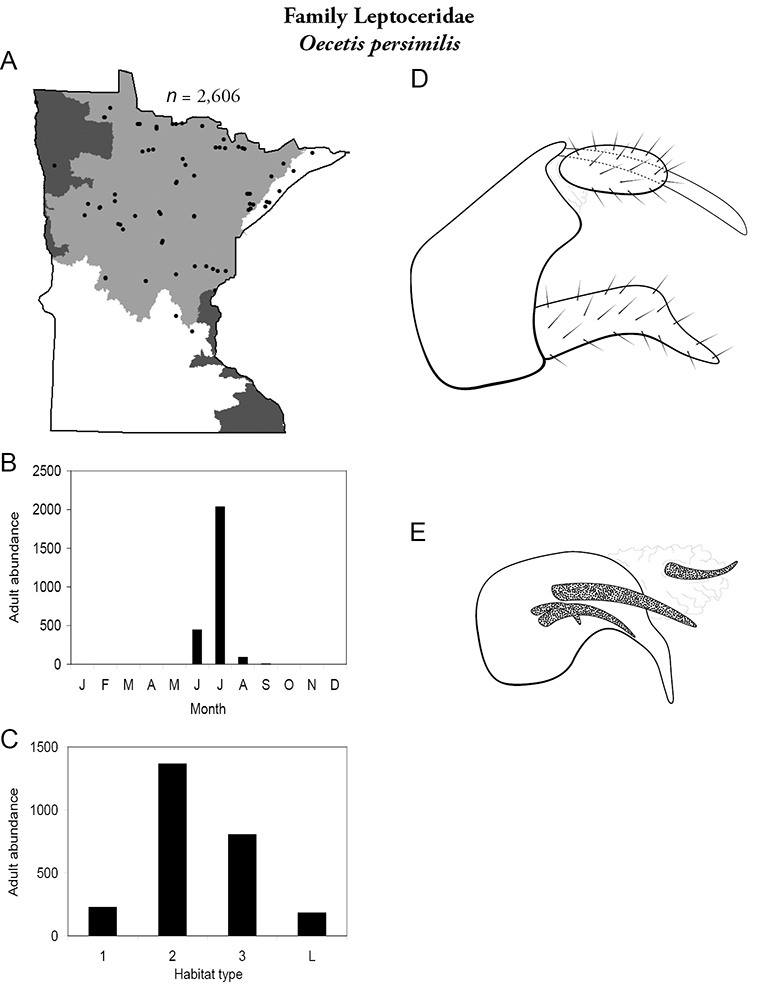
*Oecetis persimilis* **A** total specimens collected and all known collecting localities (Figure 4) **B** monthly adult abundance (1980s to present) **C** habitat preference (1980s to present) (Table 1) **D** male genital capsule **E** phallus.

***Oecetis persimilis*** (Figure 164) is known mainly from the Lake Superior and Northern Regions. It was found mostly in medium and large rivers, and was abundant in July with some specimens collected in June and August.

### Genus *Setodes*

The genus *Setodes* contains 2 species in Minnesota. For additional species, see Holzenthal (1982). Cases are composed mainly of small mineral particles (Wiggins 1996).Larvae burrow into the loose sand of river bottoms, leaving a portion of their case above the substrate. Larvae consume both plants and animals. They are the smallest of the leptocerids, ranging 5–8 mm in adult length. Wings are pale yellow colored.

**Figure 165.**
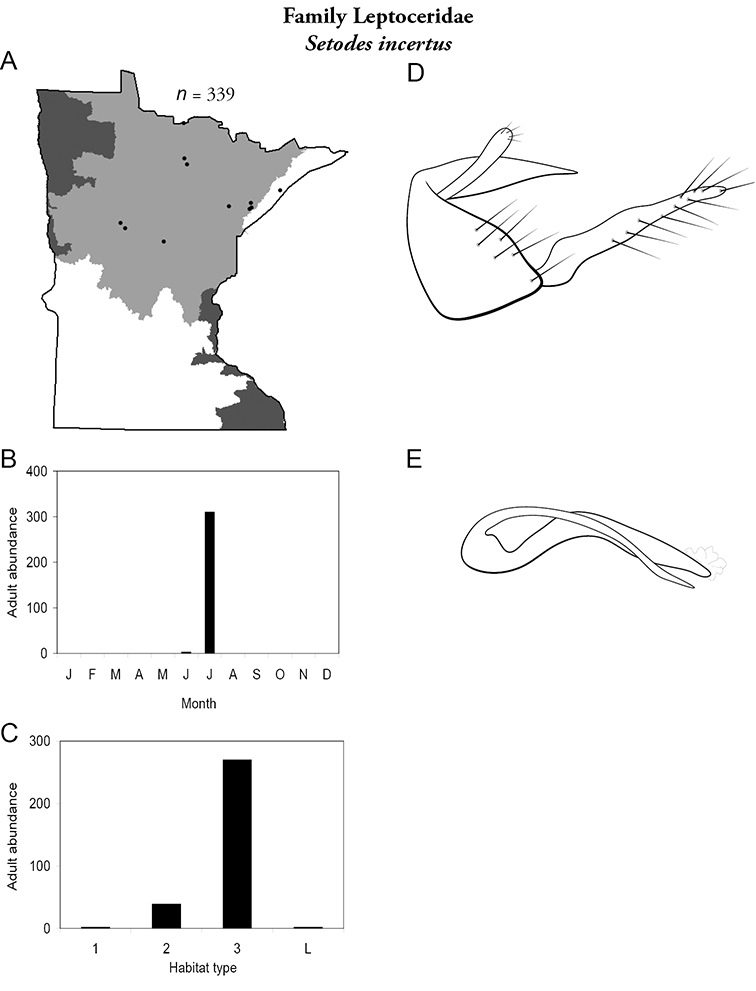
*Setodes incertus* **A** total specimens collected and all known collecting localities (Figure 4) **B** monthly adult abundance (1980s to present) **C** habitat preference (1980s to present) (Table 1) **D** male genital capsule **E** phallus.

***Setodes incertus*** (Figure 165) was collected predominantly from large rivers of the Northern Region during July.

**Figure 166.**
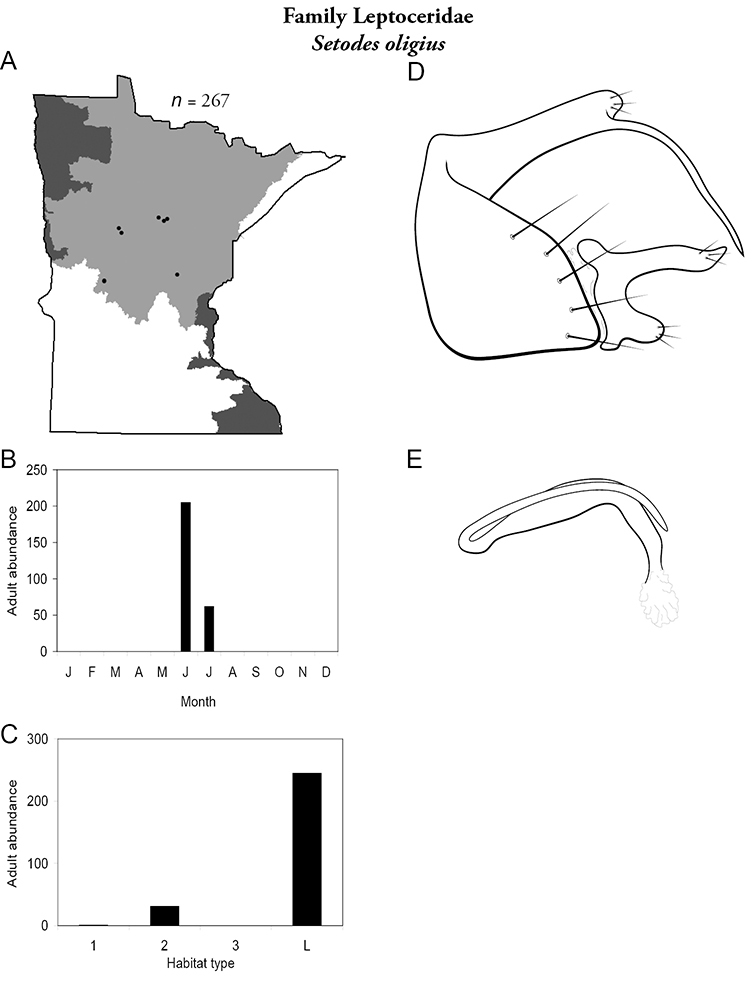
*Setodes oligius* **A** total specimens collected and all known collecting localities (Figure 4) **B** monthly adult abundance (1980s to present) **C** habitat preference (1980s to present) (Table 1) **D** male genital capsule **E** phallus.

***Setodes oligius*** (Figure 166) was found in lakes and, occasionally, medium streams of the Northern Region during June and July.

A 3rd *Setodes* species, *Setodes guttatus*, has been reported from Minnesota based on a female specimen (e.g., Etnier 1965). Due to its perceived rarity, it is listed as “Special Concern” by the Minnesota Department of Natural Resources (MNDNR 2012). Further examination, however, reidentified all *Setodes guttatus* specimens as *Setodes oligius* (Houghton and Holzenthal 2003), prompting the Minnesota Department of Natural Resources to propose delisting the species, as it almost certainly does not exist in Minnesota (MNDNR 2012). Thus, *Setodes guttatus* is not included in this manual.

### Genus *Triaenodes*

The genus *Triaenodes* contains 9 species in Minnesota. It is the 10th most species-rich genus (Figure 7). For additional species, see Manuel (2010). Larvae inhabit the beds of submerged aquatic plants. They are unique among caddisflies in their consumption of living plant material. Cases are composed of plant pieces arranged in a spiral pattern (Wiggins 1996). Adults range 8–12 mm in length. Forewings are usually yellow or light brown with distinct dark patterning on some species (Figure 292). The genus contains several common and abundant species, as well as some that are rarely collected. Females often occur in large numbers in light traps. Unfortunately, although Manuel (2010) does describe females of the genus, many of the species are very difficult to identify without male specimens.

**Figure 167.**
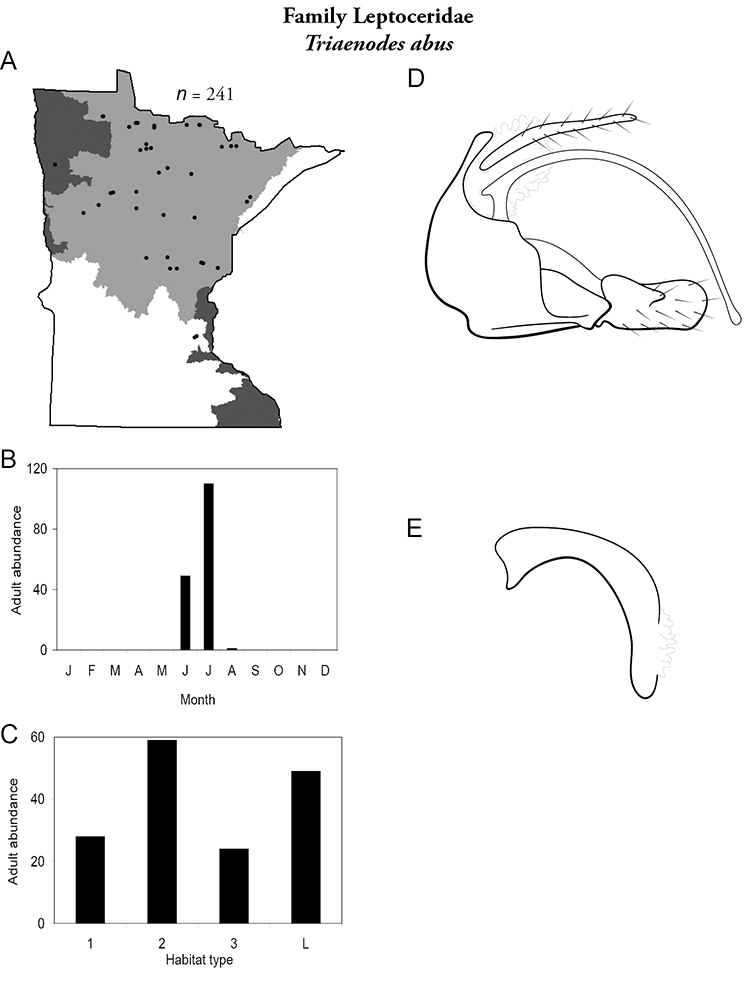
*Triaenodes abus* **A** total specimens collected and all known collecting localities (Figure 4) **B** monthly adult abundance (1980s to present) **C** habitat preference (1980s to present) (Table 1) **D** male genital capsule **E** phallus.

***Triaenodes abus*** (Figure 167) was commonly collected in the Northern Region and sporadically elsewhere. It was found in all types of habitats, especially lakes and medium rivers. Adults were abundant in June and July, with a few specimens present in August.

**Figure 168.**
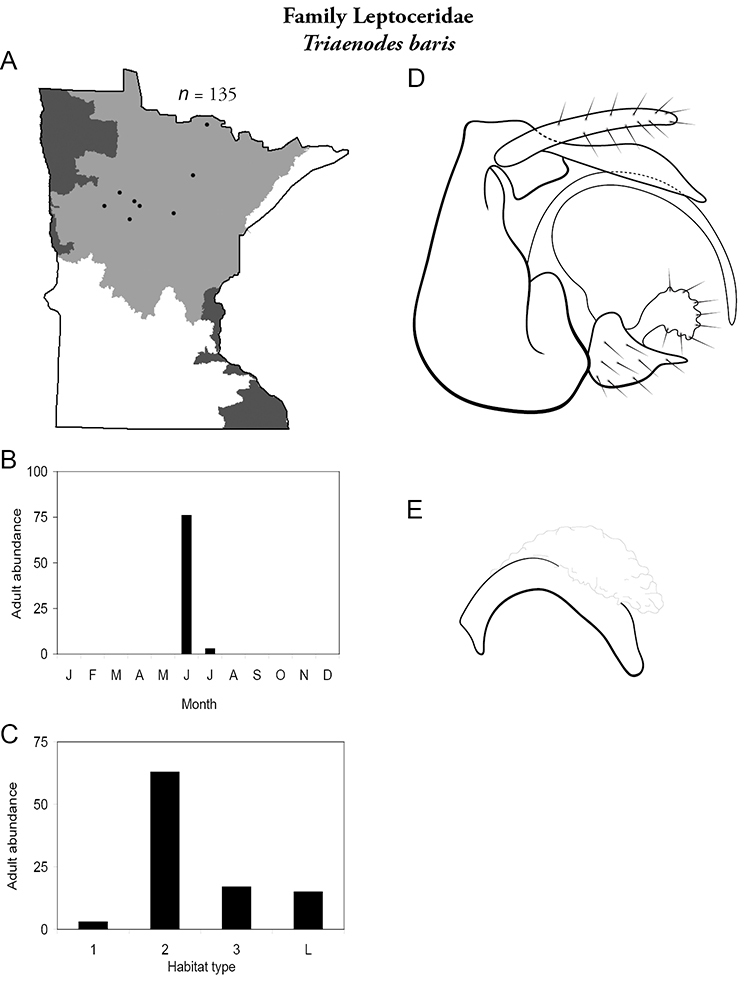
*Triaenodes baris* **A** total specimens collected and all known collecting localities (Figure 4) **B** monthly adult abundance (1980s to present) **C** habitat preference (1980s to present) (Table 1) **D** male genital capsule **E** phallus.

***Triaenodes baris*** (Figure 168) has been found exclusively in the Northern Region, mostly from medium rivers in June, but also from large rivers and lakes.

**Figure 169.**
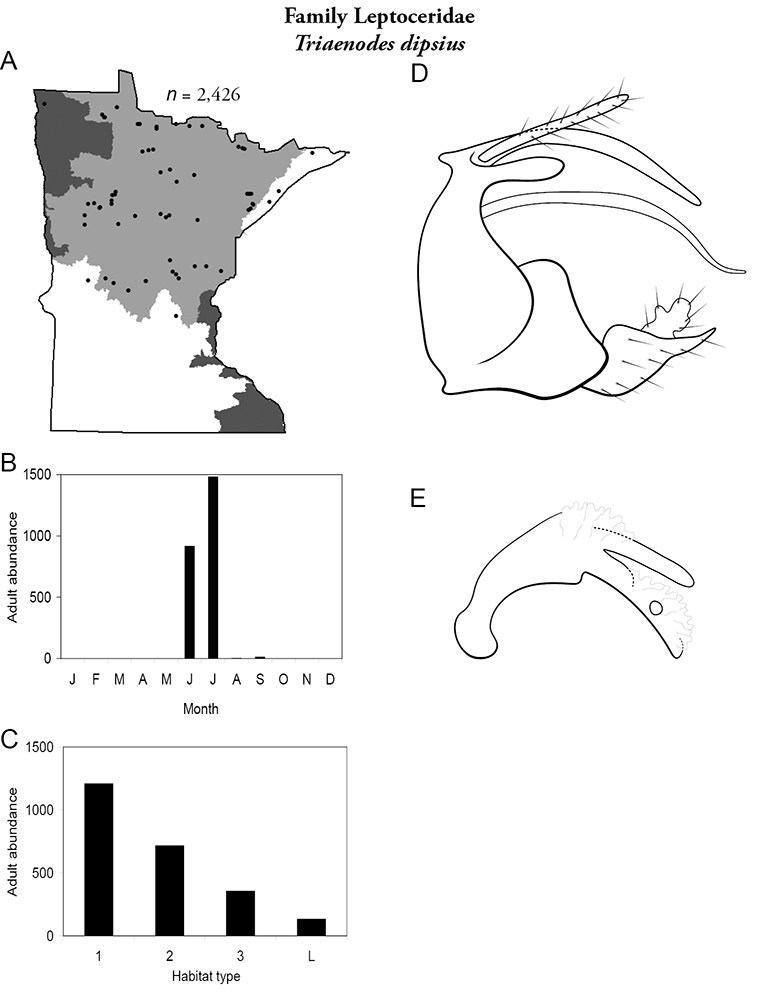
*Triaenodes dipsius* **A** total specimens collected and all known collecting localities (Figure 4) **B** monthly adult abundance (1980s to present) **C** habitat preference (1980s to present) (Table 1) **D** male genital capsule **E** phallus.

***Triaenodes dipsius*** (Figure 169) was found predominantly in the Northern Region, and sporadically elsewhere. Adults were abundant in both June and July, with a few specimens present in August and September. It was most abundant in streams, especially small streams.

**Figure 170.**
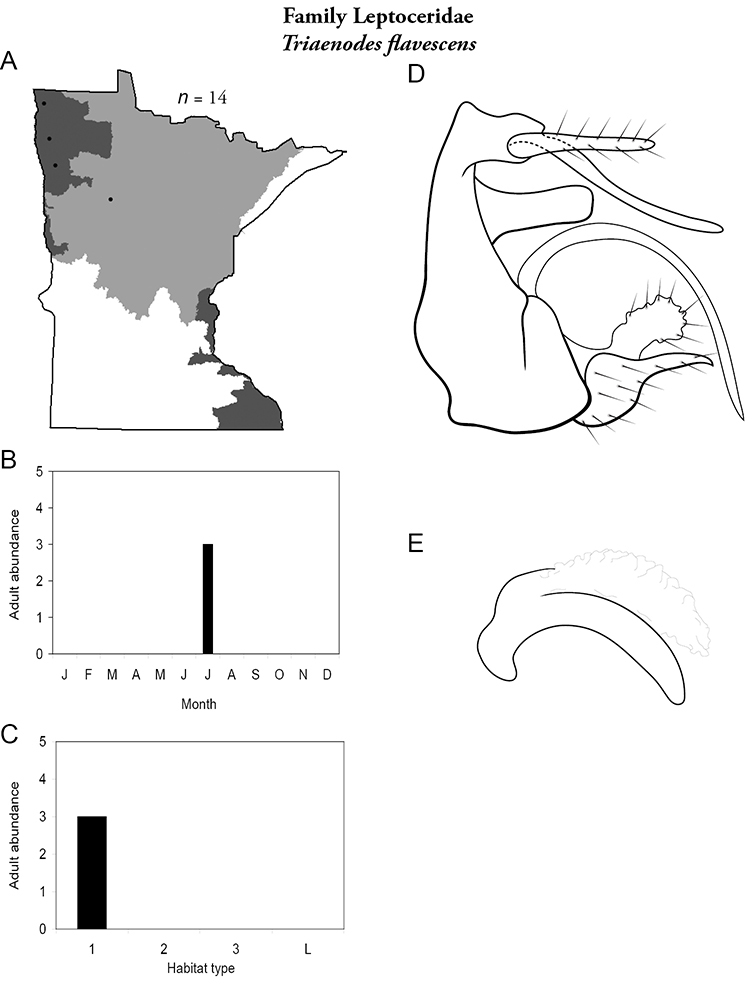
*Triaenodes flavescens* **A** total specimens collected and all known collecting localities (Figure 4) **B** monthly adult abundance (1980s to present) **C** habitat preference (1980s to present) (Table 1) **D** male genital capsule **E** phallus.

***Triaenodes flavescens*** (Figure 170) is known historically from the Northwestern Region. The species has not been collected in that region since the 1940s, however. The only recent collection occurred in Sucker Creek, Clearwater County, in the Northern Region, during July 1988. Due to its rarity and apperent decrease in distribution, the Minnesota Department of Natural Resources has proposed “Special Concern” status for the species (MNDNR 2012).

**Figure 171.**
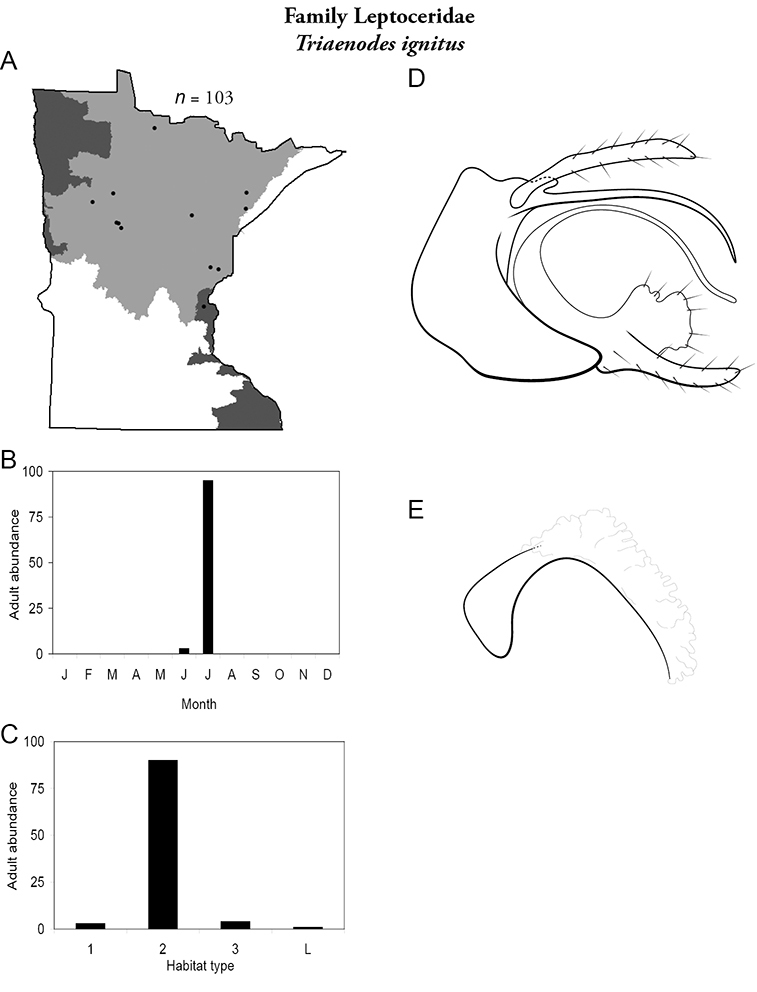
*Triaenodes ignitus* **A** total specimens collected and all known collecting localities (Figure 4) **B** monthly adult abundance (1980s to present) **C** habitat preference (1980s to present) (Table 1) **D** male genital capsule **E** phallus.

***Triaenodes ignitus*** (Figure 171) is known mainly from the Northern Region where it occurred predominantly in medium streams in July.

**Figure 172.**
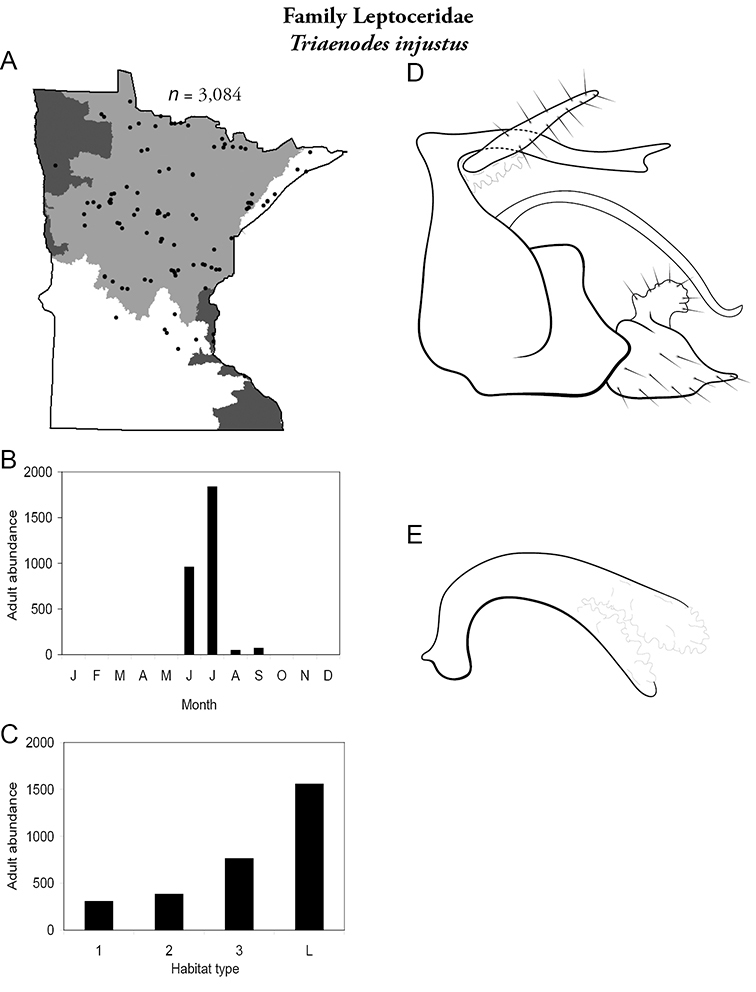
*Triaenodes injustus* **A** total specimens collected and all known collecting localities (Figure 4) **B** monthly adult abundance (1980s to present) **C** habitat preference (1980s to present) (Table 1) **D** male genital capsule **E** phallus.

***Triaenodes injustus*** (Figure 172) was abundant in the Lake Superior and Northern Regions and found occasionally in other regions. It was the single most abundant species in lakes of the Lake Superior Region (Table 3), and also found in all sizes of streams. Adults were abundant in June and July and occasionally present in August and September.

**Figure 173.**
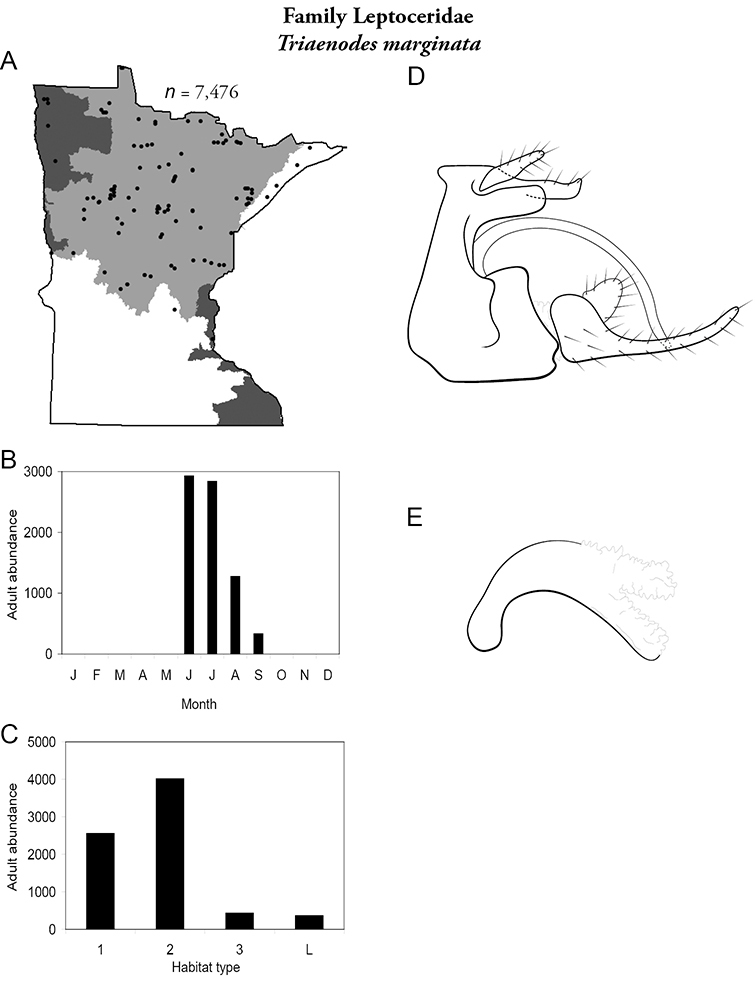
*Triaenodes marginatus* **A** total specimens collected and all known collecting localities (Figure 4) **B** monthly adult abundance (1980s to present) **C** habitat preference (1980s to present) (Table 1) **D** male genital capsule **E** phallus.

***Triaenodes marginata*** (Figure 173) was abundant in the Northern Region, especially in small and medium streams. It was also found sporadically in other regions. Overall, it was the 9th most abundant species in the state (Figure 9). Adults were abundant in June and July, moderately abundant in August, and occasionally present in September.

**Figure 174.**
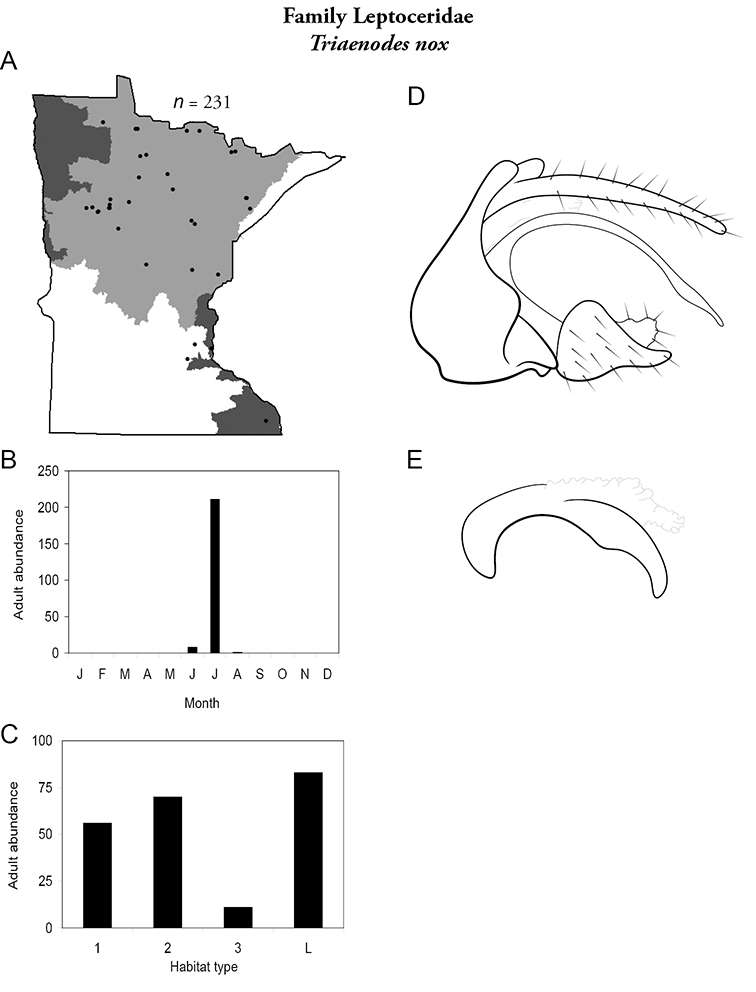
*Triaenodes nox* **A** total specimens collected and all known collecting localities (Figure 4) **B** monthly adult abundance (1980s to present) **C** habitat preference (1980s to present) (Table 1) **D** male genital capsule **E** phallus.

***Triaenodes nox*** (Figure 174) was abundant in the Northern Region and found sporadically in the other regions. Most adults were collected in July and were abundant in all habitat types except large rivers.

**Figure 175.**
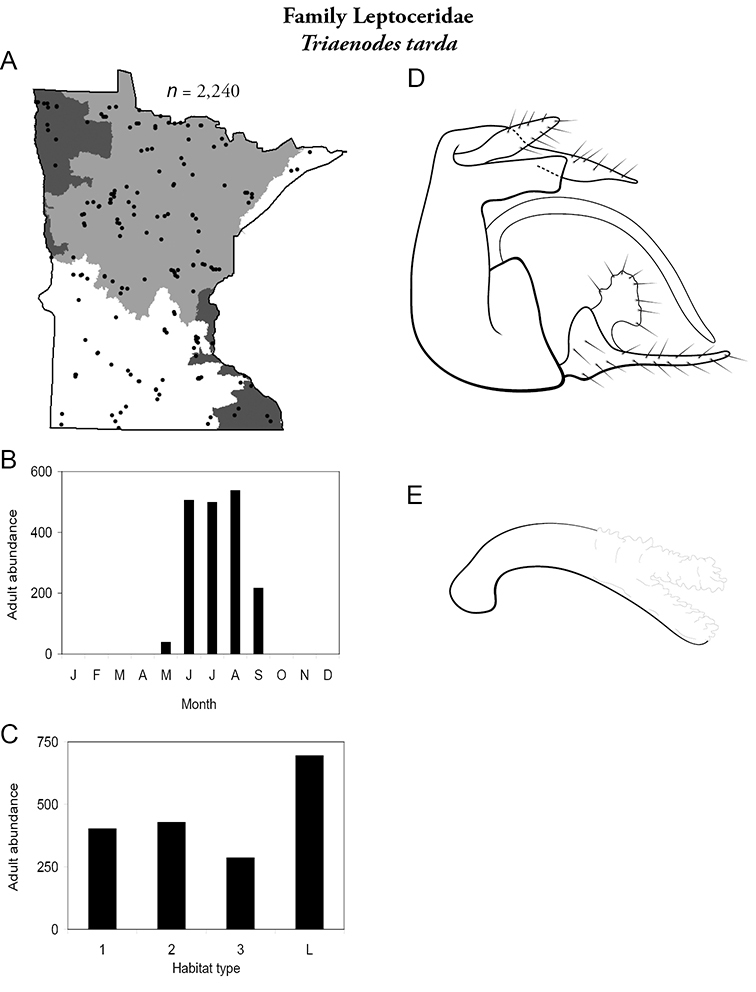
*Triaenodes tarda* **A** total specimens collected and all known collecting localities (Figure 4) **B** monthly adult abundance (1980s to present) **C** habitat preference (1980s to present) (Table 1) **D** male genital capsule **E** phallus.

***Triaenodes tarda*** (Figure 175) was the 2nd most widespread species in Minnesota (Figure 8), although it was not especially abundant. It was found in all regions and in all habitat types. Adults were present from May to September and abundant June through August.

Another *Triaenodes* species, *Triaenodes borealis*, was described by Banks (1900) from a female specimen from Minnesota. The description did not include an illustration and is likely a *nomen dubium*. Thus, it is not included in this manual.

### Genus *Ylodes*

The genus *Ylodes* contains 2 species in Minnesota. For additional species, see Manuel and Nimmo (1984). Larvae are similar to *Triaenodes* in both the cases that they build and in habitat, and the two genera appear closely related (Glover 1996, Wiggins 1996). In fact, Holzenthal and Anderson (2004) consider *Ylodes* to be a junior synonym of *Triaenodes*. Adults range 10–12 mm with dark gray wings.

**Figure 176.**
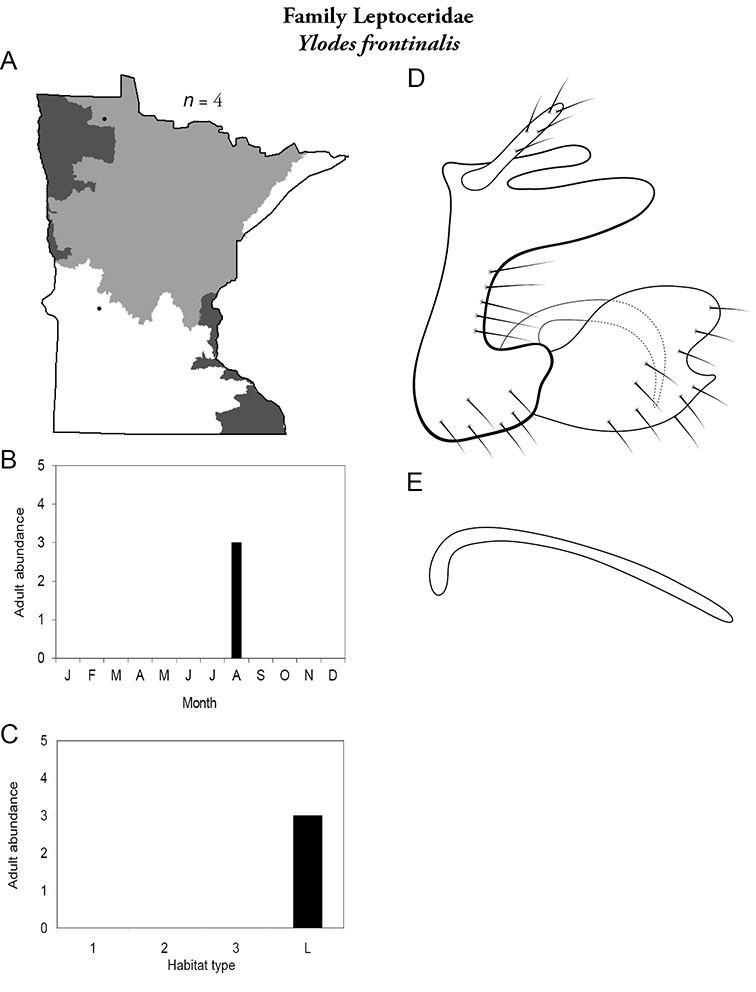
*Ylodes frontinalis* **A** total specimens collected and all known collecting localities (Figure 4) **B** monthly adult abundance (1980s to present) **C** habitat preference (1980s to present) (Table 1) **D** male genital capsule **E** phallus.

***Ylodes frontinalis*** (Figure 176) is known historically from Glacial Lake State Park in the Southern Region. The only specimens collected since the 1950s came from Hayes Lake, Hayes Lake State Park, in the Northern Region during August 2000. Due to the rarity of this species, and the loss of habitat throughout its known collecting area (Houghton 2007), “Threatened” status has been proposed for *Ylodes frontinalis* by the Minnesota Department of Natural Resources (MNDNR 2012).

**Figure 177.**
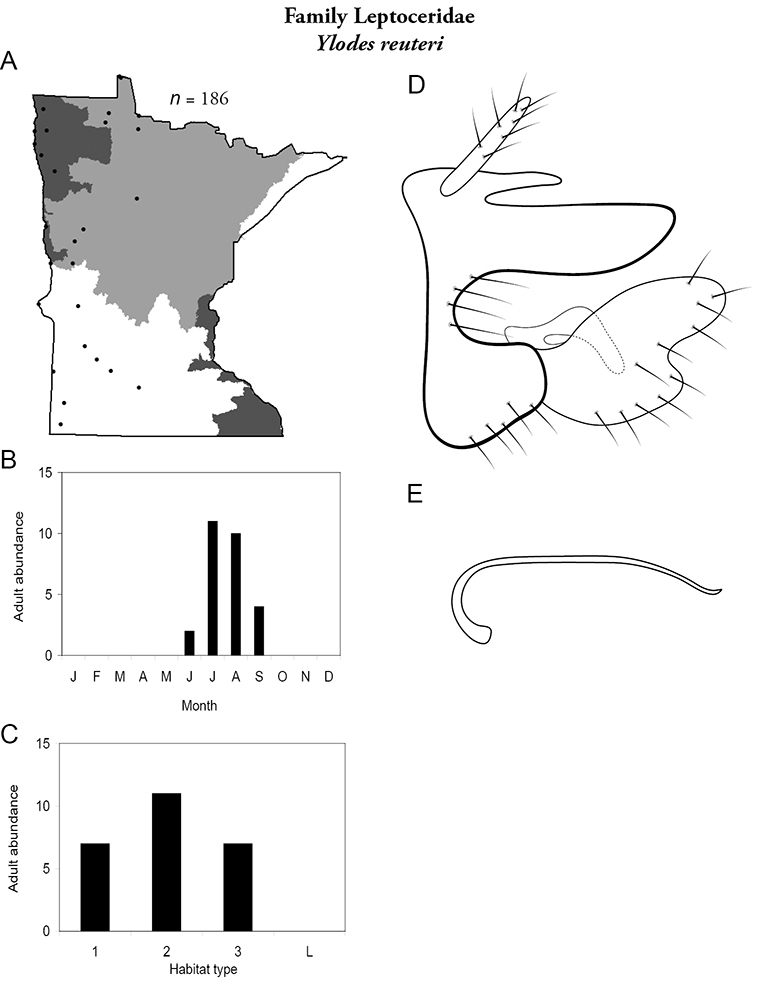
*Ylodes reuteri* **A** total specimens collected and all known collecting localities (Figure 4) **B** monthly adult abundance (1980s to present) **C** habitat preference (1980s to present) (Table 1) **D** male genital capsule **E** phallus.

***Ylodes reuteri*** (Figure 177) has been collected from the western third of the state. It was found in all sizes of streams, and present from June to September. Almost 90% of specimens, however, were found prior to 1945, suggesting that the species is decreasing in abundance.

### Family Limnephilidae

This family contains 19 genera in Minnesota: *Anabolia*, *Arctopora*, *Asynarchus*, *Chilostigma*, *Frenesia*, *Glyphopsyche*, *Grammotaulius*, *Hesperophylax*, *Hydatophylax*, *Ironoquia*, *Lenarchus*, *Leptophylax*, *Limnephilus*, *Nemotaulius*, *Onocosmoecus*, *Philarctus*, *Platycentropus*, *Pseudostenophylax*, and *Pycnopsyche*, and a total of 50 species. It is the 2nd most species-rich family in the state. Many species, however, have not been collected since the 1960s or earlier.

Larvae are found throughout a wide variety of lotic and lentic habitats. Cases are tubular in shape and constructed out of rock or plant material. Larvae range in size from 15 to 35 mm in length; cases of some species reach 80 mm. Most species are shredders, dependent on allochthonous input for food and case-building material (Wiggins 1996). Limnephilid adults are some of the largest caddisflies in Minnesota, ranging in length from 12–40 mm. Wings can be nearly any color, and frequently have notable patterning. Females of most genera are readily identifiable and so are included in this manual.

Due to their large size and corresponding long lifespan, and due to their dependence on allochthonous debris as a food source, most limnephilids are sensitive to habitat disturbance, especially removal of forest canopy cover. Thus, species in general appear to have been regionally extirpated from their historical habitats at a rate nearly 3× that of species in other families (Houghton and Holzenthal 2010). Many individual species have protected status in Minnesota, and many others appear to have decreased in distribution. Limnephilids have all but disappeared from the Northwestern and Southern Regions, despite being widespread in these regions historically. This loss is likely due to the loss of riparian canopy cover with subsequent increase in organic pollution from agriculture (Houghton 2007).

Although limnephilids remain widespread throughout the Northern and Lake Superior regions, they are usually not abundant. Furthermore, many species are present as adults during the fall and even during the winter. Thus, they can be difficult to collect. Most Minnesota limnephilid collections have yielded <5 specimens. The exception is *Pycnopsyche*, which can be very abundant during August and September. Due to this typical lack of abundance, nearly a dozen limnephilid species have been discovered in Minnesota since the 1980s and additional species may remain undiscovered.

### Genus *Anabolia*

The genus *Anabolia* contains 4 species in Minnesota. Two of the species are fairly widespread, 1 is listed as “Special Concern” by the Minnesota Department of Natural Resources (MNDNR 2012), and 1 is likely extirpated from the state. Larvae are typically found in lakes and slow-moving areas of streams where they consume decaying wood and other plant debris. Larval cases are composed of pieces of organic debris, usually arranged lengthwise (Wiggins 1996). Adults range 14–20 mm and are brown in color, with darker reticulations on the forewings. For additional species, see Schmid (1950).

**Figure 178.**
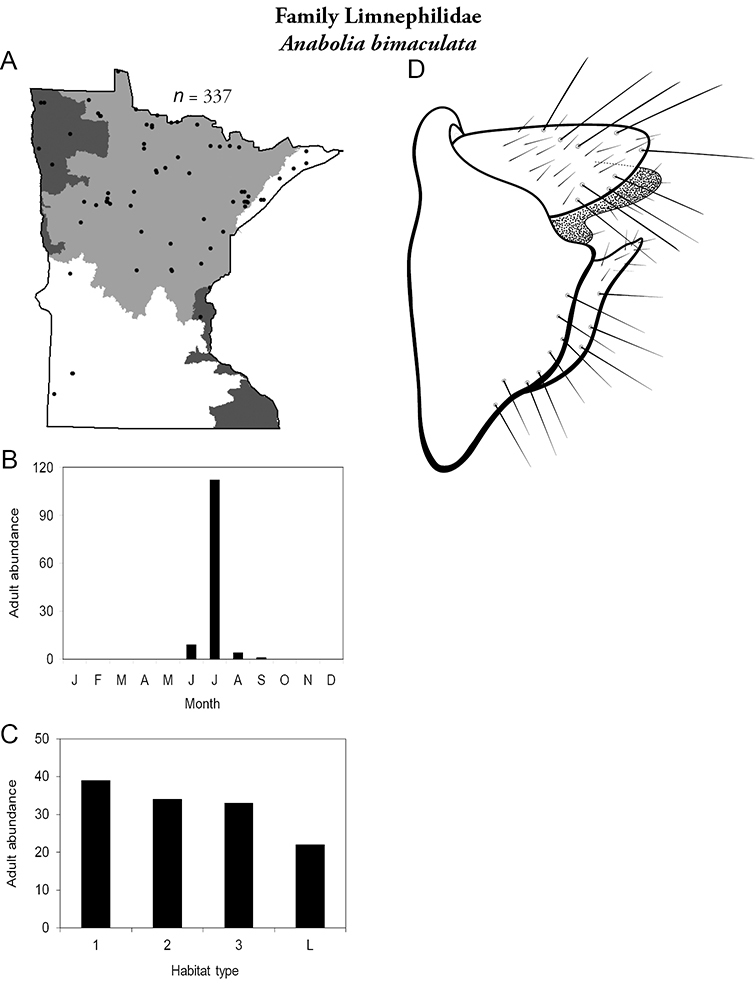
*Anabolia bimaculata* **A** total specimens collected and all known collecting localities (Figure 4) **B** monthly adult abundance (1980s to present) **C** habitat preference (1980s to present) (Table 1) **D** male genital capsule.

***Anabolia bimaculata*** (Figure 178) is known primarily from the northern half of the state, with occasional collections elsewhere. It was widespread throughout all habitats of the Lake Superior and Northern regions, although it was rarely abundant. Adults were collected mostly during July.

**Figure 179.**
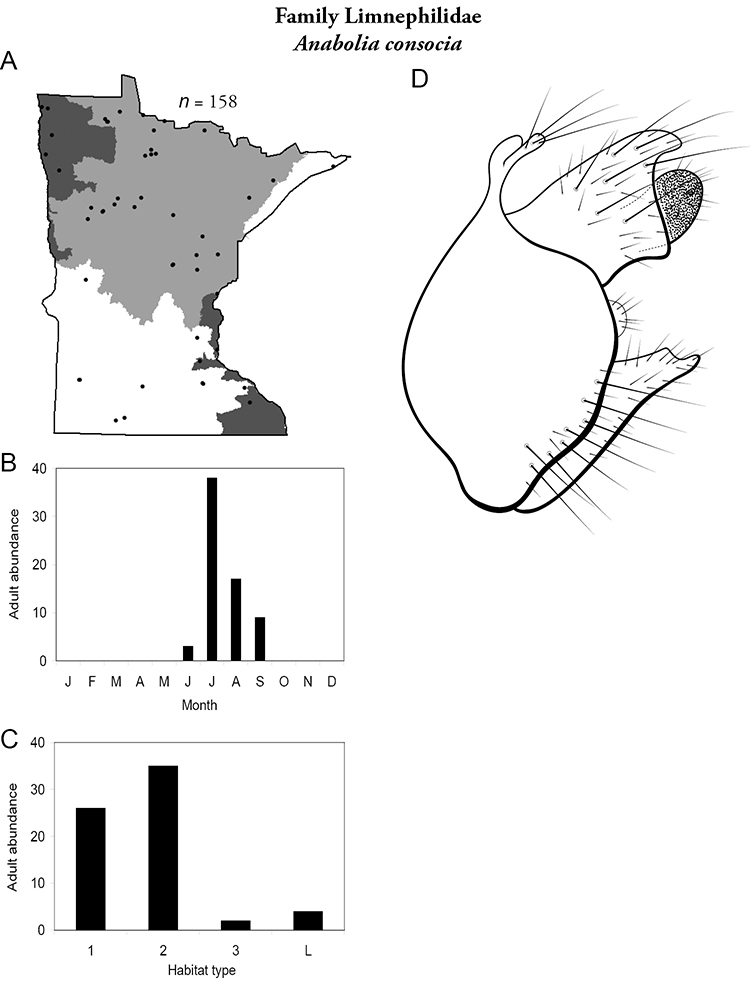
*Anabolia consocia* **A** total specimens collected and all known collecting localities (Figure 4) **B** monthly adult abundance (1980s to present) **C** habitat preference (1980s to present) (Table 1) **D** male genital capsule.

***Anabolia consocia*** (Figure 179) has similar distribution and habitat preference as *Anabolia bimaculata*; the two species were often collected together. It differs from the latter species in being more frequently collected in the southern half of the state and in having adults commonly collected into September. Overall, it was less abundant that *Anabolia bimaculata*.

**Figure 180.**
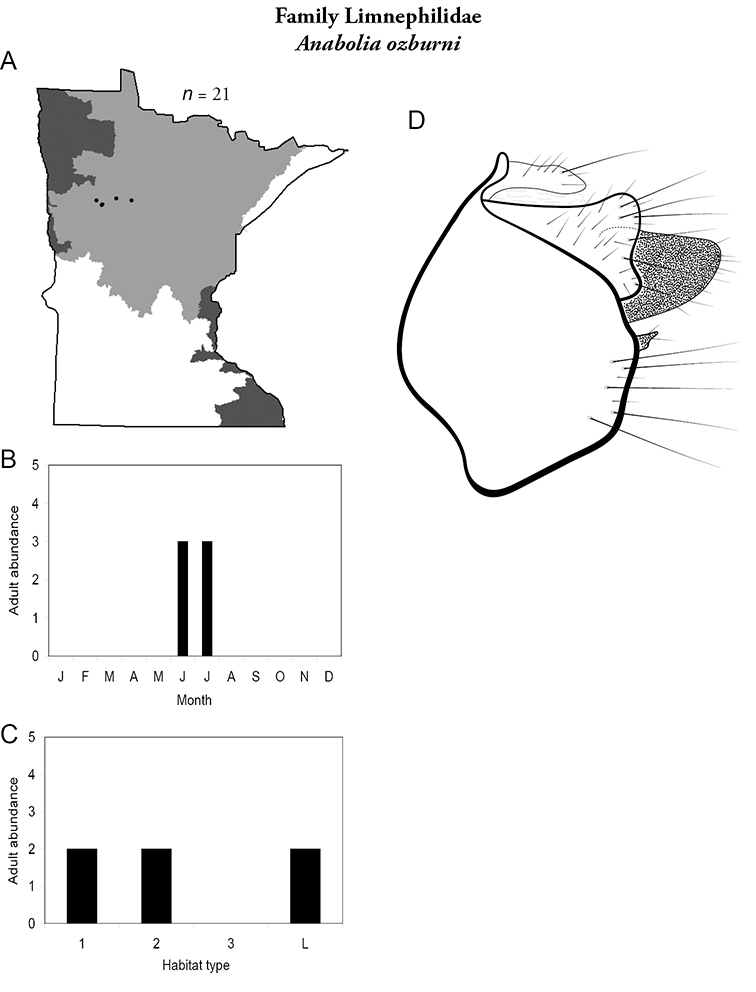
*Anabolia ozburni* **A** total specimens collected and all known collecting localities (Figure 4) **B** monthly adult abundance (1980s to present) **C** habitat preference (1980s to present) (Table 1) **D** male genital capsule.

***Anabolia ozburni*** (Figure 180) is known only from 6 specimens collected in the westcentral portion of the Northern Region during both June and July. It has been collected from both small and medium streams and their lentic headwaters. Due to the rarity of *Anabolia ozburni* and the vulnerability to riparian disturbance of habitats throughout its known range, the Minnesota Department of Natural Resources has proposed “Special Concern” status for the species (MNDNR 2012).

**Figure 181.**
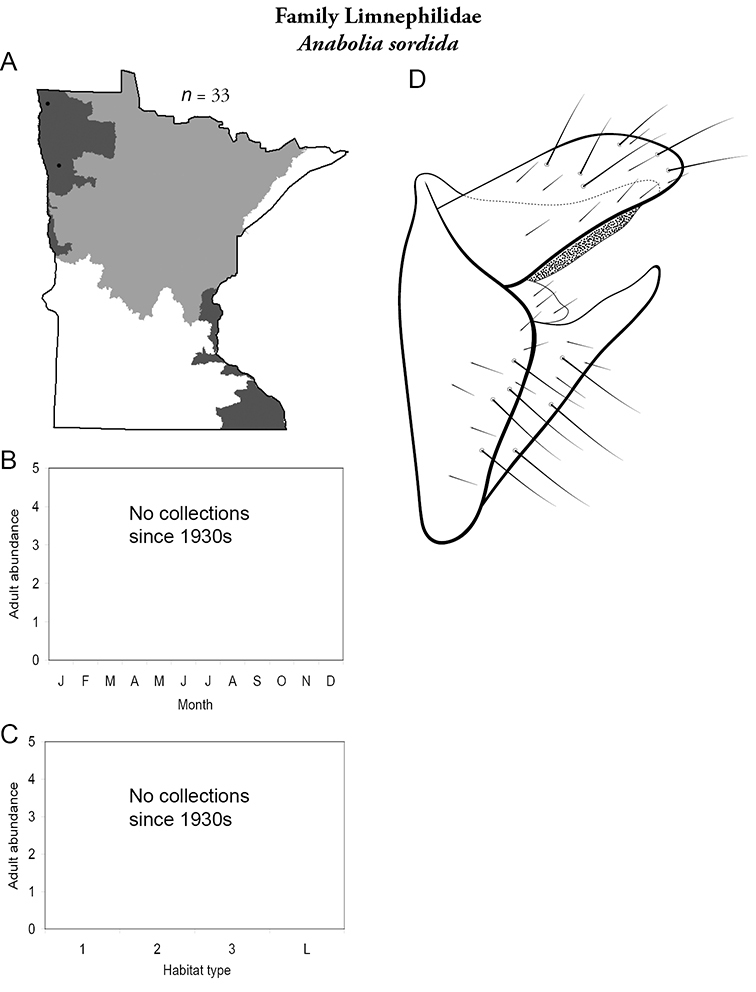
*Anabolia sordida* **A** total specimens collected and all known collecting localities (Figure 4) **B** monthly adult abundance (1980s to present) **C** habitat preference (1980s to present) (Table 1) **D** male genital capsule.

***Anabolia sordida*** (Figure 181) is known historically from 2 sites in Minnesota. Both of them are cities: Crookston and Hallock, in the Northwestern Region, and not associated with any particular habitat type. The species was collected 7 times in June, July, and August over a period from 1935 to 1937. It has not been seen in the state since this time despite extensive collecting in its known range, and appears to be extirpated. The loss of this species almost certainly has occurred due to habitat loss in the Northwestern Region since the 1930s (Houghton 2007, Houghton and Holzenthal 2010).

### Genus *Arctopora*

The genus *Arctopora* contains a single species in Minnesota. Little is known about larval biology except that they usually inhabit lakes and, sometimes, temporary pools (Wiggins 1996).

**Figure 182.**
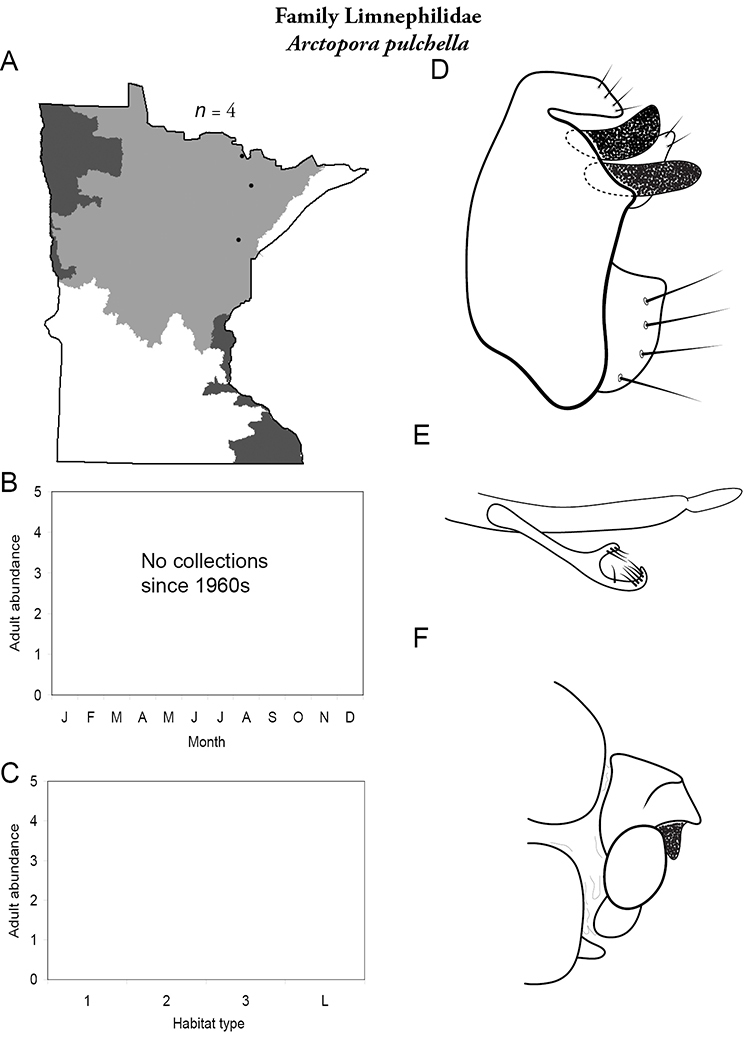
*Arctopora pulchella* **A** total specimens collected and all known collecting localities (Figure 4) **B** monthly adult abundance (1980s to present) **C** habitat preference (1980s to present) (Table 1) **D** male genital capsule **E** phallus **F** female genital capsule.

***Arctopora pulchella*** (Figure 182) is known only from 4 specimens in the Northern Region during July 1965. The species has not been collected since 1965 despite extensive collecting throughout its known range. The area known to support *Arctopora pulchella* has not been greatly disturbed since the 1960s, and so it is difficult to know if the species has been extirpated or is naturally rare and difficult to collect.

### Genus *Asynarchus*

The genus *Asynarchus* contains 3 species in Minnesota. One species appears to have decreased in its range, 1 is listed as “Threatened” by the Minnesota Department of Natural Resources (MNDNR 2012), and another appears to be extirpated from the state. Larvae are most frequently found in lakes and slow-moving areas of streams, although some species are exclusive to cold, fast-moving rivers. They usually consume decaying wood or other plant debris. Cases are usually composed of small mineral fragments (Wiggins 1996). Adults range 14–18 mm and are dark brown in color (Figure 293). For additional species, see Schmid (1954).

**Figure 183.**
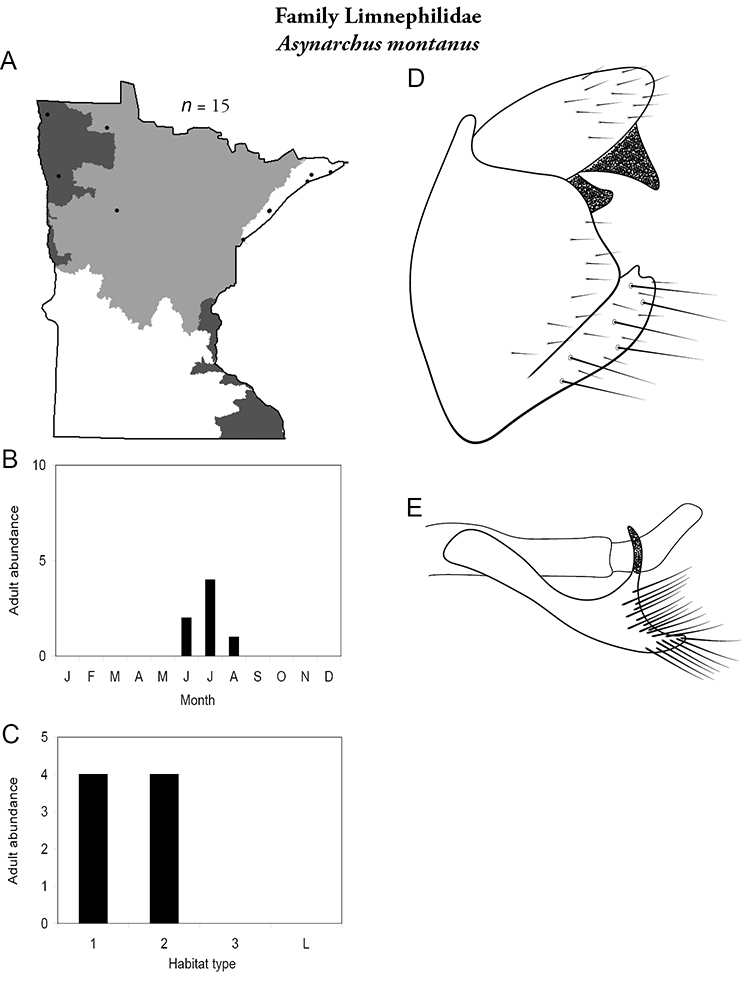
*Asynarchus montanu*s **A** total specimens collected and all known collecting localities (Figure 4) **B** monthly adult abundance (1980s to present) **C** habitat preference (1980s to present) (Table 1) **D** male genital capsule **E** phallus.

***Asynarchus montanus*** (Figure 183) is known historically from the Lake Superior, Northern, and Northwestern Regions. It has not been collected in the last region since the 1930s. Since the 1980s, it has been collected from small and medium streams from June through August.

**Figure 184.**
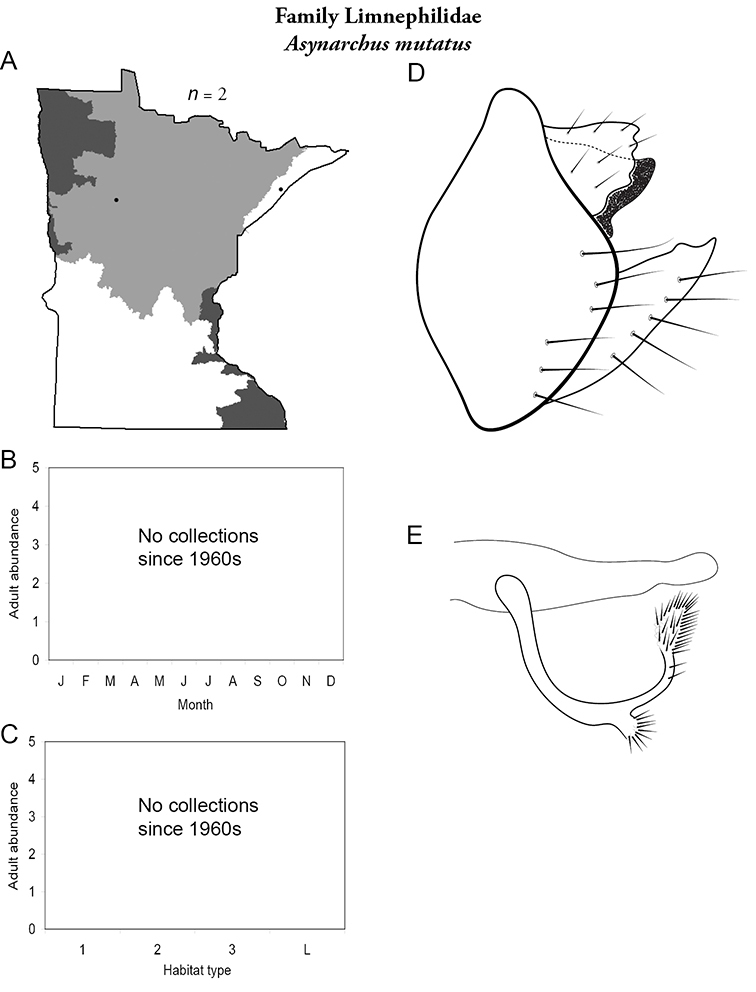
*Asynarchus mutatus* **A** total specimens collected and all known collecting localities (Figure 4) **B** monthly adult abundance (1980s to present) **C** habitat preference (1980s to present) (Table 1) **D** male genital capsule **E** phallus.

***Asynarchus mutatus*** (Figure 184) is known historically from the city of Finland in the Lake Superior Region, and Lake Itasca in the Northern Region. It has not been collected in the state since 1965. The species never appeared to be common, so it is difficult to know if it has been extirpated or is naturally rare and difficult to collect.

**Figure 185.**
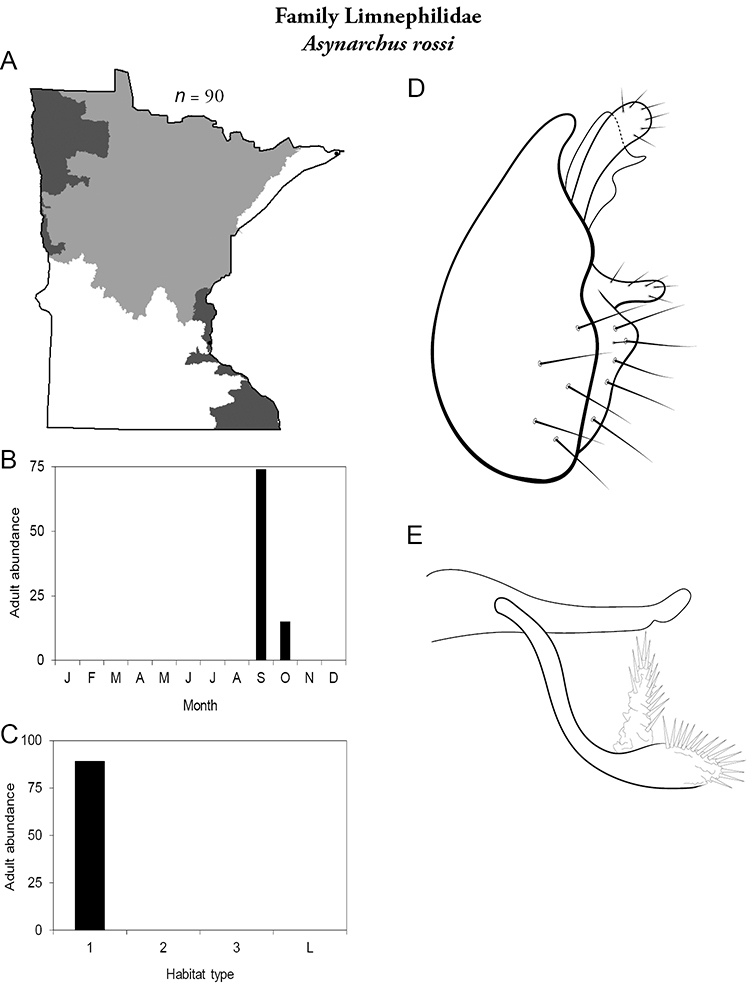
*Asynarchus rossi* **A** total specimens collected and all known collecting localities (Figure 4) **B** monthly adult abundance (1980s to present) **C** habitat preference (1980s to present) (Table 1) **D** male genital capsule **E** phallus.

***Asynarchus rossi*** (Figure 185) is the subject of systematic confusion, and is sometimes placed in the genus *Limnephilus* (Ruiter 1995). It is known in Minnesota from only 2 small streams: Grand Portage Creek, Cook County, in the Lake Superior Region, and Valley Creek, Washington County, in the Southeastern Region. It appears locally abundant in the latter location and has been collected there in 1995 and 1997. Adults were collected in September and October. Due to the rarity of *Asynarchus rossi* in Minnesota and the imminent threat of urban development around Valley Creek, the Minnesota Department of Natural Resources has proposed “Threatened” status for the species (MNDNR 2012).

### Genus *Chilostigma*

The genus *Chilostigma* contains a single species in Minnesota. The larva of the genus has yet to be definitively associated with the adult.The genus contains some of the smallest limnephilids, ranging 10–12 mm in length.

**Figure 186.**
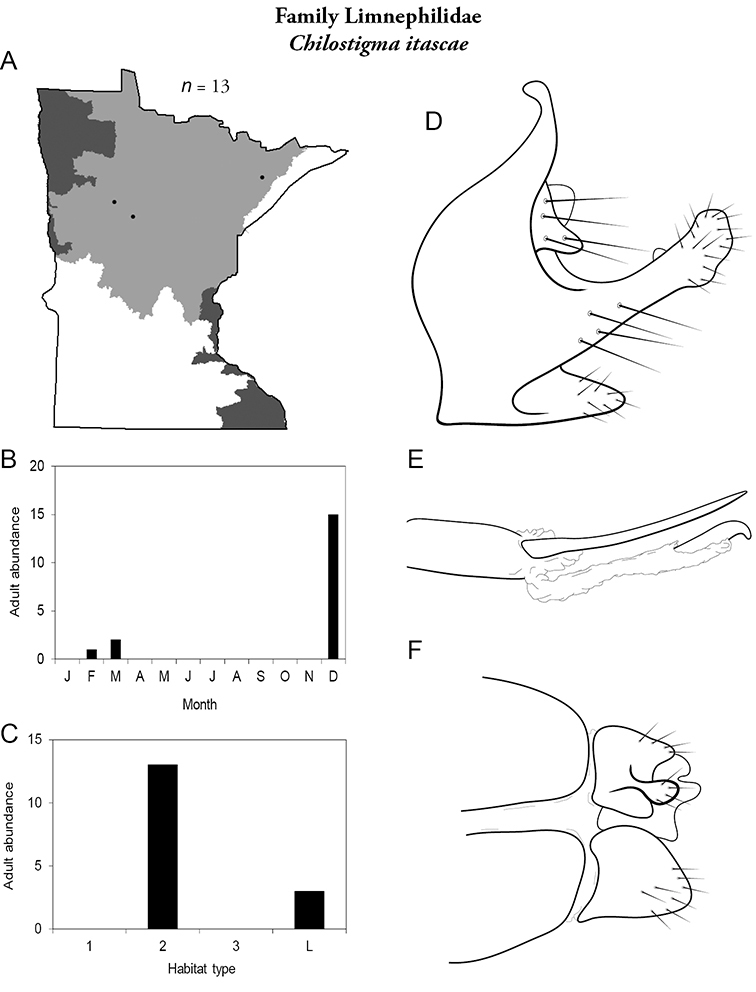
*Chilostigma itascae* **A** total specimens collected and all known collecting localities (Figure 4) **B** monthly adult abundance (1980s to present) **C** habitat preference (1980s to present) (Table 1) **D** male genital capsule **E** phallus **F** female genital capsule.

***Chilostigma itascae*** (Figure 186) is known worldwide from only 3 localities in the Northern Region. All three of these localities were either boggy, slow-moving areas of streams or else shallow lentic habitats. The species is unique in its seasonal emergence; all adults were found crawling on the surface of the snow on relatively warm days in winter and early spring. Although the species may be more common than reported due to its winter emergence, the Minnesota specimens remain the only known examples of *Chilostigma itascae* in the world. Thus, due to its extreme rarity and Minnesota endemism, *Chilostigma itascae* is listed as “Endangered” by the Minnesota Department of Natural Resources (MNDNR 2012).

### Genus *Frenesia*

The genus *Frenesia* contains a single species in Minnesota. Larvae inhabit cold streams and feed on decaying wood and leaves (Wiggins 1996). Cases are composed primarily of small mineral particles. Adult forewings are pale orange in color, with darker reticulations (Figure 293). Adults length ranges 18–22 mm.

**Figure 187.**
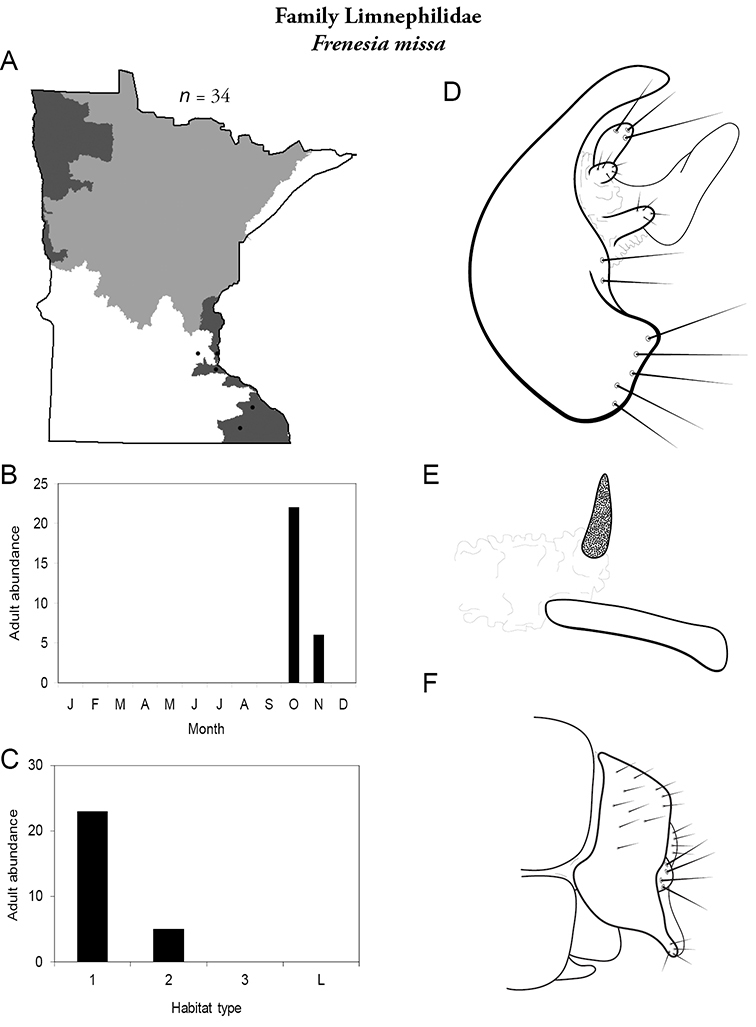
*Frenesia missa* **A** total specimens collected and all known collecting localities (Figure 4) **B** monthly adult abundance (1980s to present) **C** habitat preference (1980s to present) (Table 1) **D** male genital capsule **E** phallus **F** female genital capsule.

***Frenesia missa*** (Figure 187) is known only from small and medium streams of the Southeastern and Southern Regions. It is unique in its late fall emergence; adults were collected in October and November. It is possible that the species is more common than it appears due to its presence when few collectors are out.

### Genus *Glyphopsyche*

The genus *Glyphopsyche* contains a single species from Minnesota. For additional species, see Schmid (1952a). Larvae typically inhabit marshes, small lakes, and slow-moving areas of streams where they feed on decaying wood and other organic matter (Wiggins 1996). Cases are usually a mixture of mineral and organic fragments.

**Figure 188.**
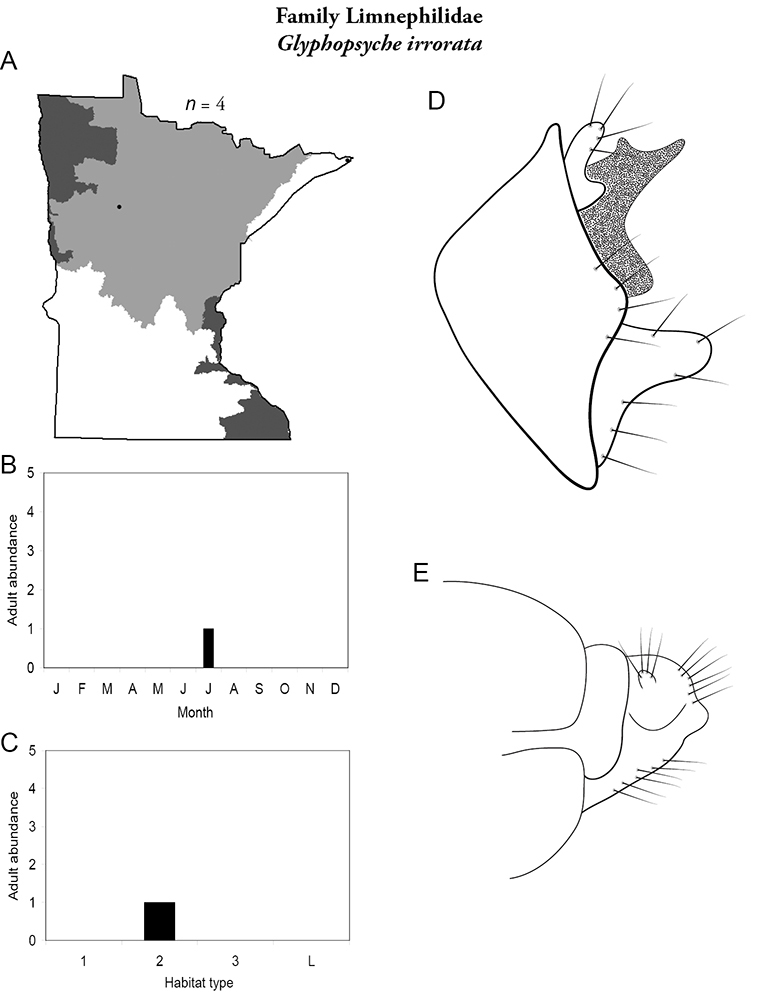
*Glyphopsyche irrorata* **A** total specimens collected and all known collecting localities (Figure 4) **B** monthly adult abundance (1980s to present) **C** habitat preference (1980s to present) (Table 1) **D** male genital capsule **E** female genital capsule.

***Glyphopsyche irrorata*** (Figure 188) is known only from Grand Portage Creek, Cook County, in the Lake Superior Region, and Lake Itasca, Clearwater County, in the Northern Region. The former collection occurred in July 2000 and the latter in July 1977. The species is unique in sometimes overwintering in the adult stage (Berte and Pritchard 1983).

### Genus *Grammotaulius*

The genus *Grammotaulius* contains a single species in Minnesota. For additional species, see Schmid (1950b). Larvae typically inhabit ponds or slow-moving areas of streams. Little else is known about their biology. Larval cases are composed of slender pieces of sedges or leaves arranged lengthwise (Wiggins 1996). Adults are 12–14 mm in length with wings of gold and red reticulations.

**Figure 189.**
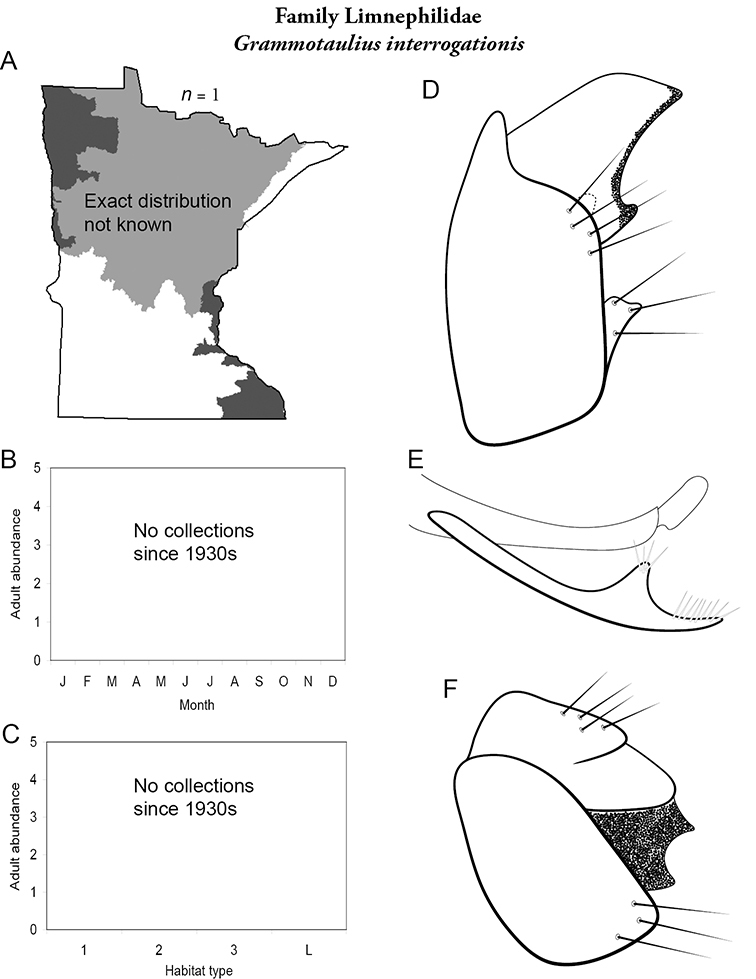
*Grammotaulius interrogationis* **A** total specimens collected and all known collecting localities (Figure 4) **B** monthly adult abundance (1980s to present) **C** habitat preference (1980s to present) (Table 1) **D** male genital capsule **E** phallus **F** female genital capsule.

***Grammotaulius interrogationis*** (Figure 189) is known only from a single specimen collected from “Cook County” in July 1938. The species has not been collected in Minnesota since then. The majority of Cook County is in the Lake Superior Region, with some in the Northern Region. Minnesota is the most southern collecting locality for the species, which occurs as far north as Greenland (Schmid 1950a).

### Genus *Hesperophylax*

The genus *Hesperophylax* contains a single species from Minnesota. For additional species, see Parker and Wiggins (1985). Larvae inhabit a wide range of streams types where they feed mostly on detritus, but may also include algae, diatoms, vascular plants, and arthropods (Parker and Wiggins 1985, Wiggins 1996). Larval cases are primarily composed of small mineral particles. Adults range 18–22 mm in length. They are strikingly colorful, with forewings of gold, yellow and brown patterning (Figure 293).

**Figure 190.**
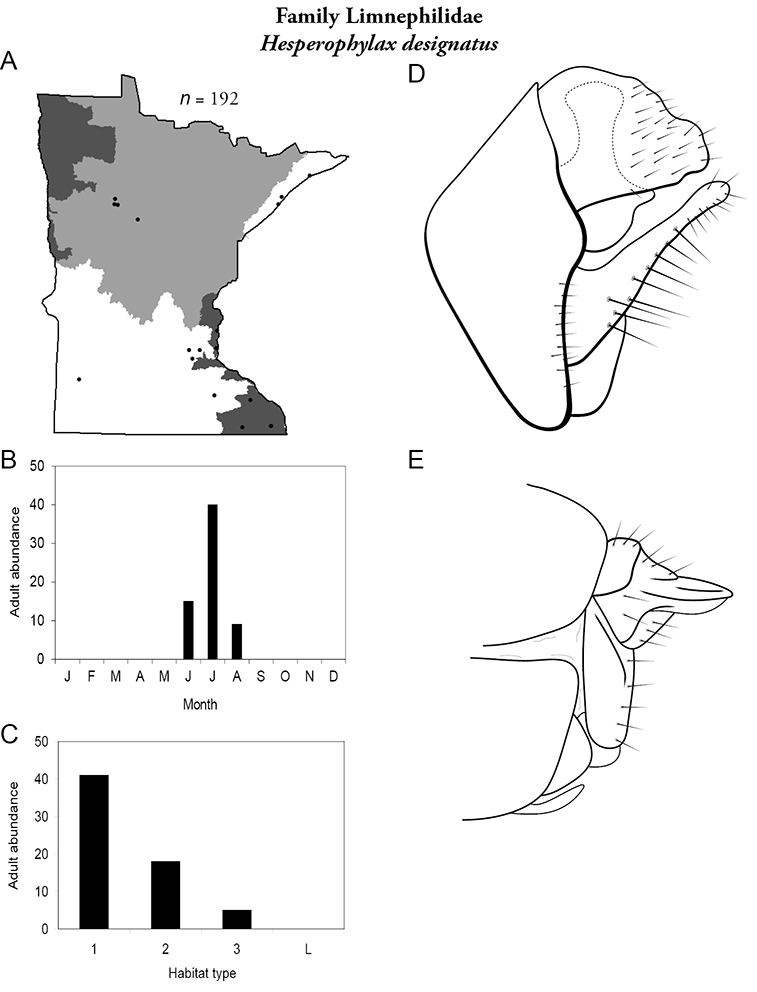
*Hesperophylax designatus* **A** total specimens collected and all known collecting localities (Figure 4) **B** monthly adult abundance (1980s to present) **C** habitat preference (1980s to present) (Table 1) **D** male genital capsule **E** female genital capsule.

***Hesperophylax designatus*** (Figure 190) has been collected from all regions except the Northwestern, but was most abundant in the southeast portion of the state. It is known primarily from medium and, especially, small streams. Adults have been collected from June through August.

### Genus *Hydatophylax*

The genus *Hydatophylax* contains a single species from Minnesota. For additional species, see Schmid (1950c). Larvae are most common in submerged piles of plant debris within small streams. Larvae consume wood or other decaying organic matter. Larval cases are composed mostly of large and irregular pieces of wood, bark, and leaves (Wiggins 1996). Cases can be near 80 mm in length. Adults are some of the largest of caddisflies, ranging up to 40 mm in length. Forewings are bright white with dark veins and reticulations.

**Figure 191.**
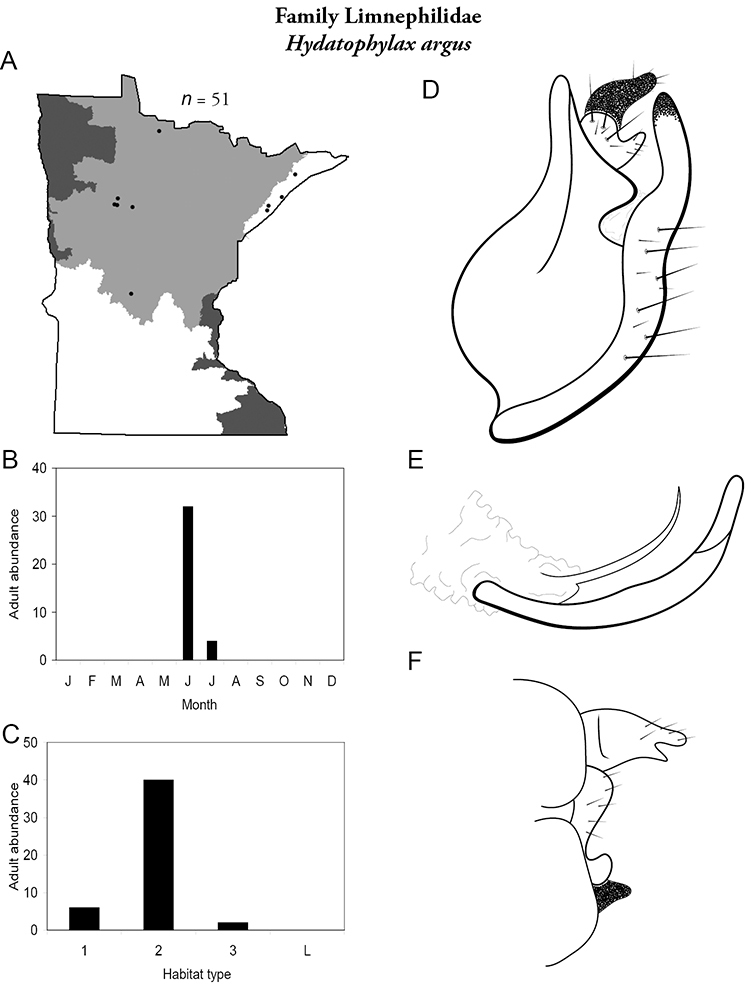
*Hydatophylax argus* **A** total specimens collected and all known collecting localities (Figure 4) **B** monthly adult abundance (1980s to present) **C** habitat preference (1980s to present) (Table 1) **D** male genital capsule **E** phallus **F** female genital capsule.

***Hydatophylax argus*** (Figure 191) is known from the Lake Superior and Northern regions. Adults were collected mainly in June and mostly from medium rivers.

### Genus *Ironoquia*

The genus *Ironoquia* contains 2 species from Minnesota. For additional species, see Schmid (1951). Larvae can inhabit a variety of habitats, from small springs to temporary pools, depending on the species (Ross 1944, Wiggins 1996). Larvae consume mainly algae and vascular plants. Larval cases are composed of tightly-packed pieces of wood and bark.

**Figure 192.**
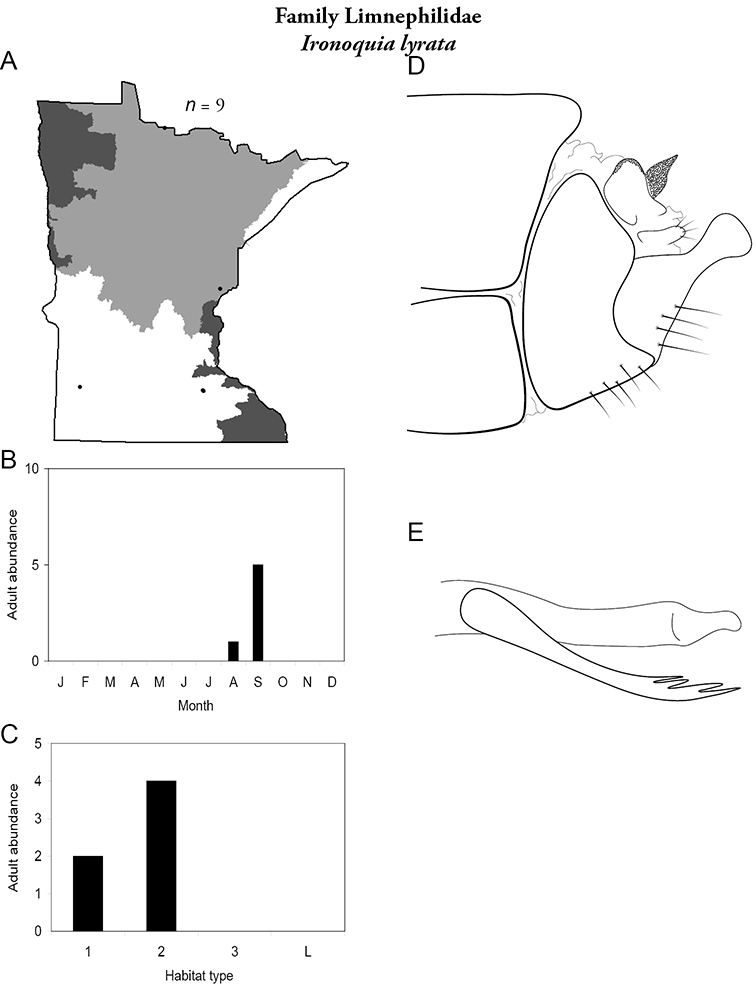
*Ironoquia lyrata* **A** total specimens collected and all known collecting localities (Figure 4) **B** monthly adult abundance (1980s to present) **C** habitat preference (1980s to present) (Table 1) **D** male genital capsule **E** phallus.

***Ironoquia lyrata*** (Figure 192) has been collected from small and medium streams of the Northern and Southern Regions. Adults were present mainly in September.

**Figure 193.**
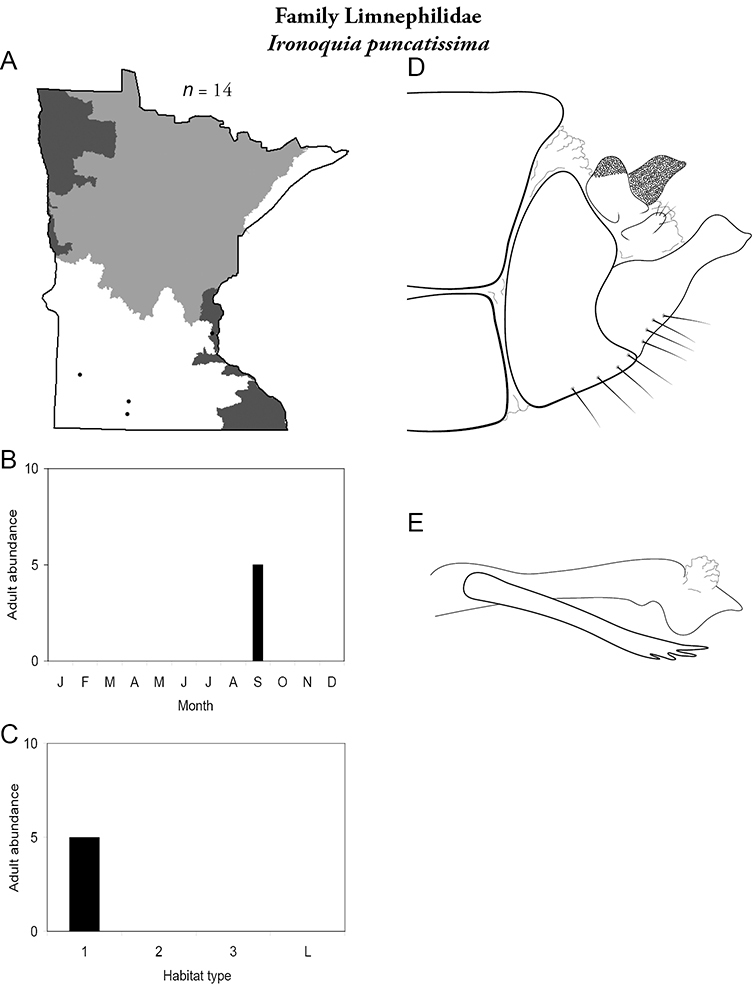
*Ironoquia punctatissima* **A** total specimens collected and all known collecting localities (Figure 4) **B** monthly adult abundance (1980s to present) **C** habitat preference (1980s to present) (Table 1) **D** male genital capsule **E** phallus.

***Ironoquia puncatissima*** (Figure 193) is only known from small streams, mainly in the Southern Region. Adults were caught in September. Undisturbed small streams are extremely rare in the Southern Region and are vulnerable to urban and agricultural development (Houghton 2007). Due to the rarity of this species and the rarity and vulnerability of suitable habitats, the Minnesota Department of Natural Resources has proposed “Threatened” status for *Ironoquia puncatissima*(MNDNR 2012).

### Genus *Lenarchus*

The genus *Lenarchus* contains 2 species from Minnesota. Both are very rare. For additional species, see Schmid (1952b). Larvae typically inhabit small lakes, marshes, and slow-moving areas of streams where they feed mainly on detritus (Wiggins 1996). Larval cases may include pieces of sedges arranged longitudinally.

**Figure 194.**
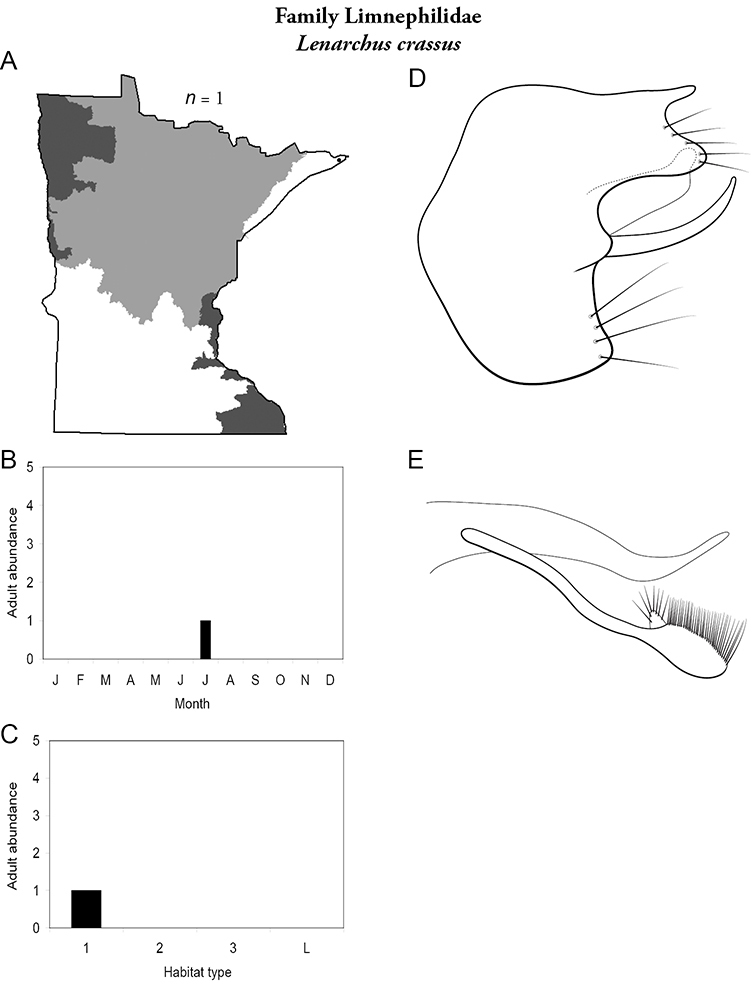
*Lenarchus crassus* **A** total specimens collected and all known collecting localities (Figure 4) **B** monthly adult abundance (1980s to present) **C** habitat preference (1980s to present) (Table 1) **D** male genital capsule **E** phallus.

***Lenarchus crassus*** (Figure 194) is known only from an unnamed spring near Grand Portage National Monument in the Lake Superior Region collected during July 2000.

**Figure 195.**
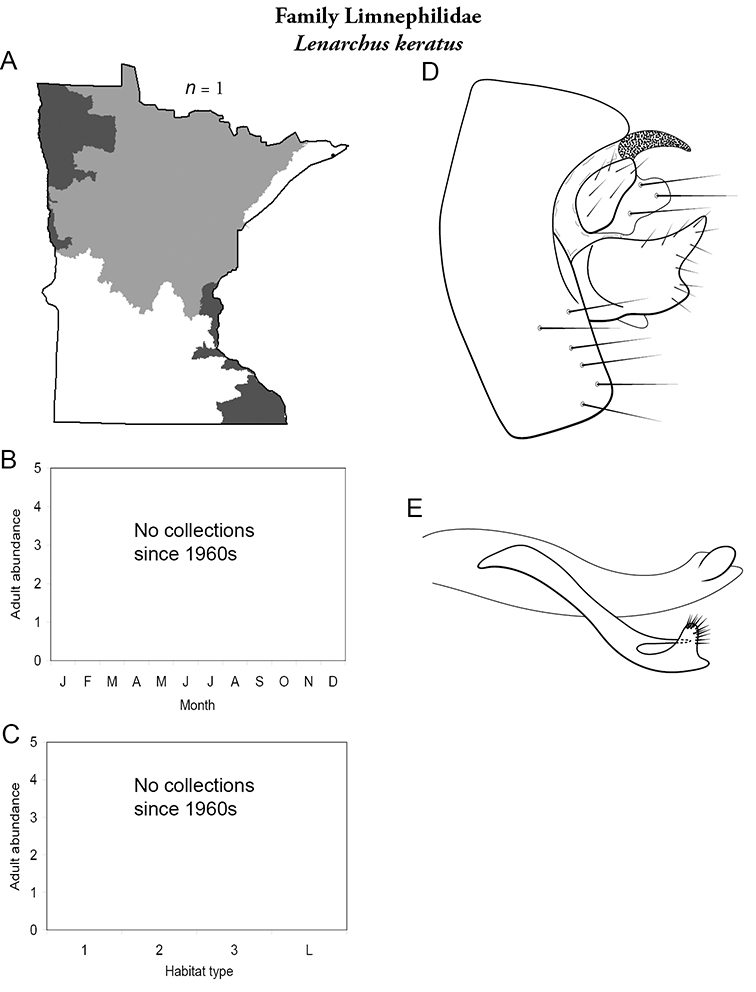
*Lenarchus keratus* **A** total specimens collected and all known collecting localities (Figure 4) **B** monthly adult abundance (1980s to present) **C** habitat preference (1980s to present) (Table 1) **D** male genital capsule **E** phallus.

***Lenarchus keratus*** (Figure 195) is known only from the City of Hovland in the Lake Superior Region. A single specimen was collected in 1965. It has not been found in Minnesota since this collection.

### Genus *Leptophylax*

The genus *Leptophylax* contains a single species in North America and in Minnesota. Larvae have yet to be positively associated with the adults. Adults are brown in color and 10–14 mm in length.

**Figure 196.**
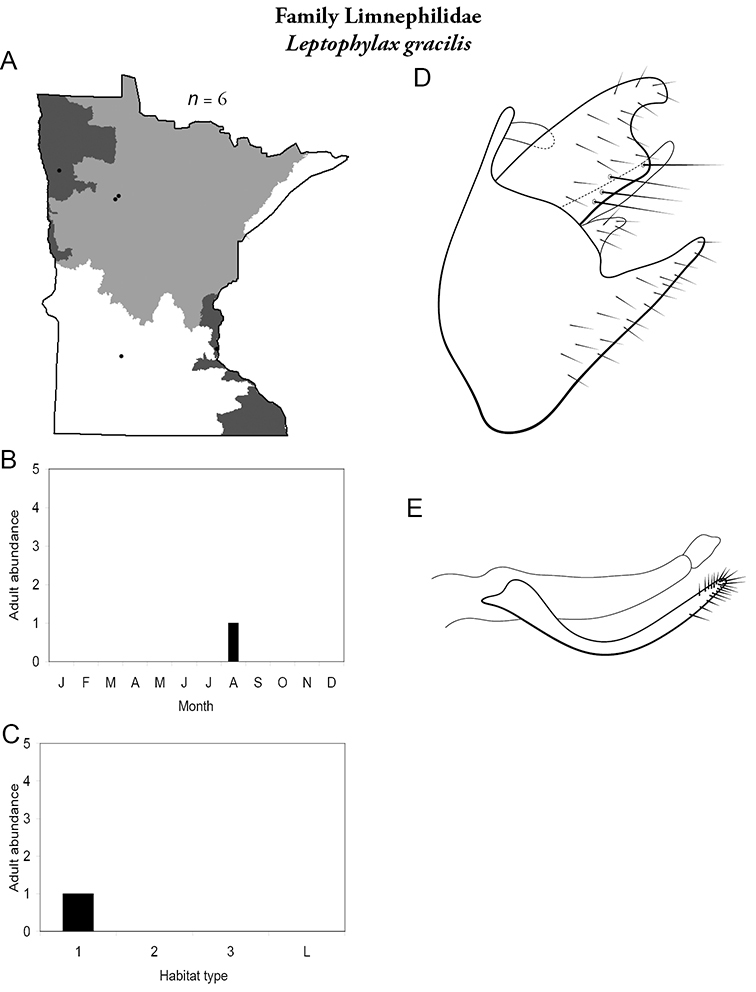
*Leptophylax gracilis* **A** total specimens collected and all known collecting localities (Figure 4) **B** monthly adult abundance (1980s to present) **C** habitat preference (1980s to present) (Table 1) **D** male genital capsule **E** phallus.

***Leptophylax gracilis*** (Figure 196) has been collected from all regions except the Lake Superior. All collections except 1, however, occurred in the 1940s or earlier. The most recent collection occurred from an unnamed tributary of the Mississippi River, Hubbard County, in the Northern Region during August 1989.

### Genus *Limnephilus*

The genus *Limnephilus* contains 19 species from Minnesota. It is the 2nd most species-rich genus in the state. The genus contains around 100 species in North America (Ruiter 1995) and is difficult to characterize. Most larvae are lentic, although many occur in different types of streams. Larvae are typically detritivores or shredders (Wiggins 1996). Larval cases can be composed of nearly any combination of organic or mineral particles depending on the species. Adults are typically 15–20 mm in length. Most species are either tan or dark grey, sometimes with darker reticulations on the forewings (Figure 293–294). Some species, especially *Limnephilus hyalinus* and *Limnephilus ornatus* have striking patterns of yellow and orange on their forewings.

Many *Limnephilus* species historically common throughout the state are now restricted to the Lake Superior and Northern Regions. Several others are known in Minnesota exclusively from 1 or few specimens collected in the 1960s or earlier. For additional species, see Ruiter (1995).

**Figure 197.**
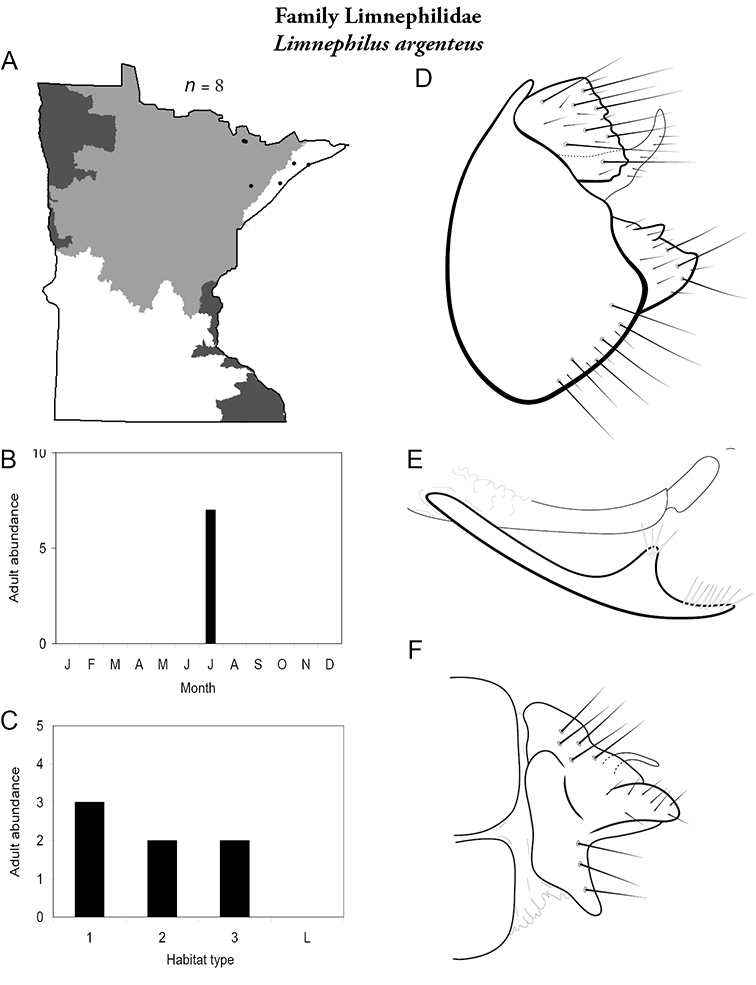
*Limnephilus argenteus* **A** total specimens collected and all known collecting localities (Figure 4) **B** monthly adult abundance (1980s to present) **C** habitat preference (1980s to present) (Table 1) **D** male genital capsule **E** phallus **F** female genital capsule.

***Limnephilus argenteus*** (Figure 197) in known exclusively from all sizes of streams in July. It has been found in or near the Lake Superior Region, but was not abundant.

**Figure 198.**
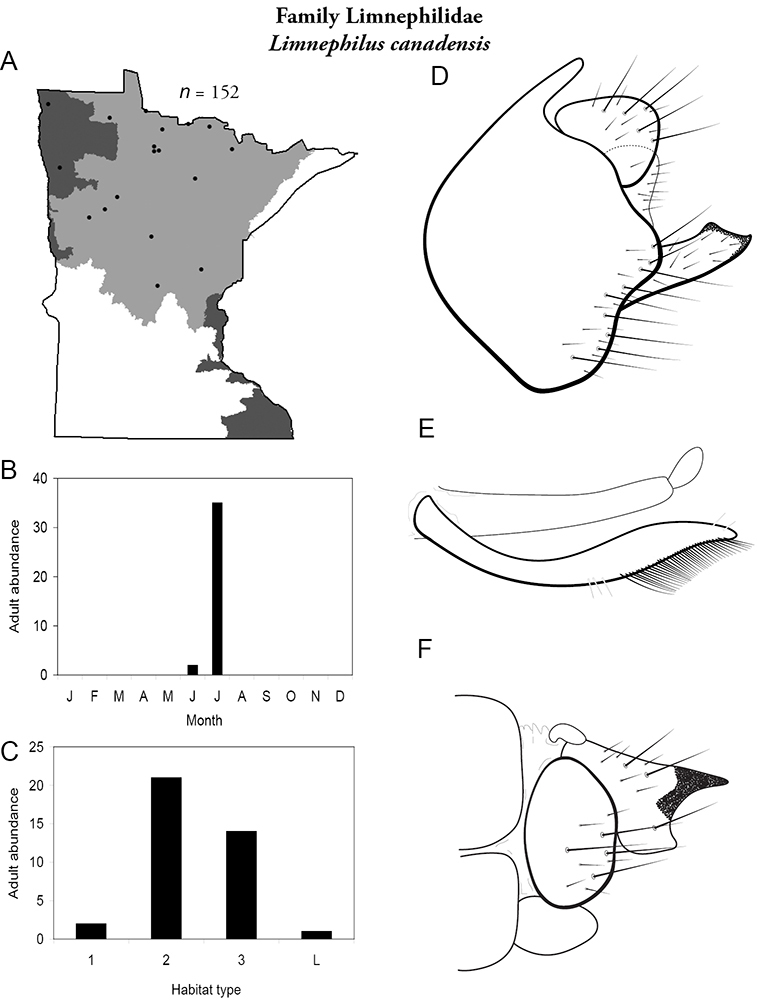
*Limnephilus canadensis* **A** total specimens collected and all known collecting localities (Figure 4) **B** monthly adult abundance (1980s to present) **C** habitat preference (1980s to present) (Table 1) **D** male genital capsule **E** phallus **F** female genital capsule.

***Limnephilus canadensis*** (Figure 198) has been historically collected throughout the Northern and Northwestern Regions. All specimens since the 1930s, however, have come from the Northern Region. Adults were most common in July, and were found primarily in medium and large rivers.

**Figure 199.**
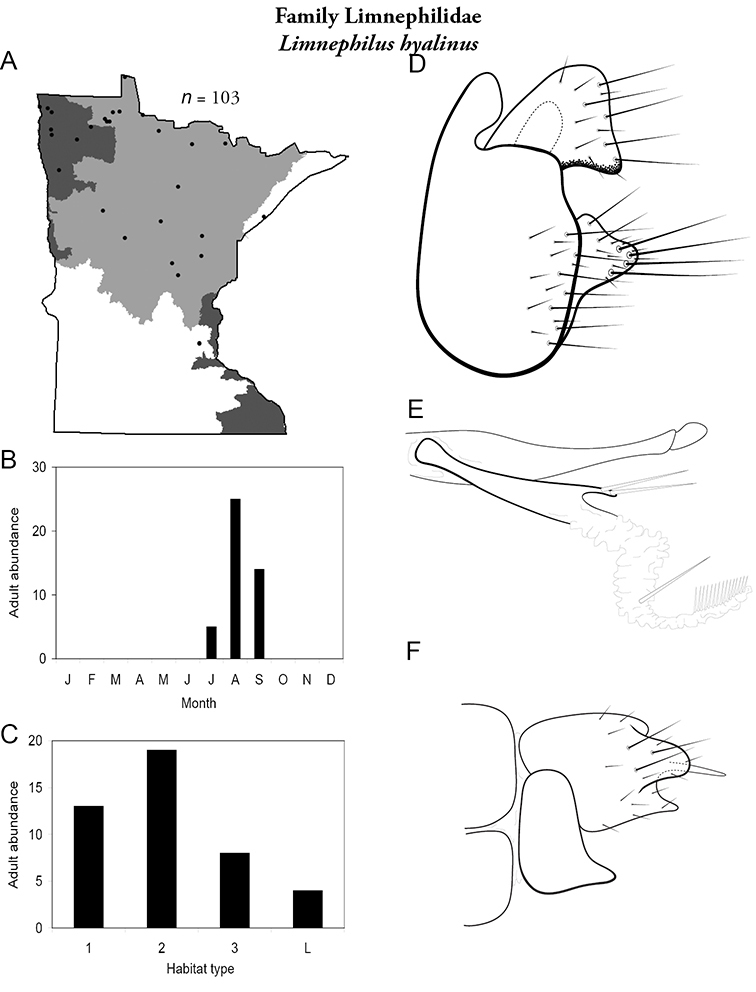
*Limnephilus hyalinus* **A** total specimens collected and all known collecting localities (Figure 4) **B** monthly adult abundance (1980s to present) **C** habitat preference (1980s to present) (Table 1) **D** male genital capsule **E** phallus **F** female genital capsule.

***Limnephilus hyalinus*** (Figure 199) is known primarily from the Northern and Northwestern regions, with sporadic collections from other regions. It is one of the few limnephilids to still be found in the Northwestern Region. Adults were present from July to September. It was collected from all habitat types, especially medium rivers.

**Figure 200.**
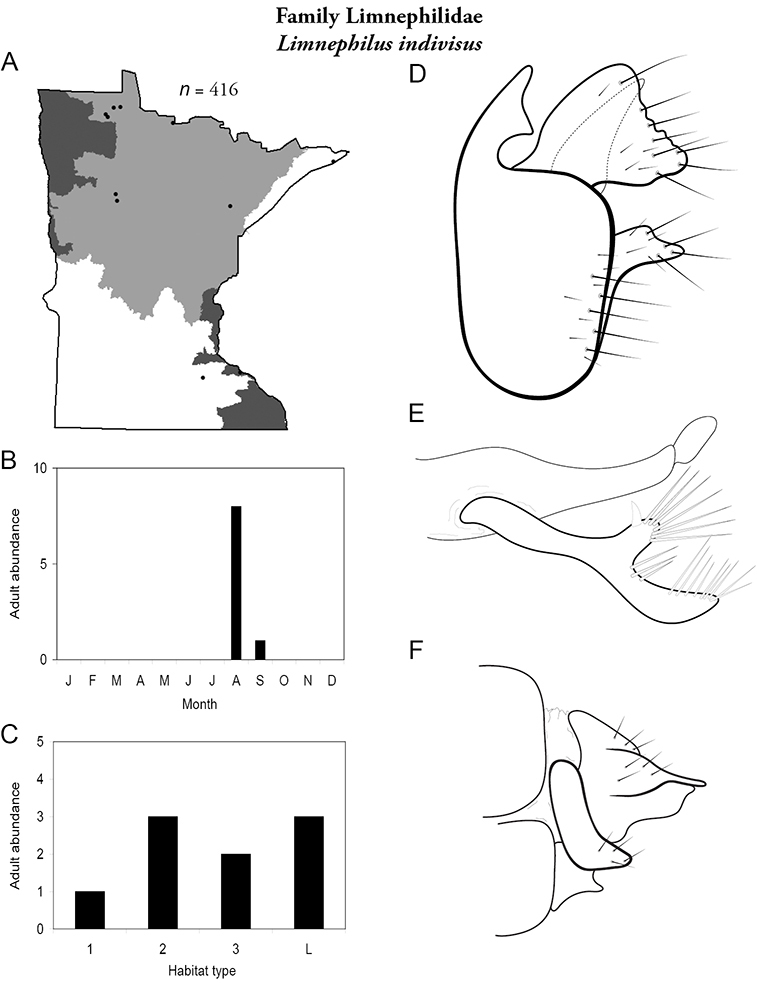
*Limnephilus indivisus* **A** total specimens collected and all known collecting localities (Figure 4) **B** monthly adult abundance (1980s to present) **C** habitat preference (1980s to present) (Table 1) **D** male genital capsule **E** phallus **F** female genital capsule.

***Limnephilus indivisus*** (Figure 200) has been found sporadically in all regions of the state. It has, however, been collected only in the Northern Region since the 1960s. Further, nearly 98% of total specimens were collected prior to the 1970s, suggesting that the species is decreasing in abundance through out the state. The few recent specimens were collected from all habitat types, mainly during August.

**Figure 201.**
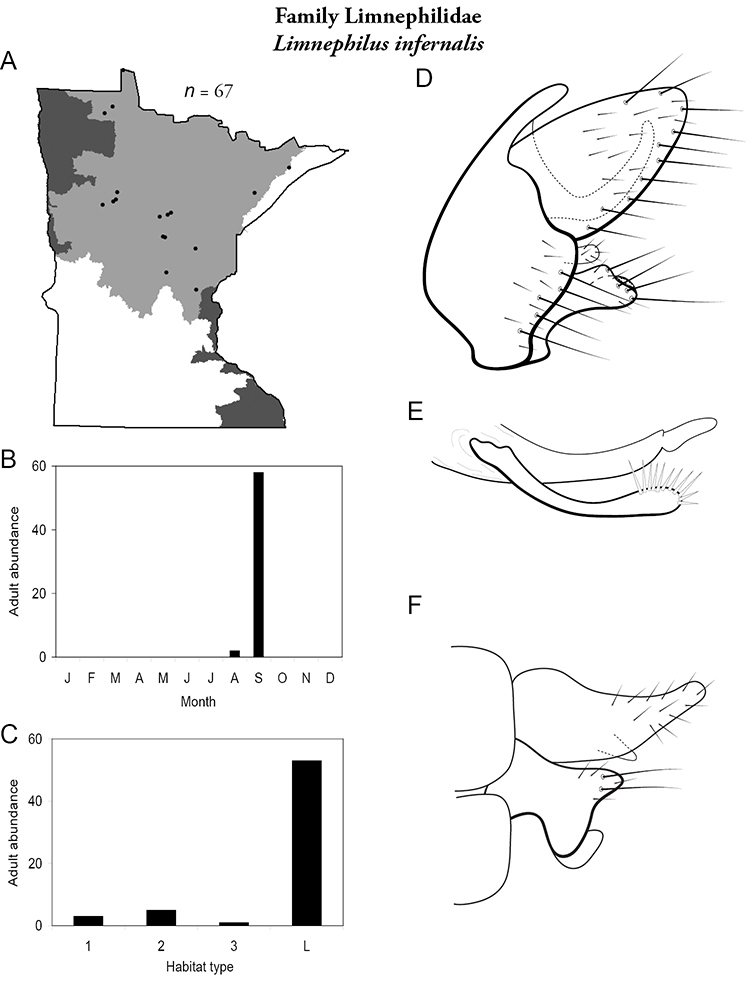
*Limnephilus infernalis* **A** total specimens collected and all known collecting localities (Figure 4) **B** monthly adult abundance (1980s to present) **C** habitat preference (1980s to present) (Table 1) **D** male genital capsule **E** phallus **F** female genital capsule.

***Limnephilus infernalis*** (Figure 201) has been collected from throughout the Northern Region, mostly from lakes during September.

**Figure 202.**
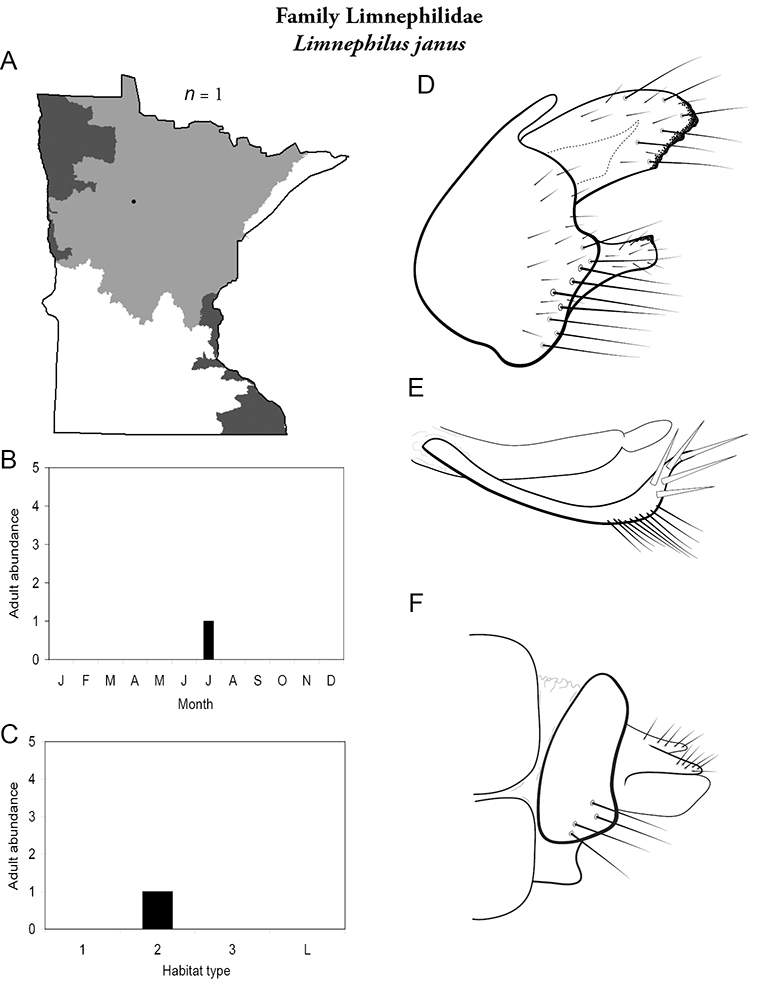
*Limnephilus janus* **A** total specimens collected and all known collecting localities (Figure 4) **B** monthly adult abundance (1980s to present) **C** habitat preference (1980s to present) (Table 1) **D** male genital capsule **E** phallus **F** female genital capsule.

***Limnephilus janus*** (Figure 202) has been collected only from Little Elbow Creek, Mahnomen County, in the Northern Region during July 2000. Due to the rarity of this species, the Minnesota Department of Natural Resources has proposed “Threatened” status for *Limnephilus janus* (MNDNR 2012).

**Figure 203.**
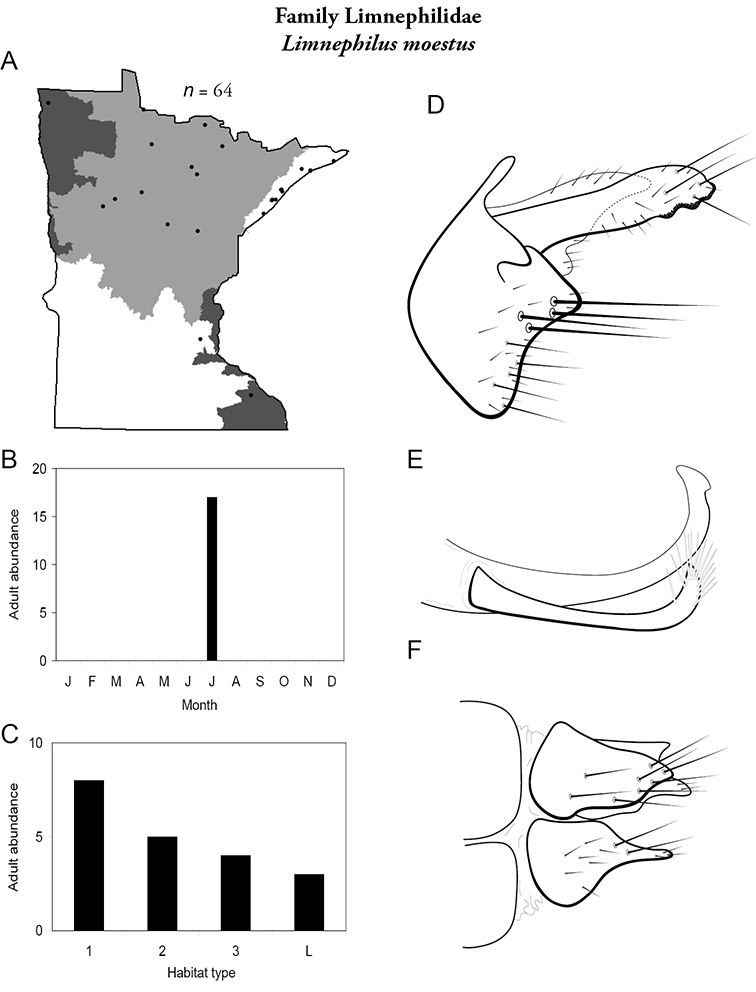
*Limnephilus moestus* **A** total specimens collected and all known collecting localities (Figure 4) **B** monthly adult abundance (1980s to present) **C** habitat preference (1980s to present) (Table 1) **D** male genital capsule **E** phallus **F** female genital capsule.

***Limnephilus moestus*** (Figure 203) has been collected only from the Lake Superior and Northern Regions since the 1960s, with some previous collections in the other regions of the state. It was found in all habitat types, exclusively during July.

**Figure 204.**
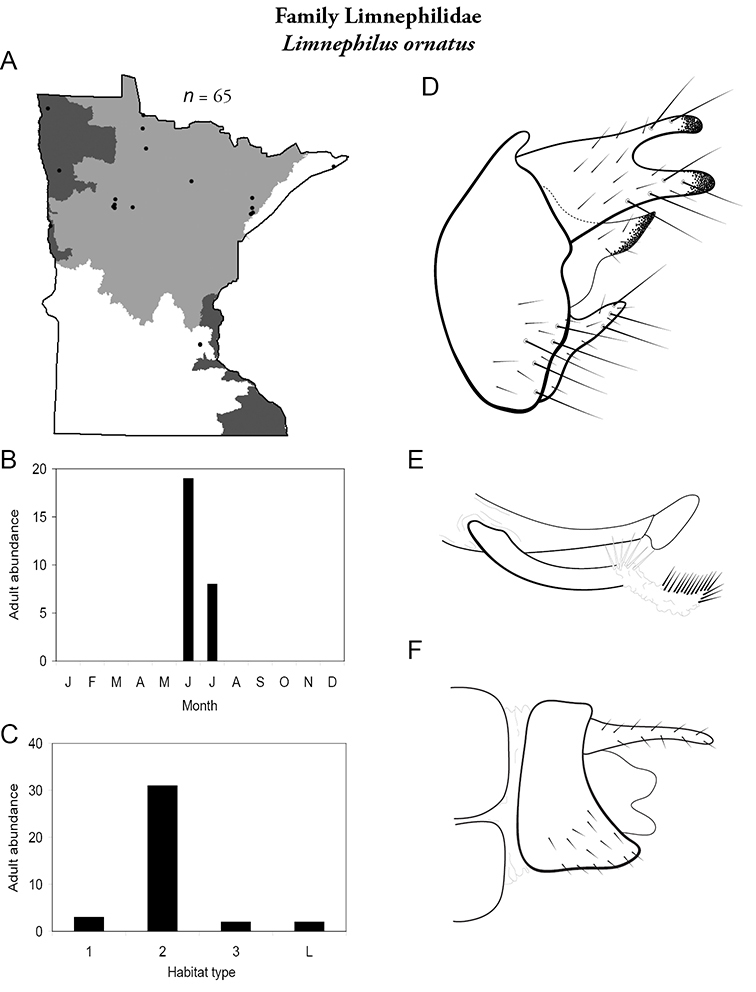
*Limnephilus ornatus* **A** total specimens collected and all known collecting localities (Figure 4) **B** monthly adult abundance (1980s to present) **C** habitat preference (1980s to present) (Table 1) **D** male genital capsule **E** phallus **F** female genital capsule.

***Limnephilus ornatus*** (Figure 204) has been historically found in all regions except the Southeastern. Since the 1960s, however, it is only known from the Lake Superior and Northern Regions. It has been collected mostly from medium rivers in June and July.

**Figure 205.**
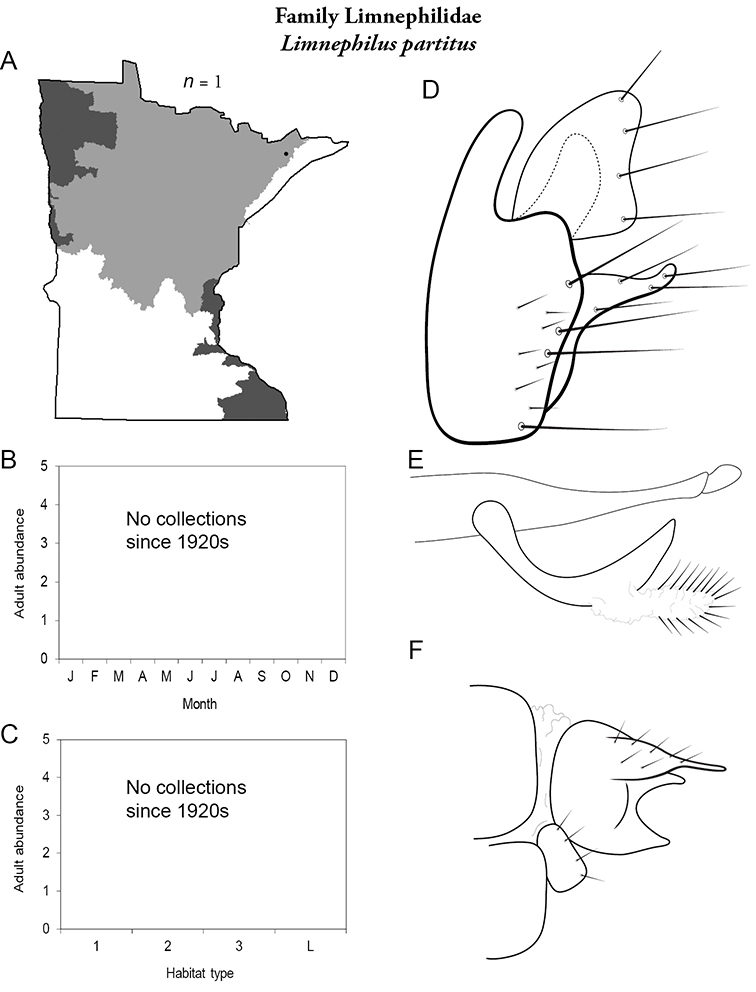
*Limnephilus partitus* **A** total specimens collected and all known collecting localities (Figure 4) **B** monthly adult abundance (1980s to present) **C** habitat preference (1980s to present) (Table 1) **D** male genital capsule **E** phallus **F** female genital capsule.

***Limnephilus partitus*** (Figure 205) is known only from a single specimen collected from an unknown locality on the Kawishiwi River in the Lake Superior Region. The locality on the species’ distribution map is, thus, an approximation. The date of the collection is also unknown. The collector of the specimen, R.L. Knight, accessioned specimens into the UMSP during the 1920s. Thus, it is presumed that the specimen was collected during this period. The species has not been collected since.

**Figure 206.**
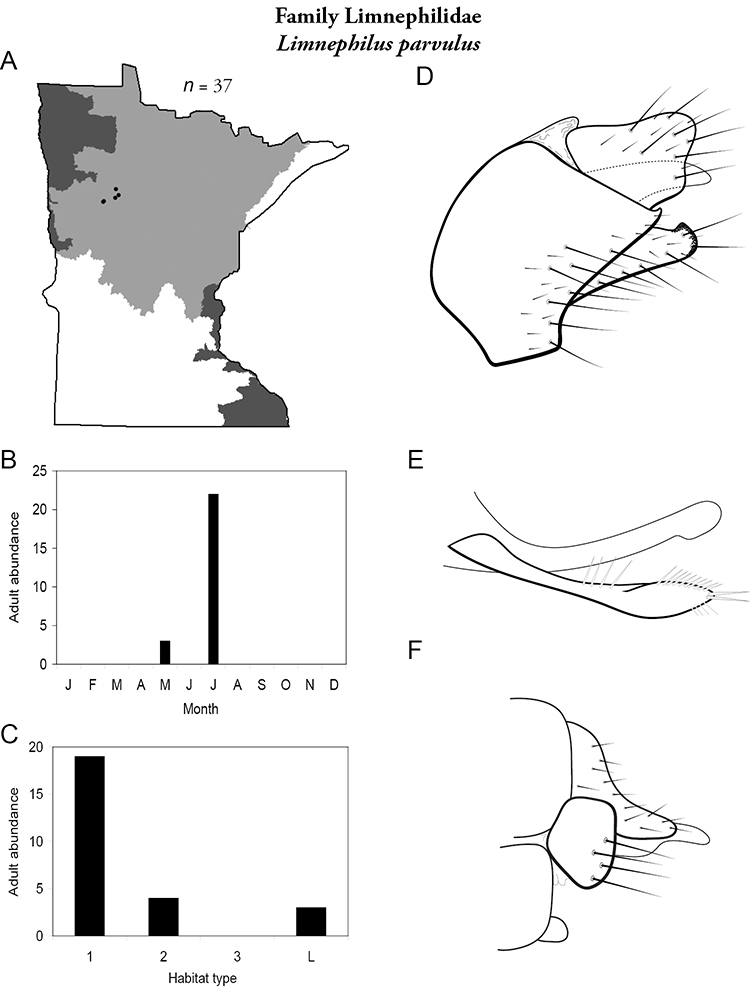
*Limnephilus parvulus* **A** total specimens collected and all known collecting localities (Figure 4) **B** monthly adult abundance (1980s to present) **C** habitat preference (1980s to present) (Table 1) **D** male genital capsule **E** phallus **F** female genital capsule.

***Limnephilus parvulus*** (Figure 206) is known only from sites in Becker and Clearwater Counties in the Northern Region. Some adults were present in May; most were collected in July. It was found in all habitats except large rivers, and was most abundant in small streams.

**Figure 207.**
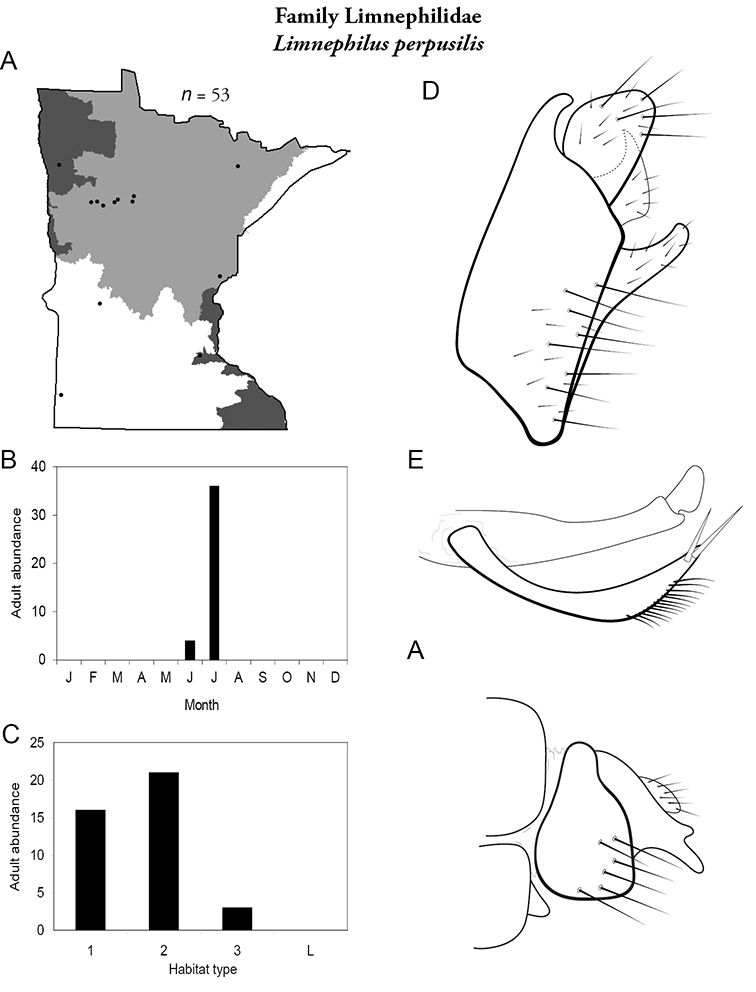
*Limnephilus perpusillis* **A** total specimens collected and all known collecting localities (Figure 4) **B** monthly adult abundance (1980s to present) **C** habitat preference (1980s to present) (Table 1) **D** male genital capsule **E** phallus **F** female genital capsule.

***Limnephilus perpusilis*** (Figure 207) is only known from the Northern Region since the 1960s, although it has been collected historically from all regions except the Lake Superior. It was found predominantly in small and medium streams. Adults were present mainly in July.

**Figure 208.**
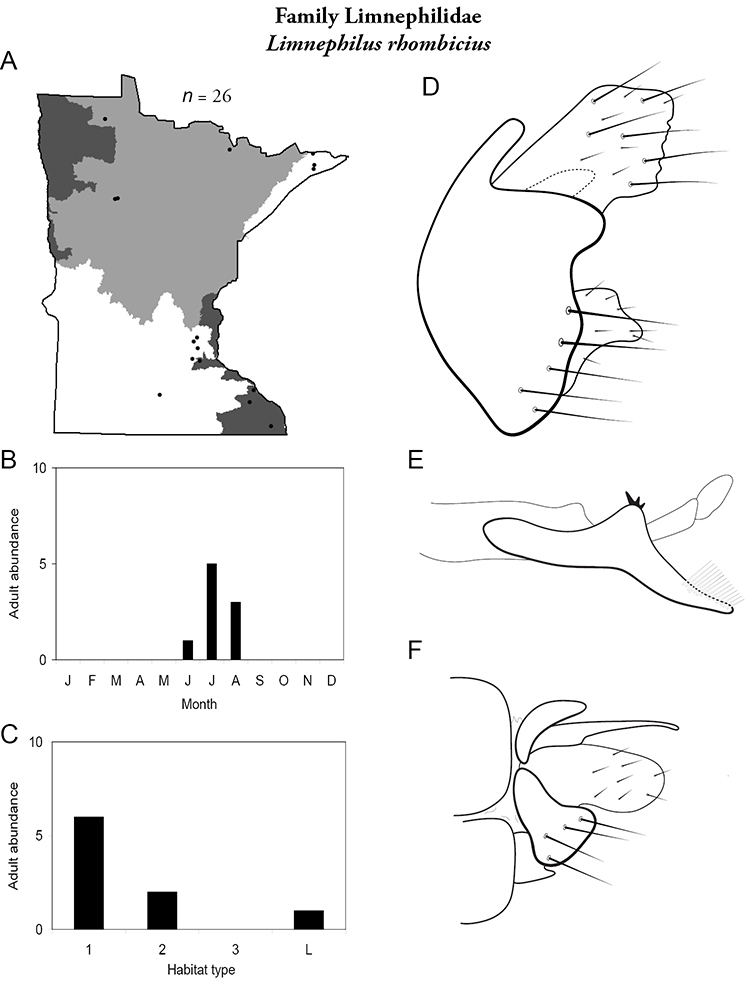
*Limnephilus rhombicius* **A** total specimens collected and all known collecting localities (Figure 4) **B** monthly adult abundance (1980s to present) **C** habitat preference (1980s to present) (Table 1) **D** male genital capsule **E** phallus **F** female genital capsule.

***Limnephilus rhombicius*** (Figure 208) has been found sporadically from throughout the state, although all collections since the 1960s have occurred in the Lake Superior and Northern Regions. It was present in small and medium streams as well as lakes. Adults were present from June through August.

**Figure 209.**
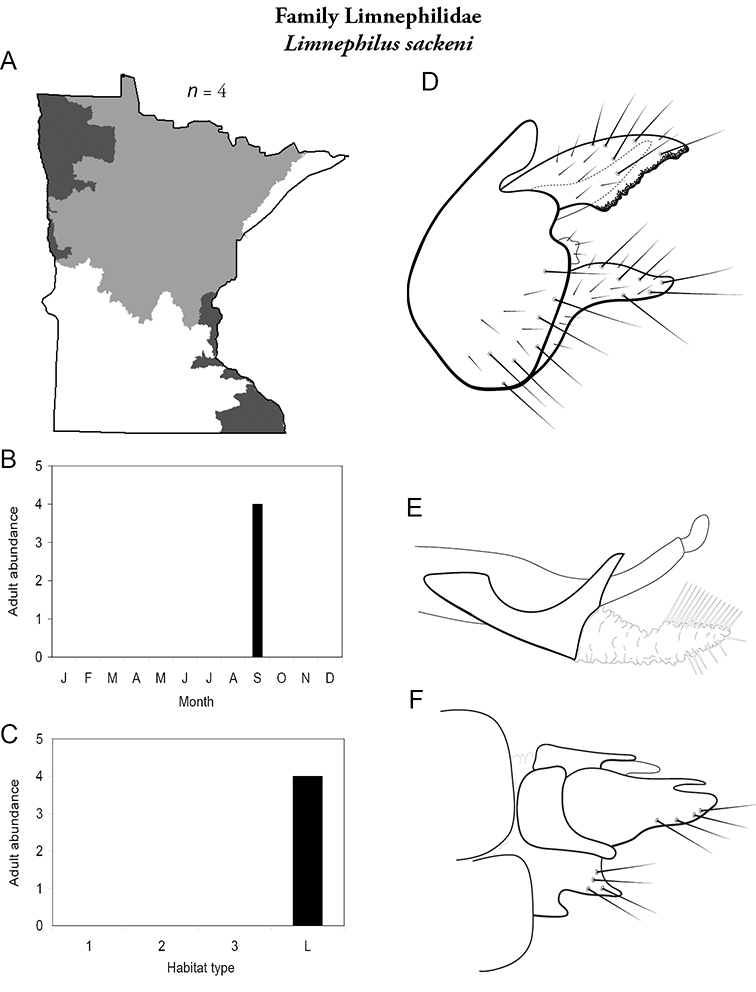
*Limnephilus sackeni* **A** total specimens collected and all known collecting localities (Figure 4) **B** monthly adult abundance (1980s to present) **C** habitat preference (1980s to present) (Table 1) **D** male genital capsule **E** phallus **F** female genital capsule.

***Limnephilus sackeni*** (Figure 209) is known only from 4 specimens collected from Lake of the Woods, Lake of the Woods County, in the Northern Region during September 1999.

**Figure 210.**
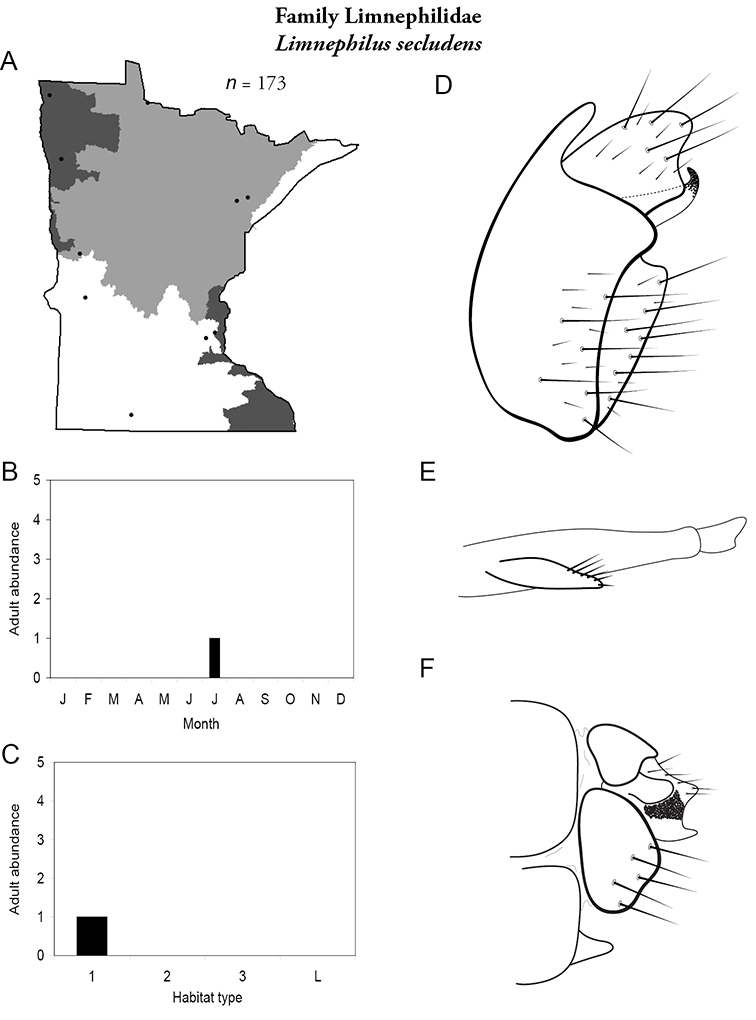
*Limnephilus secludens* **A** total specimens collected and all known collecting localities (Figure 4) **B** monthly adult abundance (1980s to present) **C** habitat preference (1980s to present) (Table 1) **D** male genital capsule **E** phallus **F** female genital capsule.

***Limnephilus secludens*** (Figure 210) is known historically from the Northern, Northwestern, and Southern Regions. It was particularly abundant in the Northwestern Region and represented in that region by >100 specimens collected from 1935 to 1937. It has not been collected in the Northwestern Region since 1941. Since 1968, it is known only from a single specimen collected from an unnamed spring, Martin County, in the Southern Region during July 1999. Such habitat types are now extremely rare in the Northwestern and Southern Regions (Houghton 2007). Due to its precipitous decrease in abundance and distribution, and the lack of undisturbed habitats throughout its range, the Minnesota Department of Natural Resources has proposed “Endangered” status for *Limnephilus secludens* (MNDNR 2012).

**Figure 211.**
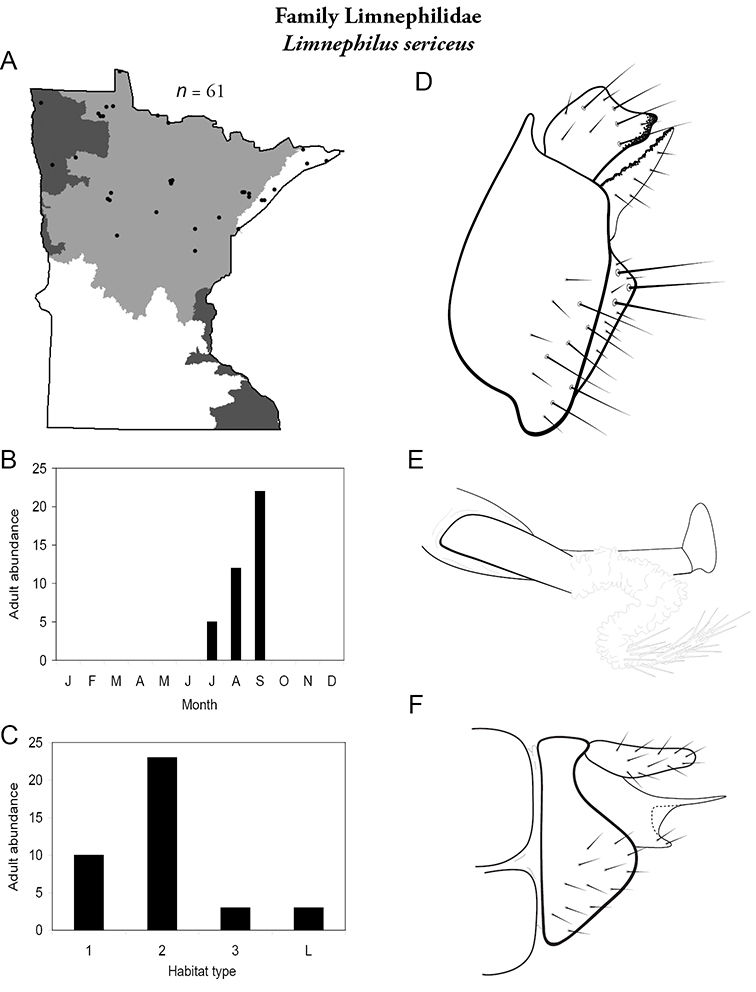
*Limnephilus sericeus* **A** total specimens collected and all known collecting localities (Figure 4) **B** monthly adult abundance (1980s to present) **C** habitat preference (1980s to present) (Table 1) **D** male genital capsule **E** phallus **F** female genital capsule.

***Limnephilus sericeus*** (Figure 211) has been collected from the Lake Superior and Northern Regions since 1940; it was also collected in the Northwestern Region during the 1930s. It was most abundant in small and, especially, medium streams. Adults were present in July and August, and most abundant in September.

**Figure 212.**
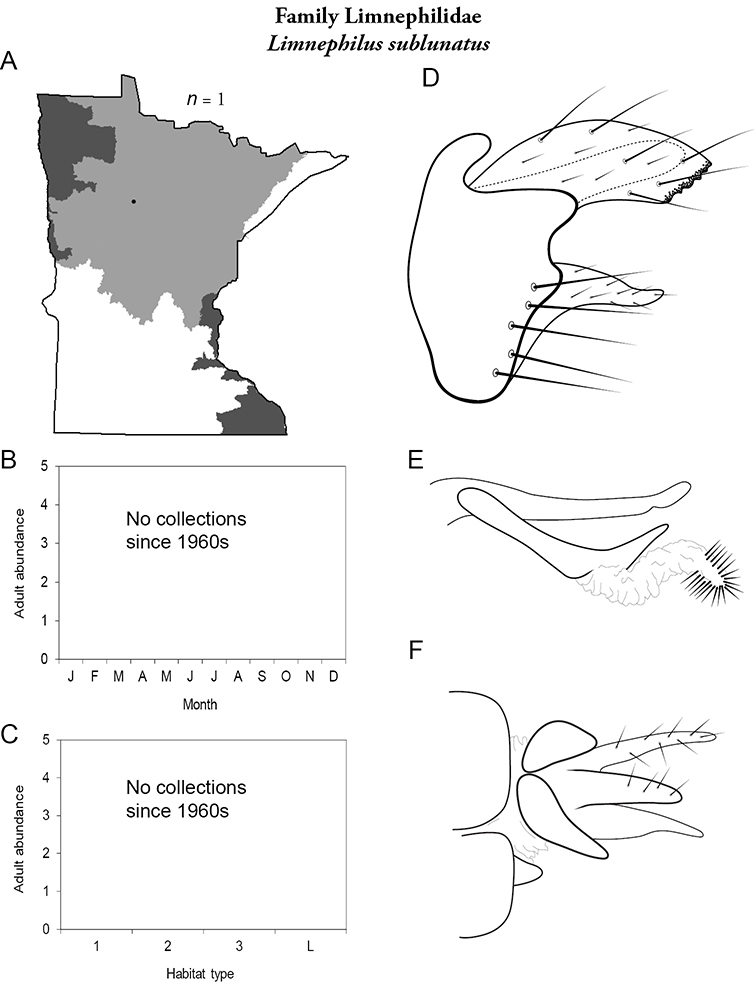
*Limnephilus sublunatus* **A** total specimens collected and all known collecting localities (Figure 4) **B** monthly adult abundance (1980s to present) **C** habitat preference (1980s to present) (Table 1) **D** male genital capsule **E** phallus **F** female genital capsule.

***Limnephilus sublunatus*** (Figure 212) is known only from a single specimen collected from the city of Guthrie, Hubbard County, in the Northern Region during July 1965.

**Figure 213.**
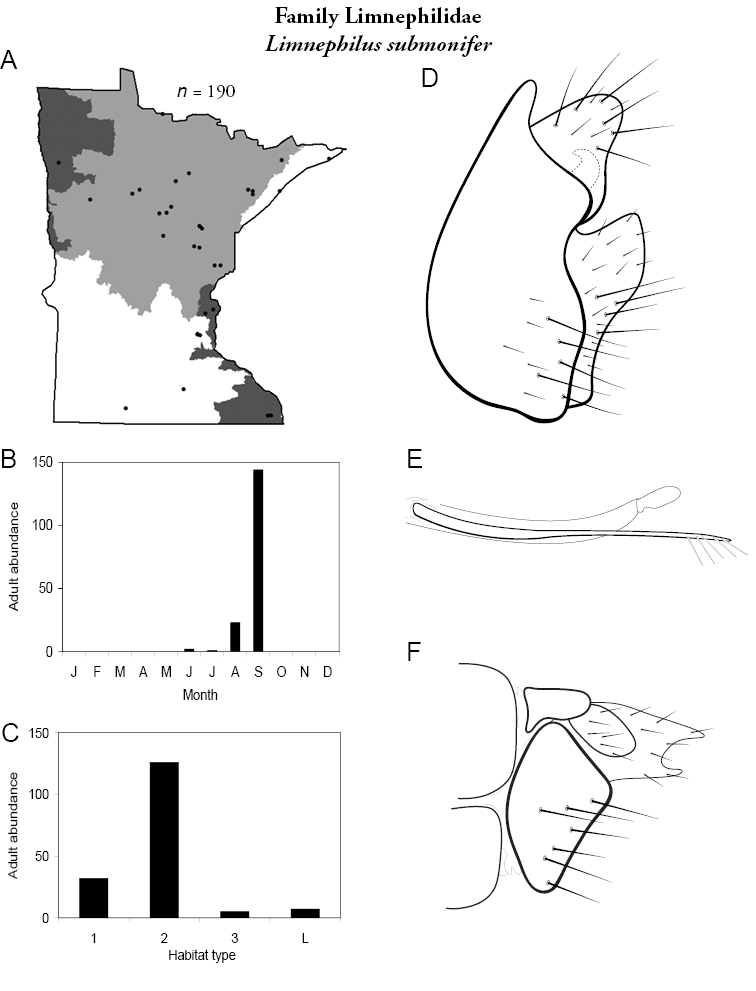
*Limnephilus submonifer* **A** total specimens collected and all known collecting localities (Figure 4) **B** monthly adult abundance (1980s to present) **C** habitat preference (1980s to present) (Table 1) **D** male genital capsule **E** phallus **F** female genital capsule.

***Limnephilus submonifer*** (Figure 213) has historically been found throughout the state. Since the 1960s, however, it has been collected only in the Lake Superior and Northern Regions. It was most abundant in small and, especially, medium stream. Adults were abundant in September and present June through August.

**Figure 214.**
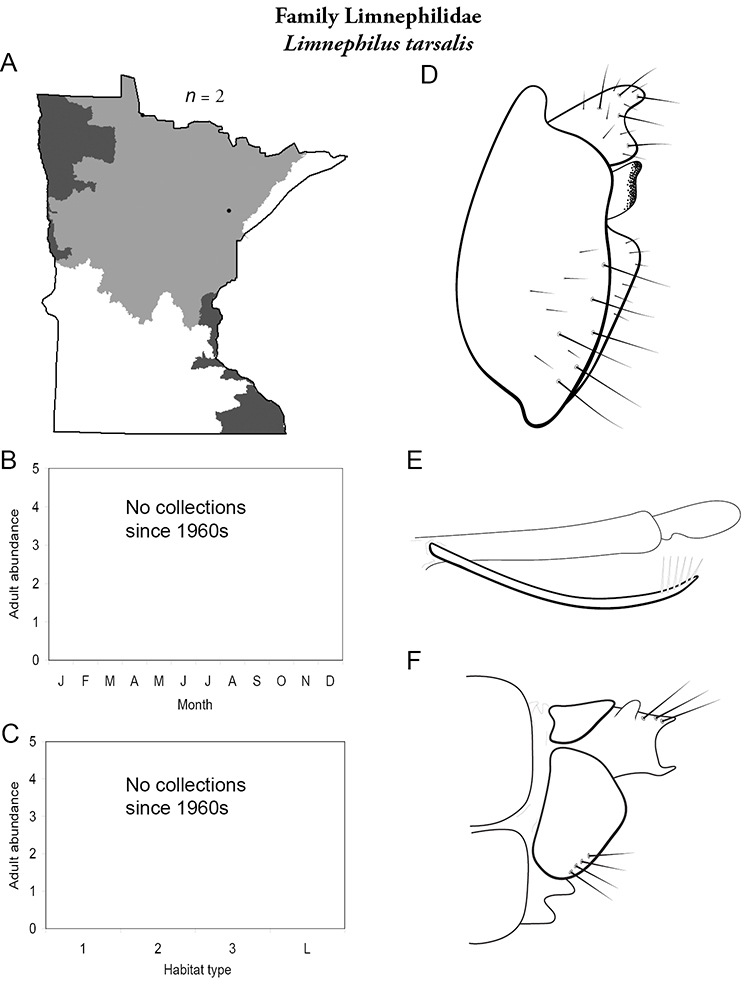
*Limnephilus tarsalis* **A** total specimens collected and all known collecting localities (Figure 4) **B** monthly adult abundance (1980s to present) **C** habitat preference (1980s to present) (Table 1) **D** male genital capsule **E** phallus **F** female genital capsule.

***Limnephilus tarsalis*** (Figure 214) is known only from the cities of Baudette in Lake of the Woods County, and Cotton in Saint Louis County. Both sites are in the Northern Region. It has not been collected since the 1960s, however, and it is difficult to know if it is extirpated or just difficult to collect.

**Figure 215.**
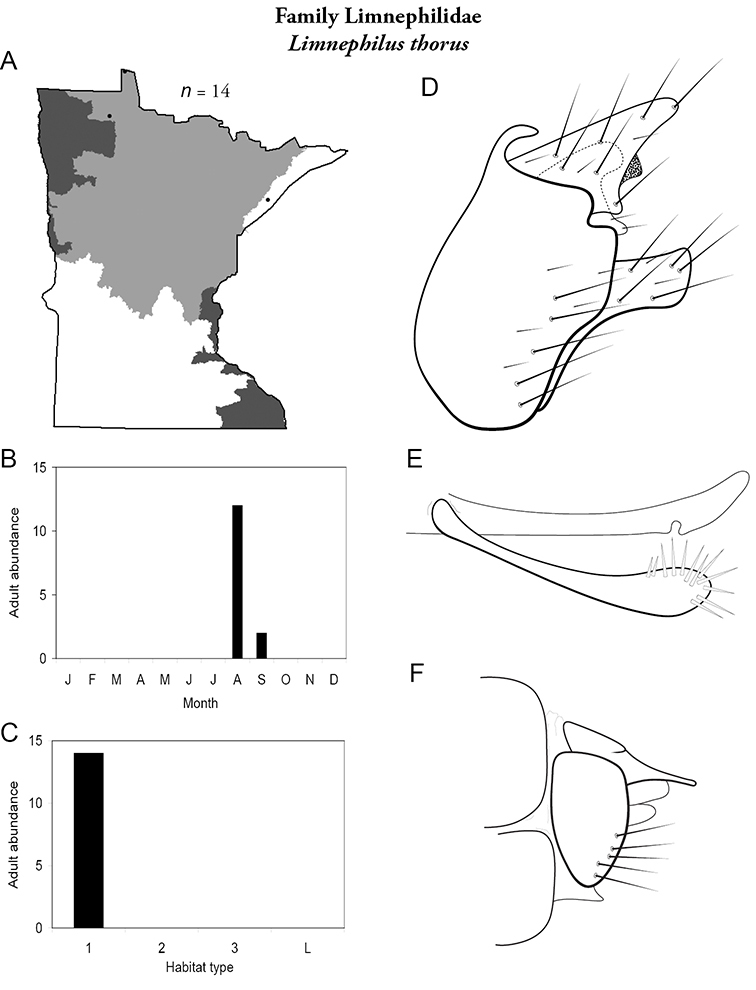
*Limnephilus thorus* **A** total specimens collected and all known collecting localities (Figure 4) **B** monthly adult abundance (1980s to present) **C** habitat preference (1980s to present) (Table 1) **D** male genital capsule **E** phallus **F** female genital capsule.

***Limnephilus thorus*** (Figure 215) is known from a few sites in the Lake Superior and Northern Regions. Adults were present mainly in August and found exclusively in small streams.

Another *Limnephilus* species, *Limnephilus externus*, was reported from Minnesota based on a single pupa (Etnier 1965). No adults have been collected from the state. The species is restricted to the western U.S. (Ruiter 1995) and is unlikely to occur in Minnesota. Thus, *Limnephilus externus* is not included in this manual. Another *Limnephilus* species, *Limnephilus acrocurvus*, now designated as junior synonym of *Limnephilus dispar* (Ruiter 1995), is also excluded from this manual due to a lack of adult specimens collected from the state.

### Genus *Nemotaulius*

The genus *Nemotaulius* contains a single species in Minnesota. For additional species, see Schmid (1952a). Larvae are typically found in beds of aquatic macrophytes in lakes, marshes, and slow-moving areas of streams (Wiggins 1996). They feed primarily on plant debris. Larval cases are usually composed of large leaf pieces arranged as dorsal and ventral layers with the larva “sandwiched” between them. Adults are large and distinctive; ranging 25–30 mm in length and with the apical margin of the forewings notably scalloped.

**Figure 216.**
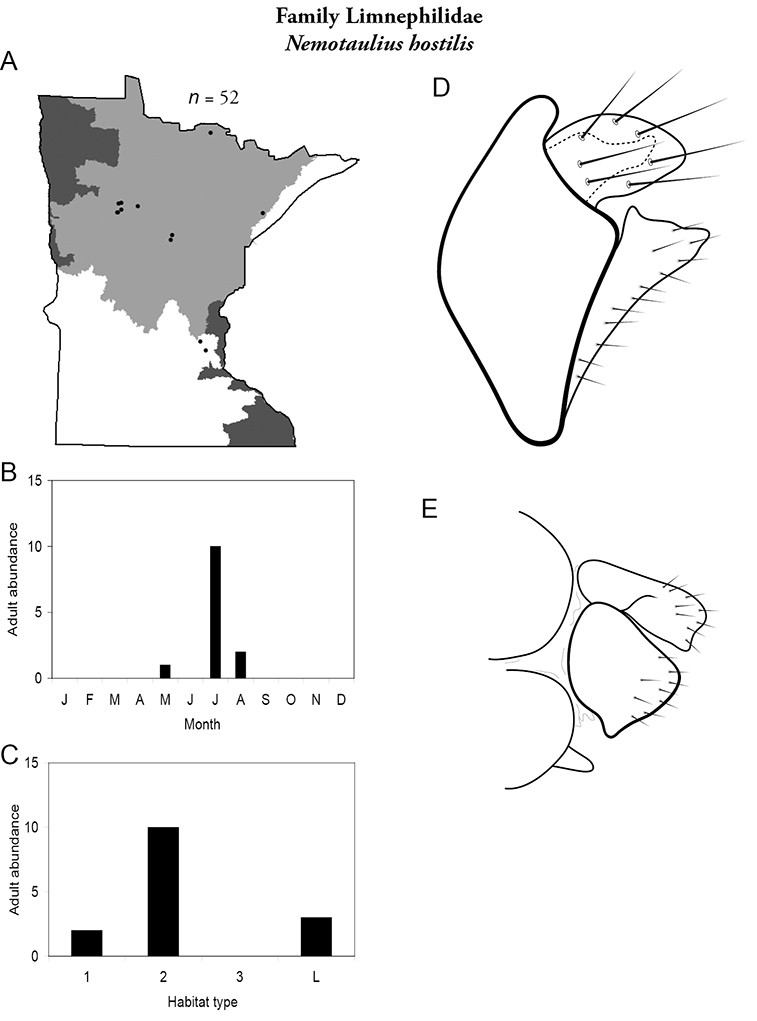
*Nemotaulius hostilis* **A** total specimens collected and all known collecting localities (Figure 4) **B** monthly adult abundance (1980s to present) **C** habitat preference (1980s to present) (Table 1) **D** male genital capsule **E** female genital capsule.

***Nemotaulius hostilis*** (Figure 216) is known mainly from the Northern Region, with some scattered records from the Southern Region. It was found predominantly in medium rivers, but also in lakes and small streams. Most adults were caught in July, with a few in May and August.

### Genus *Onocosmoecus*

The genus *Onocosmoecus* contains a single species in Minnesota. For additional species, see Wiggins and Richardson (1986). Larvae are usually found in slow-moving areas of cold streams. They consume mainly plant debris and decaying organic matter. Larval cases are composed of thin pieces of wood, bark, and leaves (Wiggins 1996). Adults range 18–22 mm in length and are pale orange in color. Superficially, they resemble adults of *Pycnopsyche*, with which they are often collected.

**Figure 217.**
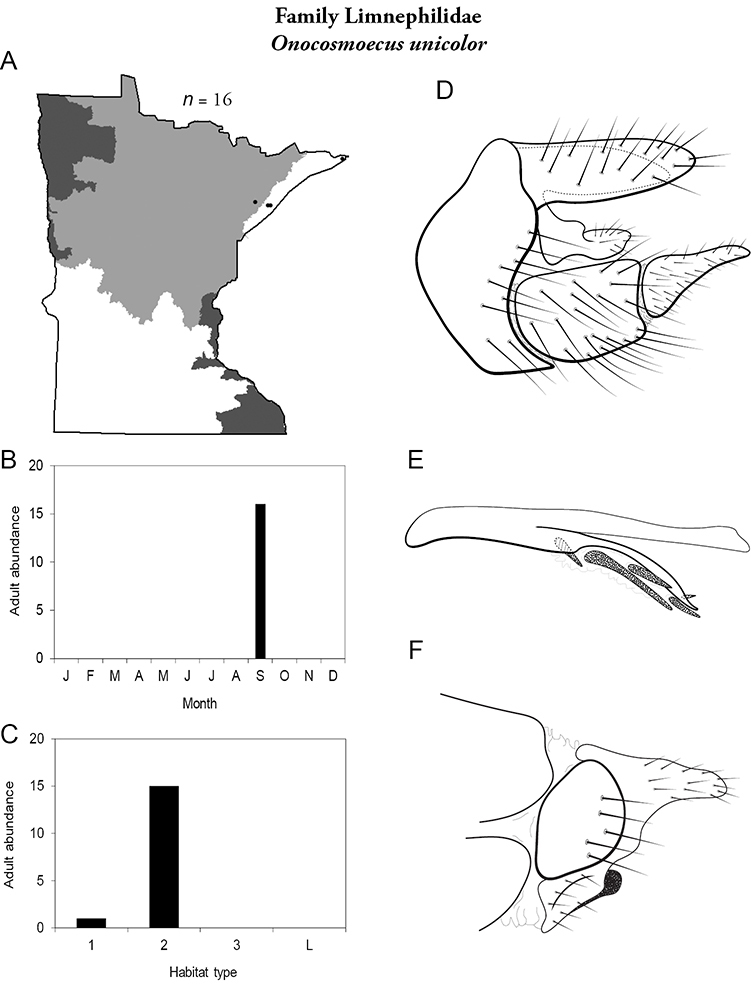
*Onocosmoecus unicolor* **A** total specimens collected and all known collecting localities (Figure 4) **B** monthly adult abundance (1980s to present) **C** habitat preference (1980s to present) (Table 1) **D** male genital capsule **E** phallus **F** female genital capsule.

***Onocosmoecus unicolor*** (Figure 217) has been collected only from or near the Lake Superior Region. It was found in small and medium rivers, exclusively during September.

### Genus *Philarctus*

The genus *Philarctus* contains a single species in Minnesota. Larvae typically inhabit lakes and slow-moving areas of streams where they feed mainly on detritus (Wiggins 1996). Larval cases can be constructed of small mineral particles, sedge seeds, or even clam or snail shells.

**Figure 218.**
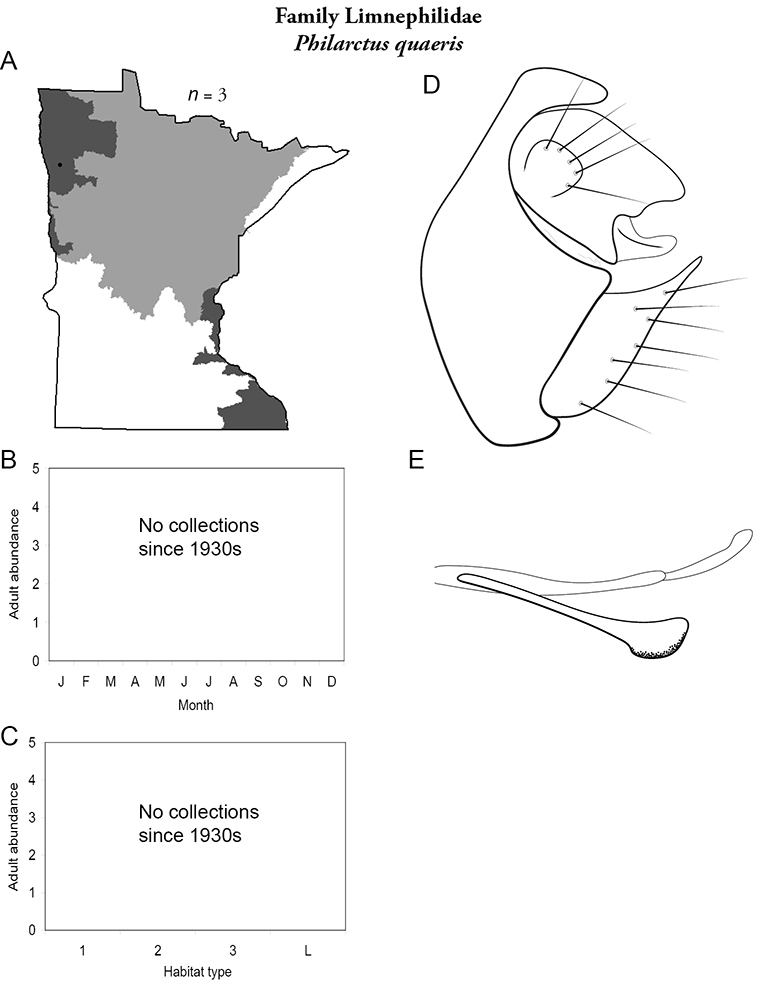
*Philarctus quaeris* **A** total specimens collected and all known collecting localities (Figure 4) **B** monthly adult abundance (1980s to present) **C** habitat preference (1980s to present) (Table 1) **D** male genital capsule **E** phallus.

***Philarctus quaeris*** (Figure 218) is known in Minnesota only from 3 collections in the 1930s from the City of Crookston, Polk County, in the Northwestern Region. These collections pre-date the majority of habitat destruction in this region (Houghton 2007). The species has not been collected since and is presumed extirpated from the state.

### Genus *Platycentropus*

The genus *Platycentropus* contains 2 fairly common species in Minnesota that differ mainly in their adult flight period. Larvae live in a wide variety of habitats, from cool streams to warm ponds (Wiggins 1996). They feed mostly on plant debris. Larval cases are composed of long pieces of grasses or sedges arranged transversely. Adults range 20–25 mm in length. They have striking forewings of gold and orange patterning (Figure 294).

**Figure 219.**
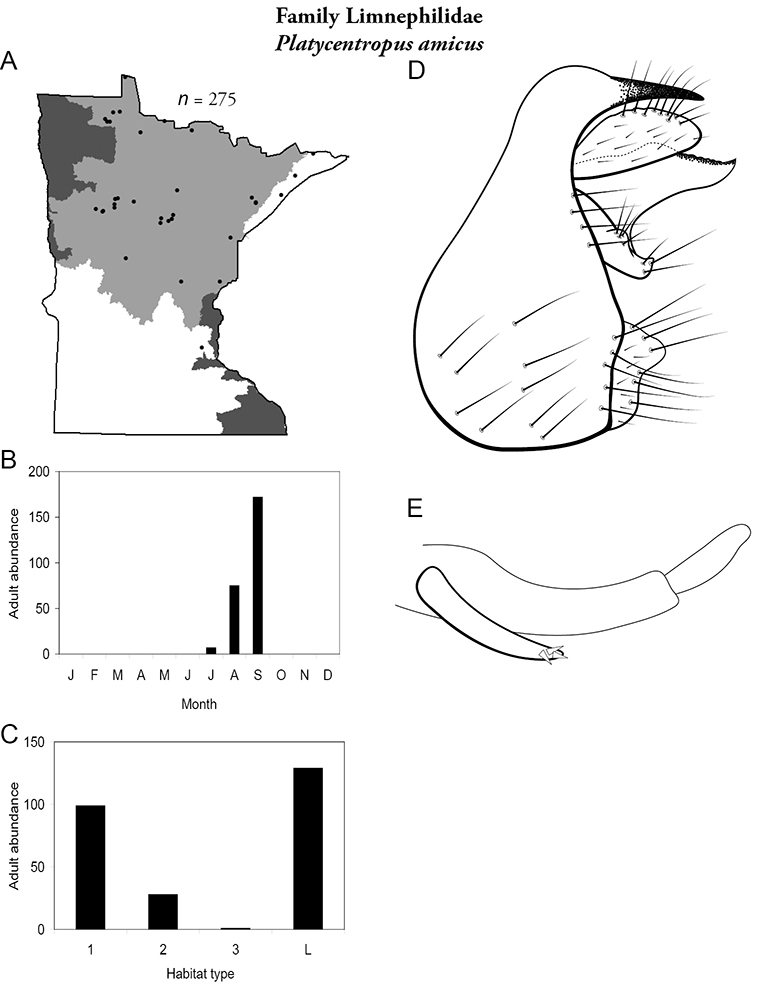
*Platycentropus amicus* **A** total specimens collected and all known collecting localities (Figure 4) **B** monthly adult abundance (1980s to present) **C** habitat preference (1980s to present) (Table 1) **D** male genital capsule **E** phallus.

***Platycentropus amicus*** (Figure 219) has been found throughout the Lake Superior and Northern Regions. It was most abundant in lakes and small streams and the interface between them. Adults were most abundant in August and, especially, September.

**Figure 220.**
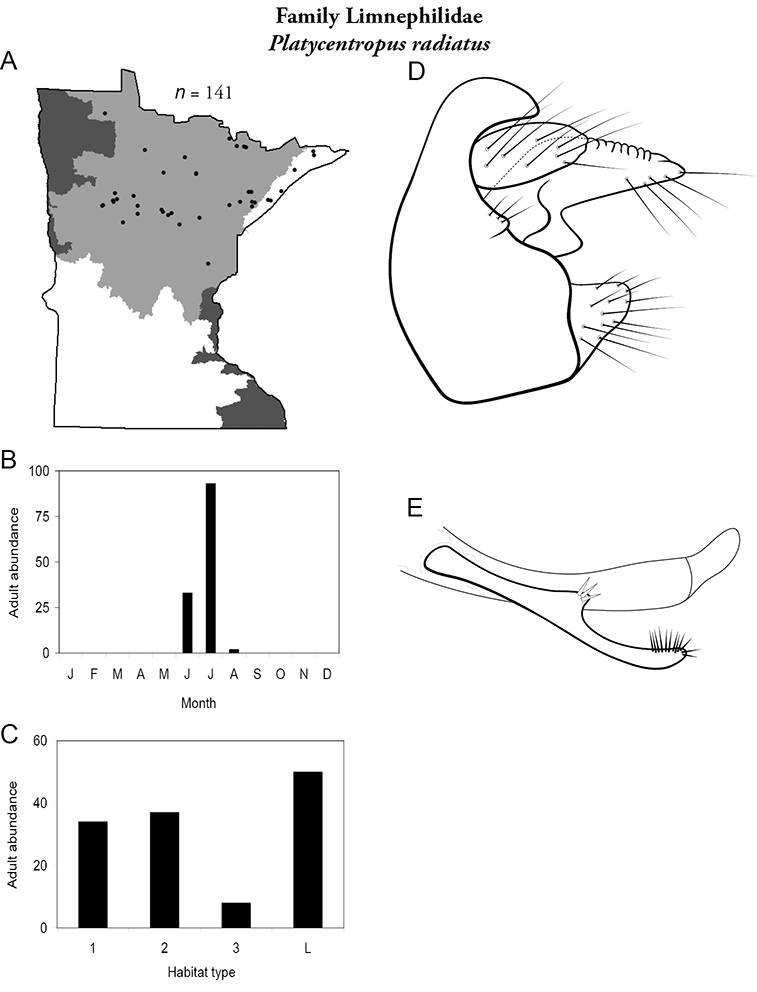
*Platycentropus radiatus* **A** total specimens collected and all known collecting localities (Figure 4) **B** monthly adult abundance (1980s to present) **C** habitat preference (1980s to present) (Table 1) **D** male genital capsule **E** phallus.

***Platycentropus radiatus*** (Figure 220) had a similar distribution and habitat preference as *Platycentropus amicus*. It differs in its greater prevalence in medium rivers and its greater abundance in June and July.

### Genus *Pseudostenophylax*

The genus *Pseudostenophylax* contains a single species in Minnesota. Larvae are usually found in cold springs or streams. Larval cases are composed of small uniform mineral particles (Wiggins 1996). Adults are light brown in color and range 15–18 mm in length.

**Figure 221.**
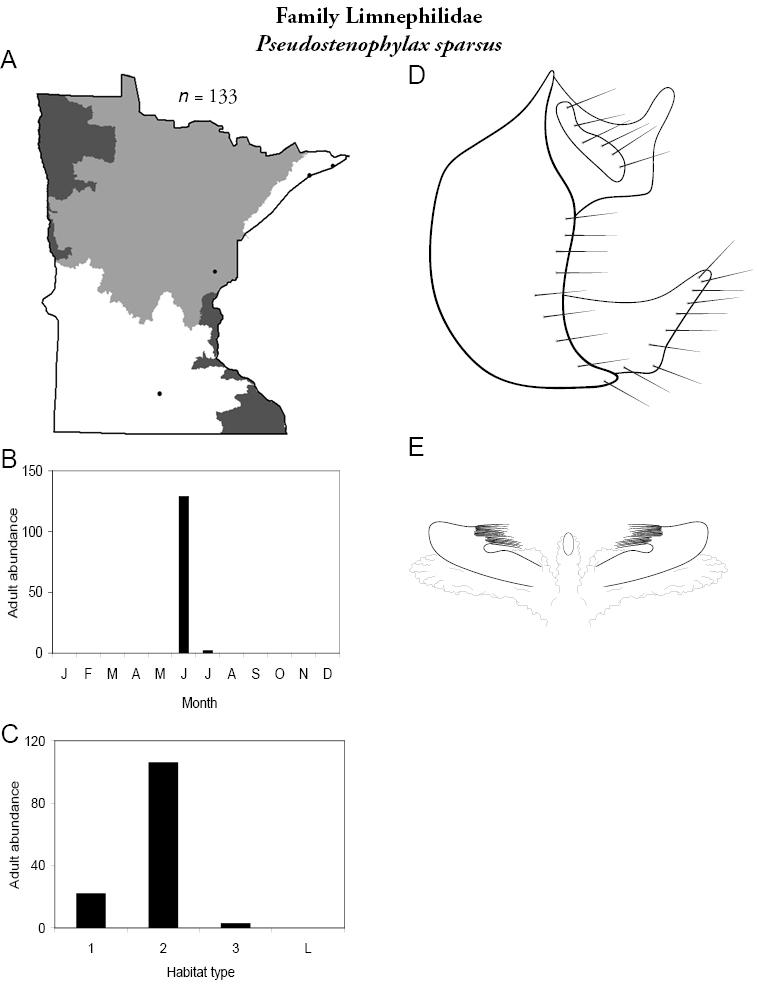
*Pseudostenophylax sparsus* **A** total specimens collected and all known collecting localities (Figure 4) **B** monthly adult abundance (1980s to present) **C** habitat preference (1980s to present) (Table 1) **D** male genital capsule **E** phallus (dorsal view, reduced 50%).

***Pseudostenophylax sparsus*** (Figure 221). has been found sporadically from the Lake Superior, Northern, and Southern Regions. It was locally abundant throughout Minneopa State Park in the Southern Region. It was collected mainly from small and, especially, medium streams and present mostly in June.

Another species of *Pseudostenophylax*, *Pseudostenophylax uniformis*, was reported from Minnesota by Houghton et al. (2001). The species is now considered a subspecies of *Pseudostenophylax sparsus* (Schmid 1991, Morse 2011). Thus, it is not included in this manual.

### Genus *Pycnopsyche*

The genus *Pycnopsyche* contains 5 species in Minnesota. For additional species, see Betten (1950). Larvae are usually the most common and conspicuous limnephilid in slow-moving areas of woodland streams. They can be found clinging to vegetation or large rocks, or simply walking along the stream bottom. Larval cases are typically constructed of organic material, although some species can utilize mineral fragments if preferred material is not available (Houghton et al. 2011c). Larvae typically finish maturing in late spring or early summer, and then undergo diapause until pupation. Adults emerge in the fall and are frequently the most abundant and conspicuous caddisflies in August and September light traps in Minnesota. Adults range 18–22 mm in length and are pale orange in color (Figure 294).

**Figure 222.**
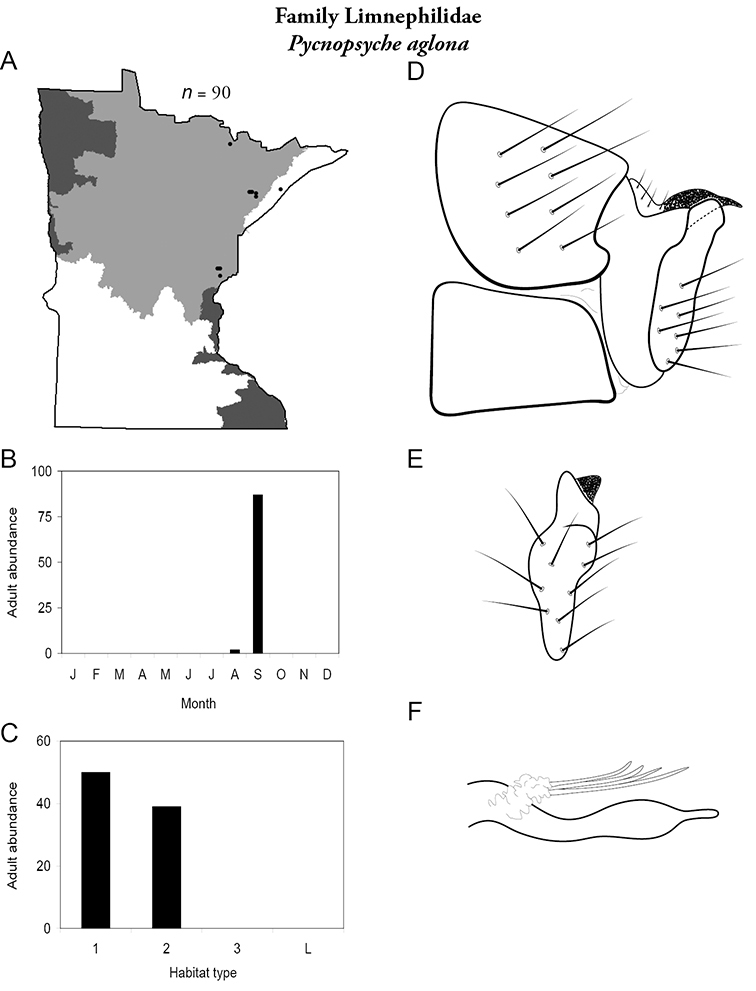
*Pycnopsyche aglona* **A** total specimens collected and all known collecting localities (Figure 4) **B** monthly adult abundance (1980s to present) **C** habitat preference (1980s to present) (Table 1) **D** male genital capsule **E** male inferior appendage (caudal view) **F** phallus.

***Pycnopsyche aglona*** (Figure 222) is known from the Lake Superior and Northern Regions. It has been collected exclusively from small and medium streams, and is most abundant during September.

**Figure 223.**
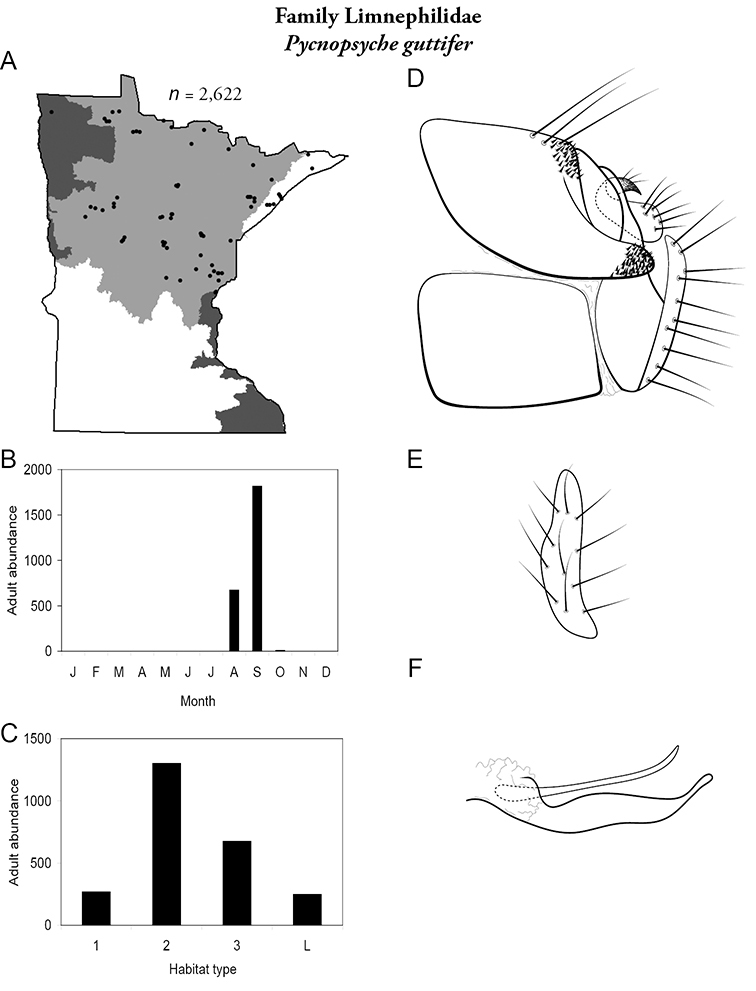
*Pycnopsyche guttifer* **A** total specimens collected and all known collecting localities (Figure 4) **B** monthly adult abundance (1980s to present) **C** habitat preference (1980s to present) (Table 1) **D** male genital capsule **E** male inferior appendage (caudal view) **F** phallus.

***Pycnopsyche guttifer*** (Figure 223) is known mostly from the Lake Superior and Northern Regions where it was, by far, the most abundant of the *Pycnopsyche* species. It was found in all habitat types, but was most abundant in medium and large rivers. Adults were collected in August and, especially, September.

**Figure 224.**
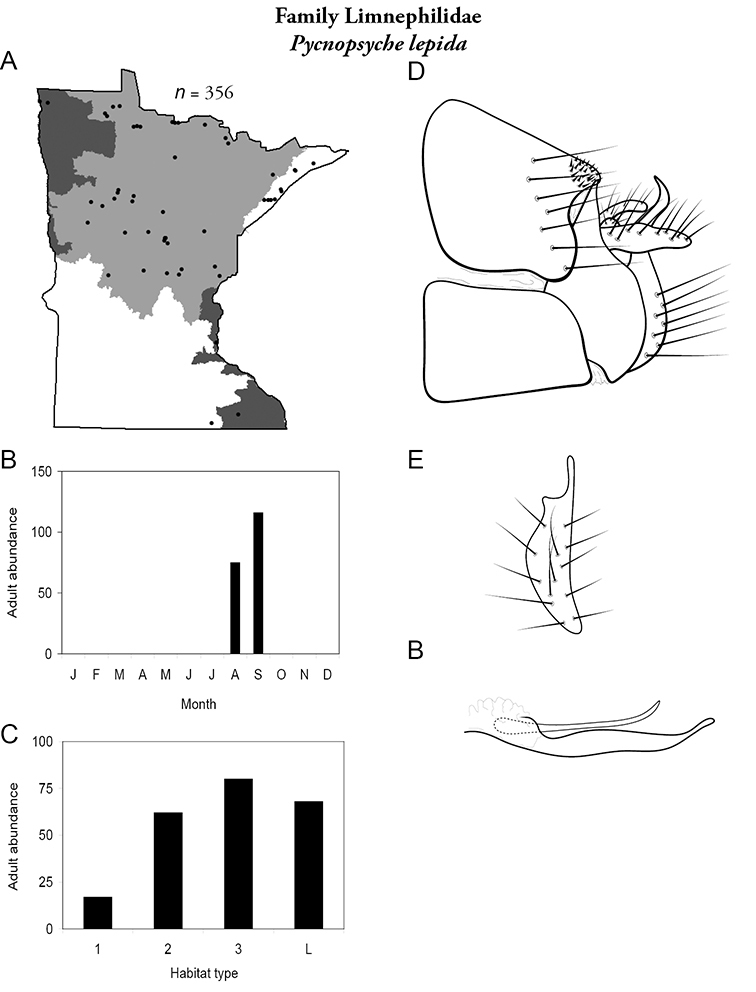
*Pycnopsyche lepida* **A** total specimens collected and all known collecting localities (Figure 4) **B** monthly adult abundance (1980s to present) **C** habitat preference (1980s to present) (Table 1) **D** male genital capsule **E** male inferior appendage (caudal view) **F** phallus.

***Pycnopsyche lepida*** (Figure 224) is also known mostly from the Lake Superior and Northern region, with scattered records from the other regions. Adults were collected in August and September from all habitat types.

**Figure 225.**
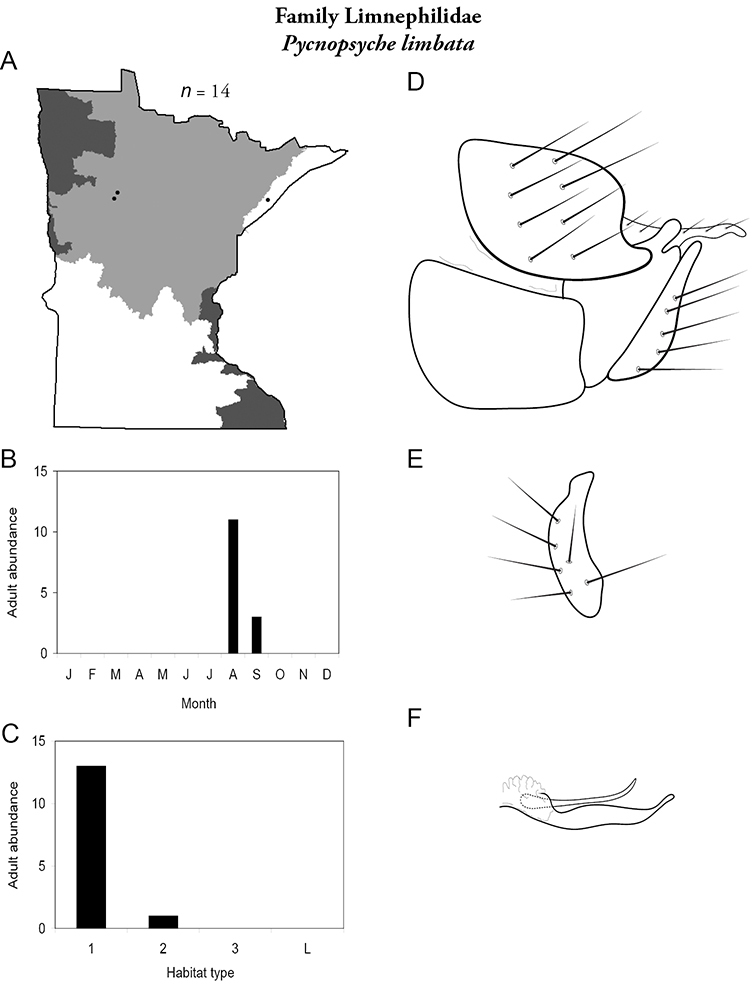
*Pycnopsyche limbata* **A** total specimens collected and all known collecting localities (Figure 4) **B** monthly adult abundance (1980s to present) **C** habitat preference (1980s to present) (Table 1) **D** male genital capsule **E** male inferior appendage (caudal view) **F** phallus.

***Pycnopsyche limbata*** (Figure 225) is known from scattered localities in the Lake Superior and Northern Regions. Adults were collected in August and September, almost exclusively from small streams.

**Figure 226.**
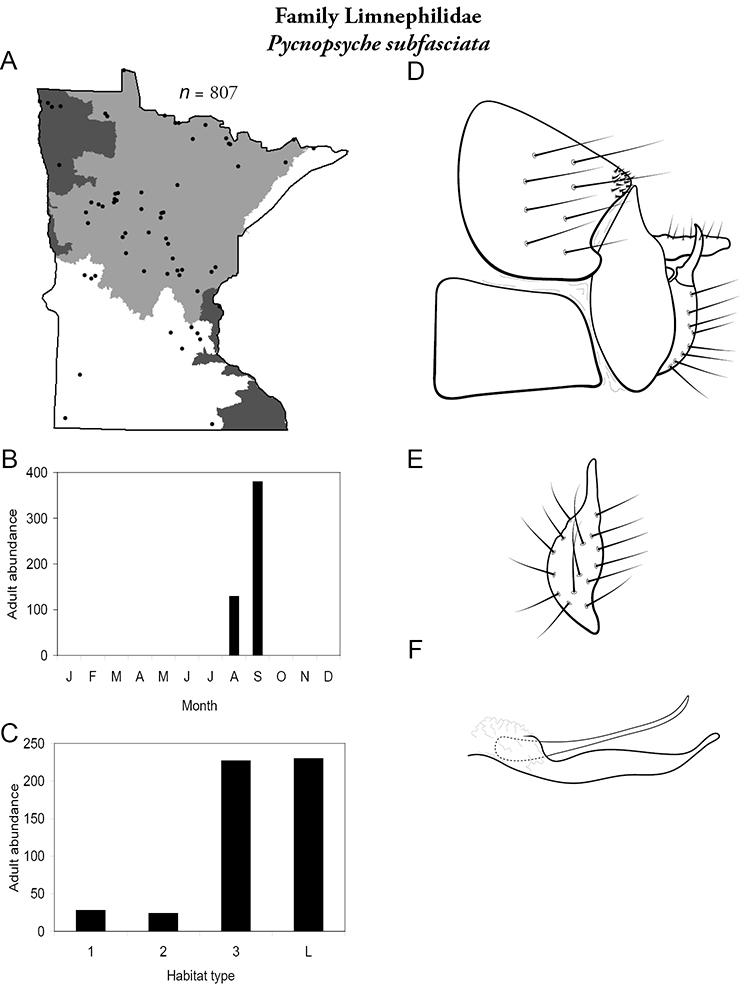
*Pycnopsyche subfasciata* **A** total specimens collected and all known collecting localities (Figure 4) **B** monthly adult abundance (1980s to present) **C** habitat preference (1980s to present) (Table 1) **D** male genital capsule **E** male inferior appendage (caudal view) **F** phallus.

***Pycnopsyche subfasciata*** (Figure 226) was the most widespread of the *Pycnopsyche* species, found in all regions. It was most abundant in lakes and large rivers, and collected during August and September.

Another *Pycnopsyche* species, *Pycnopsyche scabripennis*, was reported from Minnesota from a single larva (Lager et al. 1979). No adults of this species have been collected from the state. Thus, without confirmation, *Pycnopsyche scabripennis* is not included in this manual.

### Family Molannidae

This family contains one genus in Minnesota, *Molanna*, and a total of 4 species. Larvae are typically found on rocky substrates where they graze on periphyton (Wiggins 1996). They construct portable cases that superficially resemble “mummy-style” sleeping bags, with a central tube and lateral flanges. Adults are black or dark brown in color and 8–12 mm in length (Figure 294).

### Genus *Molanna*

The genus*Molanna* contains 4 species in Minnesota. Larvae can be found in both lakes and streams. Individual species often have a strong preference for a particular type of habitat.

**Figure 227.**
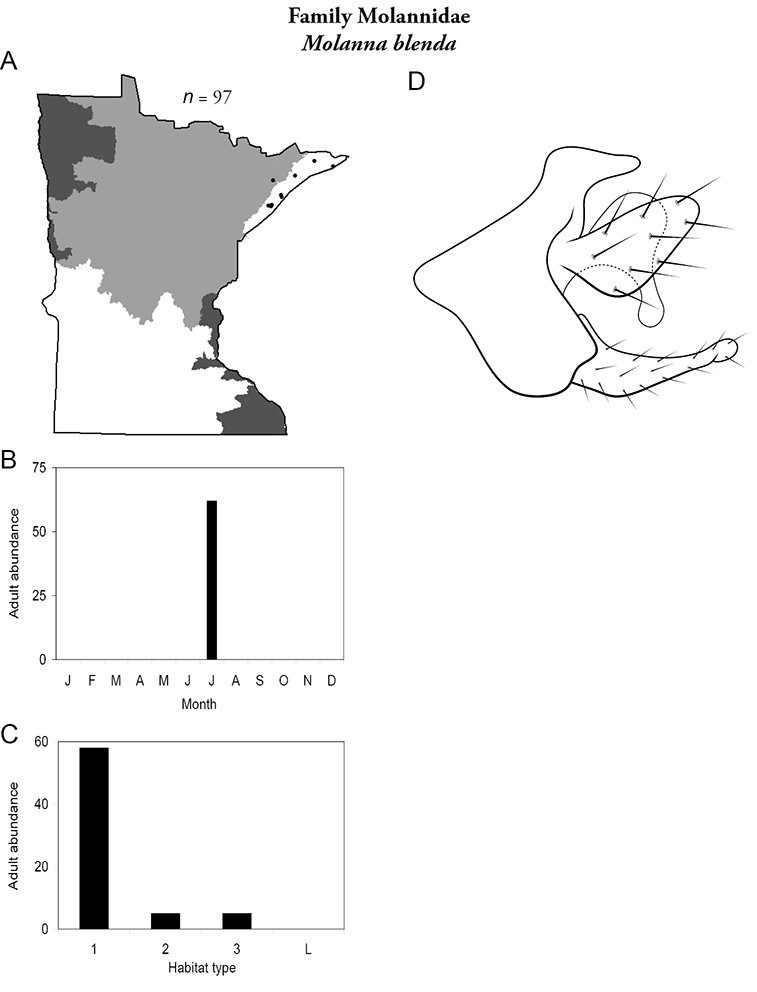
*Molanna blenda* **A** total specimens collected and all known collecting localities (Figure 4) **B** monthly adult abundance (1980s to present) **C** habitat preference (1980s to present) (Table 1) **D** male genital capsule.

***Molanna blenda***(Figure 227) has only been found in or near the Lake Superior Region during July. It is known only from streams, typically small streams.

**Figure 228.**
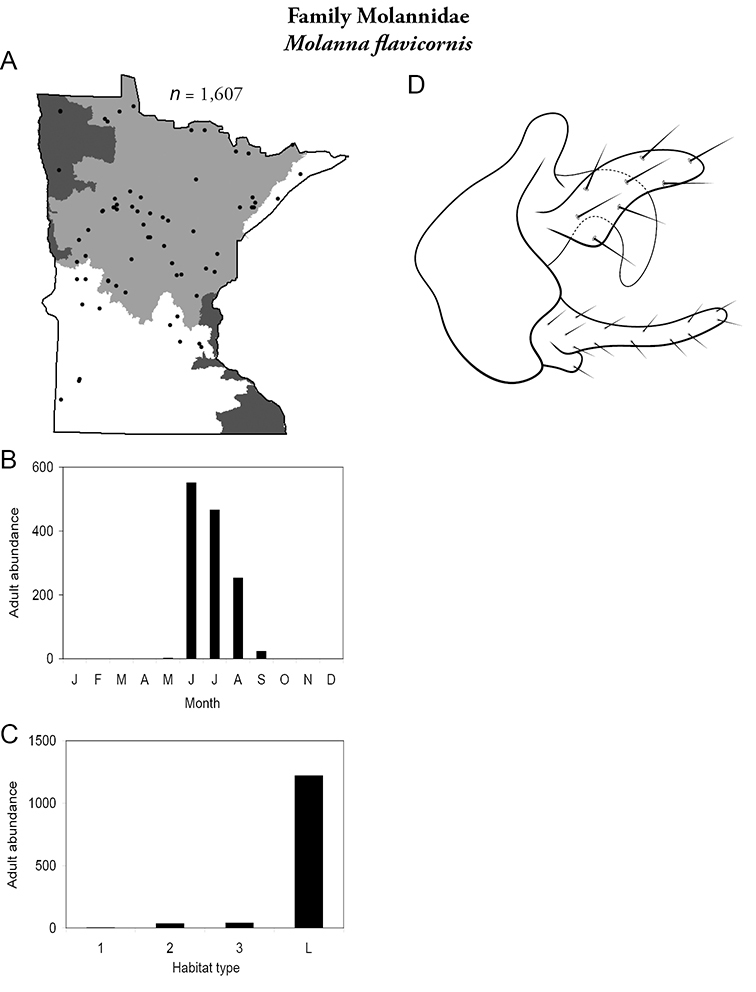
*Molanna flavicornis* **A** total specimens collected and all known collecting localities (Figure 4) **B** monthly adult abundance (1980s to present) **C** habitat preference (1980s to present) (Table 1) **D** male genital capsule.

***Molanna flavicornis*** (Figure 228) has been collected throughout all regions except the Southeastern. It was found almost exclusively in lakes, and abundant from June through August, with a few specimens collected in September.

**Figure 229.**
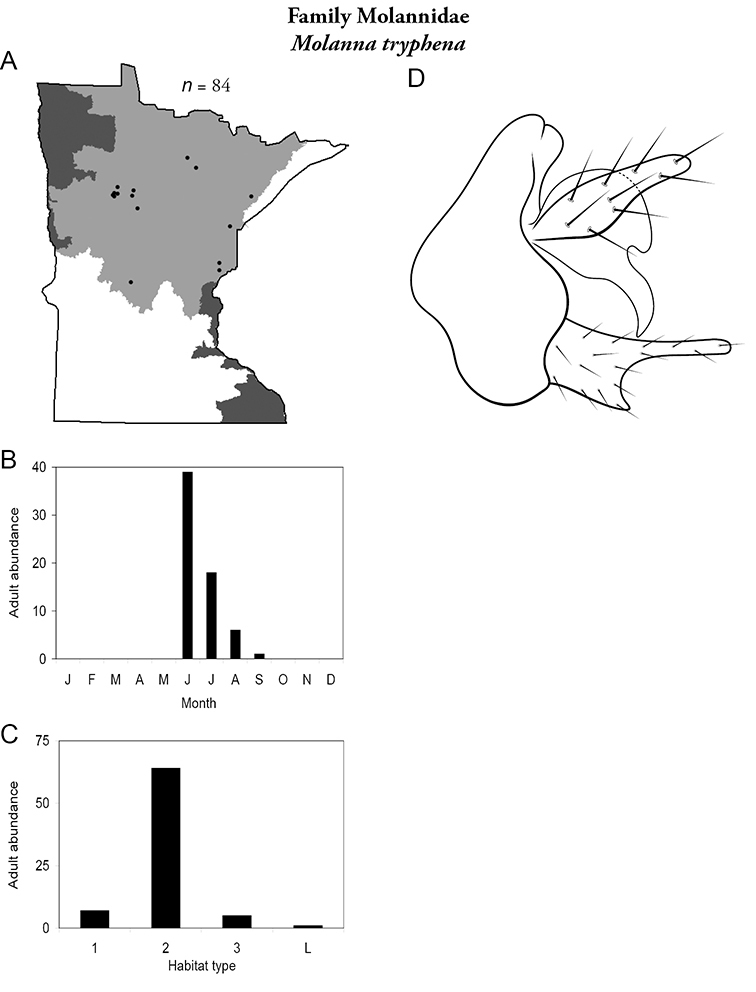
*Molanna tryphena* **A** total specimens collected and all known collecting localities (Figure 4) **B** monthly adult abundance (1980s to present) **C** habitat preference (1980s to present) (Table 1) **D** male genital capsule.

***Molanna tryphena*** (Figure 229) was found predominately in medium streams and is only known from the Northern Region. Adults have been collected mostly in June and July, with some specimens found in August and September.

**Figure 230.**
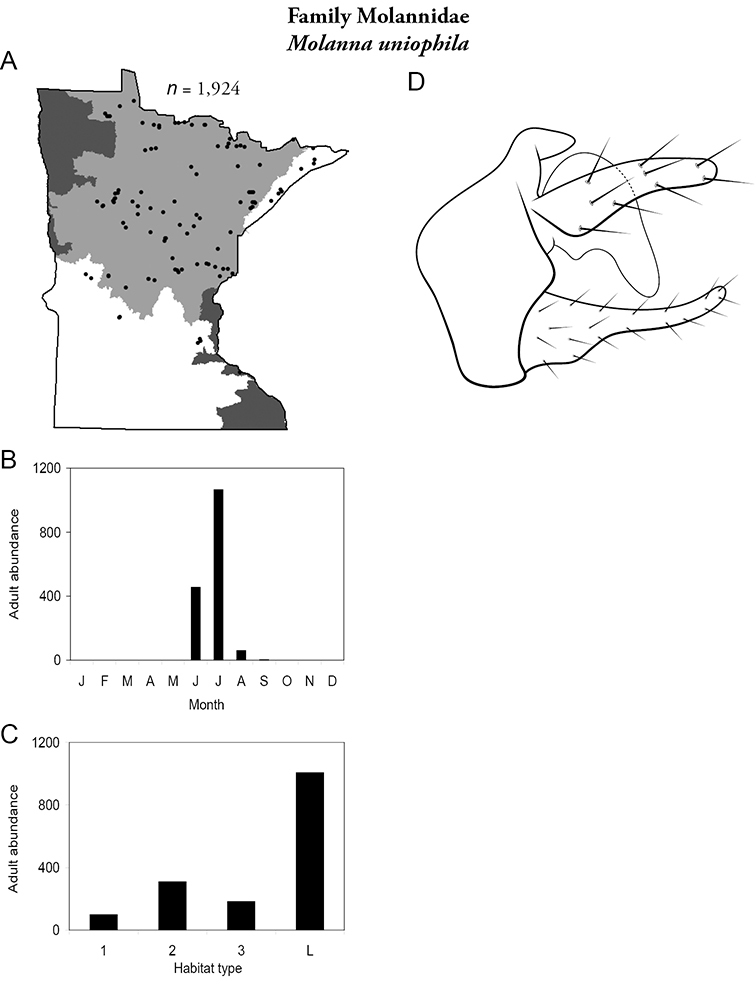
*Molanna uniophila* **A** total specimens collected and all known collecting localities (Figure 4) **B** monthly adult abundance (1980s to present) **C** habitat preference (1980s to present) (Table 1) **D** male genital capsule.

***Molanna uniophila*** (Figure 230) was the most abundant of the *Molanna* species, found in both lakes and streams, but more commonly in lakes. It was common throughout the Lake Superior and Northern Regions and found occasionally in the Southern Region.

Another *Molanna* species, *Molanna ulmerina*, was reported from a specimen of unknown sex (Harris et al. 1991). The whereabouts of this specimen is unknown. In the absence of specimens to confirm the species’ presence in Minnesota, *Molanna ulmerina* is not included in this manual.

### Family Odontoceridae

This family contains a single genus in Minnesota, *Psilotreta*, and a single species. For additional species, see Parker and Wiggins (1987). Larvae of *Psilotreta* live in fast-moving areas of streams where they are found on medium and large rocks or occasionally buried into sandy substrates (Wiggins 1996). Larvae consume algae, vascular plants, and other organic particles. They are unique in forming large aggregations prior to pupation. Adults are dark brown in color, and 6–8 mm in length.

### Genus *Psilotreta*

**Figure 231.**
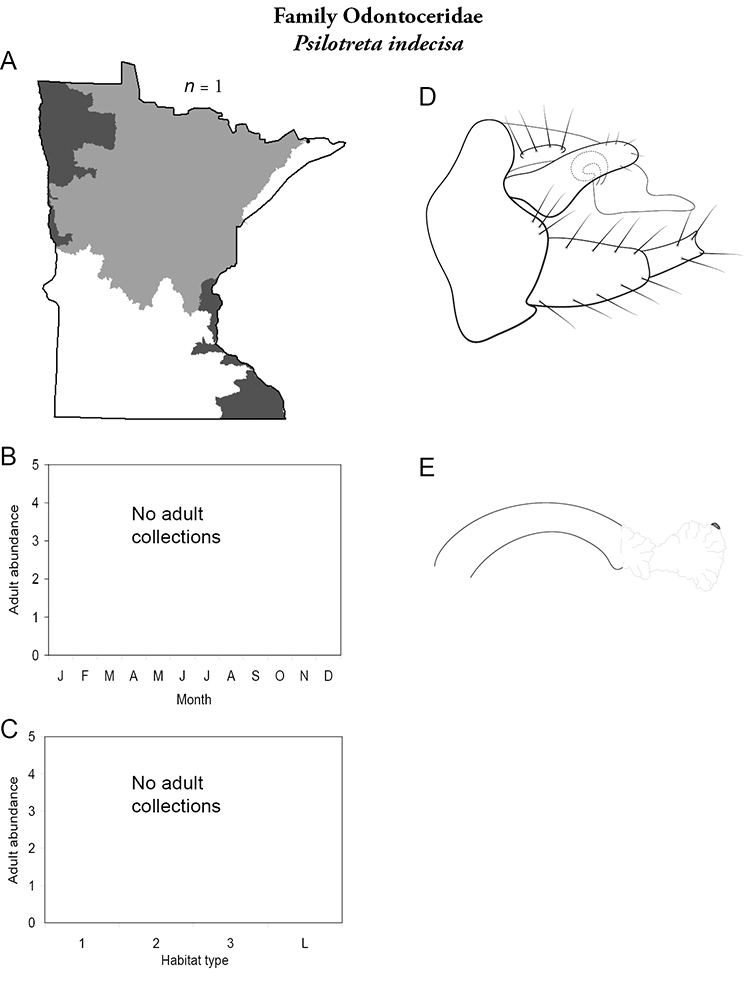
*Psilotreta indecisa* **A** total specimens collected and all known collecting localities (Figure 4) **B** monthly adult abundance (1980s to present) **C** habitat preference (1980s to present) (Table 1) **D** male genital capsule **E** phallus.

***Psilotreta indecisa*** (Figure 231) is known in Minnesota only from a single set of larval sclerites collected from the Cross River, Cook County, at the eastern edge of the Northern Region during August 2007. The Cross River is a medium-sized, fast-flowing woodland stream. The actual organism had already vacated its case and emerged as an adult before the collection. Thus, adults have not been definitely associated with these specimens and it is possible that they may of a different species. The abandoned sclerites, however, do key to *Psilotreta indecisa* using Parker and Wiggins (1987). Further, the other known species of *Psilotreta* are restricted to the far eastern portion of the U.S., whereas *Psilotreta indecisa* has previously been found as far west as Wisconsin (Morse 2011).

### Family Philopotamidae

This family contains 3 genera in Minnesota: *Chimarra*, *Dolophilodes*, and *Wormaldia*, and a total of 6 species. The last genus, however, has not been collected in Minnesota since the 1960s. Larvae, especially those of *Chimarra*, are common, conspicuous, and sometimes very abundant on the undersides of medium to large rocks in most types of streams. Larvae of all genera are filtering collectors. They construct a sac-like silken net that they use to capture small suspended particulate organic matter from the stream current (Wiggins 1996). Adults of all genera range 8–10 mm in length.

### Genus *Chimarra*

The genus *Chimarra* contains 4 species in Minnesota. For additional species, see Armitage (1991) or Lago and Harris (1987). Specimens were frequently very abundant in light traps. Both the body and the wings of adults are jet-black in color.

**Figure 232.**
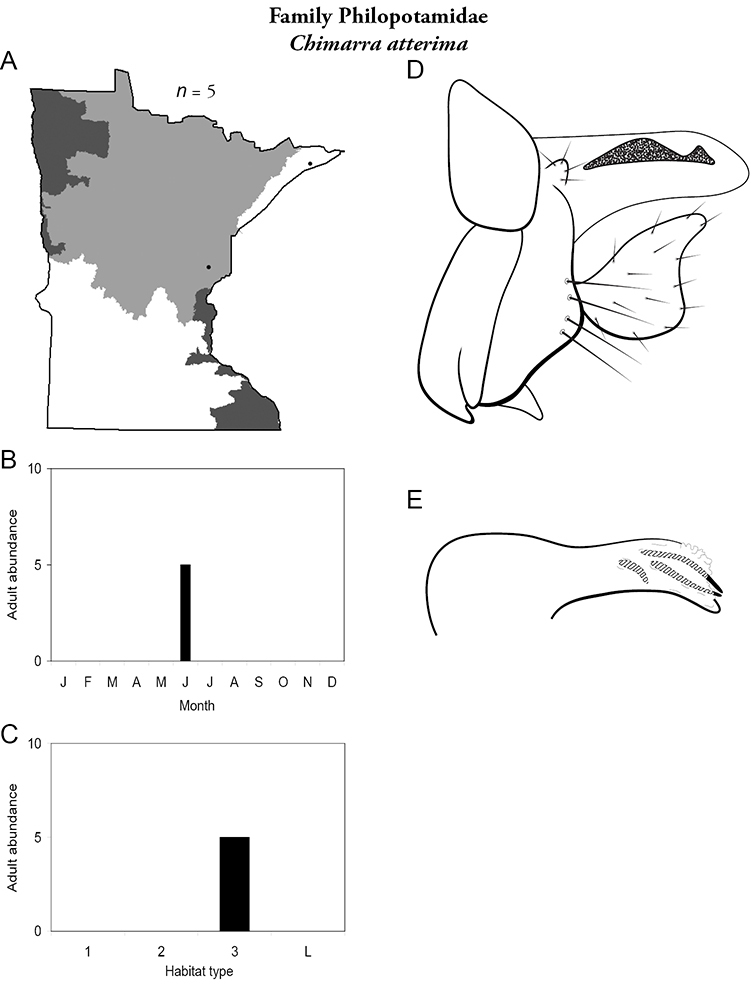
*Chimarra aterrima* **A** total specimens collected and all known collecting localities (Figure 4) **B** monthly adult abundance (1980s to present) **C** habitat preference (1980s to present) (Table 1) **D** male genital capsule **E** phallus.

***Chimarra atterima*** (Figure 232) is known from only 2 locations in the Lake Superior and Northern Regions collected during June. Both localities were large, fast-moving rivers.

**Figure 233.**
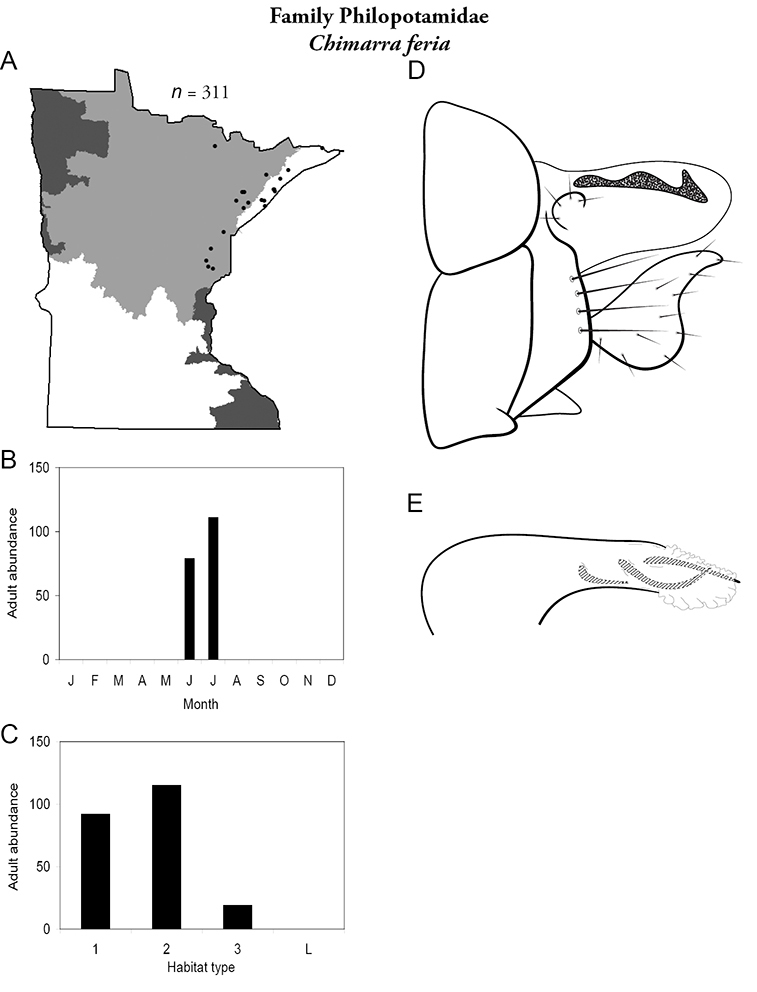
*Chimarra feria* **A** total specimens collected and all known collecting localities (Figure 4) **B** monthly adult abundance (1980s to present) **C** habitat preference (1980s to present) (Table 1) **D** male genital capsule **E** phallus.

***Chimarra feria*** (Figure 233) is known mostly from cold small and medium rivers of the Lake Superior and Northern Regions. Specimens were collected in June and July.

**Figure 234.**
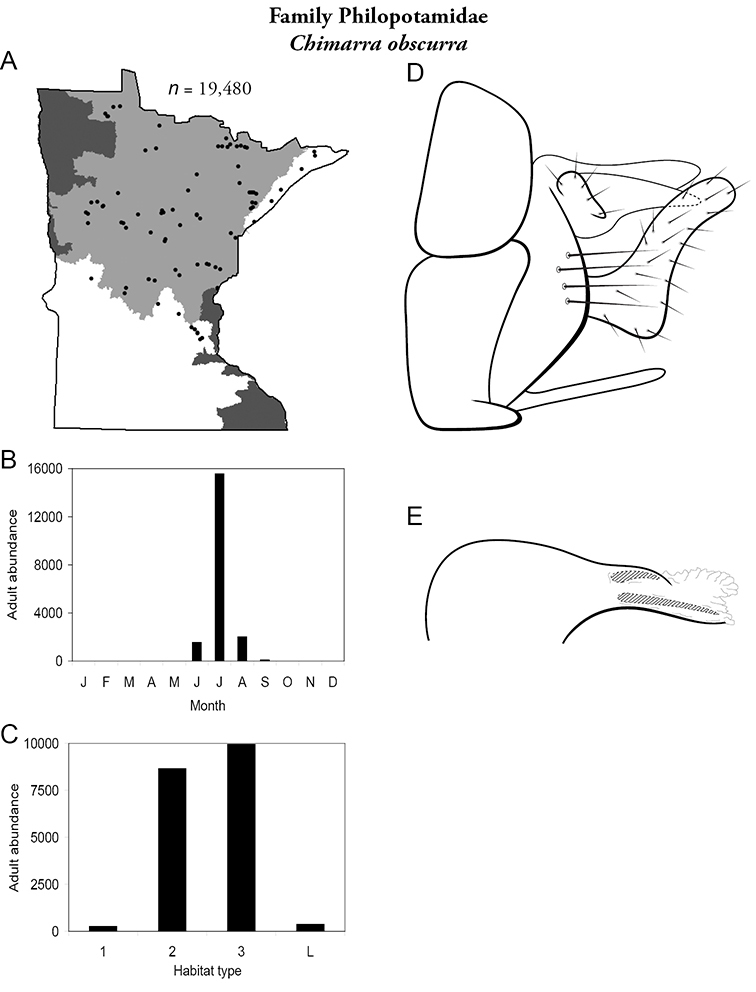
*Chimarra obscurra* **A** total specimens collected and all known collecting localities (Figure 4) **B** monthly adult abundance (1980s to present) **C** habitat preference (1980s to present) (Table 1) **D** male genital capsule **E** phallus.

***Chimarra obscurra*** (Figure 234) was common and abundant in the Lake Superior, Southern and, especially, the Northern Region. It was most abundant in medium and large rivers. It was the single most abundant species in medium rivers of the Northern Region, and the 3rd most abundant species in large rivers of the region (Table 4). Overall, it was the 4th most abundant species in Minnesota, with many collections approaching or exceeding 1000 specimens (Figure 9). Most adults were caught in July, with a few specimens found in June, August, and September.

**Figure 235.**
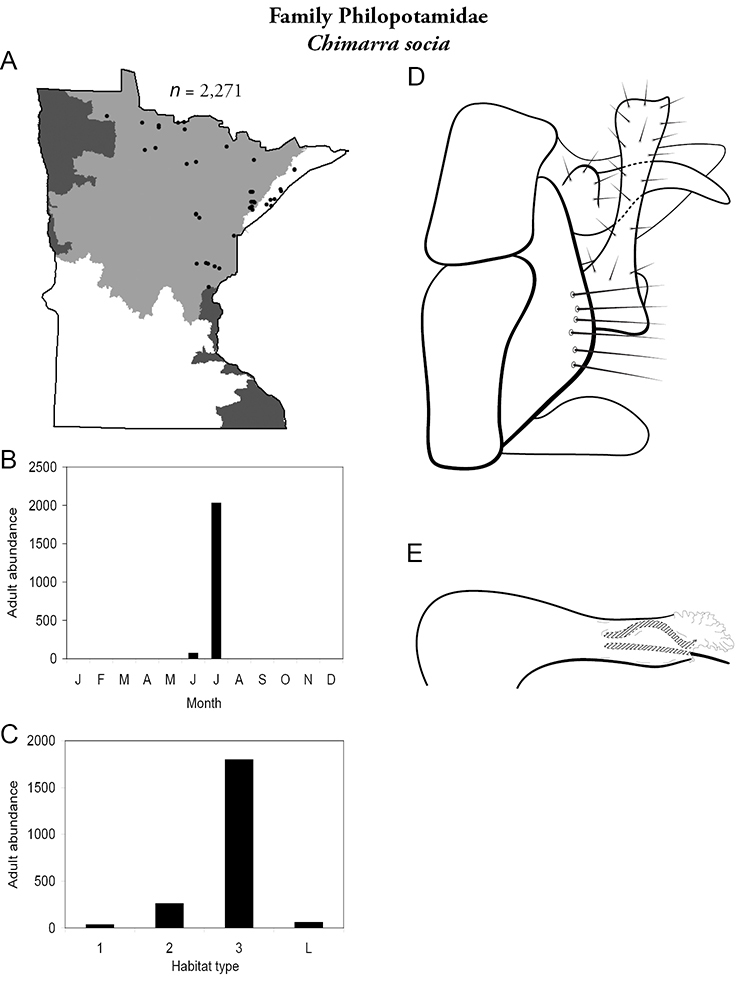
*Chimarra socia* **A** total specimens collected and all known collecting localities (Figure 4) **B** monthly adult abundance (1980s to present) **C** habitat preference (1980s to present) (Table 1) **D** male genital capsule **E** phallus.

***Chimarra socia*** (Figure 235) was found in the Lake Superior and Northern Regions. It was most abundant in large rivers, and was the 2nd most abundant species overall in large rivers of the Lake Superior Region (Table 3). Adults were found in June and, especially, July. In one large river, the Rainy River in Koochiching County, a specimen of *Chimarra socia* was discovered with 2 complete sets of male genitalia, one of the very few known cases of a “supermale” caddisfly (Houghton 2004c).

### Genus *Dolophilodes*

The genus *Dolophilodes* contains a single species in Minnesota. Wings of adults are brown in color with darker brown reticulations. For additional species, see Armitage (1991).

**Figure 236.**
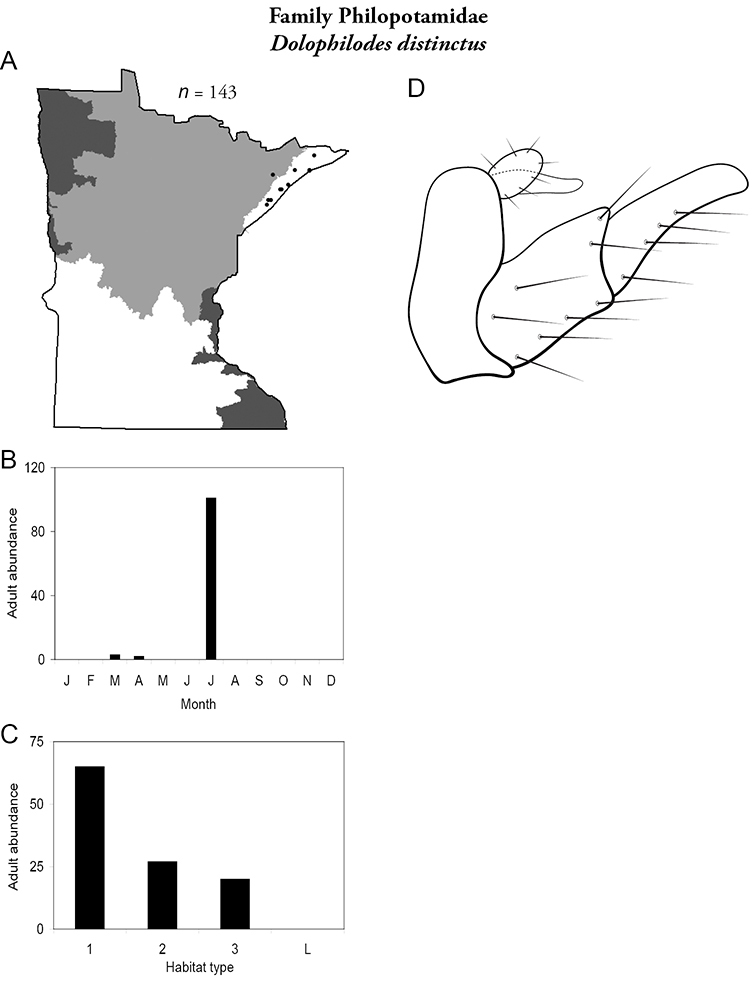
*Dolophilodes distinctus* **A** total specimens collected and all known collecting localities (Figure 4) **B** monthly adult abundance (1980s to present) **C** habitat preference (1980s to present) (Table 1) **D** male genital capsule.

***Dolophilodes distinctus*** (Figure 236) is known only from or near the Lake Superior Region, where it was common in small streams, and also found in medium and large rivers. It was the 3rd most abundant species in small streams of the region (Table 3). Adults were most abundant in July. Some specimens, including brachypterous females, emerged as early as March, breeding on the surface of the snow. These early emergent specimens were able to remain active for 4 days while contained within a 5°C darkened refrigerator. When placed in a ventilated container at 22° C, the individuals expired within 15 minutes (unpublished data).

### Genus *Wormaldia*

The genus *Wormaldia* contains a single species in Minnesota. For additional species, see Armitage (1991) or Munoz and Holzenthal (2008). Wings of adults are brown in color with darker brown reticulations.

**Figure 237.**
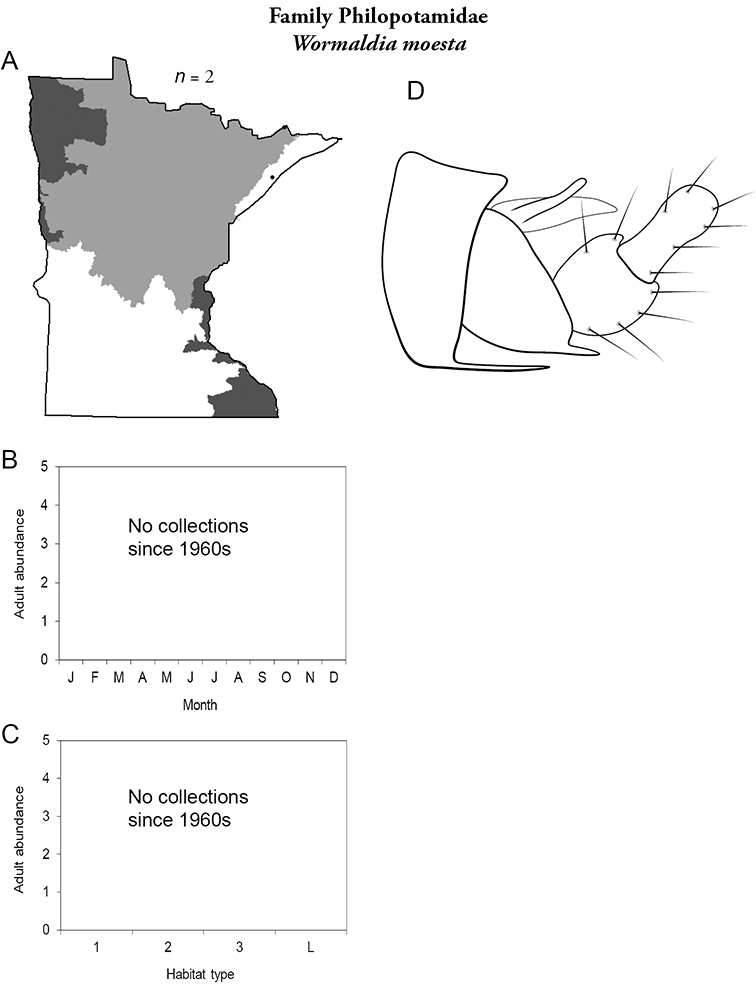
*Wormaldia moesta* **A** total specimens collected and all known collecting localities (Figure 4) **B** monthly adult abundance (1980s to present) **C** habitat preference (1980s to present) (Table 1) **D** male genital capsule.

***Wormaldia moesta*** (Figure 237) is known only from 2 specimens from northeastern Minnesota. It has not been collected since the 1960s. It is not clear if the species has been extirpated or is rare and difficult to collect.

### Family Phryganeidae

This family contains 8 genera in Minnesota: *Agrypnia*, *Banksiola*, *Beothukus*, *Fabria*, *Hagenella*, *Oligostomis*, *Phryganea*, and *Ptilostomis*, and a total of 18 species. For additional species of all genera, see Wiggins (1998). Larvae are usually shredders, although some may be omnivores (Wiggins 1996). Cases are tubular and usually constructed of organic material arranged in a concentric or spiral pattern. The phryganeids are some of the largest of all caddisflies, with adults ranging 10–35 mm in length.

Due to their large size and corresponding long lifespan, and also due to their shredder feeding habits, phryganeids appear to be very sensitive to habitat disturbance, especially the modification of the riparian canopy. Since the 1940s, phryganeid species have been extirpated from disturbed habitats in Minnesota, particularly those in the Northwestern and Southern Regions, at nearly 4× the rate of species in other families (Houghton and Holzenthal 2010). Three species: *Agrypnia glacialis*, *Banksiola dossuaria*, and *Banksiola smithi* have not been collected in Minnesota since the 1950s and may be extirpated from the entire state.

### Genus *Agrypnia*

The genus*Agrypnia* contains 6 species in Minnesota, although 1 is likely extirpated. Larvae can be either shredders or omnivores (Wiggins 1996) and are often found in both lakes and streams. Adults range 20–25 mm in length and are usually brown or grey in color with pronounced darker reticulations on the wings, except for *Agrypnia straminea* which is smaller and uniformly straw-colored (Figure 295).

**Figure 238.**
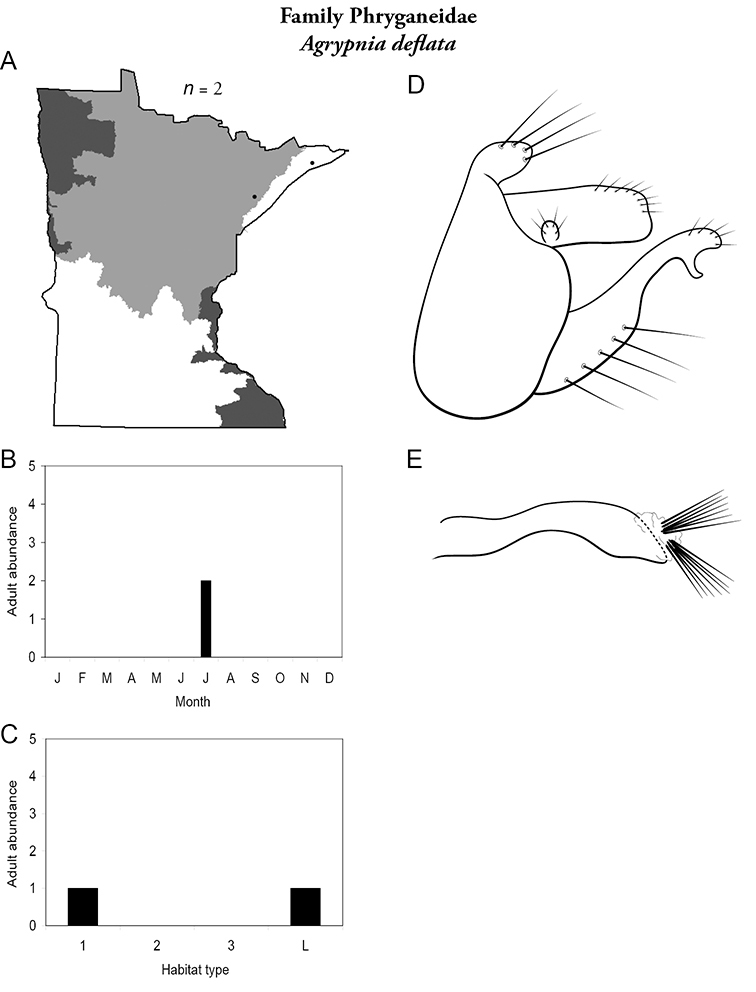
*Agrypnia deflata* **A** total specimens collected and all known collecting localities (Figure 4) **B** monthly adult abundance (1980s to present) **C** habitat preference (1980s to present) (Table 1) **D** male genital capsule **E** phallus.

***Agrypnia deflata*** (Figure 238) is known only from 2 specimens collected from a lake and small stream during July in and near the Lake Superior Region.

**Figure 239.**
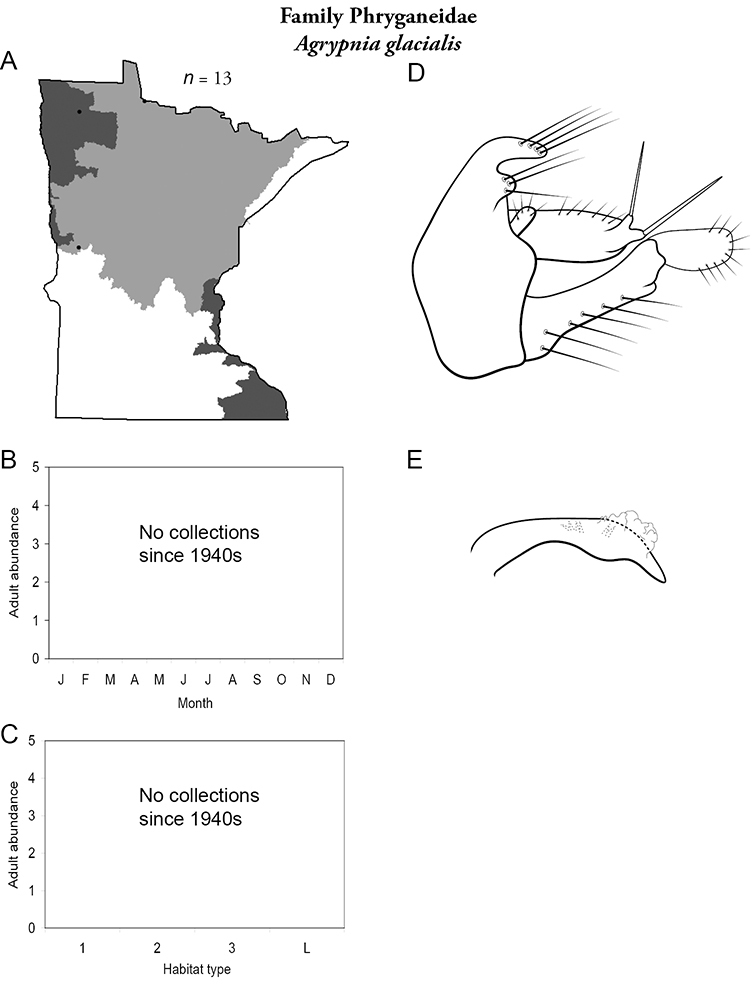
*Agrypnia glacialis* **A** total specimens collected and all known collecting localities (Figure 4) **B** monthly adult abundance (1980s to present) **C** habitat preference (1980s to present) (Table 1) **D** male genital capsule **E** phallus.

***Agrypnia glacialis*** (Figure 239) was collected several times between 1935 and 1941 in the northwestern portion of the state. It has not been collected in Minnesota since 1941 and is presumed extirpated from the state, likely due to agricultural disturbance throughout its historical range (Houghton 2007, Houghton and Holzenthal 2010).

**Figure 240.**
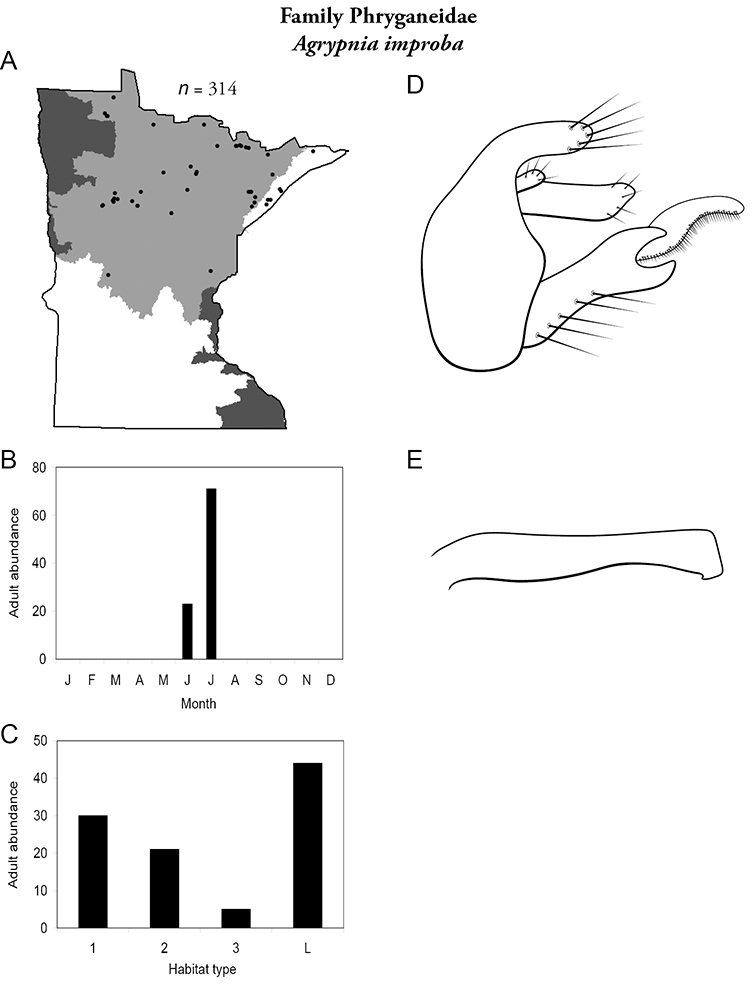
*Agrypnia improba* **A** total specimens collected and all known collecting localities (Figure 4) **B** monthly adult abundance (1980s to present) **C** habitat preference (1980s to present) (Table 1) **D** male genital capsule **E** phallus.

***Agrypnia improba*** (Figure 240) was found mainly in small and medium streams and from lakes. It is known only from the Lake Superior and Northern Regions. Adults were collected in June and July.

**Figure 241.**
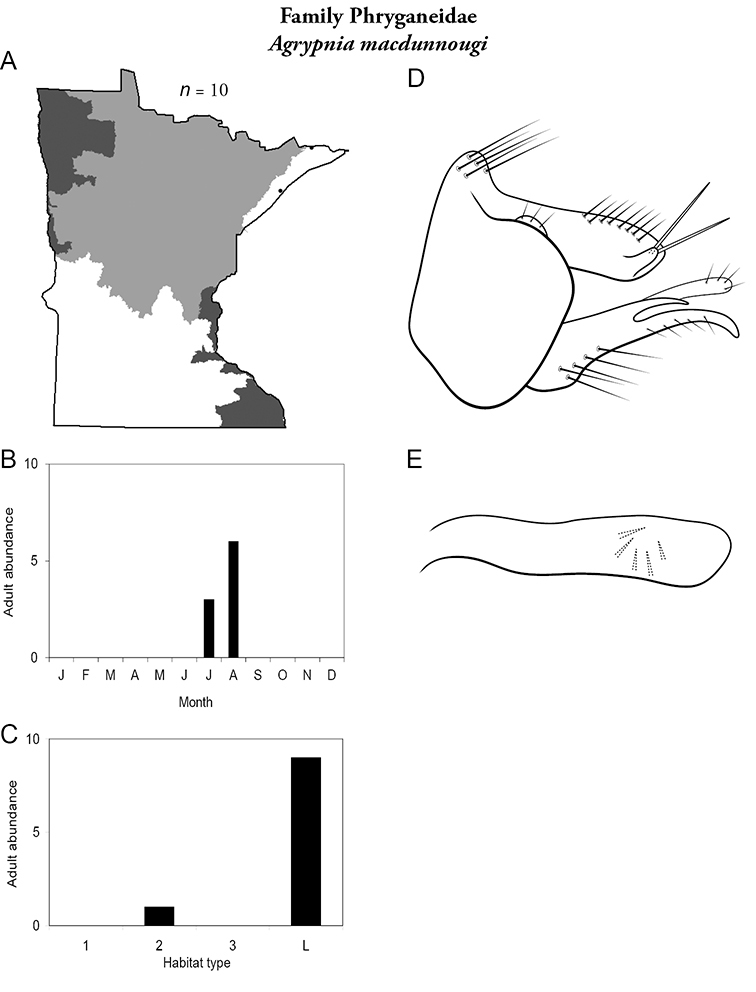
*Agrypnia macdunnoughi* **A** total specimens collected and all known collecting localities (Figure 4) **B** monthly adult abundance (1980s to present) **C** habitat preference (1980s to present) (Table 1) **D** male genital capsule **E** phallus.

***Agrypnia macdunnougi*** (Figure 241) is known only from a lake and medium stream in the Lake Superior Region. Specimens were collected in July and August.

**Figure 242.**
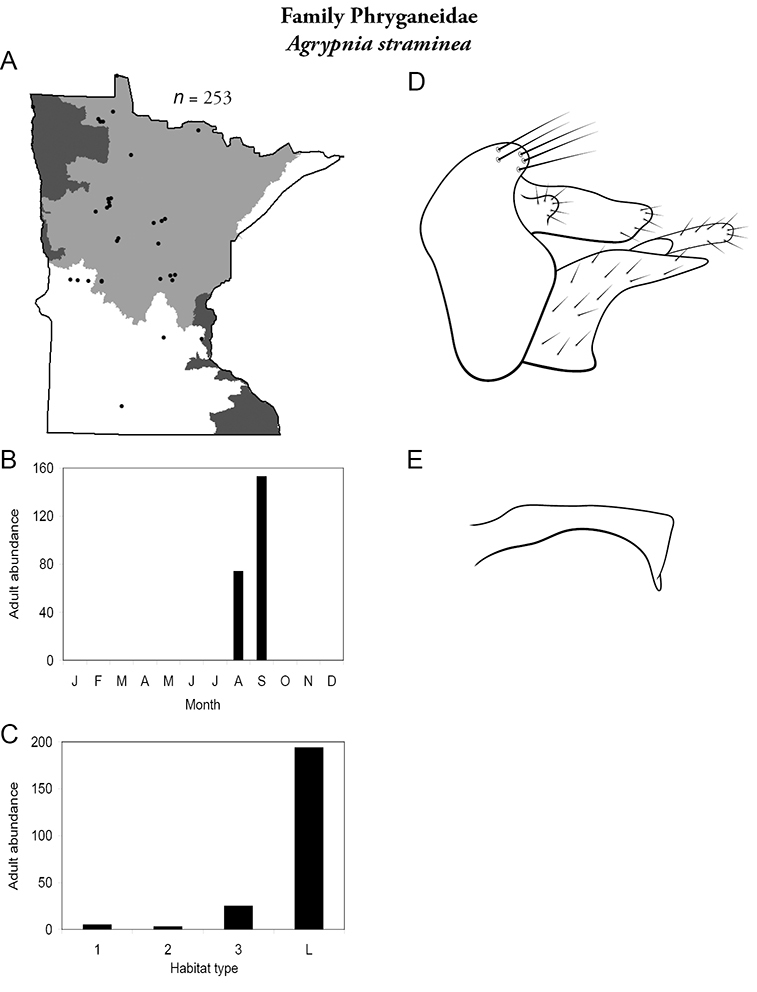
*Agrypnia straminea* **A** total specimens collected and all known collecting localities (Figure 4) **B** monthly adult abundance (1980s to present) **C** habitat preference (1980s to present) (Table 1) **D** male genital capsule **E** phallus.

***Agrypnia straminea*** (Figure 242) was the most abundant of the *Agrypnia* species. It was found mostly in lakes throughout the Northern and Southern Regions and collected during August and September.

**Figure 243.**
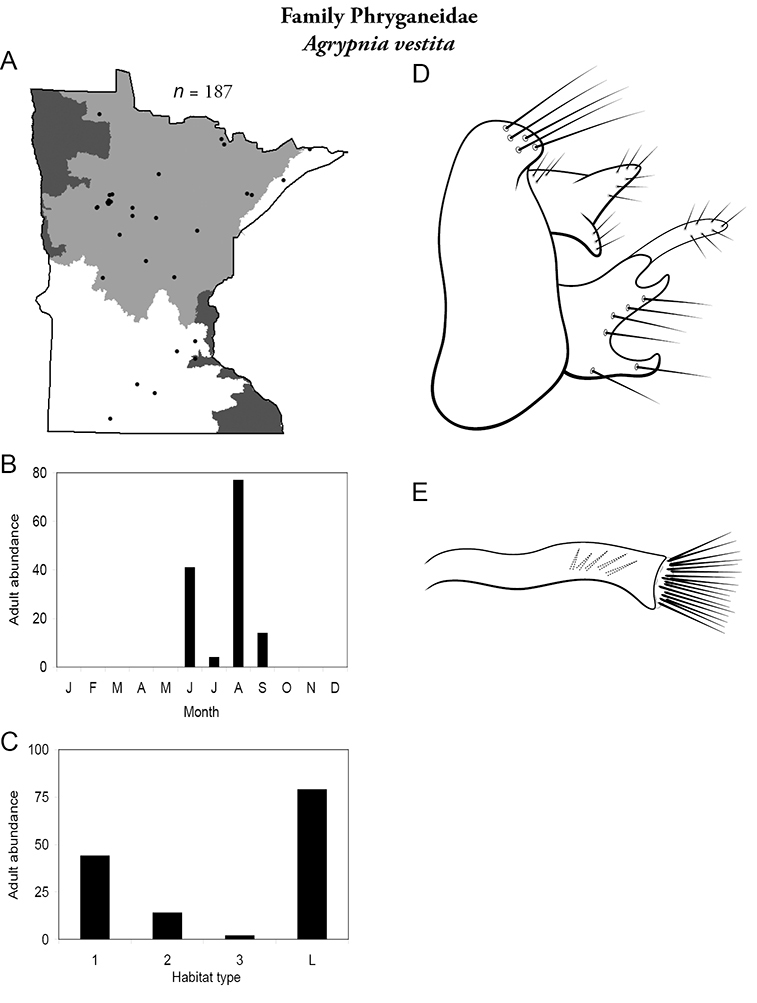
*Agrypnia vestita* **A** total specimens collected and all known collecting localities (Figure 4) **B** monthly adult abundance (1980s to present) **C** habitat preference (1980s to present) (Table 1) **D** male genital capsule **E** phallus.

***Agrypnia vestita*** (Figure 243) was found predominantly in the Northern and Southern Regions, typically in lakes, or less commonly in small and medium streams. Adults were present from June to September.

Two other *Agrypnia* species: *Agrypnia colorata* and *Agrypnia obsoleta* have been reported from northeastern Minnesota (MacLean 1995). The specimens of the former record were located, and re-identified as *Agrypnia straminea*, a species known throughout Minnesota. Specimens of the latter record were not located. The species, however, is predominantly European and restricted to only extreme northern North America (Wiggins 1998). It is unlikely to occur near Minnesota. It is likely that this record is actually of the similar *Agrypnia deflata*, which is known from northeastern Minnesota. Neither of these species are included in this manual.

### Genus *Banksiola*

The genus*Banksiola* contains 3 species in Minnesota. One is common and the other 2 appear extirpated from the state. Larvae inhabit lakes and wetlands as well as slow-moving areas of streams. They typically start out as shredders, but may become predatory in later instars (Wiggins 1996). Adult wings are usually brown or orange in color with distinctive dark reticulations (Figure 295). They are some of the smallest phryganeids, ranging 10–15 mm in length.

**Figure 244.**
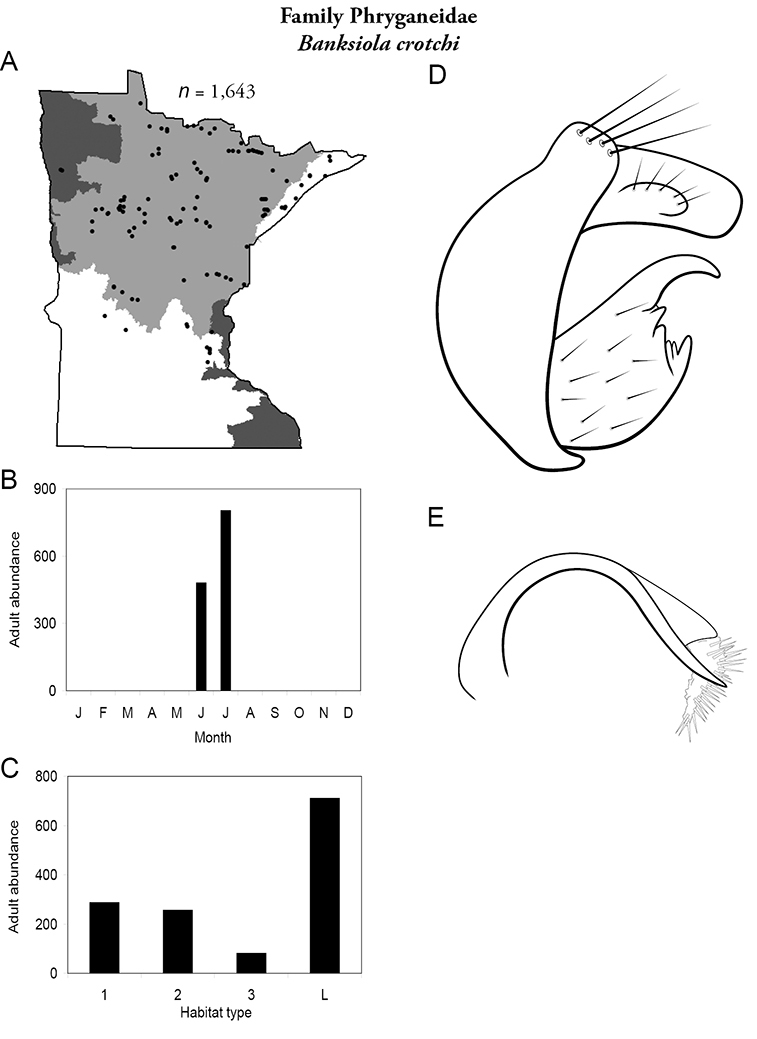
*Banksiola crotchi* **A** total specimens collected and all known collecting localities (Figure 4) **B** monthly adult abundance (1980s to present) **C** habitat preference (1980s to present) (Table 1) **D** male genital capsule **E** phallus.

***Banksiola crotchi*** (Figure 244) is the only species in the genus to have been collected since the 1950s. It was common and abundant throughout the Lake Superior and Northern Regions and found sporadically elsewhere. Adults were collected during June and July from small and medium streams and, more commonly, from lakes and wetlands. The presence of this species was determined by Houghton (2004b) to be one of the best indicators of undisturbed medium rivers in Minnesota.

**Figure 245.**
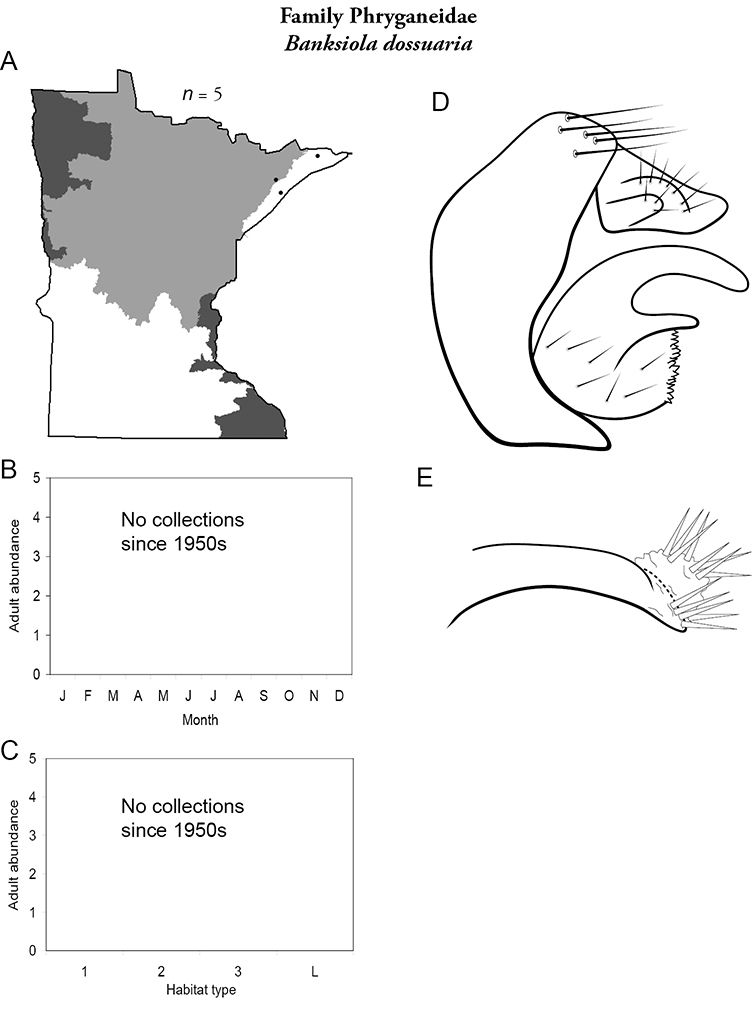
*Banksiola dossuaria* **A** total specimens collected and all known collecting localities (Figure 4) **B** monthly adult abundance (1980s to present) **C** habitat preference (1980s to present) (Table 1) **D** male genital capsule **E** phallus.

***Banksiola dossuaria*** (Figure 245) is known historically from and near the Lake Superior Region, but has not been collected since the 1950s. It appears to have always been rare, and so it is not clear if the species is extirpated from the state or merely difficult to collect (Houghton and Holzenthal 2010).

**Figure 246.**
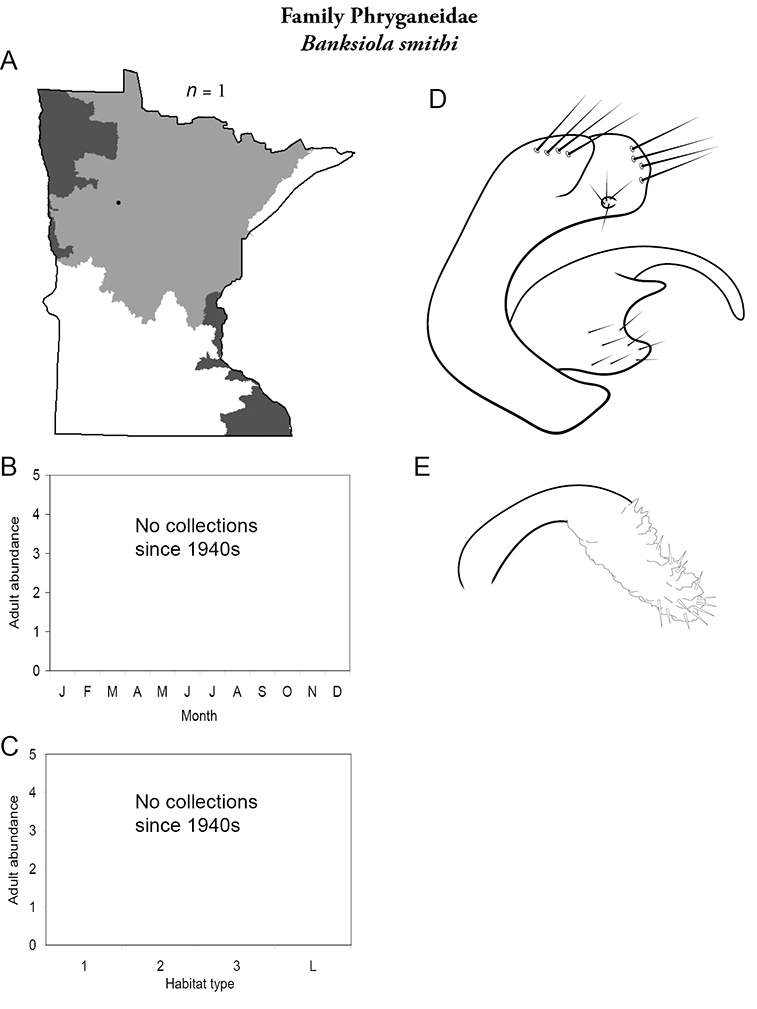
*Banksiola smithi* **A** total specimens collected and all known collecting localities (Figure 4) **B** monthly adult abundance (1980s to present) **C** habitat preference (1980s to present) (Table 1) **D** male genital capsule **E** phallus.

***Banksiola smithi*** (Figure 246) is known only from an unknown site in Lake Itasca State Park in the Northern Region. The date of collection and collector are both unknown, but the condition of the specimen and the type of vial used both appear to indicate the 1930–1940 era. It has not been collected since.

### Genus *Beothukus*

The genus *Beothukus* contains a single species in North America and in Minnesota. Larvae are known from bogs, including a sphagnum pool with a pH of 4.2 (Wiggins and Larson 1989). Larvae are omnivores, consuming algae, plants, and arthropods (Wiggins 1996). Adults are 10–15 mm in length and light brown in color.

**Figure 247.**
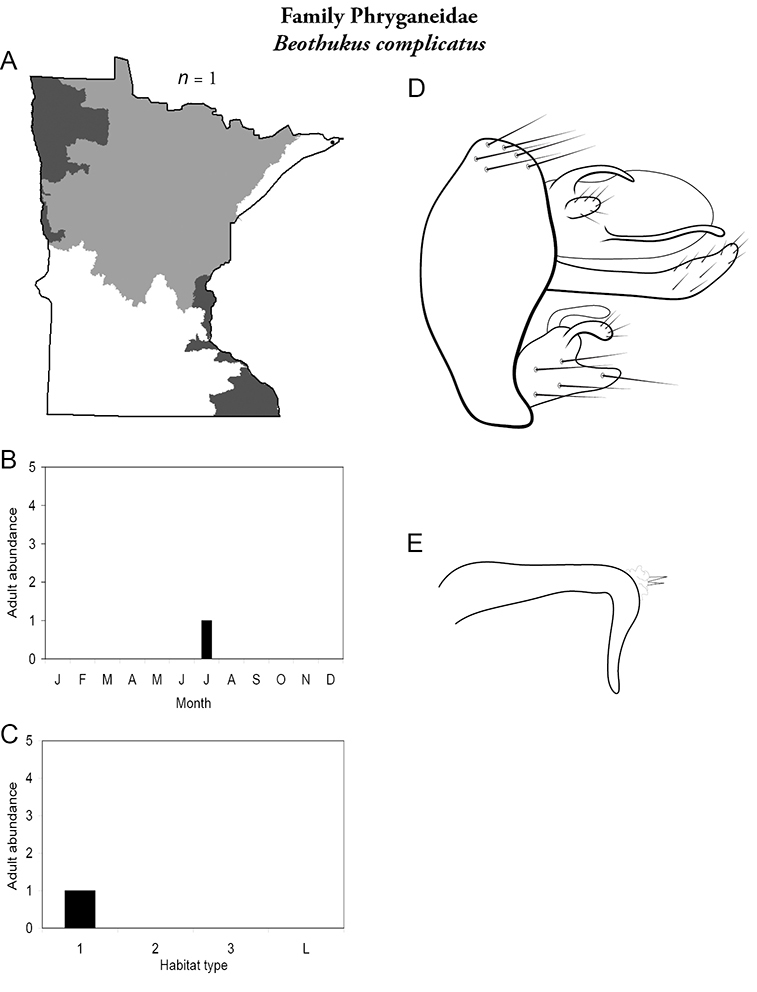
*Beothukus complicatus* **A** total specimens collected and all known collecting localities (Figure 4) **B** monthly adult abundance (1980s to present) **C** habitat preference (1980s to present) (Table 1) **D** male genital capsule **E** phallus.

***Beothukus complicatus*** (Figure 247) is known from only a single specimen collected from a small unnamed spring near Grand Portage National Monument in the Lake Superior region during July 2000.

### Genus *Fabria*

The genus *Fabria* contains a single species in North America and in Minnesota. Larvae inhabit lakes and slow-moving areas of streams, particularly areas with dense beds of aquatic plants (Wiggins 1996). Cases are unique among phryganeids in having long pieces of plant material trailing off of the end of the case and obscuring the spiral pattern. Adults are 15–20 mm in length and light brown in color.

**Figure 248.**
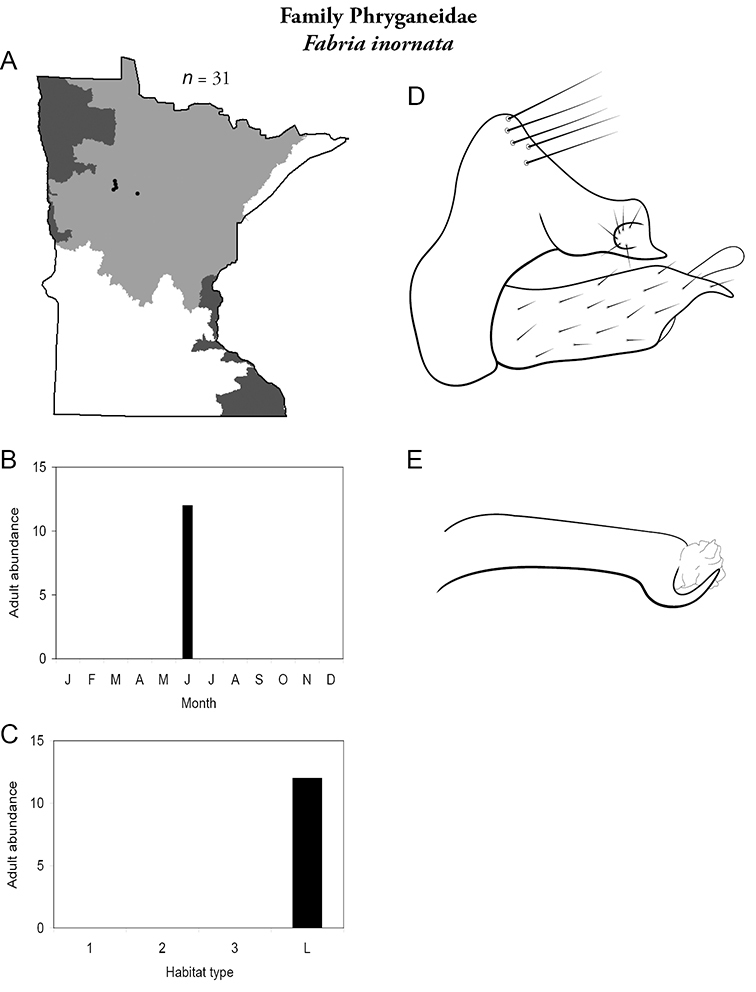
*Fabria inornata* **A** total specimens collected and all known collecting localities (Figure 4) **B** monthly adult abundance (1980s to present) **C** habitat preference (1980s to present) (Table 1) **D** male genital capsule **E** phallus.

***Fabria inornata*** (Figure 248) is known only from lakes in the area around Lake Itasca State Park in the Northern Region. Adults were found only in June.

### Genus *Hagenella*

The genus *Hagenella* contains a single species in Minnesota. Larvae live in slow-moving areas of streams. They construct cases of leaf pieces arranged in rings instead of the typical phryganeid spiral pattern (Wiggins 1996). Adults are 10–15 in length. Wings are yellow or orange in color with very dark and pronounced wing veins and reticulations.

**Figure 249.**
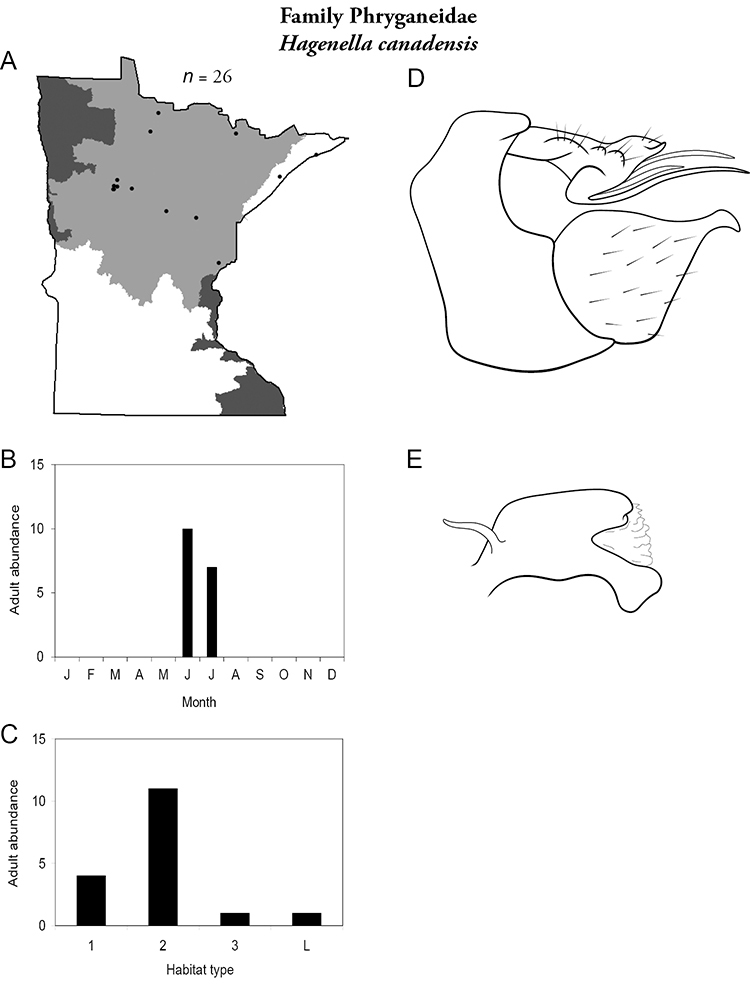
*Hagenella canadensis* **A** total specimens collected and all known collecting localities (Figure 4) **B** monthly adult abundance (1980s to present) **C** habitat preference (1980s to present) (Table 1) **D** male genital capsule **E** phallus.

***Hagenella canadensis*** (Figure 249) was found in the Lake Superior and Northern Regions, mostly in small and medium streams. All collections occurred in June and July.

### Genus *Oligostomis*

The genus *Oligostomis* contains a single species in Minnesota. Larvae inhabit slow-moving areas of cool woodland streams. They eat mostly vascular plant tissue, but also consume algae and small arthropods (Wiggins 1996). Larval cases are composed of pieces of leaves and bark arranged in a ring pattern. Adults range 12–15 mm and have yellow wings with dark brown reticulations. Adults of *Oligostomis* in North America are diurnal, and it is thought that their colorful wings serve an aposematic function (Wiggins 1998).

**Figure 250.**
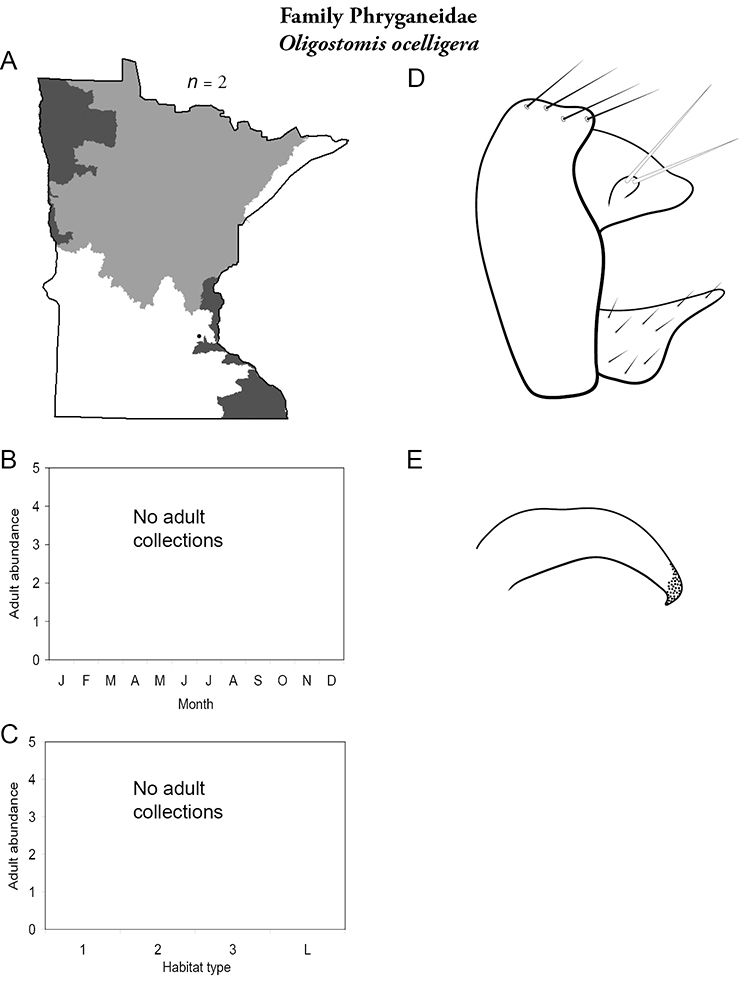
*Oligostomis ocelligera* **A** total specimens collected and all known collecting localities (Figure 4) **B** monthly adult abundance (1980s to present) **C** habitat preference (1980s to present) (Table 1) **D** male genital capsule **E** phallus.

***Oligostomis ocelligera*** (Figure 250) is known in Minnesota only from 2 larval specimens. These specimens have not been associated with an adult, and so it is possible that they may be of a different species. The range and size, however, are consistent with *Oligostomis ocelligera*, which has also been found in Wisconsin. The Minnesota larvae came from a very small stream of the Black Dog Wildlife Refuge in the Southern Region during December 1989.

### Genus *Phryganea*

The genus *Phryganea* contains 2 species in Minnesota. Larvae are typically found in lakes and slow-moving areas of streams. Specimens have been located 100 m deep in Lake Superior (Selgeby 1974). Larvae consume both plants and animals, depending on availability (Wiggins 1996). Adults are some of the largest of all caddisflies, ranging 25–35 mm in length. Wings are dark grey in color with dark brown mottling.

**Figure 251.**
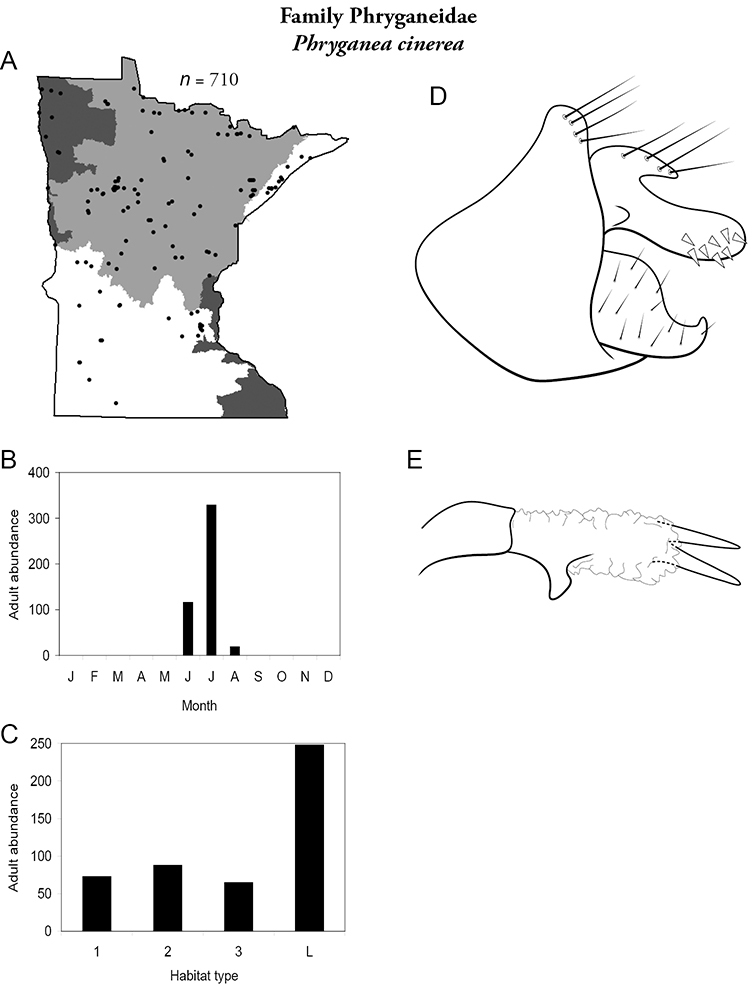
*Phryganea cinerea* **A** total specimens collected and all known collecting localities (Figure 4) **B** monthly adult abundance (1980s to present) **C** habitat preference (1980s to present) (Table 1) **D** male genital capsule **E** phallus.

***Phryganea cinerea*** (Figure 251) was found throughout all regions except the Southeastern. It was most abundant in lakes, but also found in all sizes of streams. Adults were present June through August.

**Figure 252.**
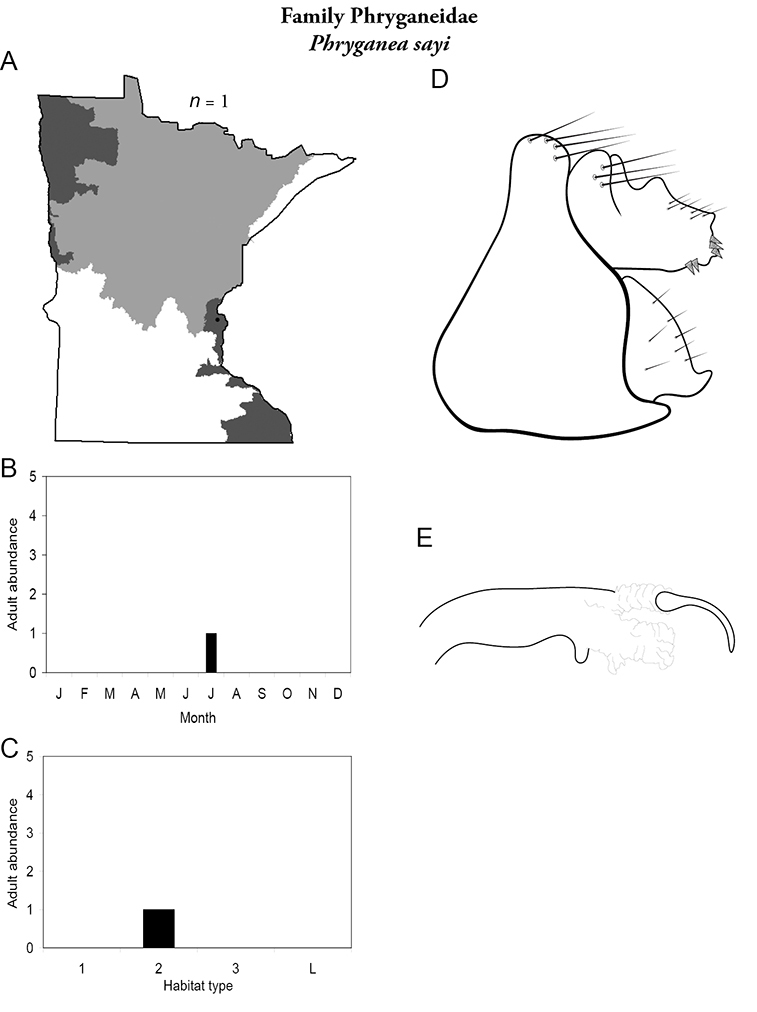
*Phryganea sayi* **A** total specimens collected and all known collecting localities (Figure 4) **B** monthly adult abundance (1980s to present) **C** habitat preference (1980s to present) (Table 1) **D** male genital capsule **E** phallus.

***Phryganea sayi*** (Figure 252) is only known in Minnesota from a single specimen collected from the Sunrise River, Chisago County, in the Southeastern Region during July 2004.

### Genus *Ptilostomis*

The genus *Ptilostomis* contains 3 species in Minnesota. Two of them are common throughout much of the state and frequently collected together. Larvae can occur in nearly any habitat, from small springs to vernal pools (Wiggins 1996). Larvae are primarily predatory. Adults are 25–30 mm in length. Forewings are amber-colored with dark brown reticulations and covered with yellow setae (Figure 295).

**Figure 253.**
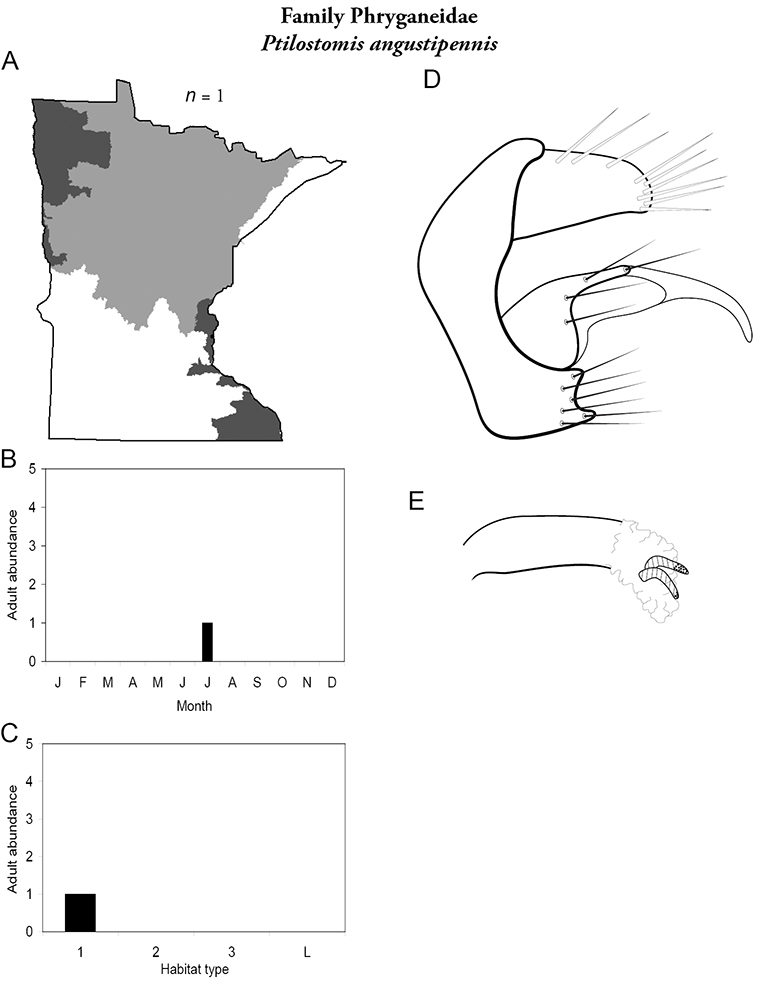
*Ptilostomis angustipennis* **A** total specimens collected and all known collecting localities (Figure 4) **B** monthly adult abundance (1980s to present) **C** habitat preference (1980s to present) (Table 1) **D** male genital capsule **E** phallus.

***Ptilostomis angustipennis*** (Figure 253) is known only from a single collection from Mill Creek, William O’Brien State Park, in the Southeastern Region during July 2002.

**Figure 254.**
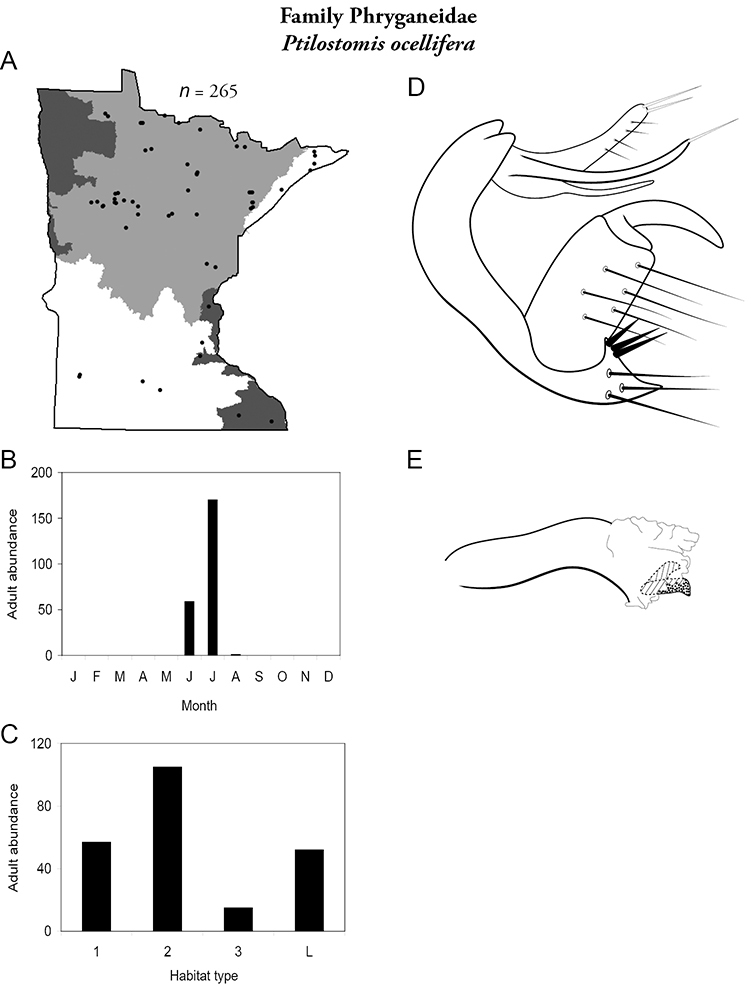
*Ptilostomis ocellifera* **A** total specimens collected and all known collecting localities (Figure 4) **B** monthly adult abundance (1980s to present) **C** habitat preference (1980s to present) (Table 1) **D** male genital capsule **E** phallus.

***Ptilostomis ocellifera*** (Figure 254) was found in all regions except the Northwestern. It was most common in lakes and small to medium streams. Adults were collected primarily in June and July.

**Figure 255.**
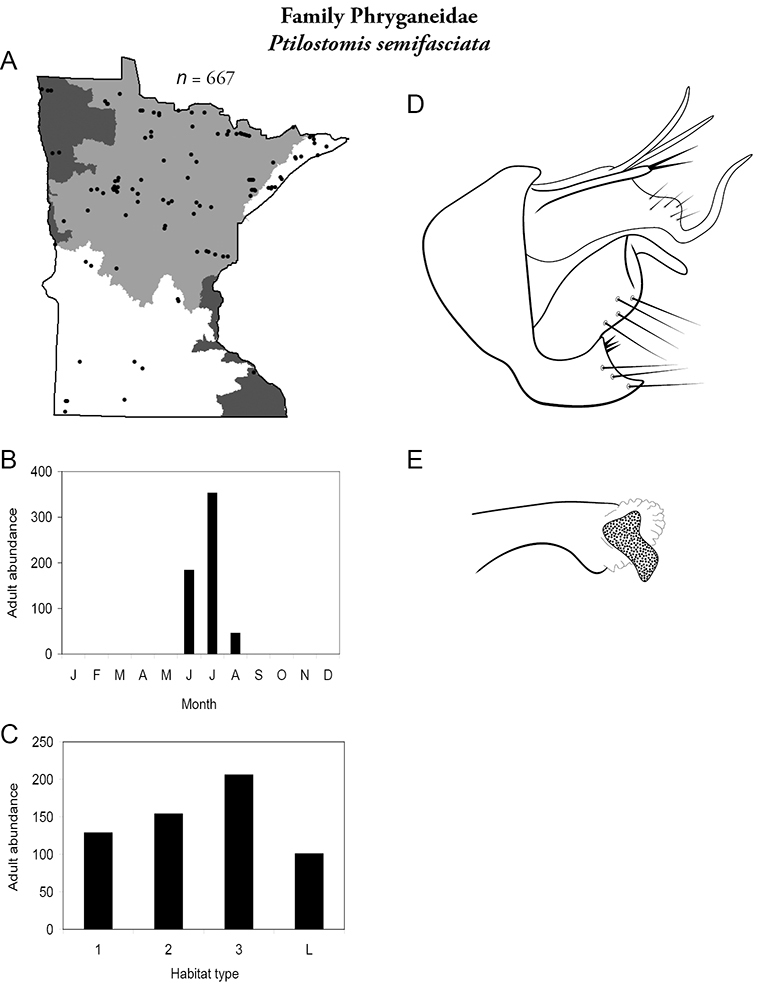
*Ptilostomis semifasciata* **A** total specimens collected and all known collecting localities (Figure 4) **B** monthly adult abundance (1980s to present) **C** habitat preference (1980s to present) (Table 1) **D** male genital capsule **E** phallus.

***Ptilostomis semifasciata*** (Figure 255) had a similar distribution and flight periodicity as *Ptilostomis ocellifera*, differing in its greater affinity for larger rivers and its presence in all regions.

### Family Polycentropodidae

This family contains 4 genera in Minnesota: *Cyrnellus*, *Neureclipsis*, *Nyctiophylax*, and *Polycentropus*, and a total of 25 species. It is the 5th most species-rich family (Figure 6). Larvae produce silken tubular retreats which are affixed to the undersides of rocks, and are typically either predators or detritivores (Wiggins 1996). Individual species exhibit a wide variety of habitat preferences, even within a genus. Adults range 5–12 mm in length. For additional species of all genera, see Armitage and Hamilton (1990).

### Genus *Cyrnellus*

The genus*Cyrnellus* contains a single species in North America and in Minnesota. Larvae are most abundant in large rivers, but can occur in nearly any habitat type. Larval retreats are flattened roofs of silk against a depression on the undersides of rocks (Wiggins 1996). Larvae are mainly detritivores. Adults are 6–8 mm in length and golden brown in color.

**Figure 256.**
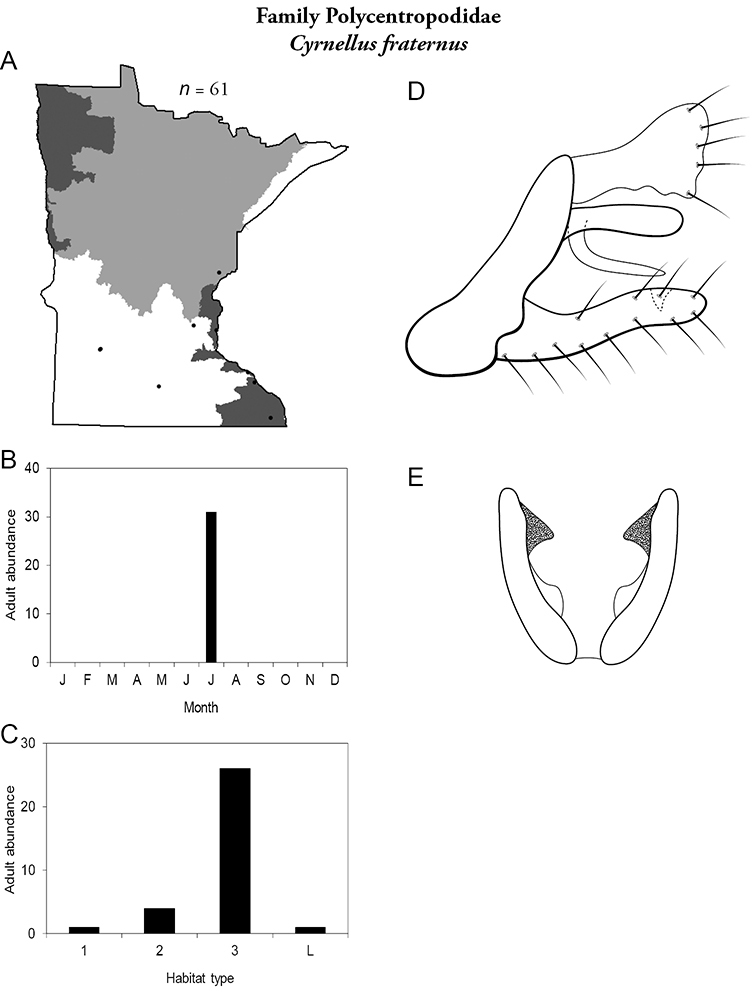
*Cyrnellus fraternus* **A** total specimens collected and all known collecting localities (Figure 4) **B** monthly adult abundance (1980s to present) **C** habitat preference (1980s to present) (Table 1) **D** male genital capsule **E** male inferior appendages (ventral view).

***Cyrnellus fraternus*** (Figure 256) has been collected sporadically, mainly from the Southeastern and Southern Regions. Adults were only found in July and were predominantly from large rivers.

### Genus *Neureclipsis*

The genus *Neureclipsis* contains 3 species in Minnesota. Larvae are found in streams of all sizes. They feed mainly on small arthropods caught in their trumpet-shaped capture nets (Wiggins 1996). Adults are 8–10 mm in length and brown in color.

**Figure 257.**
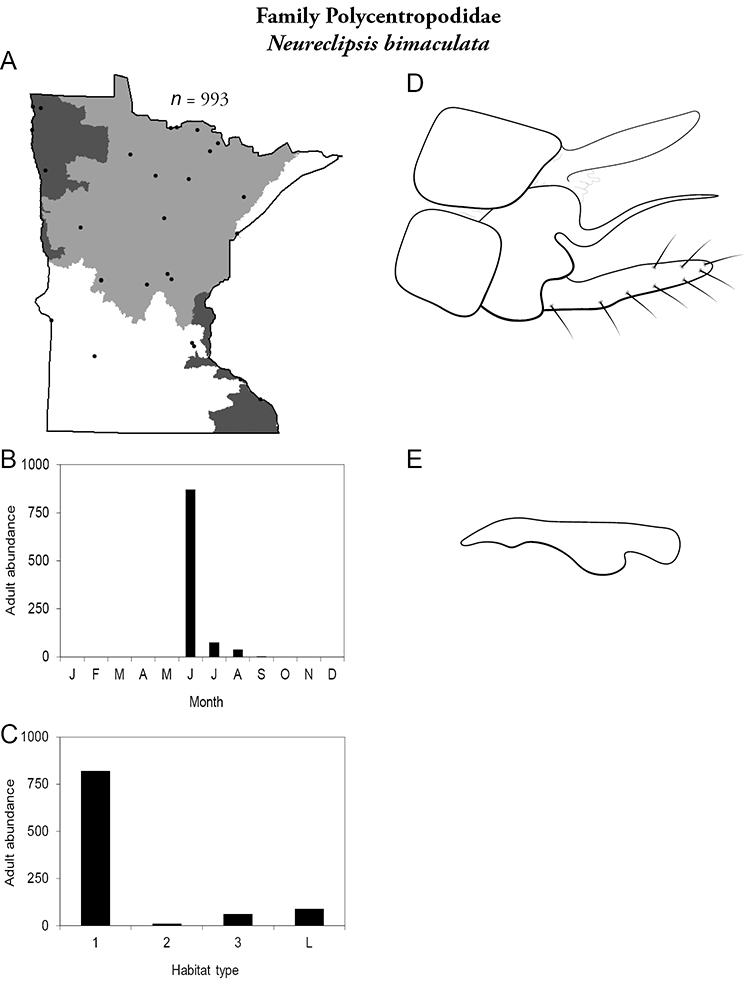
*Neureclipsis bimaculata* **A** total specimens collected and all known collecting localities (Figure 4) **B** monthly adult abundance (1980s to present) **C** habitat preference (1980s to present) (Table 1) **D** male genital capsule **E** phallus.

***Neureclipsis bimaculata*** (Figure 257) is known mainly from small streams. It has been found in all regions, but was most abundant in the Northern Region. Adults were most abundant in June, with some specimens present in July and August.

**Figure 258.**
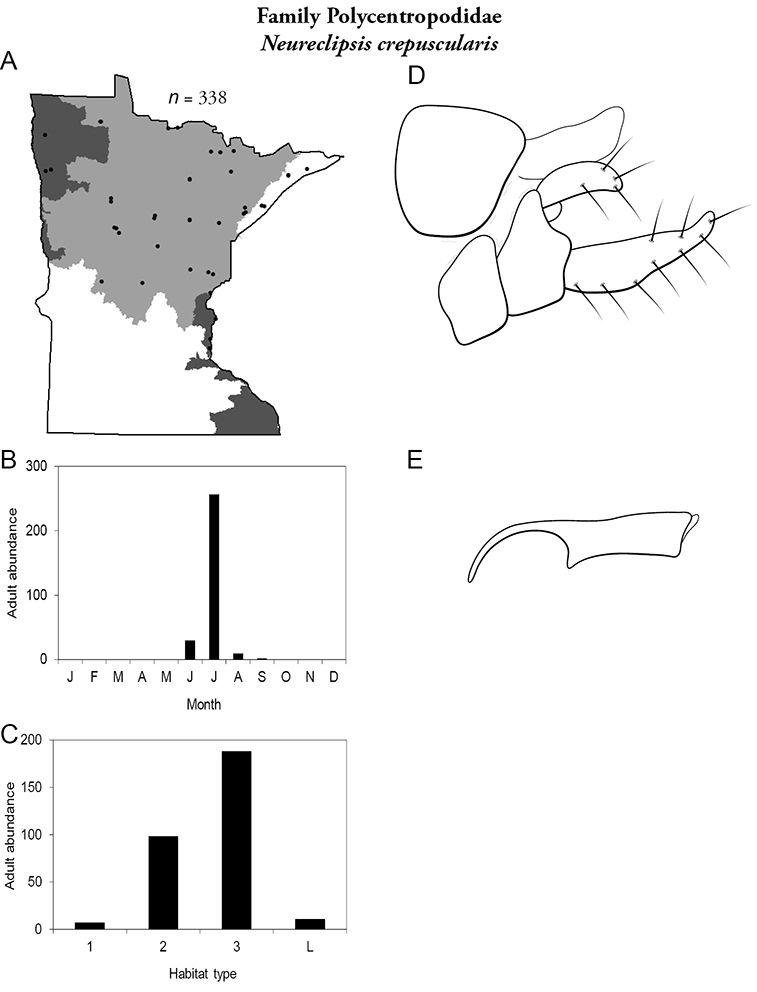
*Neureclipsis crepuscularis* **A** total specimens collected and all known collecting localities (Figure 4) **B** monthly adult abundance (1980s to present) **C** habitat preference (1980s to present) (Table 1) **D** male genital capsule **E** phallus.

***Neureclipsis crepuscularis*** (Figure 258) has been found in the northeastern 2/3 of the state, and is thus known from all regions except the Southern. It was found predominantly in streams, especially medium and large rivers. The majority of adults were found in July, with some in June, August, and September.

**Figure 259.**
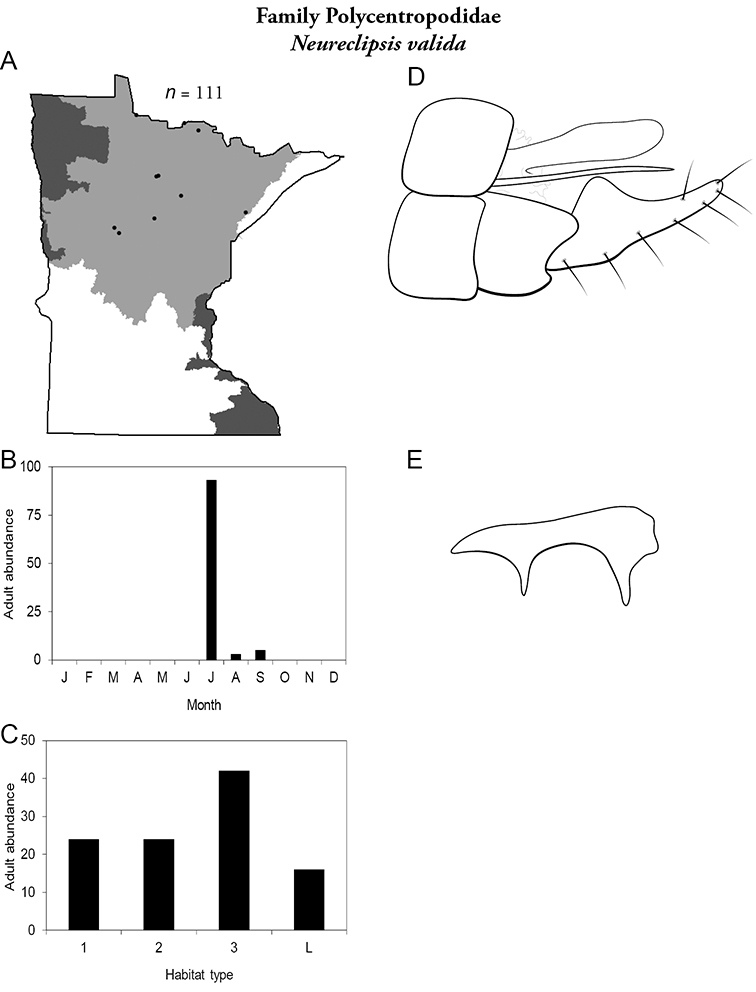
*Neureclipsis valida* **A** total specimens collected and all known collecting localities (Figure 4) **B** monthly adult abundance (1980s to present) **C** habitat preference (1980s to present) (Table 1) **D** male genital capsule **E** phallus.

***Neureclipsis valida*** (Figure 259) has only been collected in the Northern Region. It was found in all habitat types, with large rivers being the most common type. Adults were abundant in July, and present in August and September.

### Genus *Nyctiophylax*

The genus *Nyctiophylax* contains 4 species in Minnesota. For additional species, see Morse (1972). Larvae inhabit lakes and slow-moving areas of streams. Their retreat is similar to that of *Cyrnellus*: a flat silken roof over a depression in a rock or submerged tree branch (Wiggins 1996). Larvae are predatory, ambushing prey from their retreats.Adults are the smallest polycentropodids, usually around 5–6 mm in length. They are brown in color, with some darker reticulations. They are some of the most difficult caddisflies to identify to the species level due, in part, to phallic characteristics that show little variation between species. Thus, phalluses are not illustrated in this manual.

**Figure 260.**
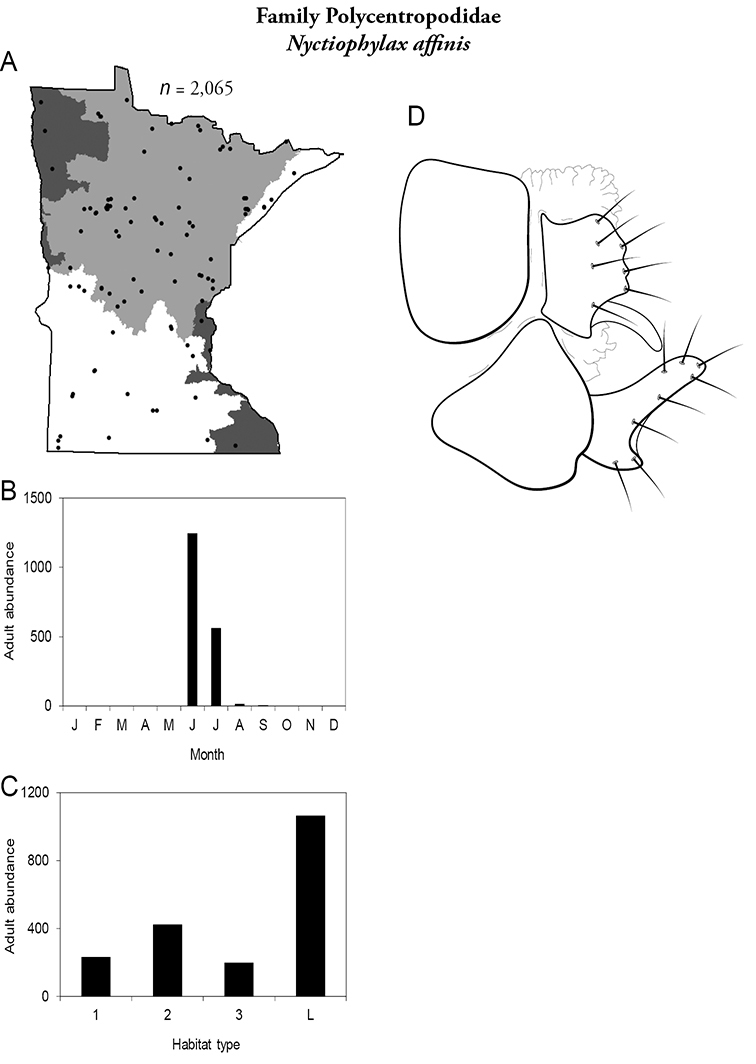
*Nyctiophylax affinis* **A** total specimens collected and all known collecting localities (Figure 4) **B** monthly adult abundance (1980s to present) **C** habitat preference (1980s to present) (Table 1) **D** male genital capsule.

***Nyctiophylax affinis*** (Figure 260) was the most widespread and abundant *Nyctiophylax* species, common in all regions. It was most abundant in lakes, although it occurred in all habitat types. Adults were collected mainly in June and July, with some presence in August.

**Figure 261.**
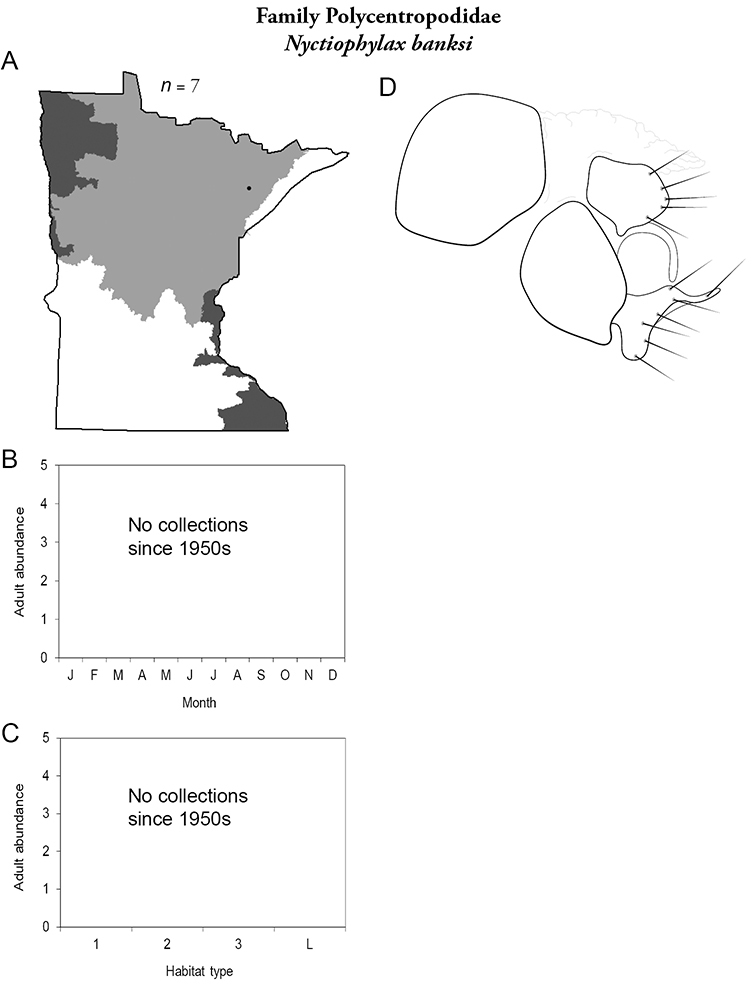
*Nyctiophylax banksi* **A** total specimens collected and all known collecting localities (Figure 4) **B** monthly adult abundance (1980s to present) **C** habitat preference (1980s to present) (Table 1) **D** male genital capsule.

***Nyctiophylax banksi*** (Figure 261) is known only from 6 specimens collected from Eaglenest Lake, Saint Louis County, in the Northern Region during 1957 and 1959. The specimens were accesioned into the Illinois Natural History Survey where they remain. The identify of the specimens as *Nyctiophylax banksi* was confirmed during this study. The species has not been found in Minnesota since these collections. It is known from South Carolina to Ontario, but is not widespread in any portion of its range (Morse 1972). It is not known if it is extirpated from Minnesota or is merely rare and difficult to collect.

**Figure 262.**
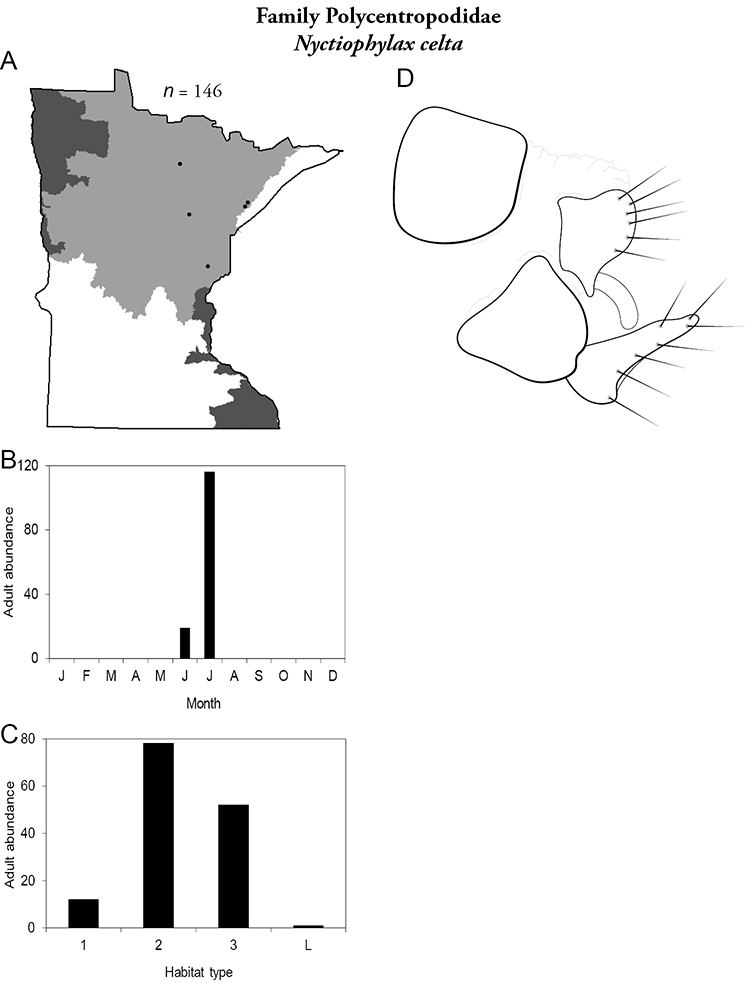
*Nyctiophylax celta* **A** total specimens collected and all known collecting localities (Figure 4) **B** monthly adult abundance (1980s to present) **C** habitat preference (1980s to present) (Table 1) **D** male genital capsule.

***Nyctiophylax celta*** (Figure 262) was only found in the Northern Region, mostly in July with some presence in June. It was found almost exclusively in rivers, particularly medium and large rivers.

**Figure 263.**
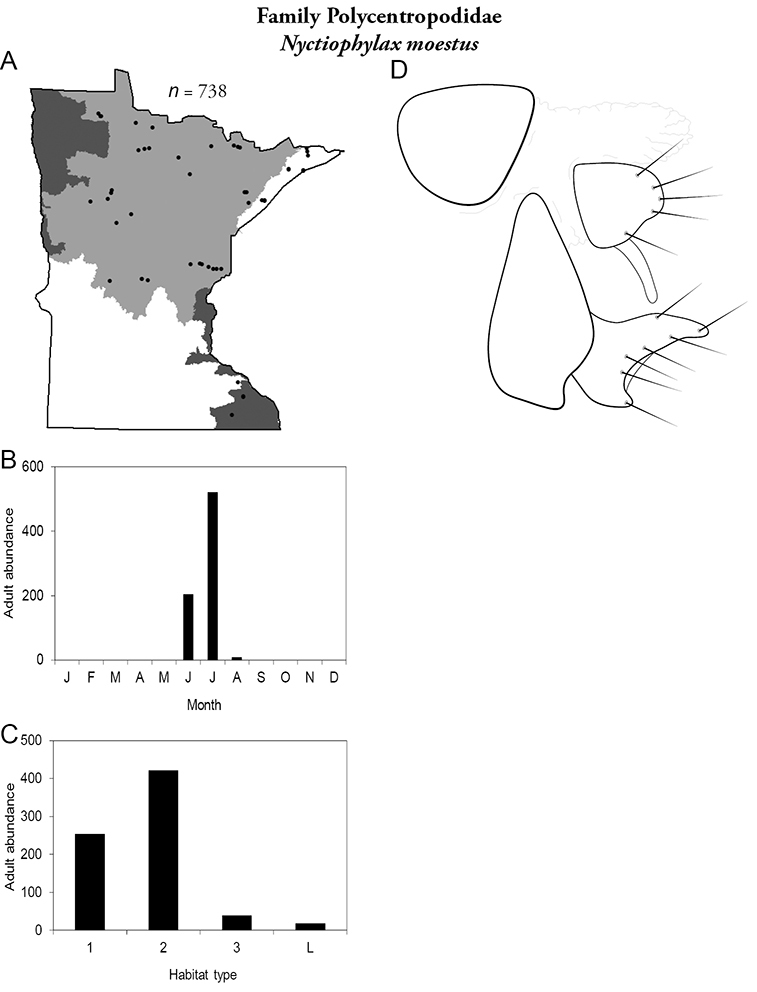
*Nyctiophylax moestus* **A** total specimens collected and all known collecting localities (Figure 4) **B** monthly adult abundance (1980s to present) **C** habitat preference (1980s to present) (Table 1) **D** male genital capsule.

***Nyctiophylax moestus*** (Figure 263) is known from the Lake Superior, Northern, and Southeastern Regions.Adults were mainly present in June and July. They were collected mostly in small and medium rivers.

### Genus *Polycentropus*

The genus*Polycentropus* contains 17 species in Minnesota. It is the 3rd most species-rich genus. Members are generally considered predators, but some of the smaller species may be detritivores (Wiggins 1996). Larvae inhabit many different types of lakes and streams, and many species exhibit no obvious habitat preference. Larval retreats are either silken tubes or bag-like structures. Adults range 6–12 mm in length. Wings are usually brown with darker reticulations, although some species are uniformly brown. Most species are known only from the northern portion of the state. Except for *Polycentropus cinereus* and *Polycentropus interruptus*, none of the species were abundant, and most collections yielded only one or a few male specimens. Females are usually much more abundant; unfortunately, they are not readily identifiable to the species level. Many of the Minnesota species have been recently been transfered into the genera *Holocentropus* and *Plectrocnemia* (Chamorro and Holzenthal 2011). This manual keeps all species in *Polycentropus*, but notes the new combinations.

**Figure 264.**
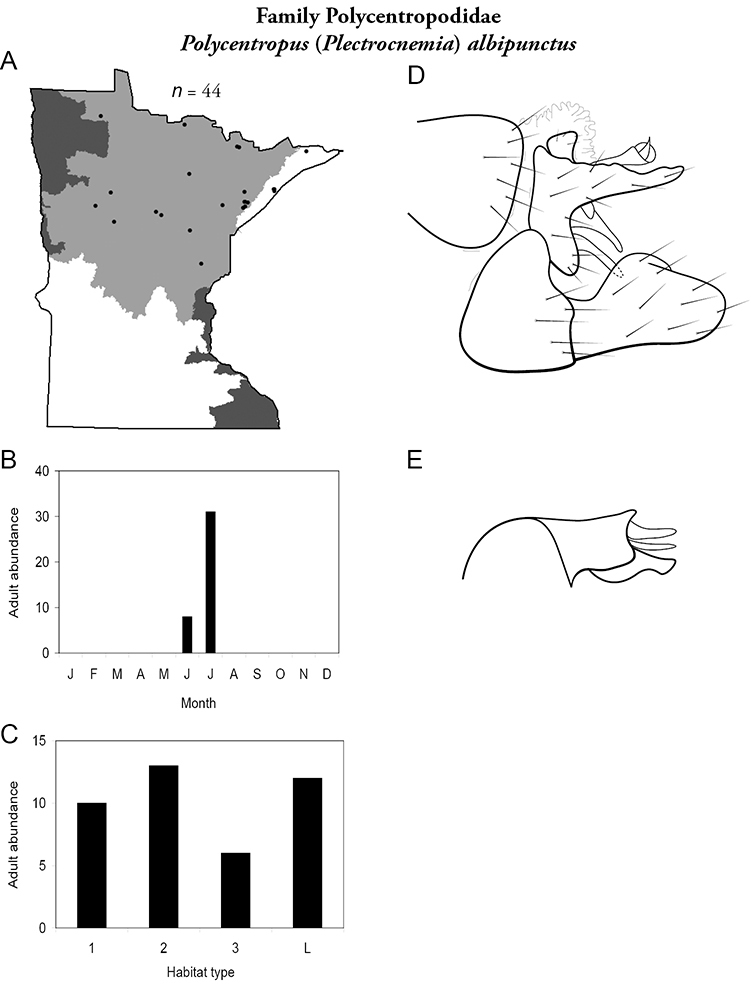
*Polycentropus albipunctus* **A** total specimens collected and all known collecting localities (Figure 4) **B** monthly adult abundance (1980s to present) **C** habitat preference (1980s to present) (Table 1) **D** male genital capsule **E** phallus.

***Polycentropus (Plectrocnemia) albipunctus*** (Figure 264) is known only from the Lake Superior and Northern regions. It was found in all habitat types, mainly during July, with a few specimens collected in June.

**Figure 265.**
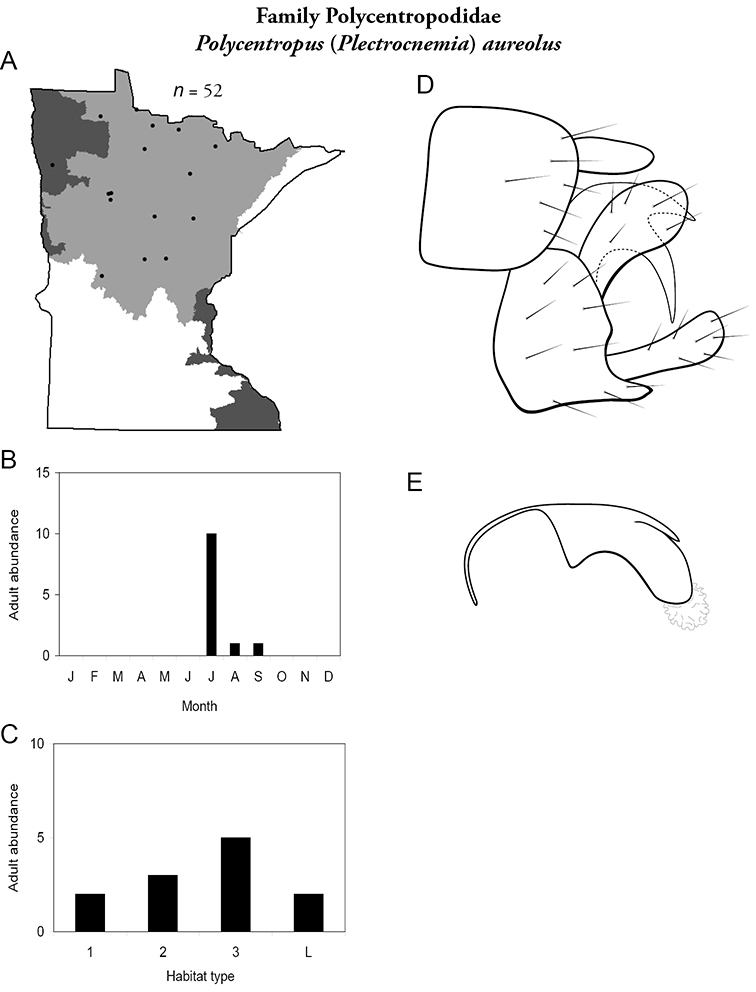
*Polycentropus aureolus* **A** total specimens collected and all known collecting localities (Figure 4) **B** monthly adult abundance (1980s to present) **C** habitat preference (1980s to present) (Table 1) **D** male genital capsule **E** phallus.

***Polycentropus (Plectrocnemia) aureolus*** (Figure 265) has been collected mainly from the Northern Region. It was found in all habitat types. Adults were most abundant in July, with some specimens in August and September.

**Figure 266.**
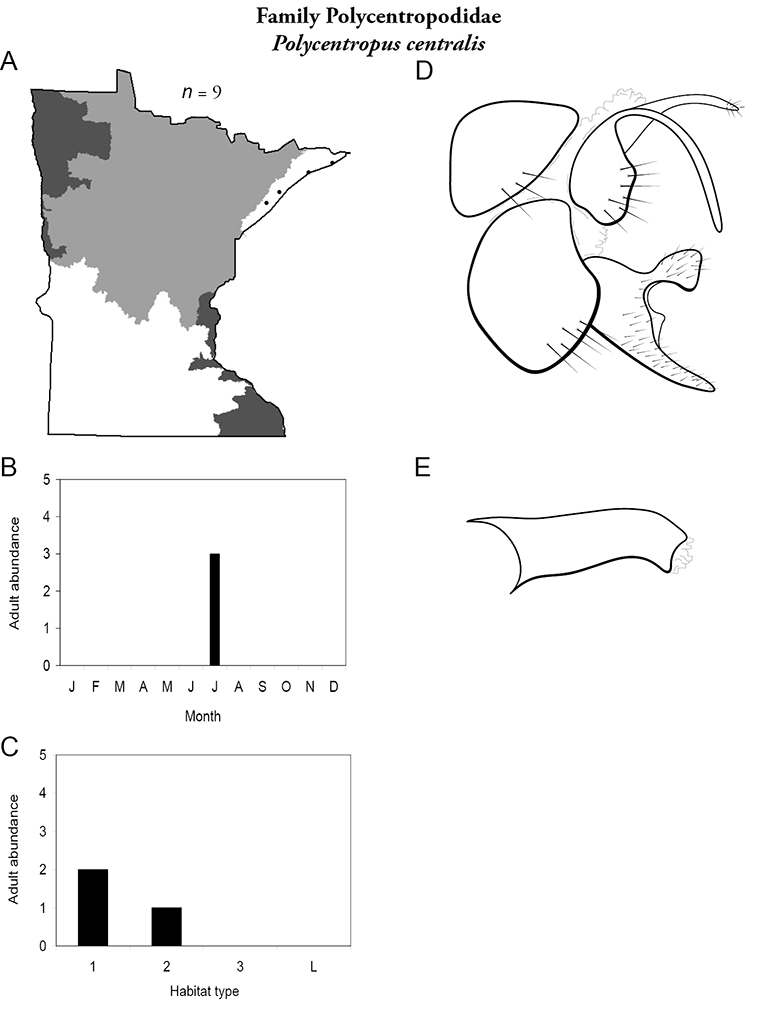
*Polycentropus centralis* **A** total specimens collected and all known collecting localities (Figure 4) **B** monthly adult abundance (1980s to present) **C** habitat preference (1980s to present) (Table 1) **D** male genital capsule **E** phallus.

***Polycentropus centralis*** (Figure 266) has only been collected from the Lake Superior Region during July. It was found in fast-moving small and medium rivers.

**Figure 267.**
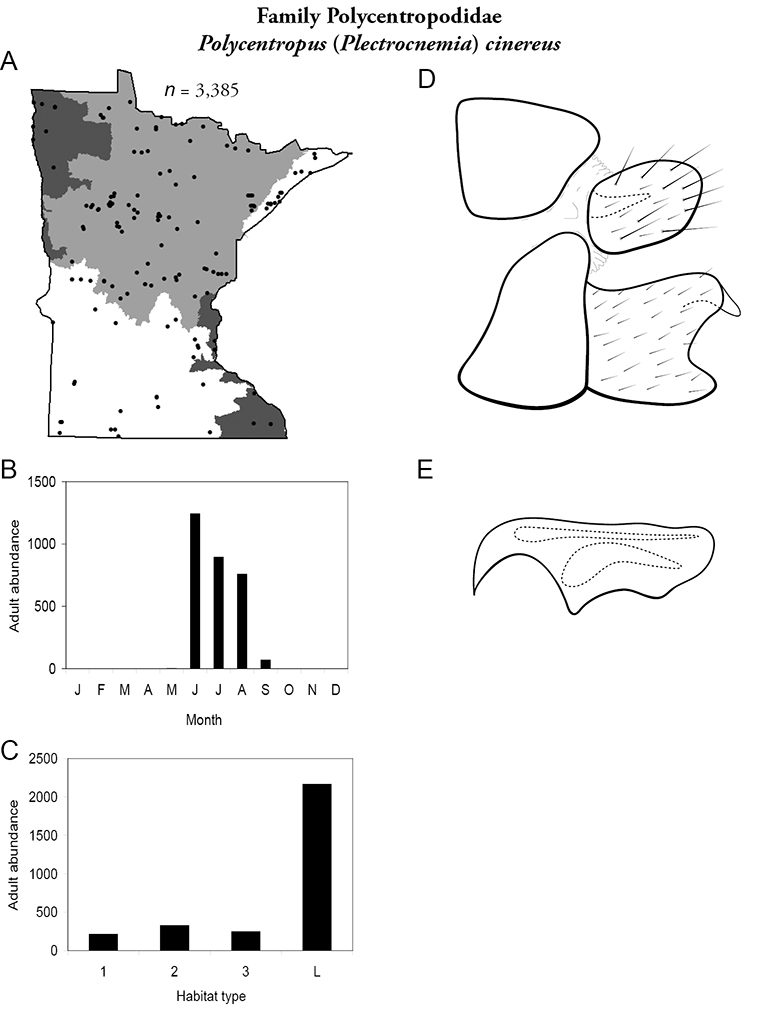
*Polycentropus cinereus* **A** total specimens collected and all known collecting localities (Figure 4) **B** monthly adult abundance (1980s to present) **C** habitat preference (1980s to present) (Table 1) **D** male genital capsule **E** phallus.

***Polycentropus (Plectrocnemia) cinereus*** (Figure 267) is the smallest *Polycentropus* species and also the most abundant. It was the 7th most widespread species in the state overall, and common in all regions (Figure 8). It was found predominantly in lakes and was the 2nd most abundant species in lakes of the Lake Superior Region (Table 3). Adults were abundant from June to August and present in September.

**Figure 268.**
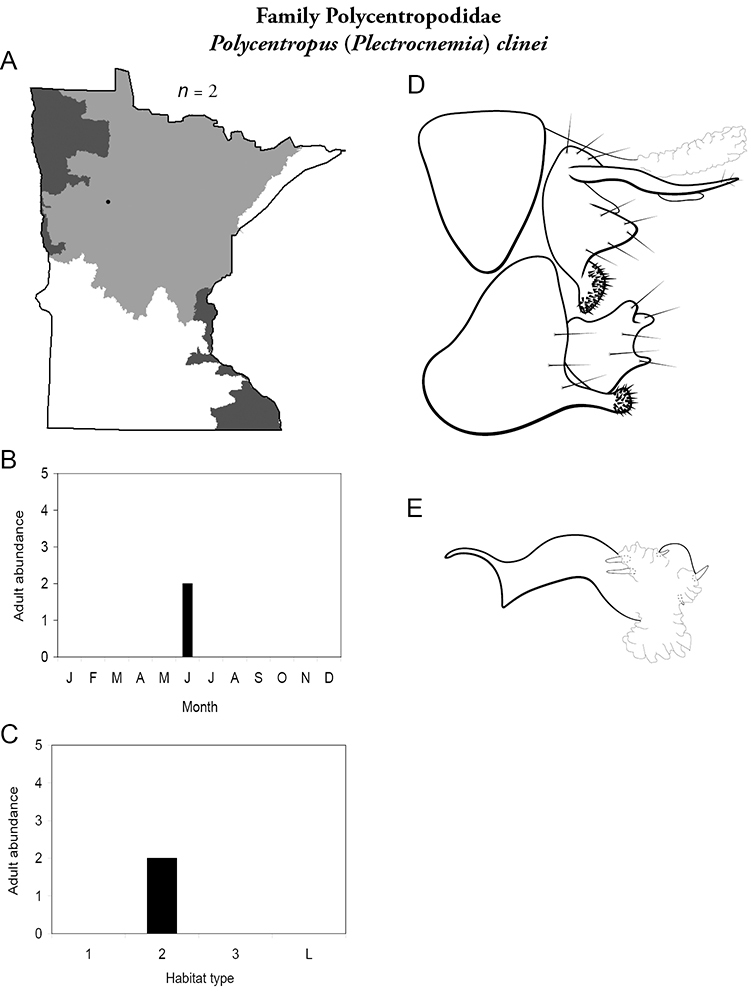
*Polycentropus clinei* **A** total specimens collected and all known collecting localities (Figure 4) **B** monthly adult abundance (1980s to present) **C** habitat preference (1980s to present) (Table 1) **D** male genital capsule **E** phallus.

***Polycentropus (Plectrocnemia) clinei*** (Figure 268) is known only from 2 specimens collected from Nicollet Creek, Clearwater County, in the Northern Region during June 1989.

**Figure 269.**
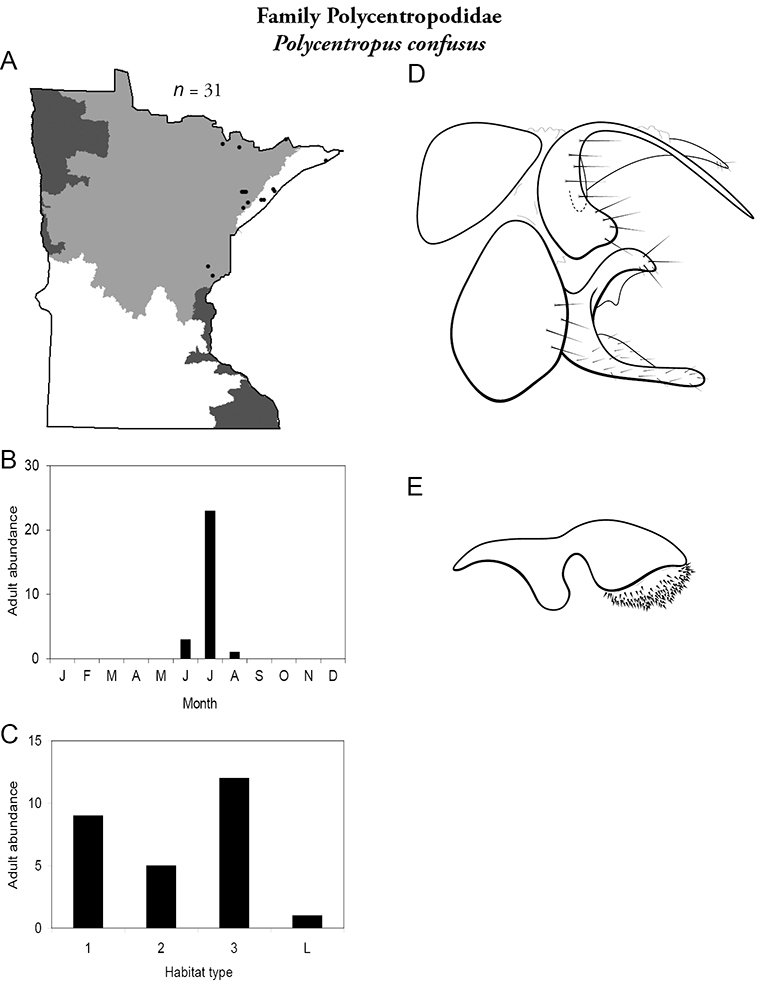
*Polycentropus confusus* **A** total specimens collected and all known collecting localities (Figure 4) **B** monthly adult abundance (1980s to present) **C** habitat preference (1980s to present) (Table 1) **D** male genital capsule **E** phallus.

***Polycentropus confusus*** (Figure 269) has been collected from the Lake Superior and Northern Regions, mainly during July. Some specimens were found in June and August. It was found mainly in streams of all sizes.

**Figure 270.**
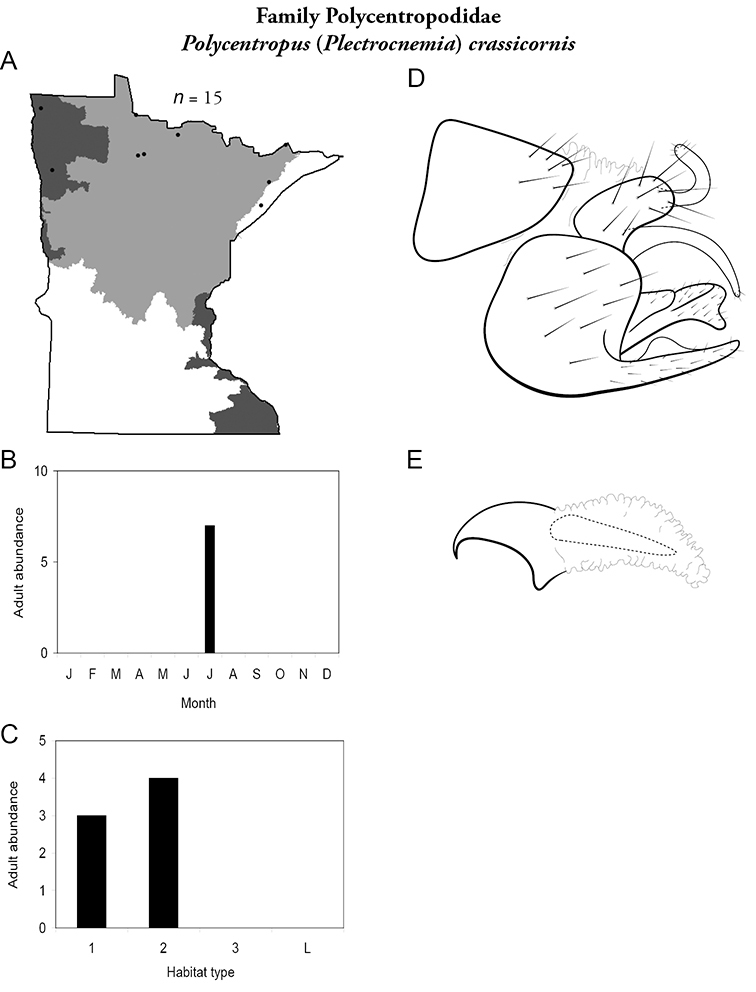
*Polycentropus crassicornis* **A** total specimens collected and all known collecting localities (Figure 4) **B** monthly adult abundance (1980s to present) **C** habitat preference (1980s to present) (Table 1) **D** male genital capsule **E** phallus.

***Polycentropus (Plectrocnemia) crassicornis*** (Figure 270) has been found sporadically in the northern third of the state, in the Lake Superior, Northern, and Northwestern Regions. All specimens were collected in July from small and medium streams.

**Figure 271.**
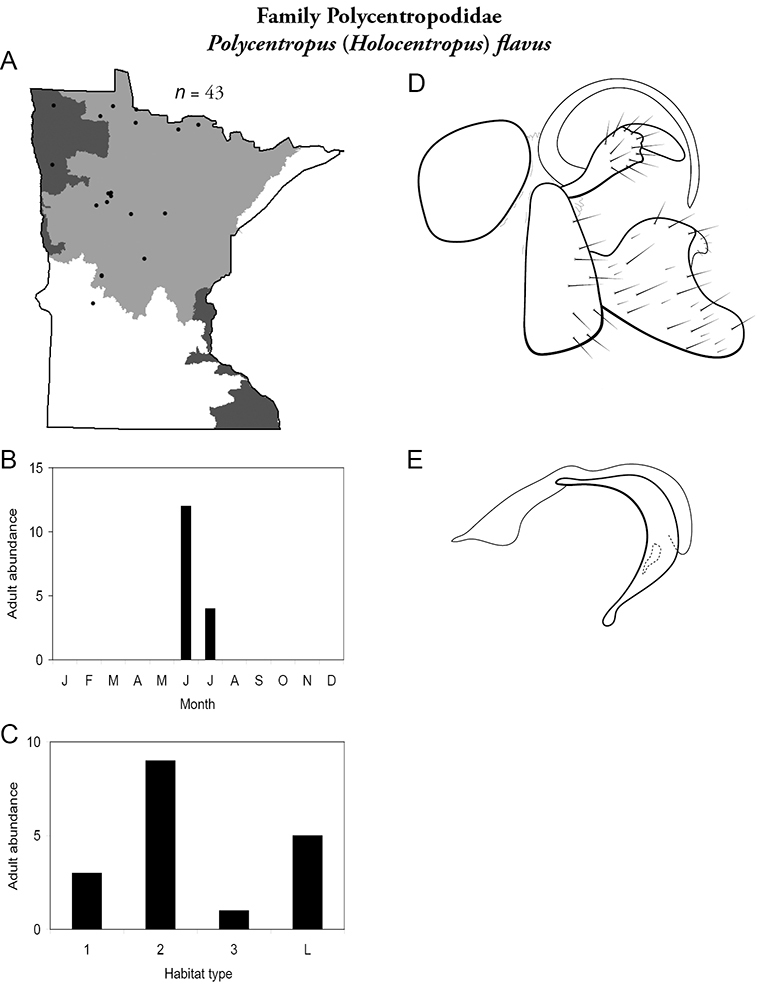
*Polycentropus flavus* **A** total specimens collected and all known collecting localities (Figure 4) **B** monthly adult abundance (1980s to present) **C** habitat preference (1980s to present) (Table 1) **D** male genital capsule **E** phallus.

***Polycentropus (Holocentropus) flavus*** (Figure 271) has been collected mostly from the Northern Region, with some specimens known historically from the Northwestern and Southern Regions. Adults were found in June and July, mainly from lakes and medium rivers.

**Figure 272.**
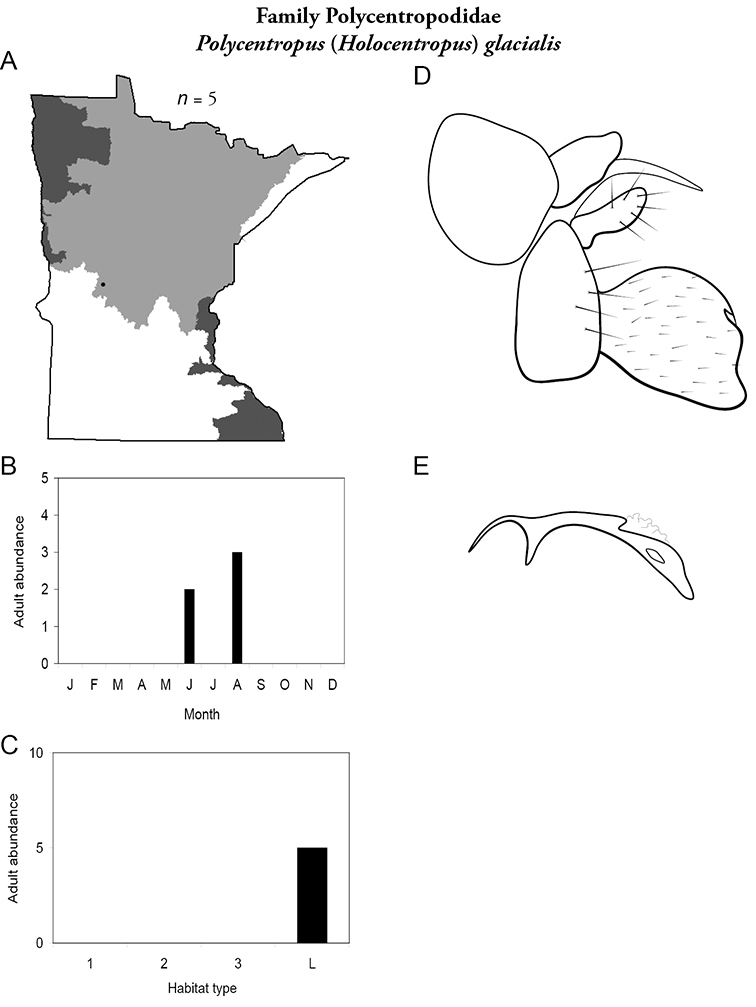
*Polycentropus glacialis* **A** total specimens collected and all known collecting localities (Figure 4) **B** monthly adult abundance (1980s to present) **C** habitat preference (1980s to present) (Table 1) **D** male genital capsule **E** phallus.

***Polycentropus (Holocentropus) glacialis*** (Figure 272) is known only from 2 collections from Lake Carlos, Lake Carlos State Park, in the Northern Region. Adults were found in June and August; no collection attempt was made in July. Due to the rarity of the species in Minnesota, and the vulnerability of its only known habitat (Houghton 2007), the Minnesota Department of Natural Resources has propsed “Threatened” status for *Polycentropus glacialis* (MNDNR 2012).

**Figure 273.**
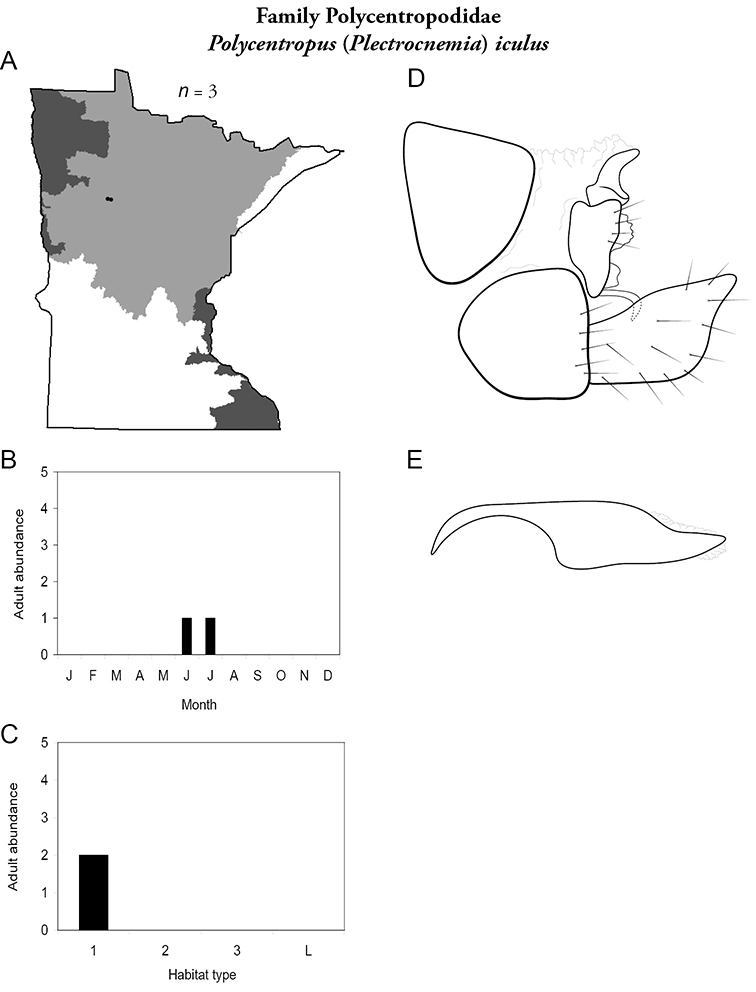
*Polycentropus iculus* **A** total specimens collected and all known collecting localities (Figure 4) **B** monthly adult abundance (1980s to present) **C** habitat preference (1980s to present) (Table 1) **D** male genital capsule **E** phallus.

***Polycentropus (Plectrocnemia) iculus*** (Figure 273) is known only from specimens collected from 2 small streams in Lake Itasca State Park in the Northern Region during June and July.

**Figure 274.**
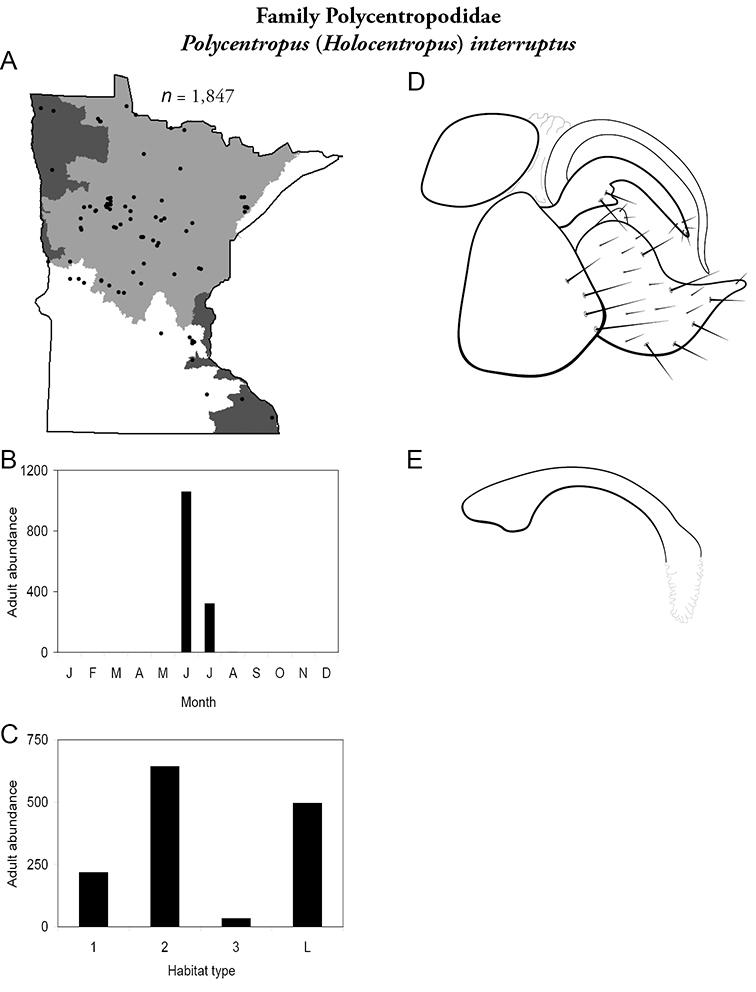
*Polycentropus interruptus* **A** total specimens collected and all known collecting localities (Figure 4) **B** monthly adult abundance (1980s to present) **C** habitat preference (1980s to present) (Table 1) **D** male genital capsule **E** phallus.

***Polycentropus (Holocentropus) interruptus*** (Figure 274) was found in all regions, particularly the Northern Region. It was most abundant in lakes and medium rivers. Adults were present in June and July.

**Figure 275.**
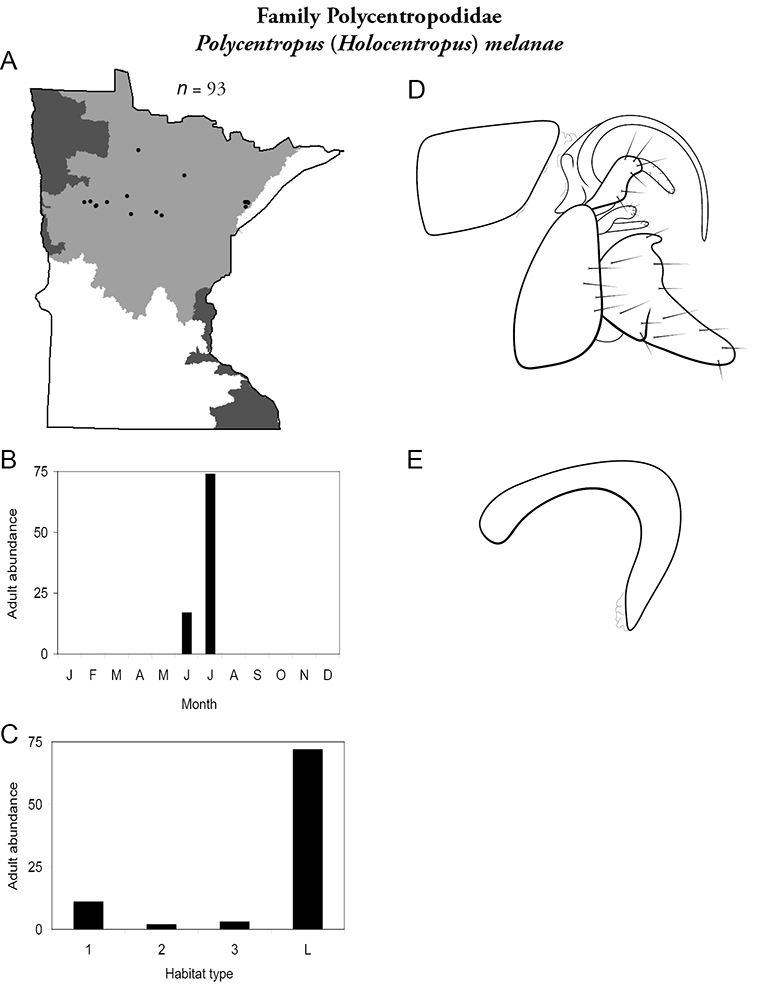
*Polycentropus melanae* **A** total specimens collected and all known collecting localities (Figure 4) **B** monthly adult abundance (1980s to present) **C** habitat preference (1980s to present) (Table 1) **D** male genital capsule **E** phallus.

***Polycentropus (Holocentropus) melanae*** (Figure 275) was only collected in the Northern Region in June and, especially, July. It was found almost exclusively in lakes.

**Figure 276.**
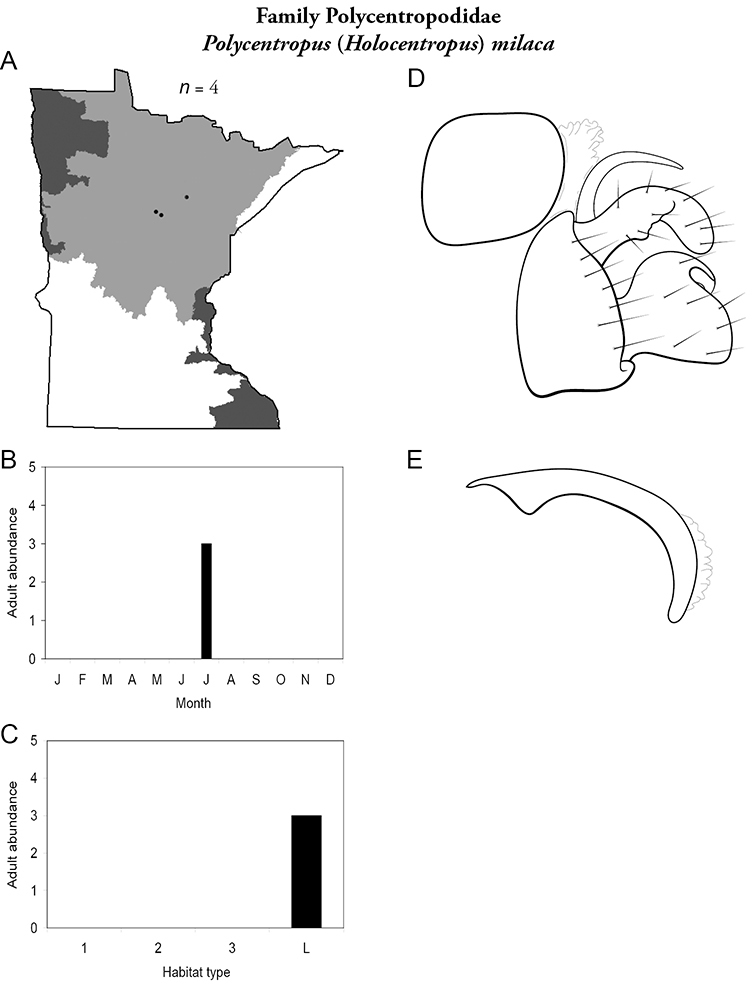
*Polycentropus milaca* **A** total specimens collected and all known collecting localities (Figure 4) **B** monthly adult abundance (1980s to present) **C** habitat preference (1980s to present) (Table 1) **D** male genital capsule **E** phallus.

***Polycentropus (Holocentropus) milaca*** (Figure 276) is known worldwide from only 4 specimens. The holotype was collected in 1965 from Link (Lynx) Lake, Itasca County, in the Northern Region (Etnier 1968). Thirty-five years later, in 2000, 3 additional specimens were found in Big Rice and Mable Lakes, Cass County, in the Northern Region (Houghton and Holzenthal 2003). These remain the only known specimens of *Polycentropus milaca* in the world. All 3 collecting sites are within 75 km of each other, and are small mesotrophic lakes with abundant littoral vegetation. All specimens were collected in July. Due to its rarity, low abundance, Minnesota endemism, and the sensitivity of its habitat, the Minnesota Department of Natural Resources has propsed “Endangered” status for *Polycentropus milaca* (MNDNR 2012).

**Figure 277.**
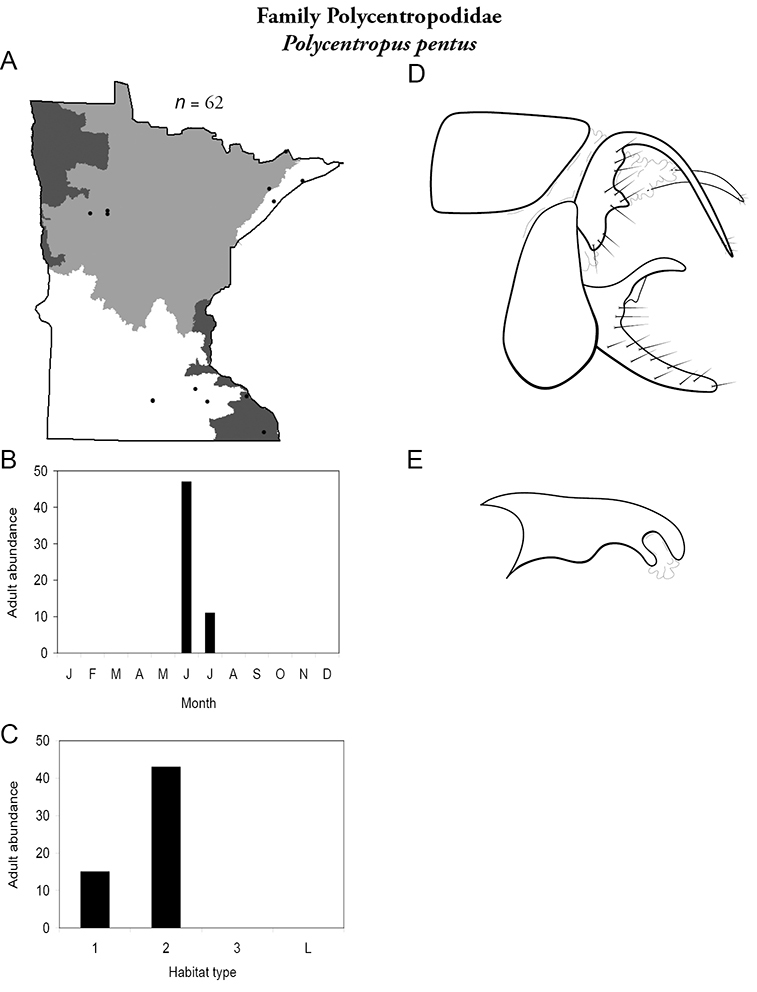
*Polycentropus pentus* **A** total specimens collected and all known collecting localities (Figure 4) **B** monthly adult abundance (1980s to present) **C** habitat preference (1980s to present) (Table 1) **D** male genital capsule **E** phallus.

***Polycentropus pentus*** (Figure 277) has been found sporadically from all regions except the Northwestern. Adults were present in June and July, and found only in small and medium rivers.

**Figure 278.**
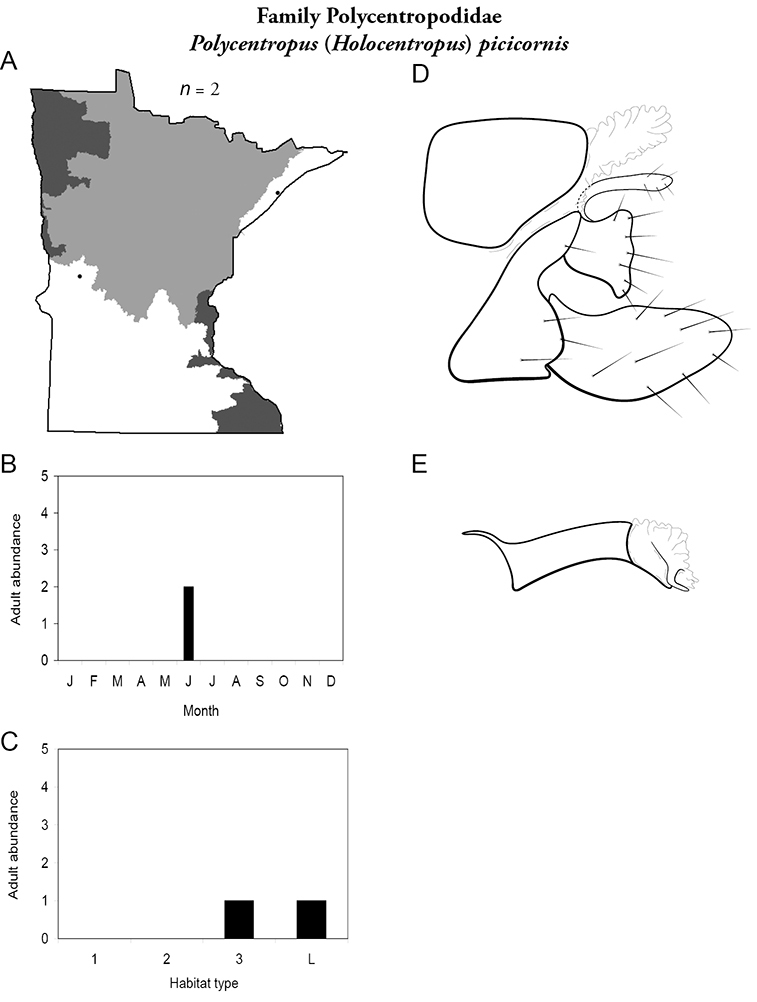
*Polycentropus picicornis* **A** total specimens collected and all known collecting localities (Figure 4) **B** monthly adult abundance (1980s to present) **C** habitat preference (1980s to present) (Table 1) **D** male genital capsule **E** phallus.

***Polycentropus (Holocentropus) picicornis*** (Figure 278) is known only from 2 specimens; 1 from a large river in the Lake Superior Region, and 1 from a lake in the Southern Region. Both specimens were found in June.

**Figure 279.**
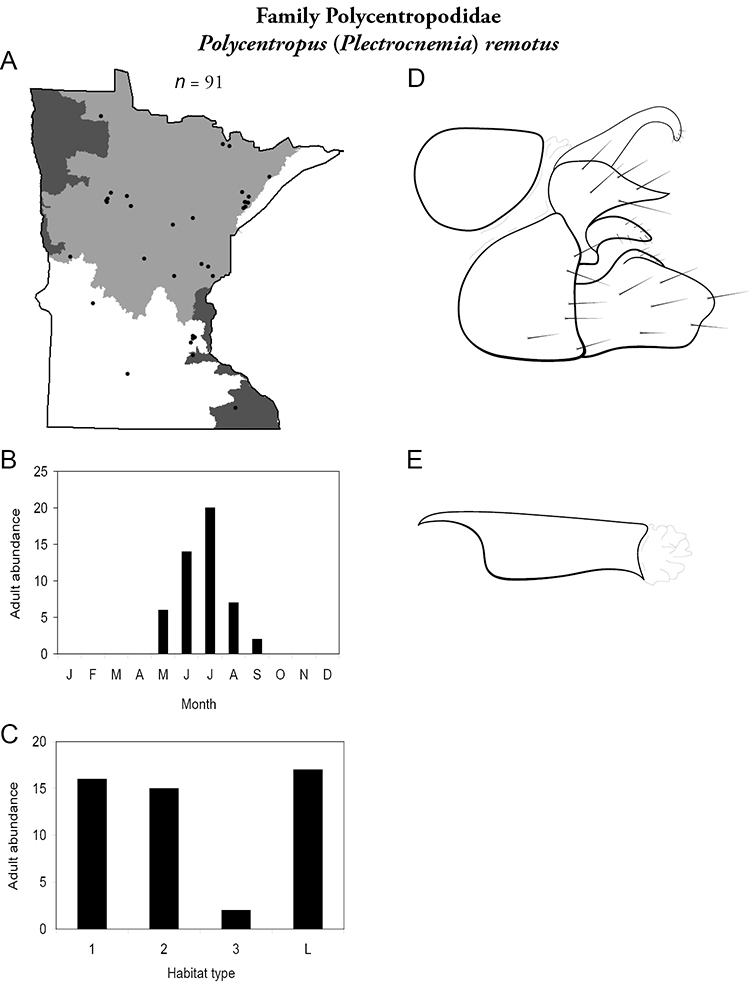
*Polycentropus remotus* **A** total specimens collected and all known collecting localities (Figure 4) **B** monthly adult abundance (1980s to present) **C** habitat preference (1980s to present) (Table 1) **D** male genital capsule **E** phallus.

***Polycentropus (Plectrocnemia) remotus*** (Figure 279) has been collected from the Northern, Southeastern, and Southern Regions. Adults were present from May through September. They were found in equal abundance in small and medium rivers, and in lakes.

**Figure 280.**
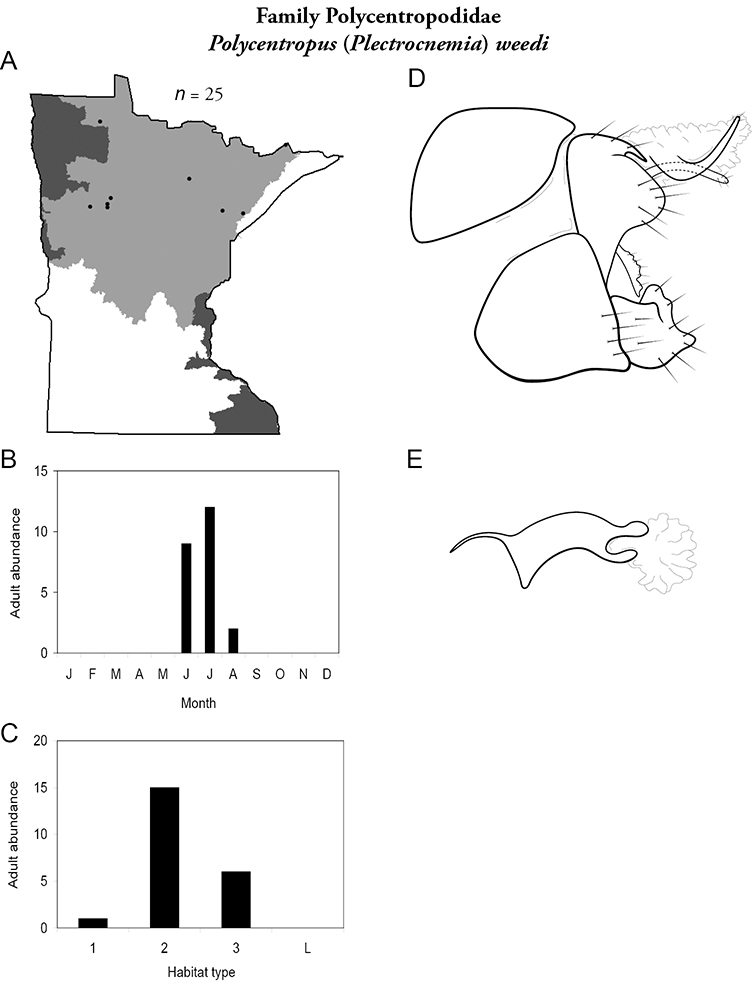
*Polycentropus weedi* **A** total specimens collected and all known collecting localities (Figure 4) **B** monthly adult abundance (1980s to present) **C** habitat preference (1980s to present) (Table 1) **D** male genital capsule **E** phallus.

***Polycentropus (Plectrocnemia) weedi*** (Figure 280) is known only from theNorthern Region, mostly from medium and large rivers. Adults were present in August, and abundant during June and July.

### Family Psychomyiidae

This family contains 2 genera in Minnesota: *Lype* and *Psychomyia*, and a total of 2 species.Both genera are 5–7 mm in length and are often confused with members of the Hydroptilidae due to this small size. Larvae are either scrapers or gathering collectors and typically found in streams (Wiggins 1996). Larvae construct fixed retreats composed of silk, or also including small organic or mineral particles. Males of both genera are infrequently collected. Illustrations of females, which are readily identifiable, are included in this manual.

### Genus *Lype*

The genus *Lype* contains a single species in North America and in Minnesota. Larvae usually inhabit small cool streams where they consume small organic particles and periphyton (Wiggins 1996). Larval retreats are usually silken tubes, sometimes incorporating detrital particles as well, and fixed on to rocks or large pieces of wood.

**Figure 281.**
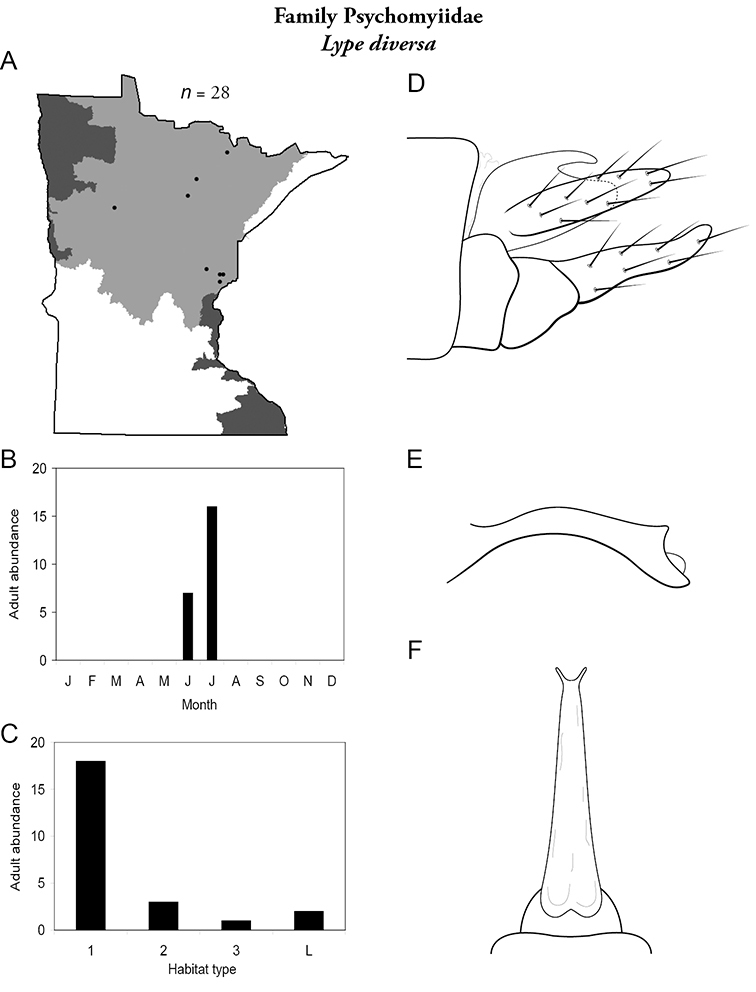
*Lype diversa* **A** total specimens collected and all known collecting localities (Figure 4) **B** monthly adult abundance (1980s to present) **C** habitat preference (1980s to present) (Table 1) **D** male genital capsule **E** phallus **F** female genital capsule (ventral view).

***Lype diversa*** (Figure 281) was sporadically collected, usually from small streams in the Northern Region during June and July. Females were far more abundant than males.

### Genus *Psychomyia*

The genus*Psychomyia* contains a single species in Minnesota. For additional species, see Armitage and Hamilton (1990). Larvae are found in all types of streams, especially larger rivers. Larvae primarily consume algae. Larval retreats are usually long, connected tubes on the surfaces of rocks (Wiggins 1996).

**Figure 282.**
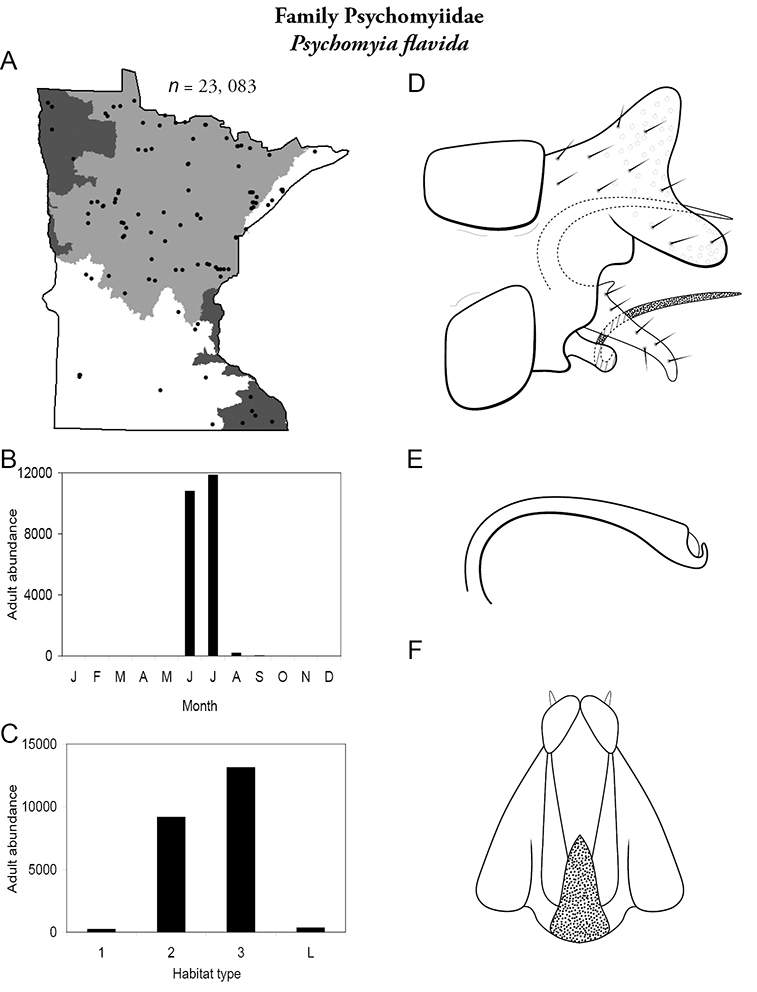
*Psychomyia flavida* **A** total specimens collected and all known collecting localities (Figure 4) **B** monthly adult abundance (1980s to present) **C** habitat preference (1980s to present) (Table 1) **D** male genital capsule **E** phallus **F** female genital capsule (ventral view).

***Psychomyia flavida*** (Figure 282) was commonly collected throughout all regions, especially the Northern. It was found primarily in medium and large rivers during June and July, with occasional specimens caught in August and September. Several collections of *Psychomyia flavida* yielded >1000 specimens, including one of nearly 10,000 specimens from the White Earth River, Mahnomen County, in the Northern Region and one of nearly 8,000 specimens from the Redwood River, Lyon County, in the Southern Region. Due in large part to this extreme abundance at specific sites, *Psychomyia flavida* was the most abundant species in large rivers of the Northern Region, and the single most abundant species overall in Minnesota, with over 23,000 specimens examined (Table 4, Figure 9). Interestingly, >99% of these specimens were female. In fact, the large collections noted above were both entirely composed of females. Male *Psychomyia flavida* specimens were only located in the extreme northeastern portion of the Northern Region. Males are far more common in other states and are readily attracted to lights (e.g., Moulton and Stewart 1996, Houghton 2001), suggesting that the populations in Minnesota may be parthenogenetic.

### Family Rhyacophilidae

This family contains a single genus, *Rhyacophila*, in Minnesota, and a total of 3 species. Larvae typically inhabit cold, fast-moving streams. They are unique among caddisflies in that they do not construct a case, instead remaining free-living until pupation (Wiggins 1996). Most species are predatory, although they may be herbivores during early instars. Adults are black or dark brown, without notable patterning on the wings. They range 14–18 mm in length.

### Genus *Rhyacophila*

The genus *Rhyacophila* contains3 species in Minnesota. All are restricted to the Lake Superior Region and only 1 is frequently encountered there. For additional species, see Schmid (1970).

**Figure 283.**
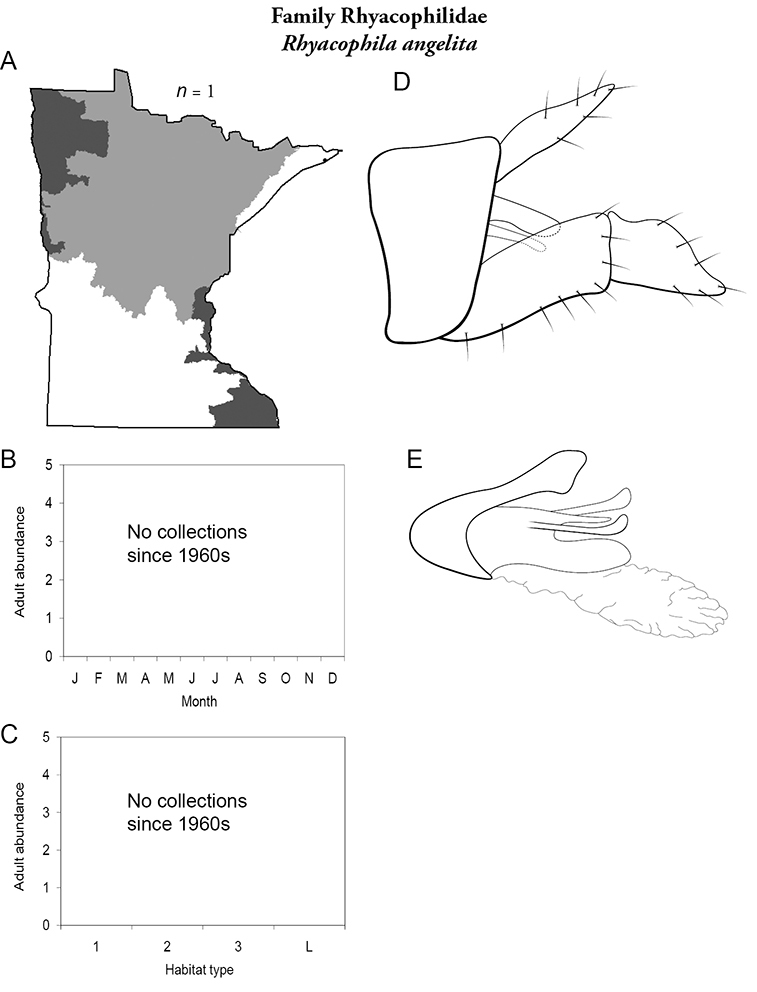
*Rhyacophila angelita* **A** total specimens collected and all known collecting localities (Figure 4) **B** monthly adult abundance (1980s to present) **C** habitat preference (1980s to present) (Table 1) **D** male genital capsule **E** phallus.

***Rhyacophila angelita***(Figure 283) is known only from a single specimen collected during July 1965 from the city of Hovland in the Lake Superior Region. It has not been collected since, and it is difficult to know if the species has been extirpated or is simply difficult to collect.

**Figure 284.**
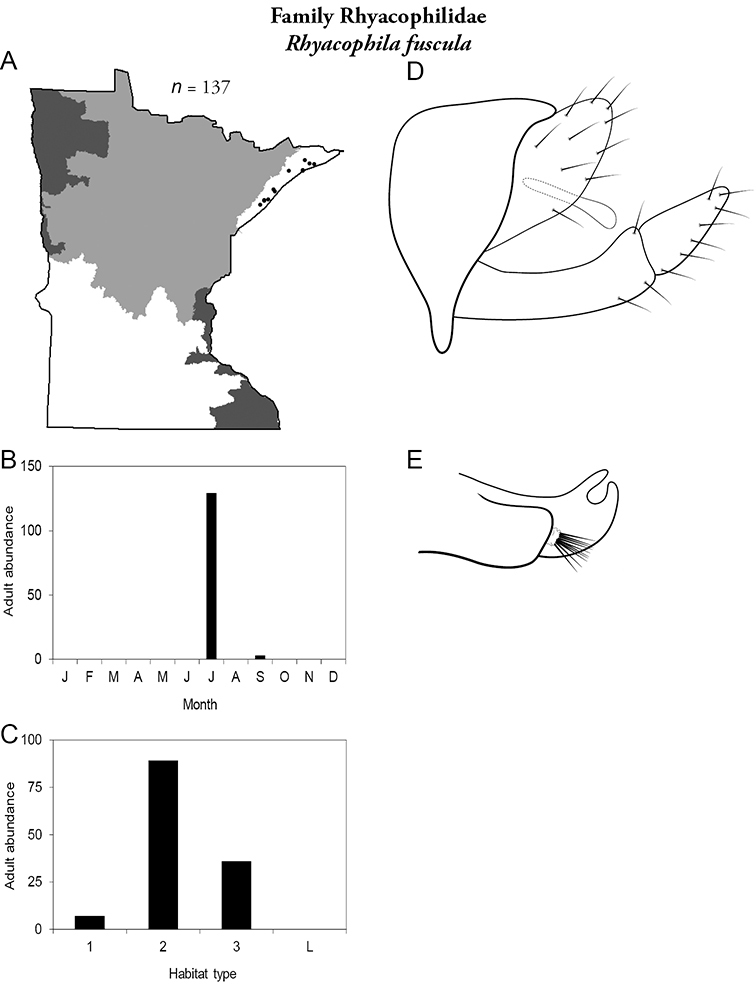
*Rhyacophila fuscula* **A** total specimens collected and all known collecting localities (Figure 4) **B** monthly adult abundance (1980s to present) **C** habitat preference (1980s to present) (Table 1) **D** male genital capsule **E** phallus.

***Rhyacophila fuscula***(Figure 284) was commonly collected throughout the Lake Superior Region from all sizes of streams, especially medium rivers. The majority of adults were present in July.

**Figure 285.**
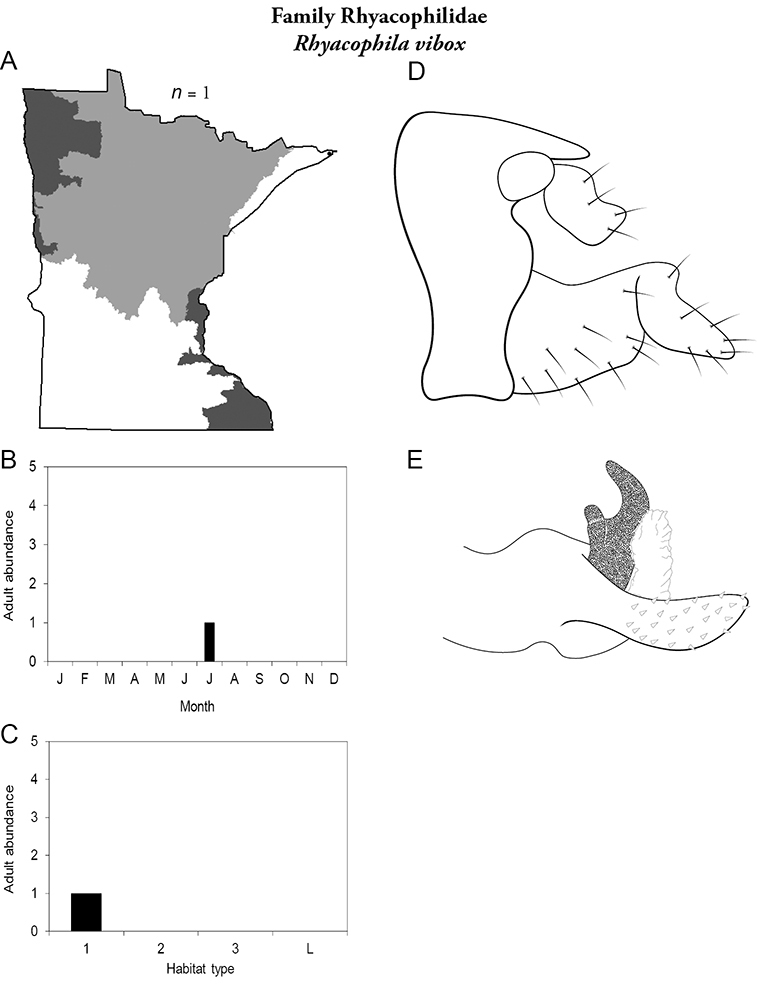
*Rhyacophila vibox* **A** total specimens collected and all known collecting localities (Figure 4) **B** monthly adult abundance (1980s to present) **C** habitat preference (1980s to present) (Table 1) **D** male genital capsule **E** phallus.

***Rhyacophila vibox***(Figure 285) is only known from a single specimen collected from Poplar Creek, Cook County, in the Lake Superior Region during June 2000.

### Family Sericostomatidae

This family contains one genus in Minnesota, *Agarodes*, and a single species. For additional species, see Keth and Harris (2008). Larvae are typically burrowing detritivores, found in the sand and gravel of lakes and streams (Wiggins 1996). Larval cases are composed of small uniform mineral particles.

### Genus *Agarodes*

The genus *Agarodes* contains a single species in Minnesota. Adults are grey in color and range 8–12 mm. Males often have unusual secondary sexual characteristics, most notably enlargement and increased setation of the antennal scapes and maxillary palpi.

**Figure 286.**
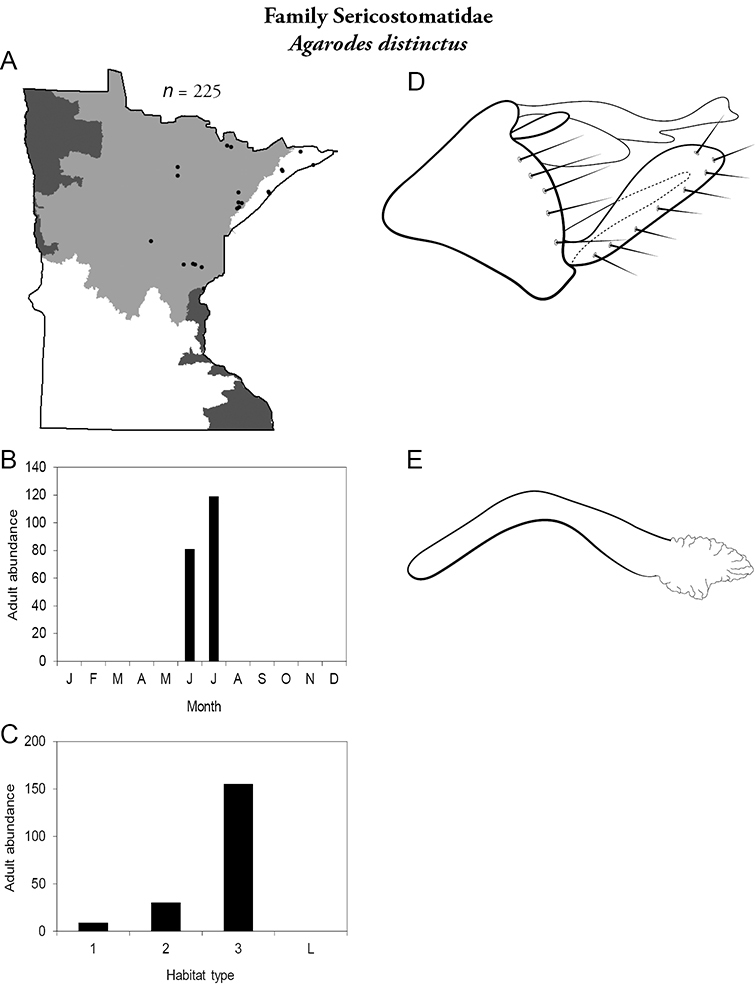
*Agarodes distinctus* **A** total specimens collected and all known collecting localities (Figure 4) **B** monthly adult abundance (1980s to present) **C** habitat preference (1980s to present) (Table 1) **D** male genital capsule **E** phallus.

***Agarodes distinctus*** (Figure 286) has been collected in June and July, mostly from large rivers. It is known only from the Lake Superior and Northern Regions.

### Family Uenoidae

This family contains a single genus in Minnesota, *Neophylax*, and a total of 3 species. One recent classification placed *Neophylax* in the family Thremmatidae (Vshivkova et al. 2007). Larvae are found in fast-moving areas of different types of streams. They construct a case of sand and small stones and consume periphyton from the surfaces of medium and large rocks (Wiggins 1996). Larvae typically finish maturing in June or early July, and then spend the remainder of the summer in a diapausing state. Often, dense aggregations of these diapausing larvae can be found on the undersides of large rocks, or else buried in shallow sand. Adults emerge in August and September and are often present into October, although it may be too cold for specimens to fly at night this late in the year (e.g., Houghton et al. 2011b). Adults range 8–12 mm in length. Wings are typically brown and often have bright orange reticulations (Figure 294).

### Genus *Neophylax*

The genus *Neophylax* contains 3 species in Minnesota. Due to the difficulty of collecting night flying adults during the typically cool autumn evenings, all 3 are probably more widespread than their known distributions suggest.

**Figure 287.**
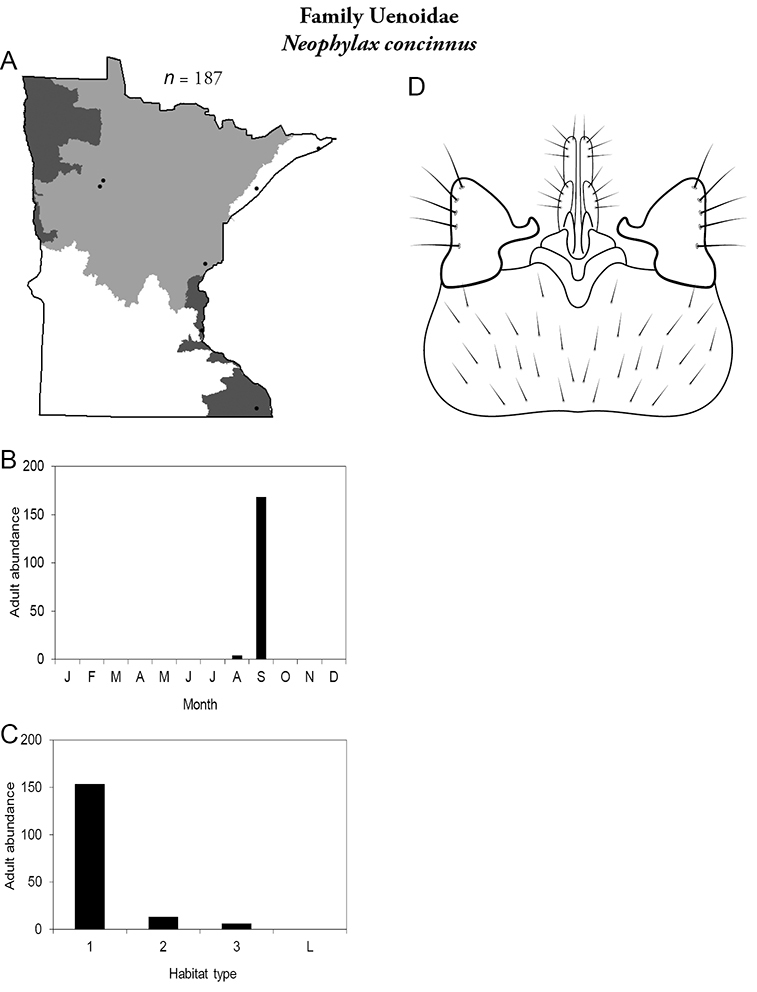
*Neophylax concinnus* **A** total specimens collected and all known collecting localities (Figure 4) **B** monthly adult abundance (1980s to present) **C** habitat preference (1980s to present) (Table 1) **D** male genital capsule (ventral view).

***Neophylax concinnus*** (Figure 287) has been collected from the Lake Superior, Northern, and Southeastern Regions. Adults were collected mainly in September and were abundant on warm evenings. It was collected almost exclusively from small streams.

**Figure 288.**
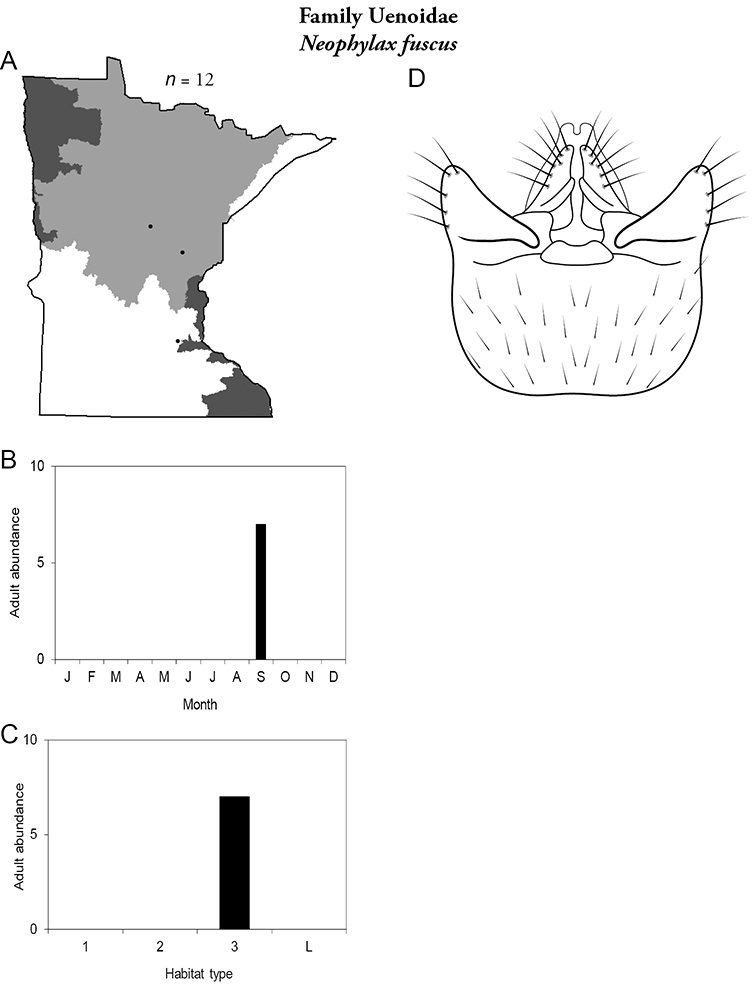
*Neophylax fuscus* **A** total specimens collected and all known collecting localities (Figure 4) **B** monthly adult abundance (1980s to present) **C** habitat preference (1980s to present) (Table 1) **D** male genital capsule (ventral view).

***Neophylax fuscus*** (Figure 288) is mainly known from the Northern Region and exclusively from large rivers. Adults were collected in September.

**Figure 289.**
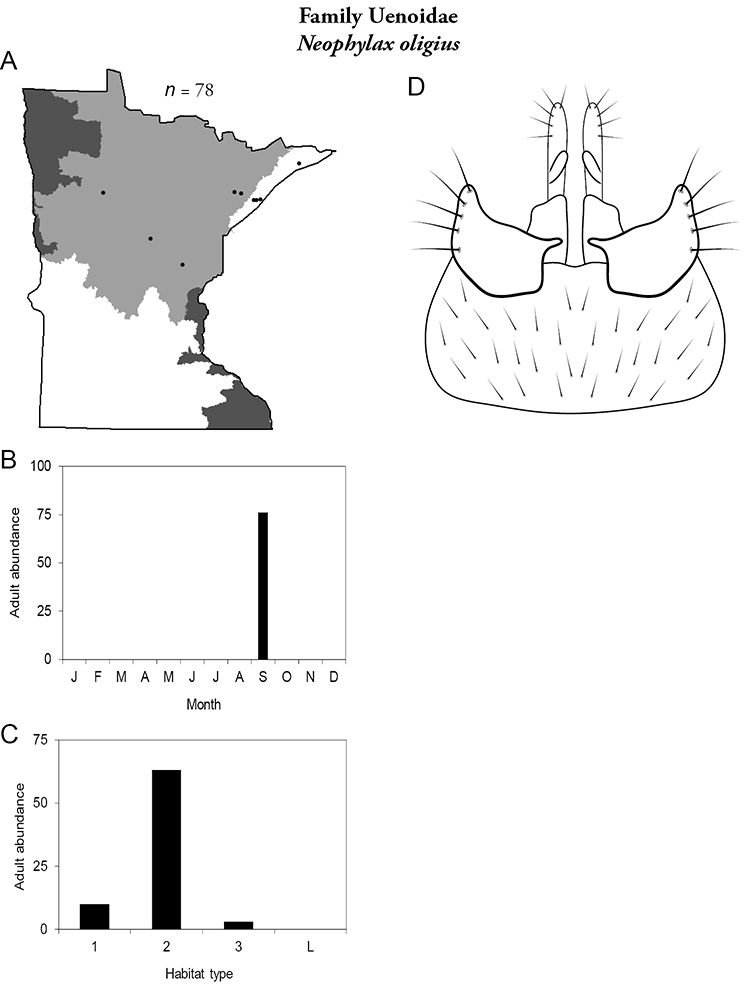
*Neophylax oligius* **A** total specimens collected and all known collecting localities (Figure 4) **B** monthly adult abundance (1980s to present) **C** habitat preference (1980s to present) (Table 1) **D** male genital capsule (ventral view).

***Neophylax oligius*** (Figure 289) is known from the Lake Superior and Northern Regions. Adults were collected only in September and typically from medium rivers.

Another species of *Neophylax*, *Neophylax nacatus*, was reported from Minnesota from a larval specimen (Lager et al. 1979). The whereabouts of this specimen is not known. No adults have been collected from Minnesota. Thus, the species is not included in this manual.

## Color plates

**Figure 290.**
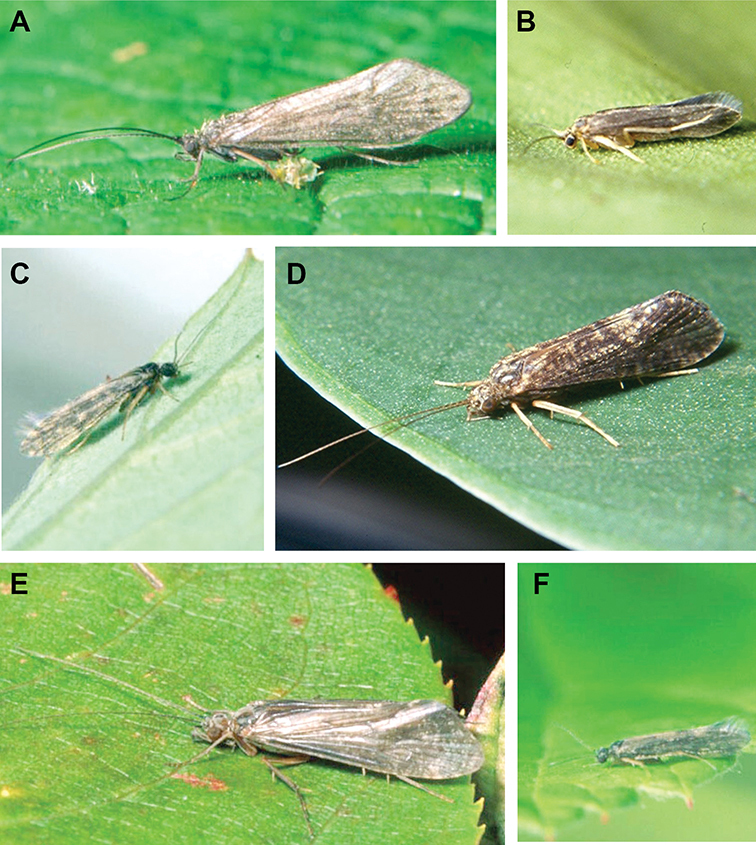
Adult specimens of **A** *Apatania zonella* (Apataniidae) **B** *Protoptila maculata* (Glossosomatidae) **C** *Agraylea multupunctata* (Hydroptilidae) **D** *Hydropsyche simulans* (Hydropsychidae) **E** *Hydropsyche betteni* (Hydropsychidae) **F** *Oxyethira forcipata* (Hydroptilidae).

**Figure 291.**
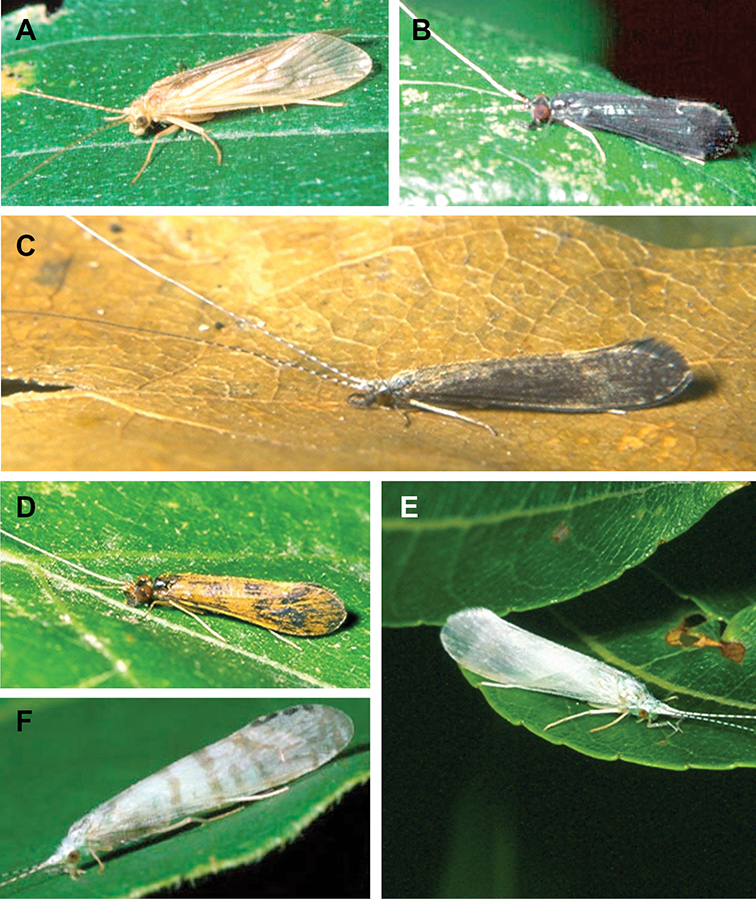
Adult specimens of **A** *Potamyia flava* (Hydropsychidae) **B** *Mystacides sepulchralis* (Leptoceridae) **C** *Leptocerus americanus* (Leptoceridae) **D** *Mystacides interjecta* (Leptoceridae) **E** *Nectopsychidae candida* (Leptoceridae) **F** *Nectopsyche exquisita* (Leptoceridae).

**Figure 292.**
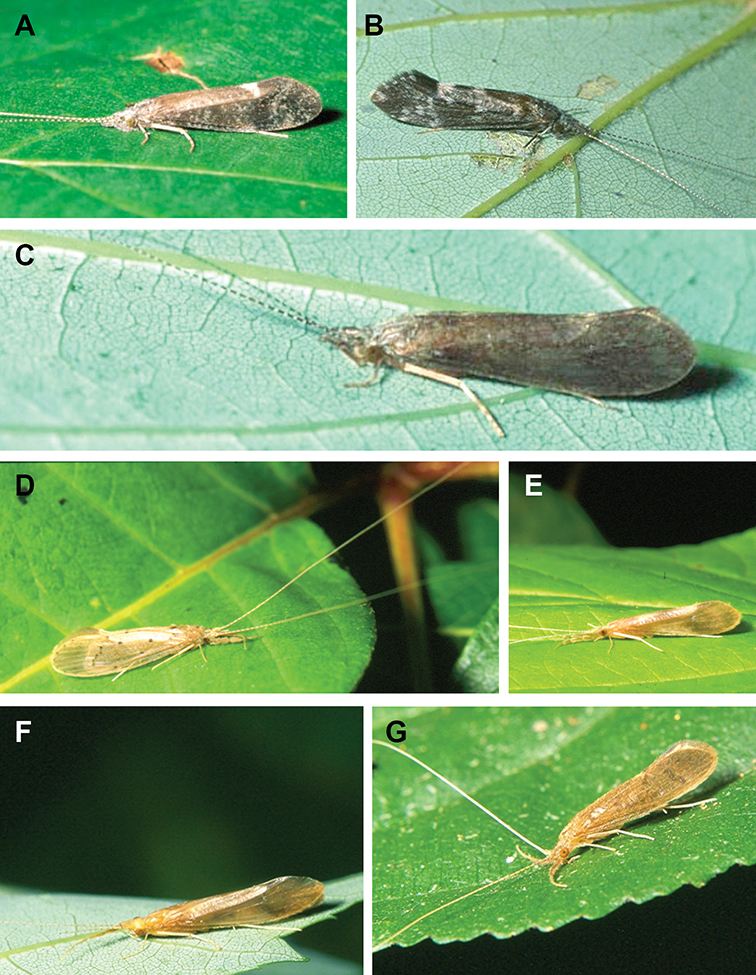
Adult specimens of **A** *Ceraclea cencellata* (Leptoceridae) **B** *Ceraclea transversa* (Leptoceridae) **C** *Ceraclea maculata* (Leptoceridae) **D** *Oecetis avara* (Leptoceridae) **E** *Triaenodes marginata* (Leptoceridae) **F** *Triaenodes tarda* (Leptoceridae) **G** *Oecetis inconspicua* (Leptoceridae).

**Figure 293.**
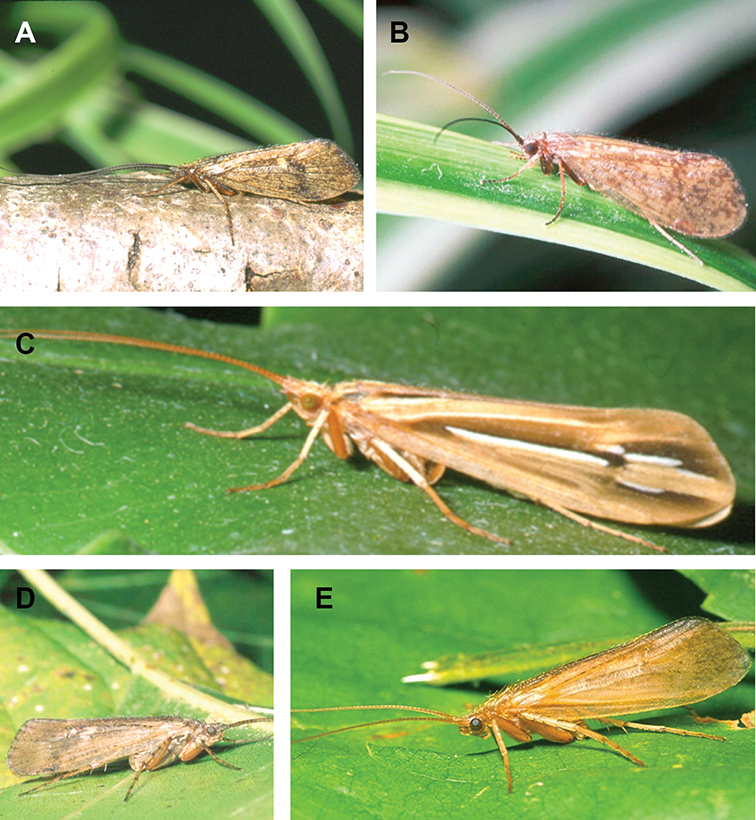
Adult specimens of **A** *Asynarchus rossi* (Limnephilidae) **B** *Frenesia missa* (Limnephilidae) **C** *Hesperophylax designatus* (Limnephilidae) **D** *Limnephilus submonifer* (Limnephilidae) **E** *Limnephilus infernalis* (Limnephilidae).

**Figure 294.**
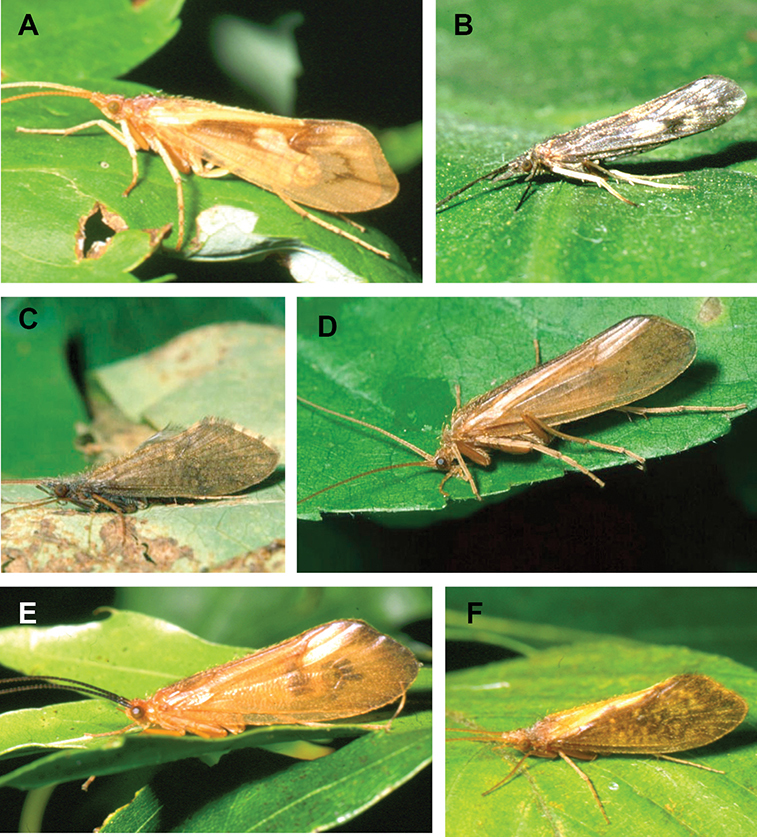
Adult specimens of **A** *Platycentropus radiatus* (Limnephilidae) **B** *Molanna uniophila* (Molannidae) **C** *Neophylax fuscus* (Uenoidae) **D** *Limnephilus indivisus* (Limnephilidae) **E** *Pycnopsyche guttifer* (Limnephilidae) **F** *Neophylax oligius* (Uenoidae).

**Figure 295.**
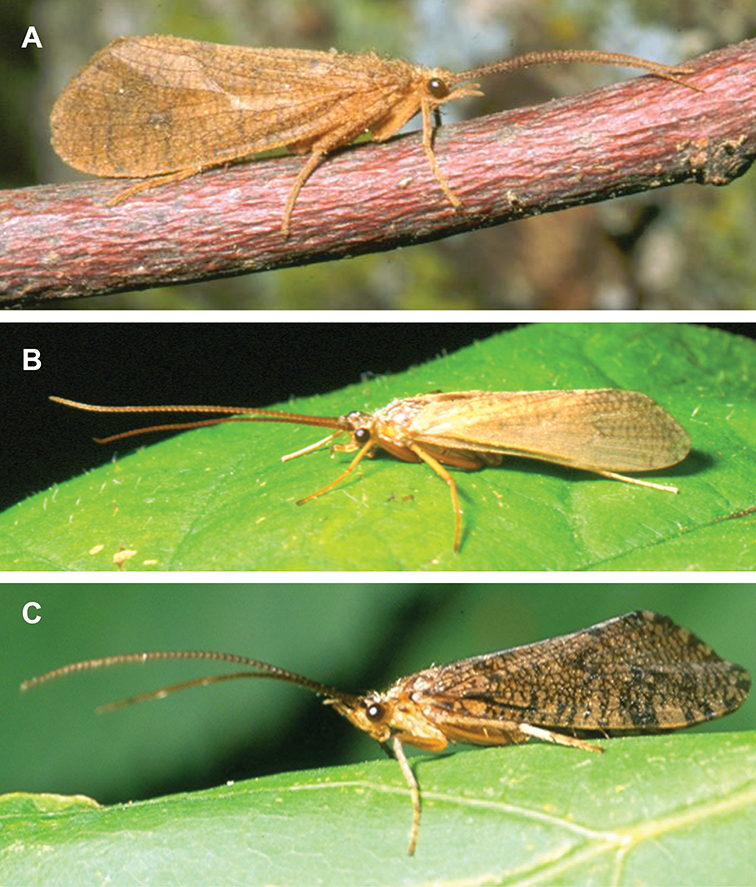
Adult specimens of **A** *Ptilostomis ocellifera* (Phryganeidae) **B** *Agrypnia straminea* (Phryganeidae) **C** *Banksiola crotchi* (Phryganeidae).
